# Supplementary figures and images for: Advancing image segmentation with DBO-Otsu: Addressing rubber tree diseases through enhanced threshold techniques (part 1 of 7)
Source: PLoS One. 2024 Mar 21;19(3):e0297284. doi: 10.1371/journal.pone.0297284 (PMC10956860; doi:10.1371/journal.pone.0297284)

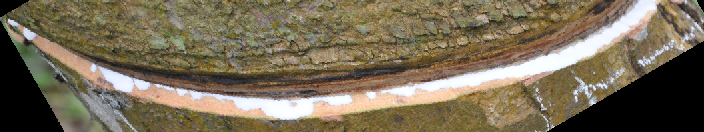

Supplement: S1 Data — (ZIP) [file pone.0297284.s001.zip › Level 1 Original Sample/1.1.jpg]

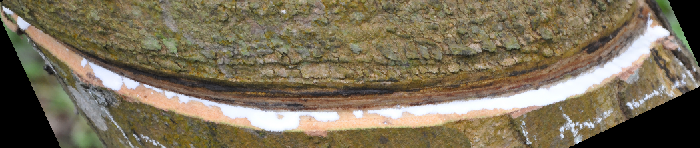

Supplement: S1 Data — (ZIP) [file pone.0297284.s001.zip › Level 1 Original Sample/1-1.jpg]

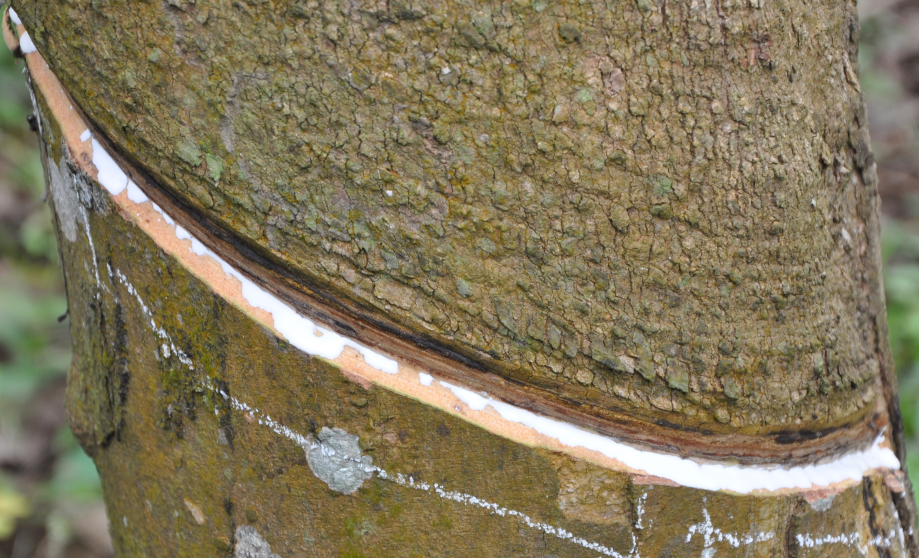

Supplement: S1 Data — (ZIP) [file pone.0297284.s001.zip › Level 1 Original Sample/1-1.png]

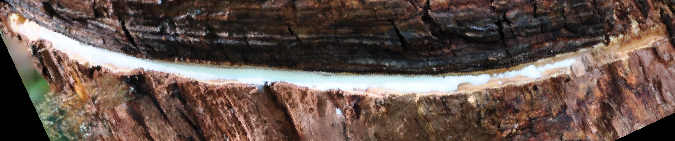

Supplement: S1 Data — (ZIP) [file pone.0297284.s001.zip › Level 1 Original Sample/1-2.jpg]

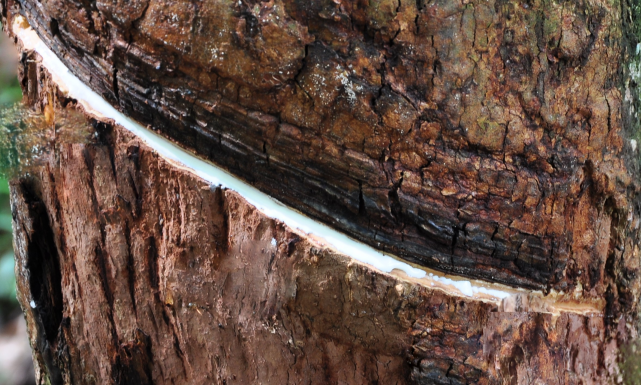

Supplement: S1 Data — (ZIP) [file pone.0297284.s001.zip › Level 1 Original Sample/1-2.png]

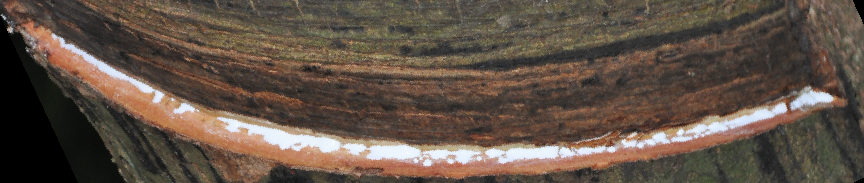

Supplement: S1 Data — (ZIP) [file pone.0297284.s001.zip › Level 1 Original Sample/1-3.jpg]

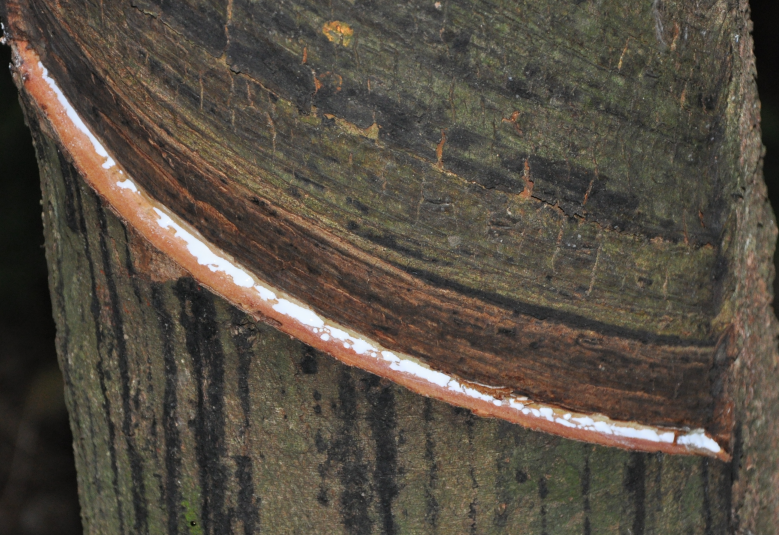

Supplement: S1 Data — (ZIP) [file pone.0297284.s001.zip › Level 1 Original Sample/1-3.png]

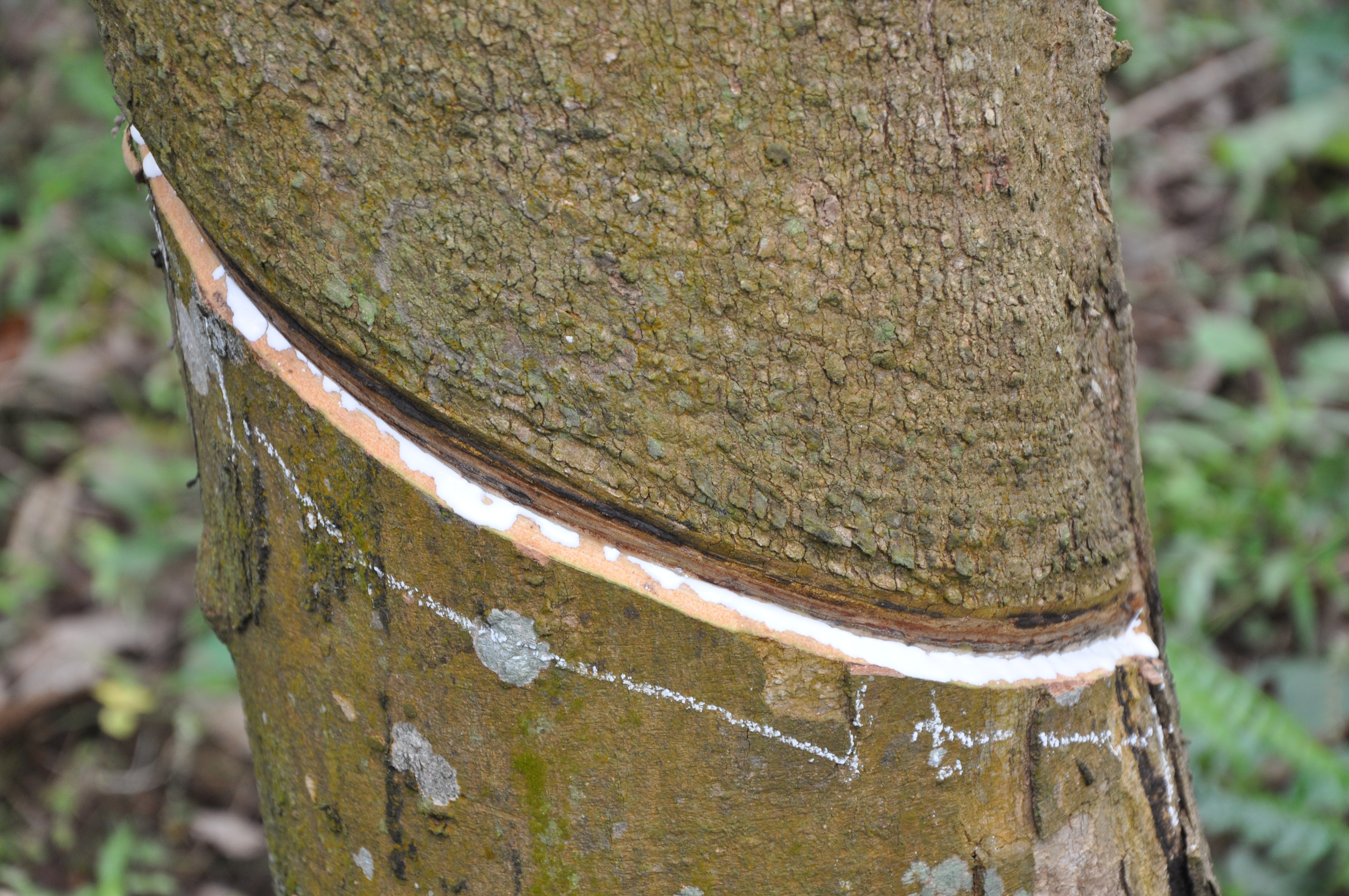

Supplement: S1 Data — (ZIP) [file pone.0297284.s001.zip › Level 1 Original Sample/1-33702-21-20141126-0089.JPG]

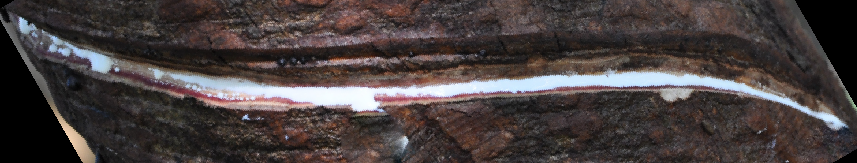

Supplement: S1 Data — (ZIP) [file pone.0297284.s001.zip › Level 1 Original Sample/1-4.jpg]

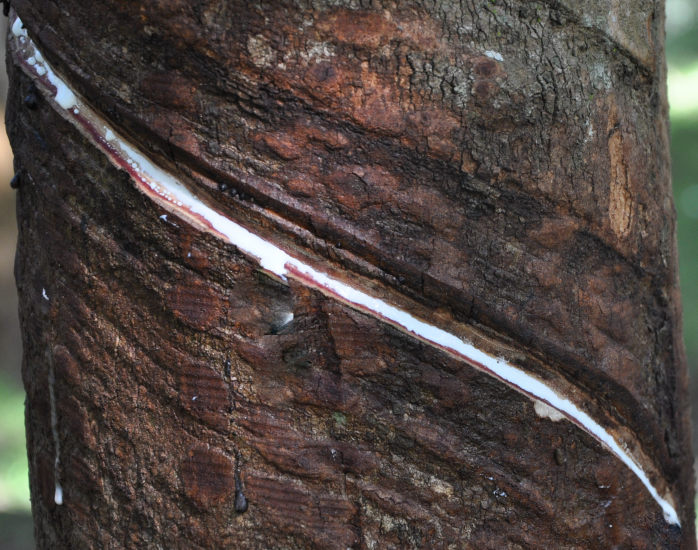

Supplement: S1 Data — (ZIP) [file pone.0297284.s001.zip › Level 1 Original Sample/1-4.png]

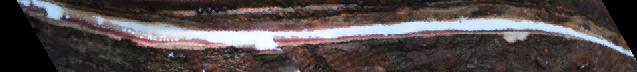

Supplement: S1 Data — (ZIP) [file pone.0297284.s001.zip › Level 1 Original Sample/1-5.jpg]

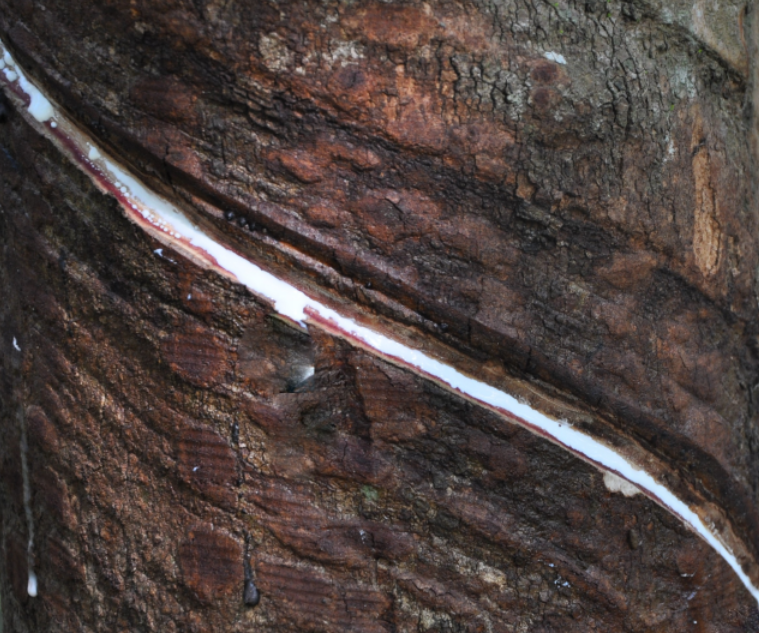

Supplement: S1 Data — (ZIP) [file pone.0297284.s001.zip › Level 1 Original Sample/1-5.png]

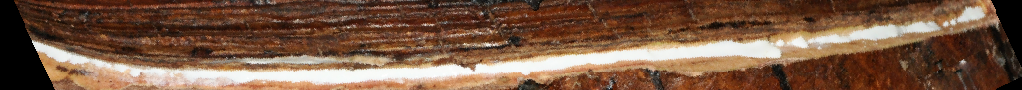

Supplement: S1 Data — (ZIP) [file pone.0297284.s001.zip › Level 1 Original Sample/1-6.jpg]

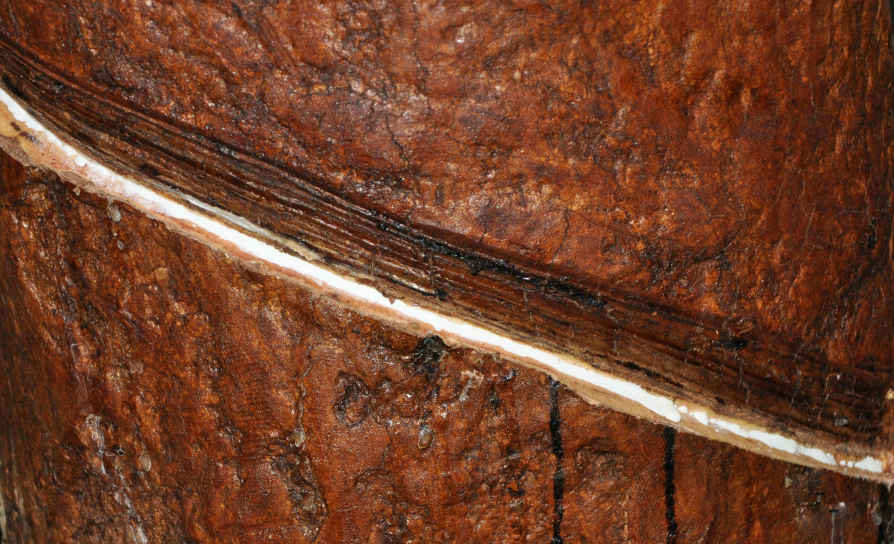

Supplement: S1 Data — (ZIP) [file pone.0297284.s001.zip › Level 1 Original Sample/1-6.png]

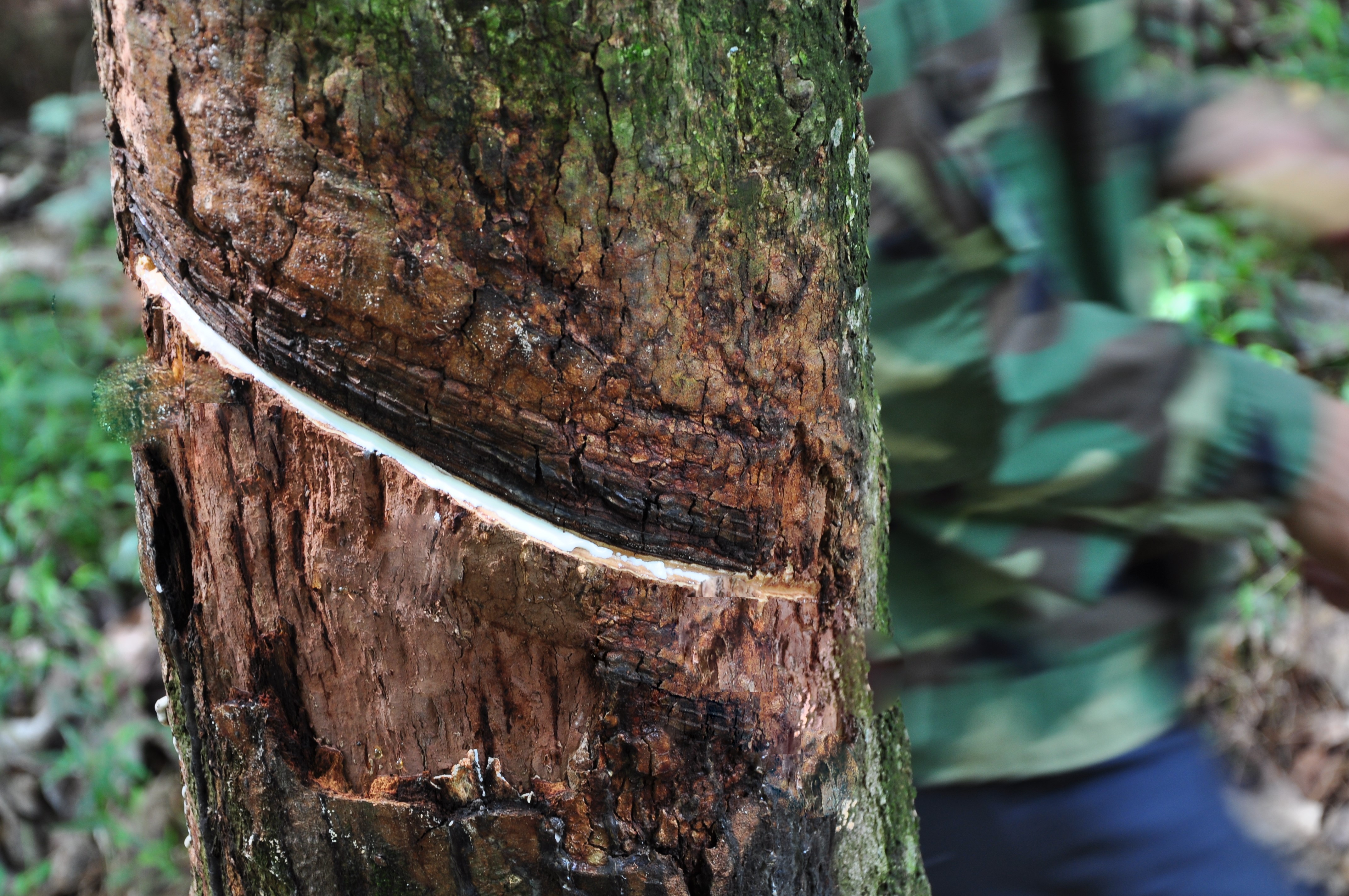

Supplement: S1 Data — (ZIP) [file pone.0297284.s001.zip › Level 1 Original Sample/1-60602-58-20140901-0379.JPG]

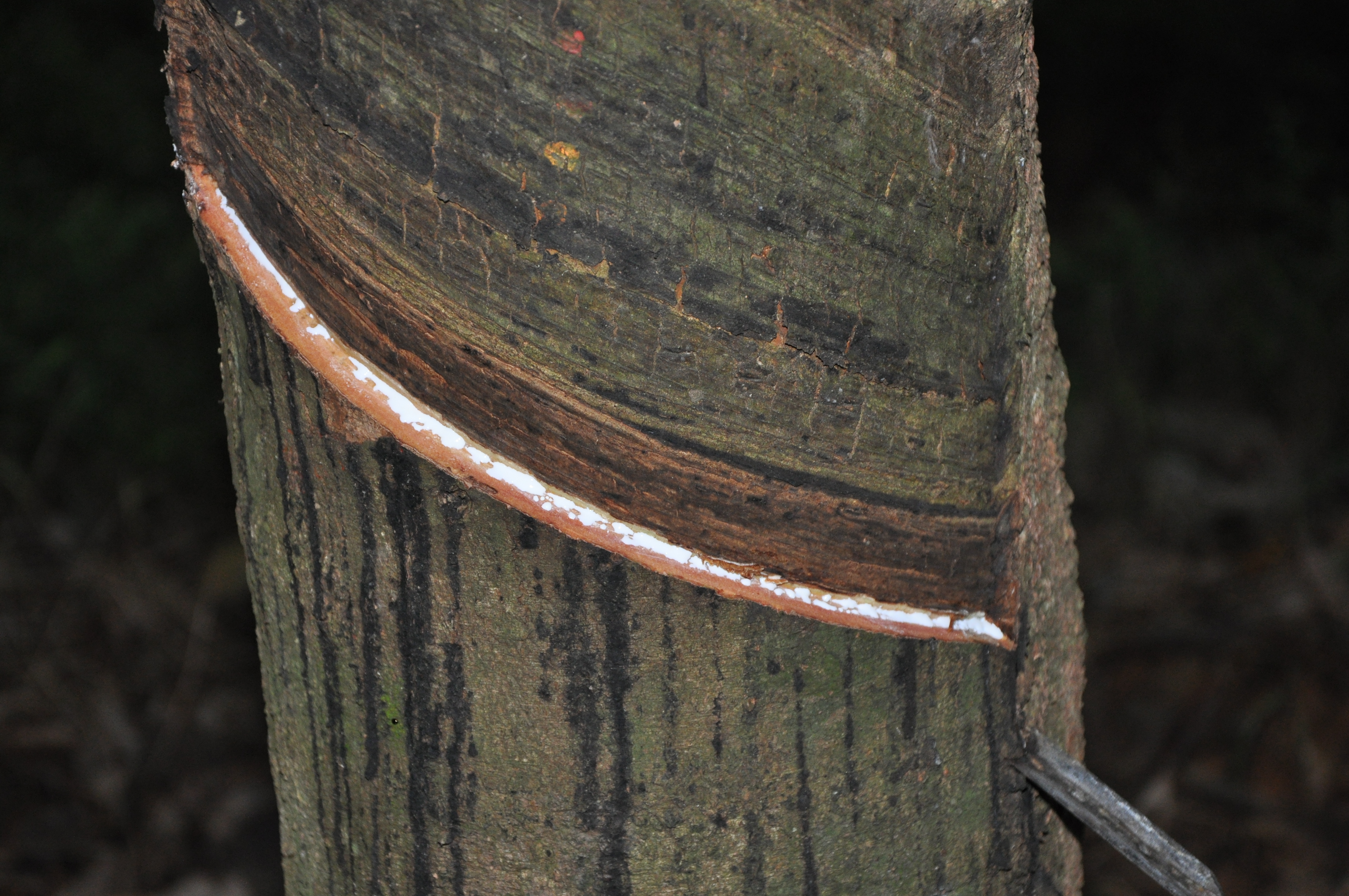

Supplement: S1 Data — (ZIP) [file pone.0297284.s001.zip › Level 1 Original Sample/1-61002-129-20140902-0027.JPG]

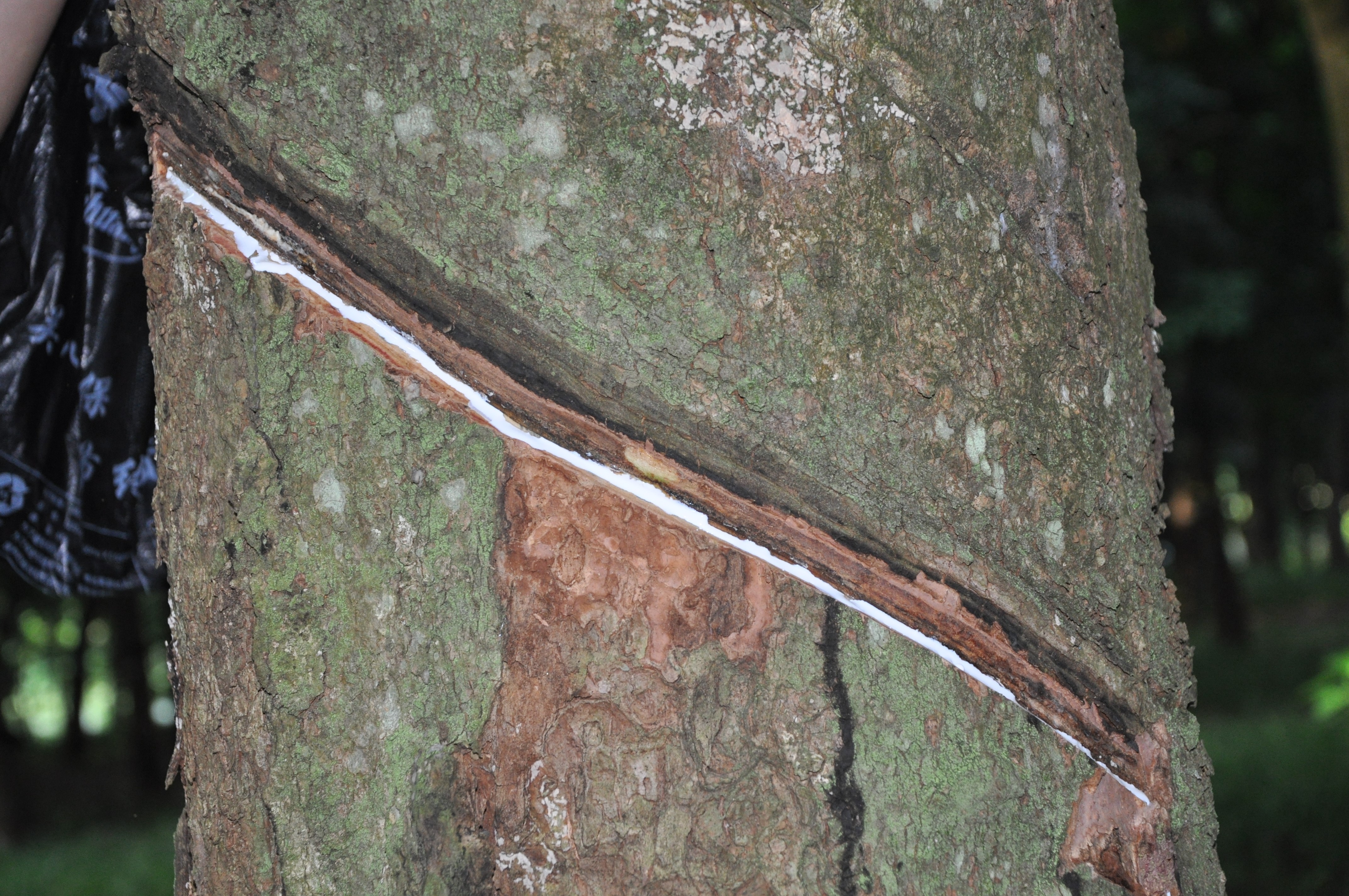

Supplement: S1 Data — (ZIP) [file pone.0297284.s001.zip › Level 1 Original Sample/1-61601-064-20150803-0064.JPG]

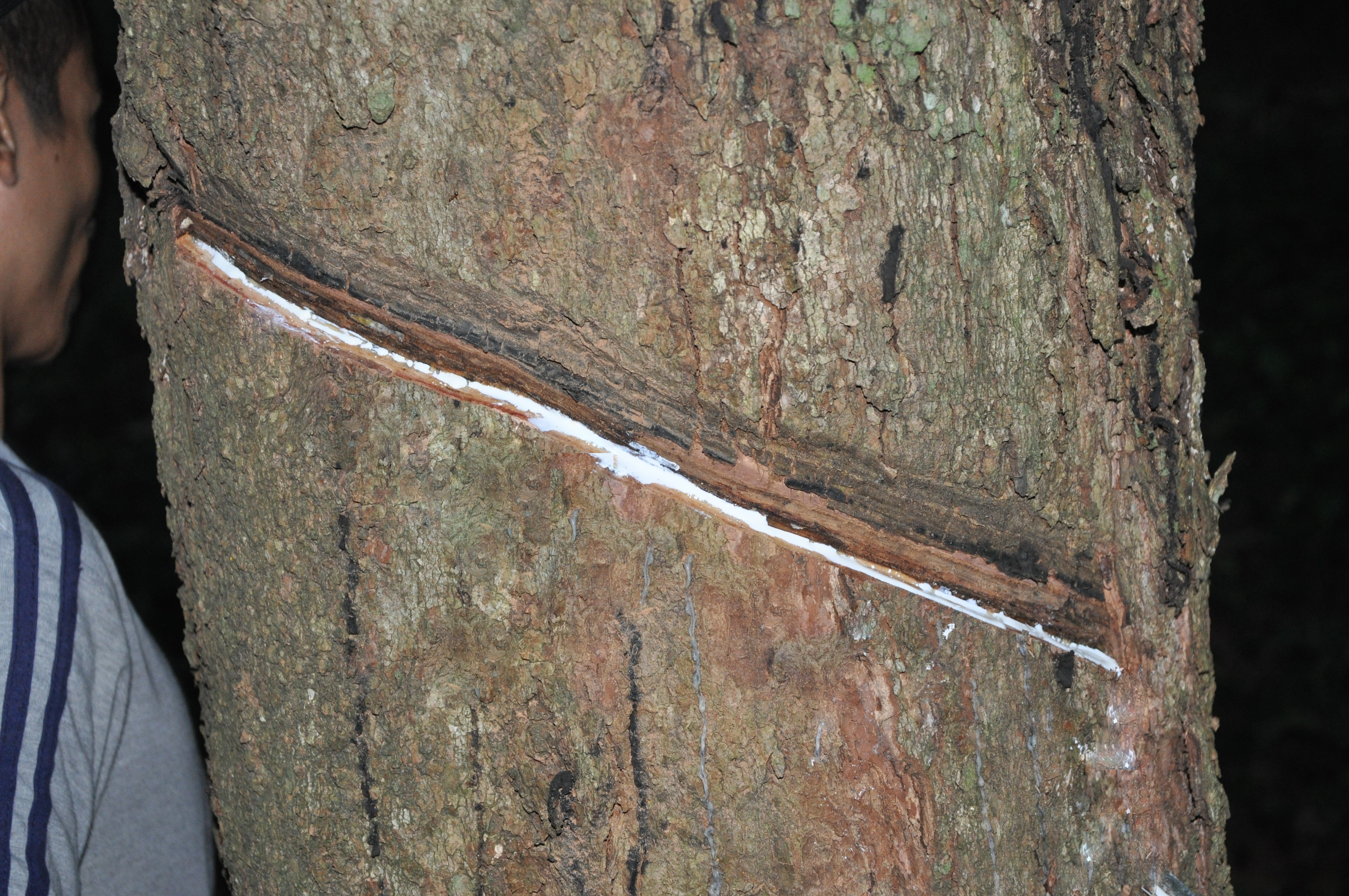

Supplement: S1 Data — (ZIP) [file pone.0297284.s001.zip › Level 1 Original Sample/1-61601-481-20150803-0481.JPG]

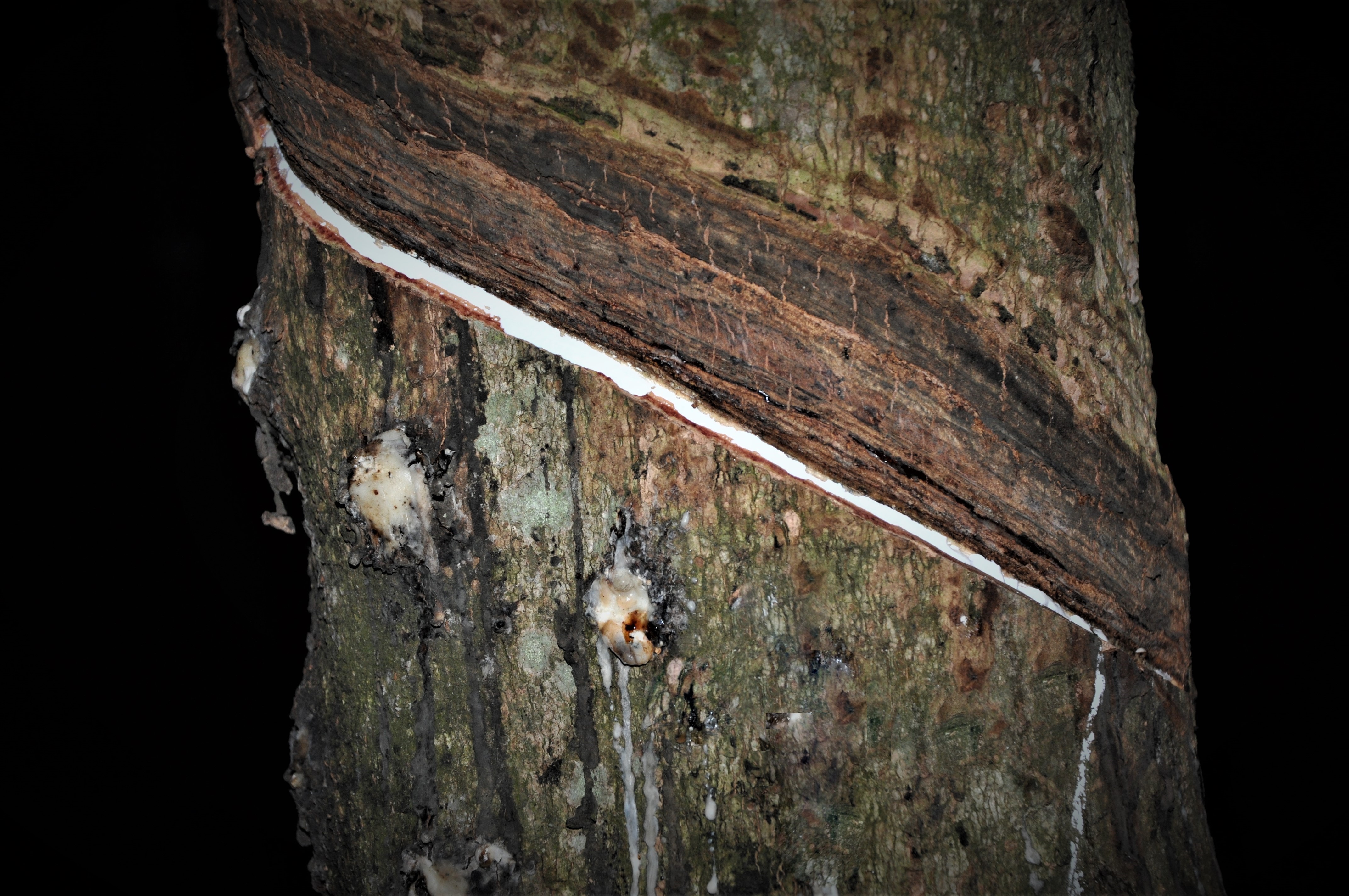

Supplement: S1 Data — (ZIP) [file pone.0297284.s001.zip › Level 1 Original Sample/1-61602-207-20141028-0047.JPG]

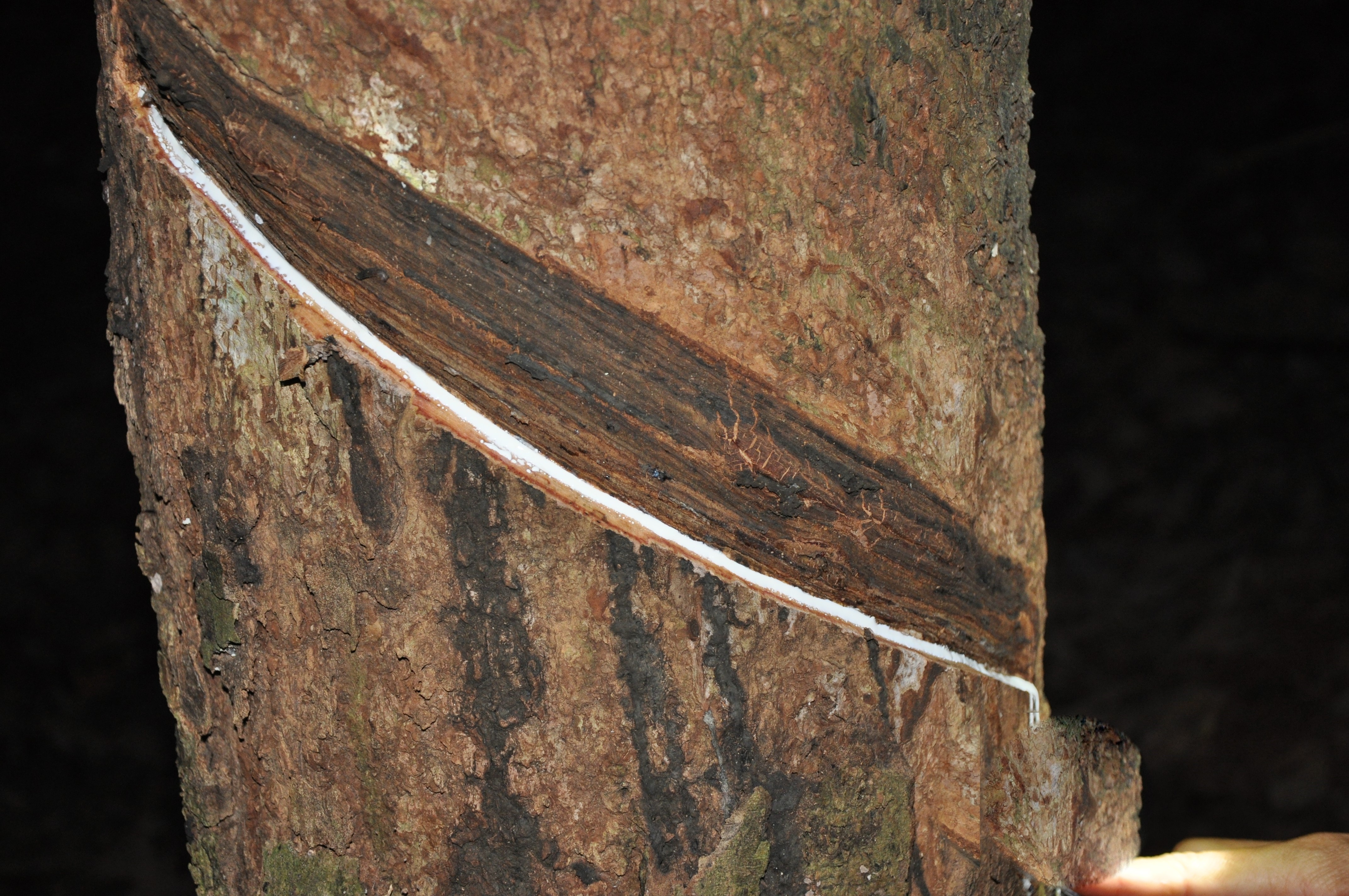

Supplement: S1 Data — (ZIP) [file pone.0297284.s001.zip › Level 1 Original Sample/1-61602-304-20141119-0079.JPG]

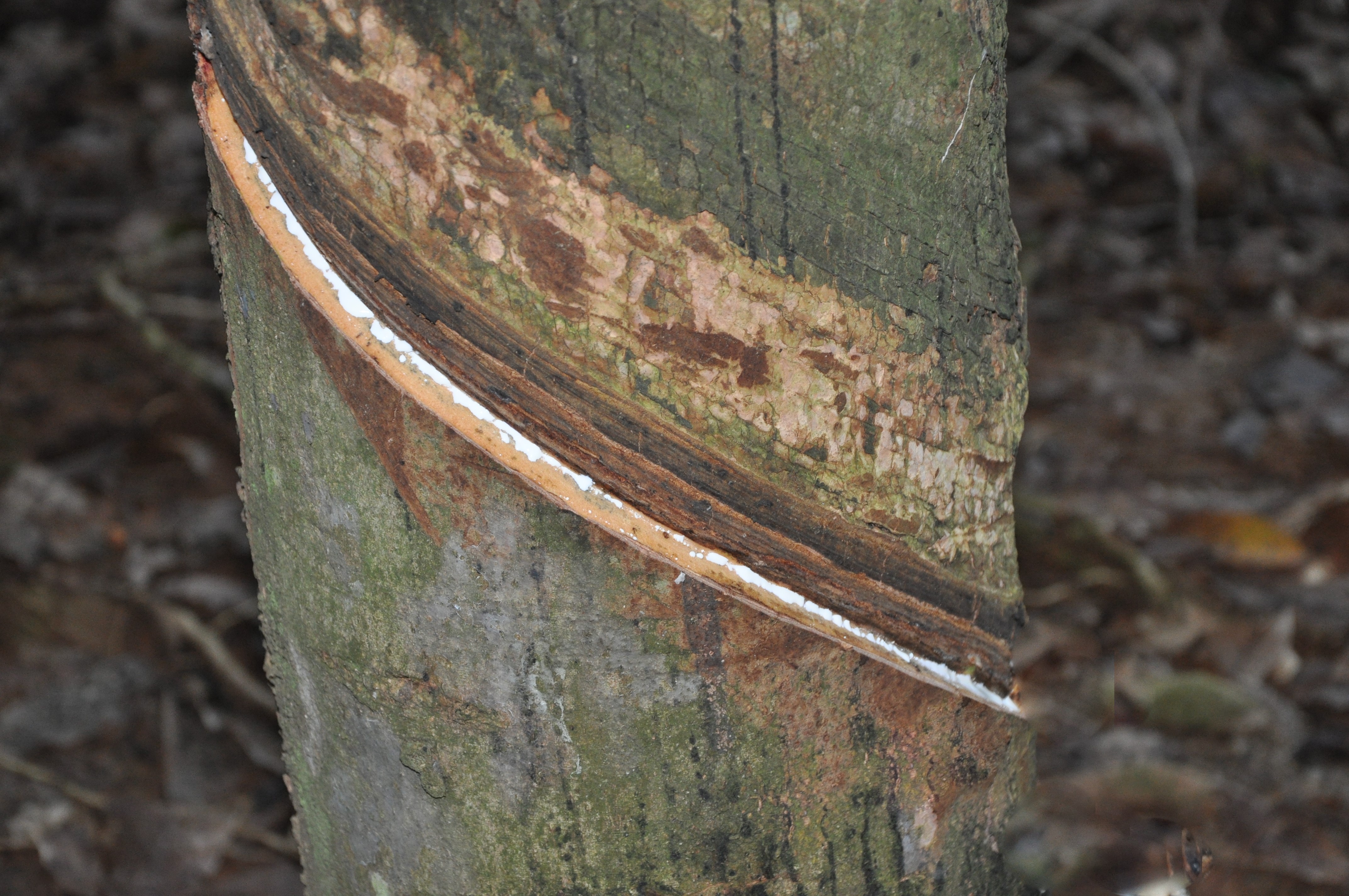

Supplement: S1 Data — (ZIP) [file pone.0297284.s001.zip › Level 1 Original Sample/1-61602-361-20141028-0149.JPG]

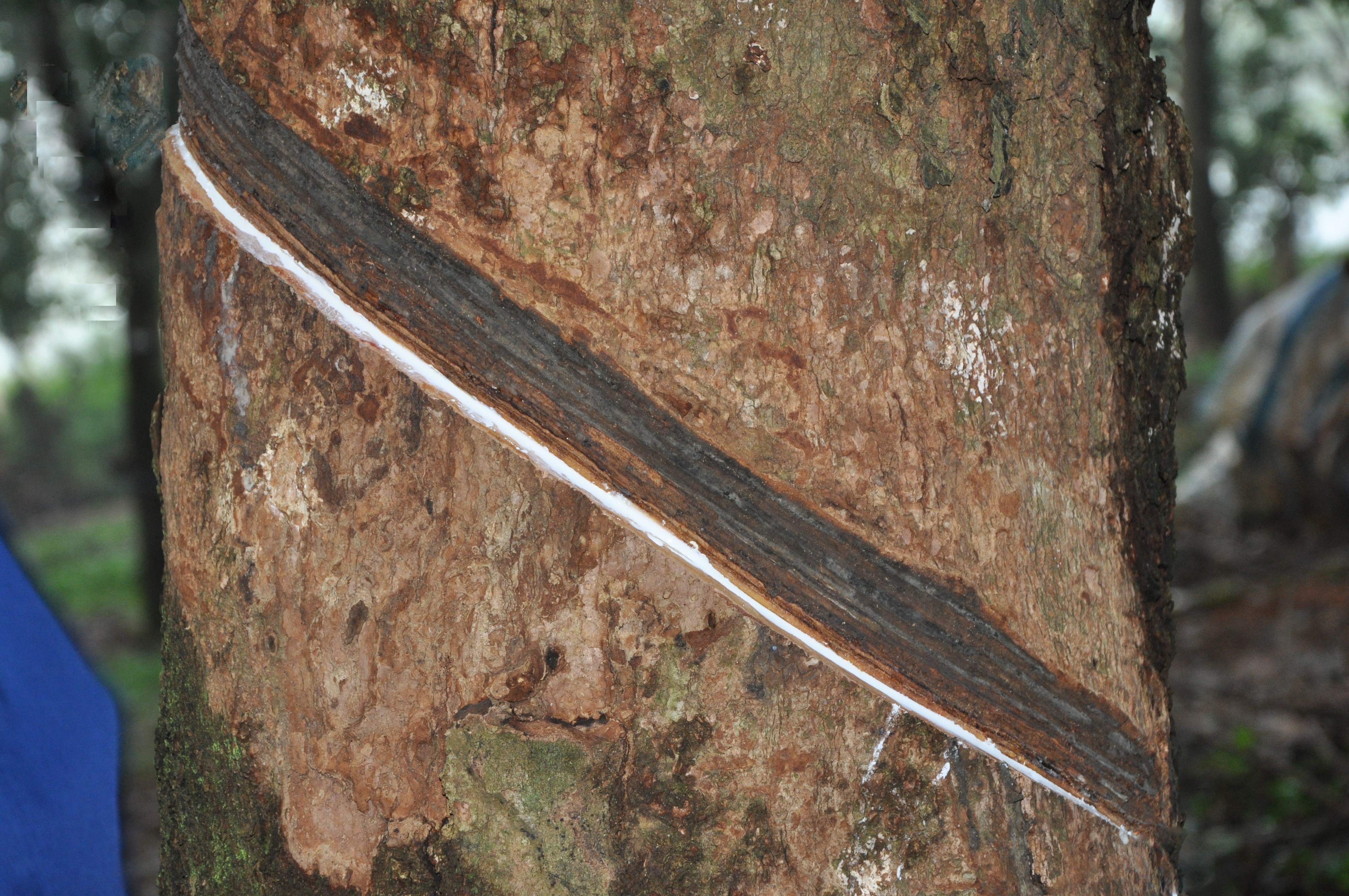

Supplement: S1 Data — (ZIP) [file pone.0297284.s001.zip › Level 1 Original Sample/1-61602-366-20140928-0083.JPG]

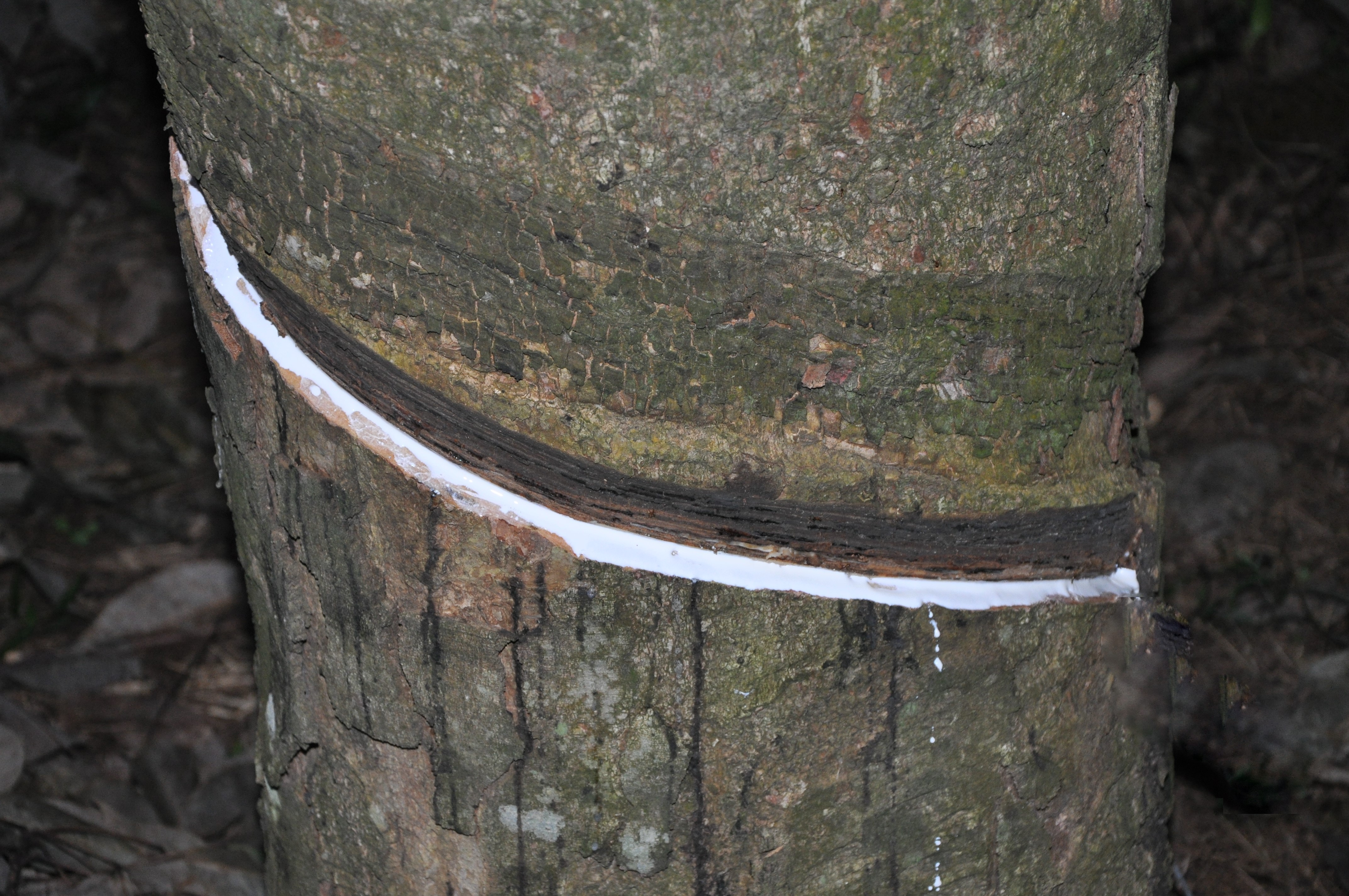

Supplement: S1 Data — (ZIP) [file pone.0297284.s001.zip › Level 1 Original Sample/1-62001-149-20140929-0024.JPG]

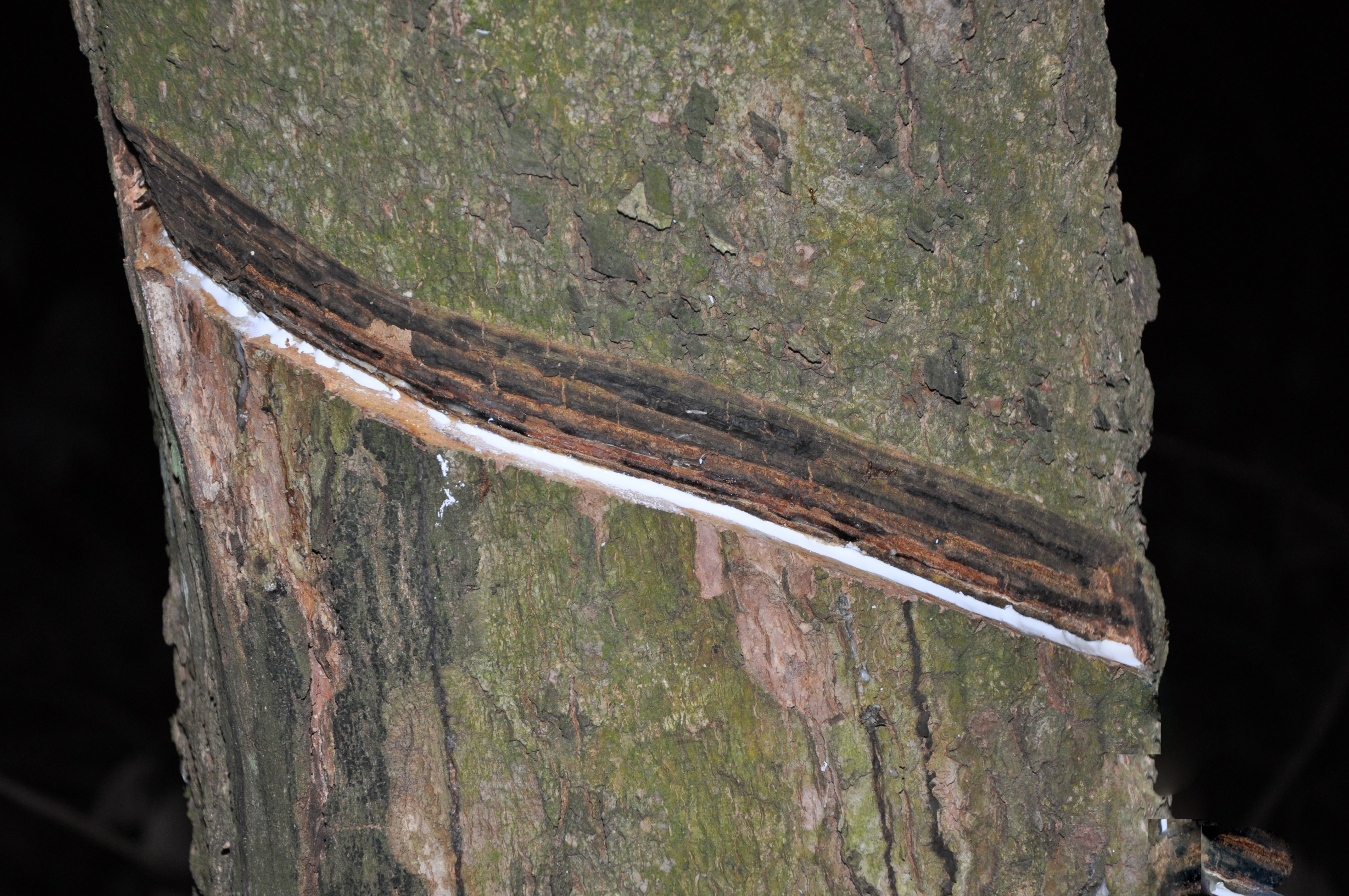

Supplement: S1 Data — (ZIP) [file pone.0297284.s001.zip › Level 1 Original Sample/1-62001-149-20141111-0022.JPG]

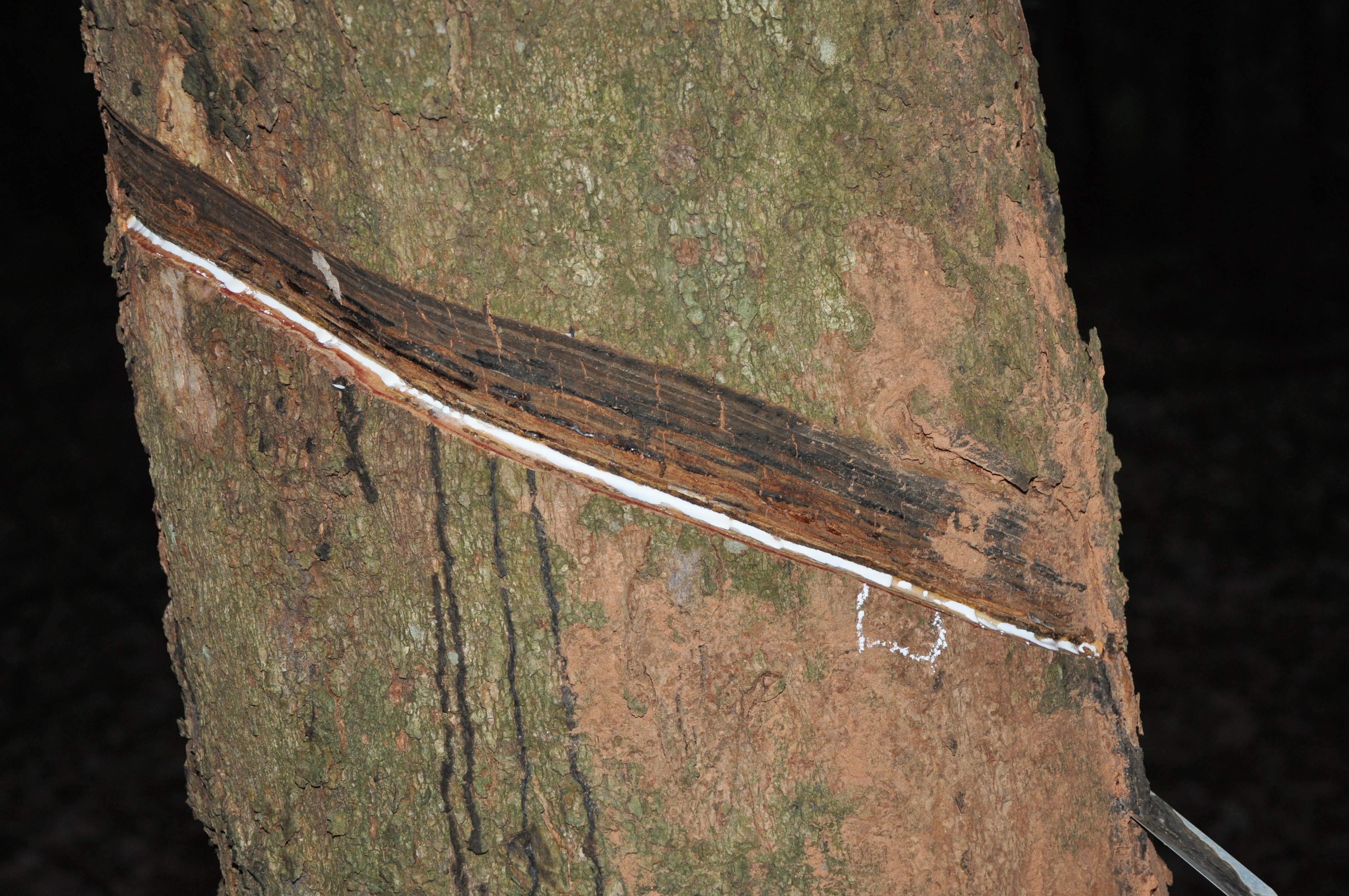

Supplement: S1 Data — (ZIP) [file pone.0297284.s001.zip › Level 1 Original Sample/1-62001-320-20141111-0076.JPG]

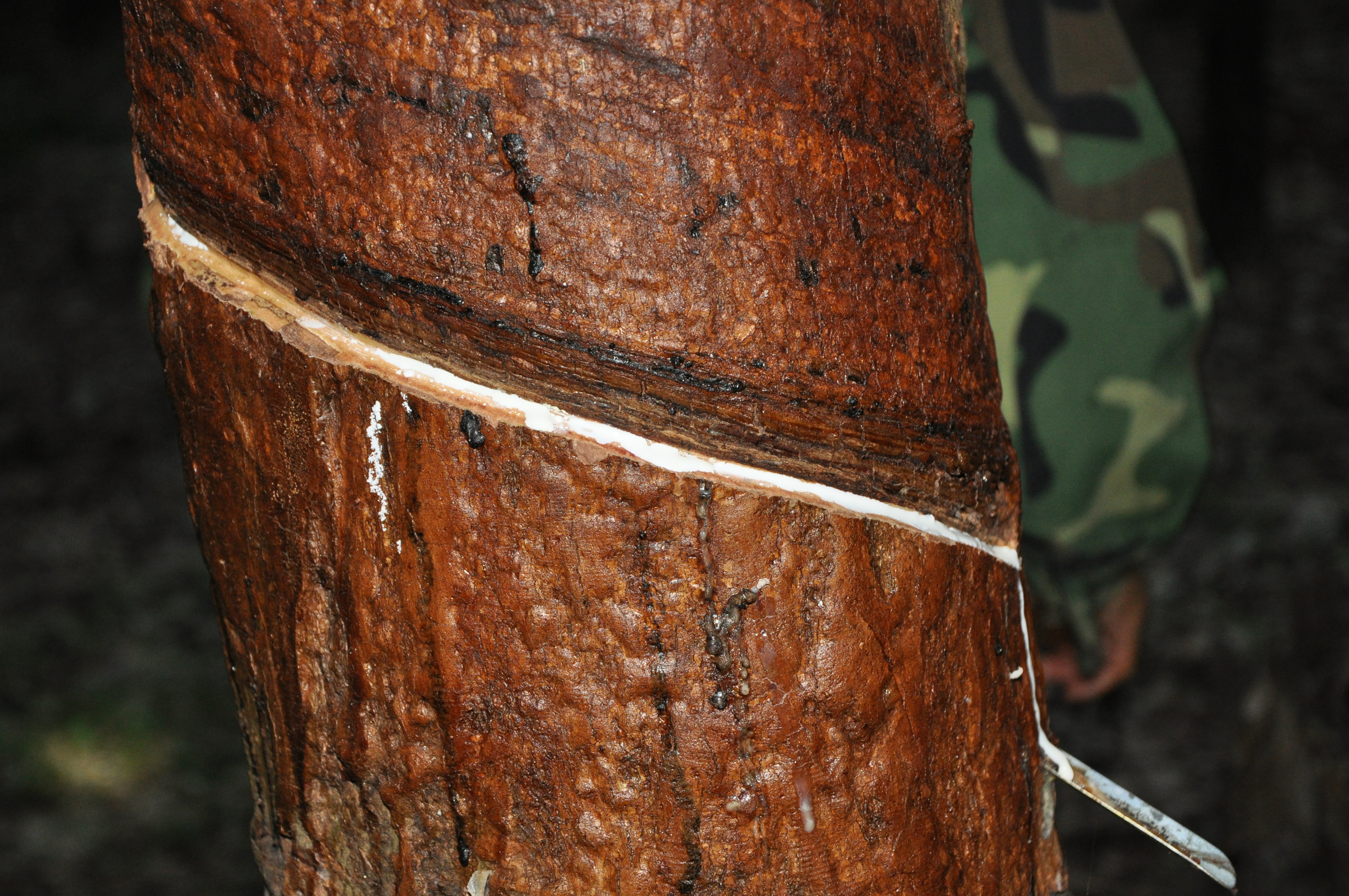

Supplement: S1 Data — (ZIP) [file pone.0297284.s001.zip › Level 1 Original Sample/1-62001-357-20141111-0109.JPG]

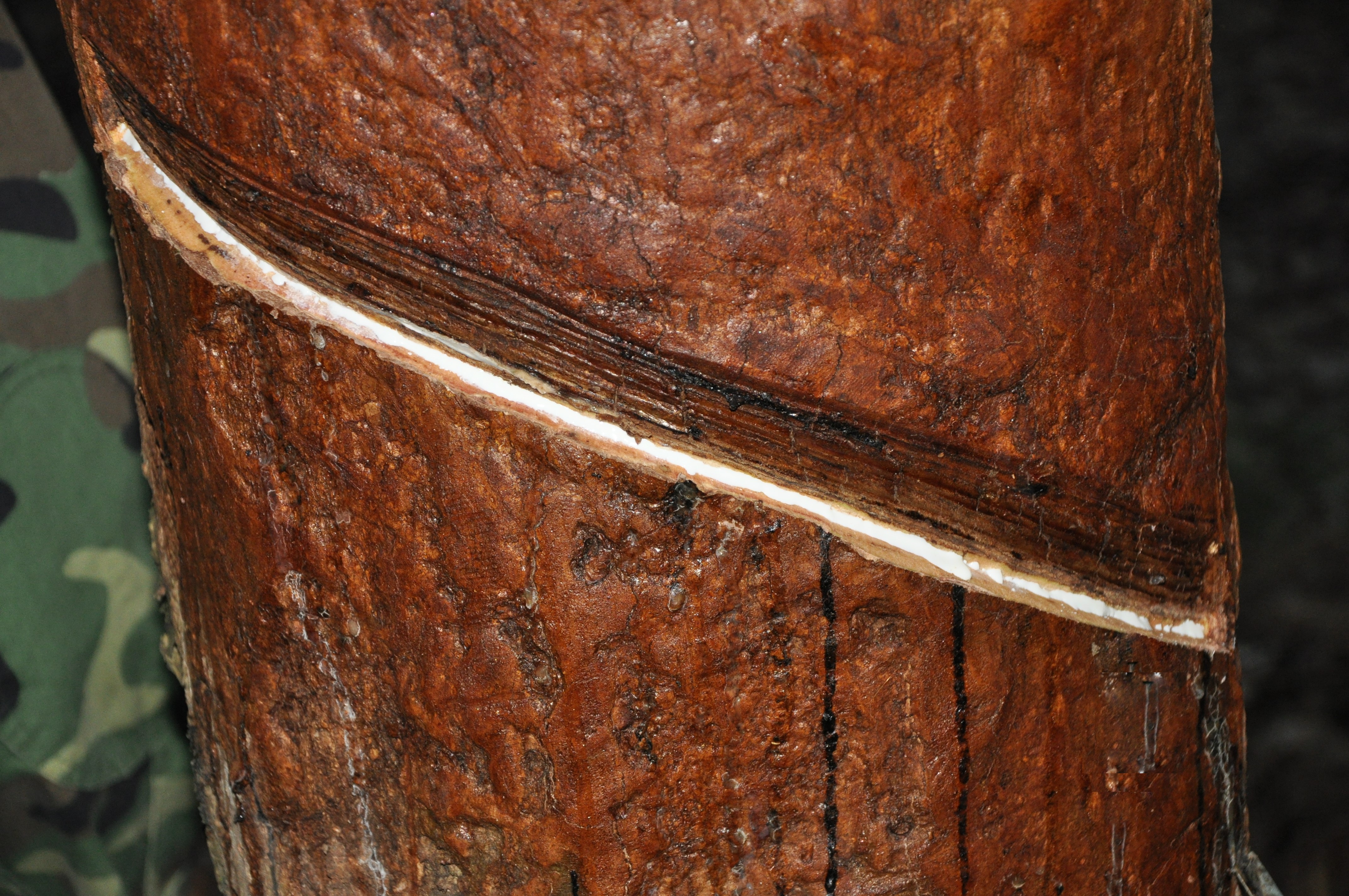

Supplement: S1 Data — (ZIP) [file pone.0297284.s001.zip › Level 1 Original Sample/1-62001-358-20141111-0107.JPG]

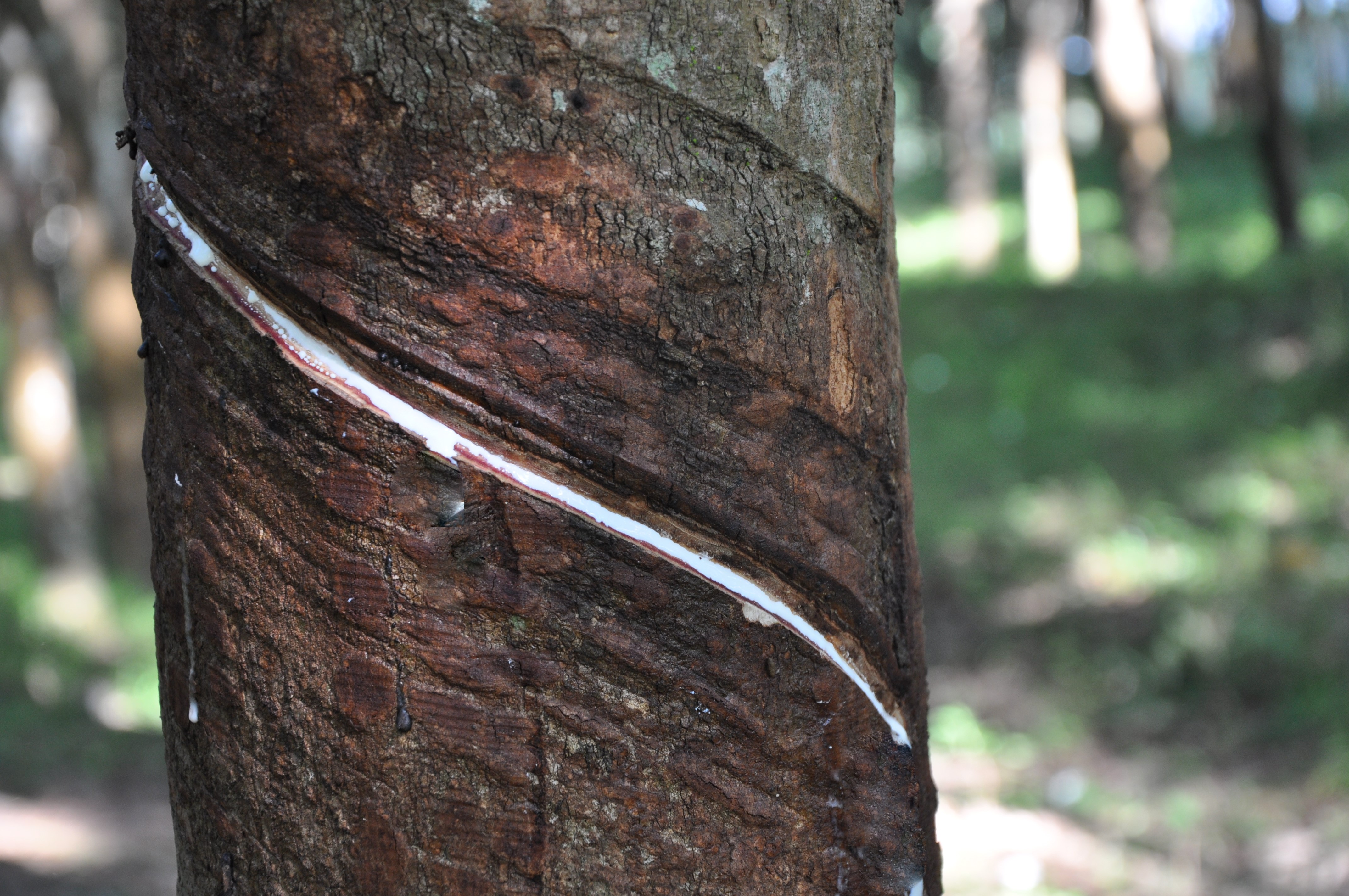

Supplement: S1 Data — (ZIP) [file pone.0297284.s001.zip › Level 1 Original Sample/1-xxxxx-21-20140904-0049.JPG]

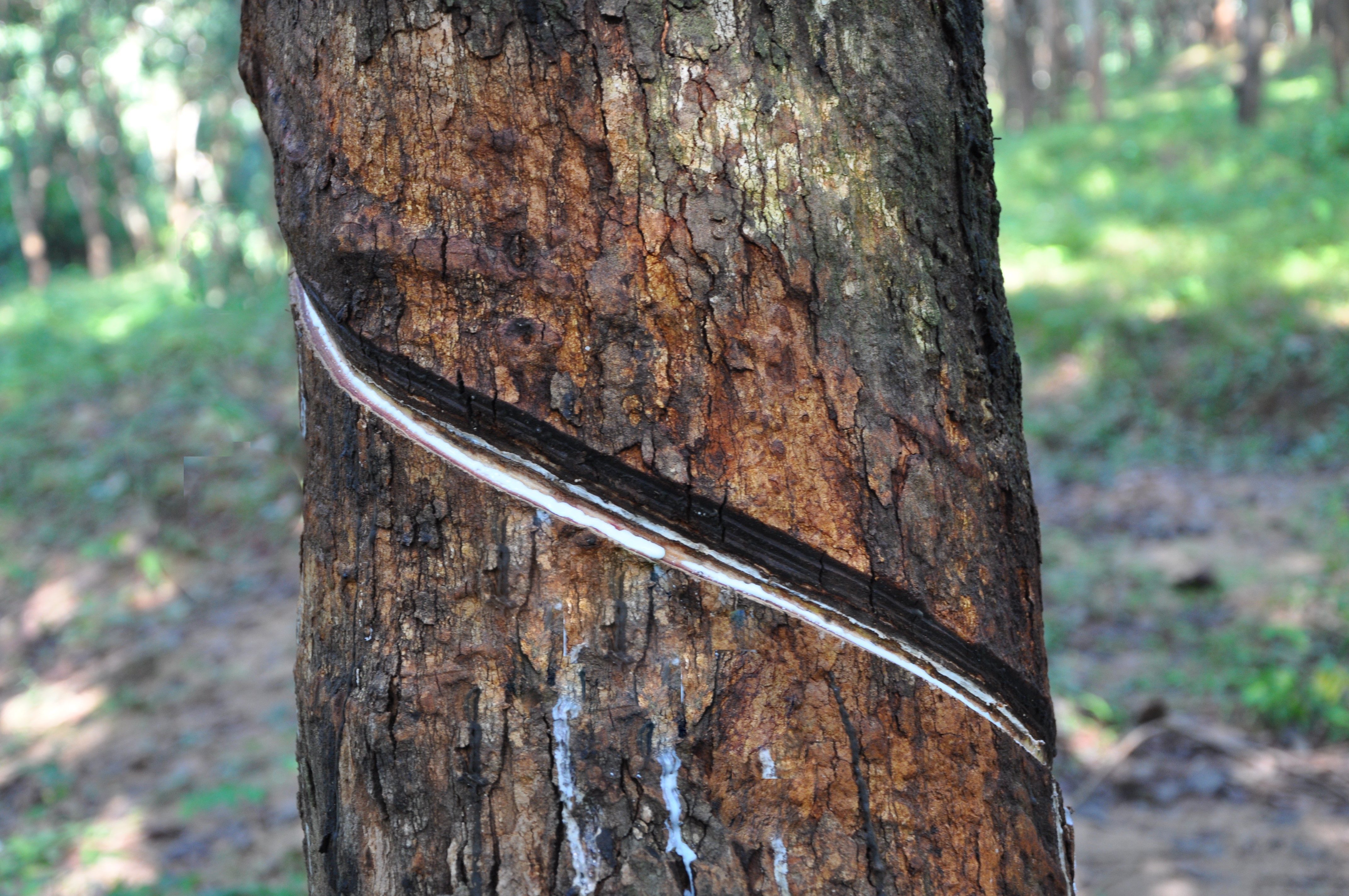

Supplement: S1 Data — (ZIP) [file pone.0297284.s001.zip › Level 1 Original Sample/1-xxxxx-99-20141021-1036.JPG]

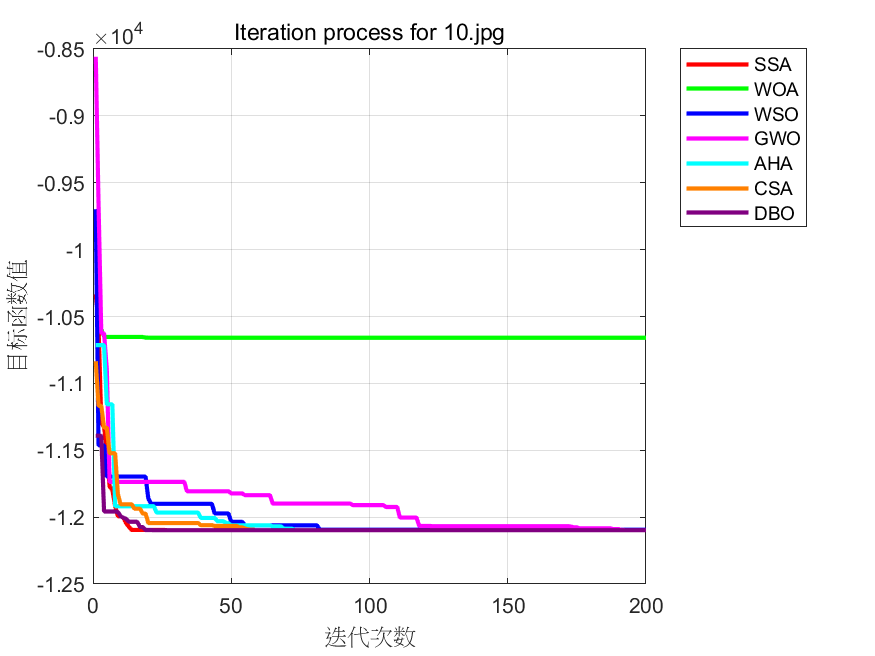

Supplement: S2 Data — (ZIP) [file pone.0297284.s002.zip › Level 1 processed Sample/iteration/10.jpg_iteration.png]

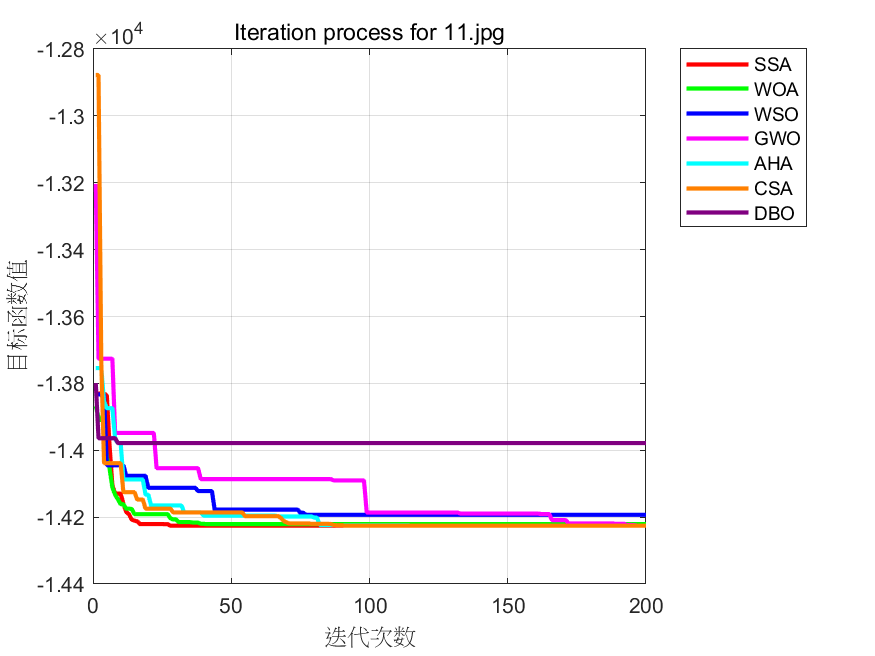

Supplement: S2 Data — (ZIP) [file pone.0297284.s002.zip › Level 1 processed Sample/iteration/11.jpg_iteration.png]

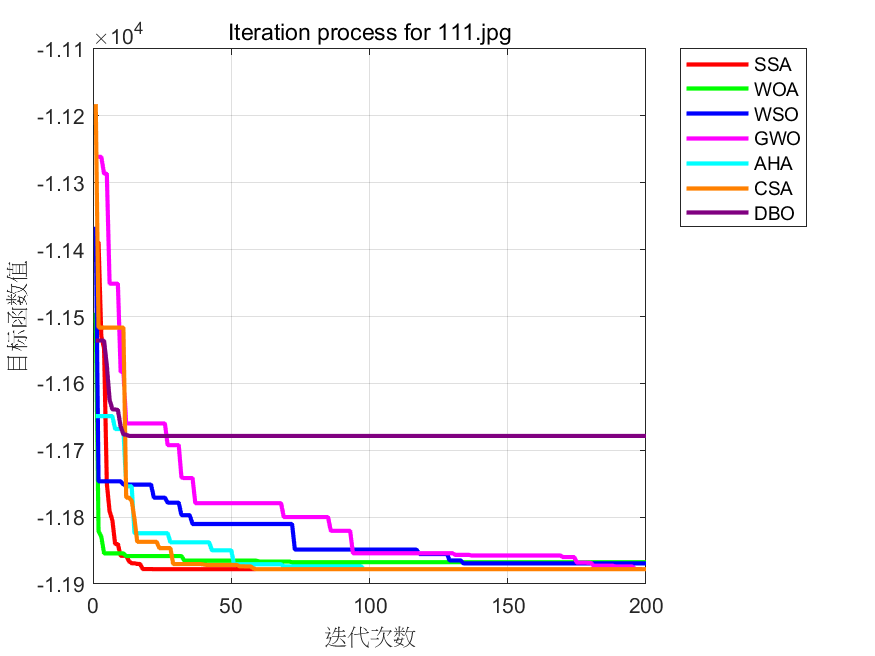

Supplement: S2 Data — (ZIP) [file pone.0297284.s002.zip › Level 1 processed Sample/iteration/111.jpg_iteration.png]

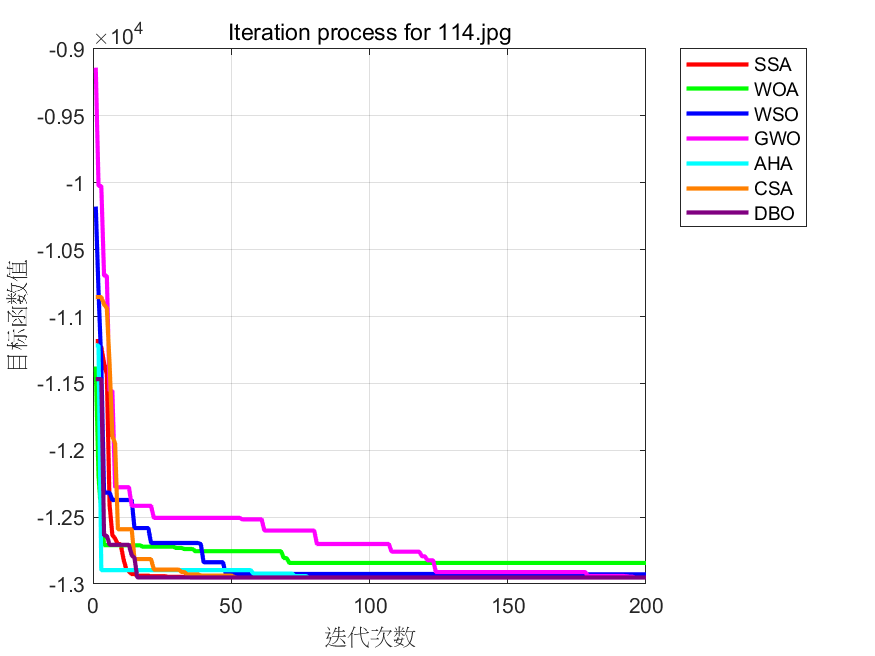

Supplement: S2 Data — (ZIP) [file pone.0297284.s002.zip › Level 1 processed Sample/iteration/114.jpg_iteration.png]

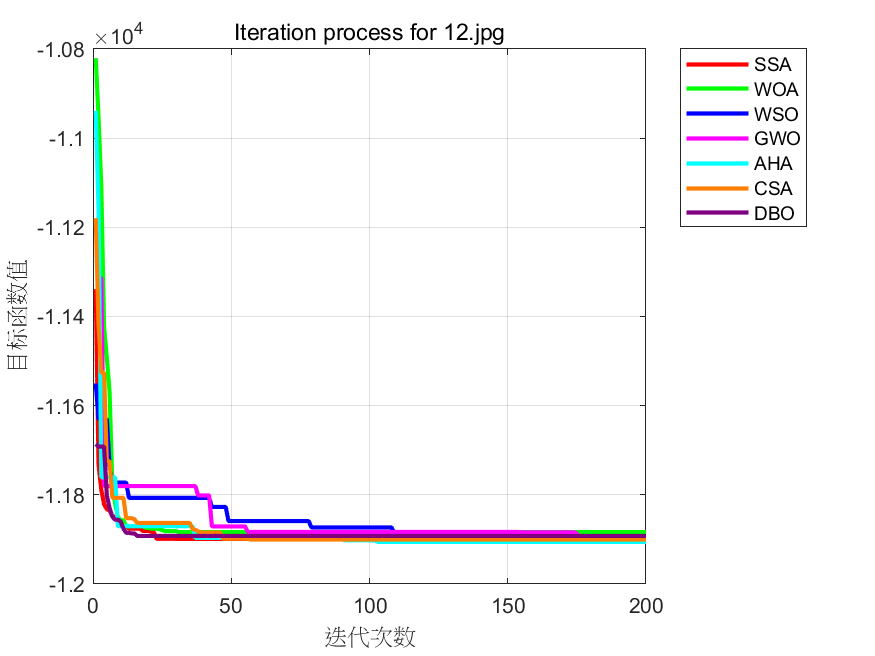

Supplement: S2 Data — (ZIP) [file pone.0297284.s002.zip › Level 1 processed Sample/iteration/12.jpg_iteration.png]

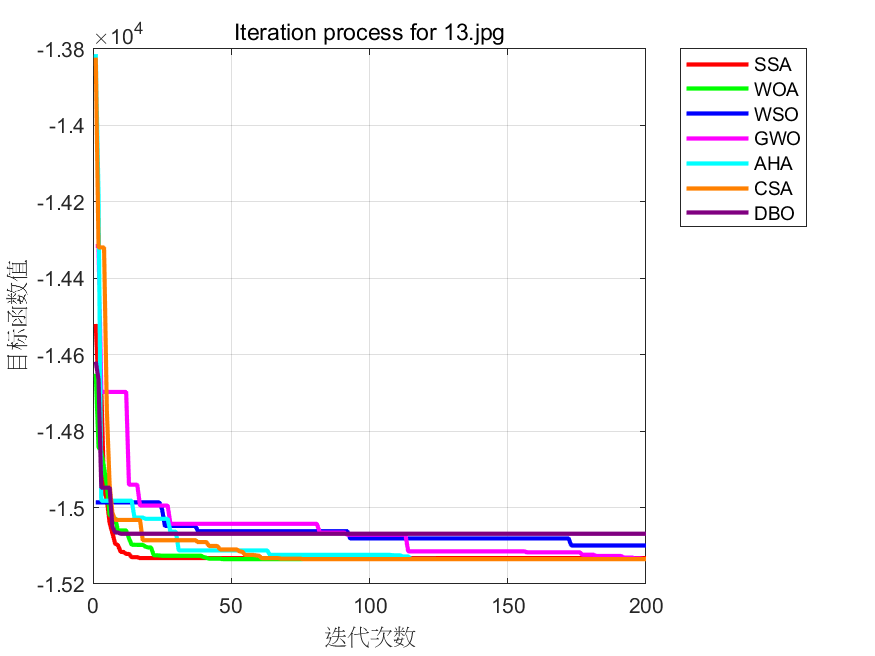

Supplement: S2 Data — (ZIP) [file pone.0297284.s002.zip › Level 1 processed Sample/iteration/13.jpg_iteration.png]

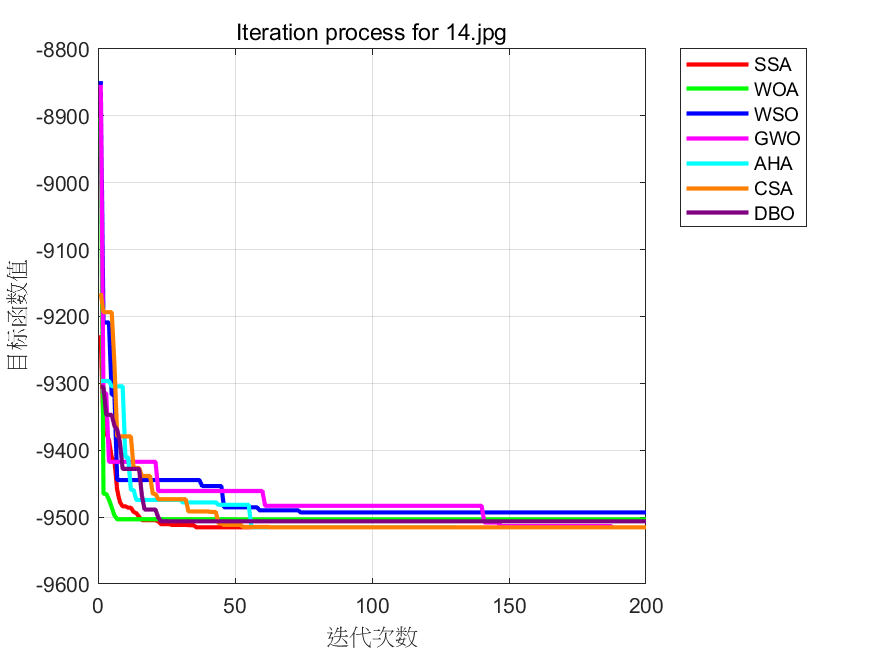

Supplement: S2 Data — (ZIP) [file pone.0297284.s002.zip › Level 1 processed Sample/iteration/14.jpg_iteration.png]

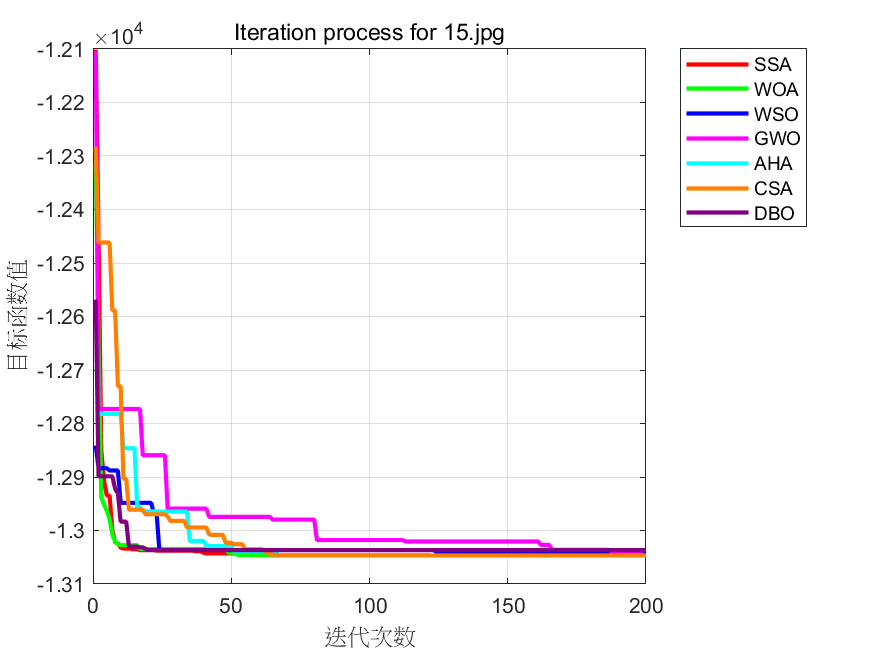

Supplement: S2 Data — (ZIP) [file pone.0297284.s002.zip › Level 1 processed Sample/iteration/15.jpg_iteration.png]

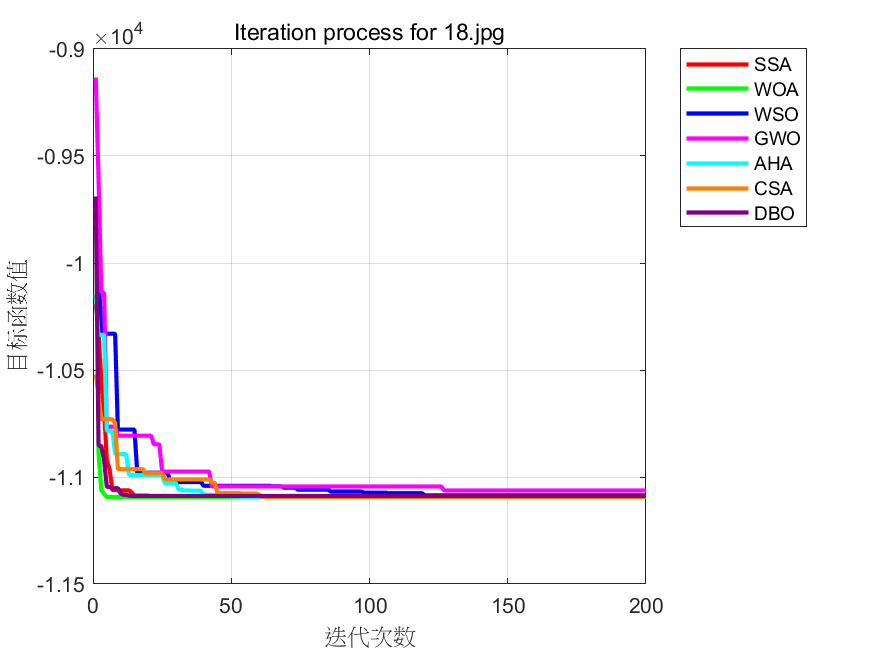

Supplement: S2 Data — (ZIP) [file pone.0297284.s002.zip › Level 1 processed Sample/iteration/18.jpg_iteration.png]

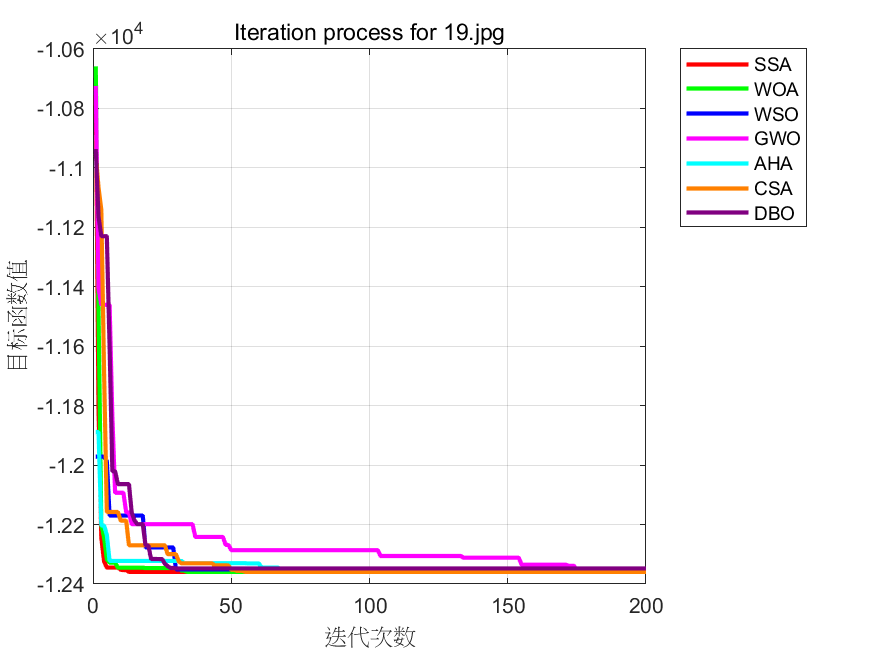

Supplement: S2 Data — (ZIP) [file pone.0297284.s002.zip › Level 1 processed Sample/iteration/19.jpg_iteration.png]

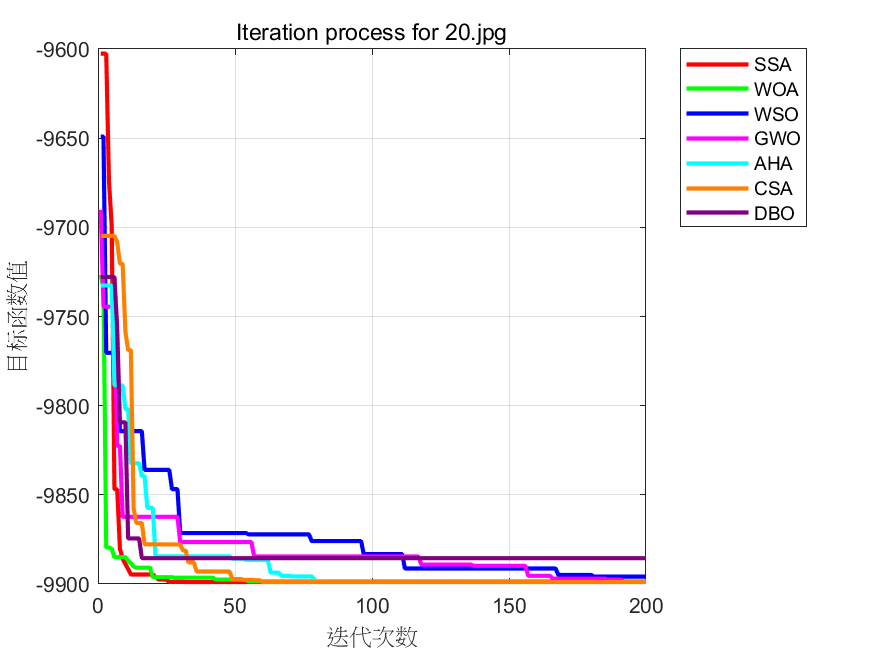

Supplement: S2 Data — (ZIP) [file pone.0297284.s002.zip › Level 1 processed Sample/iteration/20.jpg_iteration.png]

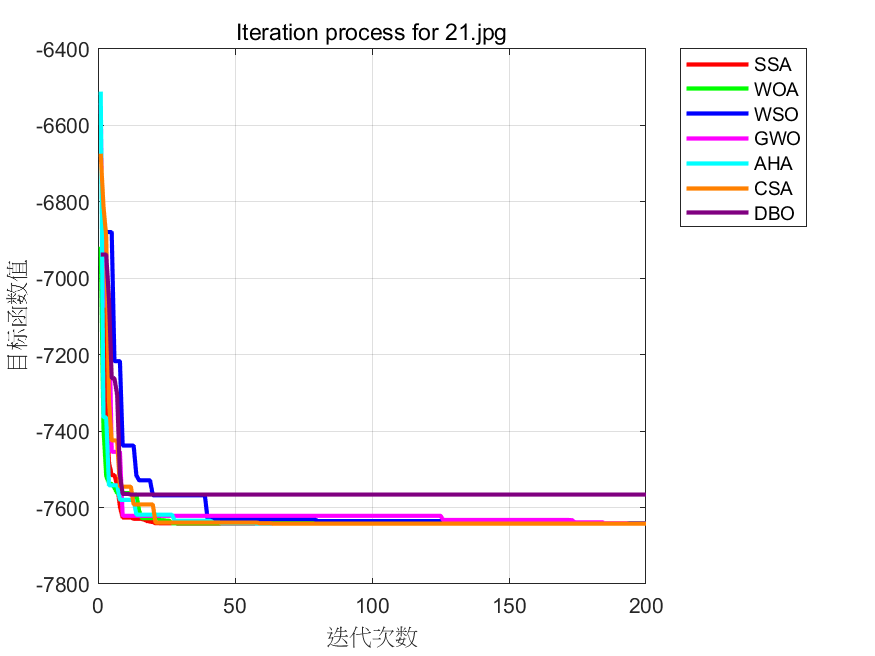

Supplement: S2 Data — (ZIP) [file pone.0297284.s002.zip › Level 1 processed Sample/iteration/21.jpg_iteration.png]

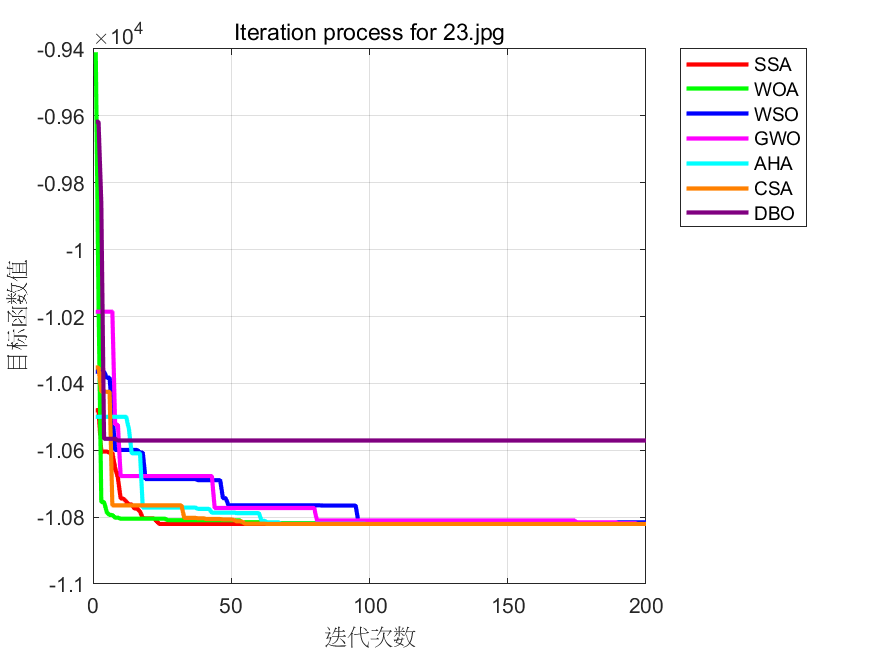

Supplement: S2 Data — (ZIP) [file pone.0297284.s002.zip › Level 1 processed Sample/iteration/23.jpg_iteration.png]

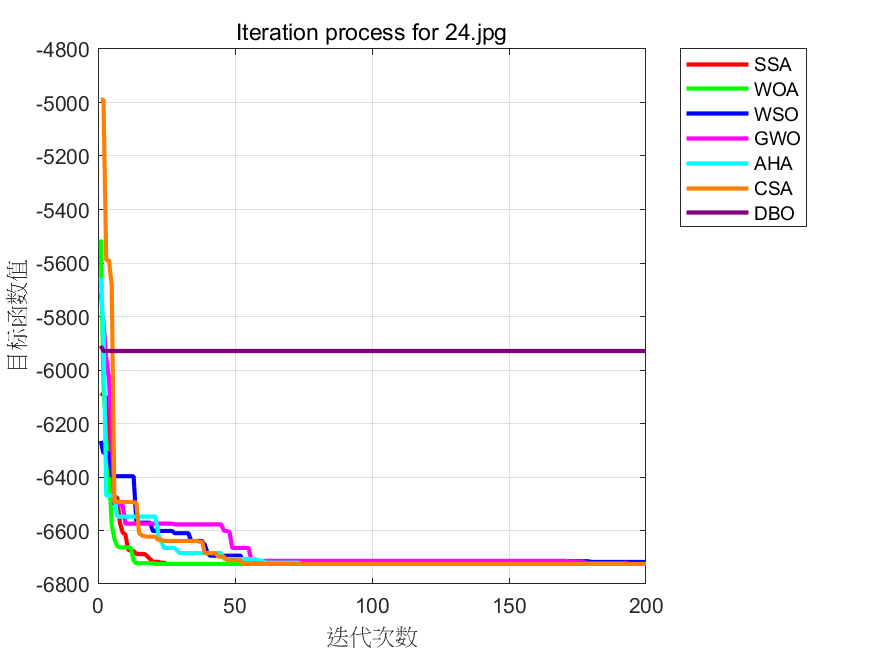

Supplement: S2 Data — (ZIP) [file pone.0297284.s002.zip › Level 1 processed Sample/iteration/24.jpg_iteration.png]

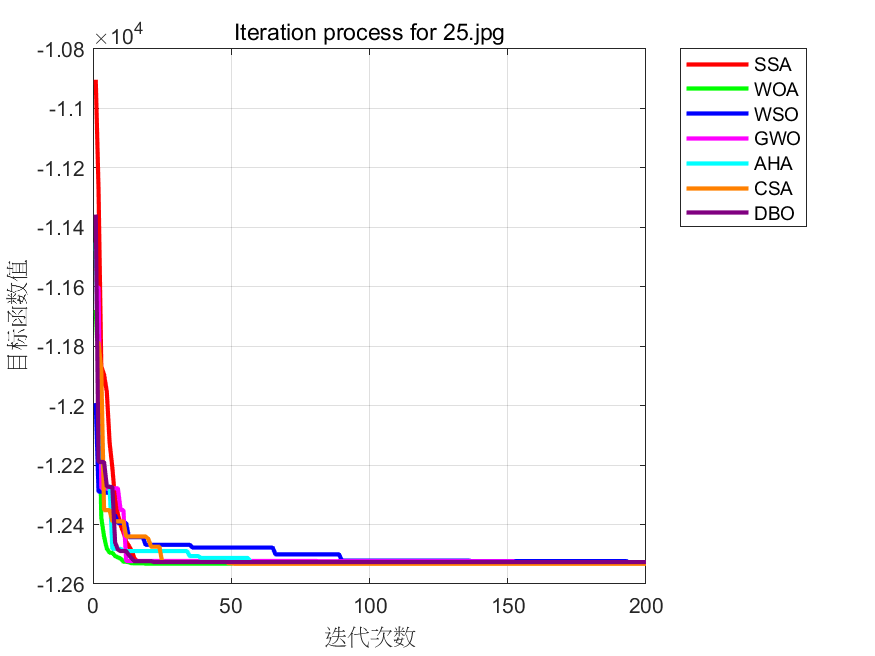

Supplement: S2 Data — (ZIP) [file pone.0297284.s002.zip › Level 1 processed Sample/iteration/25.jpg_iteration.png]

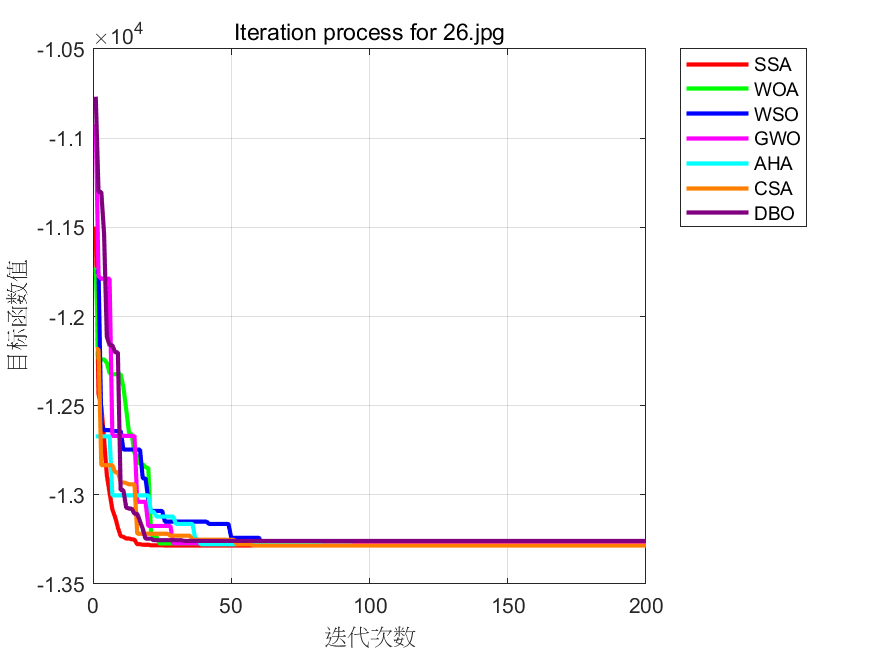

Supplement: S2 Data — (ZIP) [file pone.0297284.s002.zip › Level 1 processed Sample/iteration/26.jpg_iteration.png]

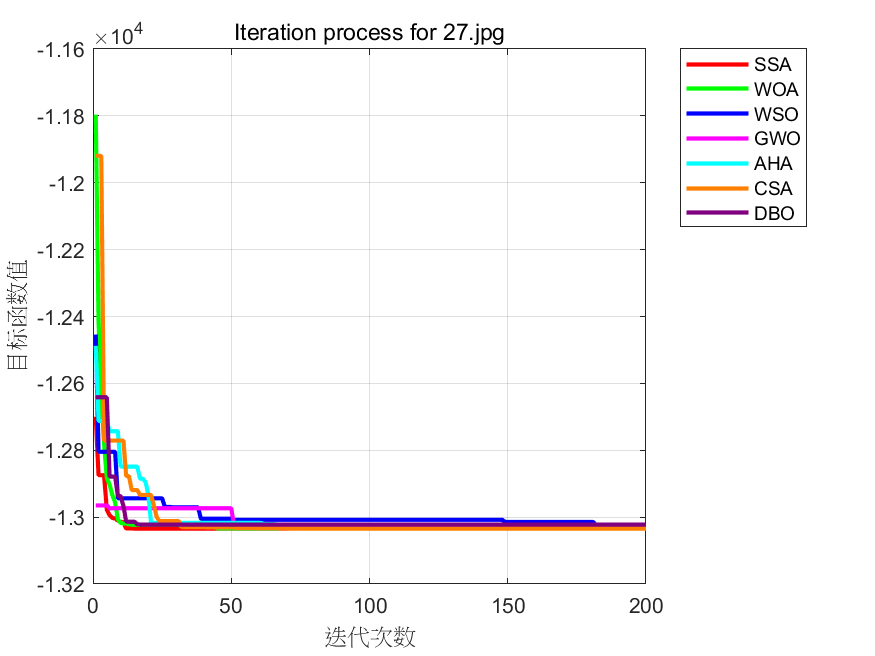

Supplement: S2 Data — (ZIP) [file pone.0297284.s002.zip › Level 1 processed Sample/iteration/27.jpg_iteration.png]

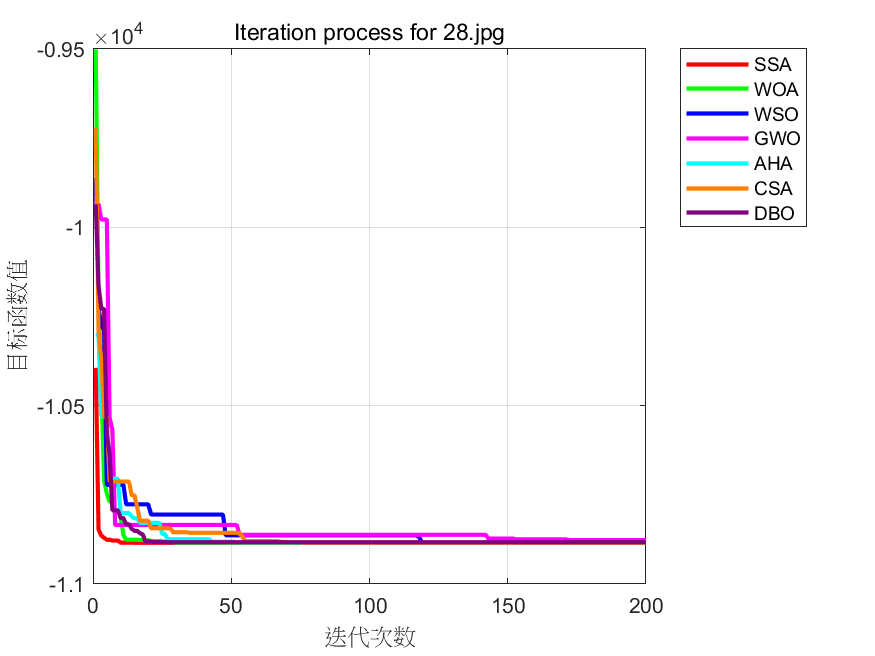

Supplement: S2 Data — (ZIP) [file pone.0297284.s002.zip › Level 1 processed Sample/iteration/28.jpg_iteration.png]

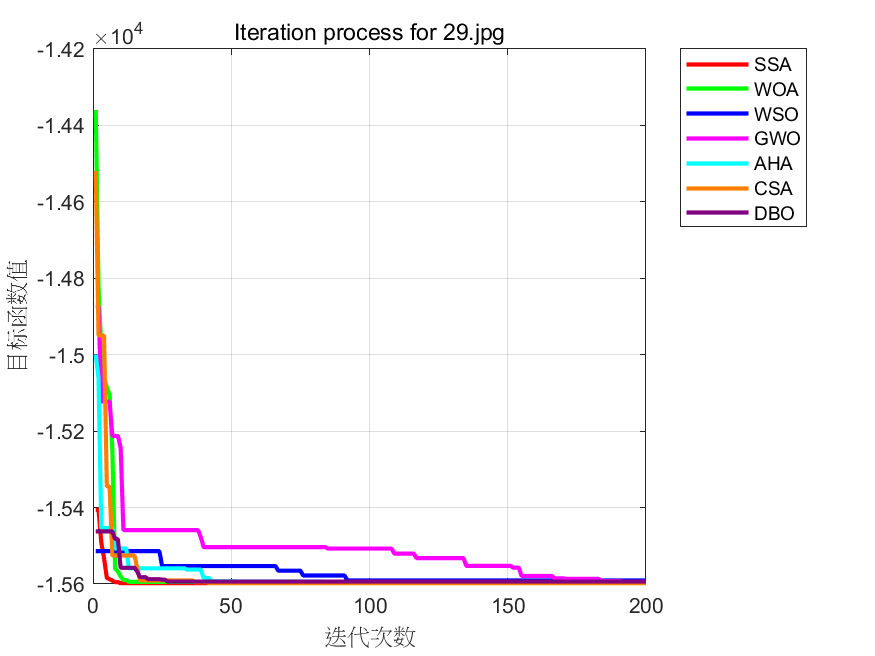

Supplement: S2 Data — (ZIP) [file pone.0297284.s002.zip › Level 1 processed Sample/iteration/29.jpg_iteration.png]

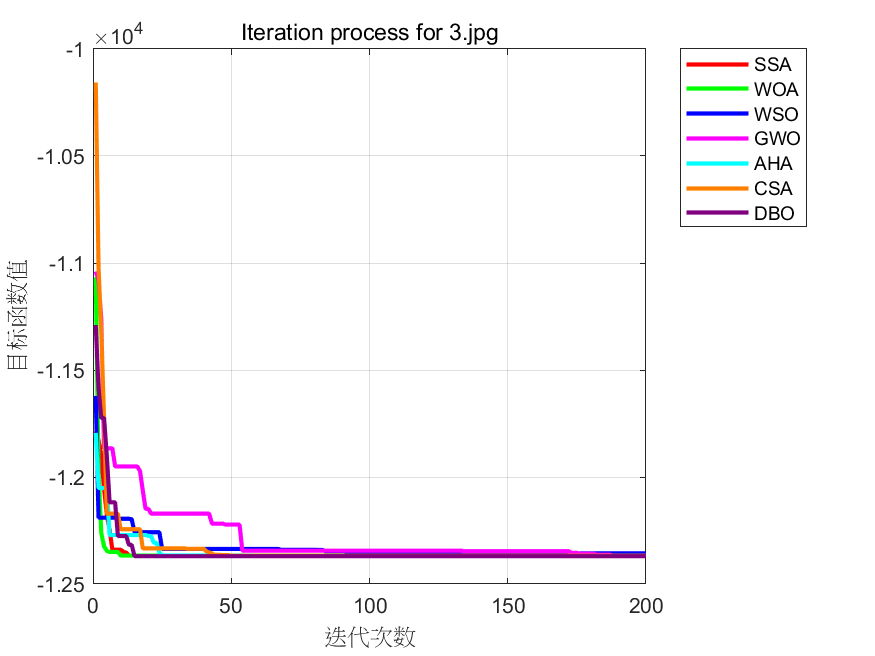

Supplement: S2 Data — (ZIP) [file pone.0297284.s002.zip › Level 1 processed Sample/iteration/3.jpg_iteration.png]

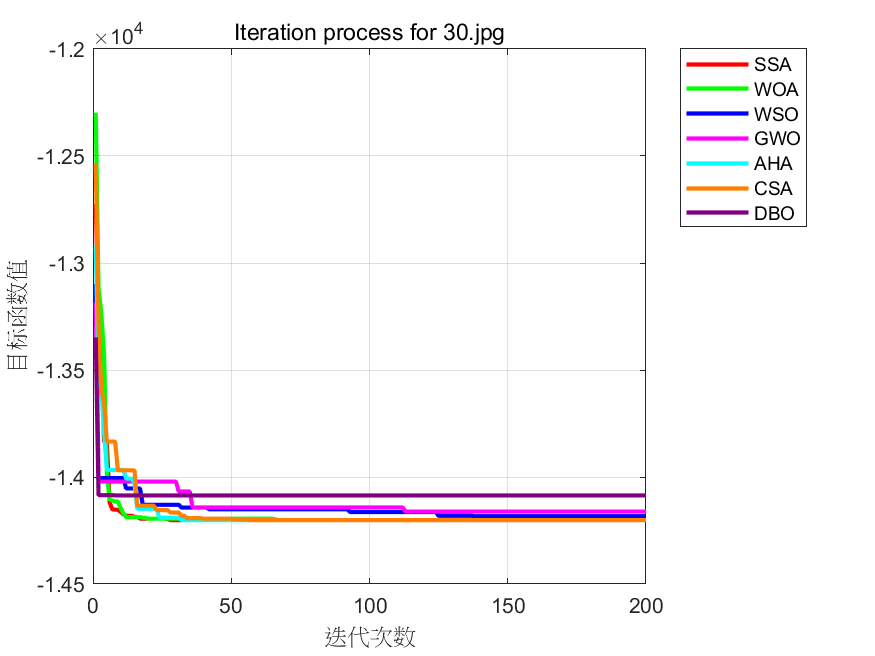

Supplement: S2 Data — (ZIP) [file pone.0297284.s002.zip › Level 1 processed Sample/iteration/30.jpg_iteration.png]

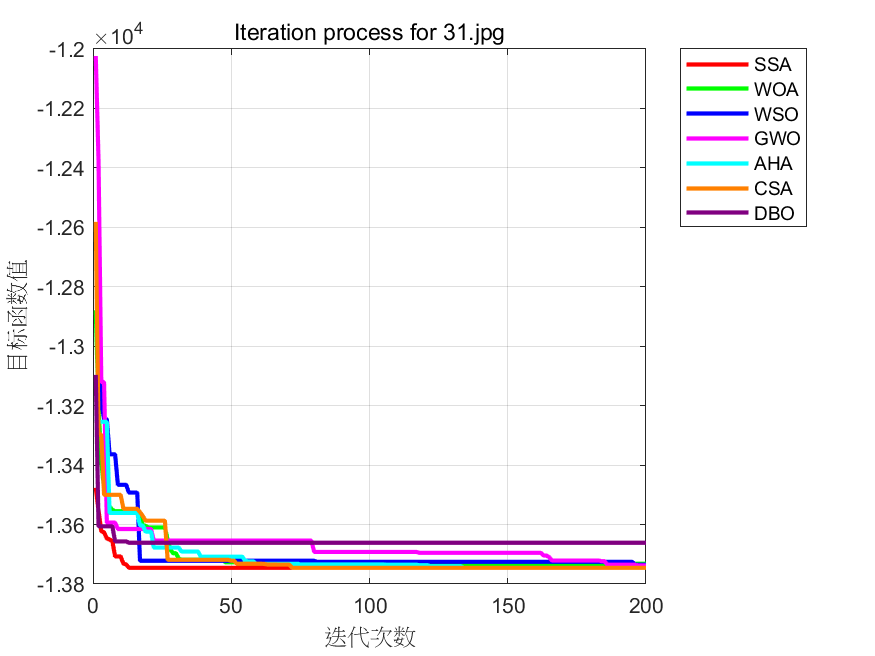

Supplement: S2 Data — (ZIP) [file pone.0297284.s002.zip › Level 1 processed Sample/iteration/31.jpg_iteration.png]

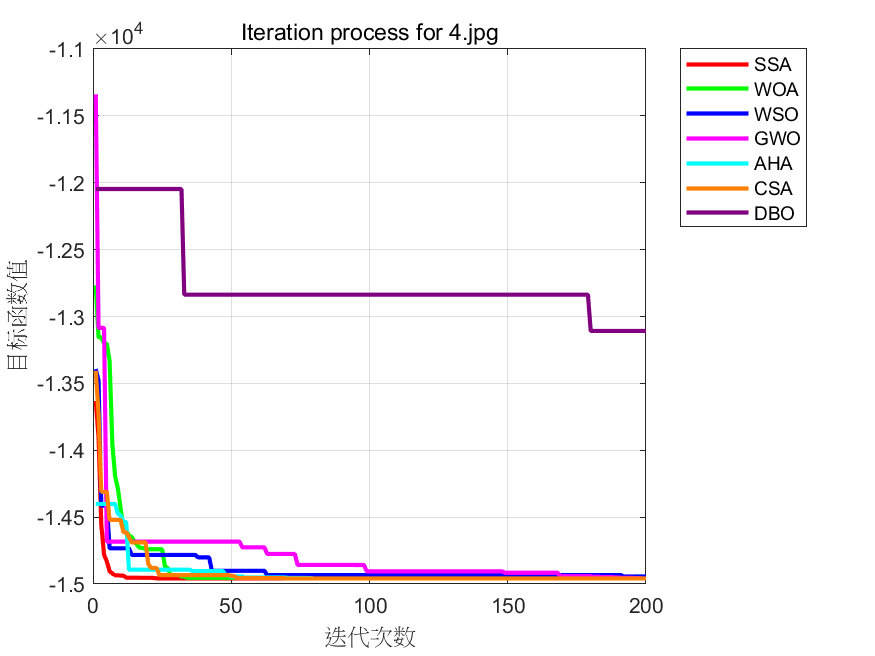

Supplement: S2 Data — (ZIP) [file pone.0297284.s002.zip › Level 1 processed Sample/iteration/4.jpg_iteration.png]

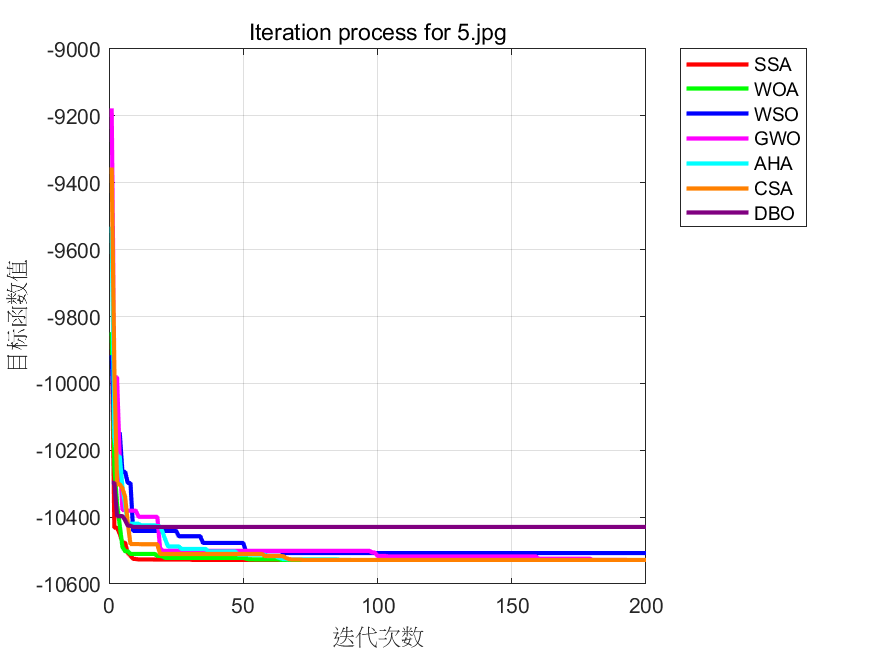

Supplement: S2 Data — (ZIP) [file pone.0297284.s002.zip › Level 1 processed Sample/iteration/5.jpg_iteration.png]

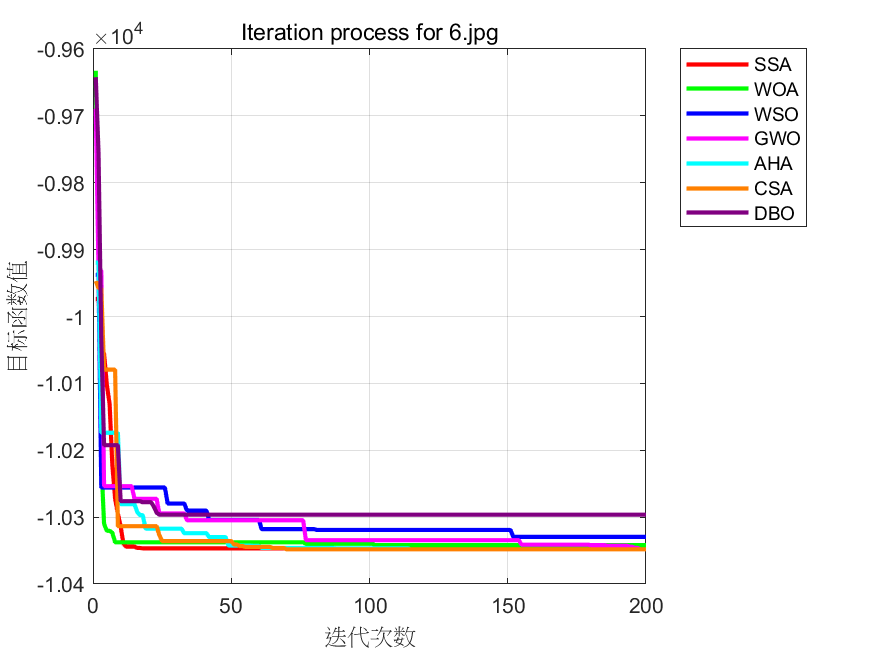

Supplement: S2 Data — (ZIP) [file pone.0297284.s002.zip › Level 1 processed Sample/iteration/6.jpg_iteration.png]

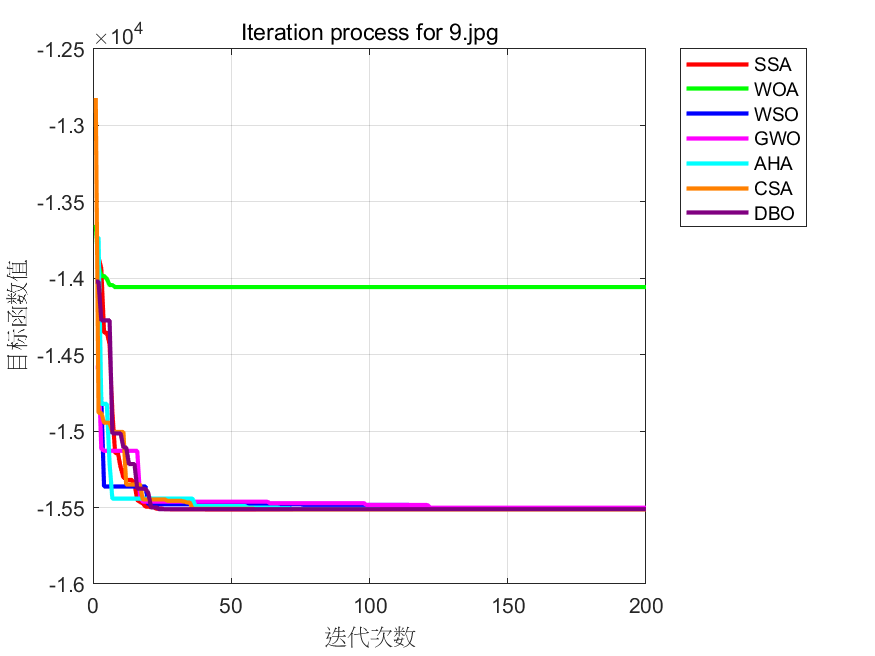

Supplement: S2 Data — (ZIP) [file pone.0297284.s002.zip › Level 1 processed Sample/iteration/9.jpg_iteration.png]

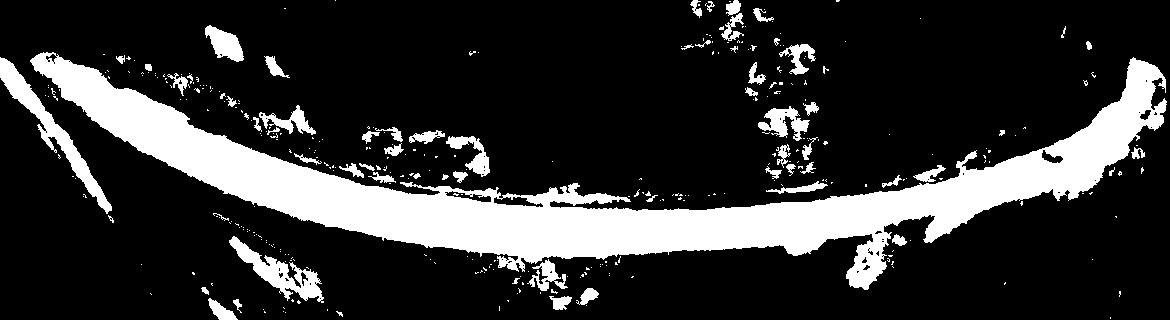

Supplement: S2 Data — (ZIP) [file pone.0297284.s002.zip › Level 1 processed Sample/processed_10/latex/AHA_latex.jpg]

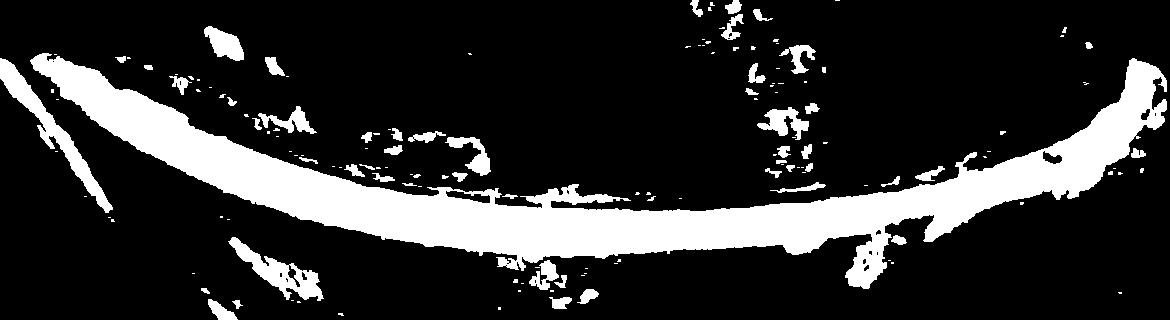

Supplement: S2 Data — (ZIP) [file pone.0297284.s002.zip › Level 1 processed Sample/processed_10/latex/DBO_latex.jpg]

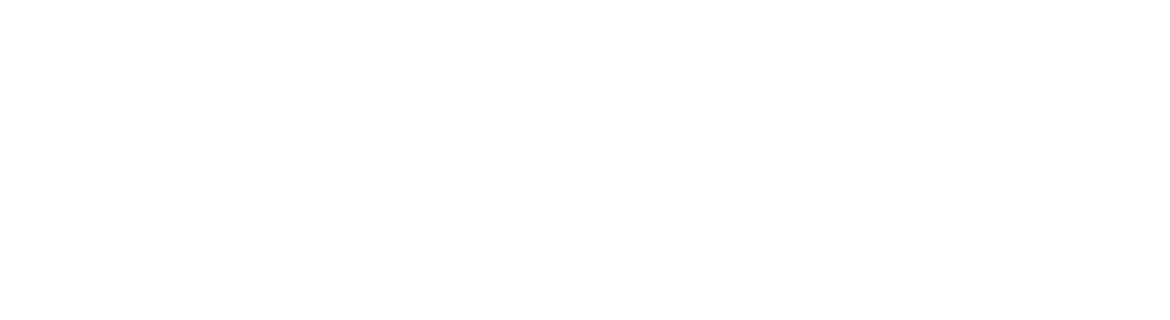

Supplement: S2 Data — (ZIP) [file pone.0297284.s002.zip › Level 1 processed Sample/processed_10/latex/OTSU_latex.jpg]

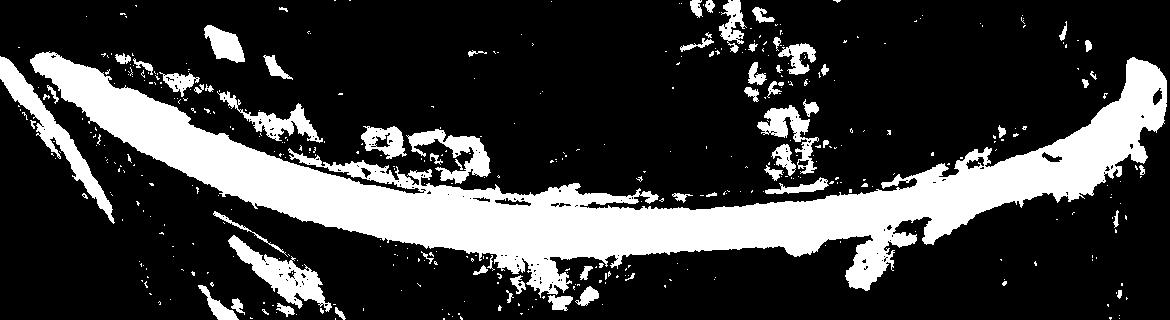

Supplement: S2 Data — (ZIP) [file pone.0297284.s002.zip › Level 1 processed Sample/processed_10/latex/WOA_latex.jpg]

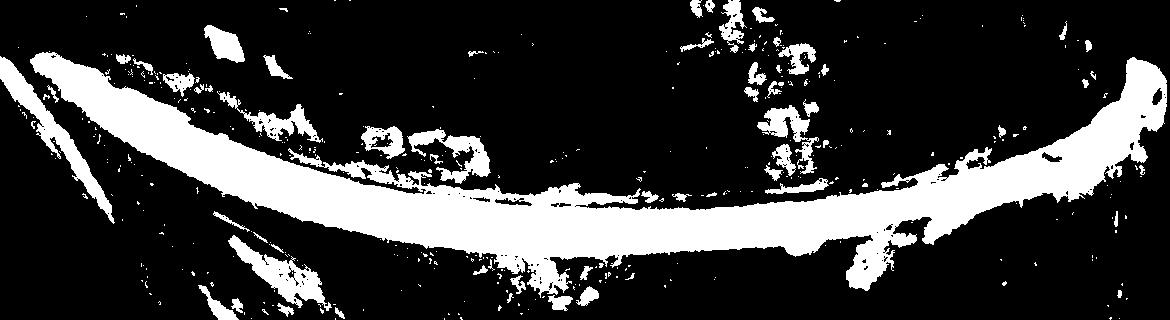

Supplement: S2 Data — (ZIP) [file pone.0297284.s002.zip › Level 1 processed Sample/processed_10/latex/WSO_latex.jpg]

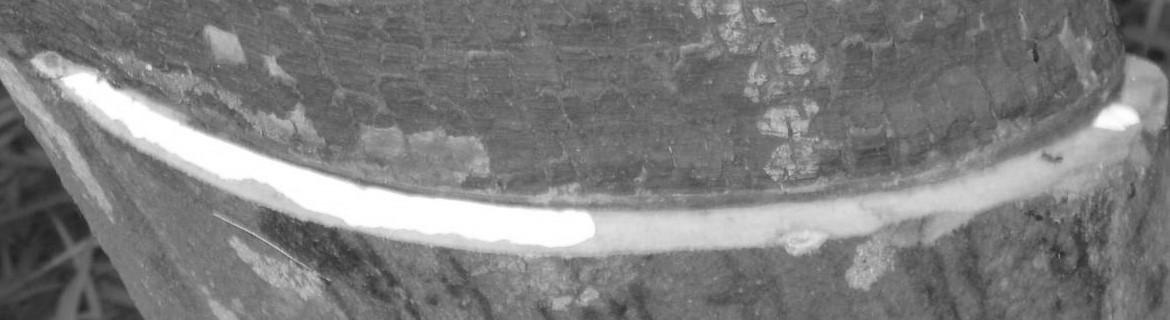

Supplement: S2 Data — (ZIP) [file pone.0297284.s002.zip › Level 1 processed Sample/processed_10/original_image.jpg]

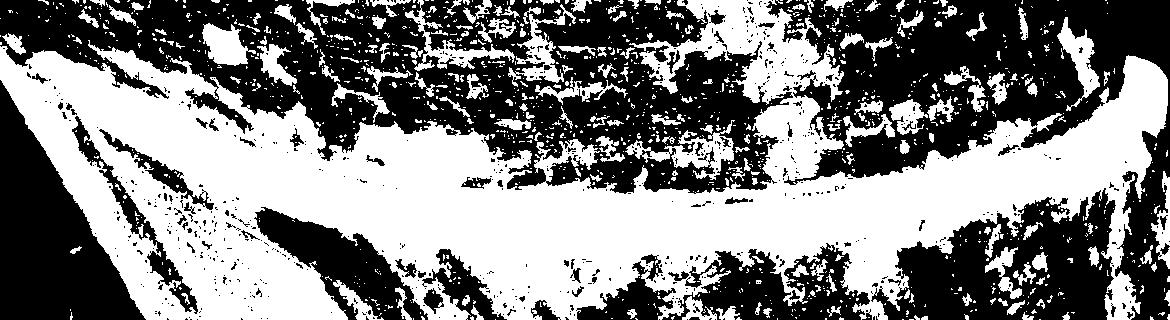

Supplement: S2 Data — (ZIP) [file pone.0297284.s002.zip › Level 1 processed Sample/processed_10/scar/AHA_scar.jpg]

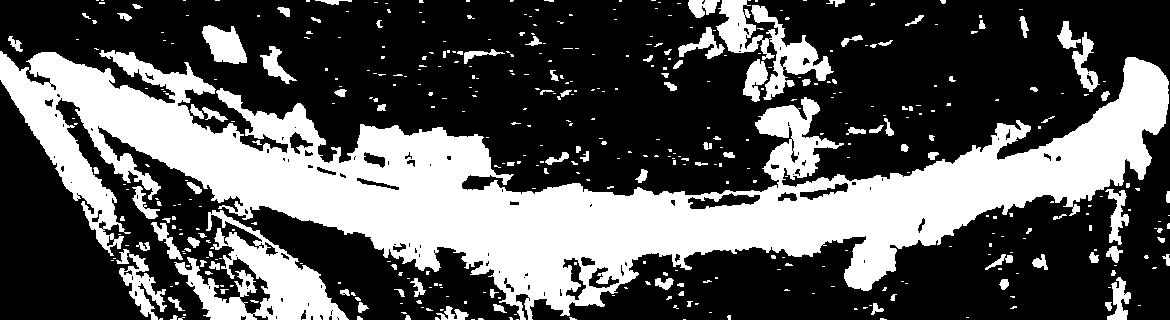

Supplement: S2 Data — (ZIP) [file pone.0297284.s002.zip › Level 1 processed Sample/processed_10/scar/DBO_scar.jpg]

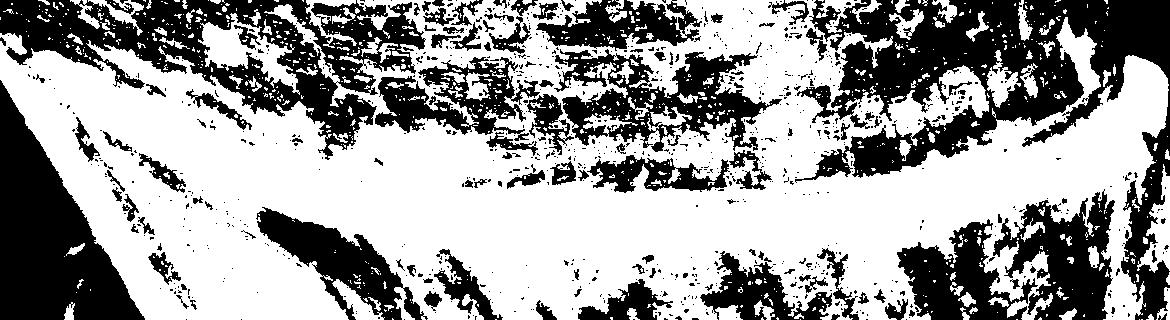

Supplement: S2 Data — (ZIP) [file pone.0297284.s002.zip › Level 1 processed Sample/processed_10/scar/WOA_scar.jpg]

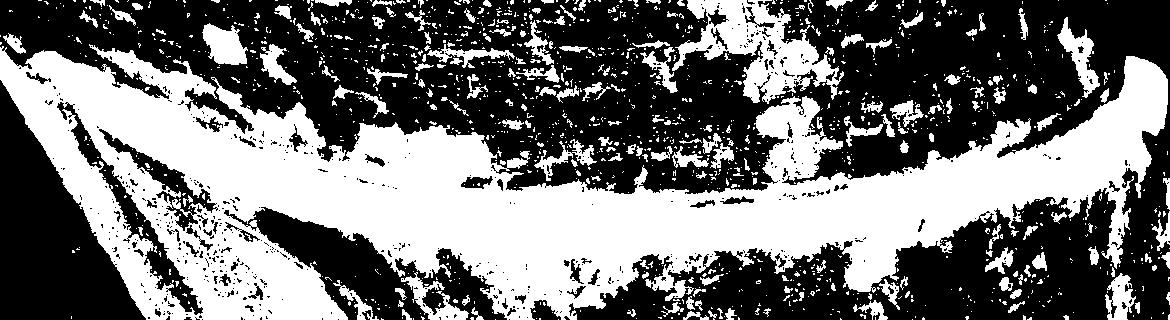

Supplement: S2 Data — (ZIP) [file pone.0297284.s002.zip › Level 1 processed Sample/processed_10/scar/WSO_scar.jpg]

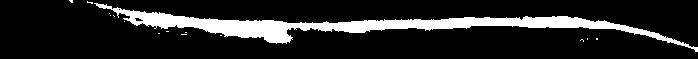

Supplement: S2 Data — (ZIP) [file pone.0297284.s002.zip › Level 1 processed Sample/processed_11/latex/AHA_latex.jpg]

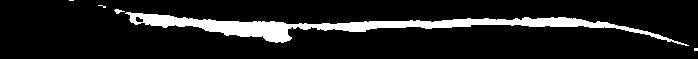

Supplement: S2 Data — (ZIP) [file pone.0297284.s002.zip › Level 1 processed Sample/processed_11/latex/DBO_latex.jpg]

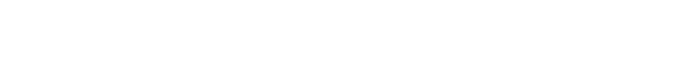

Supplement: S2 Data — (ZIP) [file pone.0297284.s002.zip › Level 1 processed Sample/processed_11/latex/OTSU_latex.jpg]

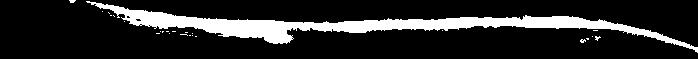

Supplement: S2 Data — (ZIP) [file pone.0297284.s002.zip › Level 1 processed Sample/processed_11/latex/WSO_latex.jpg]

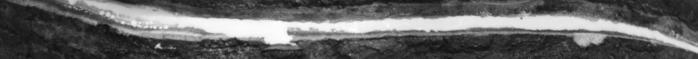

Supplement: S2 Data — (ZIP) [file pone.0297284.s002.zip › Level 1 processed Sample/processed_11/original_image.jpg]

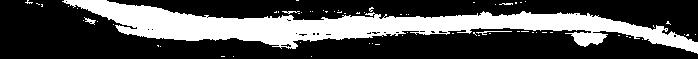

Supplement: S2 Data — (ZIP) [file pone.0297284.s002.zip › Level 1 processed Sample/processed_11/scar/AHA_scar.jpg]

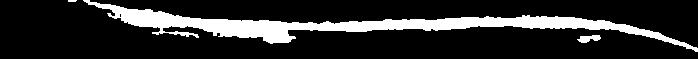

Supplement: S2 Data — (ZIP) [file pone.0297284.s002.zip › Level 1 processed Sample/processed_11/scar/DBO_scar.jpg]

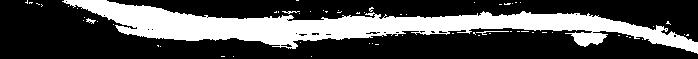

Supplement: S2 Data — (ZIP) [file pone.0297284.s002.zip › Level 1 processed Sample/processed_11/scar/WOA_scar.jpg]

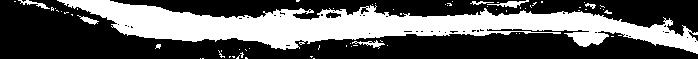

Supplement: S2 Data — (ZIP) [file pone.0297284.s002.zip › Level 1 processed Sample/processed_11/scar/WSO_scar.jpg]

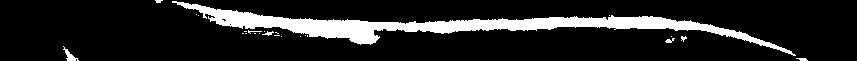

Supplement: S2 Data — (ZIP) [file pone.0297284.s002.zip › Level 1 processed Sample/processed_111/latex/AHA_latex.jpg]

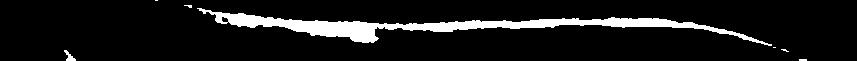

Supplement: S2 Data — (ZIP) [file pone.0297284.s002.zip › Level 1 processed Sample/processed_111/latex/DBO_latex.jpg]

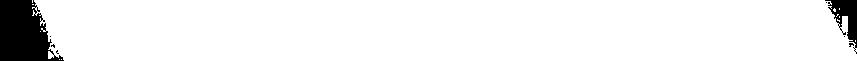

Supplement: S2 Data — (ZIP) [file pone.0297284.s002.zip › Level 1 processed Sample/processed_111/latex/OTSU_latex.jpg]

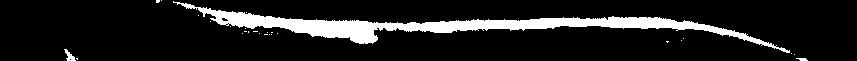

Supplement: S2 Data — (ZIP) [file pone.0297284.s002.zip › Level 1 processed Sample/processed_111/latex/WSO_latex.jpg]

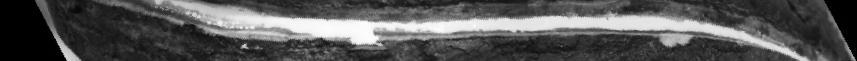

Supplement: S2 Data — (ZIP) [file pone.0297284.s002.zip › Level 1 processed Sample/processed_111/original_image.jpg]

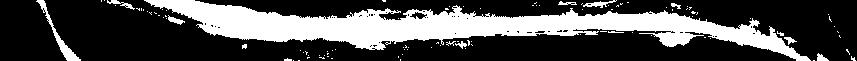

Supplement: S2 Data — (ZIP) [file pone.0297284.s002.zip › Level 1 processed Sample/processed_111/scar/AHA_scar.jpg]

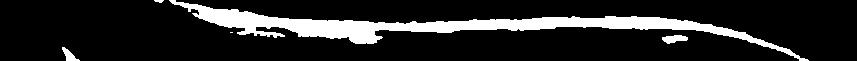

Supplement: S2 Data — (ZIP) [file pone.0297284.s002.zip › Level 1 processed Sample/processed_111/scar/DBO_scar.jpg]

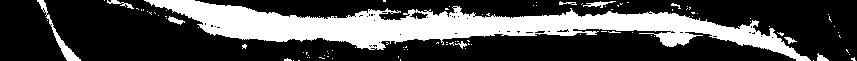

Supplement: S2 Data — (ZIP) [file pone.0297284.s002.zip › Level 1 processed Sample/processed_111/scar/WOA_scar.jpg]

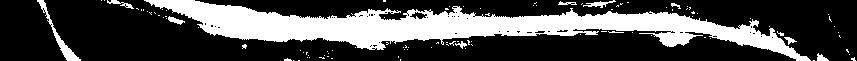

Supplement: S2 Data — (ZIP) [file pone.0297284.s002.zip › Level 1 processed Sample/processed_111/scar/WSO_scar.jpg]

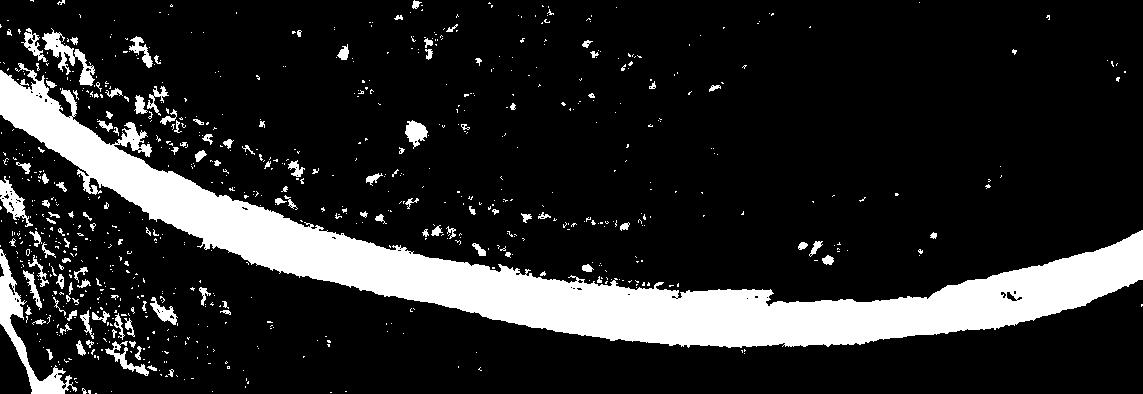

Supplement: S2 Data — (ZIP) [file pone.0297284.s002.zip › Level 1 processed Sample/processed_114/latex/AHA_latex.jpg]

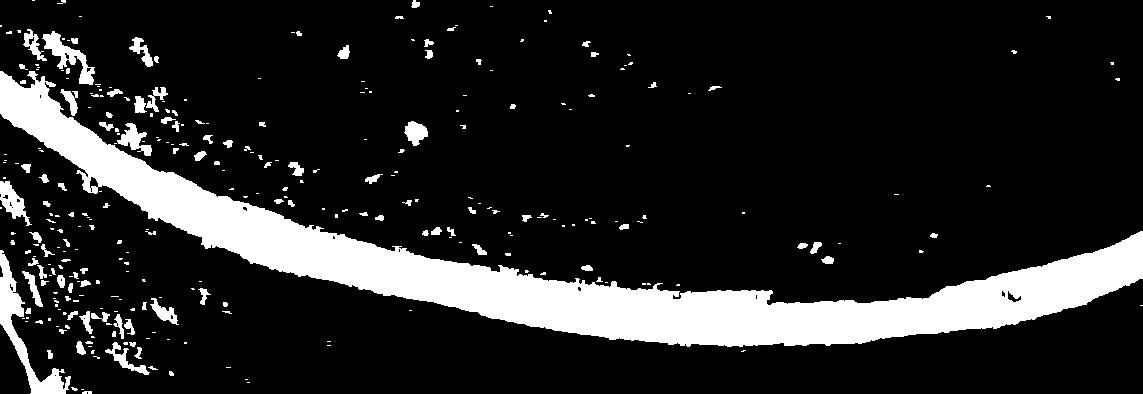

Supplement: S2 Data — (ZIP) [file pone.0297284.s002.zip › Level 1 processed Sample/processed_114/latex/DBO_latex.jpg]

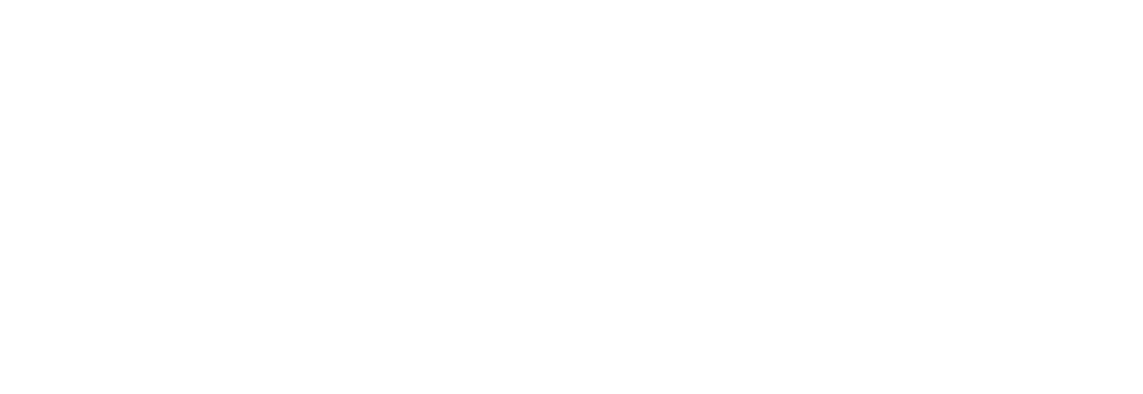

Supplement: S2 Data — (ZIP) [file pone.0297284.s002.zip › Level 1 processed Sample/processed_114/latex/OTSU_latex.jpg]

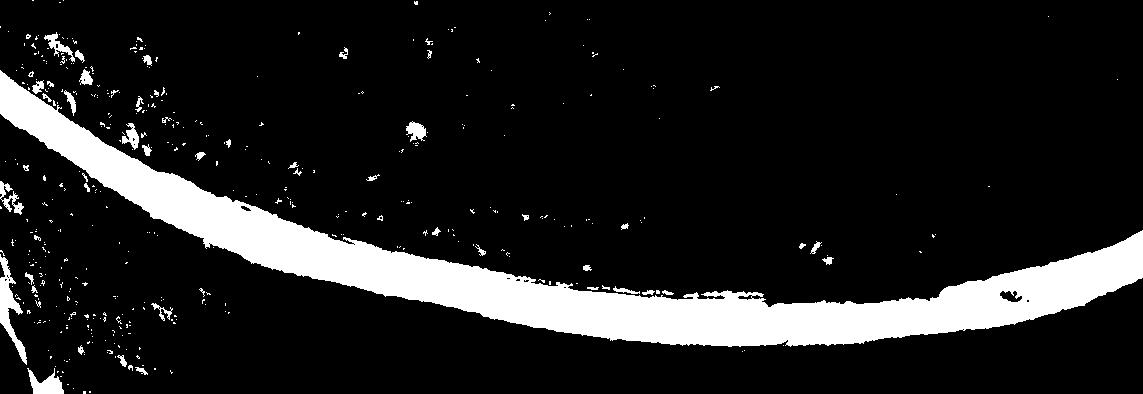

Supplement: S2 Data — (ZIP) [file pone.0297284.s002.zip › Level 1 processed Sample/processed_114/latex/WOA_latex.jpg]

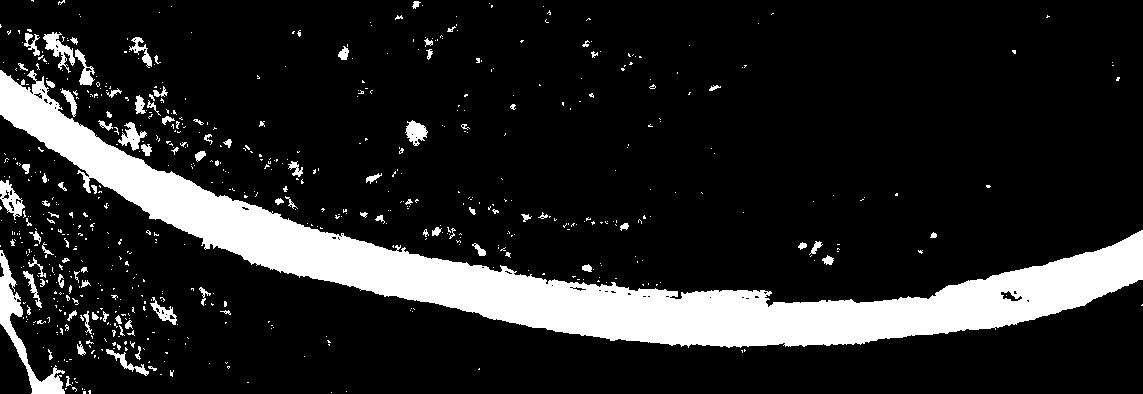

Supplement: S2 Data — (ZIP) [file pone.0297284.s002.zip › Level 1 processed Sample/processed_114/latex/WSO_latex.jpg]

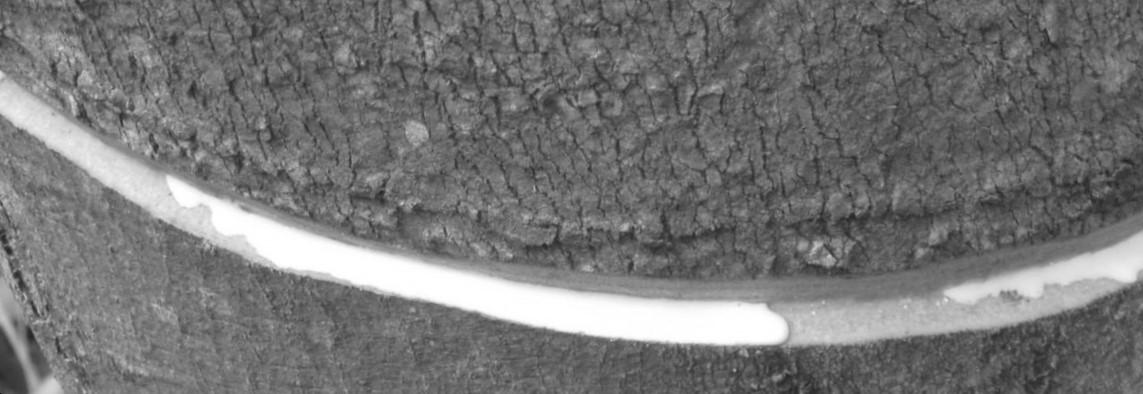

Supplement: S2 Data — (ZIP) [file pone.0297284.s002.zip › Level 1 processed Sample/processed_114/original_image.jpg]

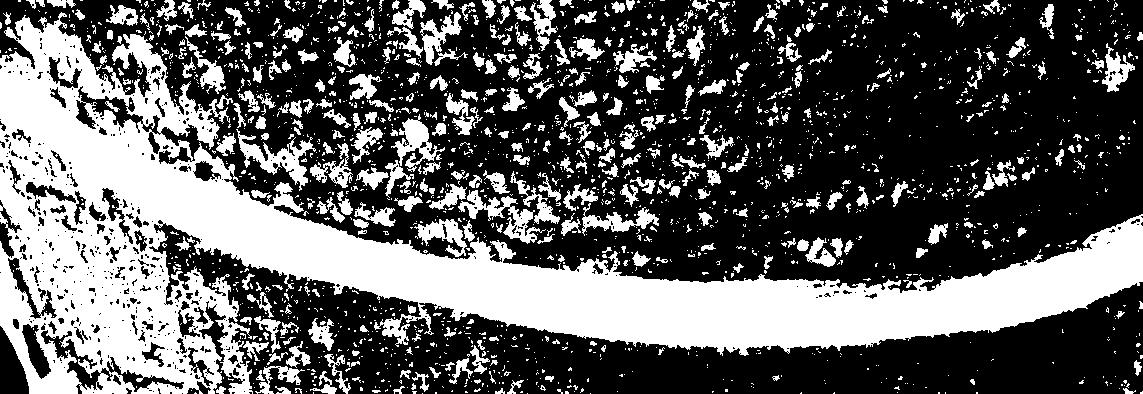

Supplement: S2 Data — (ZIP) [file pone.0297284.s002.zip › Level 1 processed Sample/processed_114/scar/AHA_scar.jpg]

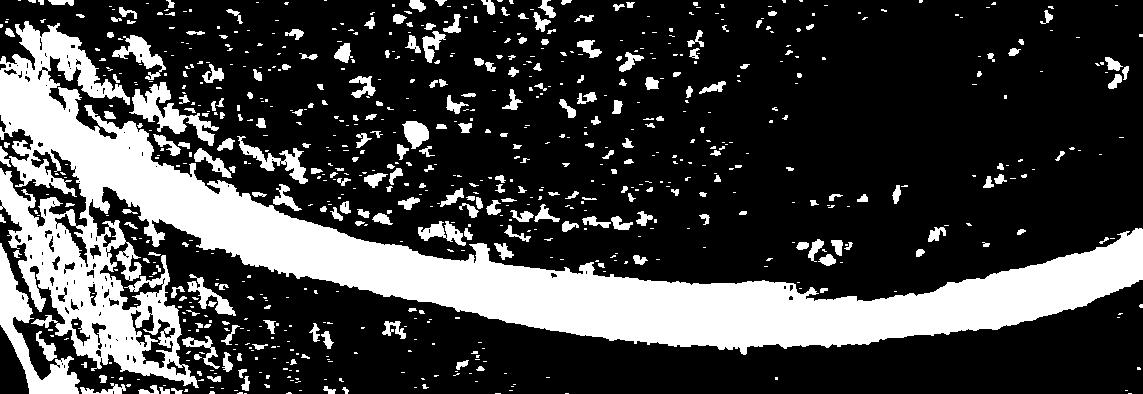

Supplement: S2 Data — (ZIP) [file pone.0297284.s002.zip › Level 1 processed Sample/processed_114/scar/DBO_scar.jpg]

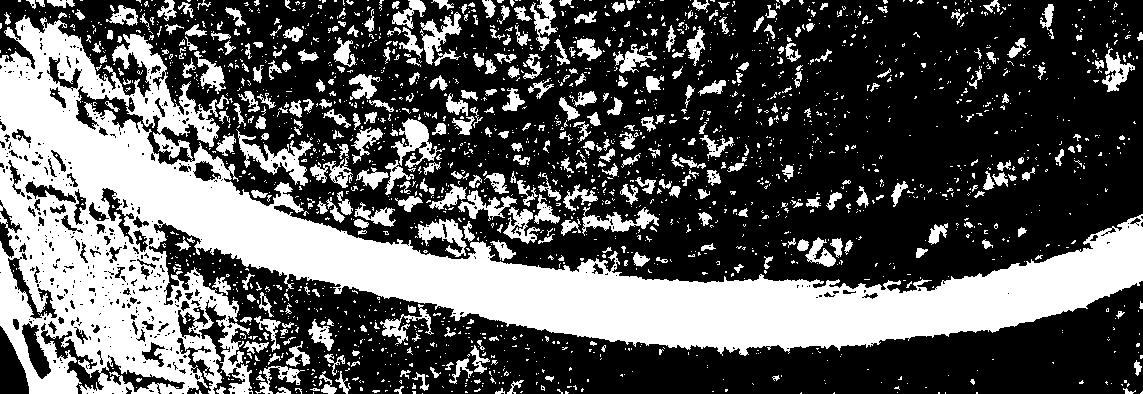

Supplement: S2 Data — (ZIP) [file pone.0297284.s002.zip › Level 1 processed Sample/processed_114/scar/WOA_scar.jpg]

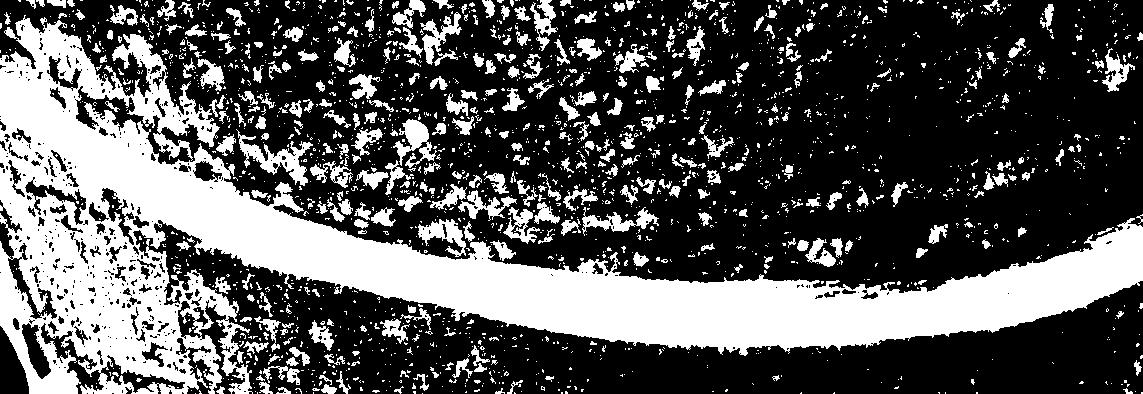

Supplement: S2 Data — (ZIP) [file pone.0297284.s002.zip › Level 1 processed Sample/processed_114/scar/WSO_scar.jpg]

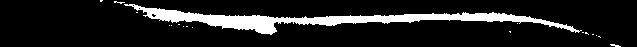

Supplement: S2 Data — (ZIP) [file pone.0297284.s002.zip › Level 1 processed Sample/processed_12/latex/AHA_latex.jpg]

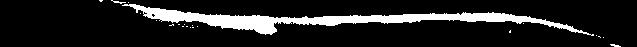

Supplement: S2 Data — (ZIP) [file pone.0297284.s002.zip › Level 1 processed Sample/processed_12/latex/CSA_latex.jpg]

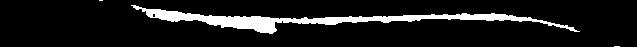

Supplement: S2 Data — (ZIP) [file pone.0297284.s002.zip › Level 1 processed Sample/processed_12/latex/DBO_latex.jpg]

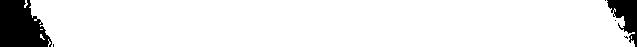

Supplement: S2 Data — (ZIP) [file pone.0297284.s002.zip › Level 1 processed Sample/processed_12/latex/OTSU_latex.jpg]

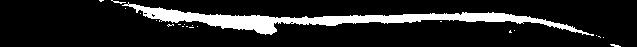

Supplement: S2 Data — (ZIP) [file pone.0297284.s002.zip › Level 1 processed Sample/processed_12/latex/WOA_latex.jpg]

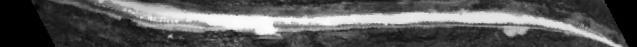

Supplement: S2 Data — (ZIP) [file pone.0297284.s002.zip › Level 1 processed Sample/processed_12/original_image.jpg]

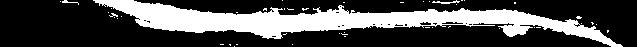

Supplement: S2 Data — (ZIP) [file pone.0297284.s002.zip › Level 1 processed Sample/processed_12/scar/AHA_scar.jpg]
