# Supplementary figures and images for: Advancing image segmentation with DBO-Otsu: Addressing rubber tree diseases through enhanced threshold techniques (part 2 of 7)
Source: PLoS One. 2024 Mar 21;19(3):e0297284. doi: 10.1371/journal.pone.0297284 (PMC10956860; doi:10.1371/journal.pone.0297284)

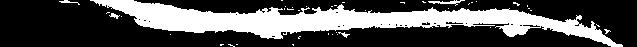

Supplement: S2 Data — (ZIP) [file pone.0297284.s002.zip › Level 1 processed Sample/processed_12/scar/CSA_scar.jpg]

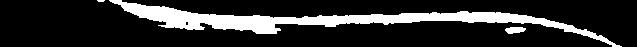

Supplement: S2 Data — (ZIP) [file pone.0297284.s002.zip › Level 1 processed Sample/processed_12/scar/DBO_scar.jpg]

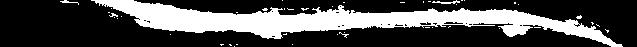

Supplement: S2 Data — (ZIP) [file pone.0297284.s002.zip › Level 1 processed Sample/processed_12/scar/GWO_scar.jpg]

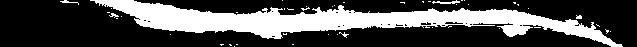

Supplement: S2 Data — (ZIP) [file pone.0297284.s002.zip › Level 1 processed Sample/processed_12/scar/WSO_scar.jpg]

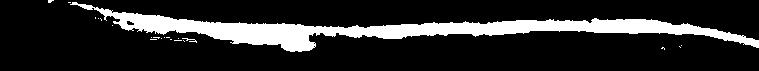

Supplement: S2 Data — (ZIP) [file pone.0297284.s002.zip › Level 1 processed Sample/processed_13/latex/AHA_latex.jpg]

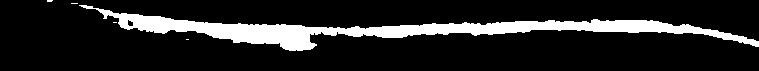

Supplement: S2 Data — (ZIP) [file pone.0297284.s002.zip › Level 1 processed Sample/processed_13/latex/DBO_latex.jpg]

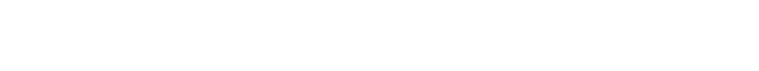

Supplement: S2 Data — (ZIP) [file pone.0297284.s002.zip › Level 1 processed Sample/processed_13/latex/OTSU_latex.jpg]

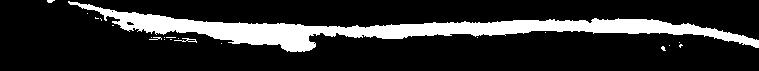

Supplement: S2 Data — (ZIP) [file pone.0297284.s002.zip › Level 1 processed Sample/processed_13/latex/SSA_latex.jpg]

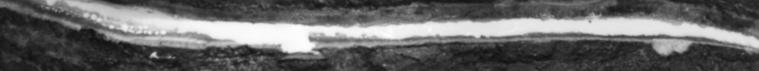

Supplement: S2 Data — (ZIP) [file pone.0297284.s002.zip › Level 1 processed Sample/processed_13/original_image.jpg]

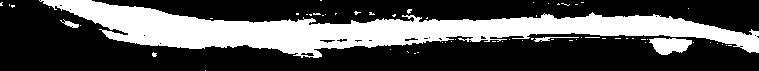

Supplement: S2 Data — (ZIP) [file pone.0297284.s002.zip › Level 1 processed Sample/processed_13/scar/AHA_scar.jpg]

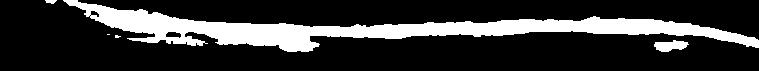

Supplement: S2 Data — (ZIP) [file pone.0297284.s002.zip › Level 1 processed Sample/processed_13/scar/DBO_scar.jpg]

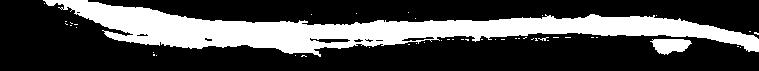

Supplement: S2 Data — (ZIP) [file pone.0297284.s002.zip › Level 1 processed Sample/processed_13/scar/WSO_scar.jpg]

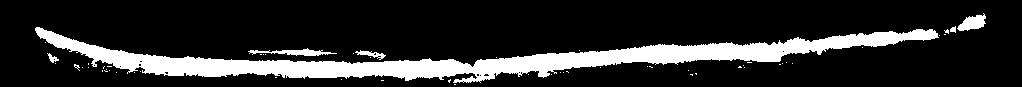

Supplement: S2 Data — (ZIP) [file pone.0297284.s002.zip › Level 1 processed Sample/processed_14/latex/AHA_latex.jpg]

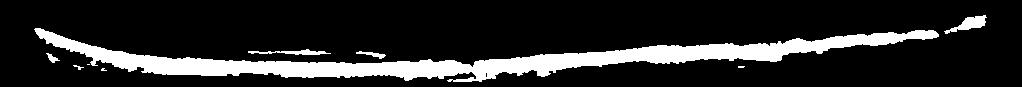

Supplement: S2 Data — (ZIP) [file pone.0297284.s002.zip › Level 1 processed Sample/processed_14/latex/DBO_latex.jpg]

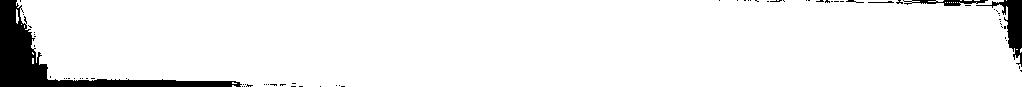

Supplement: S2 Data — (ZIP) [file pone.0297284.s002.zip › Level 1 processed Sample/processed_14/latex/OTSU_latex.jpg]

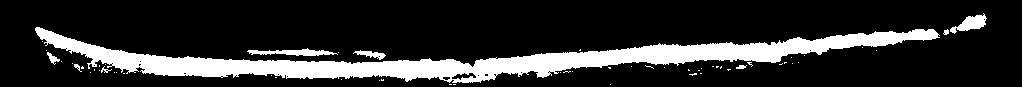

Supplement: S2 Data — (ZIP) [file pone.0297284.s002.zip › Level 1 processed Sample/processed_14/latex/WOA_latex.jpg]

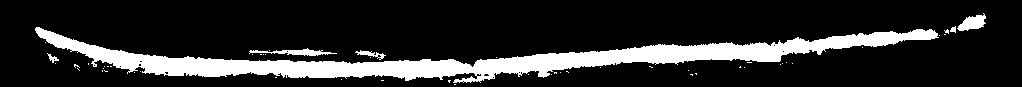

Supplement: S2 Data — (ZIP) [file pone.0297284.s002.zip › Level 1 processed Sample/processed_14/latex/WSO_latex.jpg]

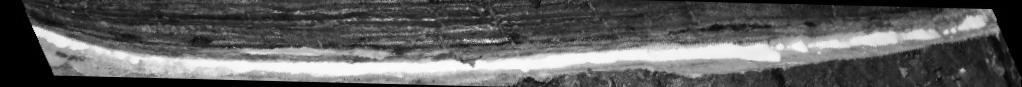

Supplement: S2 Data — (ZIP) [file pone.0297284.s002.zip › Level 1 processed Sample/processed_14/original_image.jpg]

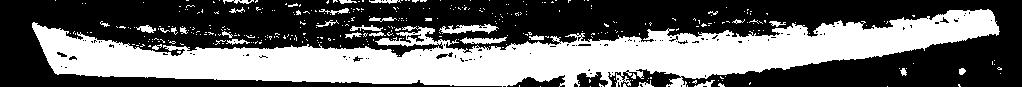

Supplement: S2 Data — (ZIP) [file pone.0297284.s002.zip › Level 1 processed Sample/processed_14/scar/AHA_scar.jpg]

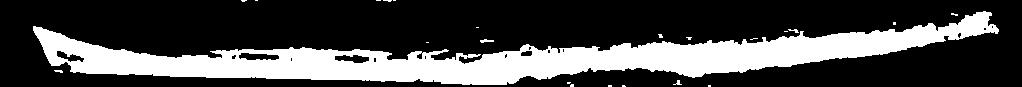

Supplement: S2 Data — (ZIP) [file pone.0297284.s002.zip › Level 1 processed Sample/processed_14/scar/DBO_scar.jpg]

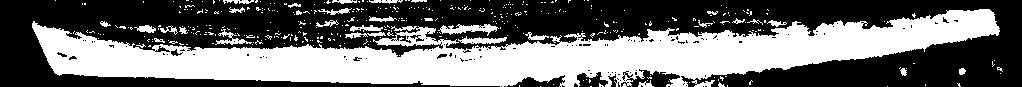

Supplement: S2 Data — (ZIP) [file pone.0297284.s002.zip › Level 1 processed Sample/processed_14/scar/WSO_scar.jpg]

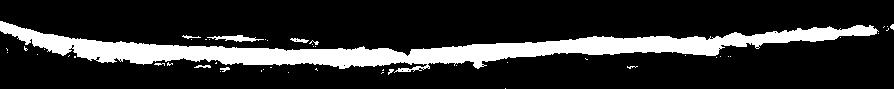

Supplement: S2 Data — (ZIP) [file pone.0297284.s002.zip › Level 1 processed Sample/processed_15/latex/AHA_latex.jpg]

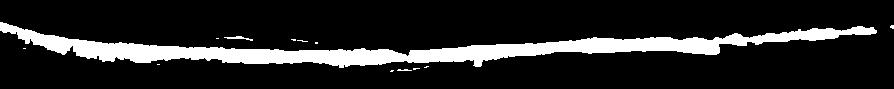

Supplement: S2 Data — (ZIP) [file pone.0297284.s002.zip › Level 1 processed Sample/processed_15/latex/DBO_latex.jpg]

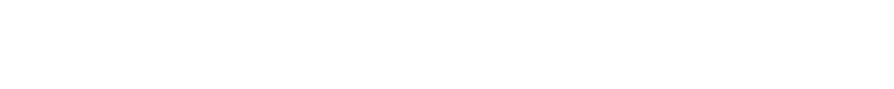

Supplement: S2 Data — (ZIP) [file pone.0297284.s002.zip › Level 1 processed Sample/processed_15/latex/OTSU_latex.jpg]

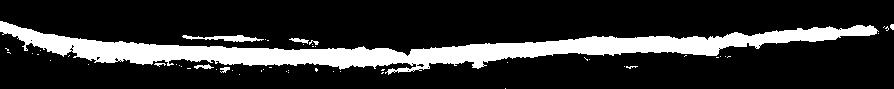

Supplement: S2 Data — (ZIP) [file pone.0297284.s002.zip › Level 1 processed Sample/processed_15/latex/WSO_latex.jpg]

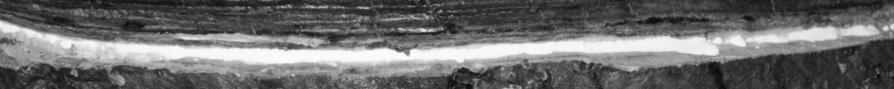

Supplement: S2 Data — (ZIP) [file pone.0297284.s002.zip › Level 1 processed Sample/processed_15/original_image.jpg]

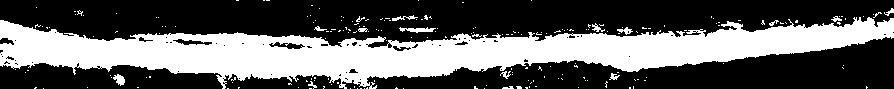

Supplement: S2 Data — (ZIP) [file pone.0297284.s002.zip › Level 1 processed Sample/processed_15/scar/AHA_scar.jpg]

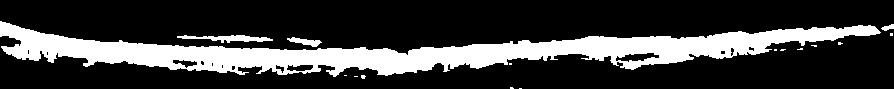

Supplement: S2 Data — (ZIP) [file pone.0297284.s002.zip › Level 1 processed Sample/processed_15/scar/DBO_scar.jpg]

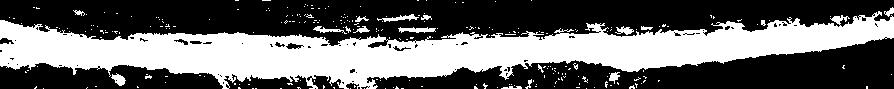

Supplement: S2 Data — (ZIP) [file pone.0297284.s002.zip › Level 1 processed Sample/processed_15/scar/WSO_scar.jpg]

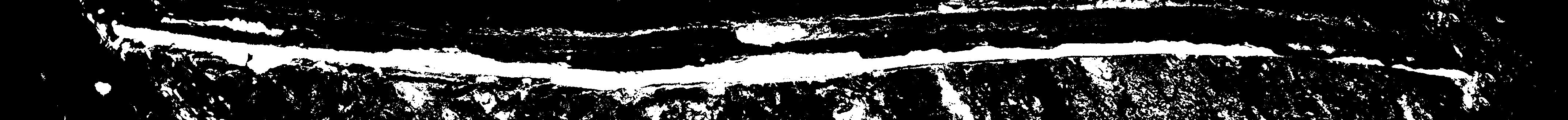

Supplement: S2 Data — (ZIP) [file pone.0297284.s002.zip › Level 1 processed Sample/processed_18/latex/AHA_latex.jpg]

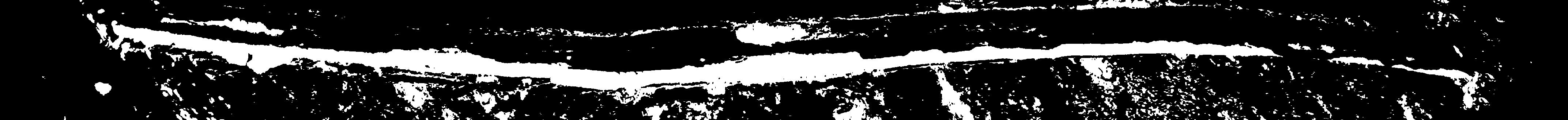

Supplement: S2 Data — (ZIP) [file pone.0297284.s002.zip › Level 1 processed Sample/processed_18/latex/DBO_latex.jpg]

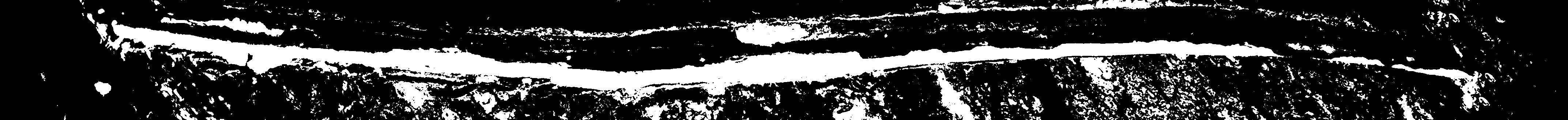

Supplement: S2 Data — (ZIP) [file pone.0297284.s002.zip › Level 1 processed Sample/processed_18/latex/GWO_latex.jpg]

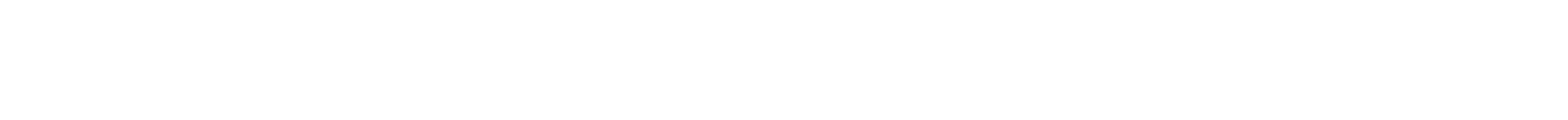

Supplement: S2 Data — (ZIP) [file pone.0297284.s002.zip › Level 1 processed Sample/processed_18/latex/OTSU_latex.jpg]

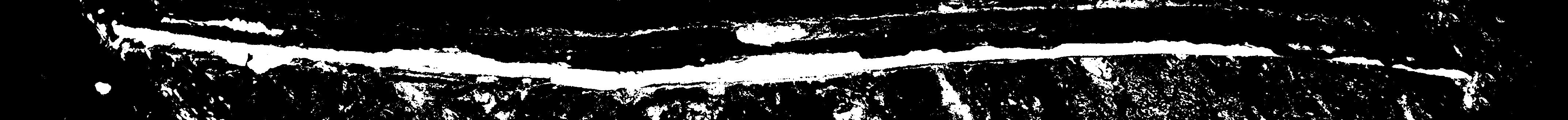

Supplement: S2 Data — (ZIP) [file pone.0297284.s002.zip › Level 1 processed Sample/processed_18/latex/WSO_latex.jpg]

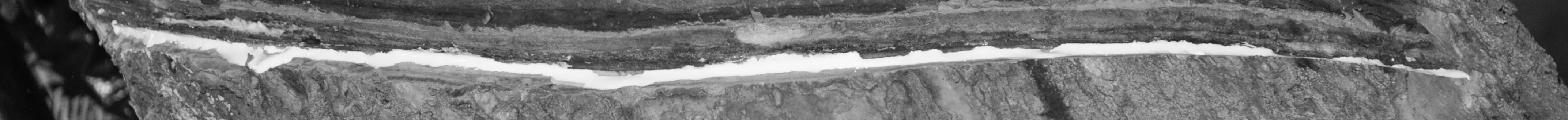

Supplement: S2 Data — (ZIP) [file pone.0297284.s002.zip › Level 1 processed Sample/processed_18/original_image.jpg]

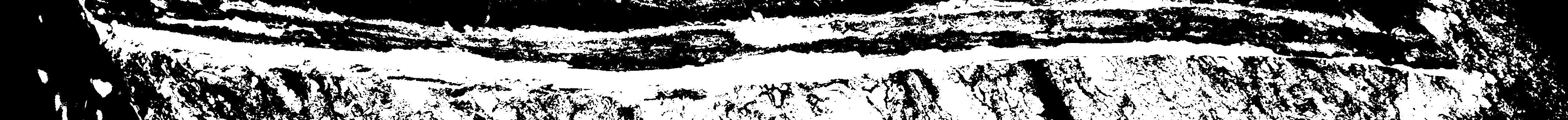

Supplement: S2 Data — (ZIP) [file pone.0297284.s002.zip › Level 1 processed Sample/processed_18/scar/AHA_scar.jpg]

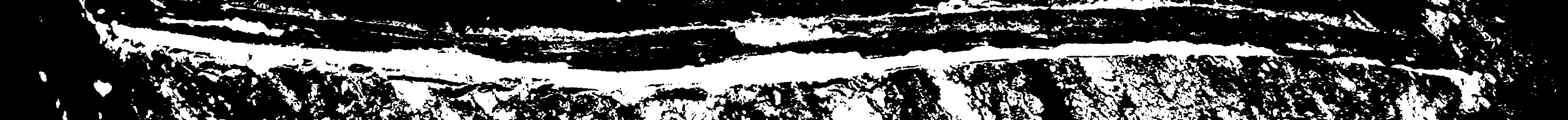

Supplement: S2 Data — (ZIP) [file pone.0297284.s002.zip › Level 1 processed Sample/processed_18/scar/DBO_scar.jpg]

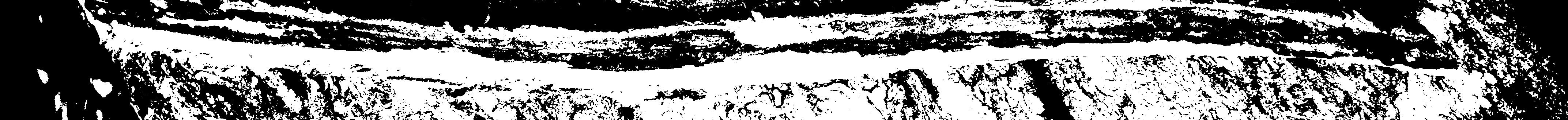

Supplement: S2 Data — (ZIP) [file pone.0297284.s002.zip › Level 1 processed Sample/processed_18/scar/GWO_scar.jpg]

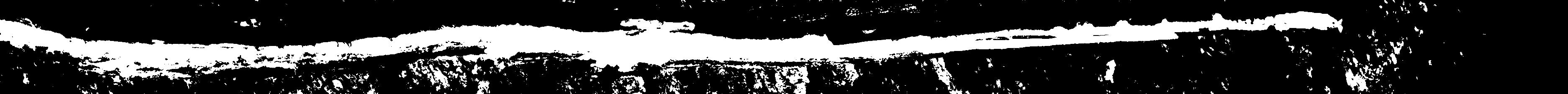

Supplement: S2 Data — (ZIP) [file pone.0297284.s002.zip › Level 1 processed Sample/processed_19/latex/AHA_latex.jpg]

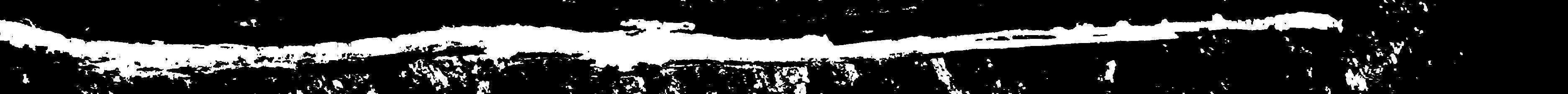

Supplement: S2 Data — (ZIP) [file pone.0297284.s002.zip › Level 1 processed Sample/processed_19/latex/DBO_latex.jpg]

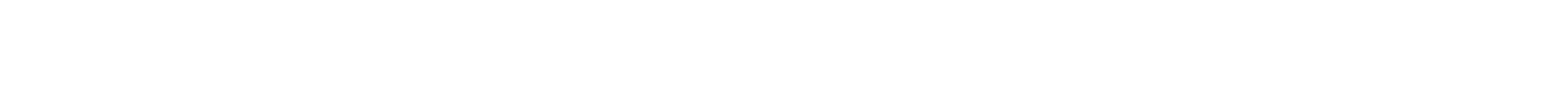

Supplement: S2 Data — (ZIP) [file pone.0297284.s002.zip › Level 1 processed Sample/processed_19/latex/OTSU_latex.jpg]

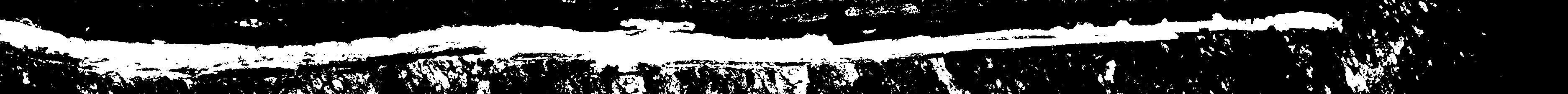

Supplement: S2 Data — (ZIP) [file pone.0297284.s002.zip › Level 1 processed Sample/processed_19/latex/WSO_latex.jpg]

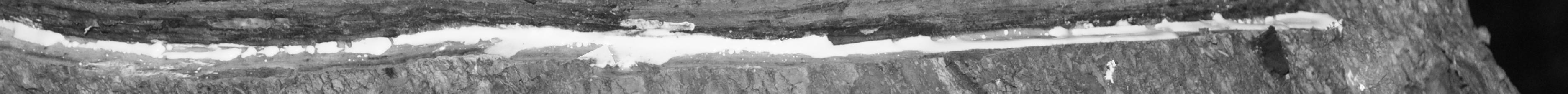

Supplement: S2 Data — (ZIP) [file pone.0297284.s002.zip › Level 1 processed Sample/processed_19/original_image.jpg]

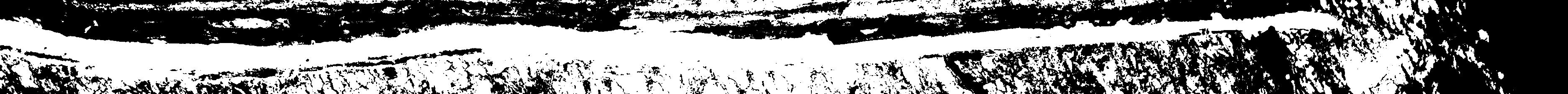

Supplement: S2 Data — (ZIP) [file pone.0297284.s002.zip › Level 1 processed Sample/processed_19/scar/AHA_scar.jpg]

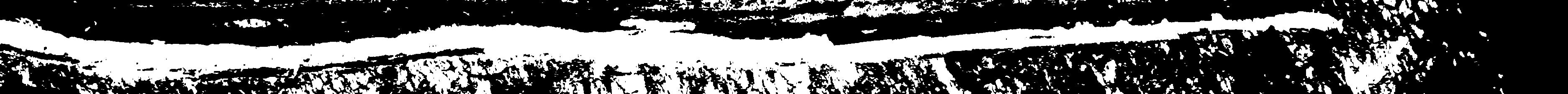

Supplement: S2 Data — (ZIP) [file pone.0297284.s002.zip › Level 1 processed Sample/processed_19/scar/DBO_scar.jpg]

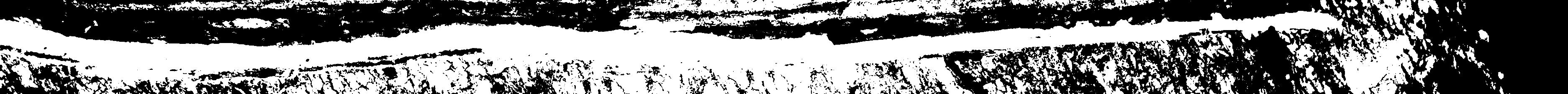

Supplement: S2 Data — (ZIP) [file pone.0297284.s002.zip › Level 1 processed Sample/processed_19/scar/WSO_scar.jpg]

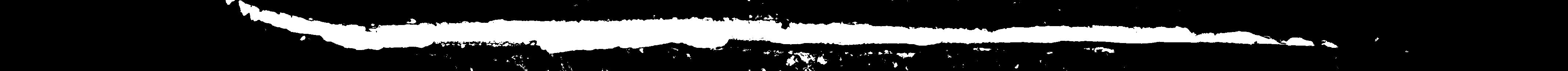

Supplement: S2 Data — (ZIP) [file pone.0297284.s002.zip › Level 1 processed Sample/processed_20/latex/AHA_latex.jpg]

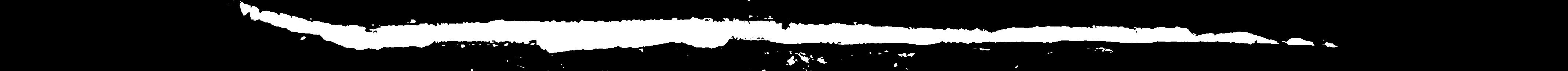

Supplement: S2 Data — (ZIP) [file pone.0297284.s002.zip › Level 1 processed Sample/processed_20/latex/DBO_latex.jpg]

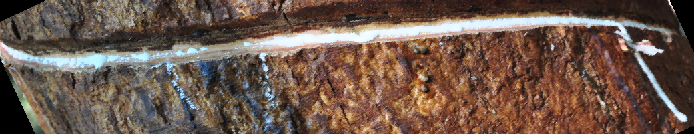

Supplement: S3 Data — (ZIP) [file pone.0297284.s003.zip › Level 2 Original Sample/2-1.jpg]

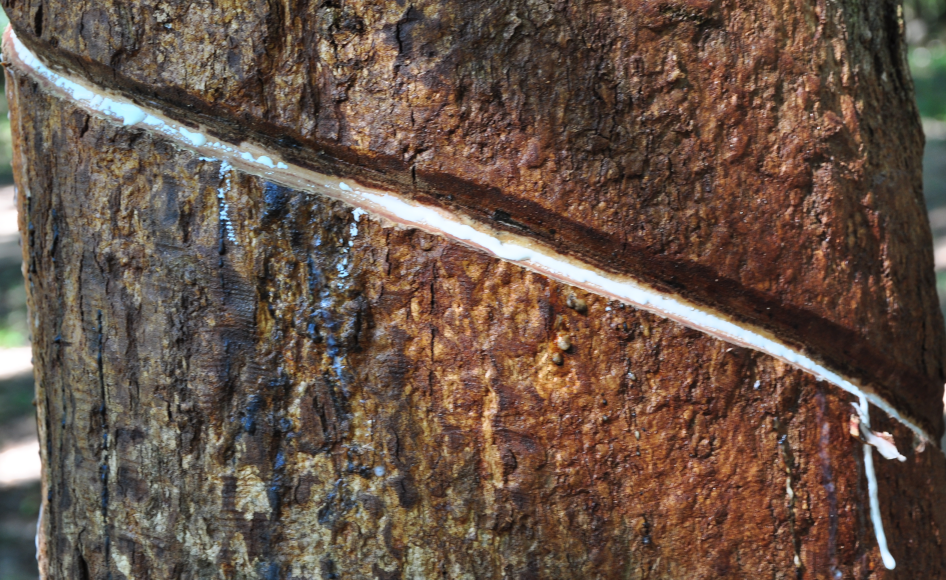

Supplement: S3 Data — (ZIP) [file pone.0297284.s003.zip › Level 2 Original Sample/2-1.png]

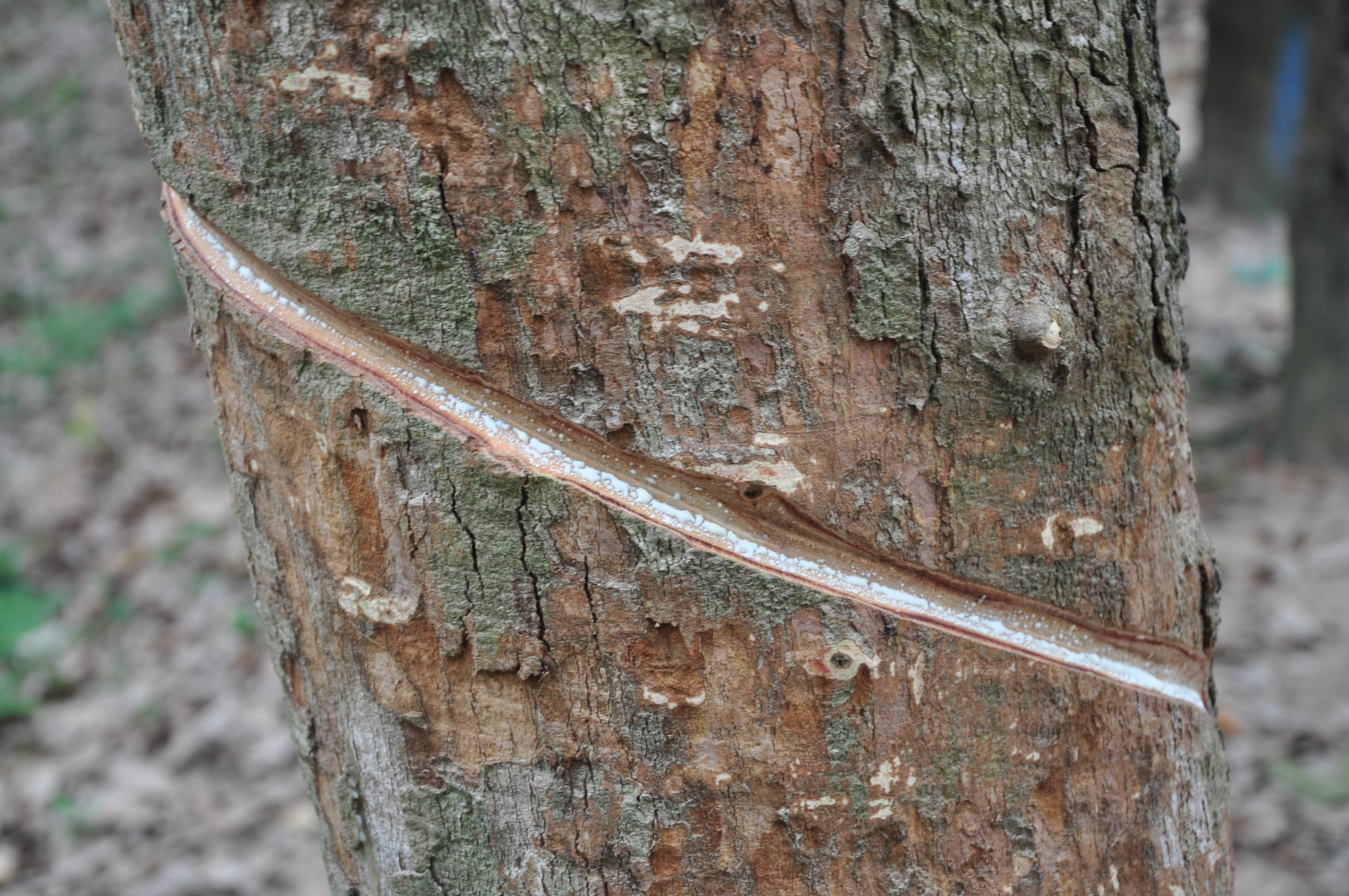

Supplement: S3 Data — (ZIP) [file pone.0297284.s003.zip › Level 2 Original Sample/2-107-20140528-0223.JPG]

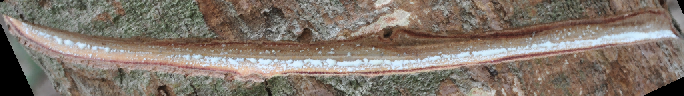

Supplement: S3 Data — (ZIP) [file pone.0297284.s003.zip › Level 2 Original Sample/2-2.jpg]

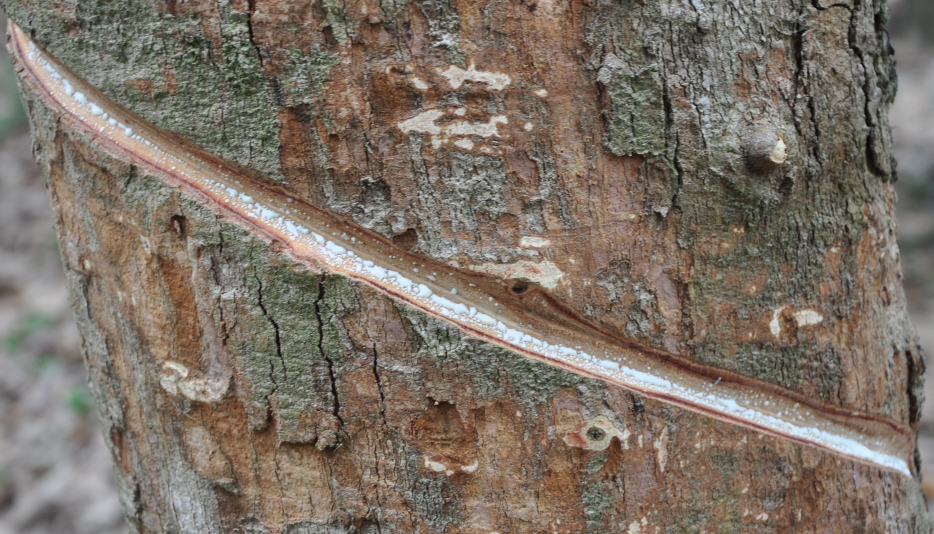

Supplement: S3 Data — (ZIP) [file pone.0297284.s003.zip › Level 2 Original Sample/2-2.png]

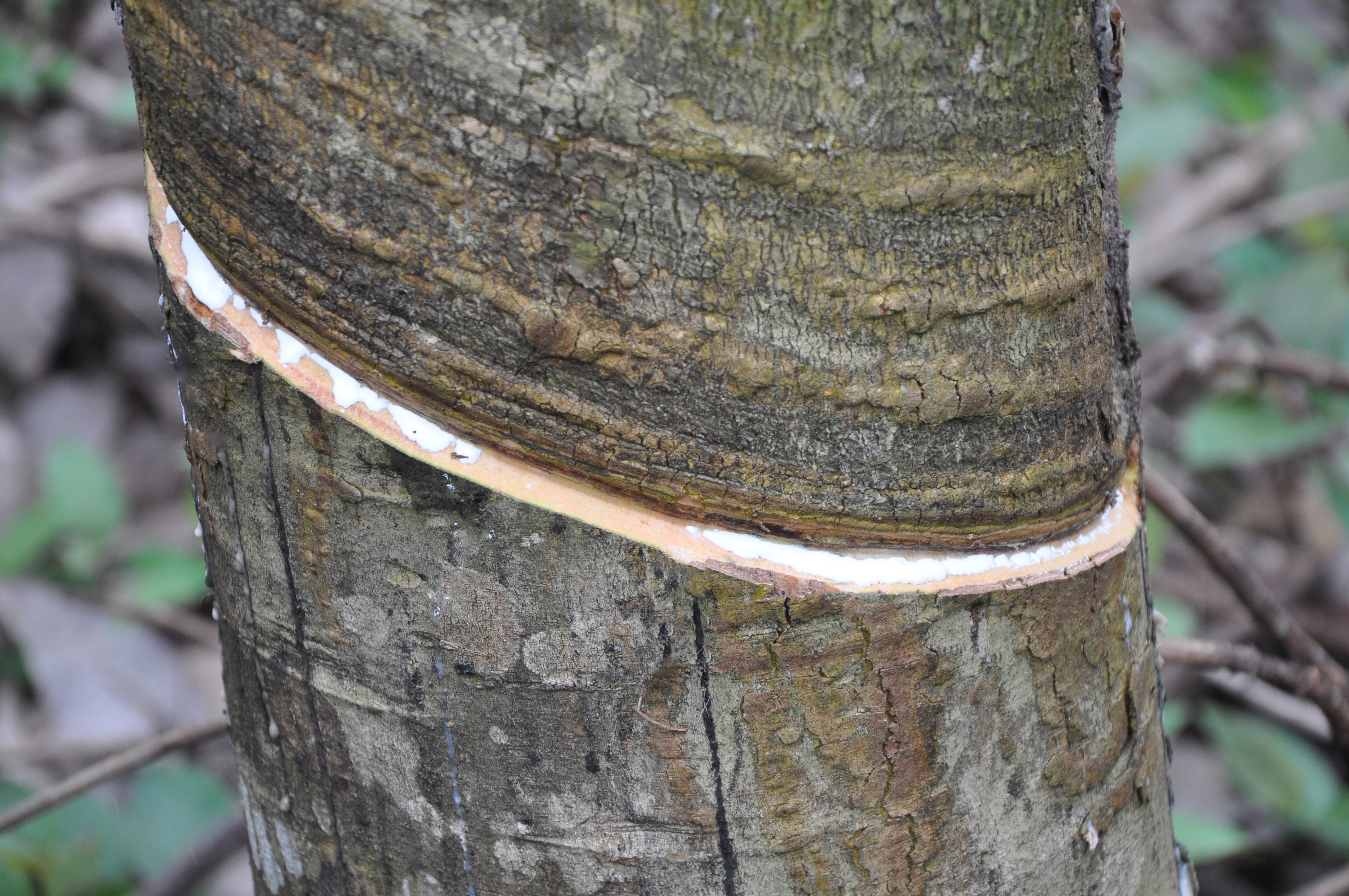

Supplement: S3 Data — (ZIP) [file pone.0297284.s003.zip › Level 2 Original Sample/2-33702-074-20141126-0097.JPG]

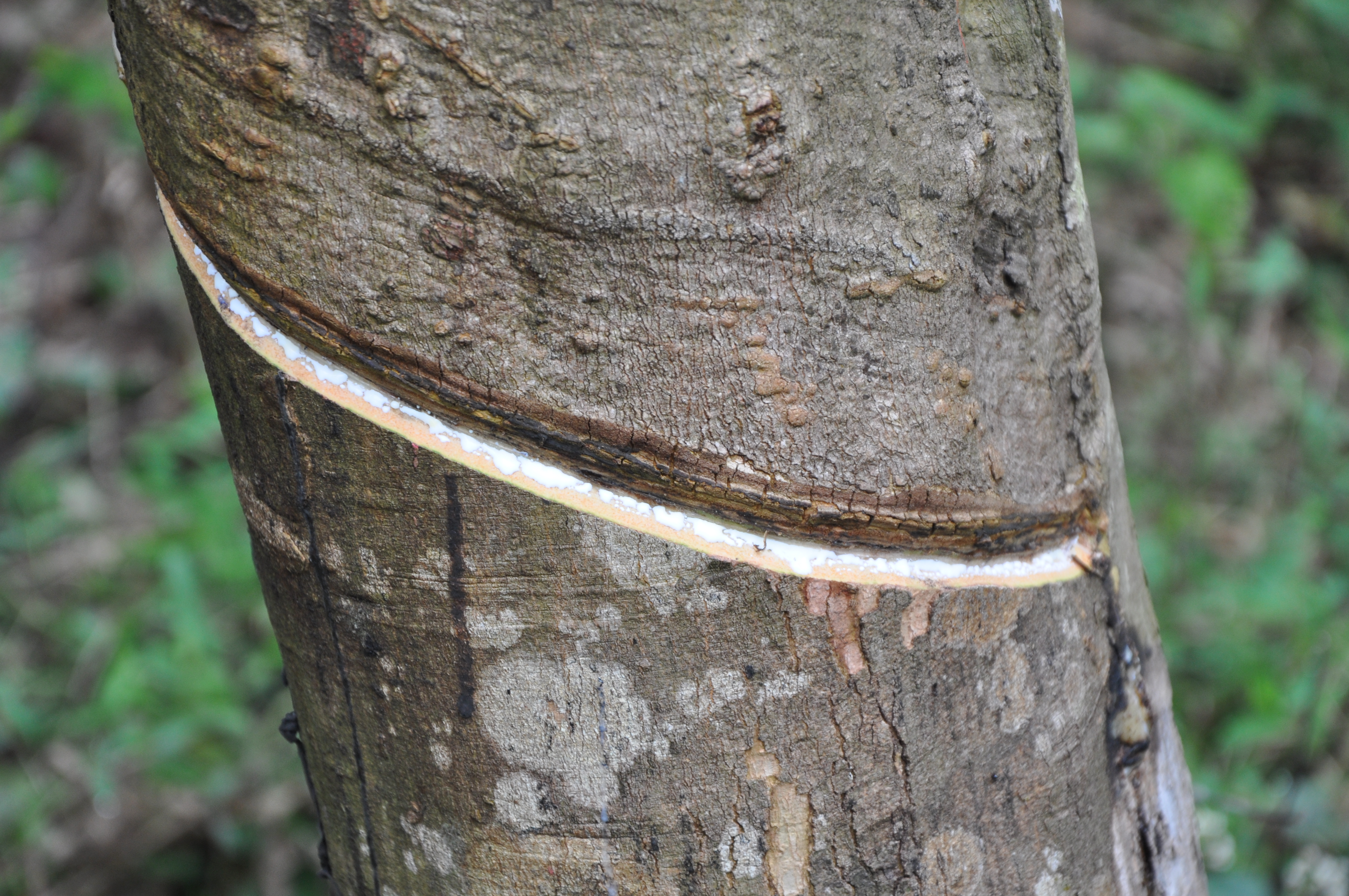

Supplement: S3 Data — (ZIP) [file pone.0297284.s003.zip › Level 2 Original Sample/2-3372-072-20141126-0095.JPG]

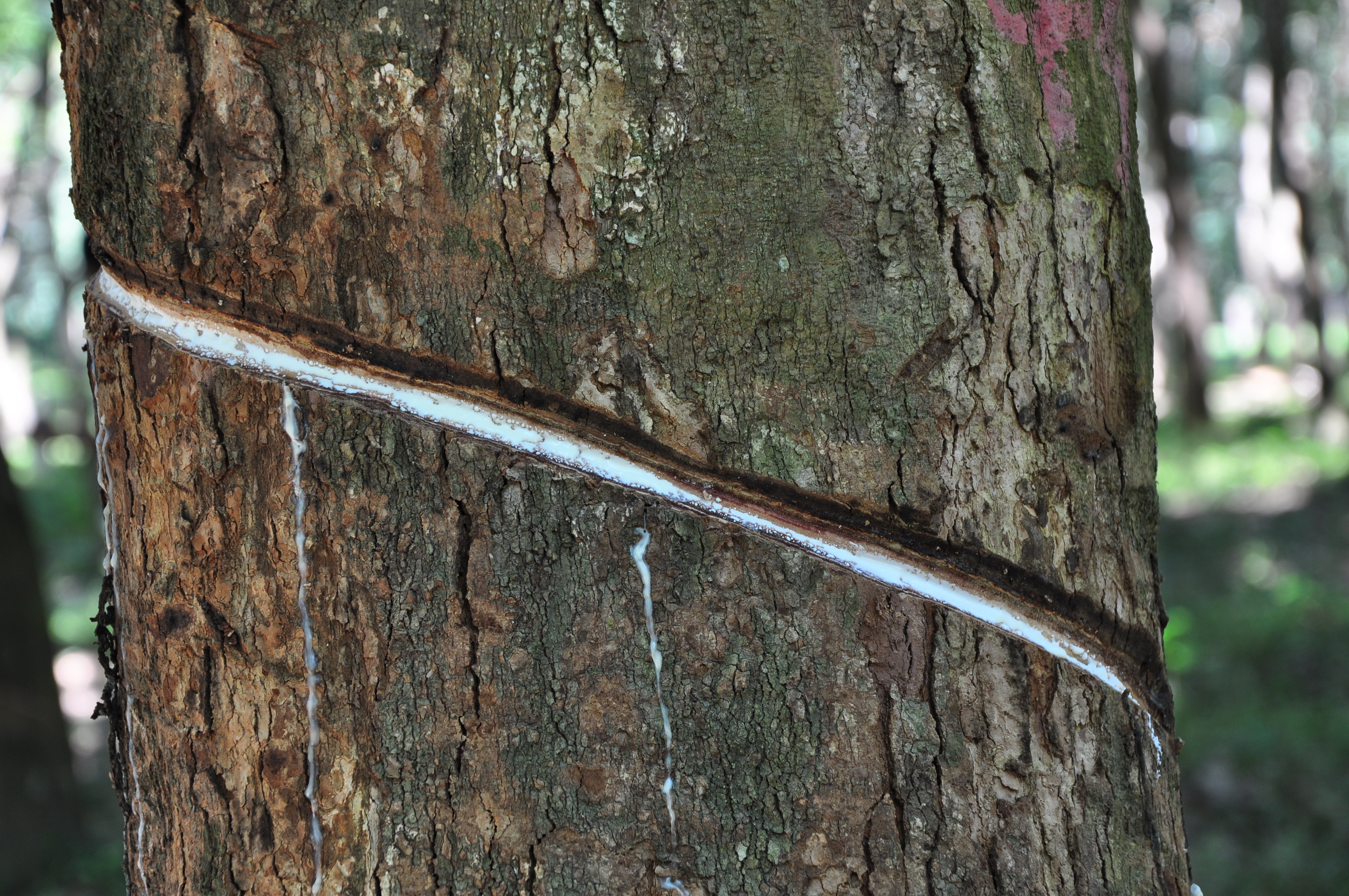

Supplement: S3 Data — (ZIP) [file pone.0297284.s003.zip › Level 2 Original Sample/2-435-20140904-0384.JPG]

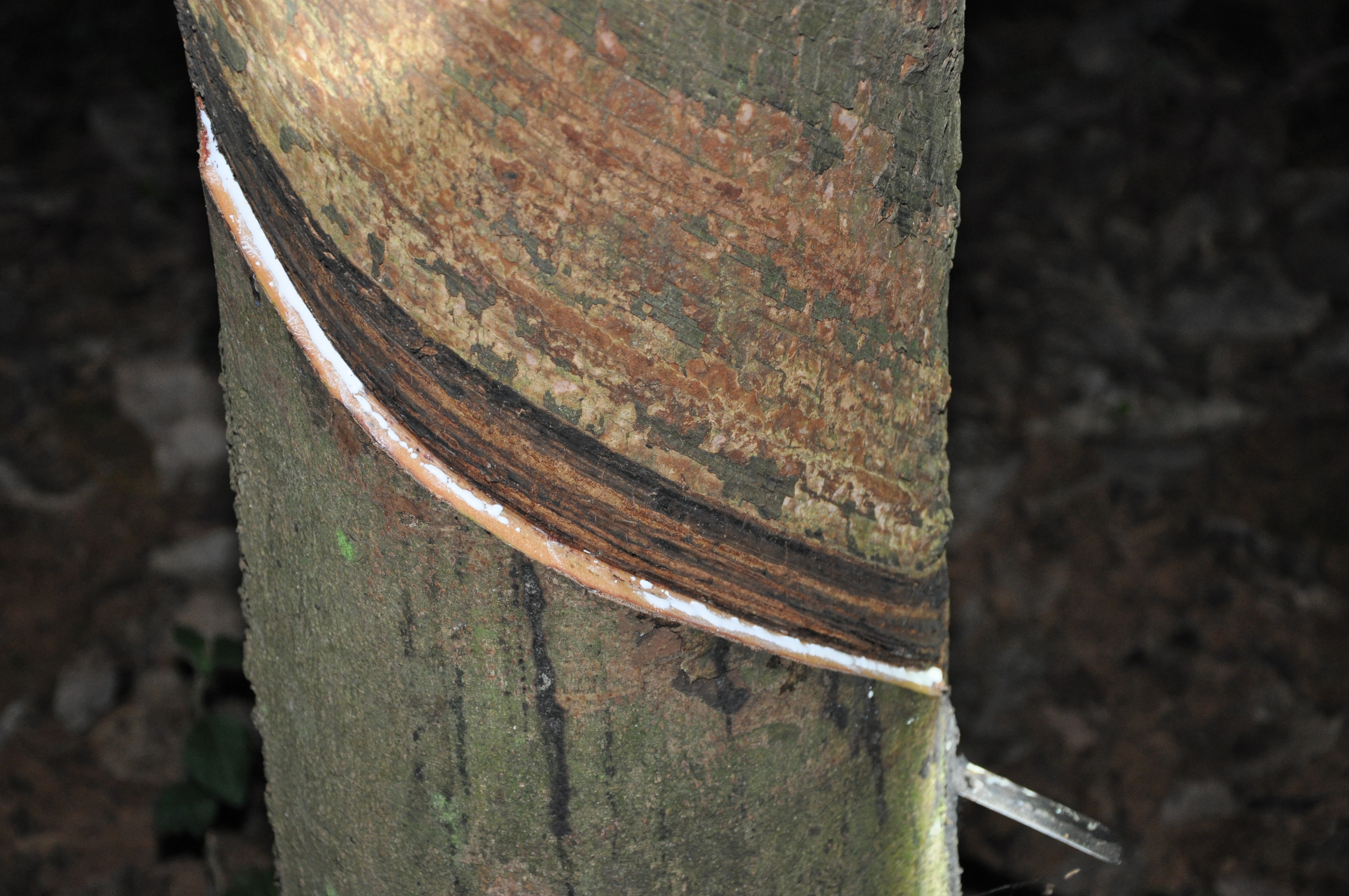

Supplement: S3 Data — (ZIP) [file pone.0297284.s003.zip › Level 2 Original Sample/2-61602-150-20141119-0008.JPG]

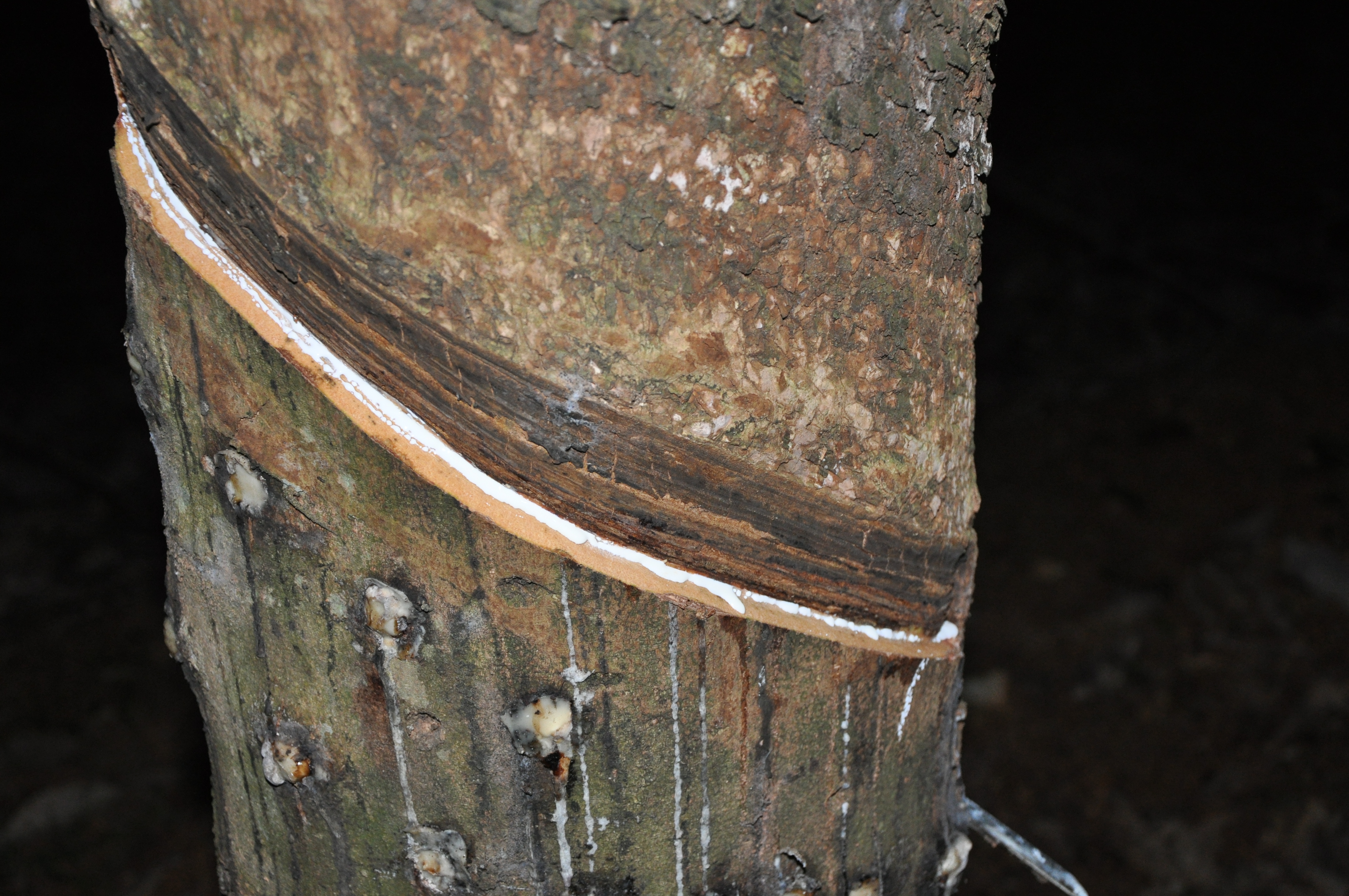

Supplement: S3 Data — (ZIP) [file pone.0297284.s003.zip › Level 2 Original Sample/2-61602-179-20141119-0042.JPG]

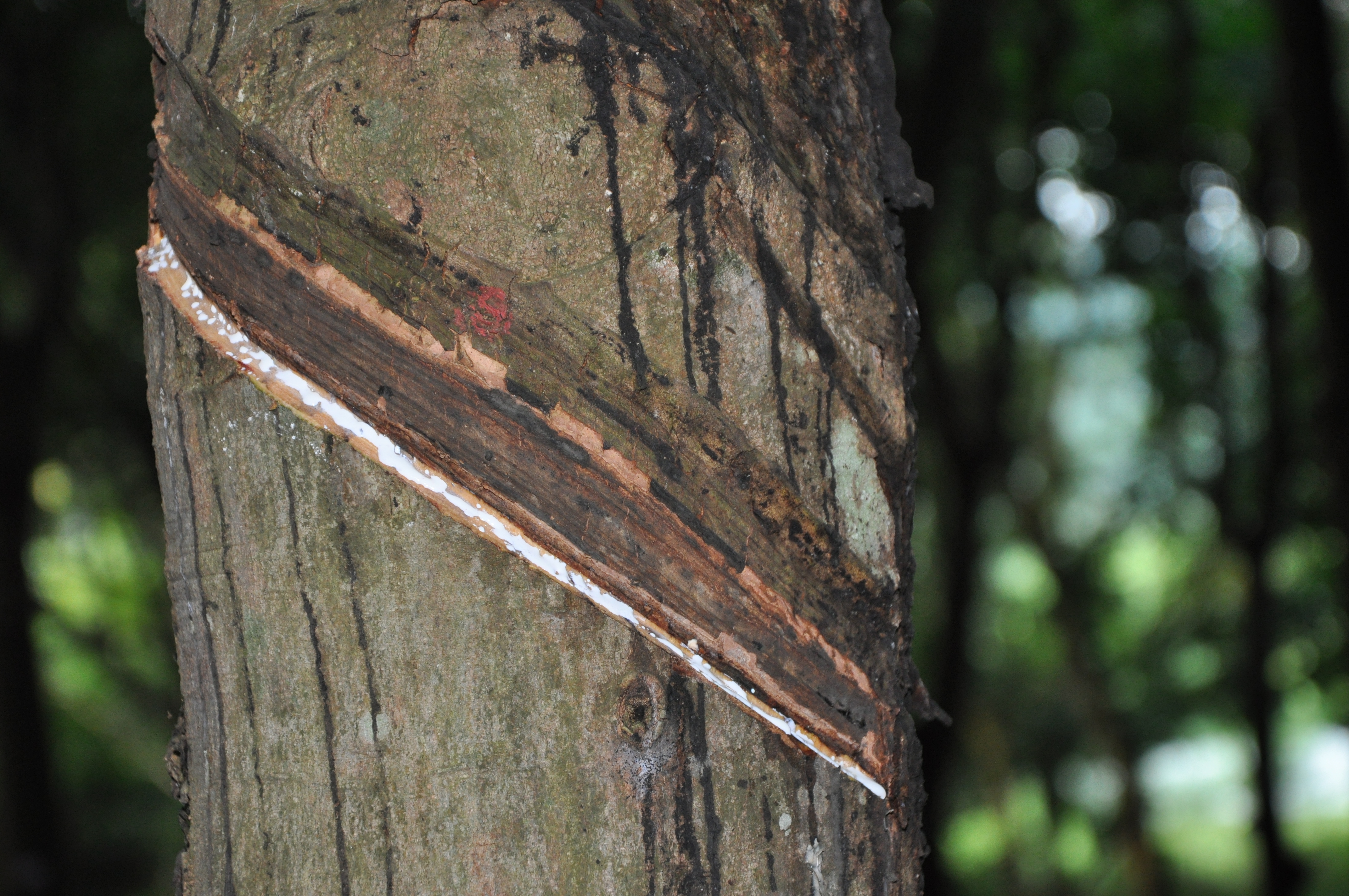

Supplement: S3 Data — (ZIP) [file pone.0297284.s003.zip › Level 2 Original Sample/2-61602-289-20140901-121.JPG]

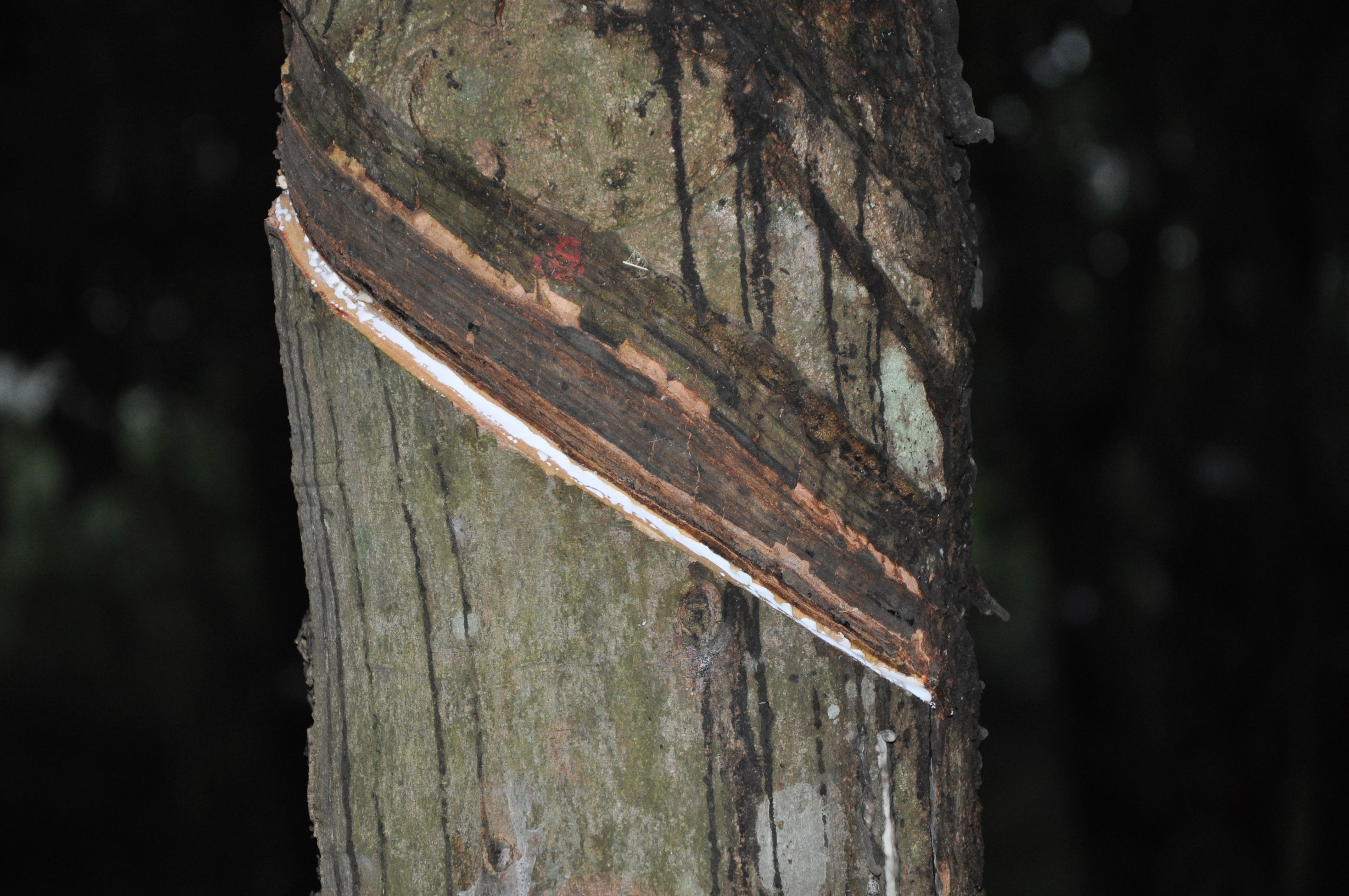

Supplement: S3 Data — (ZIP) [file pone.0297284.s003.zip › Level 2 Original Sample/2-61602-289-20140928-0031.JPG]

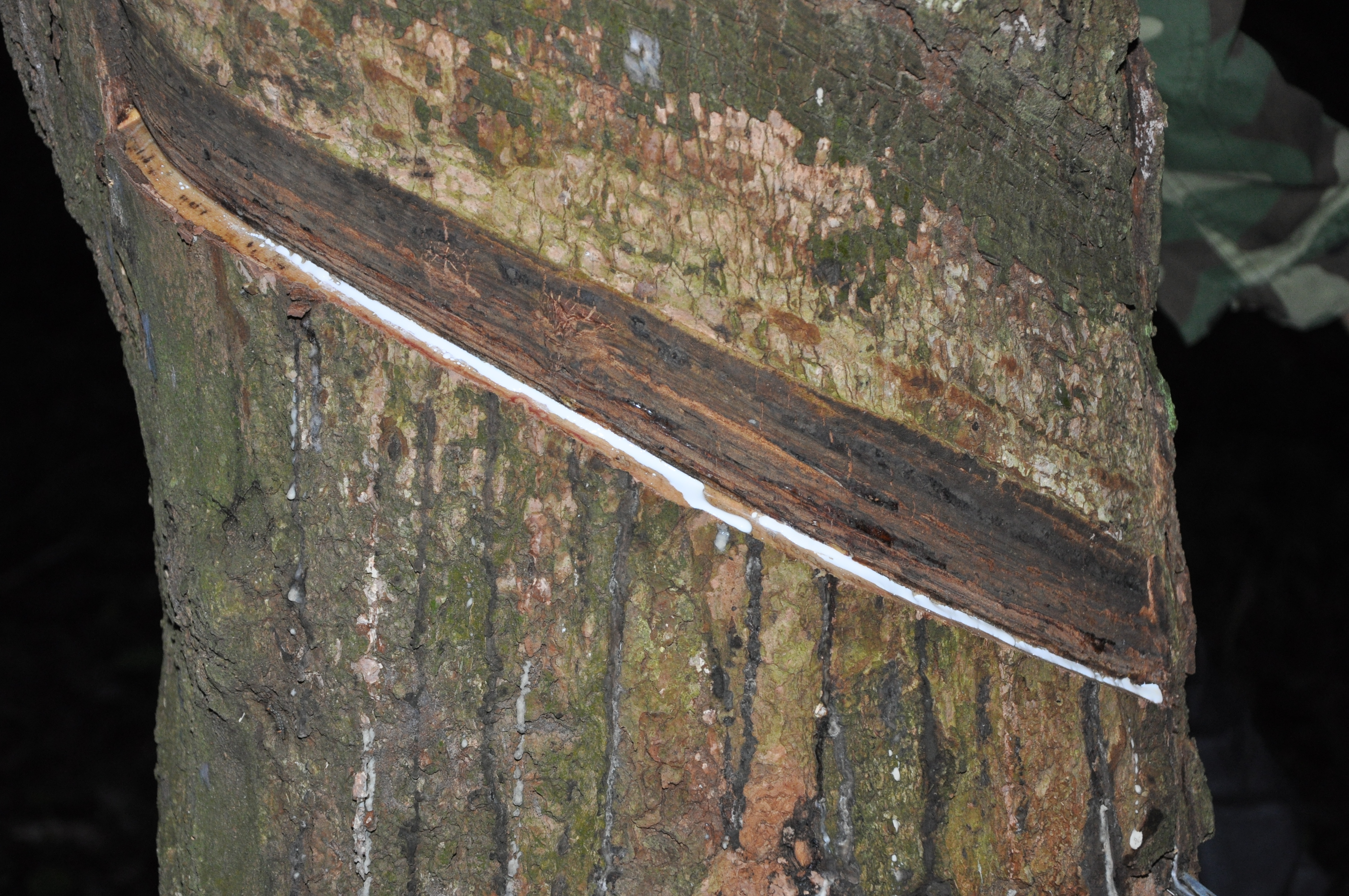

Supplement: S3 Data — (ZIP) [file pone.0297284.s003.zip › Level 2 Original Sample/2-61602-299-20141028-0069.JPG]

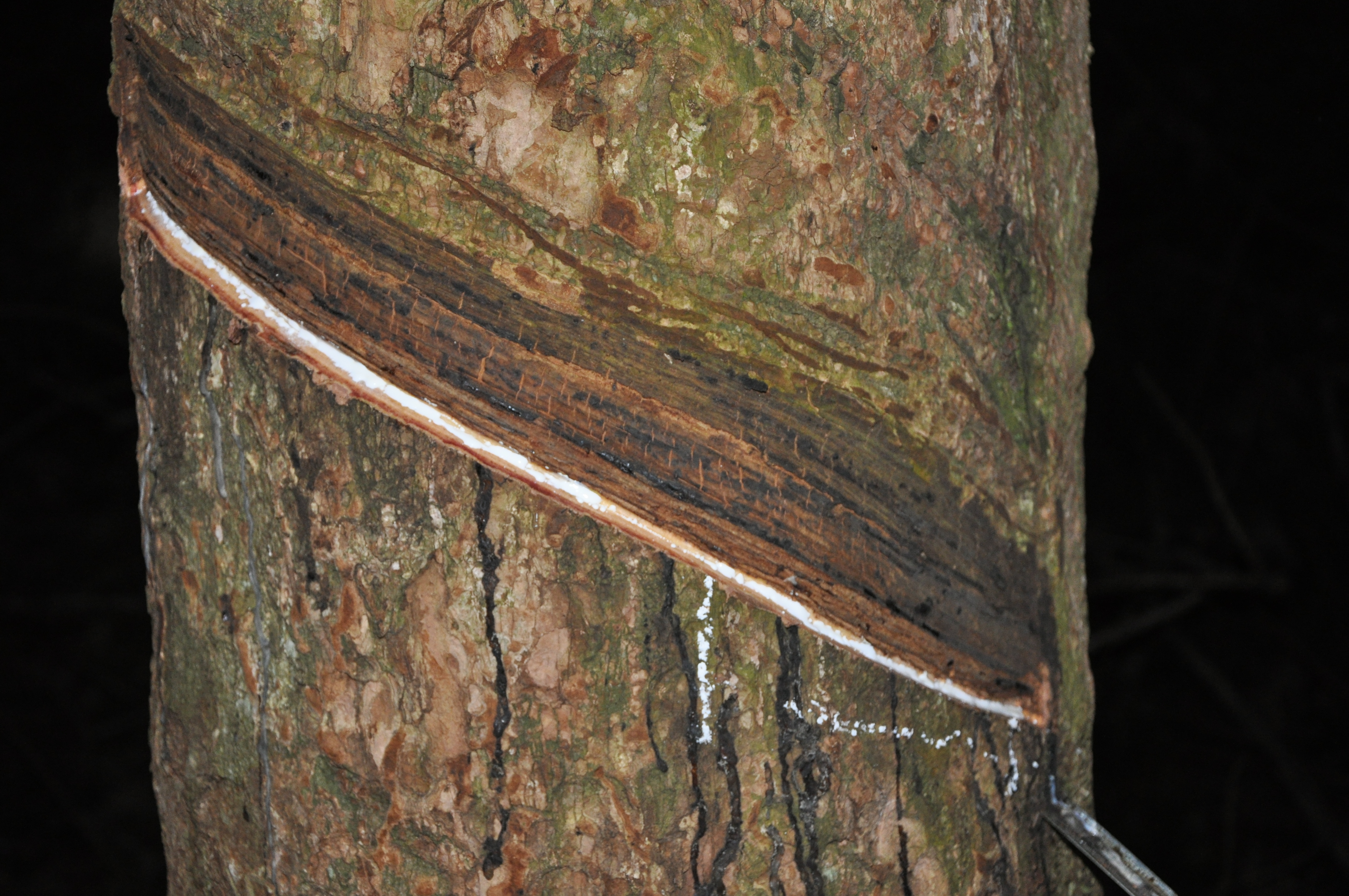

Supplement: S3 Data — (ZIP) [file pone.0297284.s003.zip › Level 2 Original Sample/2-61602-301-20141119-0067.JPG]

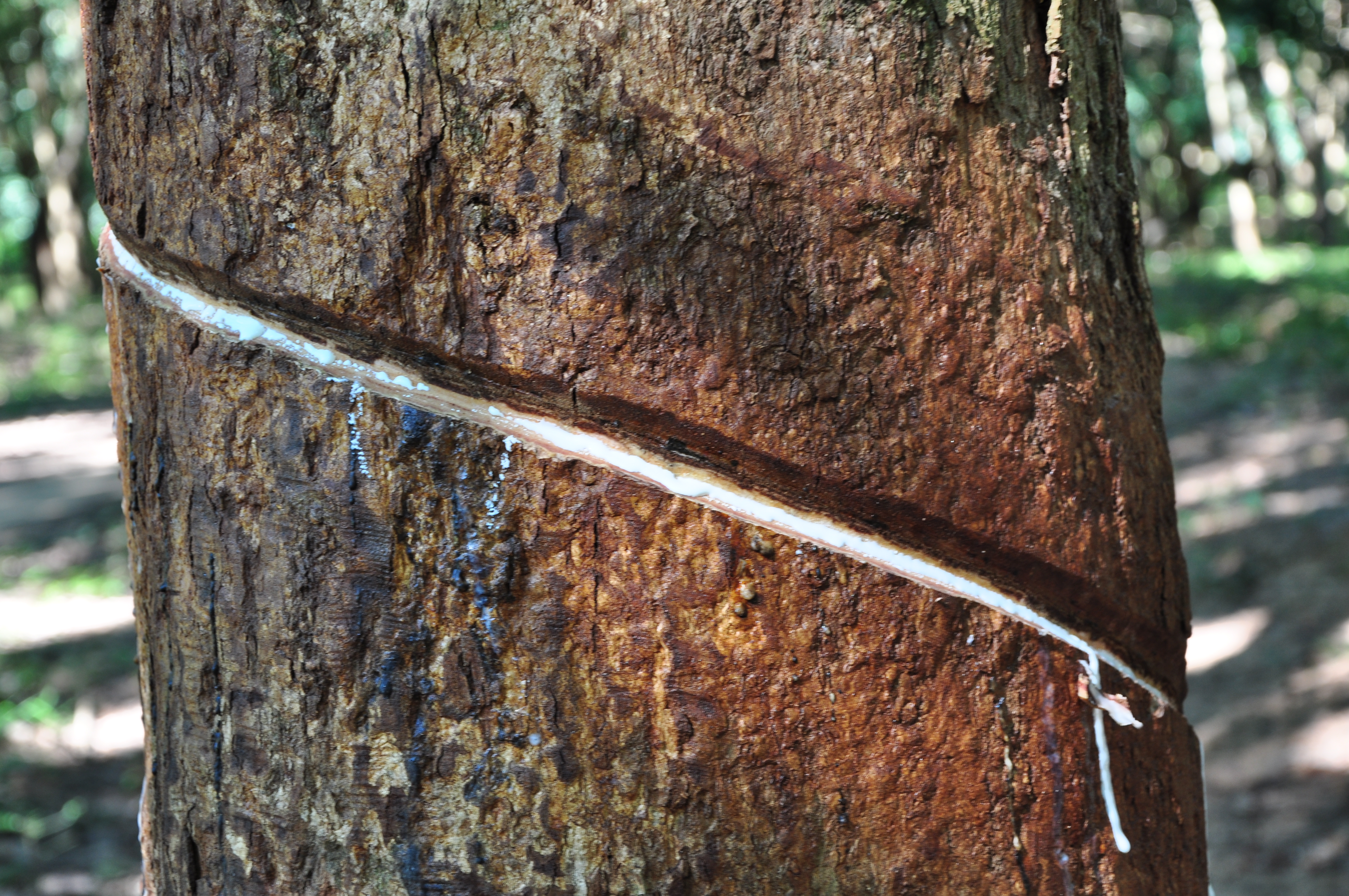

Supplement: S3 Data — (ZIP) [file pone.0297284.s003.zip › Level 2 Original Sample/2-65-20170904-0141.JPG]

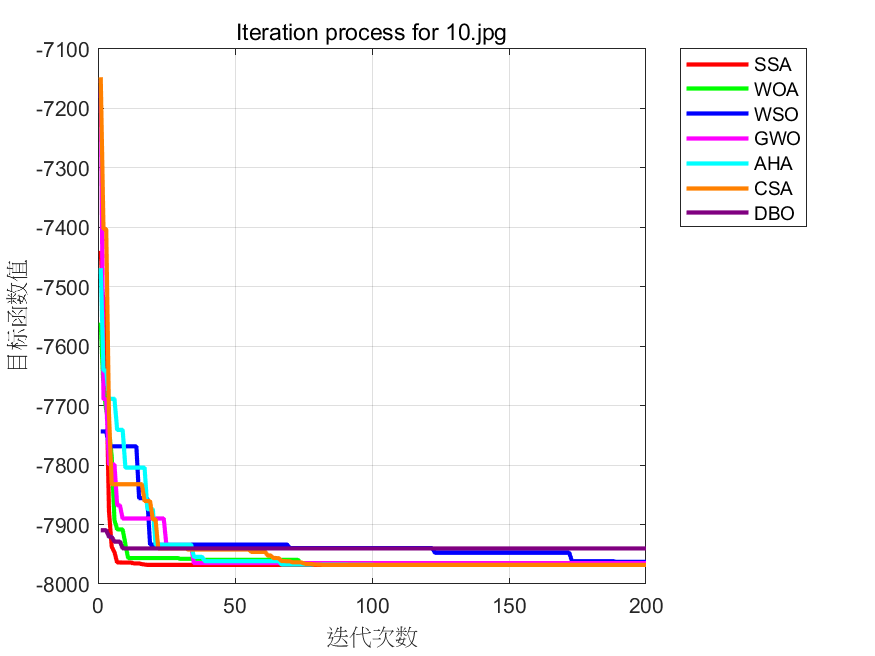

Supplement: S4 Data — (ZIP) [file pone.0297284.s004.zip › Level 2 processed Sample/iteration/10.jpg_iteration.png]

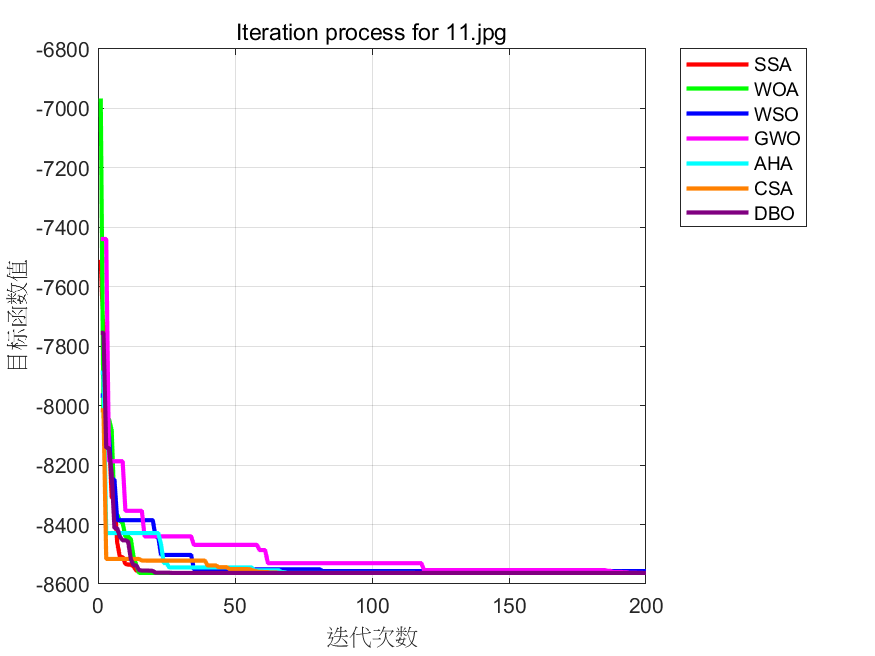

Supplement: S4 Data — (ZIP) [file pone.0297284.s004.zip › Level 2 processed Sample/iteration/11.jpg_iteration.png]

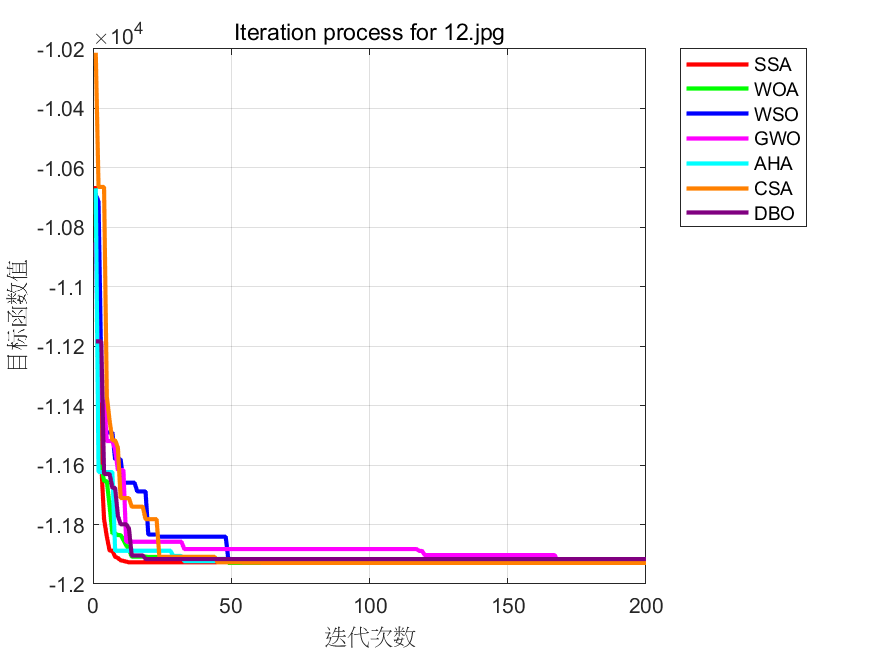

Supplement: S4 Data — (ZIP) [file pone.0297284.s004.zip › Level 2 processed Sample/iteration/12.jpg_iteration.png]

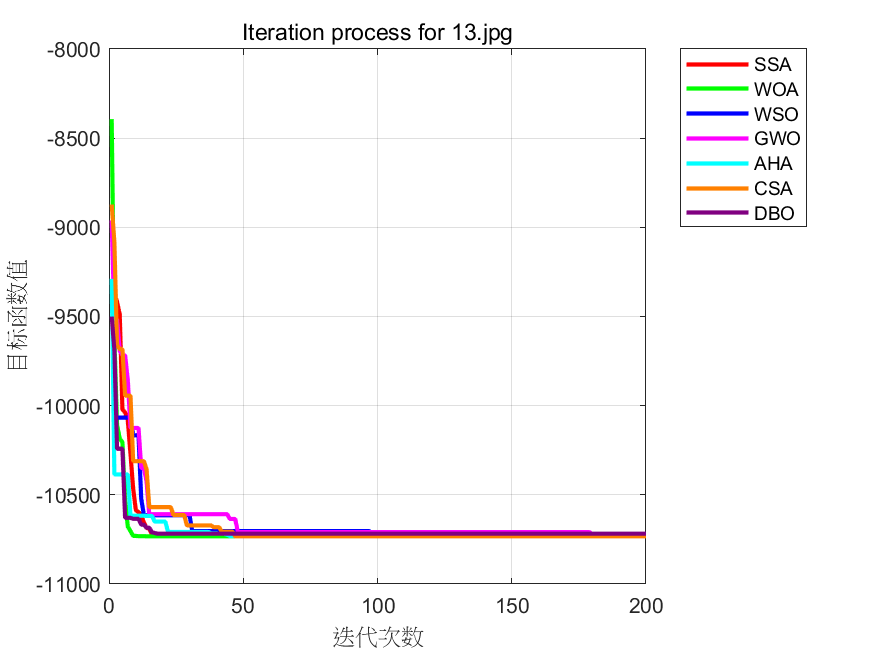

Supplement: S4 Data — (ZIP) [file pone.0297284.s004.zip › Level 2 processed Sample/iteration/13.jpg_iteration.png]

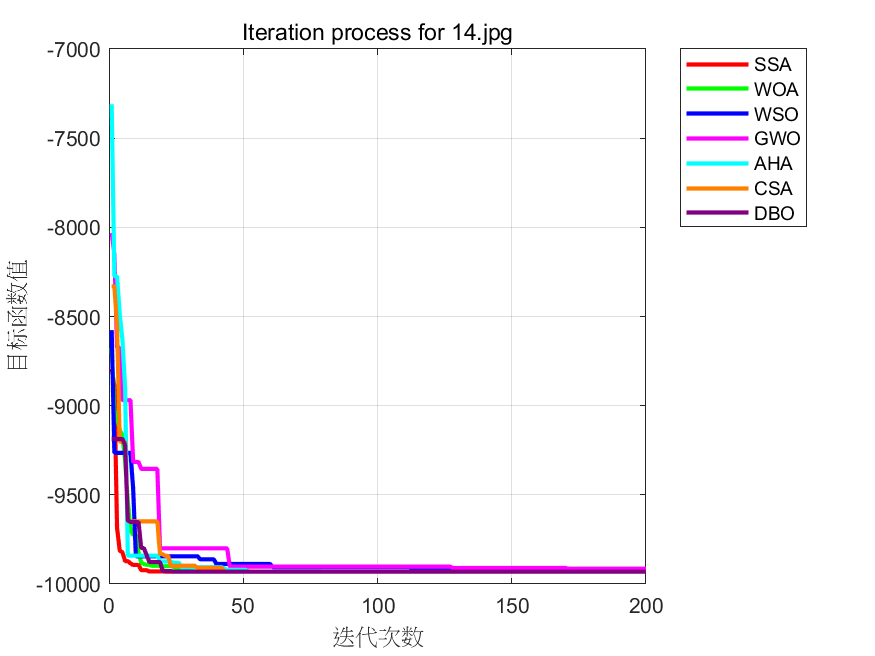

Supplement: S4 Data — (ZIP) [file pone.0297284.s004.zip › Level 2 processed Sample/iteration/14.jpg_iteration.png]

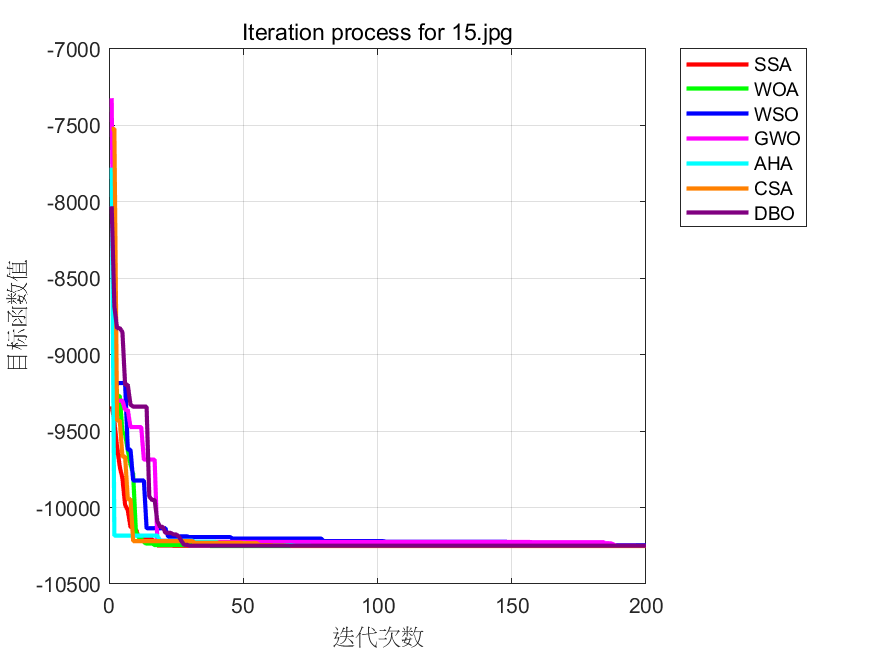

Supplement: S4 Data — (ZIP) [file pone.0297284.s004.zip › Level 2 processed Sample/iteration/15.jpg_iteration.png]

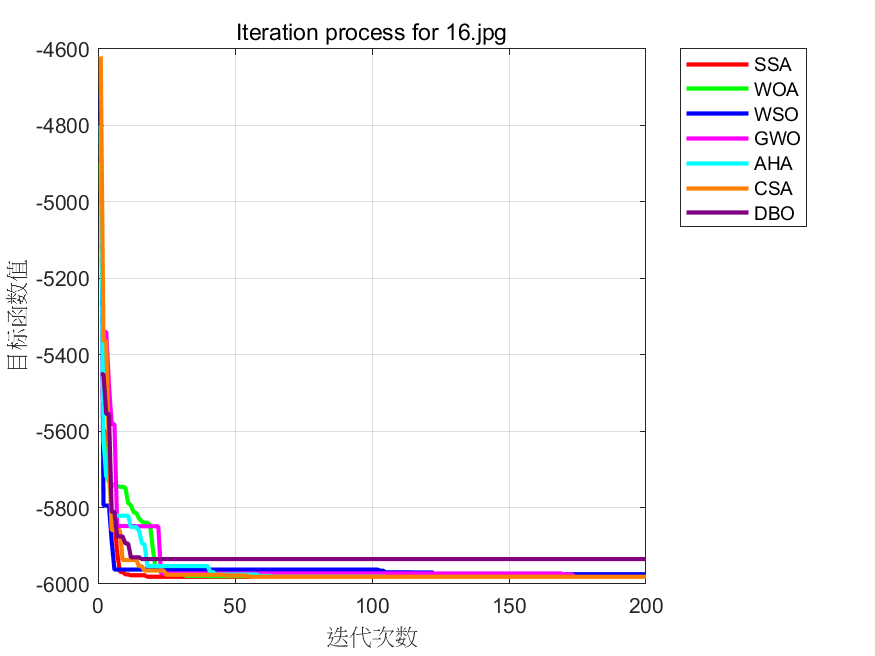

Supplement: S4 Data — (ZIP) [file pone.0297284.s004.zip › Level 2 processed Sample/iteration/16.jpg_iteration.png]

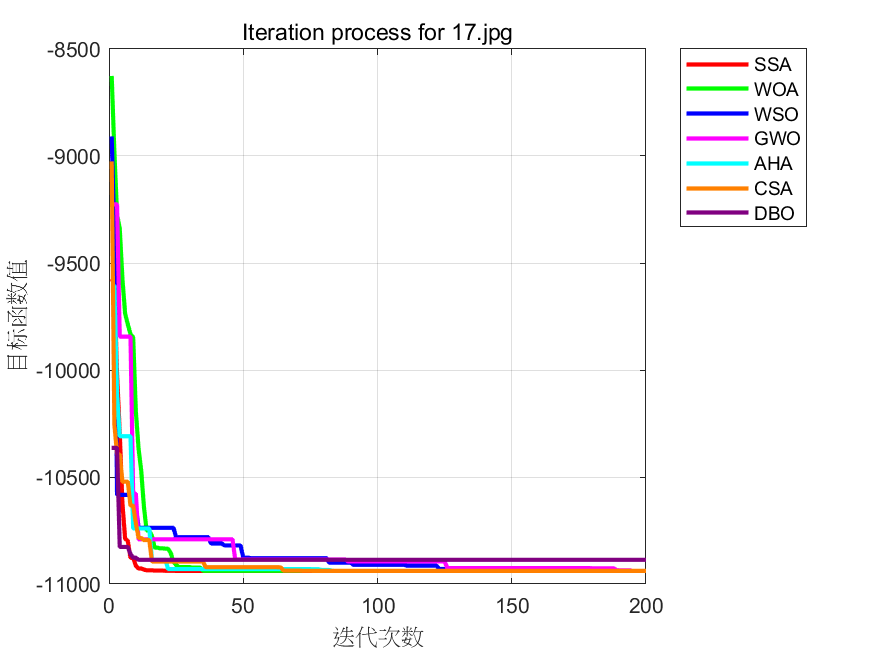

Supplement: S4 Data — (ZIP) [file pone.0297284.s004.zip › Level 2 processed Sample/iteration/17.jpg_iteration.png]

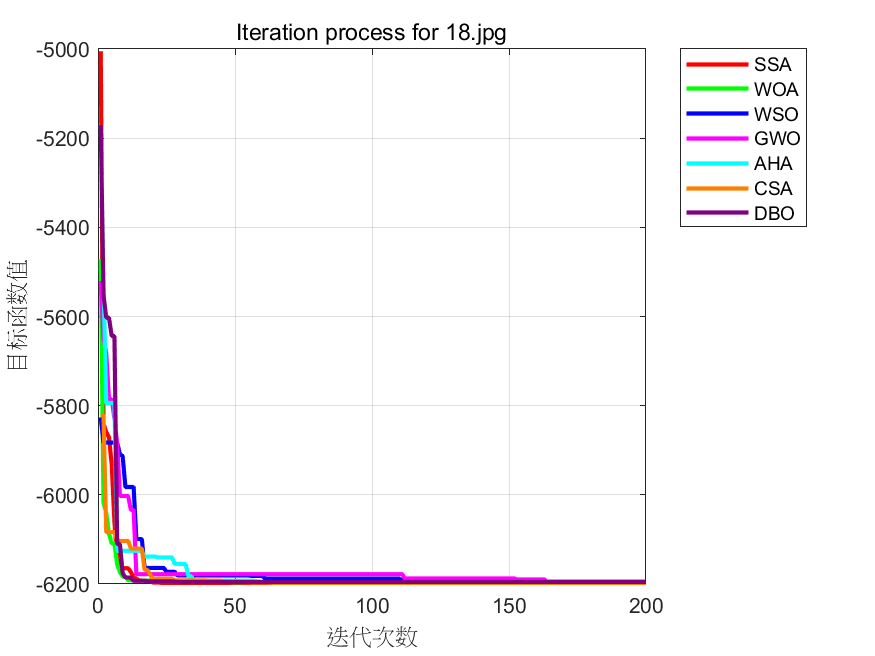

Supplement: S4 Data — (ZIP) [file pone.0297284.s004.zip › Level 2 processed Sample/iteration/18.jpg_iteration.png]

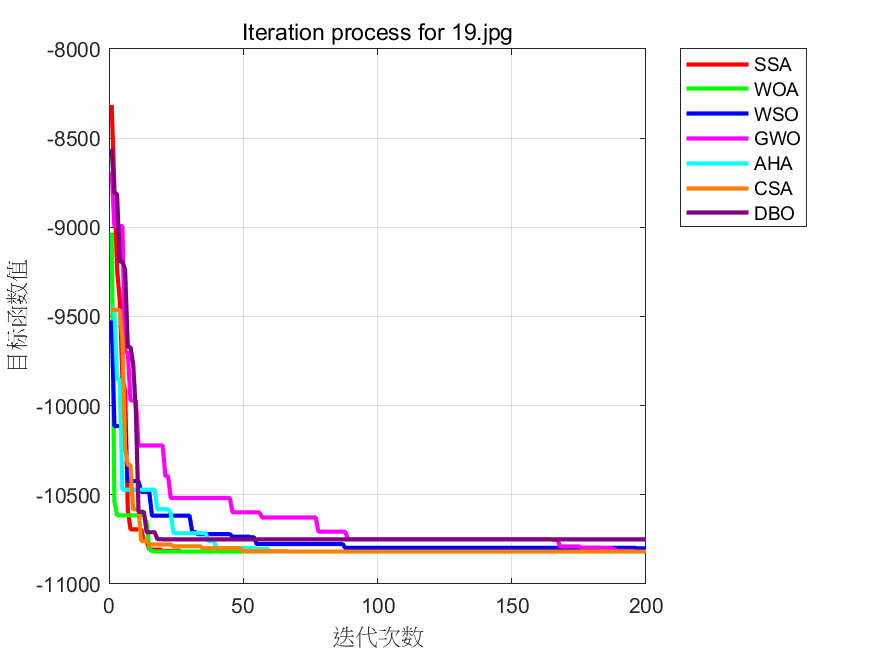

Supplement: S4 Data — (ZIP) [file pone.0297284.s004.zip › Level 2 processed Sample/iteration/19.jpg_iteration.png]

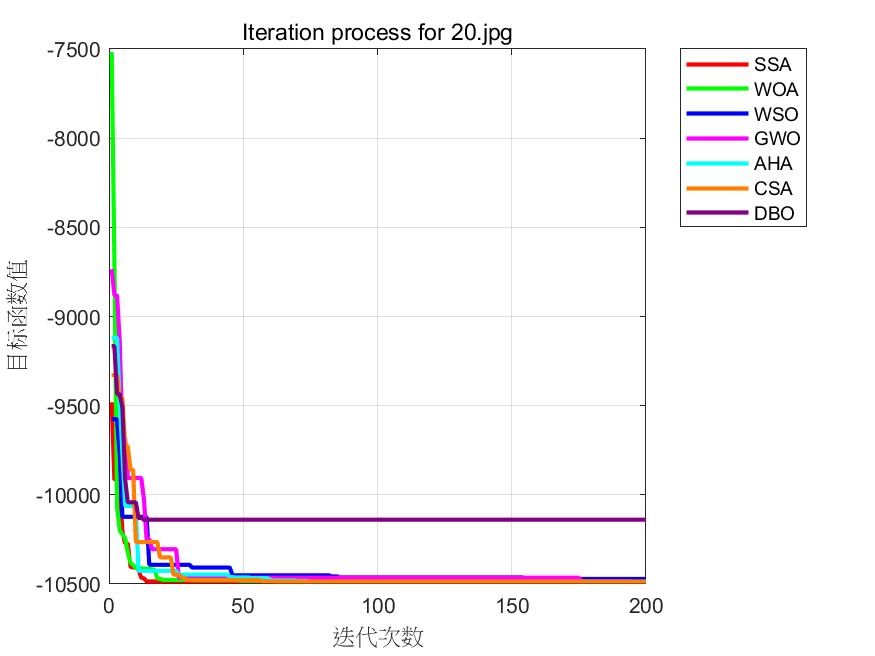

Supplement: S4 Data — (ZIP) [file pone.0297284.s004.zip › Level 2 processed Sample/iteration/20.jpg_iteration.png]

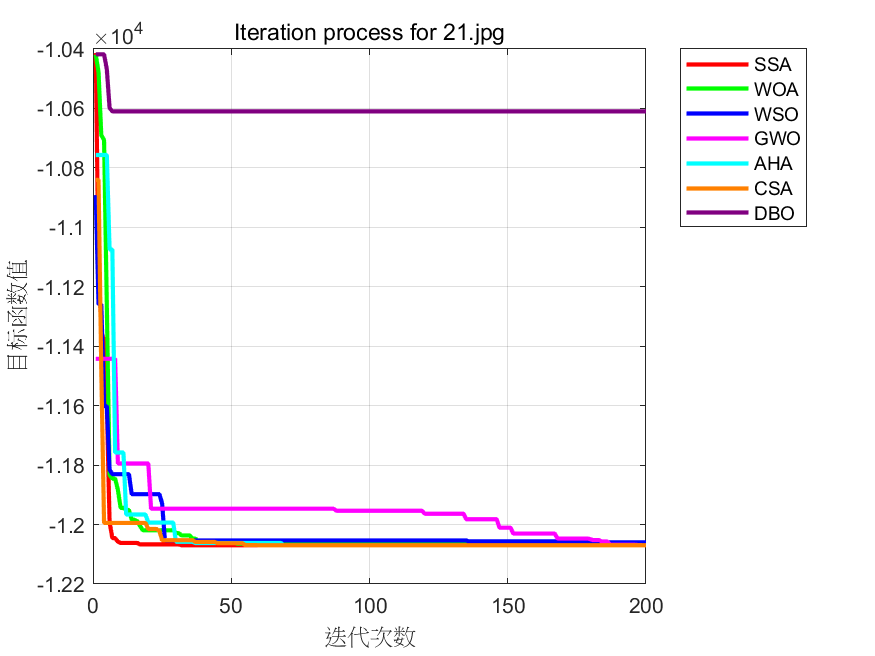

Supplement: S4 Data — (ZIP) [file pone.0297284.s004.zip › Level 2 processed Sample/iteration/21.jpg_iteration.png]

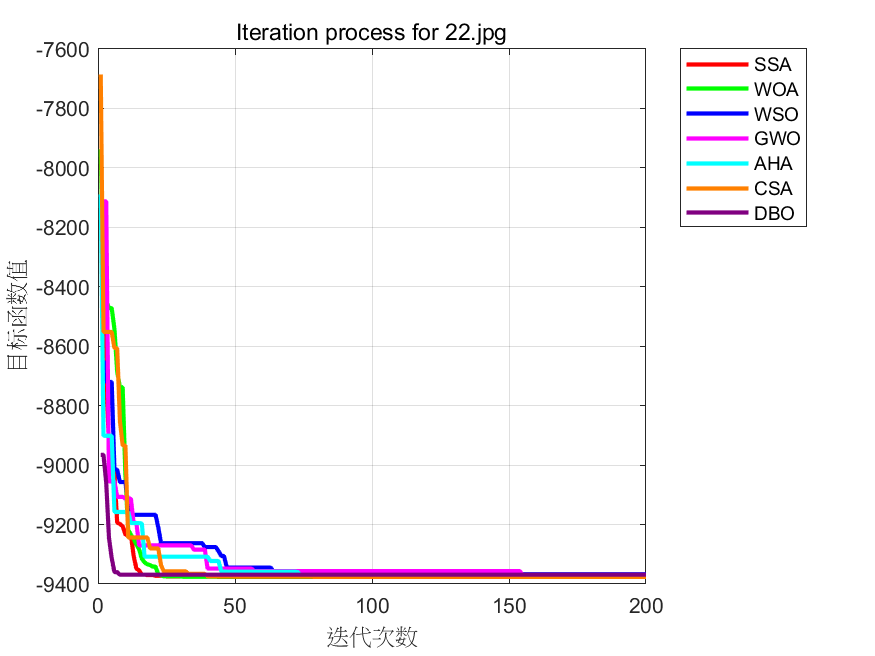

Supplement: S4 Data — (ZIP) [file pone.0297284.s004.zip › Level 2 processed Sample/iteration/22.jpg_iteration.png]

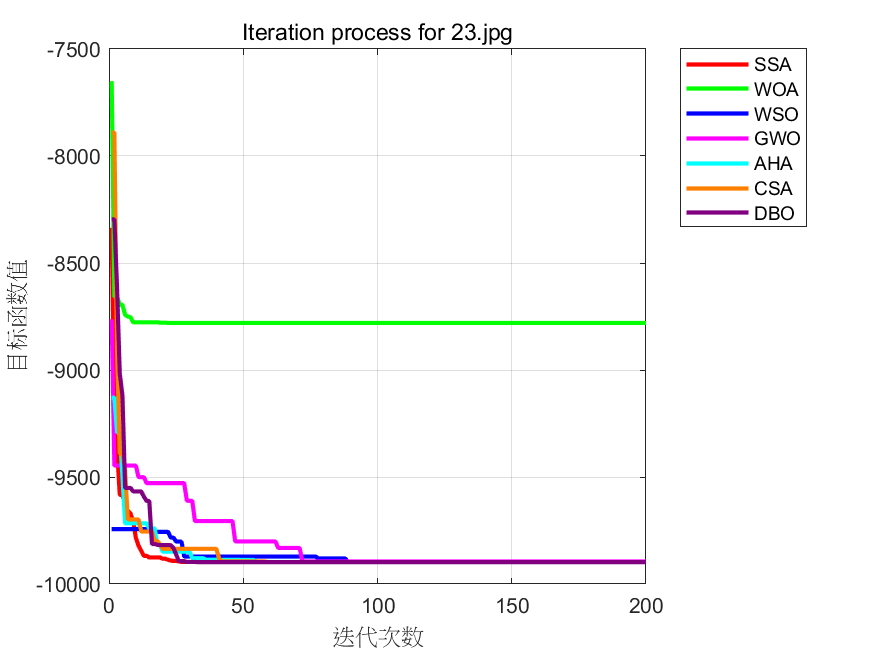

Supplement: S4 Data — (ZIP) [file pone.0297284.s004.zip › Level 2 processed Sample/iteration/23.jpg_iteration.png]

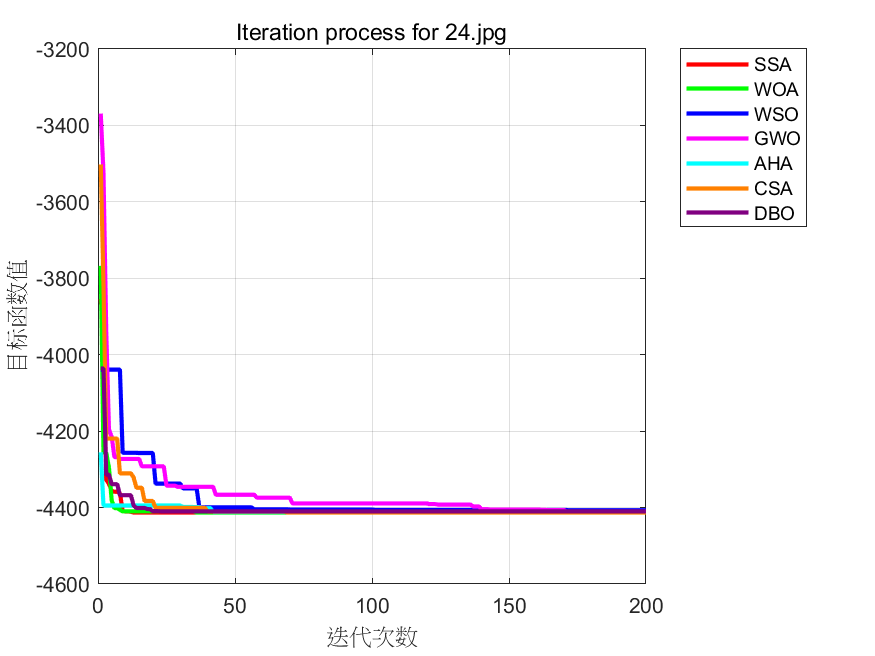

Supplement: S4 Data — (ZIP) [file pone.0297284.s004.zip › Level 2 processed Sample/iteration/24.jpg_iteration.png]

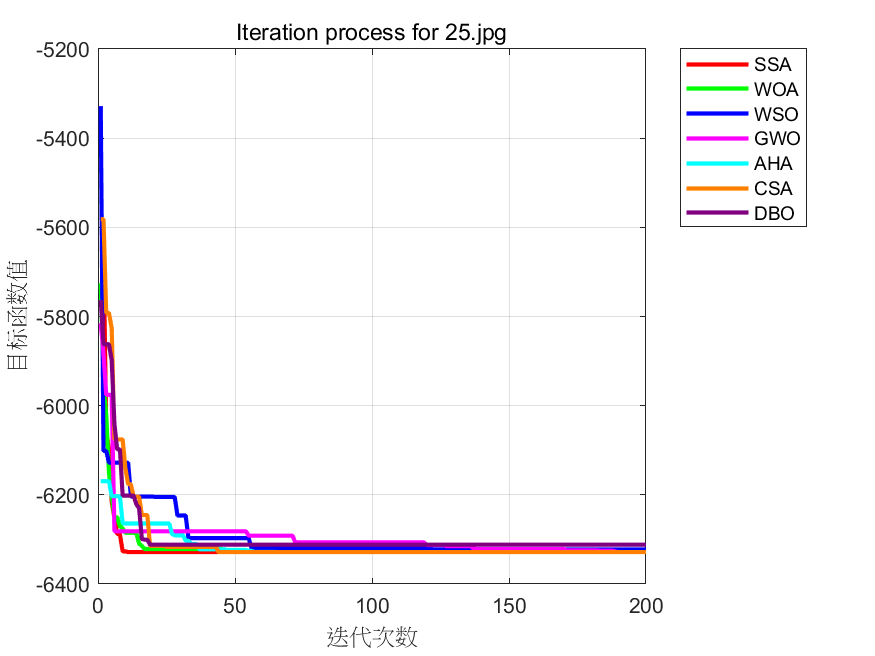

Supplement: S4 Data — (ZIP) [file pone.0297284.s004.zip › Level 2 processed Sample/iteration/25.jpg_iteration.png]

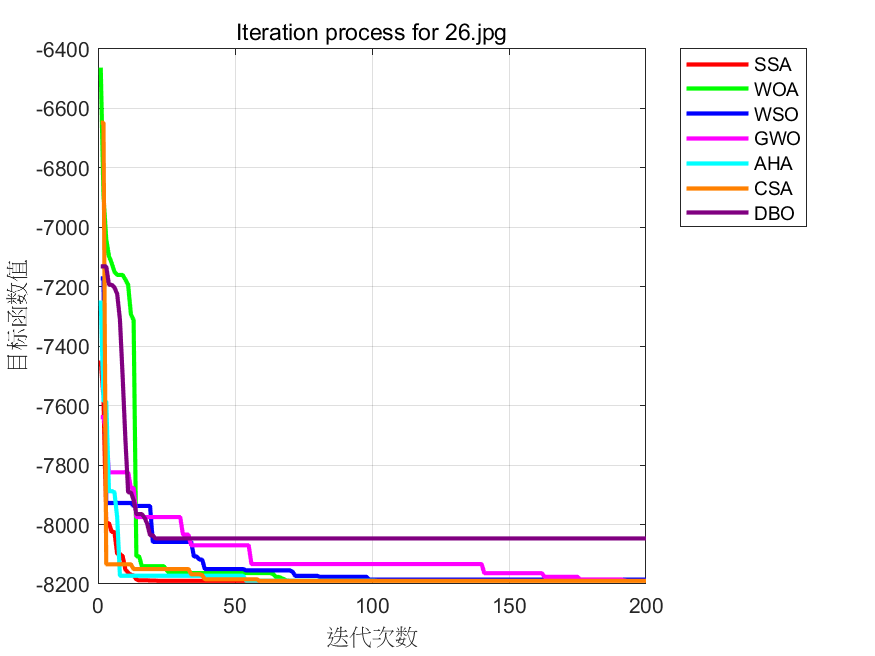

Supplement: S4 Data — (ZIP) [file pone.0297284.s004.zip › Level 2 processed Sample/iteration/26.jpg_iteration.png]

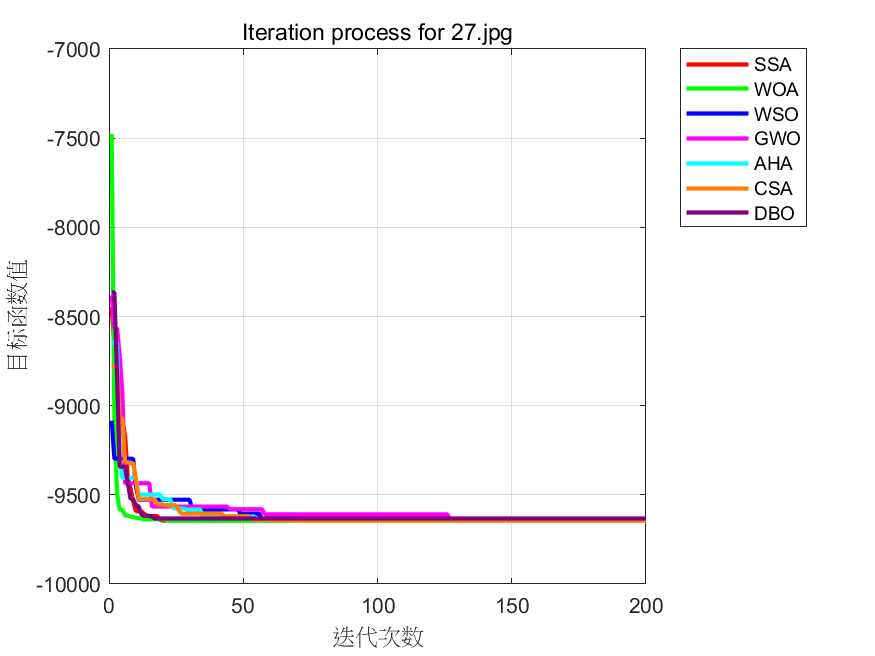

Supplement: S4 Data — (ZIP) [file pone.0297284.s004.zip › Level 2 processed Sample/iteration/27.jpg_iteration.png]

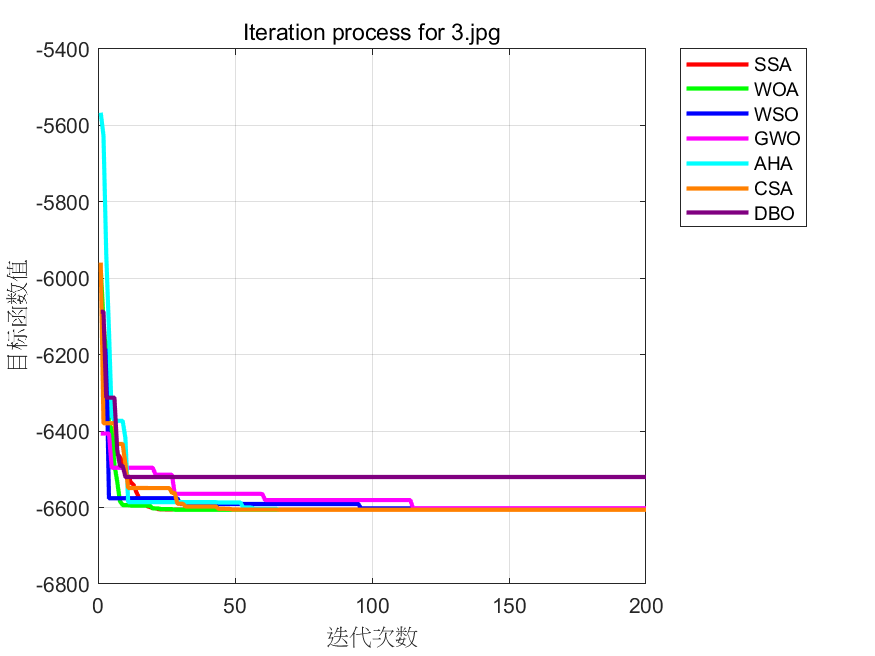

Supplement: S4 Data — (ZIP) [file pone.0297284.s004.zip › Level 2 processed Sample/iteration/3.jpg_iteration.png]

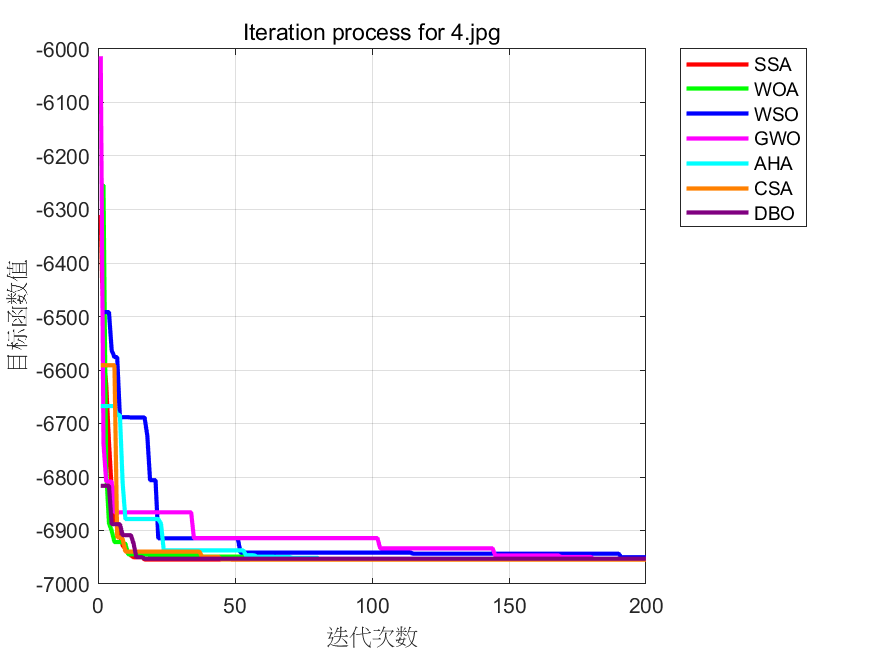

Supplement: S4 Data — (ZIP) [file pone.0297284.s004.zip › Level 2 processed Sample/iteration/4.jpg_iteration.png]

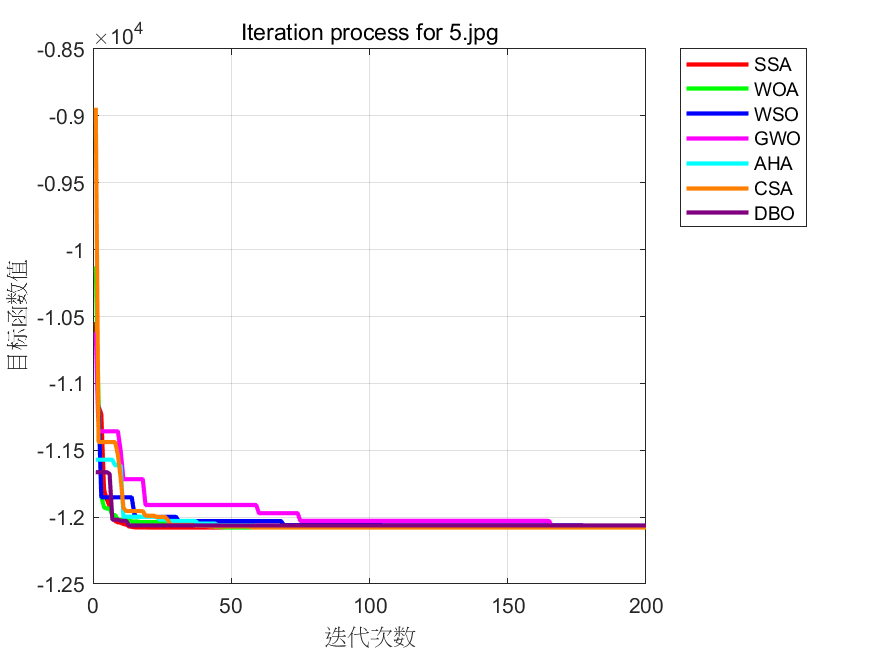

Supplement: S4 Data — (ZIP) [file pone.0297284.s004.zip › Level 2 processed Sample/iteration/5.jpg_iteration.png]

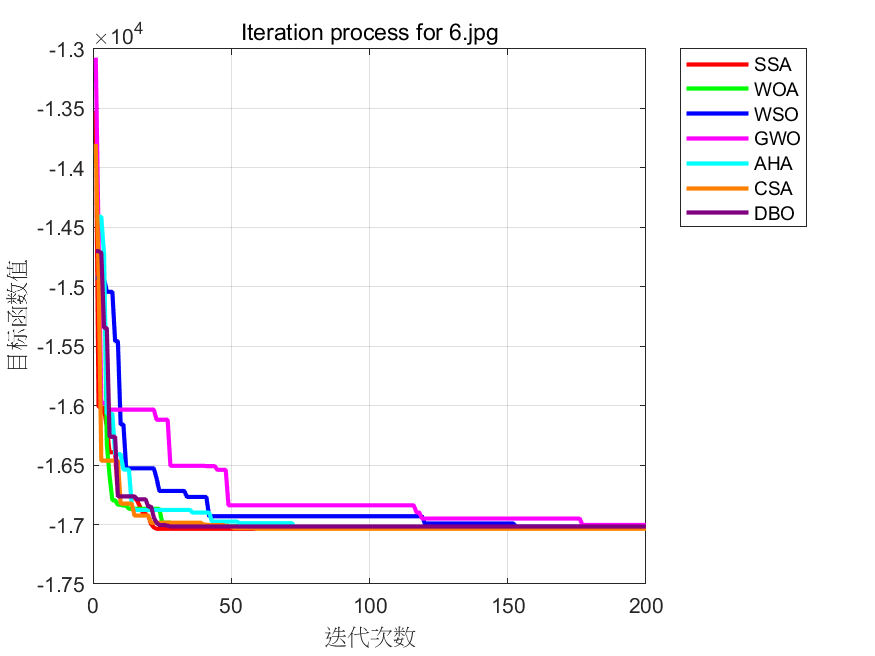

Supplement: S4 Data — (ZIP) [file pone.0297284.s004.zip › Level 2 processed Sample/iteration/6.jpg_iteration.png]

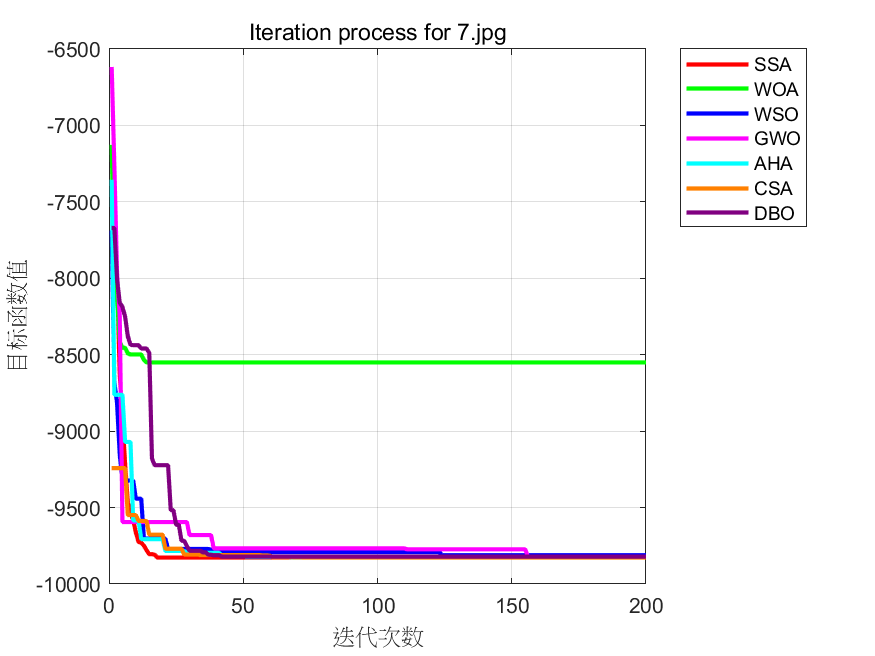

Supplement: S4 Data — (ZIP) [file pone.0297284.s004.zip › Level 2 processed Sample/iteration/7.jpg_iteration.png]

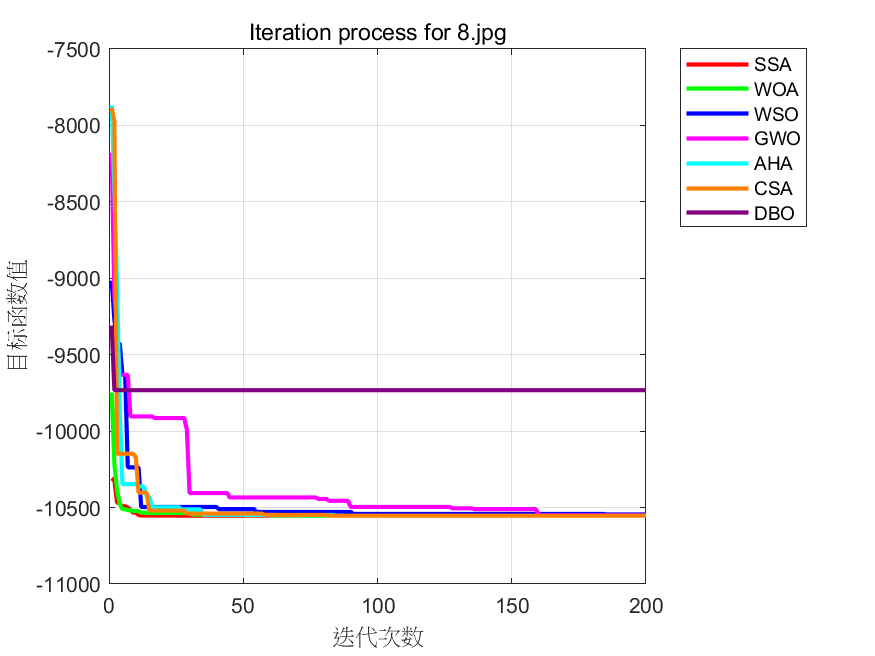

Supplement: S4 Data — (ZIP) [file pone.0297284.s004.zip › Level 2 processed Sample/iteration/8.jpg_iteration.png]

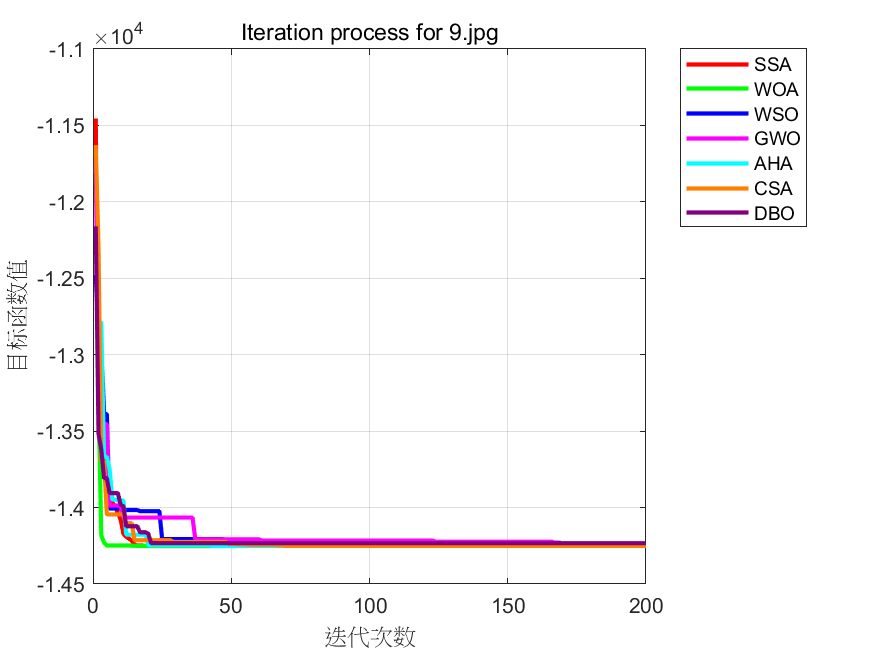

Supplement: S4 Data — (ZIP) [file pone.0297284.s004.zip › Level 2 processed Sample/iteration/9.jpg_iteration.png]

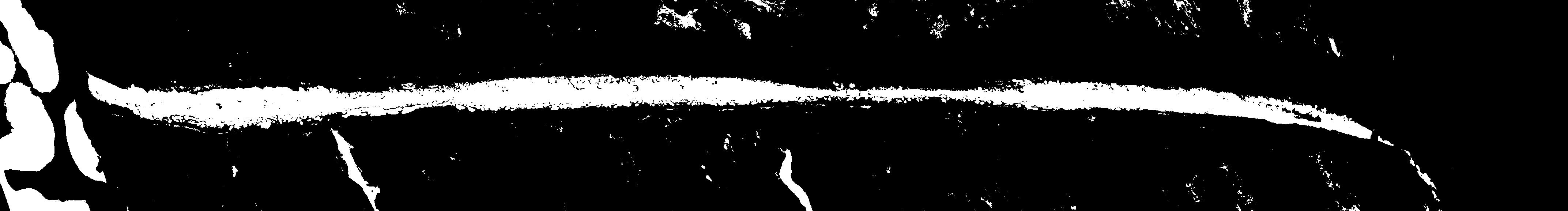

Supplement: S4 Data — (ZIP) [file pone.0297284.s004.zip › Level 2 processed Sample/processed_10/latex/AHA_latex.jpg]

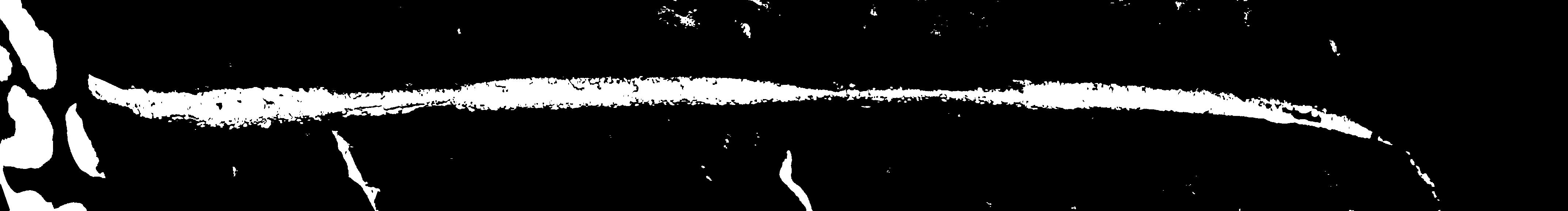

Supplement: S4 Data — (ZIP) [file pone.0297284.s004.zip › Level 2 processed Sample/processed_10/latex/DBO_latex.jpg]

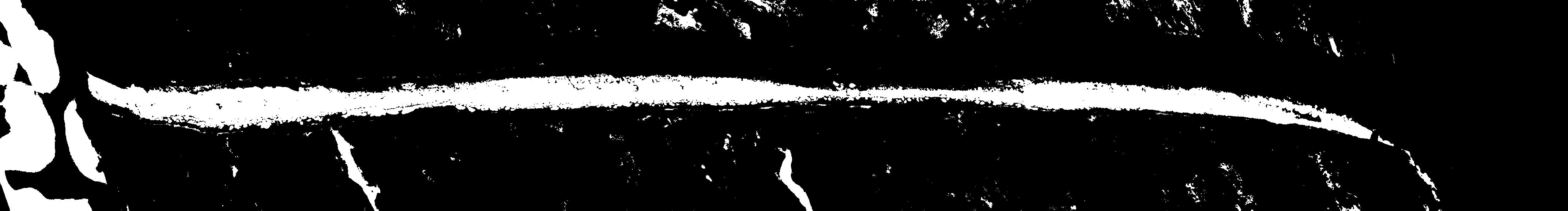

Supplement: S4 Data — (ZIP) [file pone.0297284.s004.zip › Level 2 processed Sample/processed_10/latex/GWO_latex.jpg]

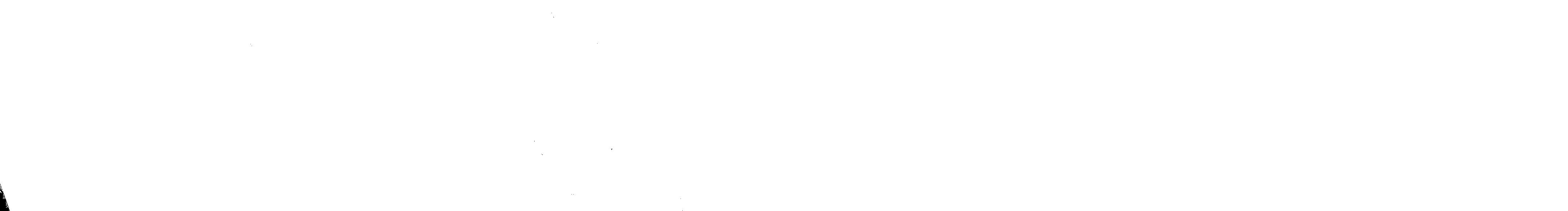

Supplement: S4 Data — (ZIP) [file pone.0297284.s004.zip › Level 2 processed Sample/processed_10/latex/OTSU_latex.jpg]

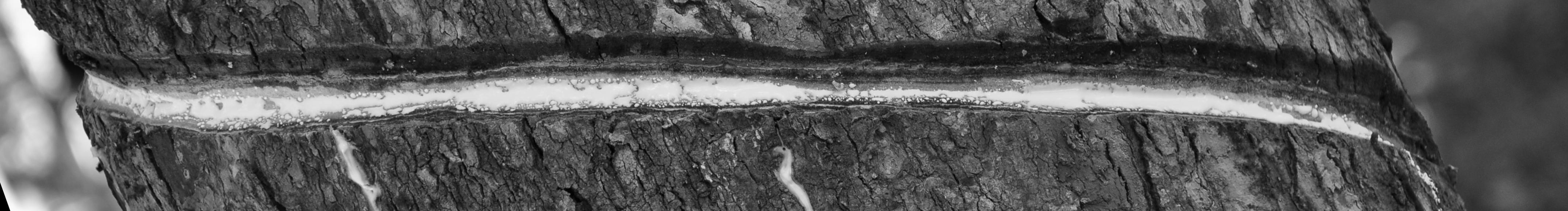

Supplement: S4 Data — (ZIP) [file pone.0297284.s004.zip › Level 2 processed Sample/processed_10/original_image.jpg]

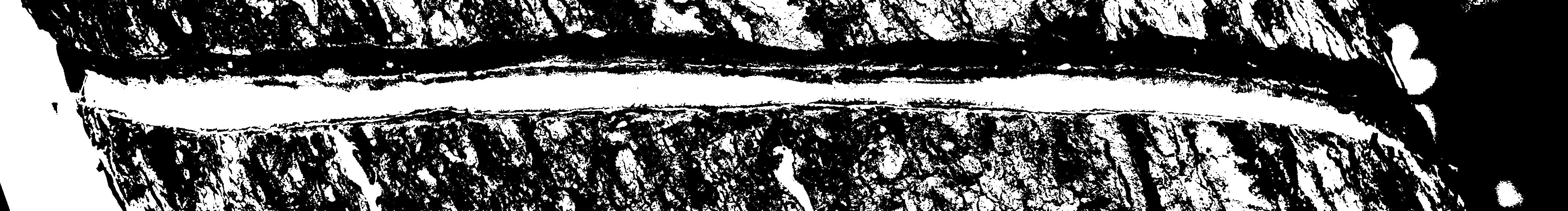

Supplement: S4 Data — (ZIP) [file pone.0297284.s004.zip › Level 2 processed Sample/processed_10/scar/AHA_scar.jpg]

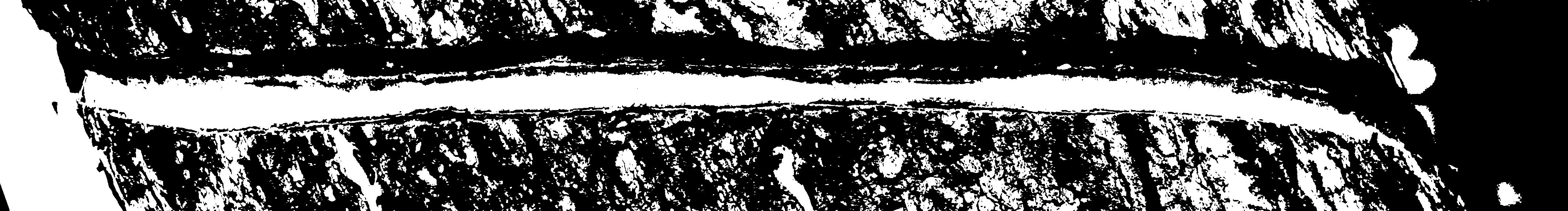

Supplement: S4 Data — (ZIP) [file pone.0297284.s004.zip › Level 2 processed Sample/processed_10/scar/DBO_scar.jpg]

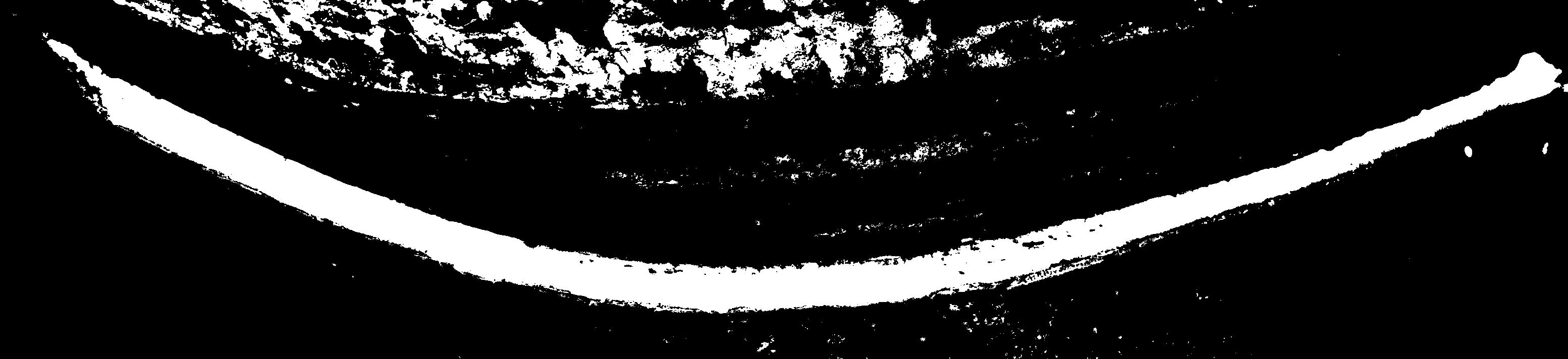

Supplement: S4 Data — (ZIP) [file pone.0297284.s004.zip › Level 2 processed Sample/processed_11/latex/AHA_latex.jpg]

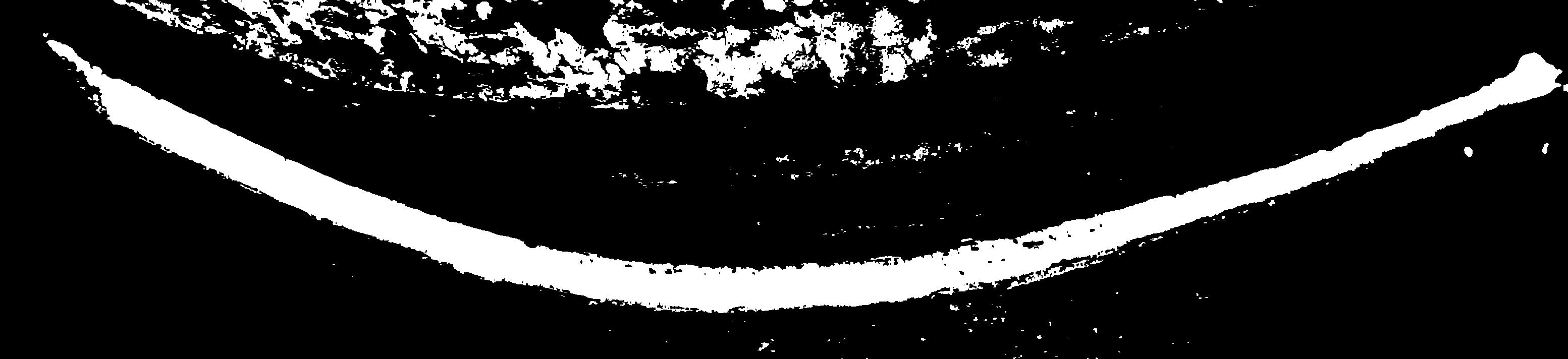

Supplement: S4 Data — (ZIP) [file pone.0297284.s004.zip › Level 2 processed Sample/processed_11/latex/DBO_latex.jpg]

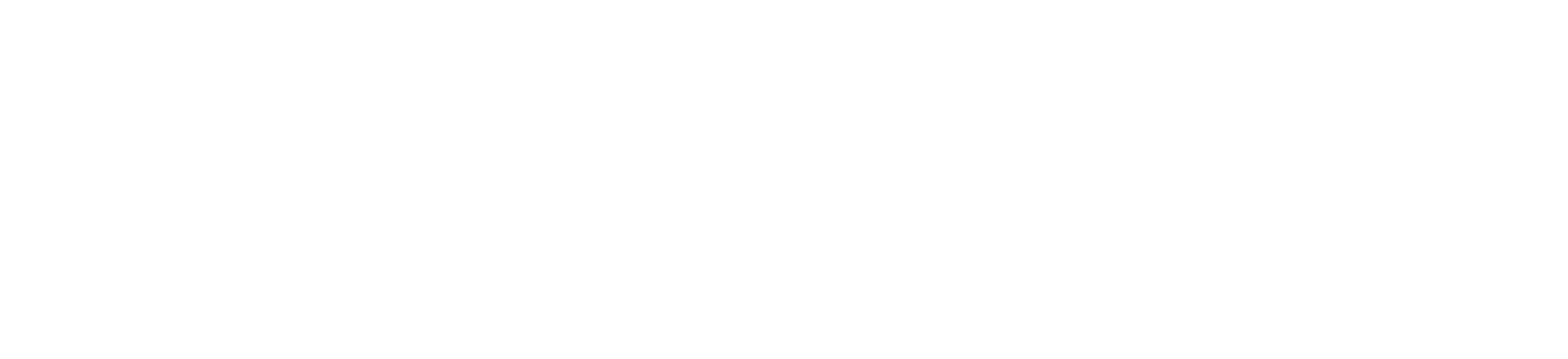

Supplement: S4 Data — (ZIP) [file pone.0297284.s004.zip › Level 2 processed Sample/processed_11/latex/OTSU_latex.jpg]

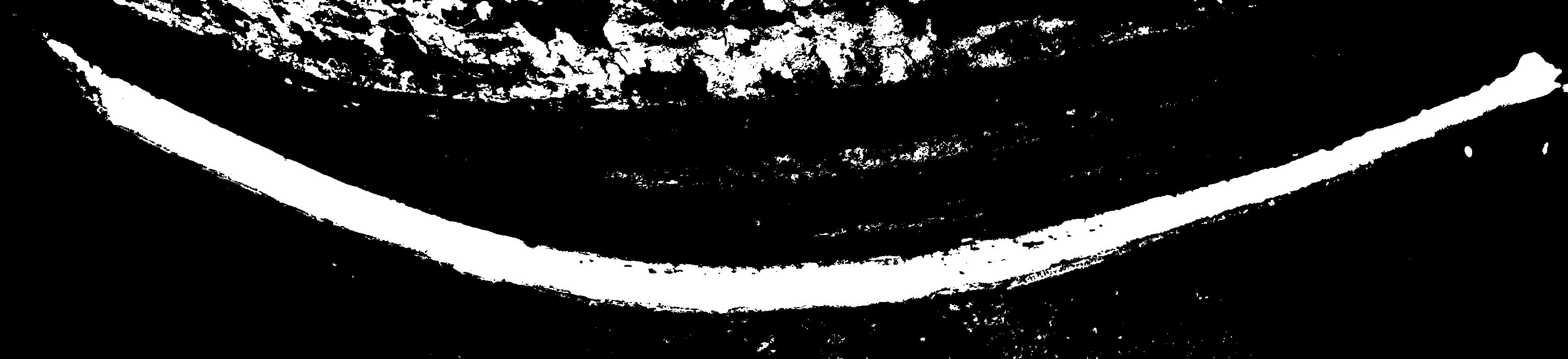

Supplement: S4 Data — (ZIP) [file pone.0297284.s004.zip › Level 2 processed Sample/processed_11/latex/WSO_latex.jpg]

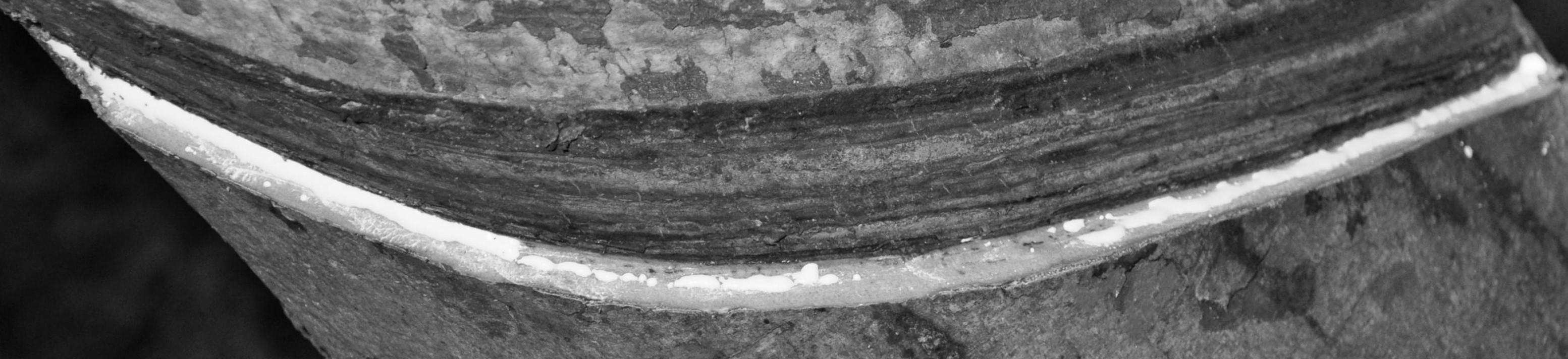

Supplement: S4 Data — (ZIP) [file pone.0297284.s004.zip › Level 2 processed Sample/processed_11/original_image.jpg]
