# Supplementary figures and images for: Advancing image segmentation with DBO-Otsu: Addressing rubber tree diseases through enhanced threshold techniques (part 3 of 7)
Source: PLoS One. 2024 Mar 21;19(3):e0297284. doi: 10.1371/journal.pone.0297284 (PMC10956860; doi:10.1371/journal.pone.0297284)

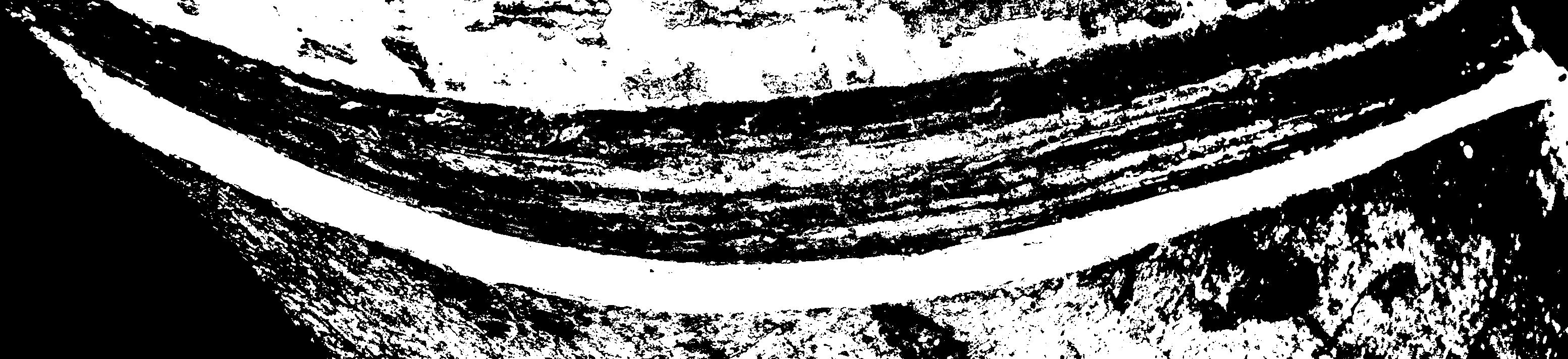

Supplement: S4 Data — (ZIP) [file pone.0297284.s004.zip › Level 2 processed Sample/processed_11/scar/AHA_scar.jpg]

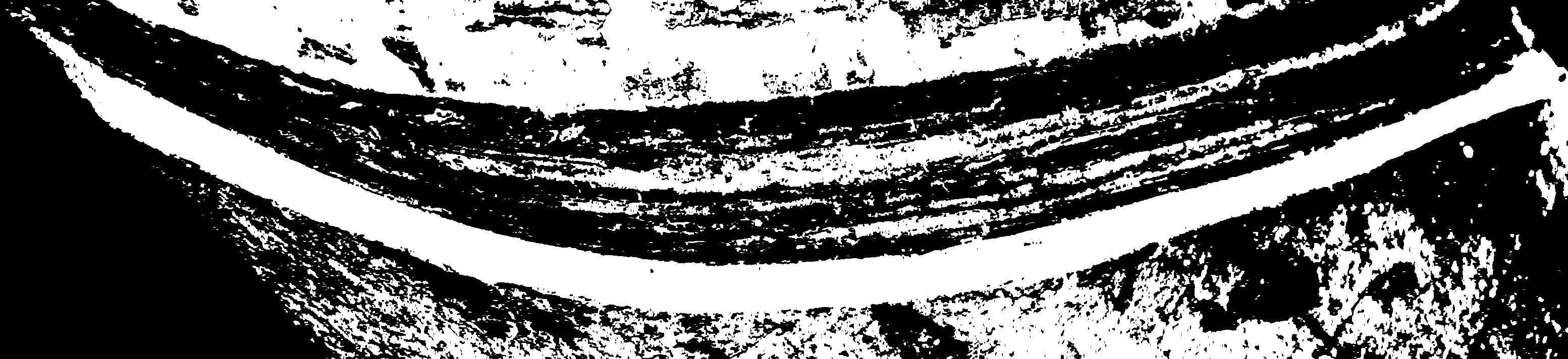

Supplement: S4 Data — (ZIP) [file pone.0297284.s004.zip › Level 2 processed Sample/processed_11/scar/DBO_scar.jpg]

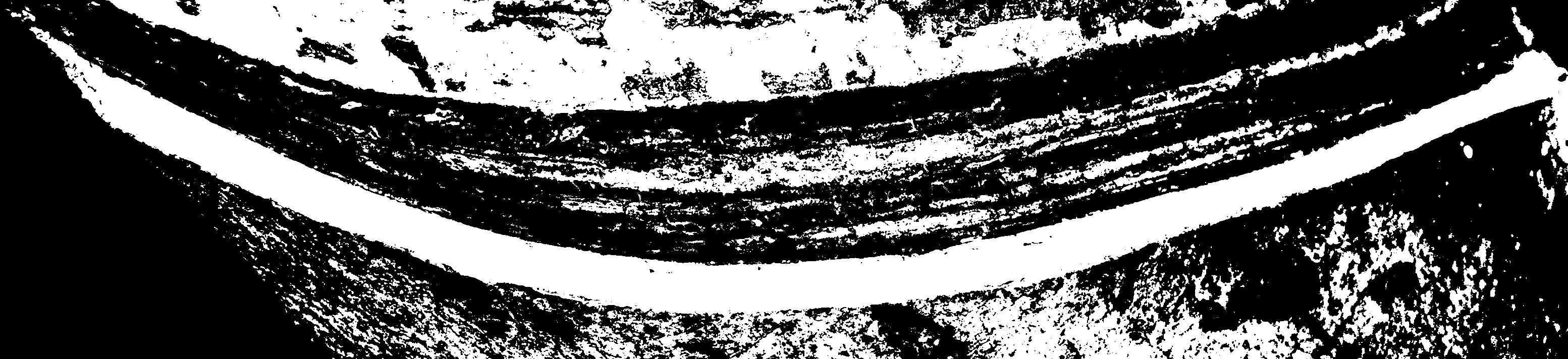

Supplement: S4 Data — (ZIP) [file pone.0297284.s004.zip › Level 2 processed Sample/processed_11/scar/WSO_scar.jpg]

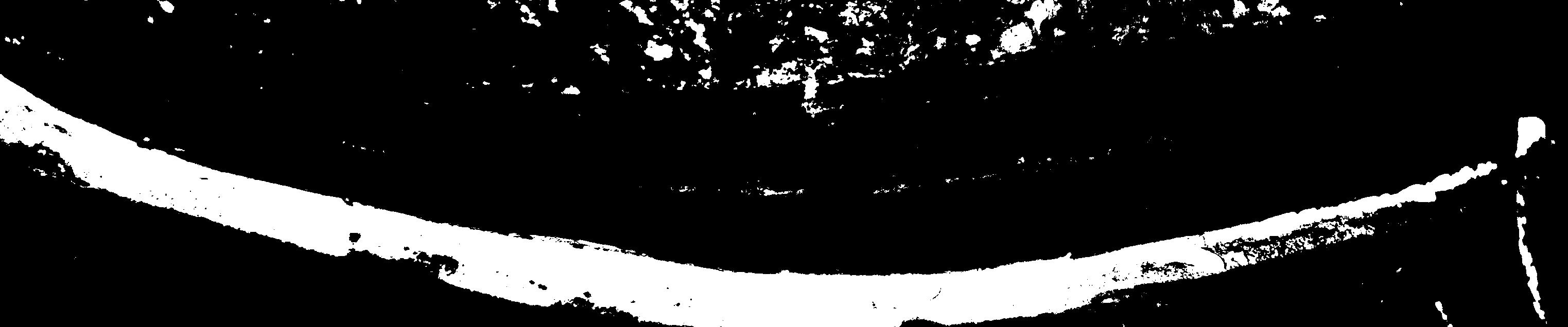

Supplement: S4 Data — (ZIP) [file pone.0297284.s004.zip › Level 2 processed Sample/processed_12/latex/AHA_latex.jpg]

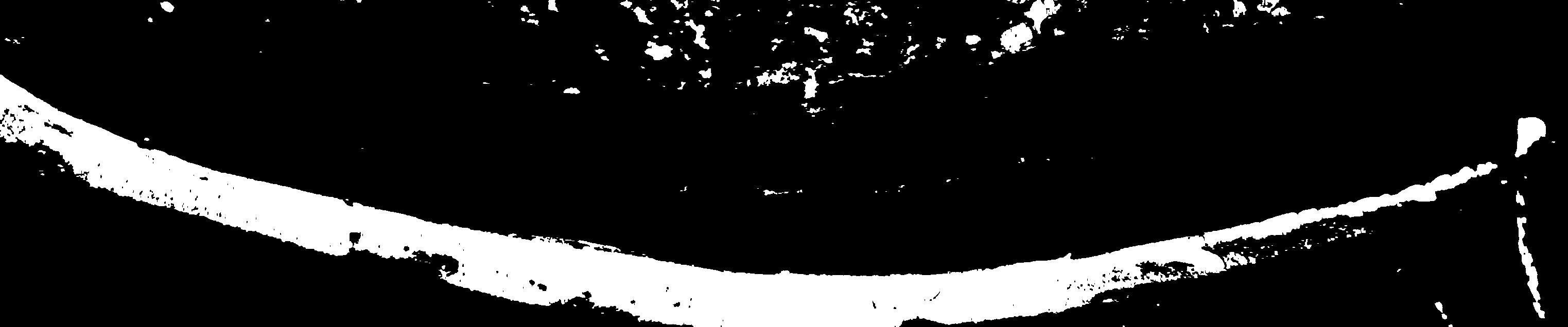

Supplement: S4 Data — (ZIP) [file pone.0297284.s004.zip › Level 2 processed Sample/processed_12/latex/DBO_latex.jpg]

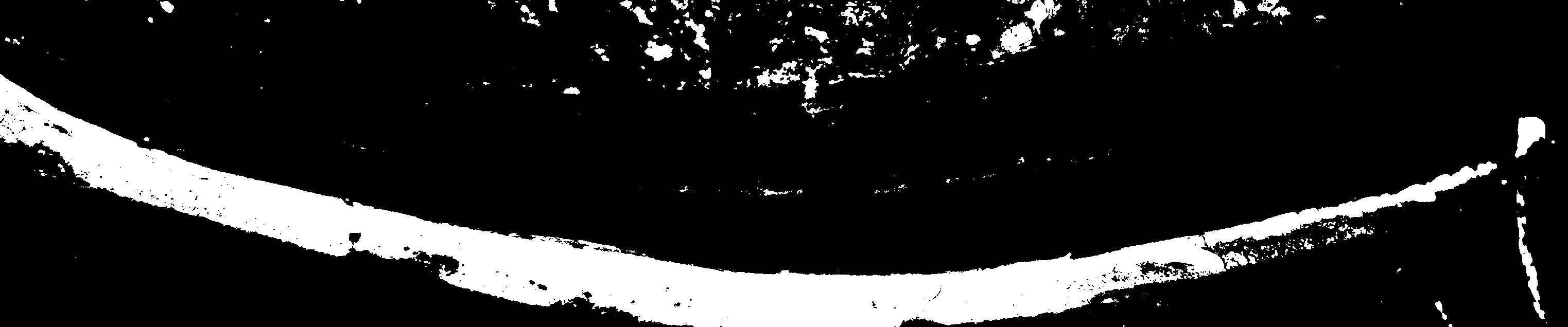

Supplement: S4 Data — (ZIP) [file pone.0297284.s004.zip › Level 2 processed Sample/processed_12/latex/GWO_latex.jpg]

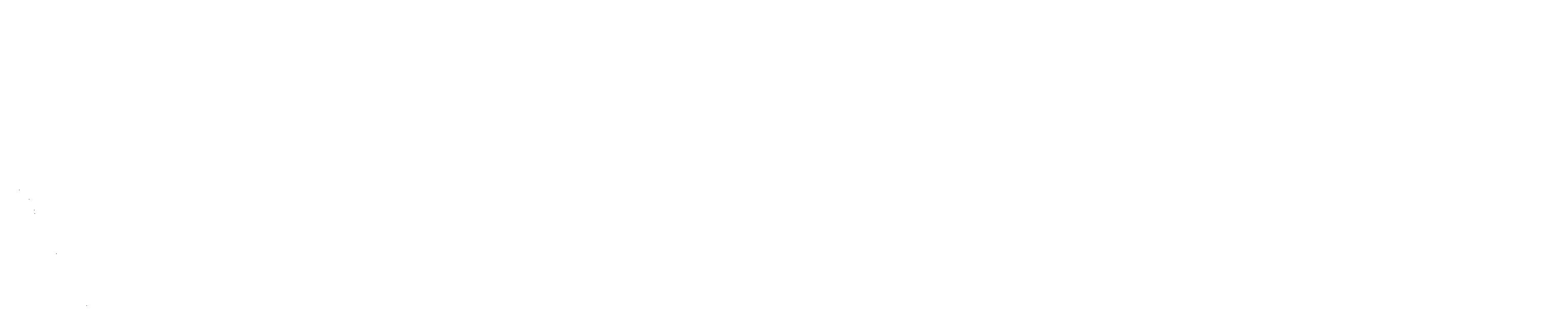

Supplement: S4 Data — (ZIP) [file pone.0297284.s004.zip › Level 2 processed Sample/processed_12/latex/OTSU_latex.jpg]

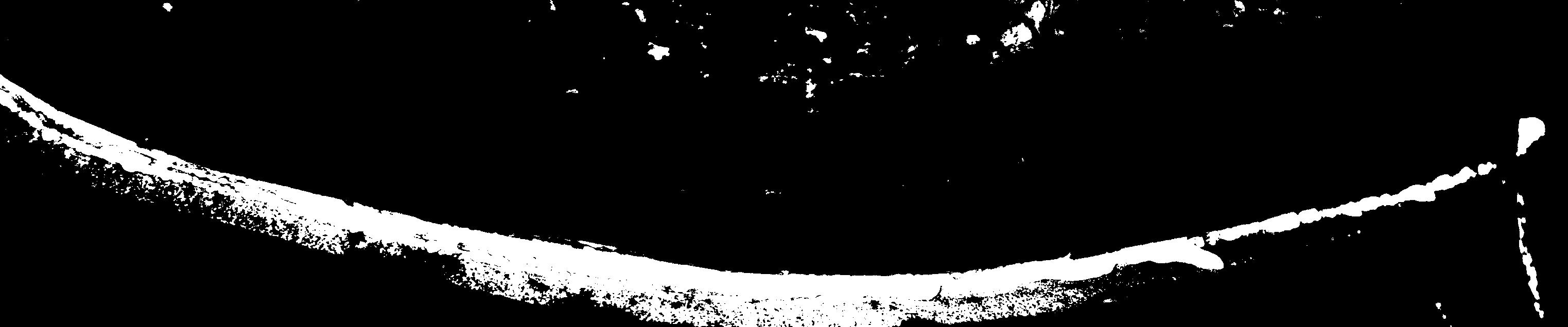

Supplement: S4 Data — (ZIP) [file pone.0297284.s004.zip › Level 2 processed Sample/processed_12/latex/WSO_latex.jpg]

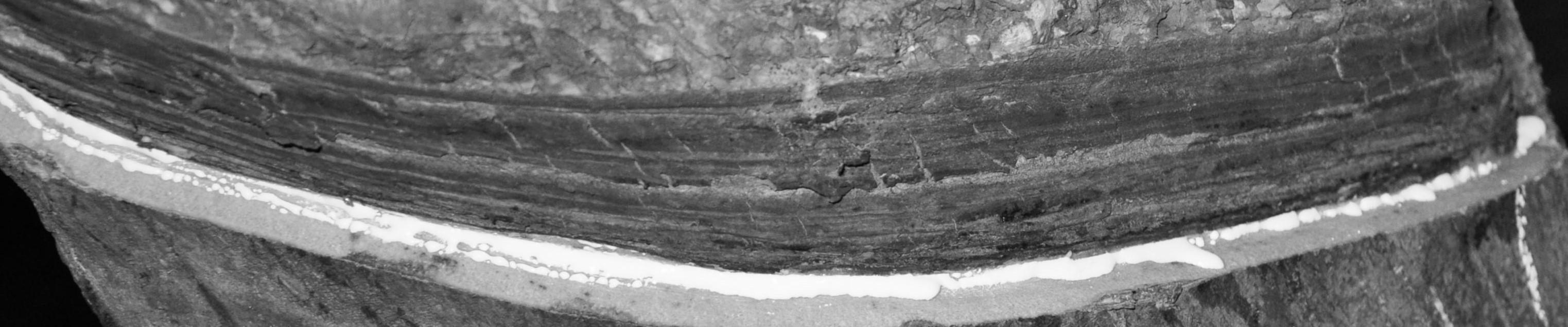

Supplement: S4 Data — (ZIP) [file pone.0297284.s004.zip › Level 2 processed Sample/processed_12/original_image.jpg]

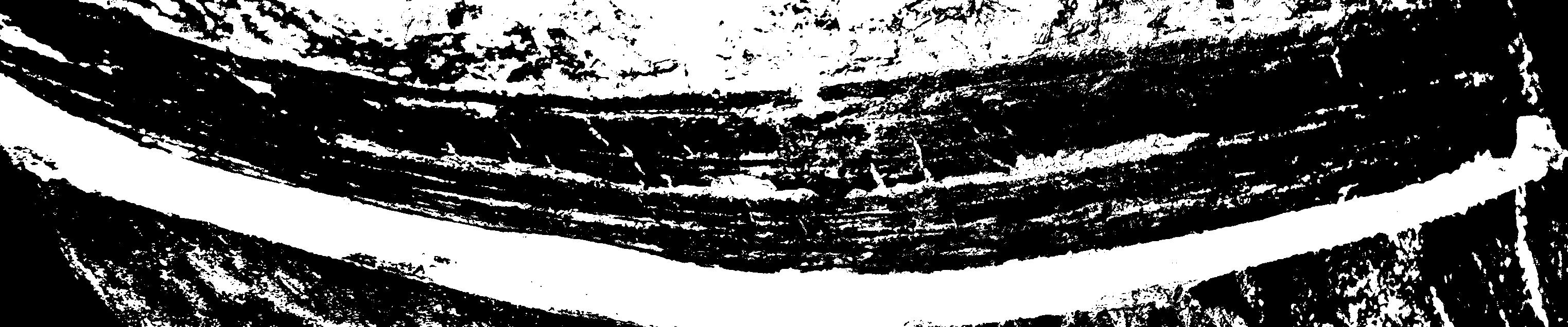

Supplement: S4 Data — (ZIP) [file pone.0297284.s004.zip › Level 2 processed Sample/processed_12/scar/AHA_scar.jpg]

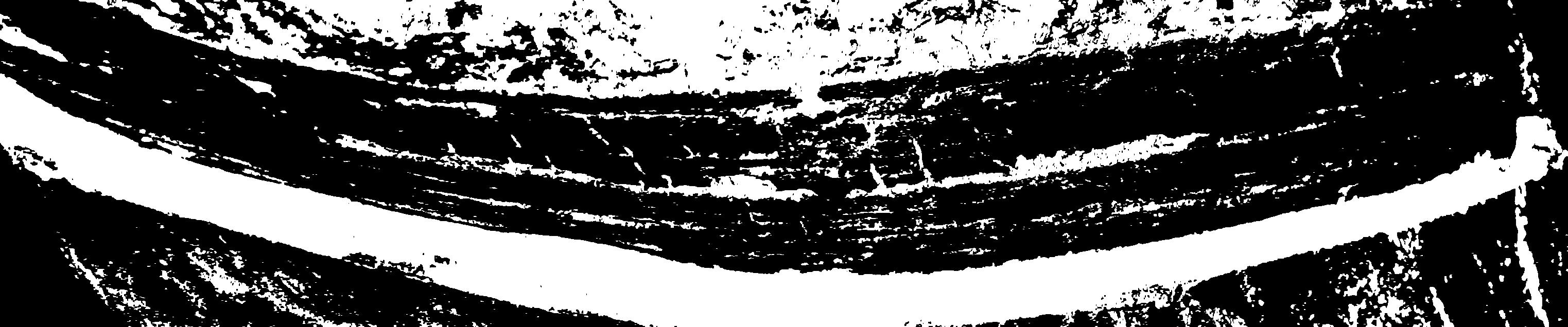

Supplement: S4 Data — (ZIP) [file pone.0297284.s004.zip › Level 2 processed Sample/processed_12/scar/DBO_scar.jpg]

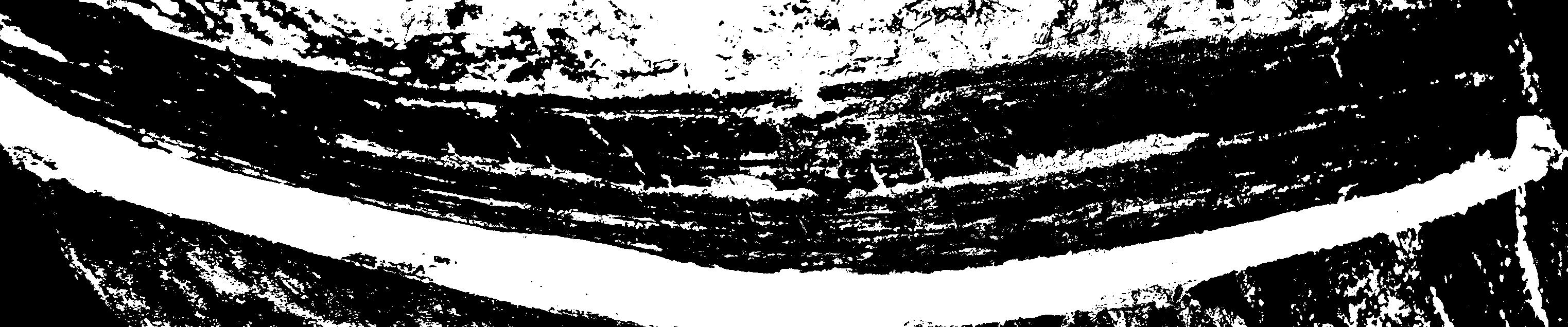

Supplement: S4 Data — (ZIP) [file pone.0297284.s004.zip › Level 2 processed Sample/processed_12/scar/GWO_scar.jpg]

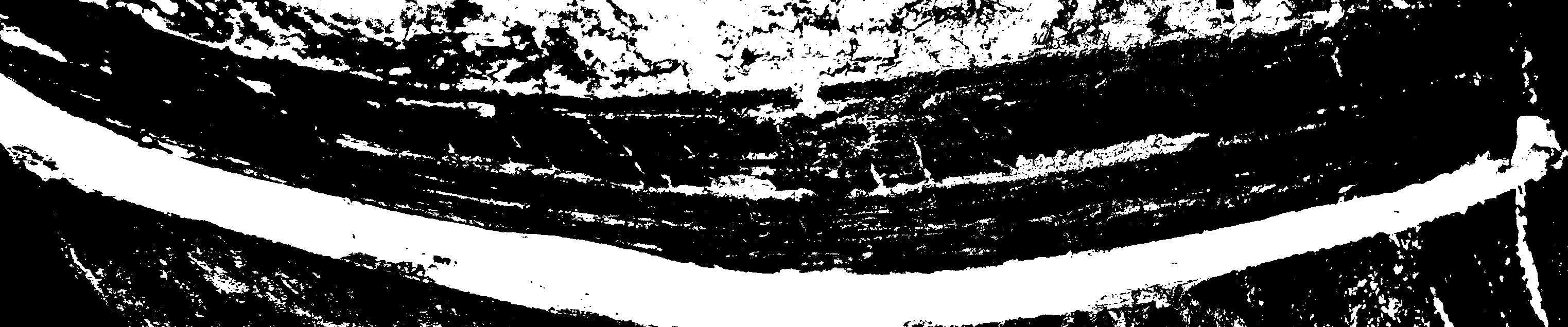

Supplement: S4 Data — (ZIP) [file pone.0297284.s004.zip › Level 2 processed Sample/processed_12/scar/WSO_scar.jpg]

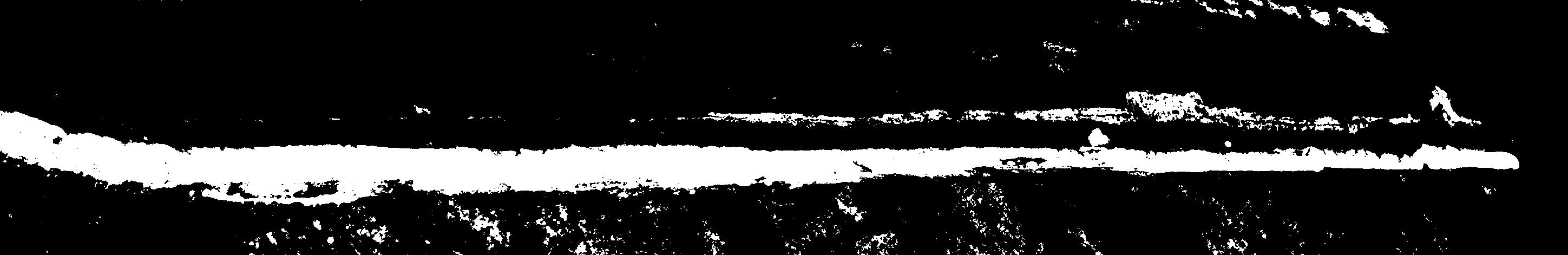

Supplement: S4 Data — (ZIP) [file pone.0297284.s004.zip › Level 2 processed Sample/processed_13/latex/AHA_latex.jpg]

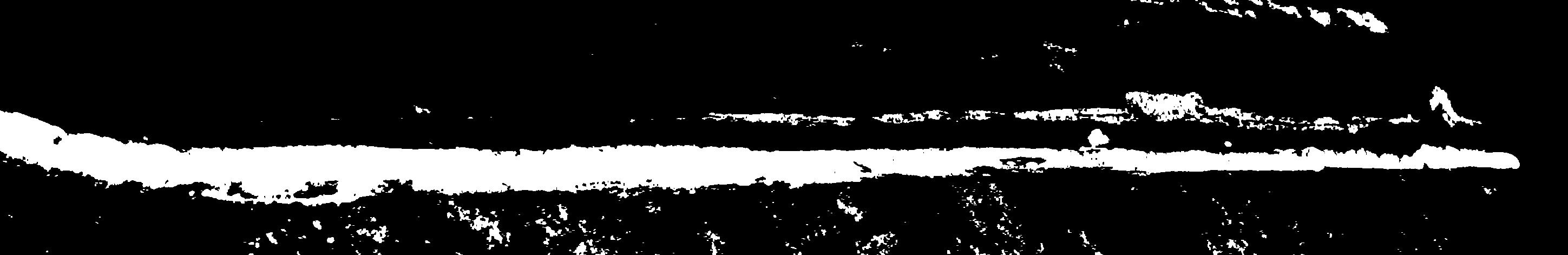

Supplement: S4 Data — (ZIP) [file pone.0297284.s004.zip › Level 2 processed Sample/processed_13/latex/DBO_latex.jpg]

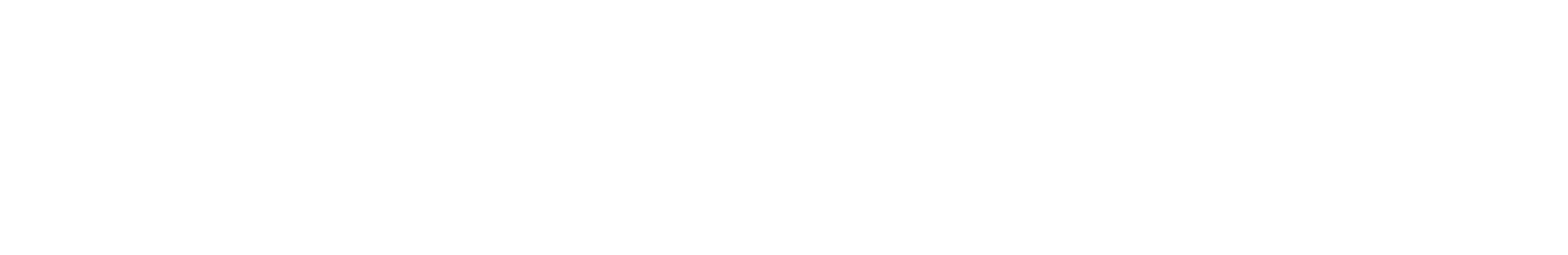

Supplement: S4 Data — (ZIP) [file pone.0297284.s004.zip › Level 2 processed Sample/processed_13/latex/OTSU_latex.jpg]

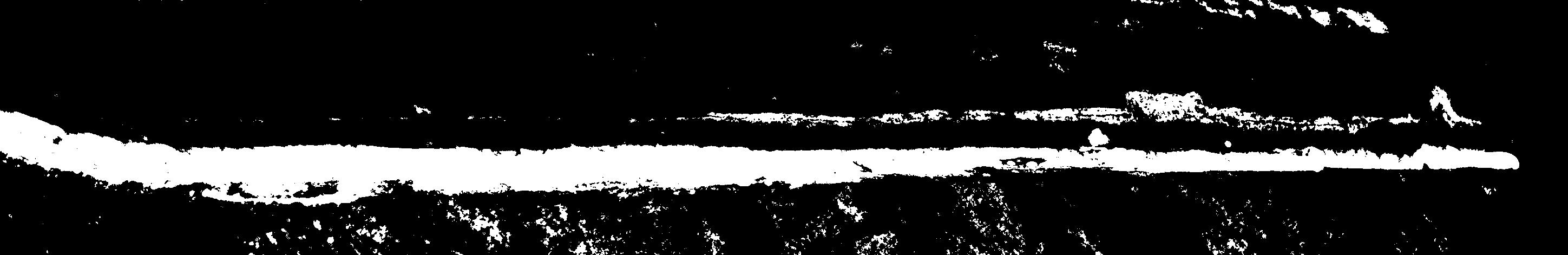

Supplement: S4 Data — (ZIP) [file pone.0297284.s004.zip › Level 2 processed Sample/processed_13/latex/WSO_latex.jpg]

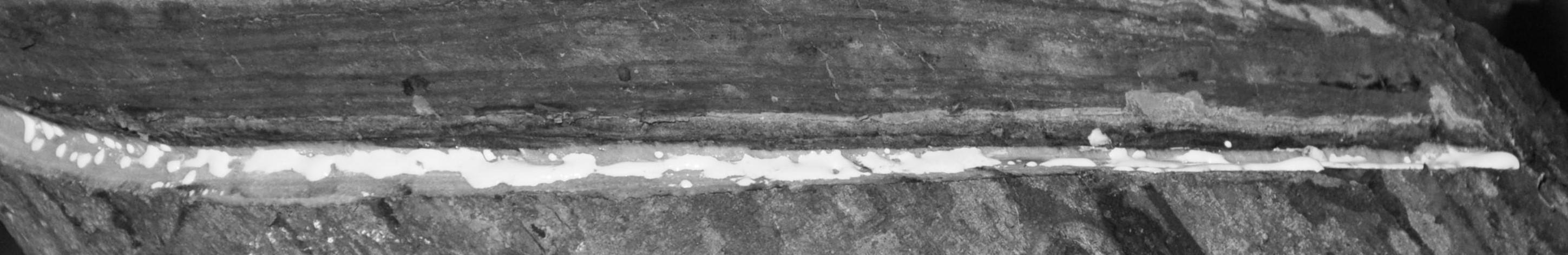

Supplement: S4 Data — (ZIP) [file pone.0297284.s004.zip › Level 2 processed Sample/processed_13/original_image.jpg]

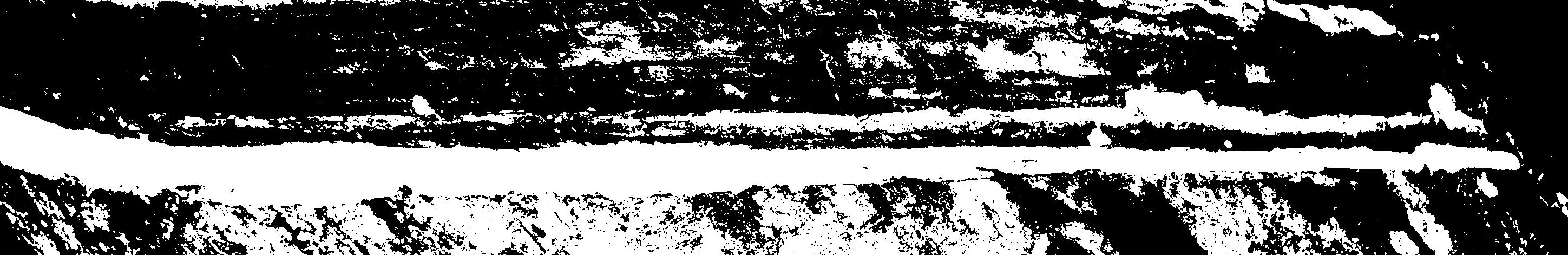

Supplement: S4 Data — (ZIP) [file pone.0297284.s004.zip › Level 2 processed Sample/processed_13/scar/AHA_scar.jpg]

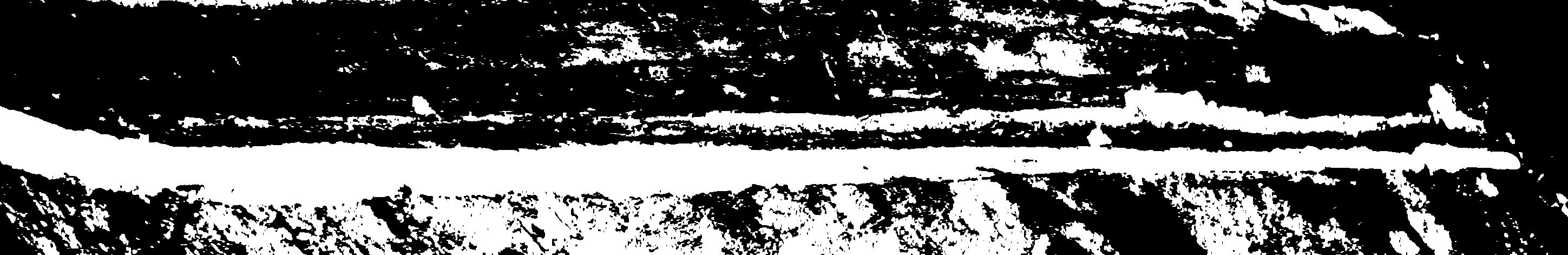

Supplement: S4 Data — (ZIP) [file pone.0297284.s004.zip › Level 2 processed Sample/processed_13/scar/DBO_scar.jpg]

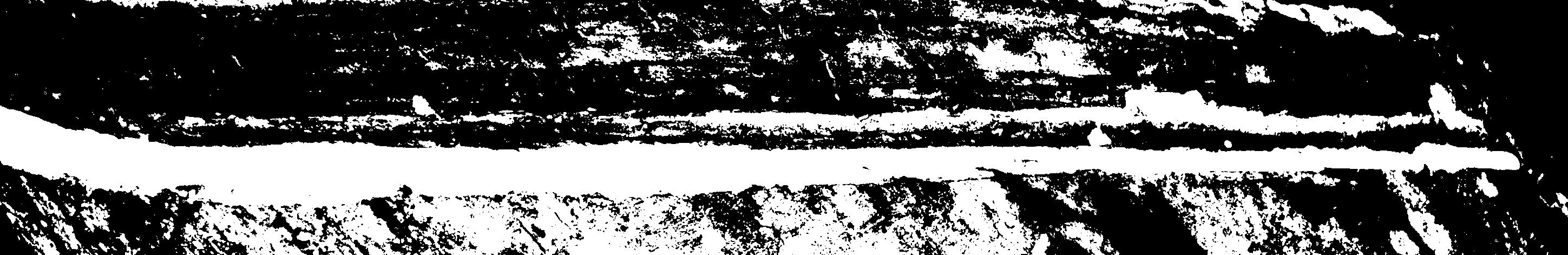

Supplement: S4 Data — (ZIP) [file pone.0297284.s004.zip › Level 2 processed Sample/processed_13/scar/WSO_scar.jpg]

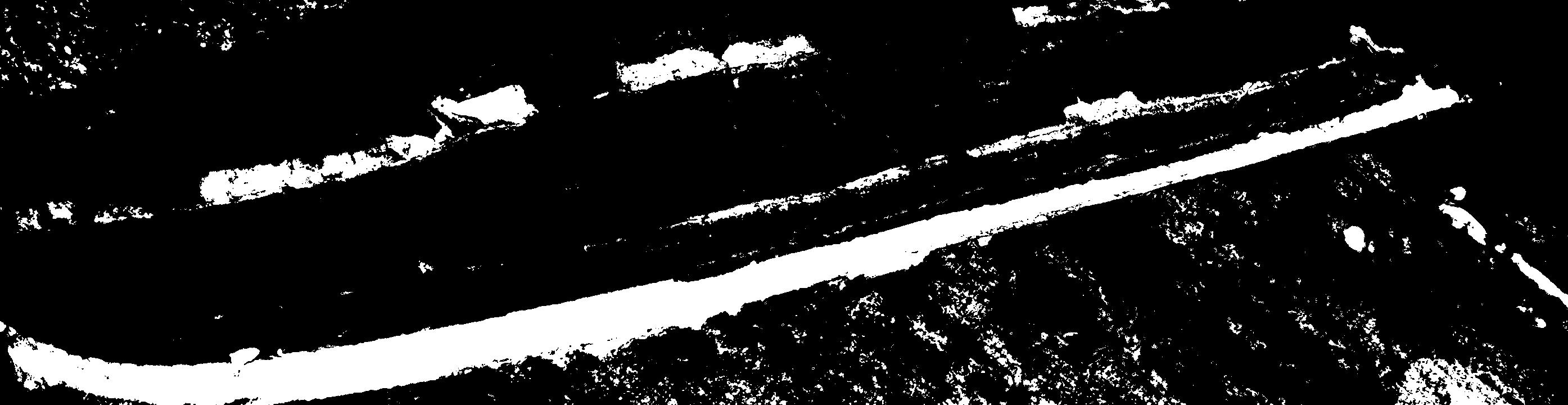

Supplement: S4 Data — (ZIP) [file pone.0297284.s004.zip › Level 2 processed Sample/processed_14/latex/AHA_latex.jpg]

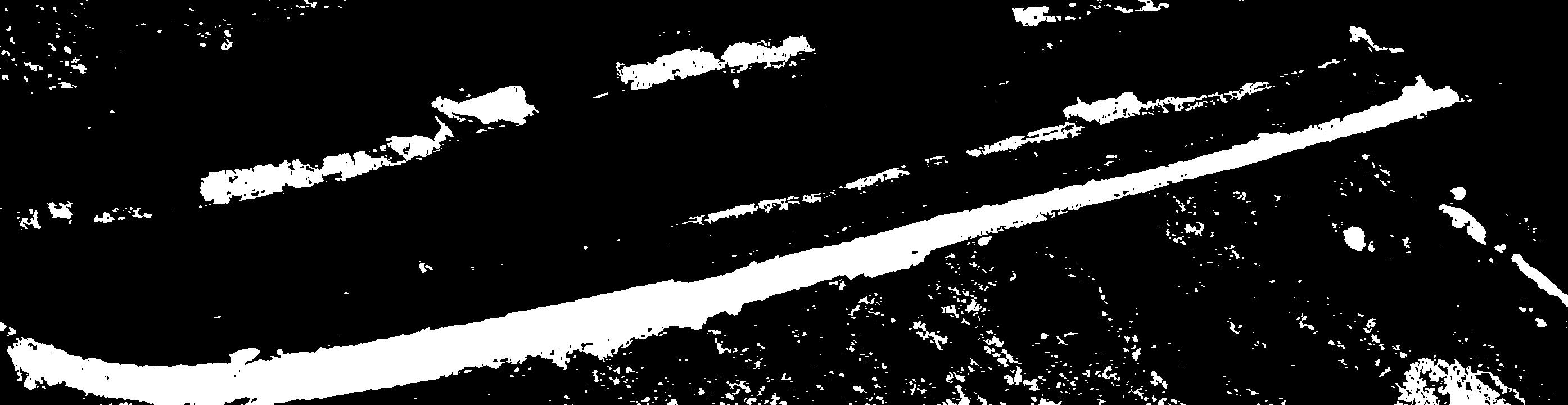

Supplement: S4 Data — (ZIP) [file pone.0297284.s004.zip › Level 2 processed Sample/processed_14/latex/DBO_latex.jpg]

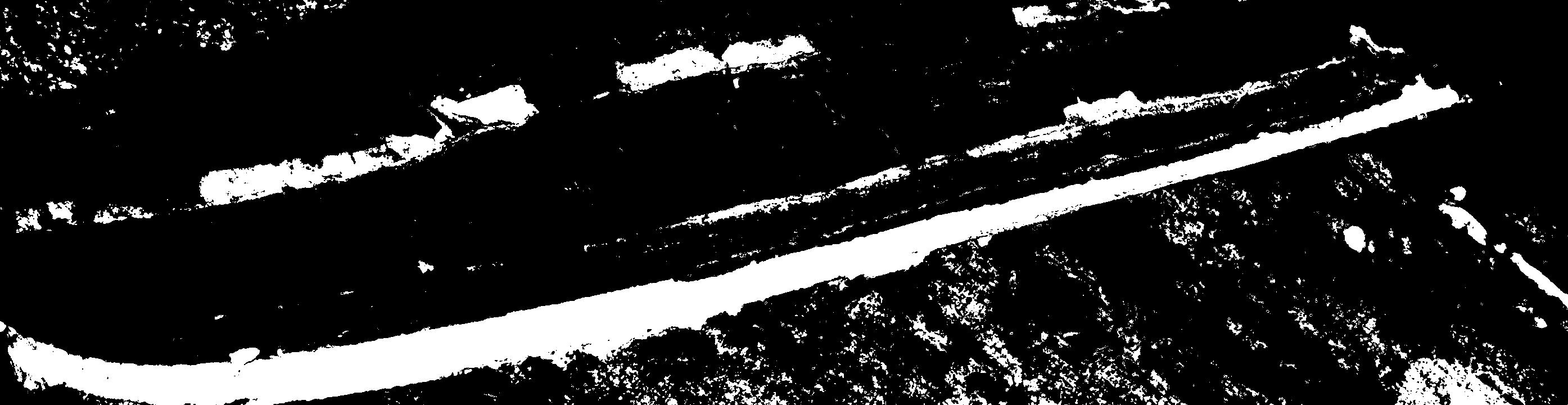

Supplement: S4 Data — (ZIP) [file pone.0297284.s004.zip › Level 2 processed Sample/processed_14/latex/GWO_latex.jpg]

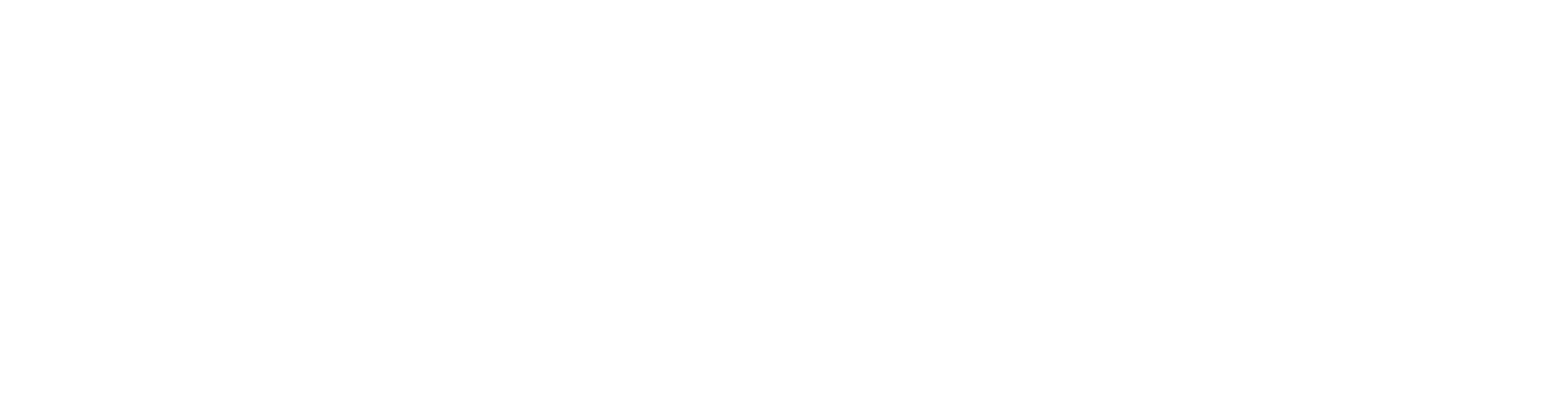

Supplement: S4 Data — (ZIP) [file pone.0297284.s004.zip › Level 2 processed Sample/processed_14/latex/OTSU_latex.jpg]

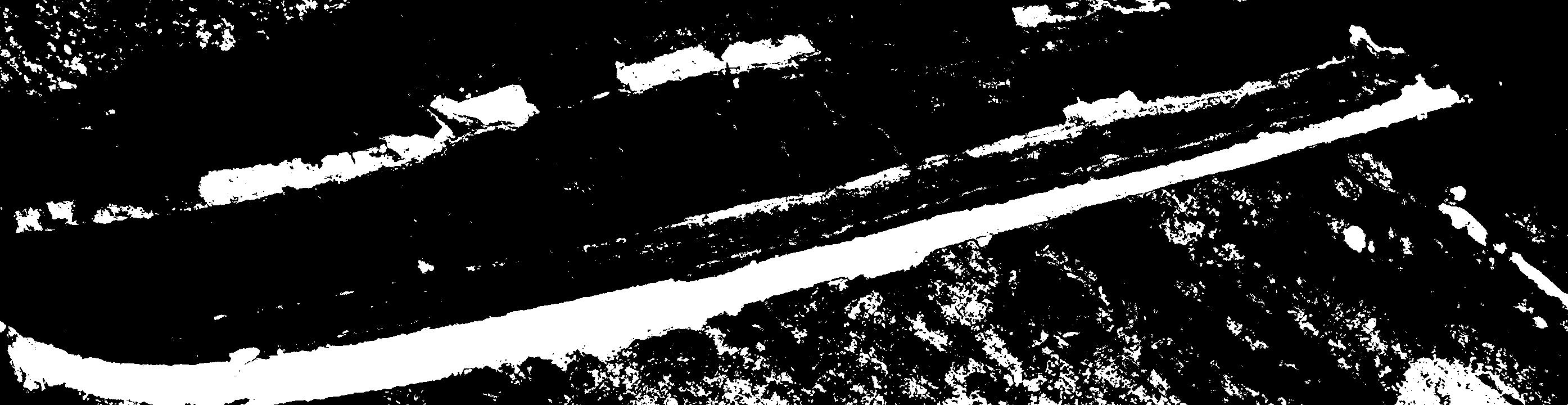

Supplement: S4 Data — (ZIP) [file pone.0297284.s004.zip › Level 2 processed Sample/processed_14/latex/WSO_latex.jpg]

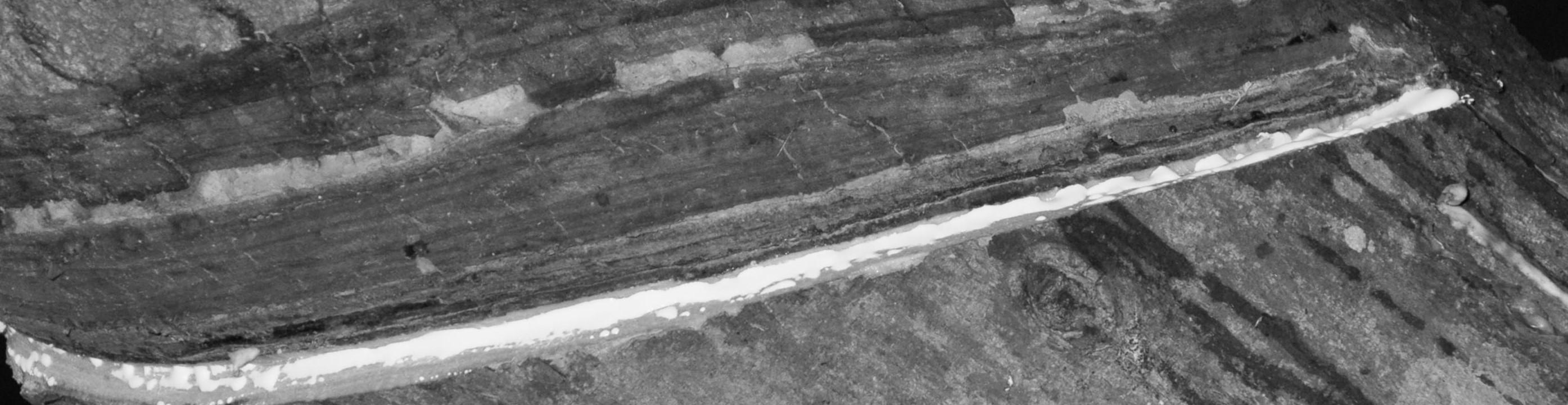

Supplement: S4 Data — (ZIP) [file pone.0297284.s004.zip › Level 2 processed Sample/processed_14/original_image.jpg]

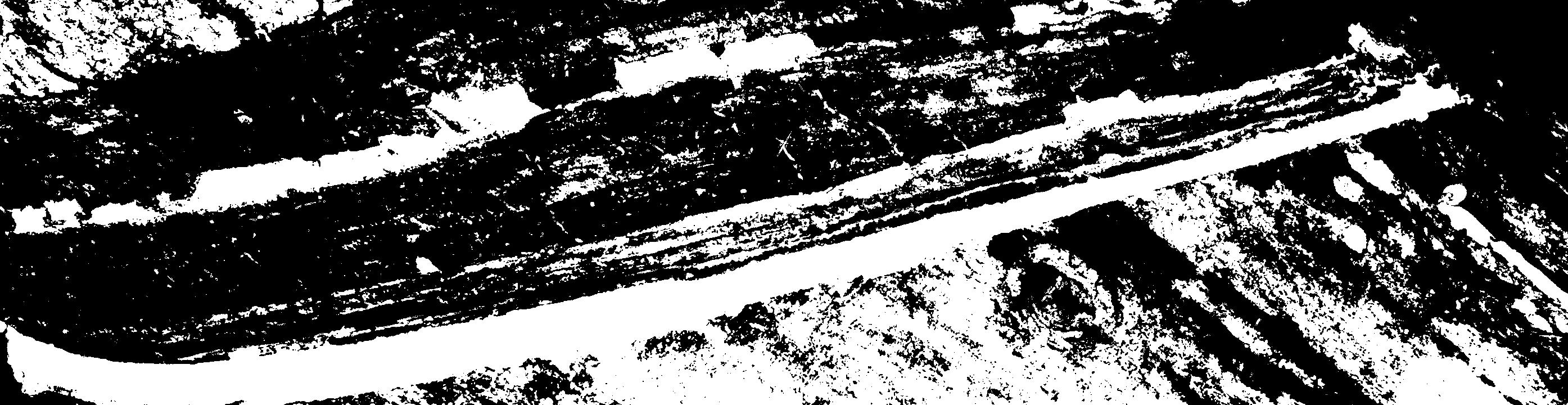

Supplement: S4 Data — (ZIP) [file pone.0297284.s004.zip › Level 2 processed Sample/processed_14/scar/AHA_scar.jpg]

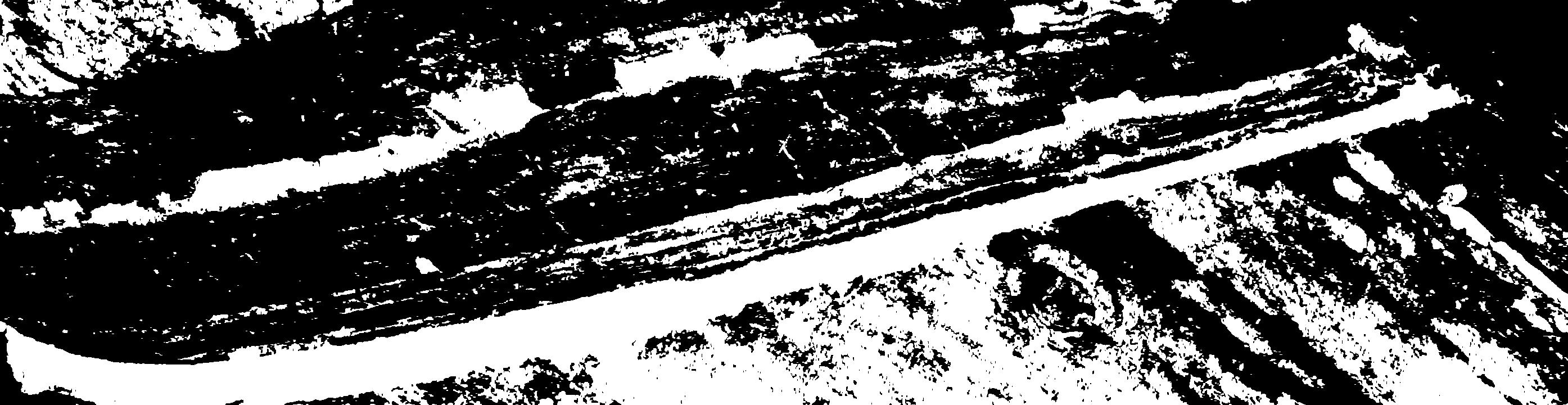

Supplement: S4 Data — (ZIP) [file pone.0297284.s004.zip › Level 2 processed Sample/processed_14/scar/DBO_scar.jpg]

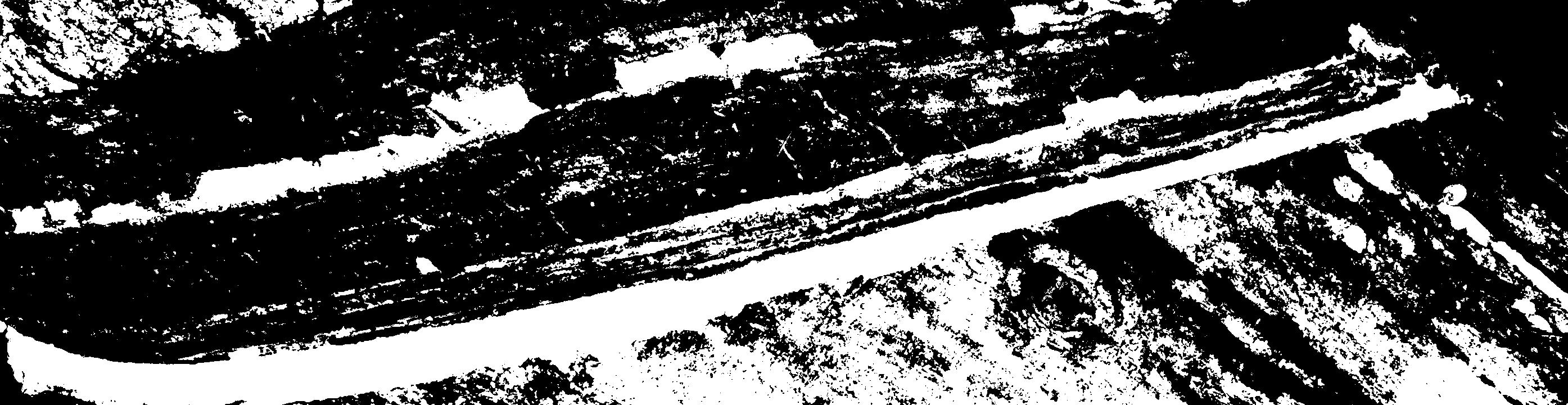

Supplement: S4 Data — (ZIP) [file pone.0297284.s004.zip › Level 2 processed Sample/processed_14/scar/WSO_scar.jpg]

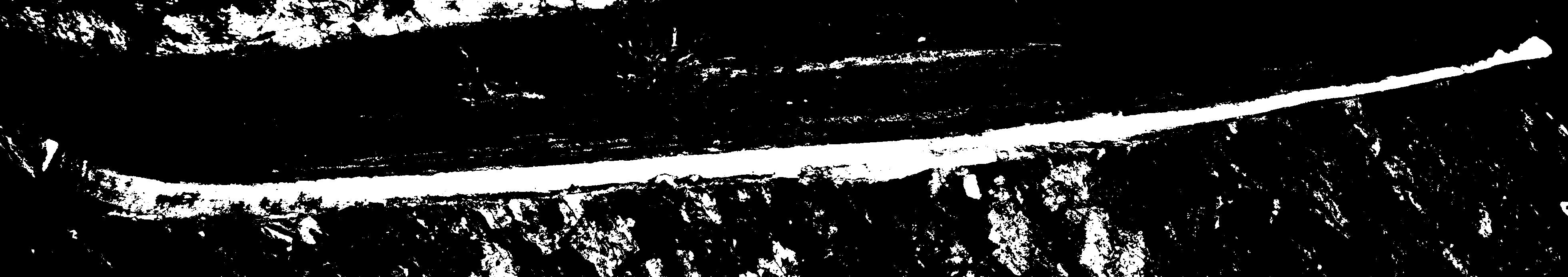

Supplement: S4 Data — (ZIP) [file pone.0297284.s004.zip › Level 2 processed Sample/processed_15/latex/AHA_latex.jpg]

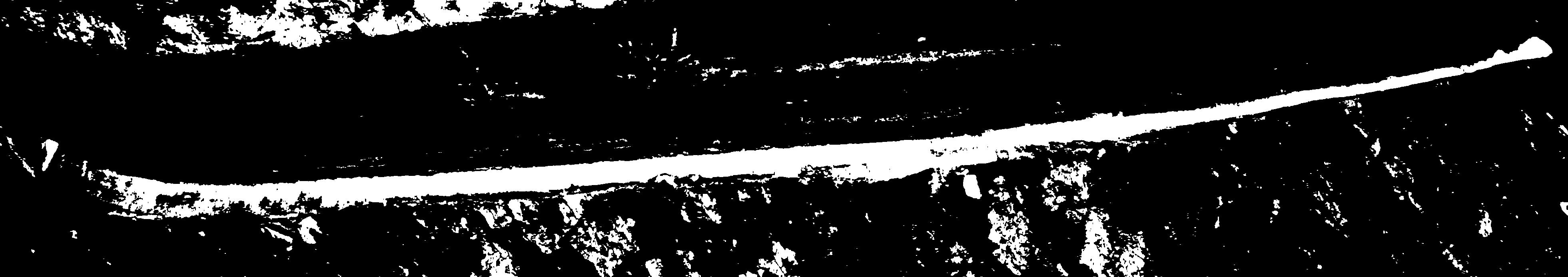

Supplement: S4 Data — (ZIP) [file pone.0297284.s004.zip › Level 2 processed Sample/processed_15/latex/DBO_latex.jpg]

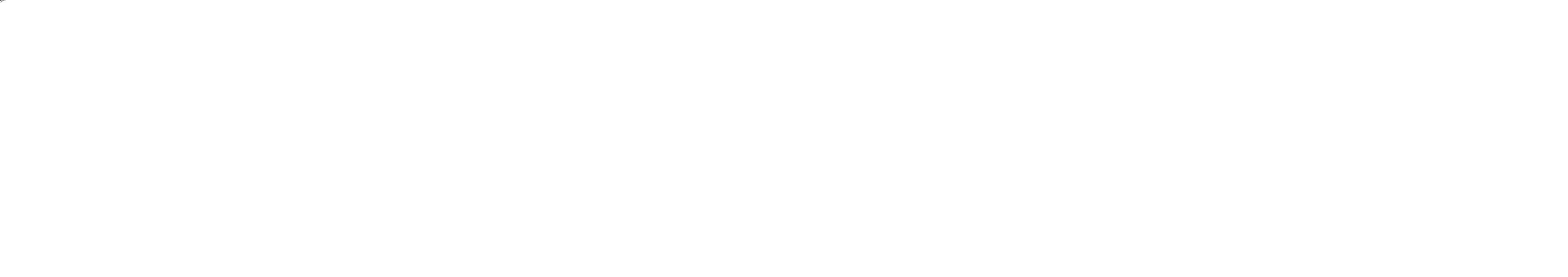

Supplement: S4 Data — (ZIP) [file pone.0297284.s004.zip › Level 2 processed Sample/processed_15/latex/OTSU_latex.jpg]

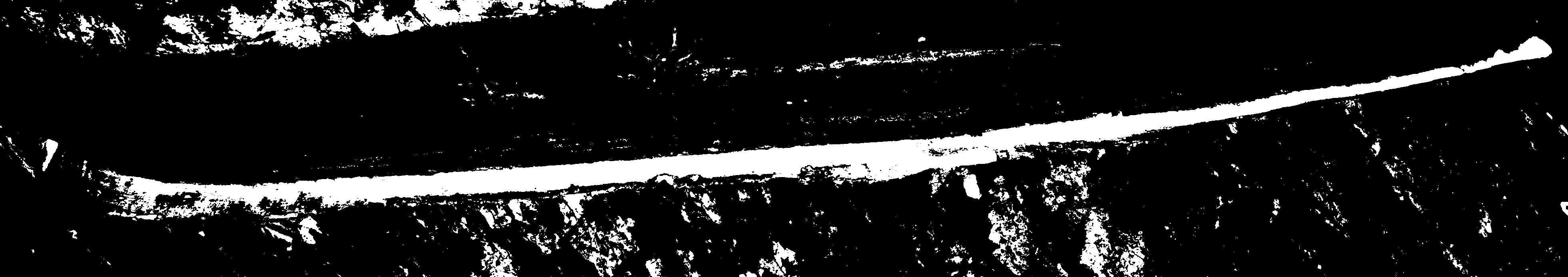

Supplement: S4 Data — (ZIP) [file pone.0297284.s004.zip › Level 2 processed Sample/processed_15/latex/WSO_latex.jpg]

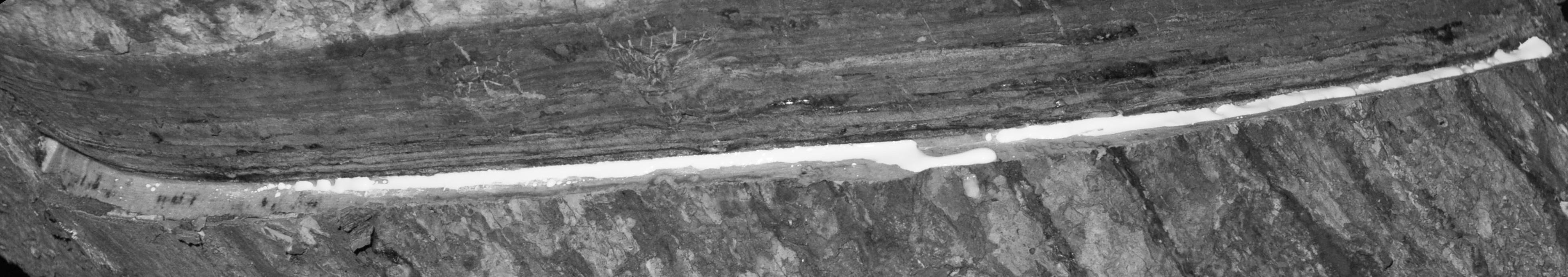

Supplement: S4 Data — (ZIP) [file pone.0297284.s004.zip › Level 2 processed Sample/processed_15/original_image.jpg]

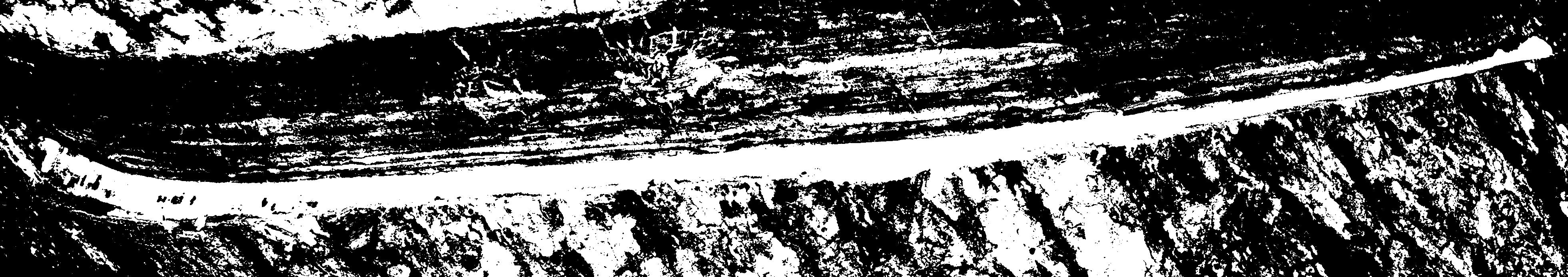

Supplement: S4 Data — (ZIP) [file pone.0297284.s004.zip › Level 2 processed Sample/processed_15/scar/AHA_scar.jpg]

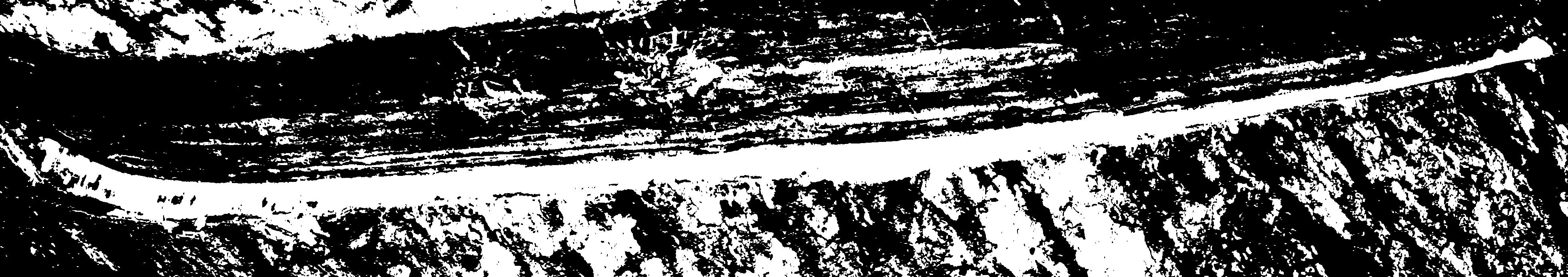

Supplement: S4 Data — (ZIP) [file pone.0297284.s004.zip › Level 2 processed Sample/processed_15/scar/DBO_scar.jpg]

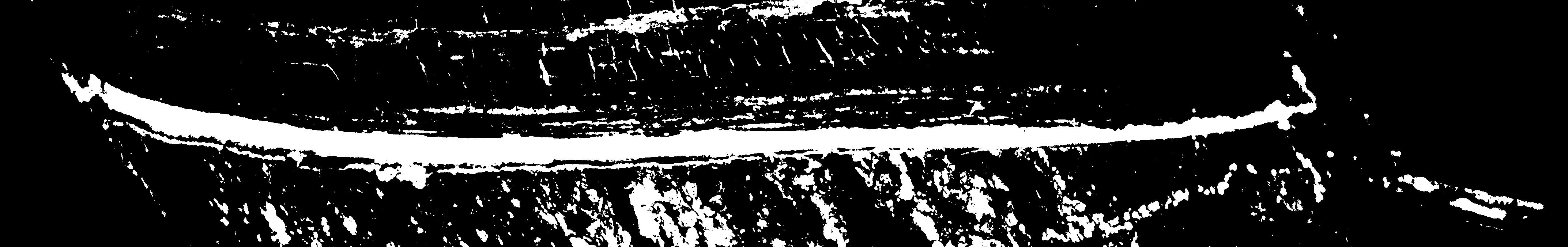

Supplement: S4 Data — (ZIP) [file pone.0297284.s004.zip › Level 2 processed Sample/processed_16/latex/AHA_latex.jpg]

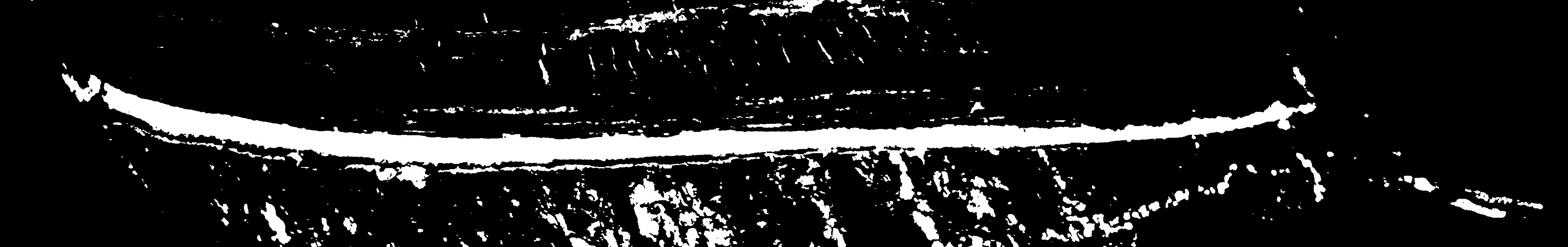

Supplement: S4 Data — (ZIP) [file pone.0297284.s004.zip › Level 2 processed Sample/processed_16/latex/DBO_latex.jpg]

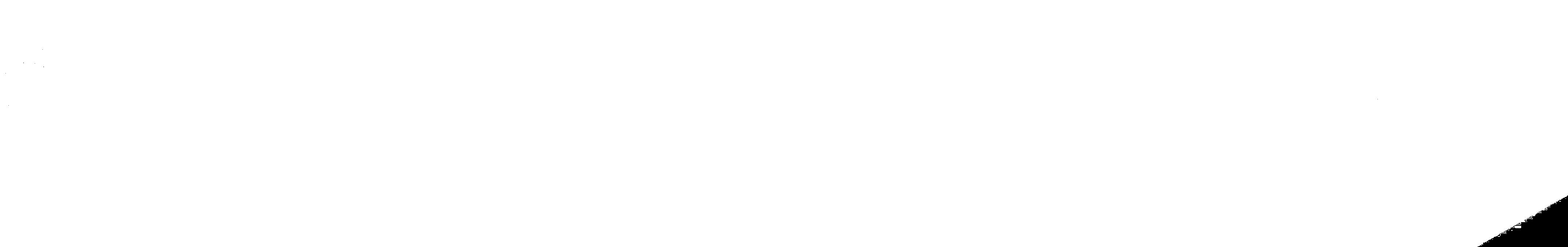

Supplement: S4 Data — (ZIP) [file pone.0297284.s004.zip › Level 2 processed Sample/processed_16/latex/OTSU_latex.jpg]

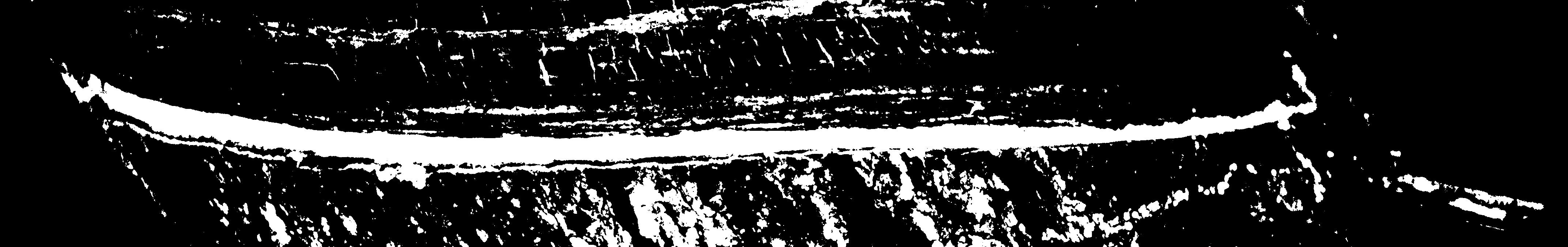

Supplement: S4 Data — (ZIP) [file pone.0297284.s004.zip › Level 2 processed Sample/processed_16/latex/WSO_latex.jpg]

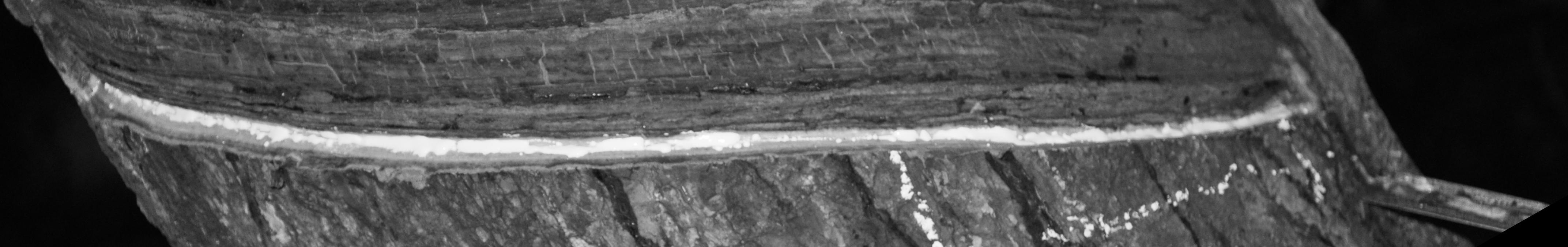

Supplement: S4 Data — (ZIP) [file pone.0297284.s004.zip › Level 2 processed Sample/processed_16/original_image.jpg]

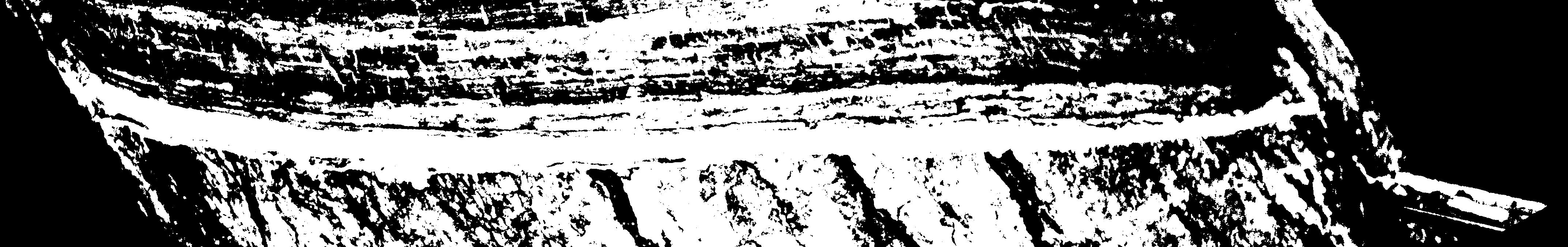

Supplement: S4 Data — (ZIP) [file pone.0297284.s004.zip › Level 2 processed Sample/processed_16/scar/AHA_scar.jpg]

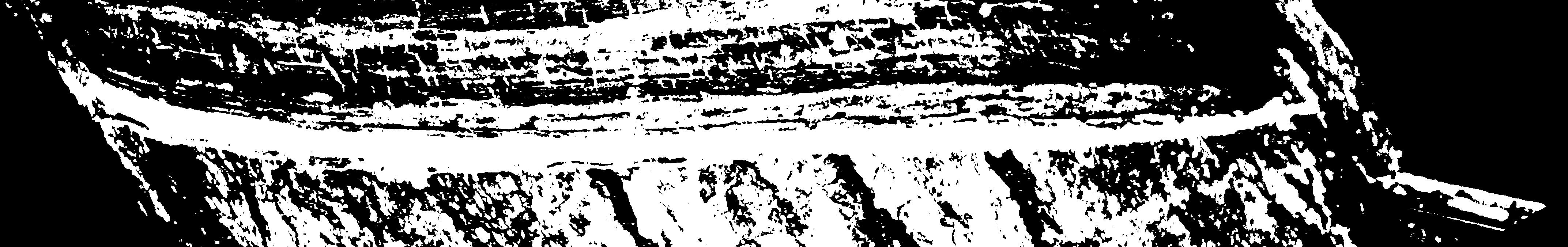

Supplement: S4 Data — (ZIP) [file pone.0297284.s004.zip › Level 2 processed Sample/processed_16/scar/DBO_scar.jpg]

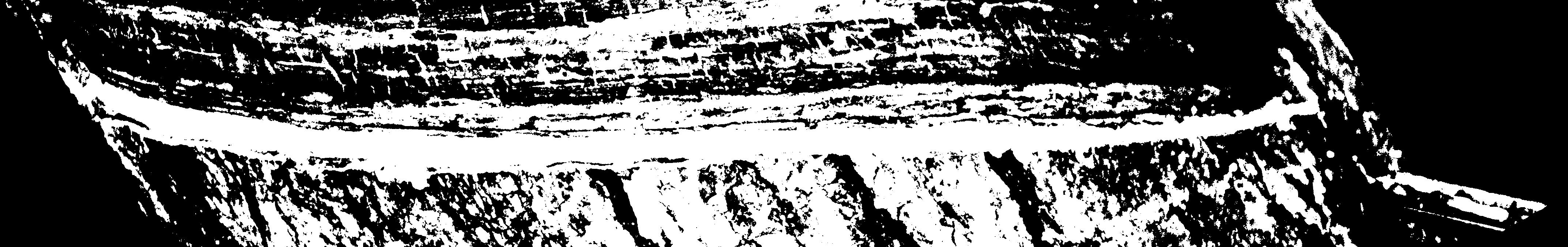

Supplement: S4 Data — (ZIP) [file pone.0297284.s004.zip › Level 2 processed Sample/processed_16/scar/WSO_scar.jpg]

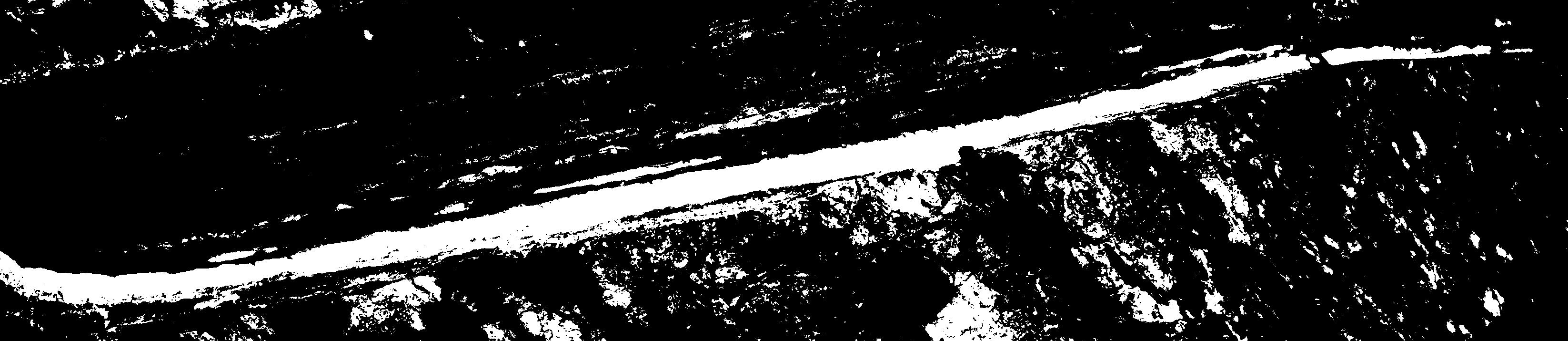

Supplement: S4 Data — (ZIP) [file pone.0297284.s004.zip › Level 2 processed Sample/processed_17/latex/AHA_latex.jpg]

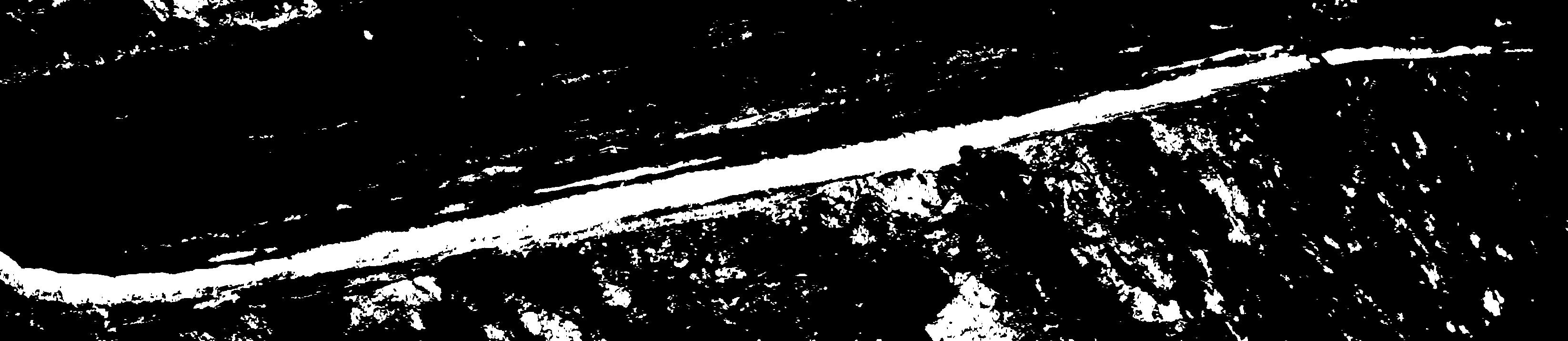

Supplement: S4 Data — (ZIP) [file pone.0297284.s004.zip › Level 2 processed Sample/processed_17/latex/DBO_latex.jpg]

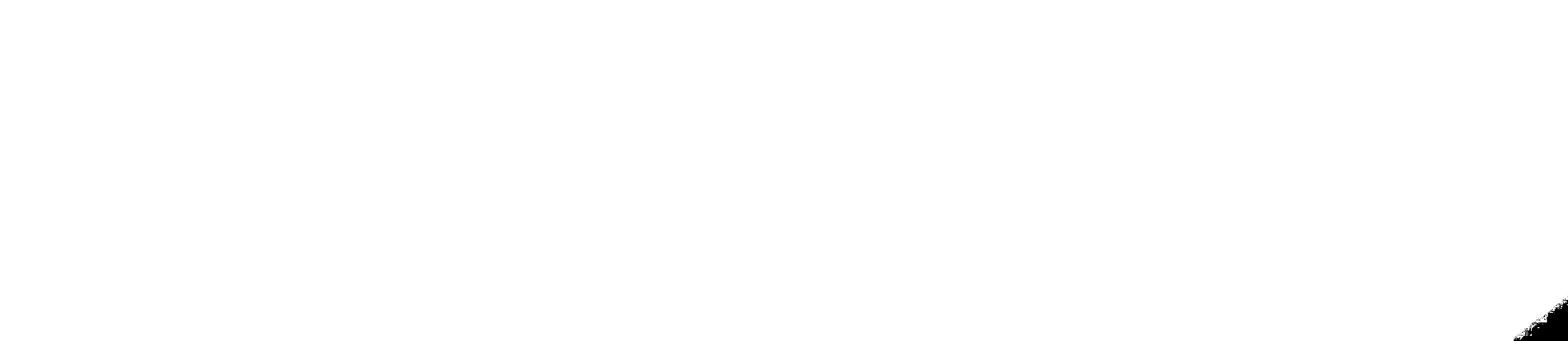

Supplement: S4 Data — (ZIP) [file pone.0297284.s004.zip › Level 2 processed Sample/processed_17/latex/OTSU_latex.jpg]

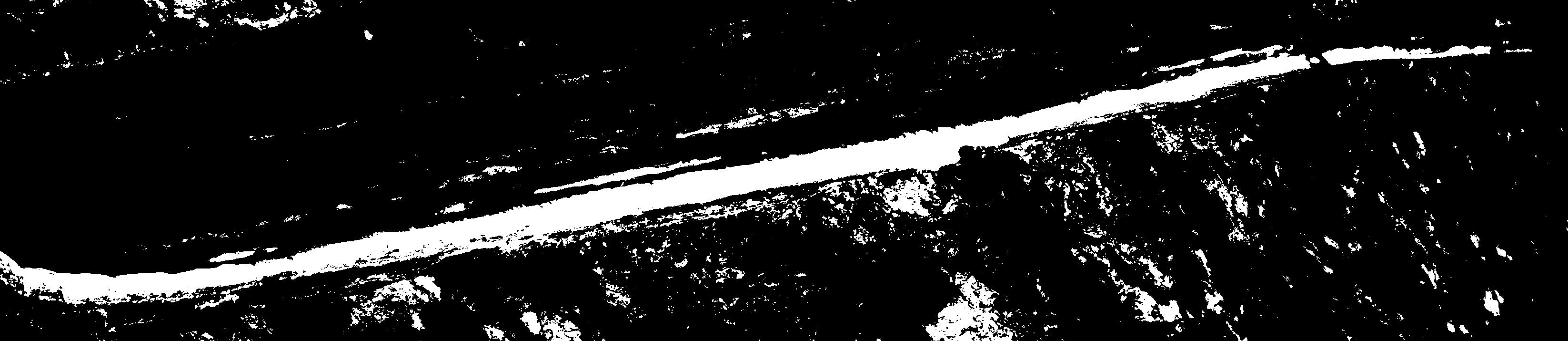

Supplement: S4 Data — (ZIP) [file pone.0297284.s004.zip › Level 2 processed Sample/processed_17/latex/WSO_latex.jpg]

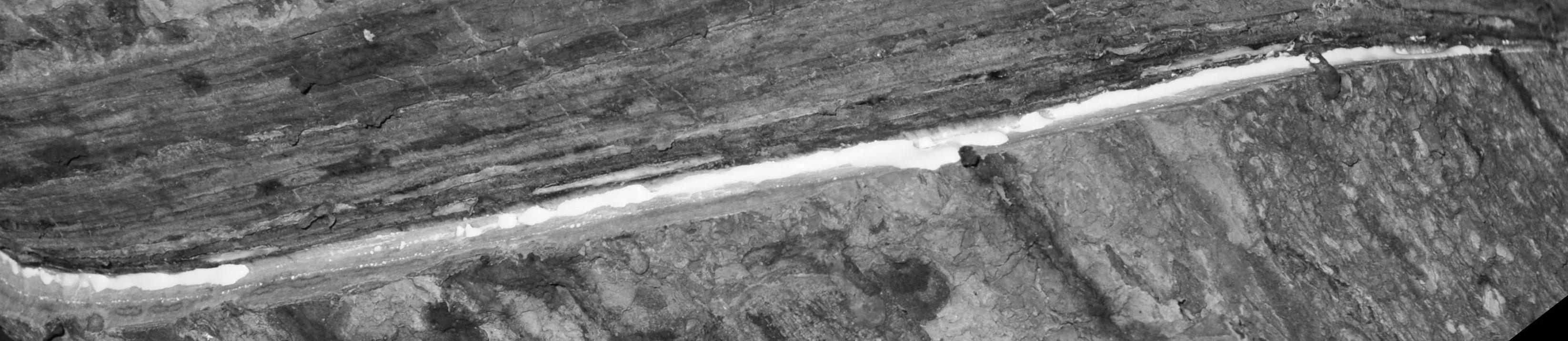

Supplement: S4 Data — (ZIP) [file pone.0297284.s004.zip › Level 2 processed Sample/processed_17/original_image.jpg]

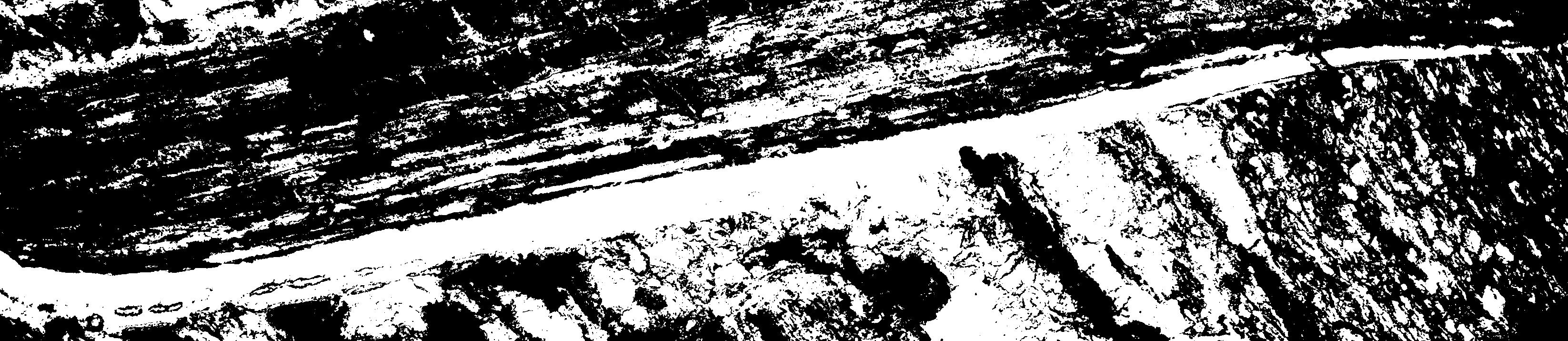

Supplement: S4 Data — (ZIP) [file pone.0297284.s004.zip › Level 2 processed Sample/processed_17/scar/AHA_scar.jpg]

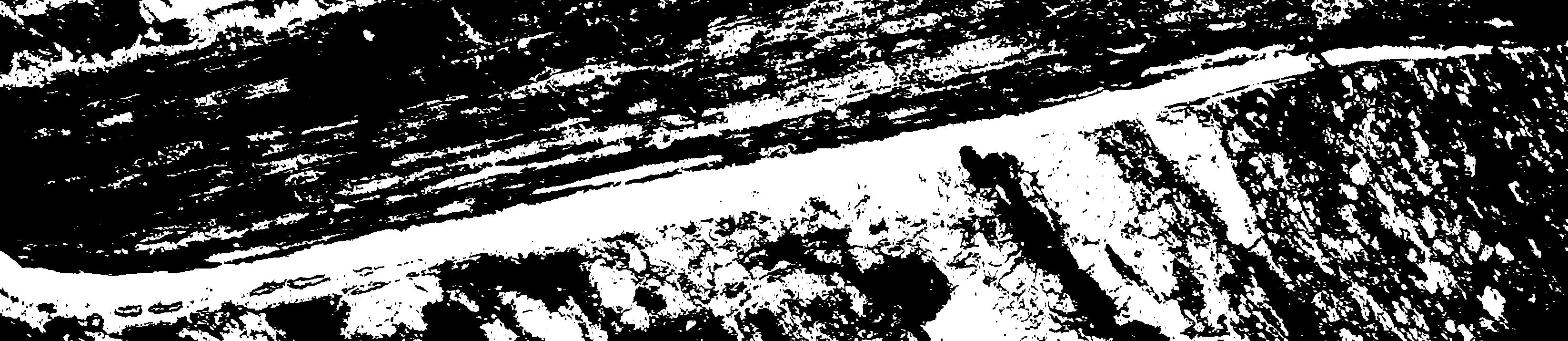

Supplement: S4 Data — (ZIP) [file pone.0297284.s004.zip › Level 2 processed Sample/processed_17/scar/DBO_scar.jpg]

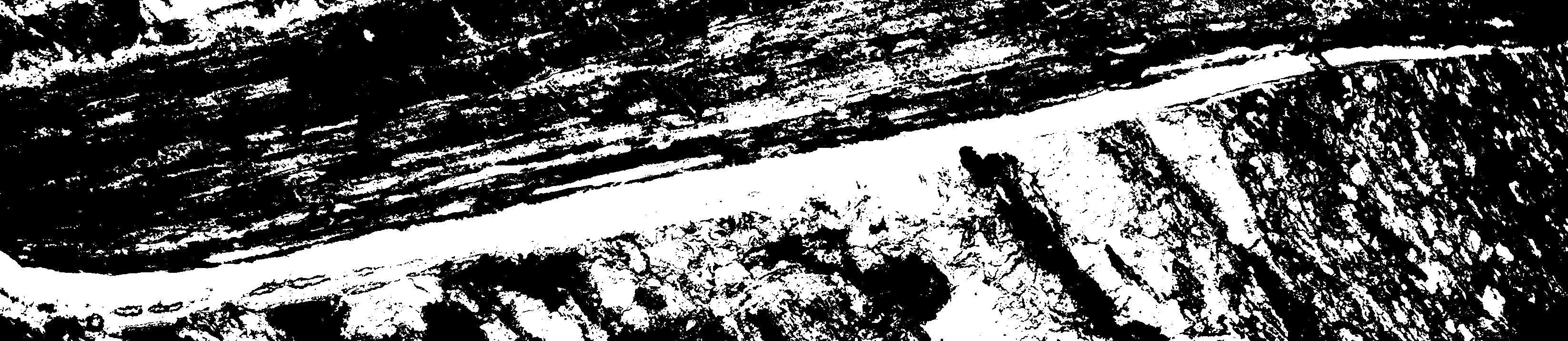

Supplement: S4 Data — (ZIP) [file pone.0297284.s004.zip › Level 2 processed Sample/processed_17/scar/WSO_scar.jpg]

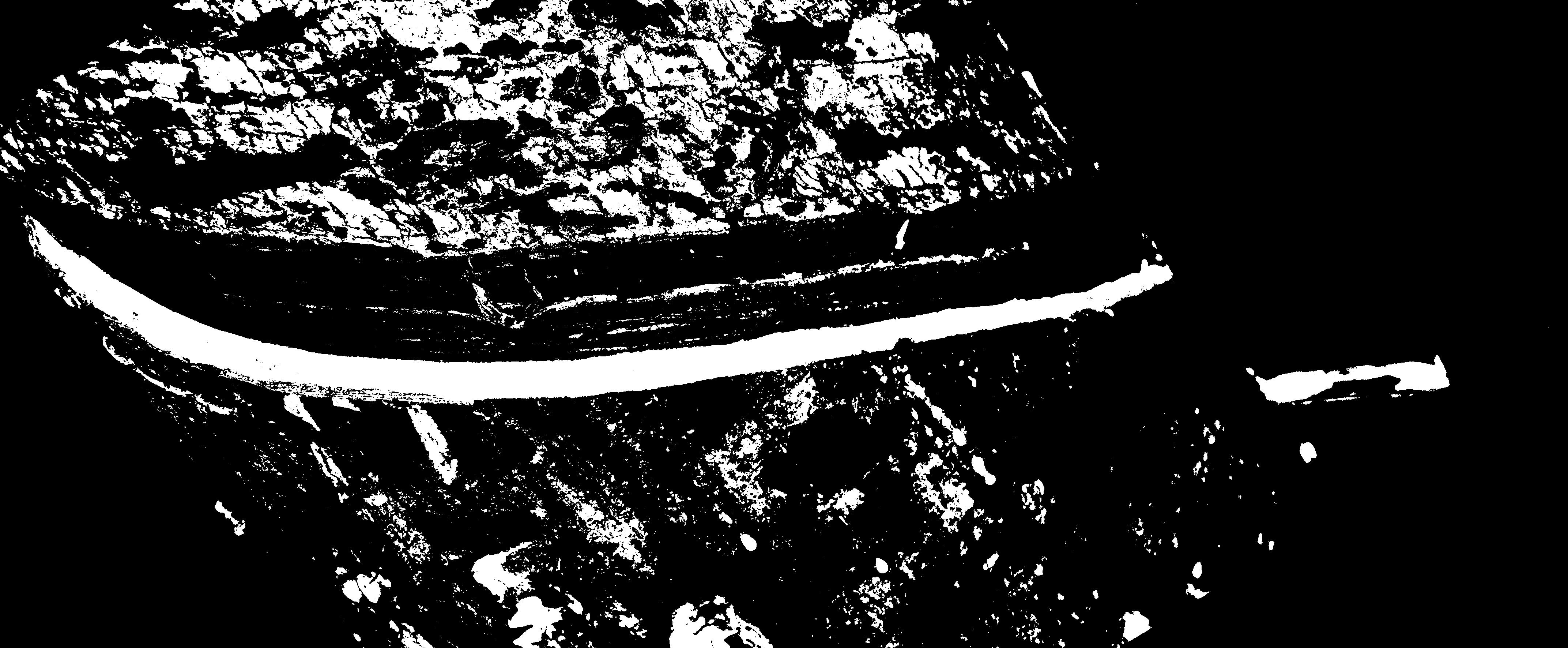

Supplement: S4 Data — (ZIP) [file pone.0297284.s004.zip › Level 2 processed Sample/processed_18/latex/AHA_latex.jpg]

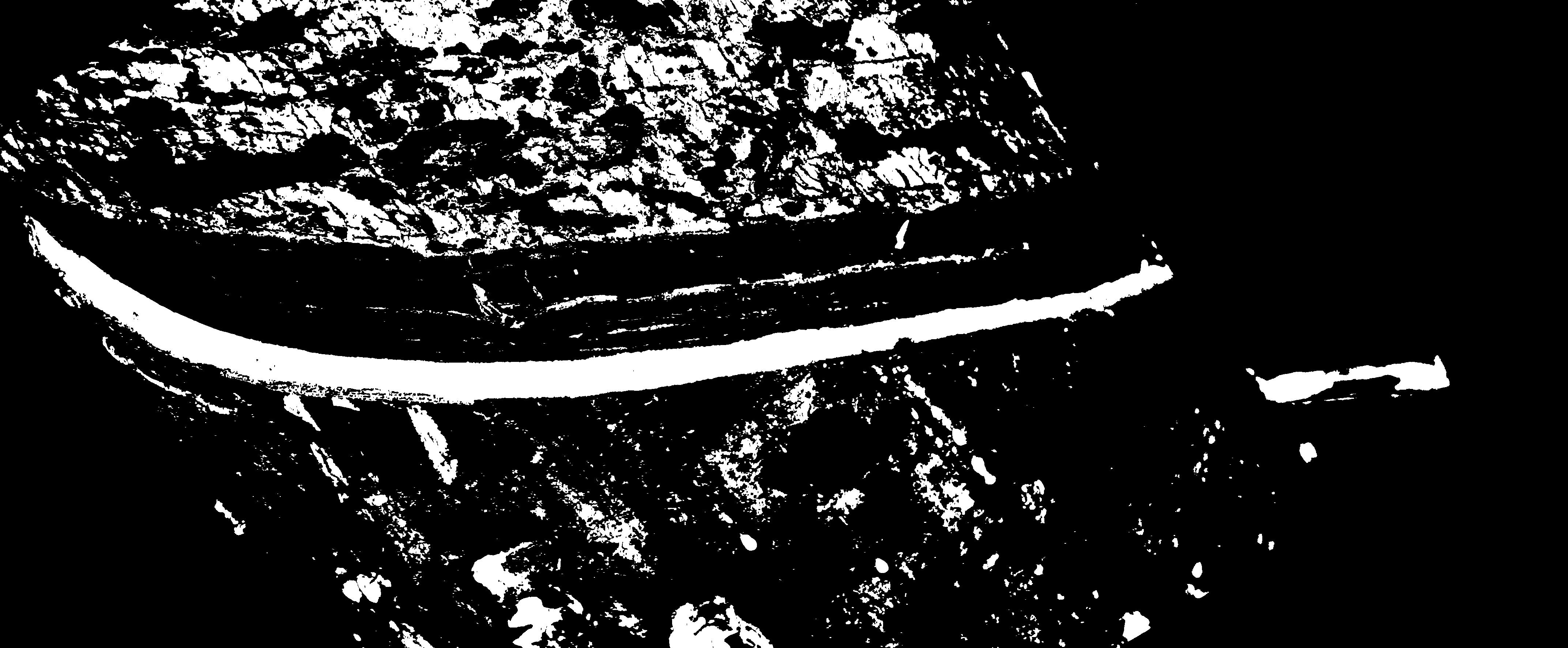

Supplement: S4 Data — (ZIP) [file pone.0297284.s004.zip › Level 2 processed Sample/processed_18/latex/DBO_latex.jpg]

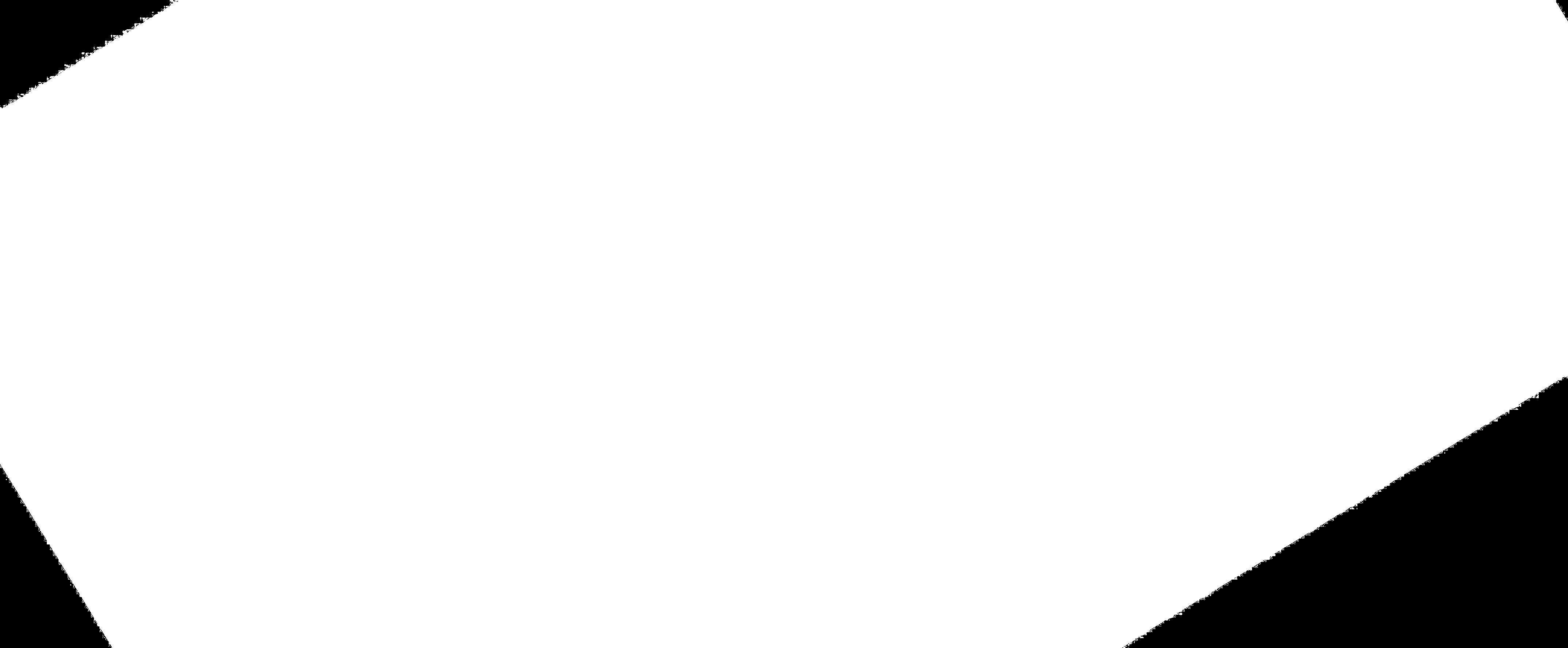

Supplement: S4 Data — (ZIP) [file pone.0297284.s004.zip › Level 2 processed Sample/processed_18/latex/OTSU_latex.jpg]

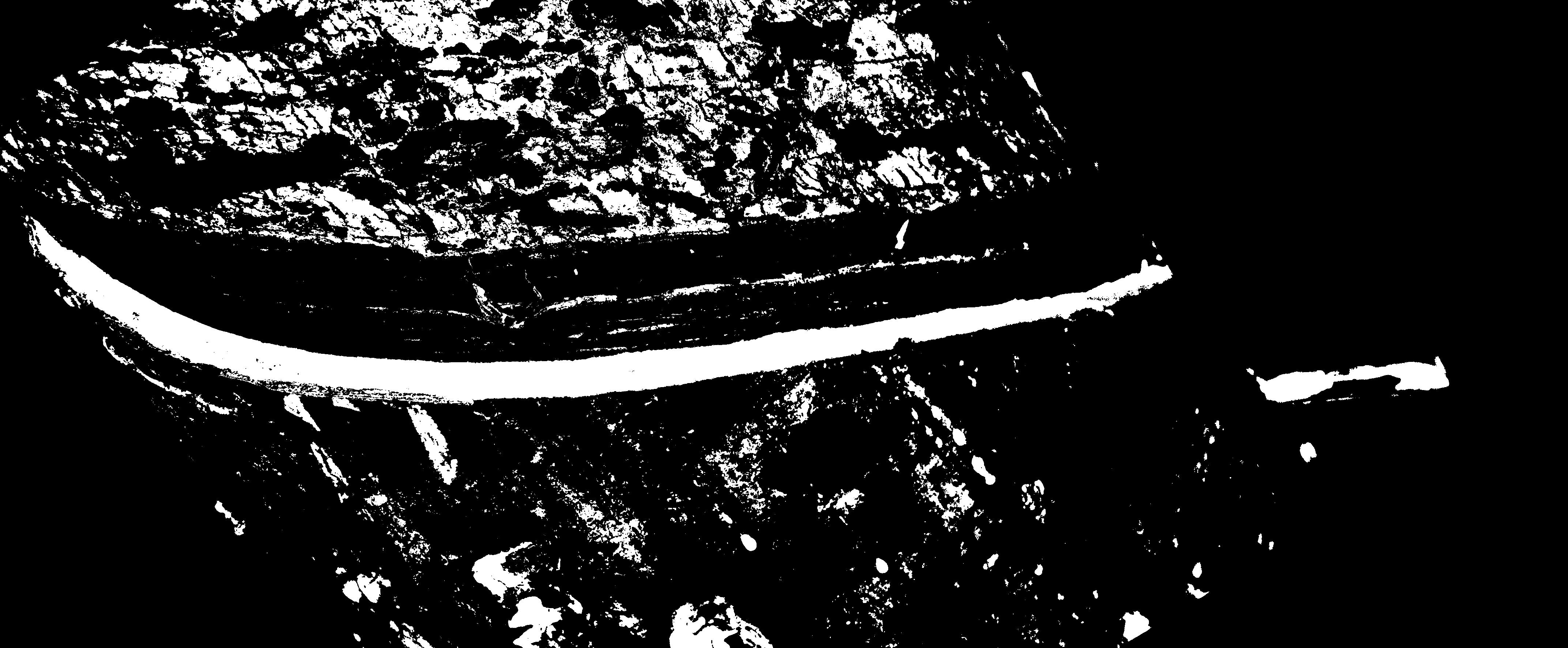

Supplement: S4 Data — (ZIP) [file pone.0297284.s004.zip › Level 2 processed Sample/processed_18/latex/WSO_latex.jpg]

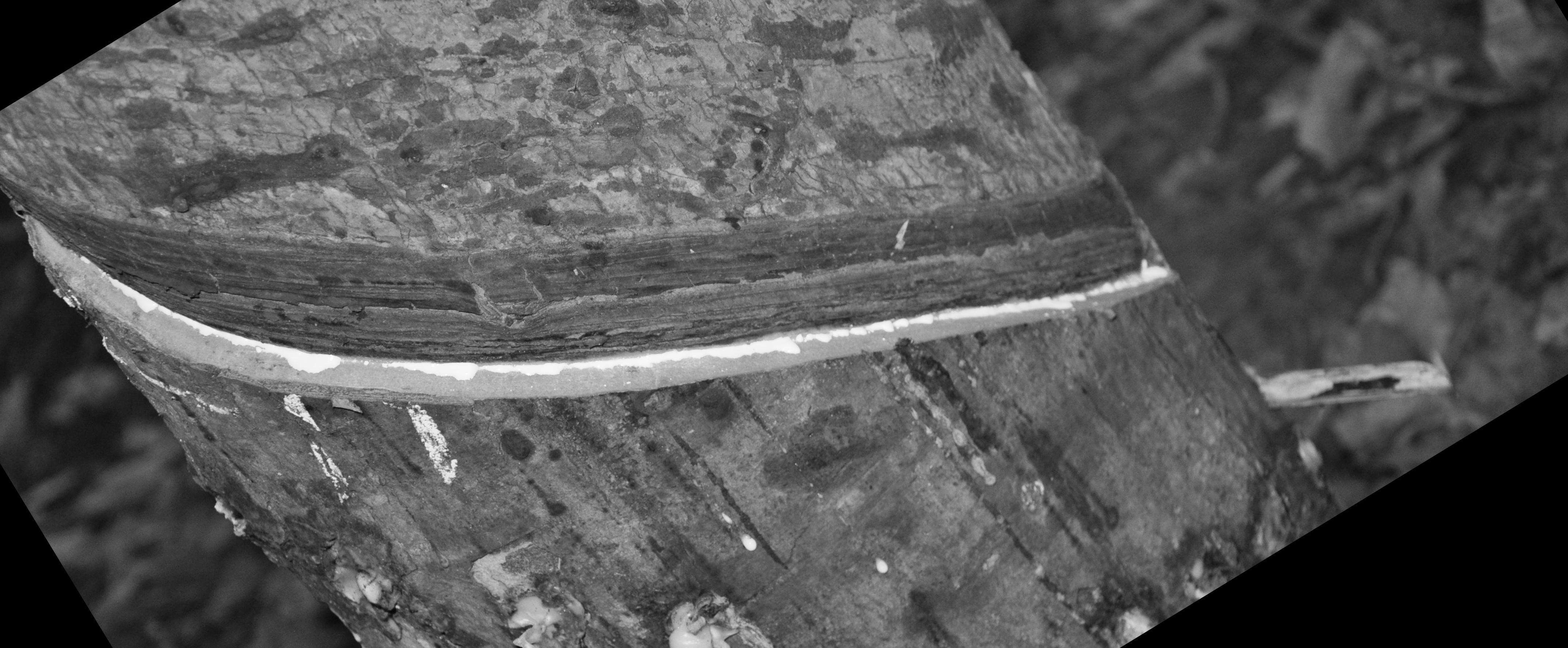

Supplement: S4 Data — (ZIP) [file pone.0297284.s004.zip › Level 2 processed Sample/processed_18/original_image.jpg]

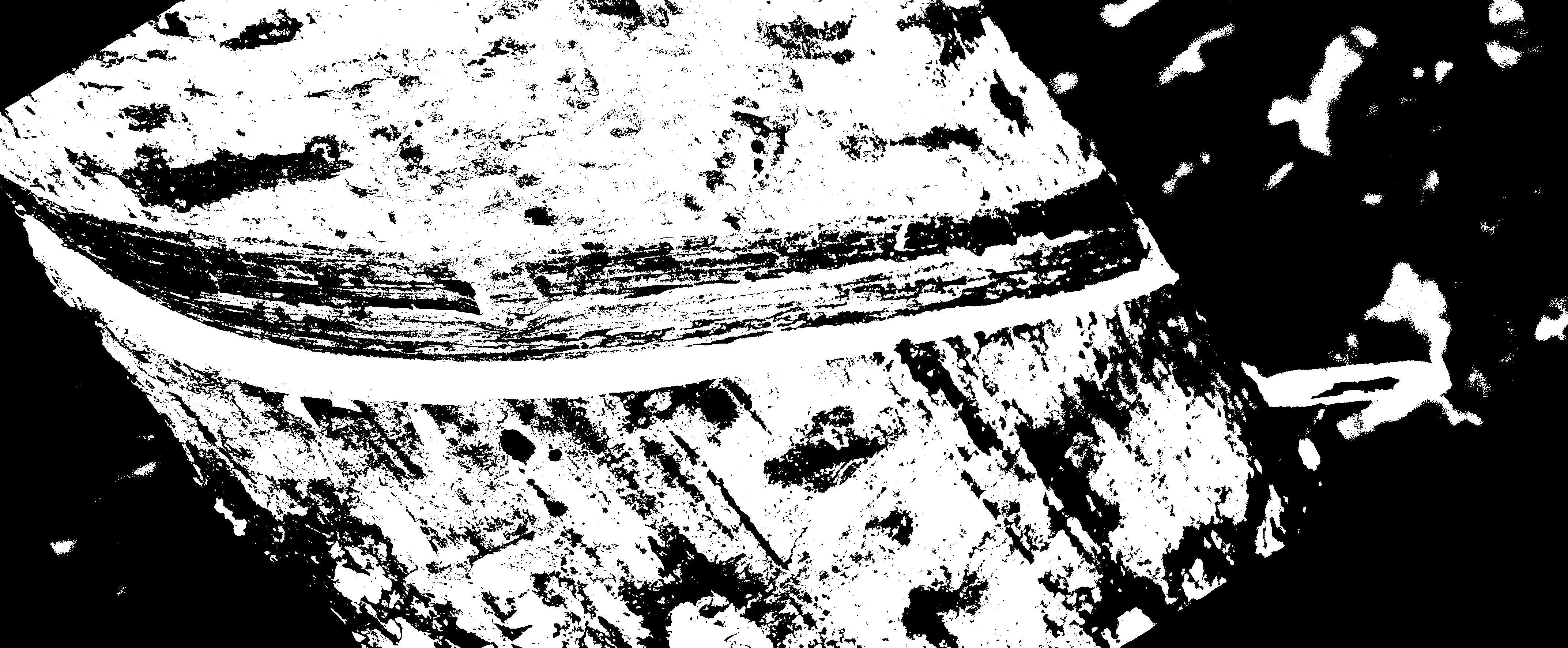

Supplement: S4 Data — (ZIP) [file pone.0297284.s004.zip › Level 2 processed Sample/processed_18/scar/AHA_scar.jpg]

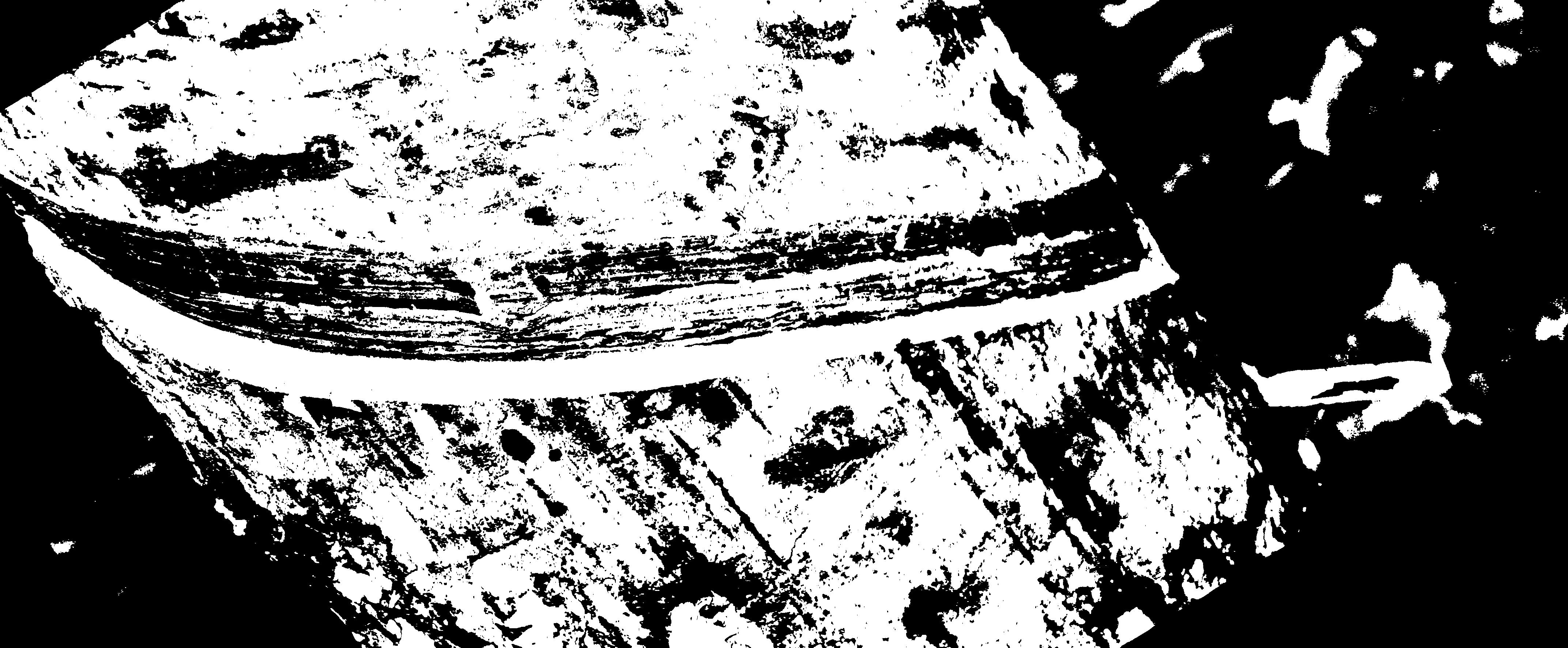

Supplement: S4 Data — (ZIP) [file pone.0297284.s004.zip › Level 2 processed Sample/processed_18/scar/DBO_scar.jpg]

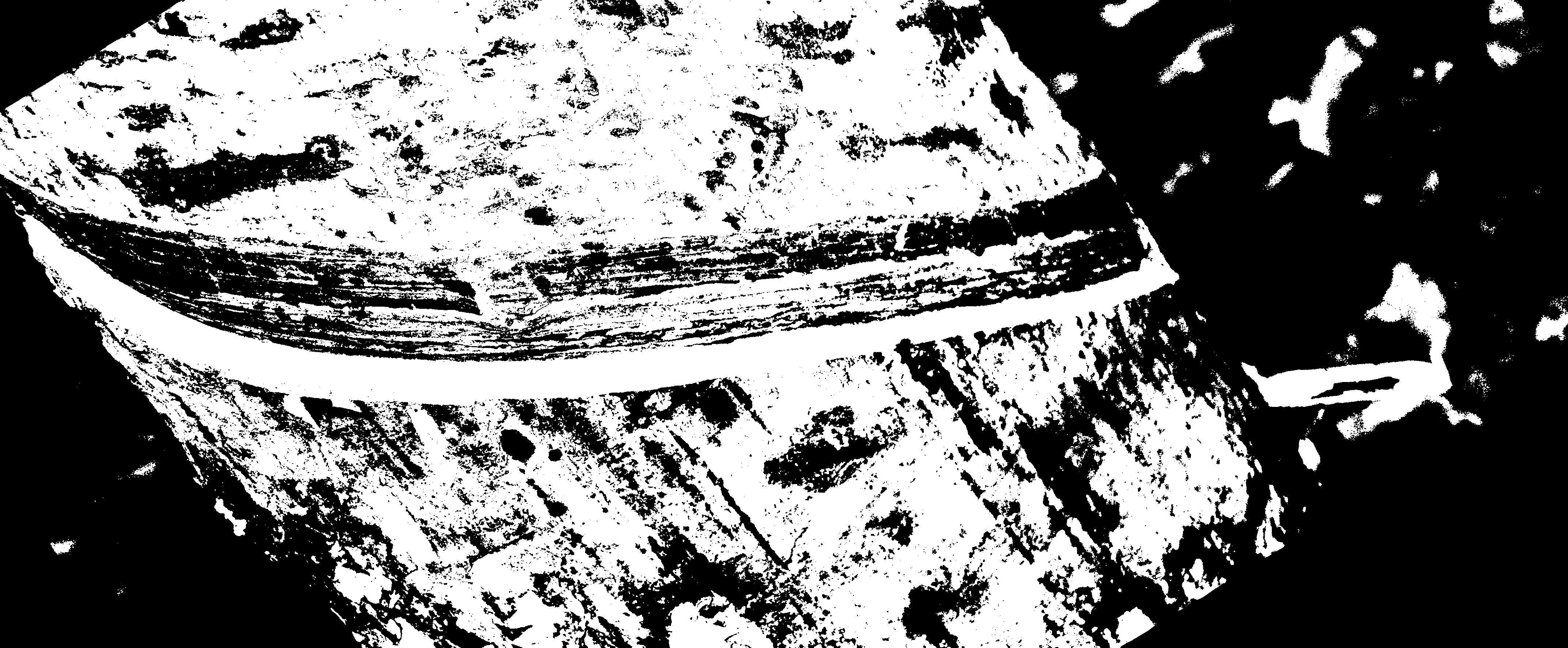

Supplement: S4 Data — (ZIP) [file pone.0297284.s004.zip › Level 2 processed Sample/processed_18/scar/WOA_scar.jpg]

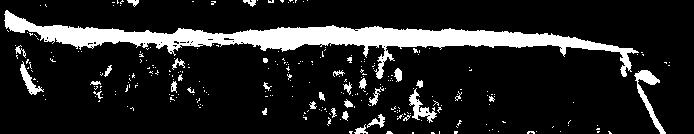

Supplement: S4 Data — (ZIP) [file pone.0297284.s004.zip › Level 2 processed Sample/processed_3/latex/AHA_latex.jpg]

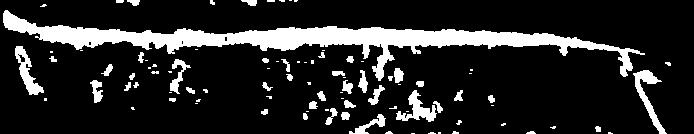

Supplement: S4 Data — (ZIP) [file pone.0297284.s004.zip › Level 2 processed Sample/processed_3/latex/DBO_latex.jpg]

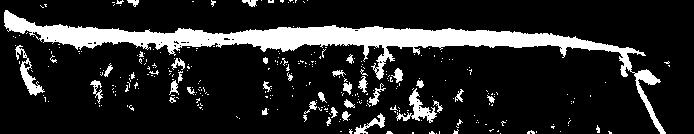

Supplement: S4 Data — (ZIP) [file pone.0297284.s004.zip › Level 2 processed Sample/processed_3/latex/GWO_latex.jpg]

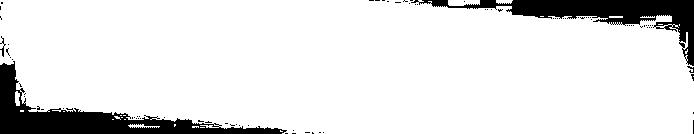

Supplement: S4 Data — (ZIP) [file pone.0297284.s004.zip › Level 2 processed Sample/processed_3/latex/OTSU_latex.jpg]

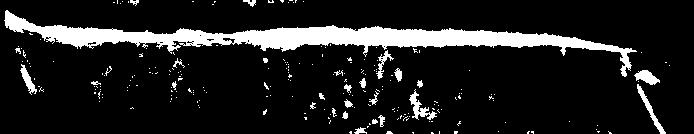

Supplement: S4 Data — (ZIP) [file pone.0297284.s004.zip › Level 2 processed Sample/processed_3/latex/WSO_latex.jpg]

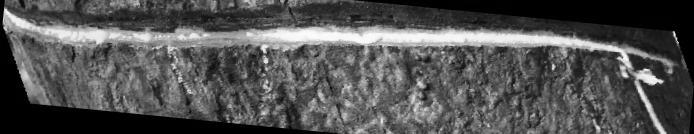

Supplement: S4 Data — (ZIP) [file pone.0297284.s004.zip › Level 2 processed Sample/processed_3/original_image.jpg]

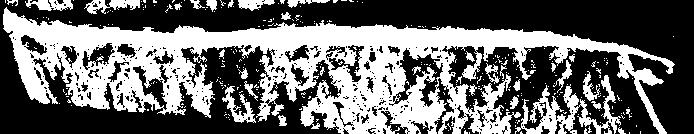

Supplement: S4 Data — (ZIP) [file pone.0297284.s004.zip › Level 2 processed Sample/processed_3/scar/AHA_scar.jpg]

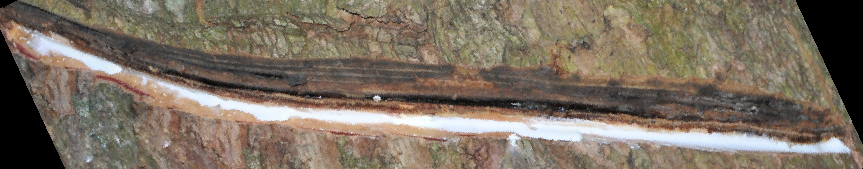

Supplement: S5 Data — (ZIP) [file pone.0297284.s005.zip › Level 3 Original Sample/3-1.jpg]

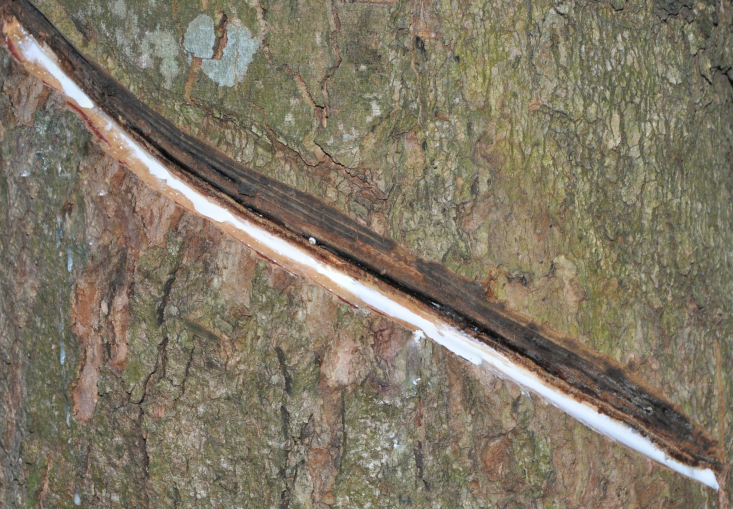

Supplement: S5 Data — (ZIP) [file pone.0297284.s005.zip › Level 3 Original Sample/3-1.png]

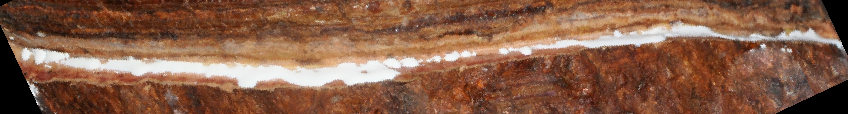

Supplement: S5 Data — (ZIP) [file pone.0297284.s005.zip › Level 3 Original Sample/3-2.jpg]

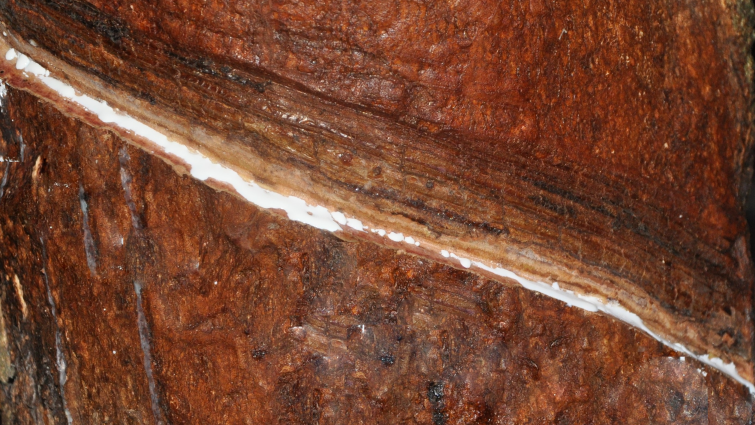

Supplement: S5 Data — (ZIP) [file pone.0297284.s005.zip › Level 3 Original Sample/3-2.png]

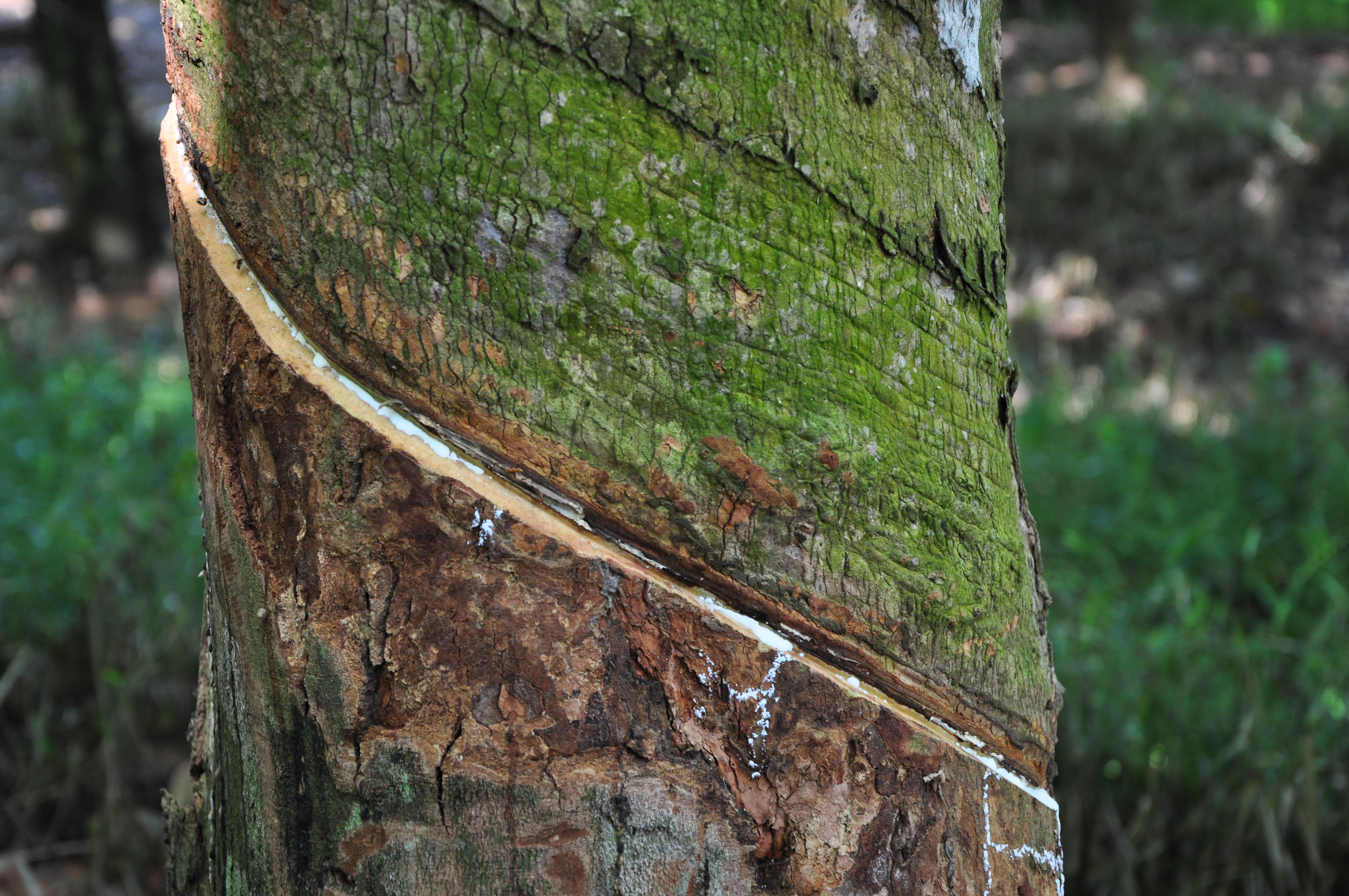

Supplement: S5 Data — (ZIP) [file pone.0297284.s005.zip › Level 3 Original Sample/3-60101-119-20140708-0260.JPG]

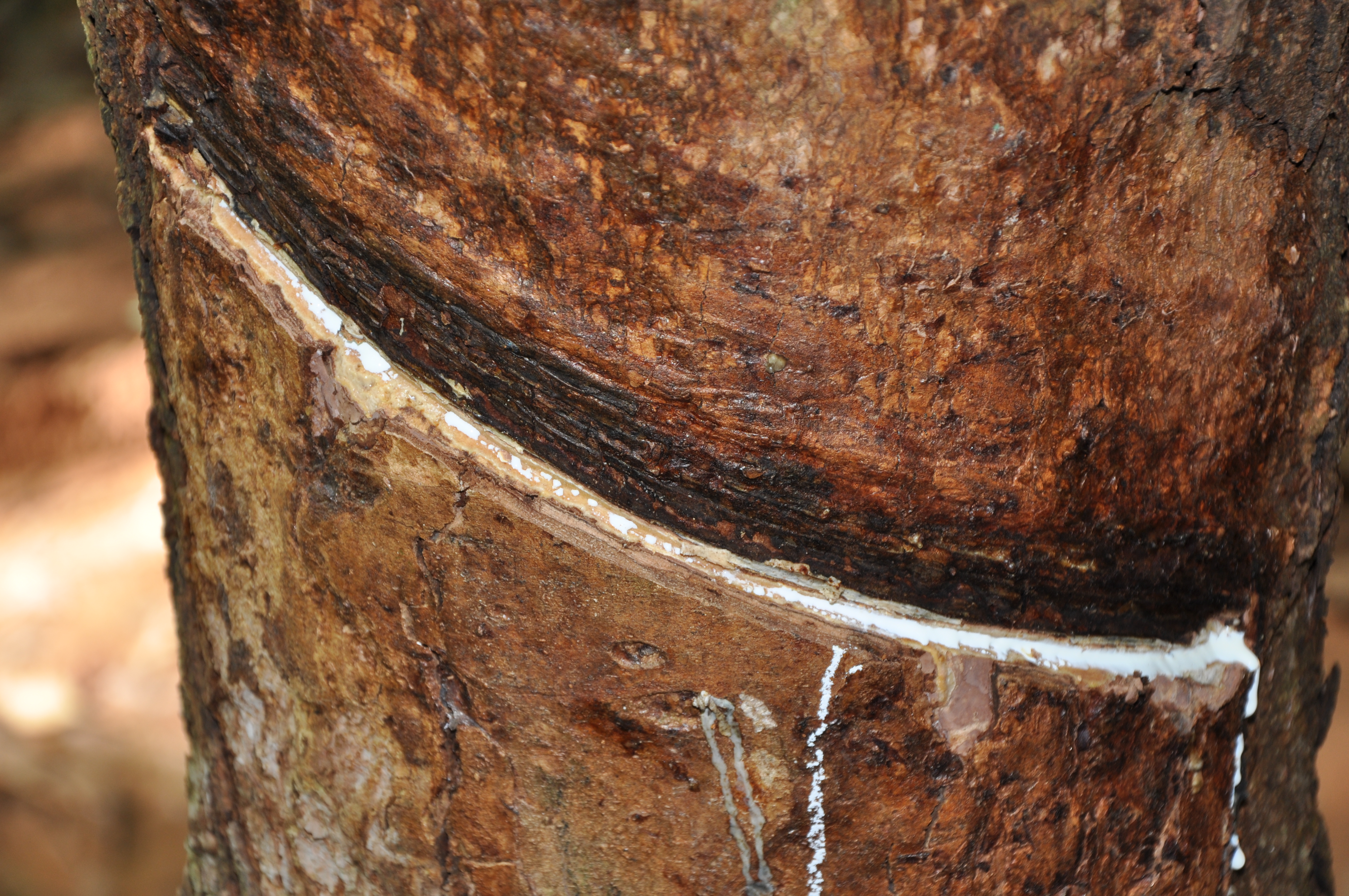

Supplement: S5 Data — (ZIP) [file pone.0297284.s005.zip › Level 3 Original Sample/3-60603-286-20140708-0184.JPG]

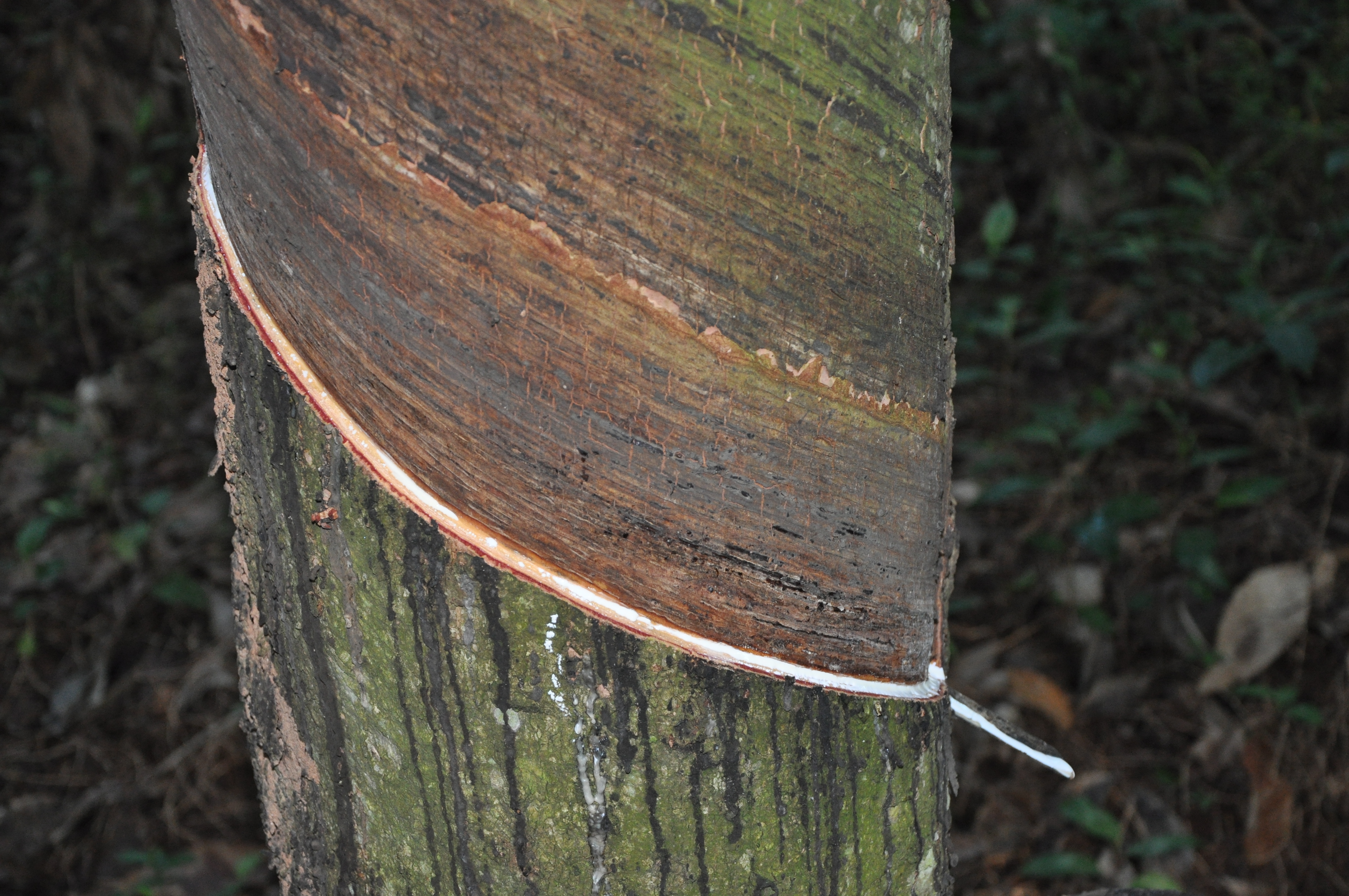

Supplement: S5 Data — (ZIP) [file pone.0297284.s005.zip › Level 3 Original Sample/3-61001-219-20140902-0072.JPG]

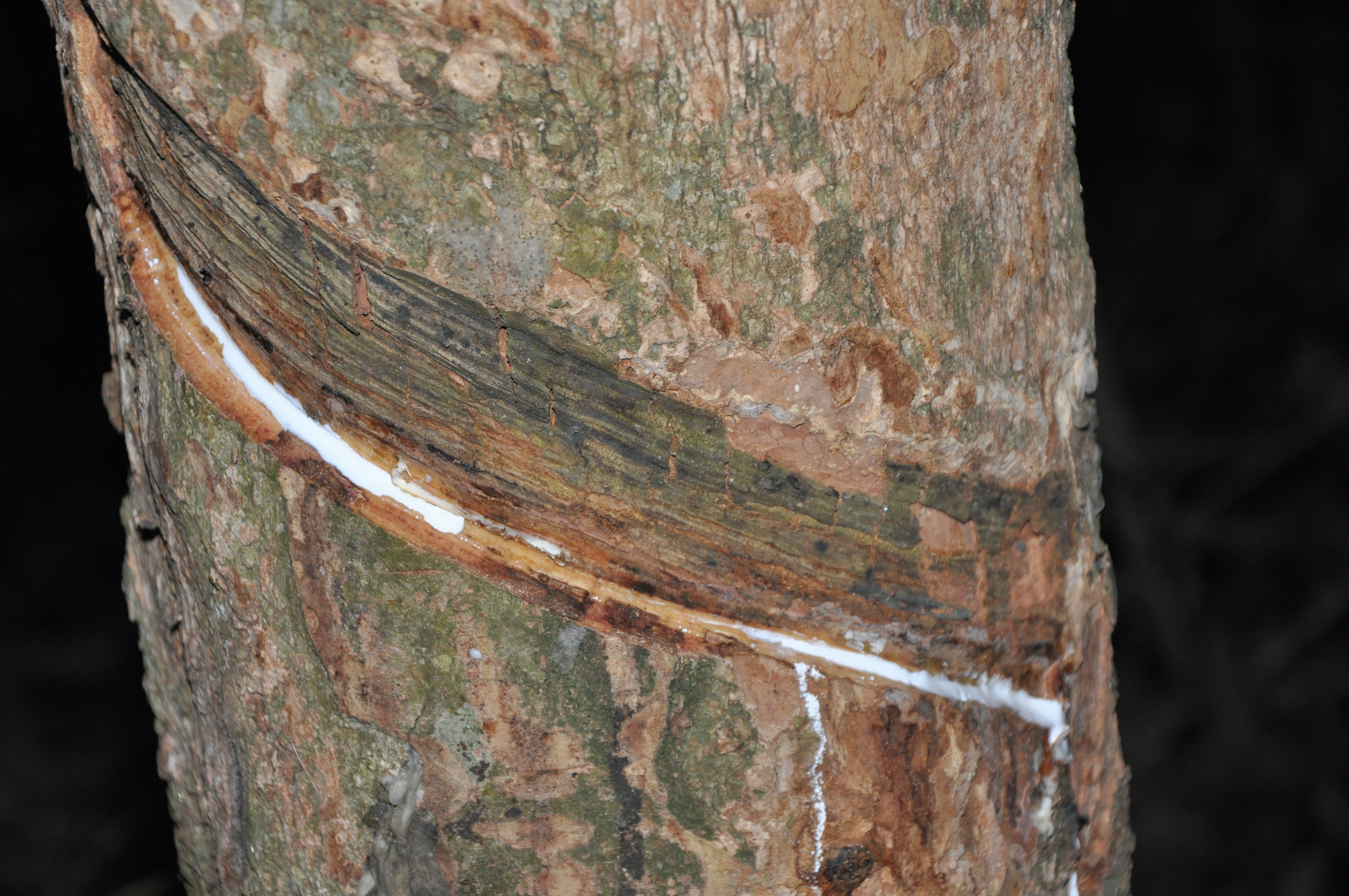

Supplement: S5 Data — (ZIP) [file pone.0297284.s005.zip › Level 3 Original Sample/3-61602-182-20150423-0042.JPG]

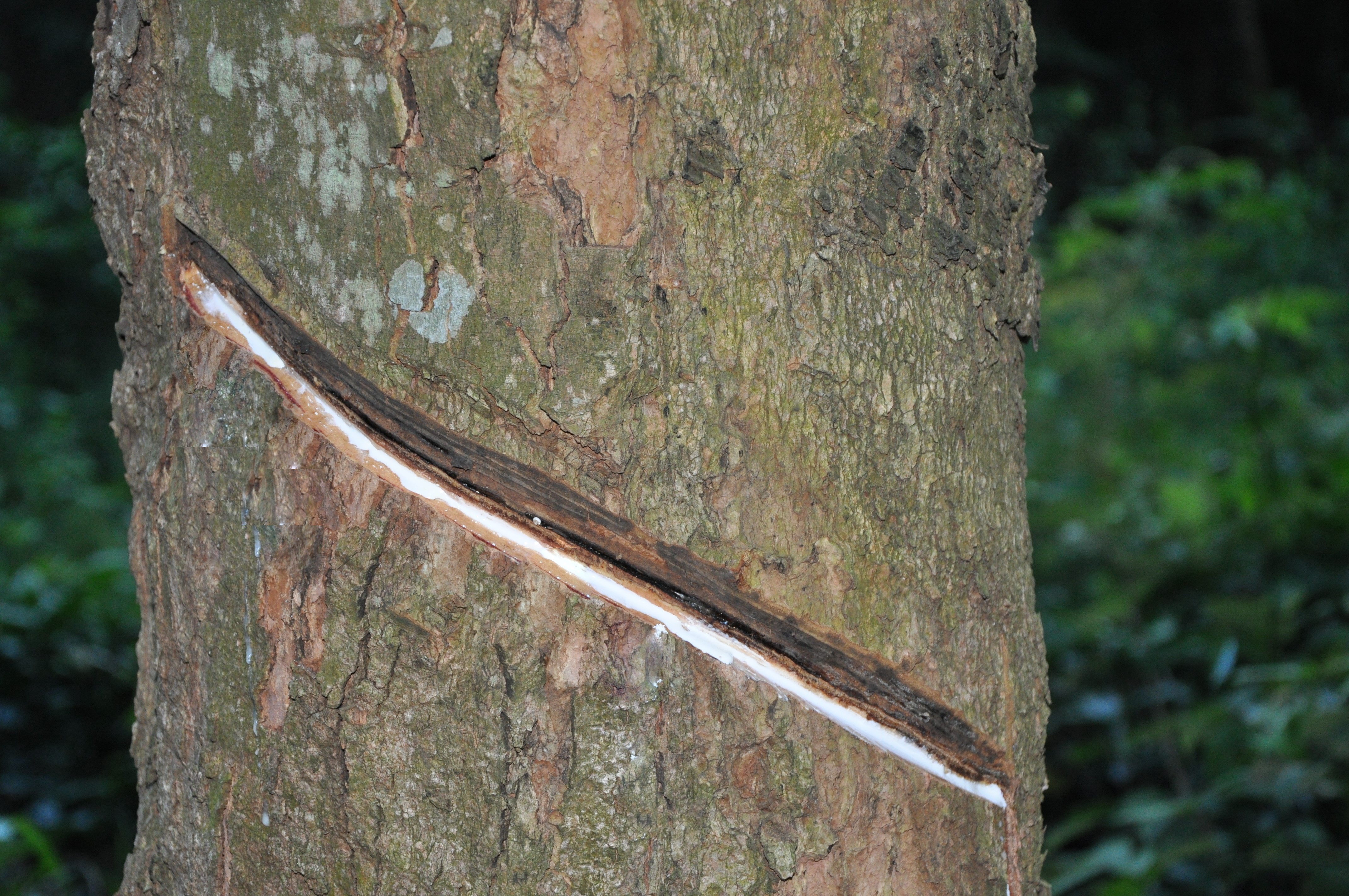

Supplement: S5 Data — (ZIP) [file pone.0297284.s005.zip › Level 3 Original Sample/3-62001-147-20140827-0053.JPG]

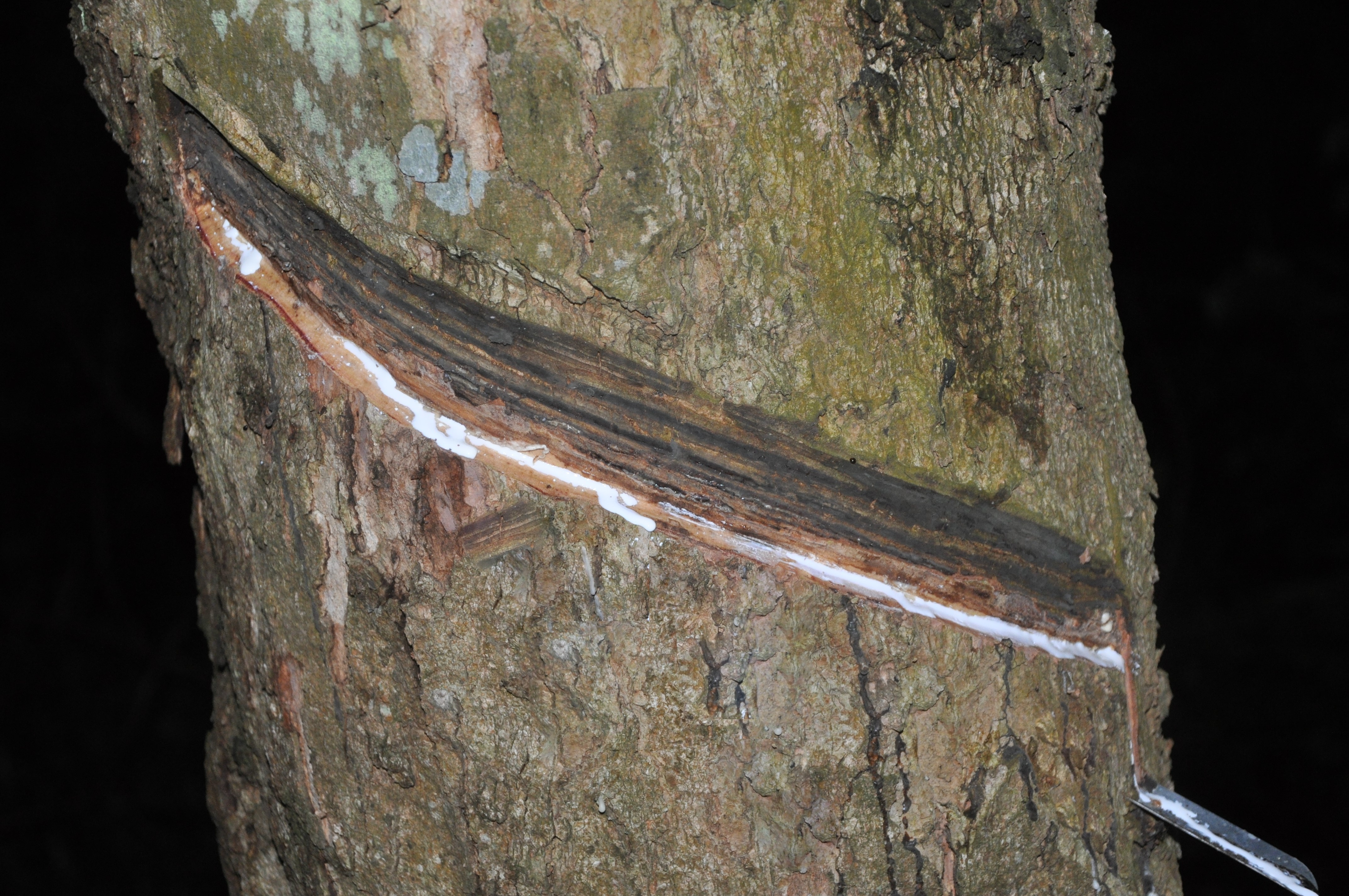

Supplement: S5 Data — (ZIP) [file pone.0297284.s005.zip › Level 3 Original Sample/3-62001-147-20150424-0190.JPG]

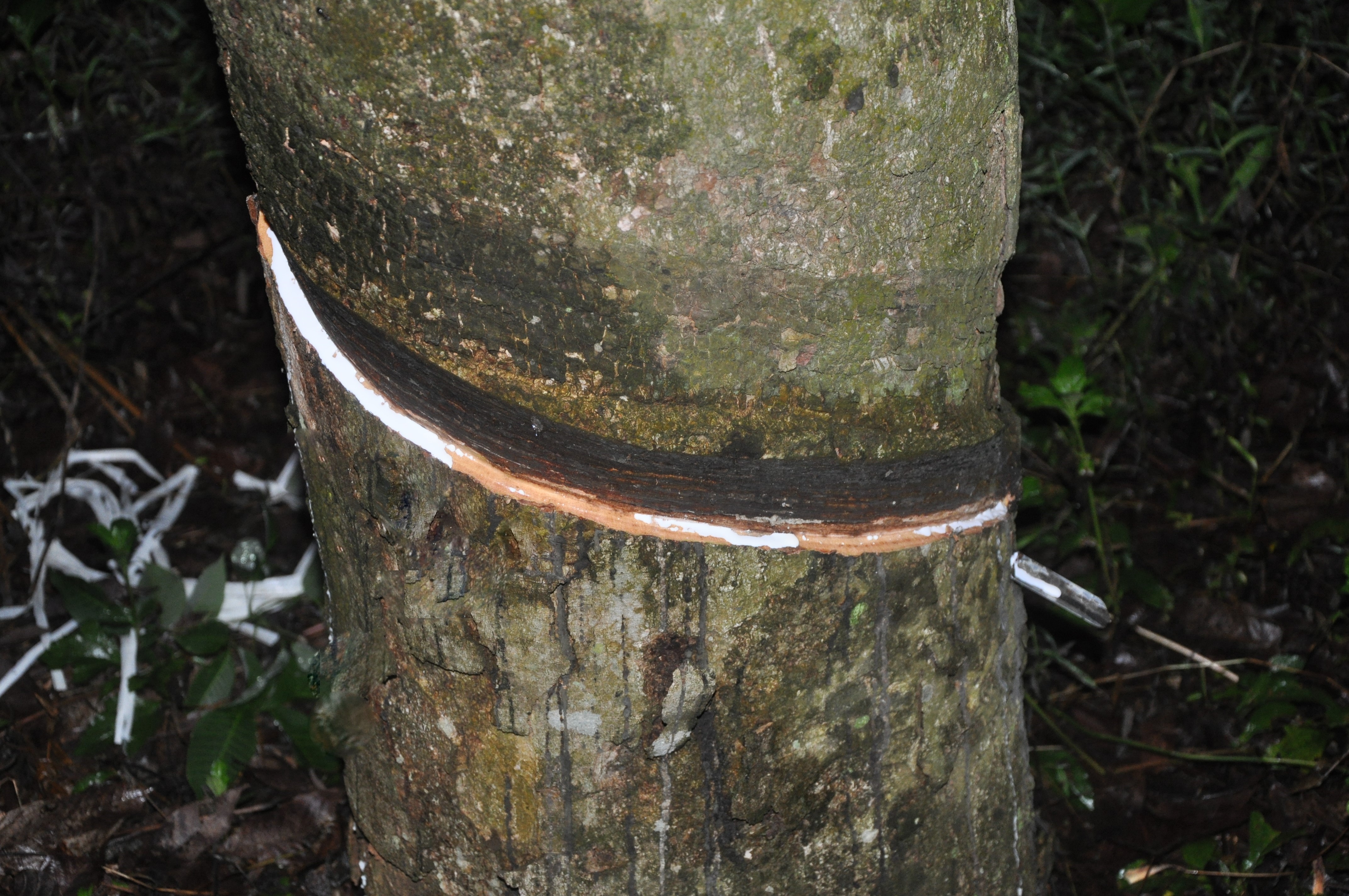

Supplement: S5 Data — (ZIP) [file pone.0297284.s005.zip › Level 3 Original Sample/3-62001-148-20150424-0188.JPG]

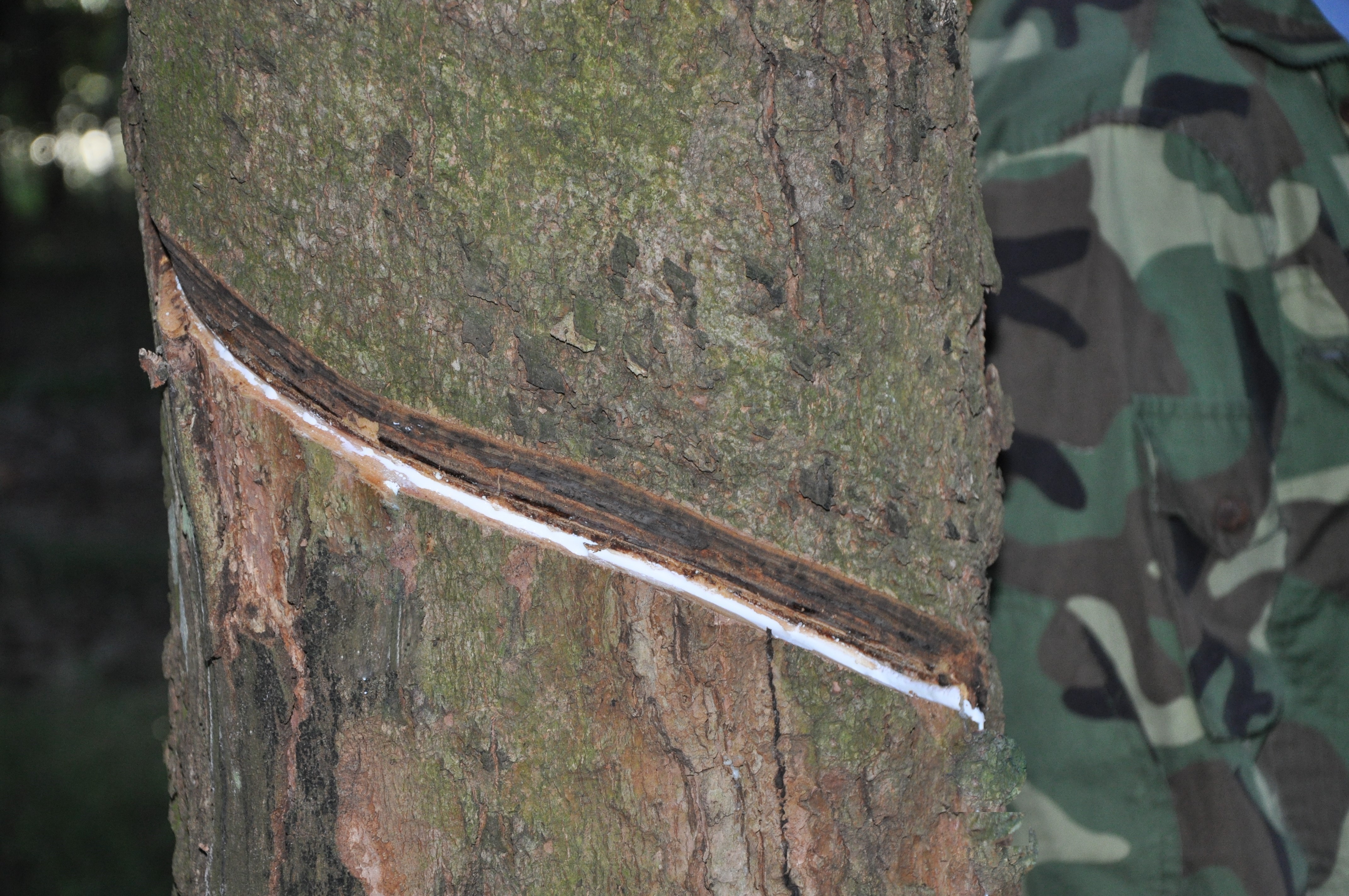

Supplement: S5 Data — (ZIP) [file pone.0297284.s005.zip › Level 3 Original Sample/3-62001-149-20140827-0049.JPG]

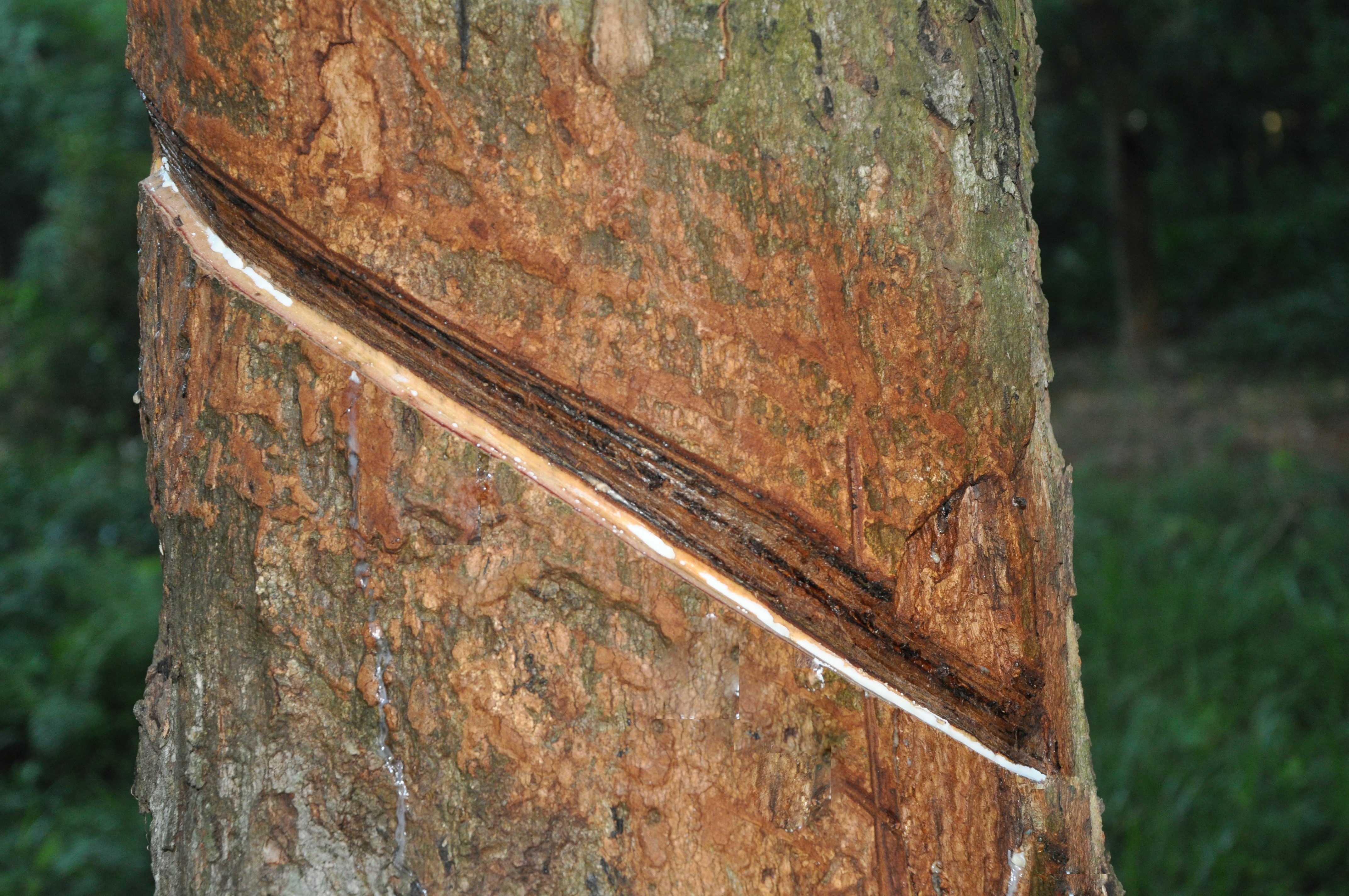

Supplement: S5 Data — (ZIP) [file pone.0297284.s005.zip › Level 3 Original Sample/3-62001-233-20140827-0076.JPG]

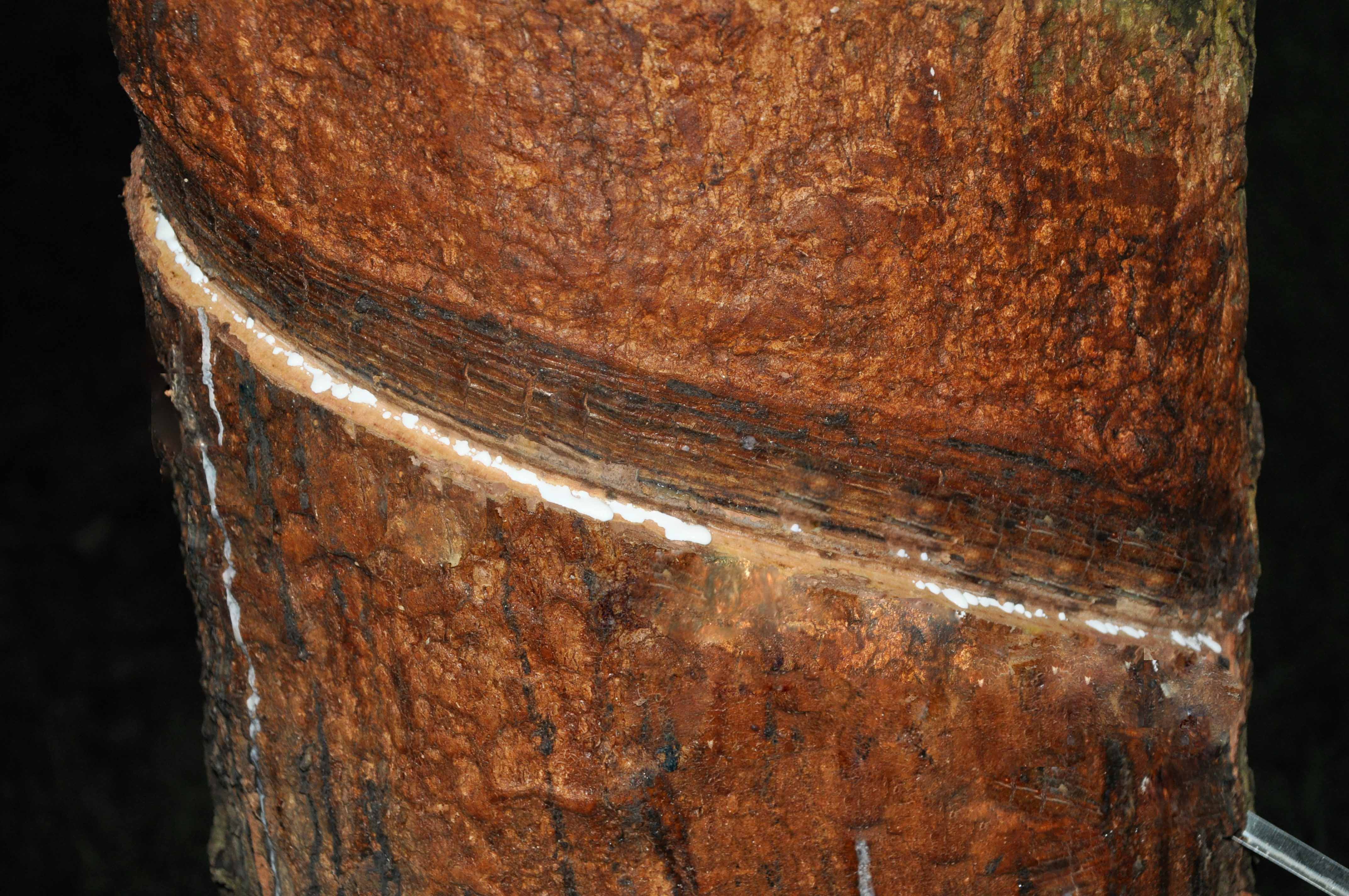

Supplement: S5 Data — (ZIP) [file pone.0297284.s005.zip › Level 3 Original Sample/3-62001-258-20150424-0241.JPG]

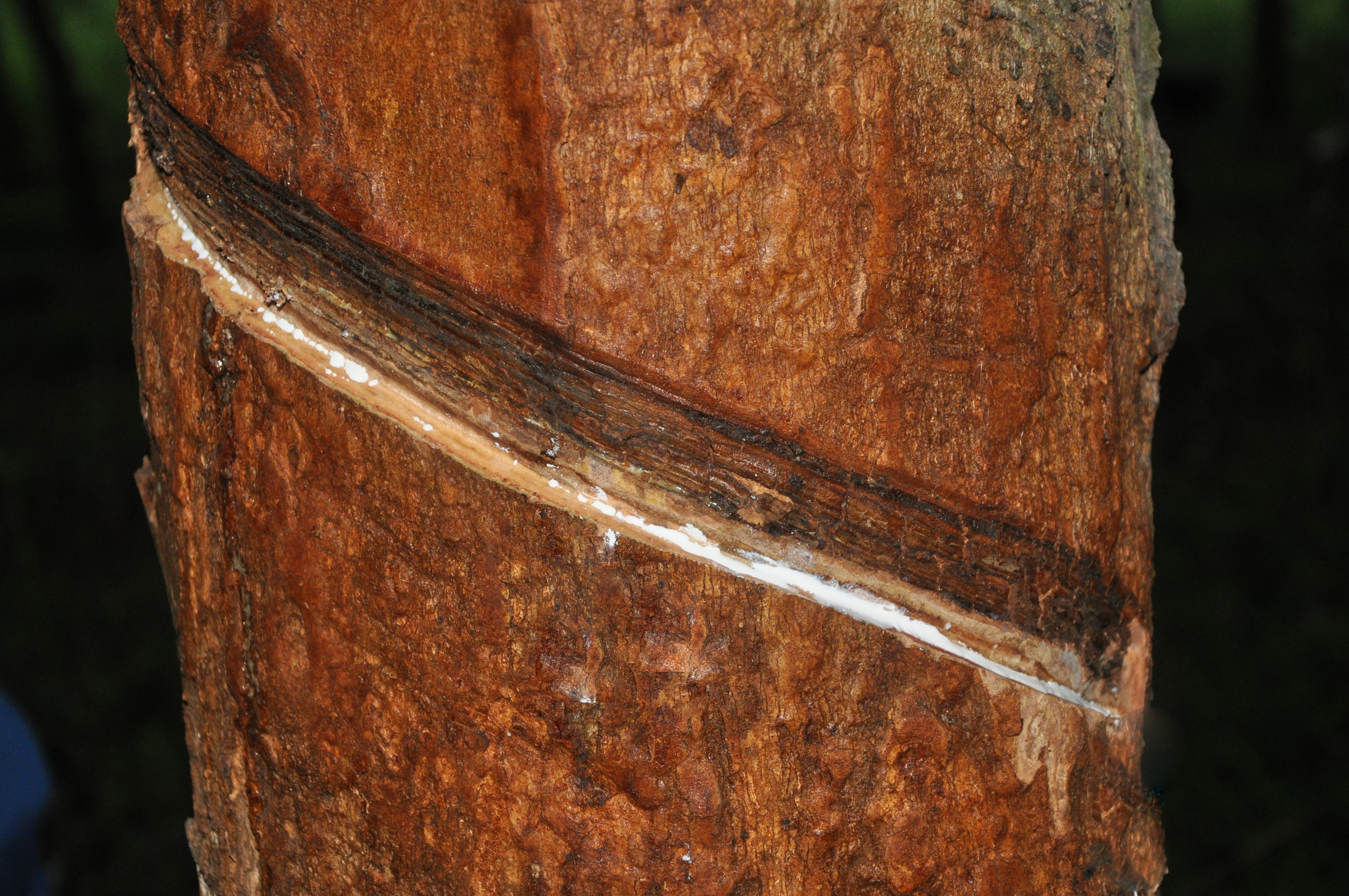

Supplement: S5 Data — (ZIP) [file pone.0297284.s005.zip › Level 3 Original Sample/3-62001-294-20150424-0308.JPG]

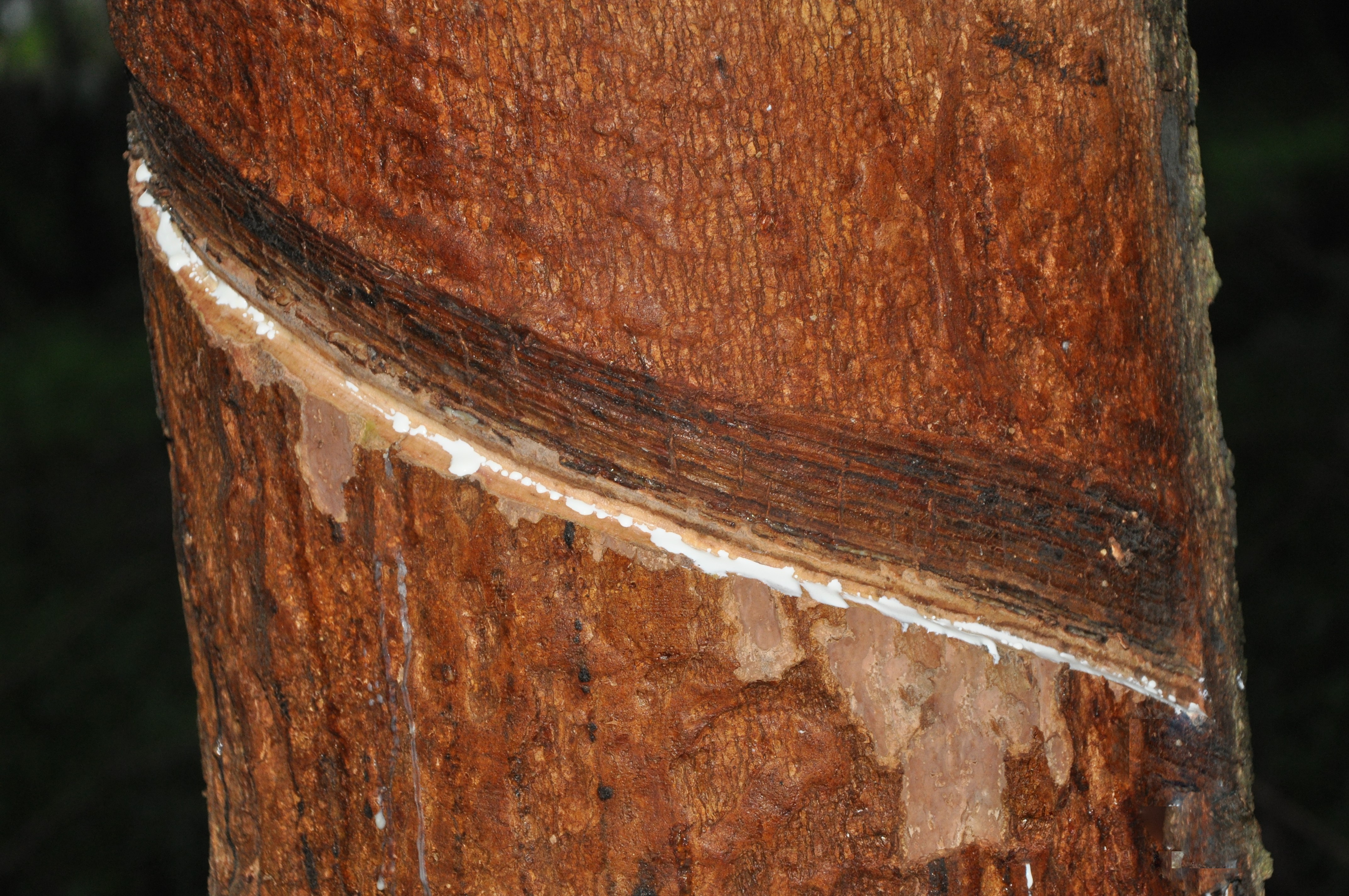

Supplement: S5 Data — (ZIP) [file pone.0297284.s005.zip › Level 3 Original Sample/3-62001-300-20150424-0302.JPG]

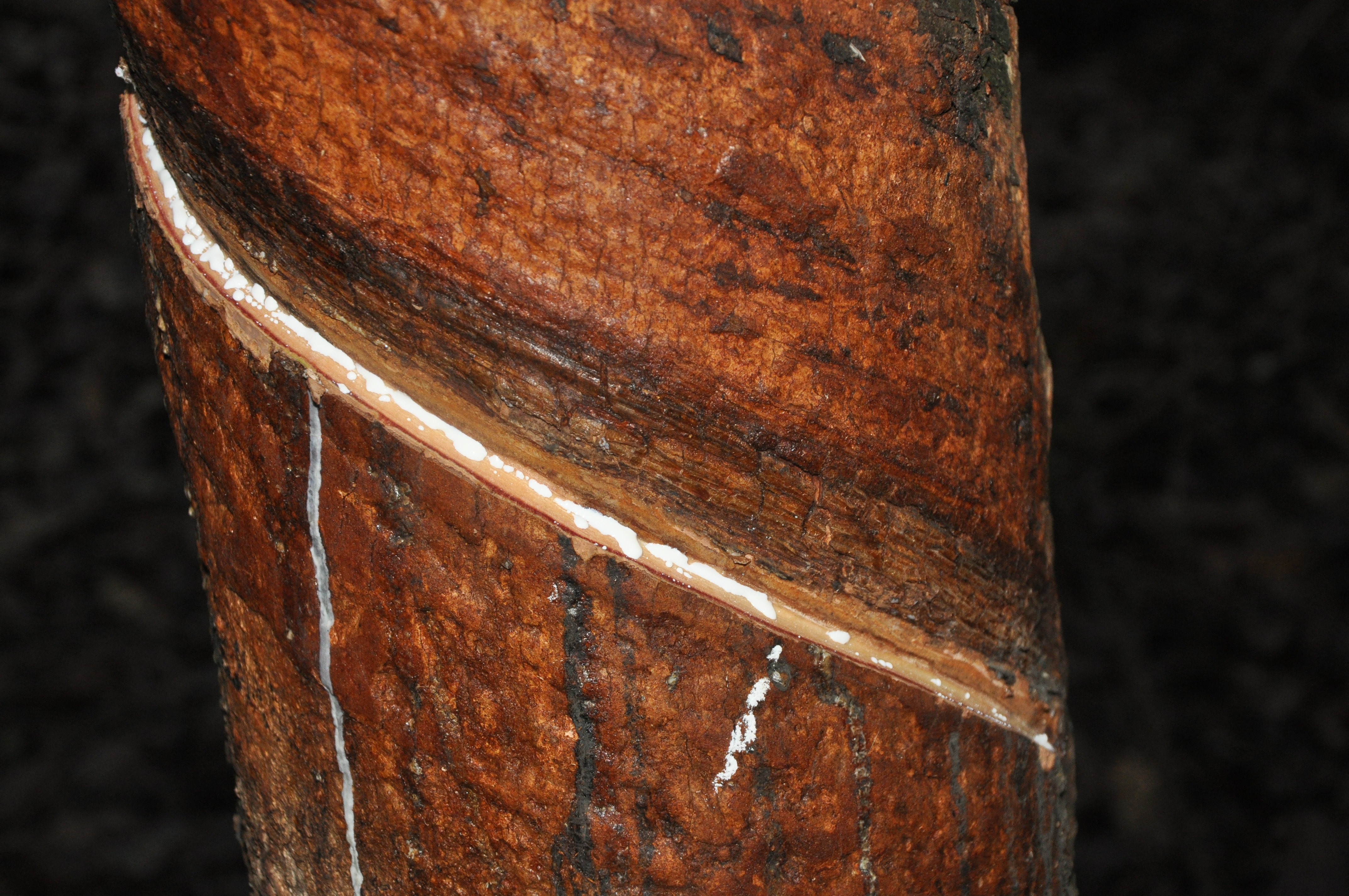

Supplement: S5 Data — (ZIP) [file pone.0297284.s005.zip › Level 3 Original Sample/3-62001-384-20150424-0288.JPG]

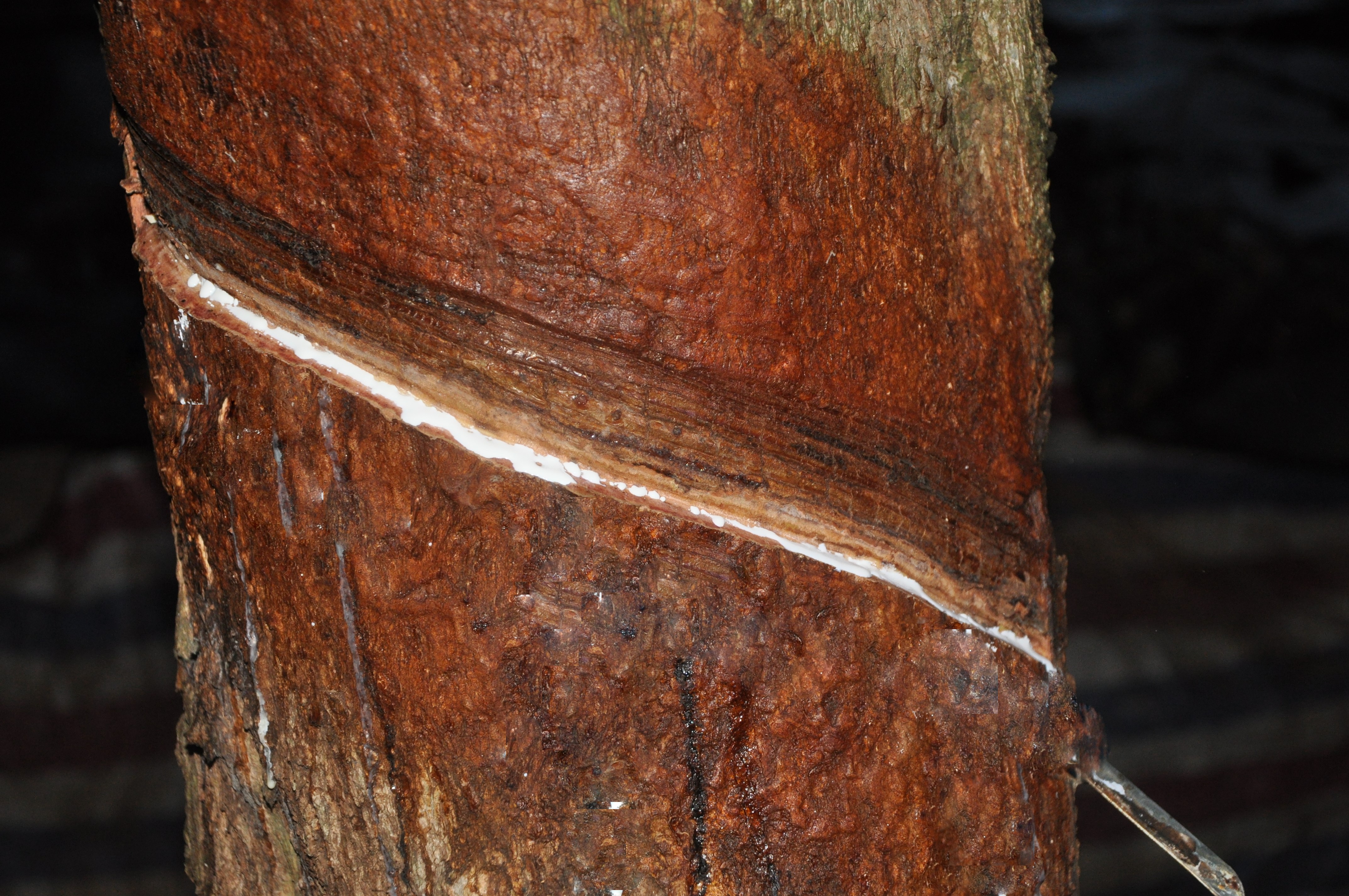

Supplement: S5 Data — (ZIP) [file pone.0297284.s005.zip › Level 3 Original Sample/3-62001-396-20150424-0223.JPG]

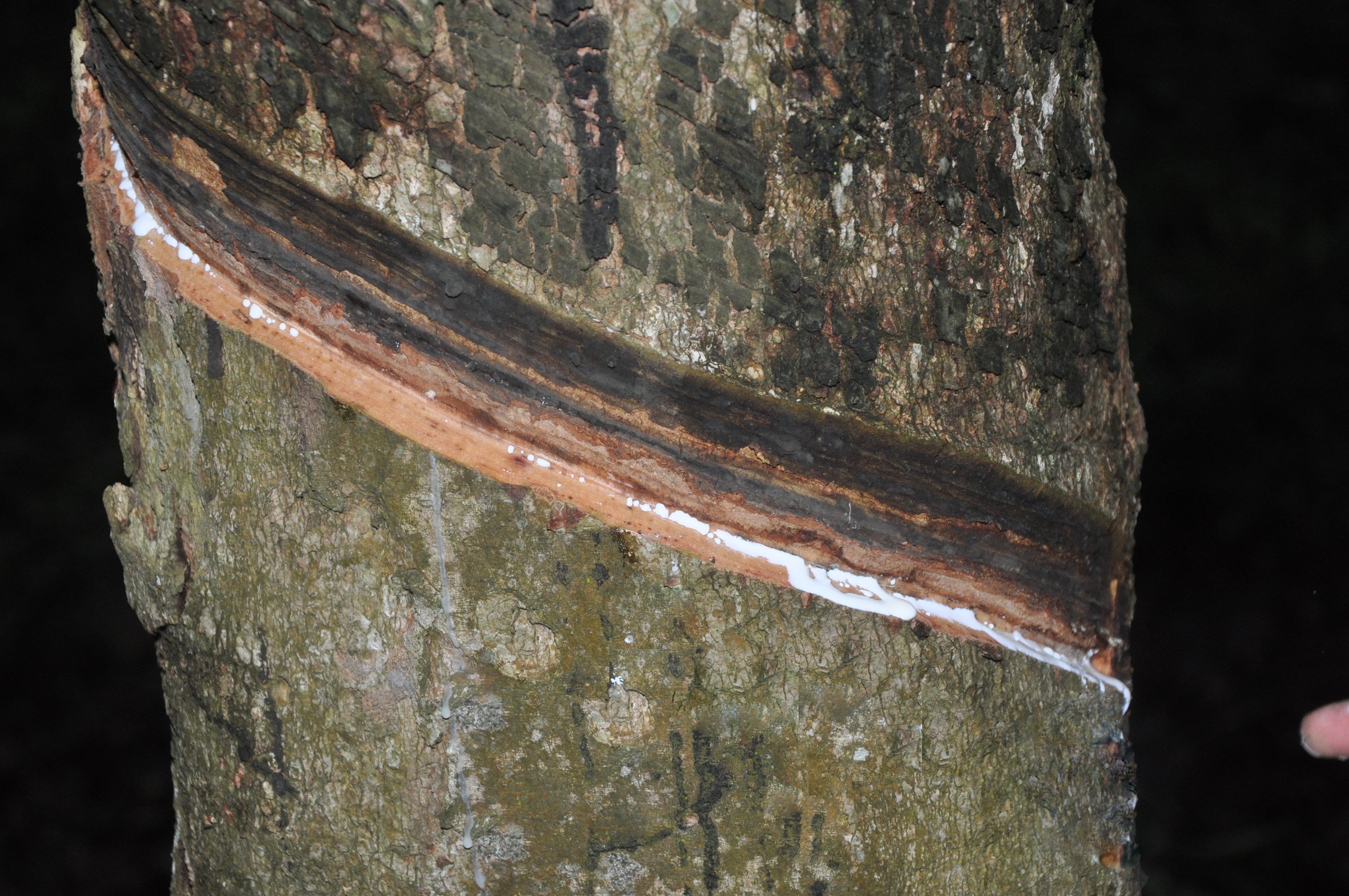

Supplement: S5 Data — (ZIP) [file pone.0297284.s005.zip › Level 3 Original Sample/3-62001-436-20150424-0276.JPG]

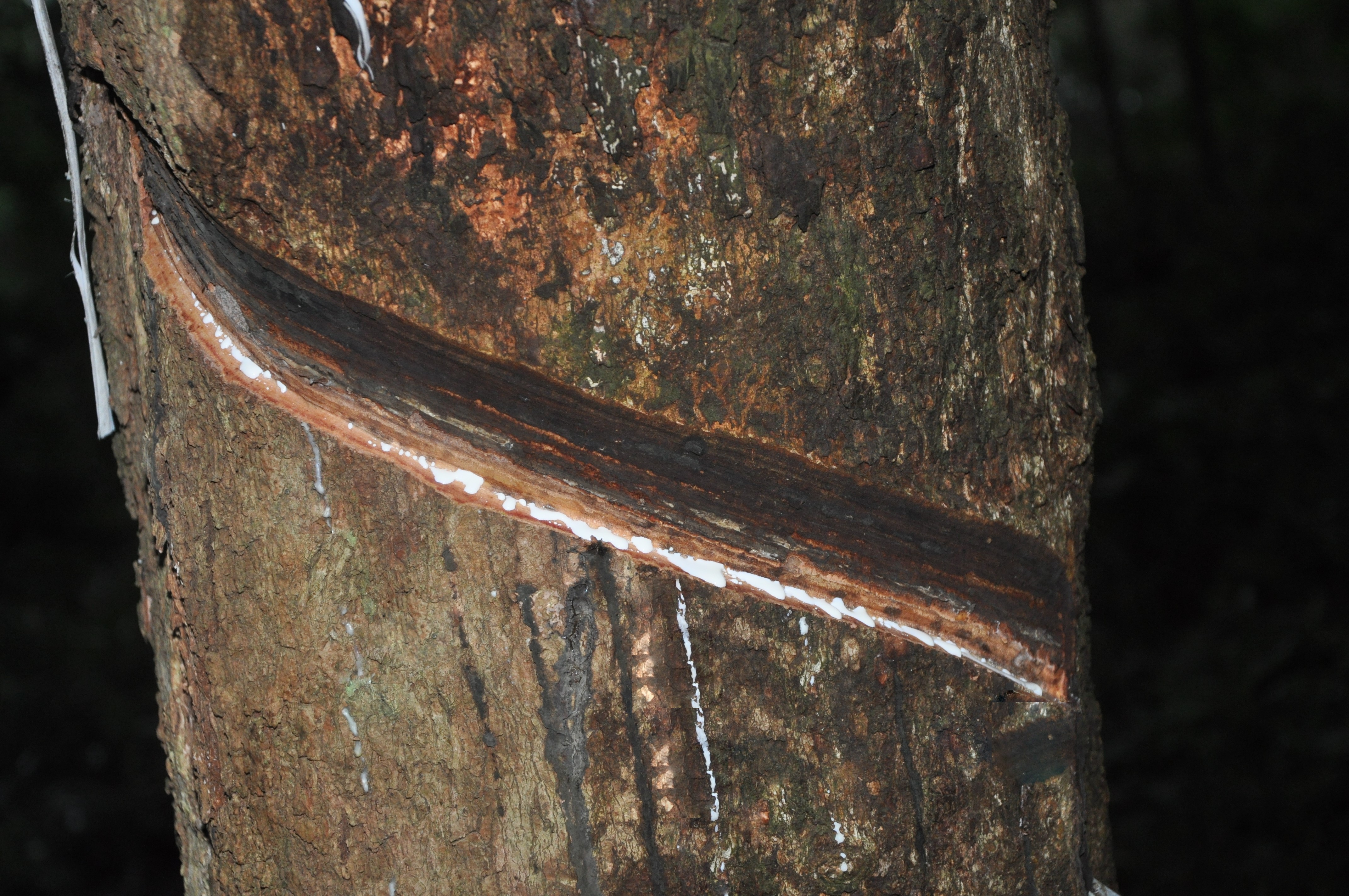

Supplement: S5 Data — (ZIP) [file pone.0297284.s005.zip › Level 3 Original Sample/3-62001-457-20150424-0280.JPG]

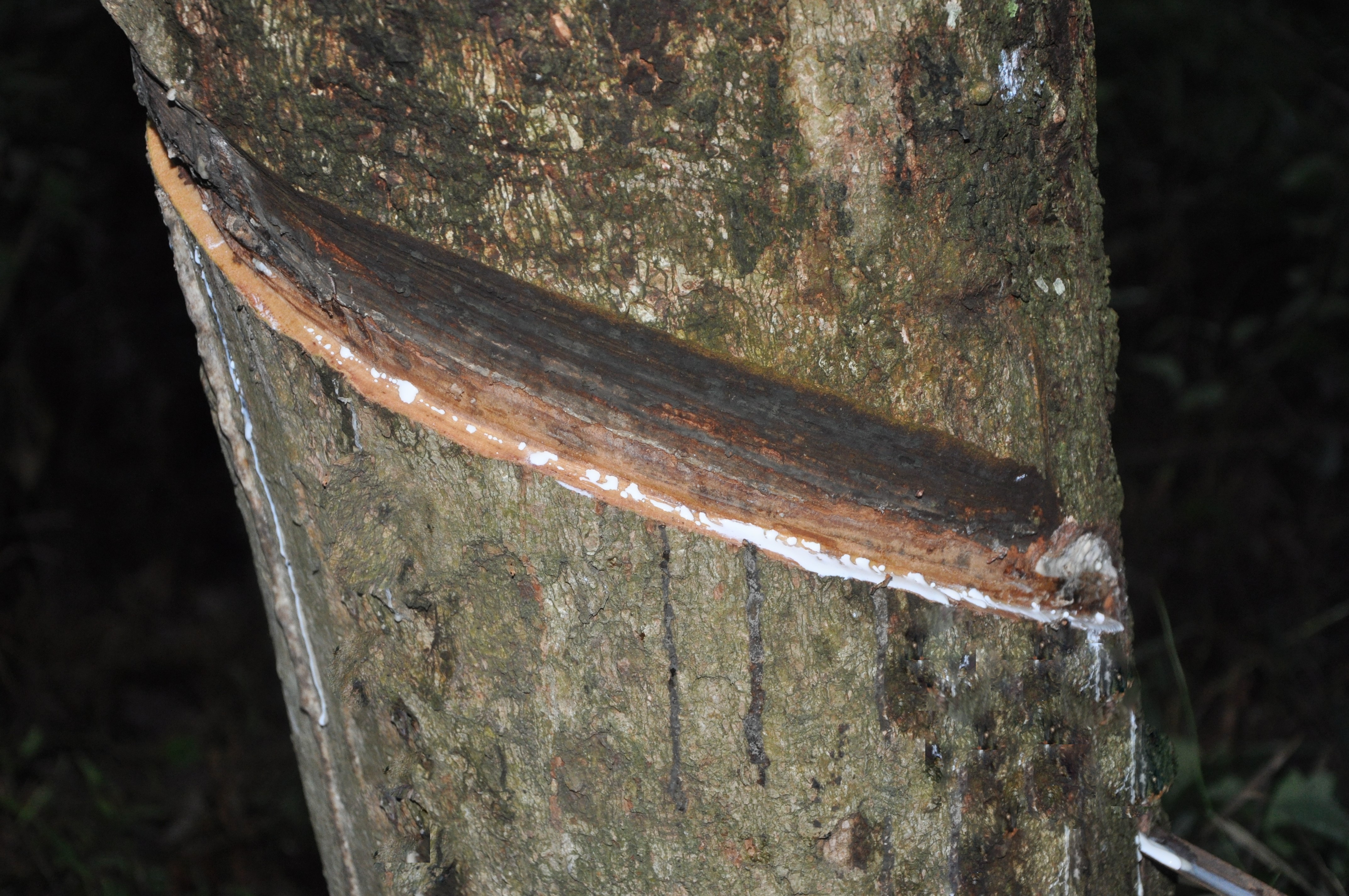

Supplement: S5 Data — (ZIP) [file pone.0297284.s005.zip › Level 3 Original Sample/3-62001-465-20150424-0272.JPG]

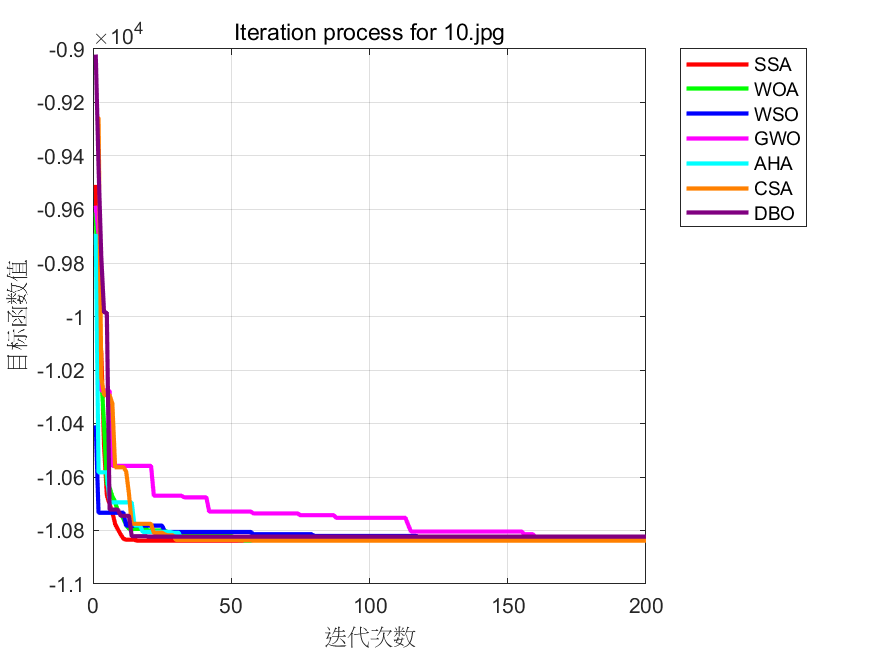

Supplement: S6 Data — (ZIP) [file pone.0297284.s006.zip › Level 3 processed Sample/iteration/10.jpg_iteration.png]

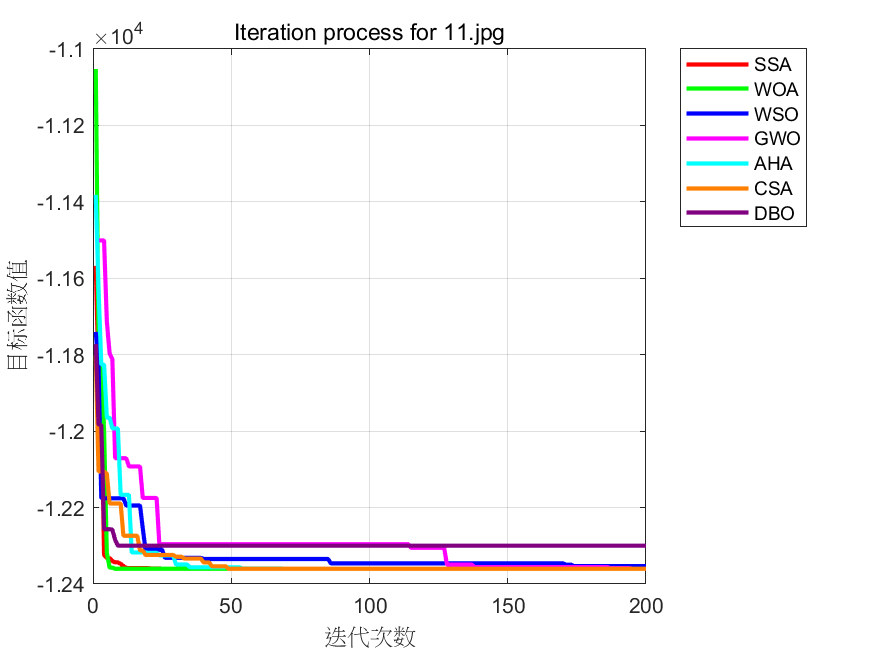

Supplement: S6 Data — (ZIP) [file pone.0297284.s006.zip › Level 3 processed Sample/iteration/11.jpg_iteration.png]

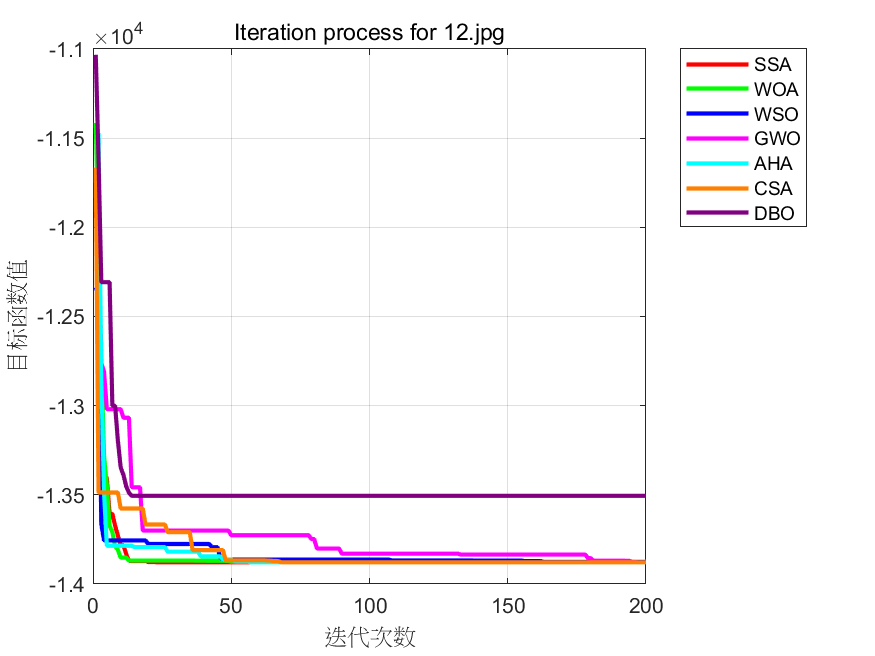

Supplement: S6 Data — (ZIP) [file pone.0297284.s006.zip › Level 3 processed Sample/iteration/12.jpg_iteration.png]

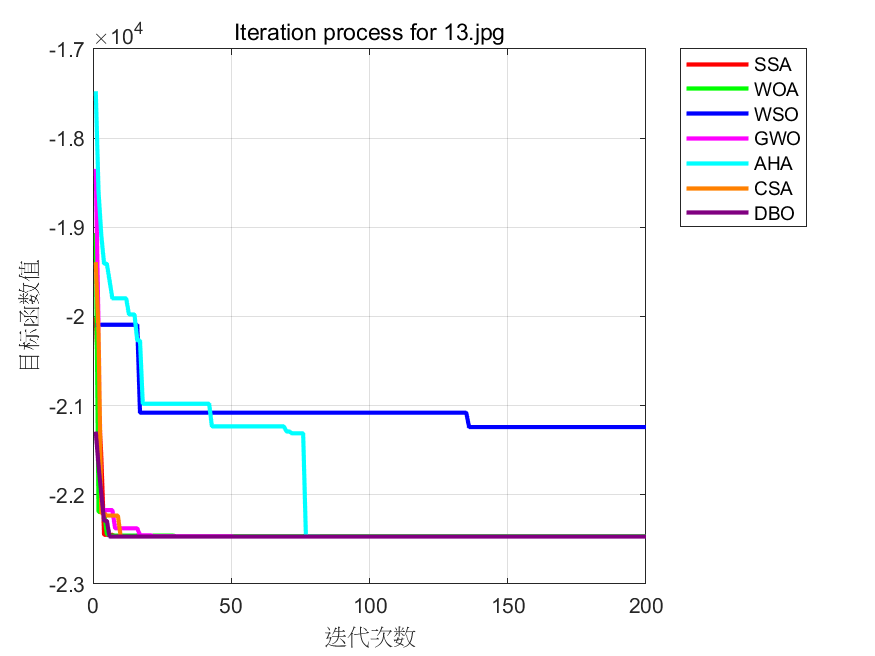

Supplement: S6 Data — (ZIP) [file pone.0297284.s006.zip › Level 3 processed Sample/iteration/13.jpg_iteration.png]

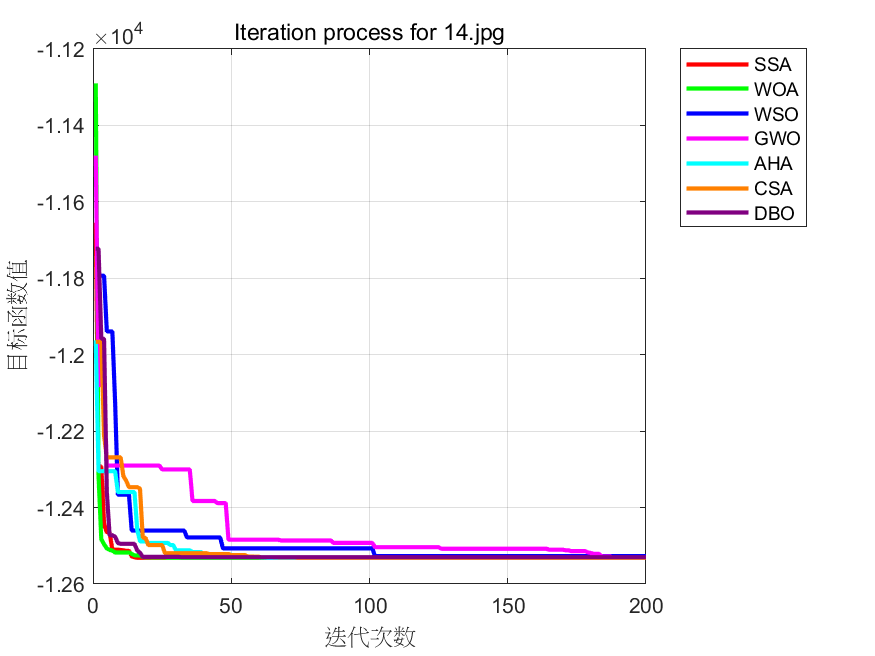

Supplement: S6 Data — (ZIP) [file pone.0297284.s006.zip › Level 3 processed Sample/iteration/14.jpg_iteration.png]

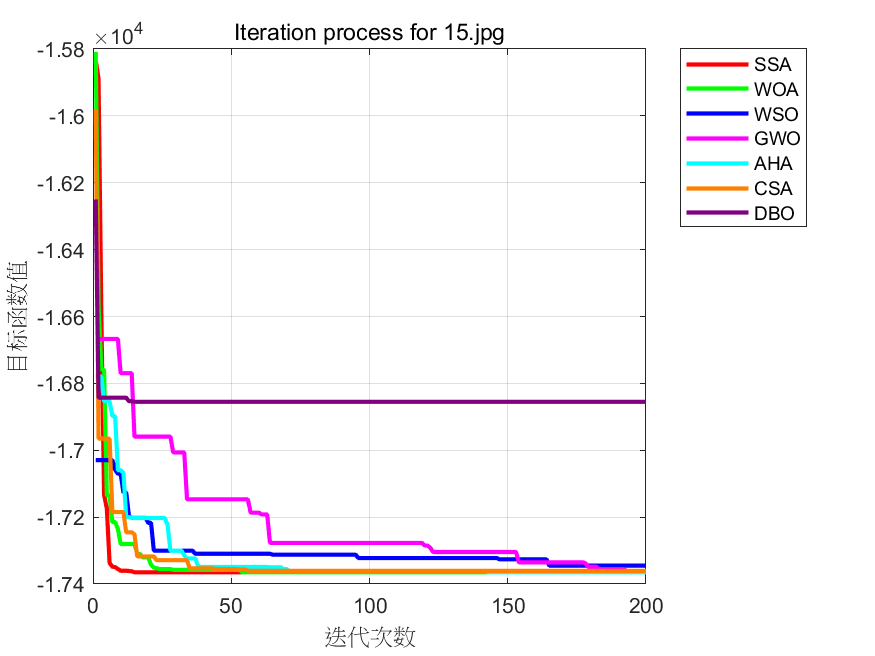

Supplement: S6 Data — (ZIP) [file pone.0297284.s006.zip › Level 3 processed Sample/iteration/15.jpg_iteration.png]

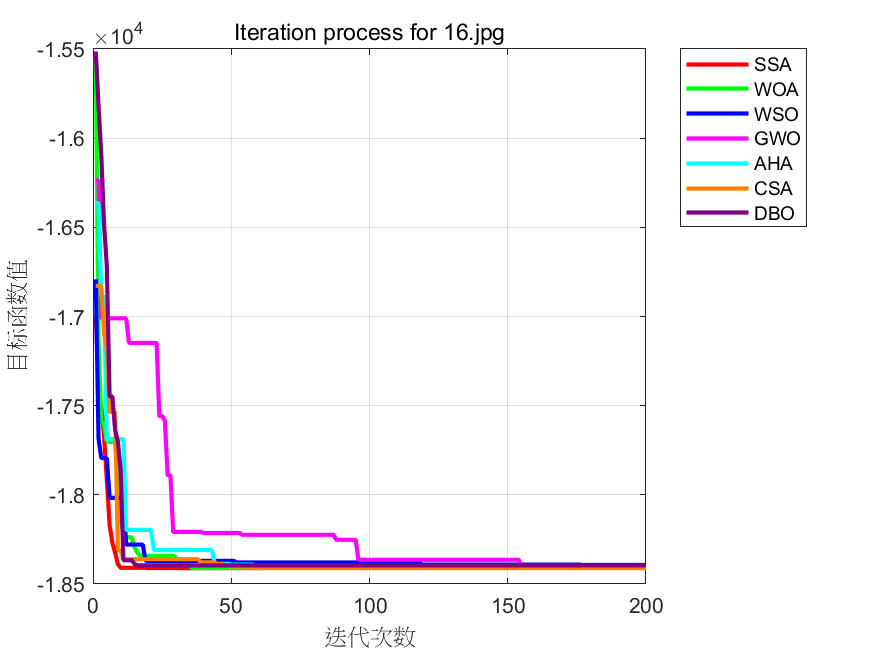

Supplement: S6 Data — (ZIP) [file pone.0297284.s006.zip › Level 3 processed Sample/iteration/16.jpg_iteration.png]

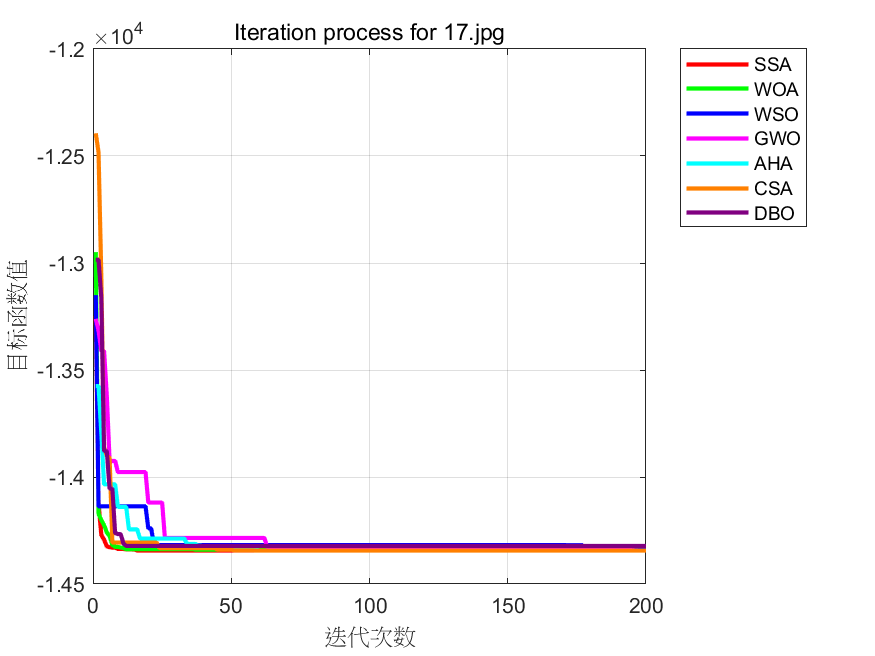

Supplement: S6 Data — (ZIP) [file pone.0297284.s006.zip › Level 3 processed Sample/iteration/17.jpg_iteration.png]

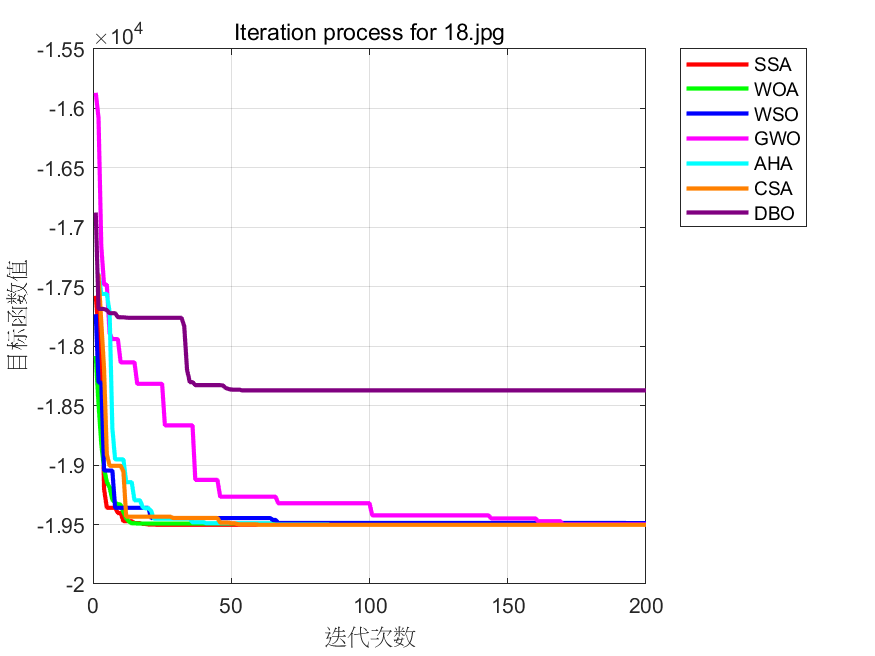

Supplement: S6 Data — (ZIP) [file pone.0297284.s006.zip › Level 3 processed Sample/iteration/18.jpg_iteration.png]

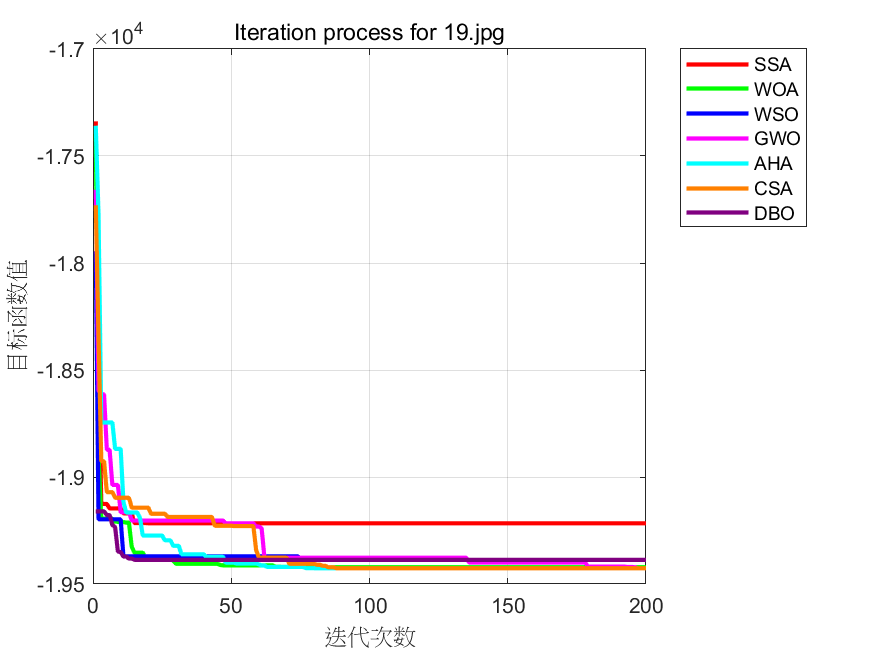

Supplement: S6 Data — (ZIP) [file pone.0297284.s006.zip › Level 3 processed Sample/iteration/19.jpg_iteration.png]

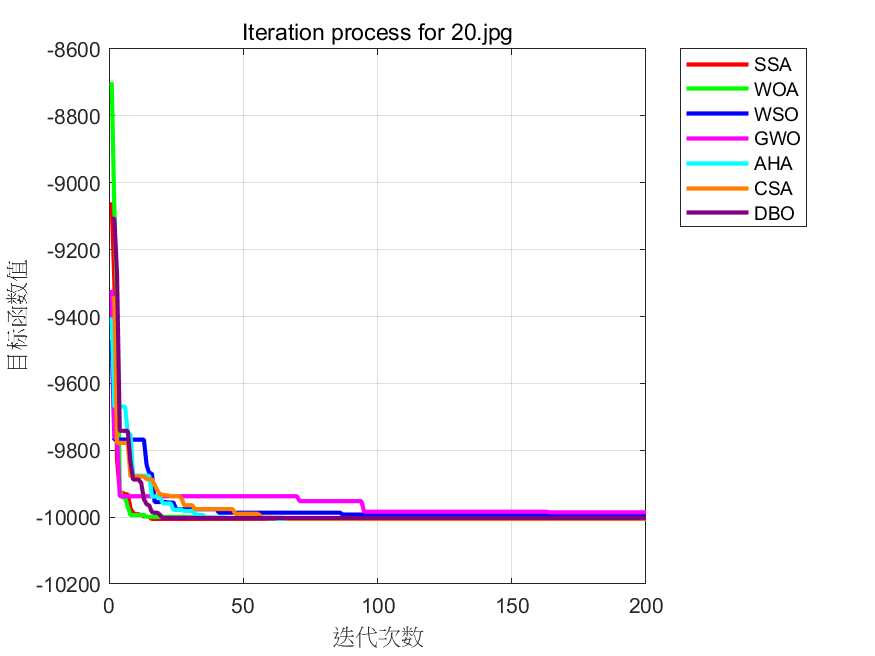

Supplement: S6 Data — (ZIP) [file pone.0297284.s006.zip › Level 3 processed Sample/iteration/20.jpg_iteration.png]
