# Supplementary figures and images for: Advancing image segmentation with DBO-Otsu: Addressing rubber tree diseases through enhanced threshold techniques (part 4 of 7)
Source: PLoS One. 2024 Mar 21;19(3):e0297284. doi: 10.1371/journal.pone.0297284 (PMC10956860; doi:10.1371/journal.pone.0297284)

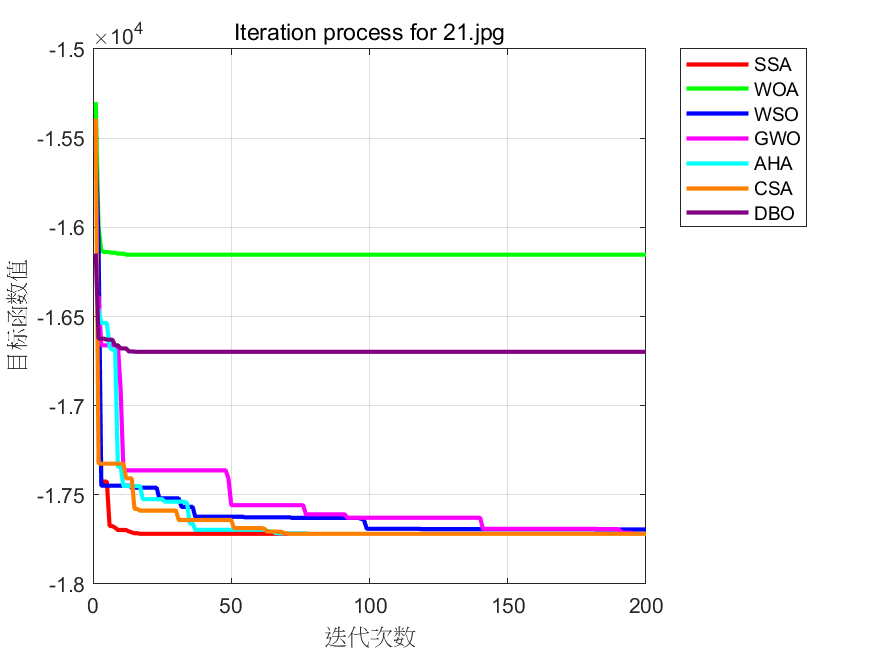

Supplement: S6 Data — (ZIP) [file pone.0297284.s006.zip › Level 3 processed Sample/iteration/21.jpg_iteration.png]

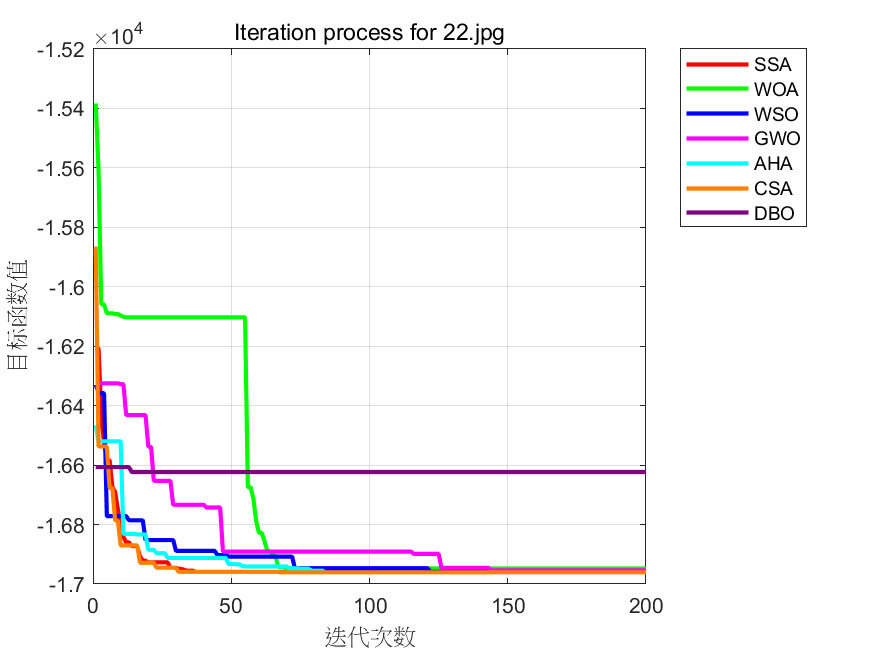

Supplement: S6 Data — (ZIP) [file pone.0297284.s006.zip › Level 3 processed Sample/iteration/22.jpg_iteration.png]

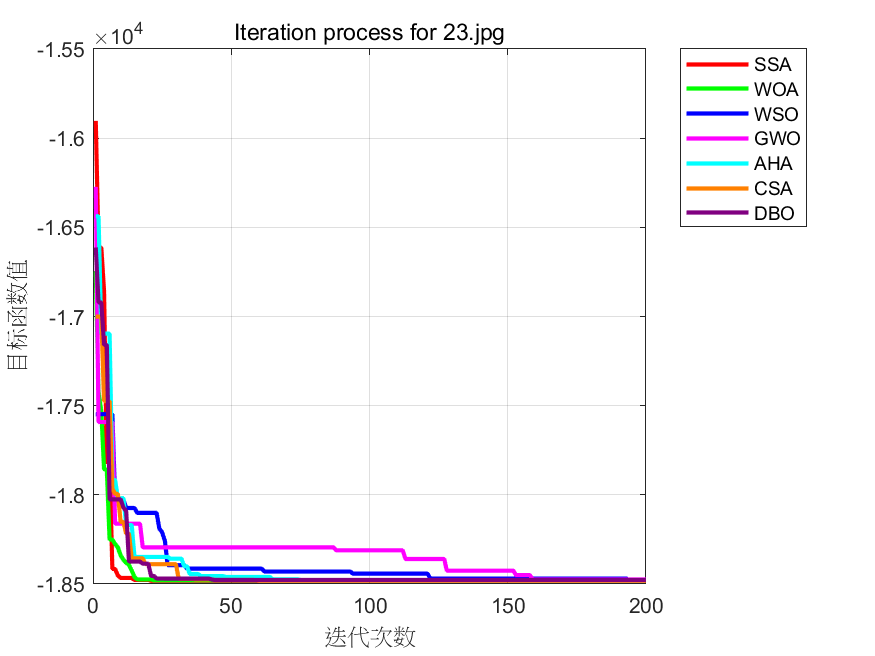

Supplement: S6 Data — (ZIP) [file pone.0297284.s006.zip › Level 3 processed Sample/iteration/23.jpg_iteration.png]

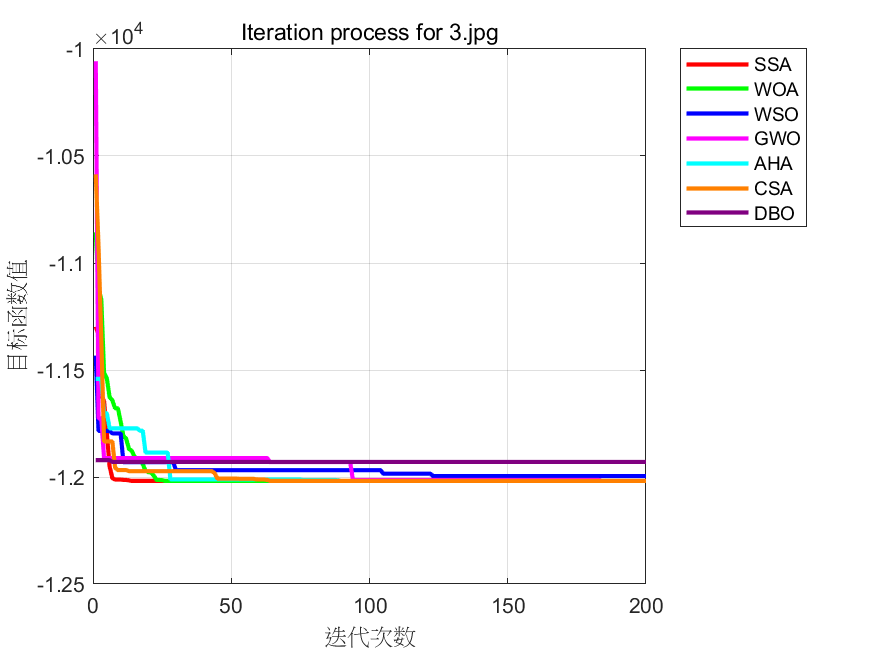

Supplement: S6 Data — (ZIP) [file pone.0297284.s006.zip › Level 3 processed Sample/iteration/3.jpg_iteration.png]

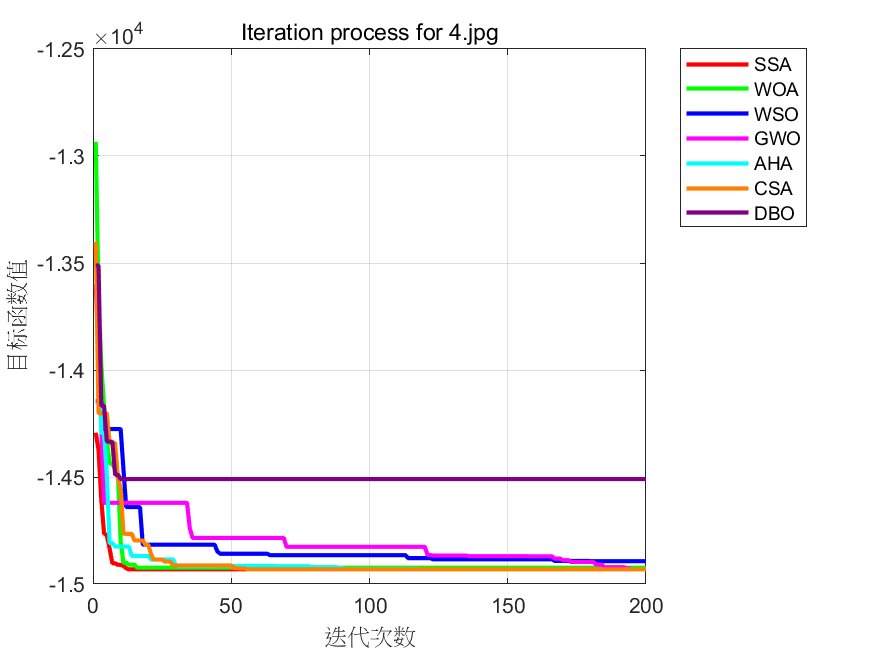

Supplement: S6 Data — (ZIP) [file pone.0297284.s006.zip › Level 3 processed Sample/iteration/4.jpg_iteration.png]

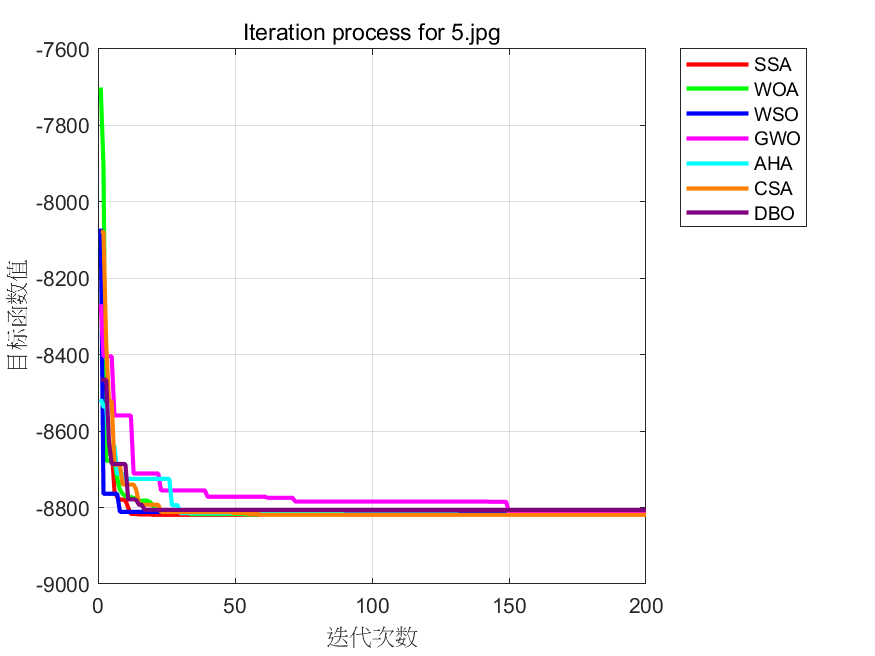

Supplement: S6 Data — (ZIP) [file pone.0297284.s006.zip › Level 3 processed Sample/iteration/5.jpg_iteration.png]

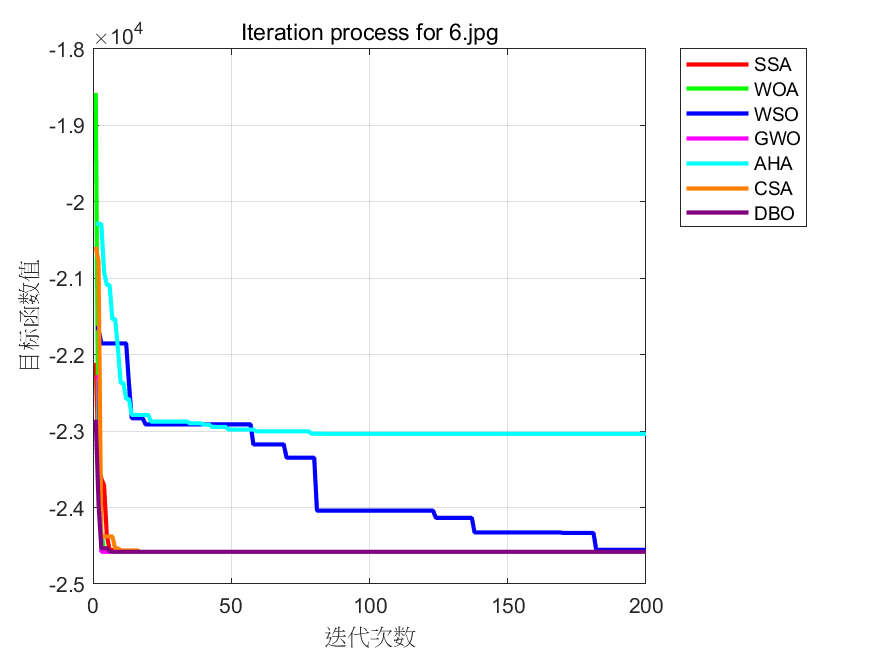

Supplement: S6 Data — (ZIP) [file pone.0297284.s006.zip › Level 3 processed Sample/iteration/6.jpg_iteration.png]

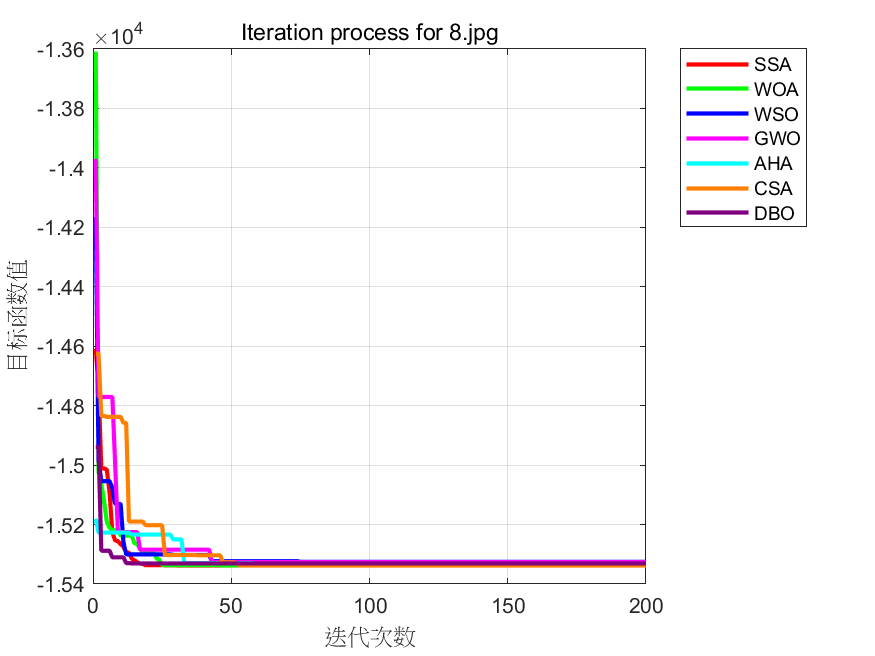

Supplement: S6 Data — (ZIP) [file pone.0297284.s006.zip › Level 3 processed Sample/iteration/8.jpg_iteration.png]

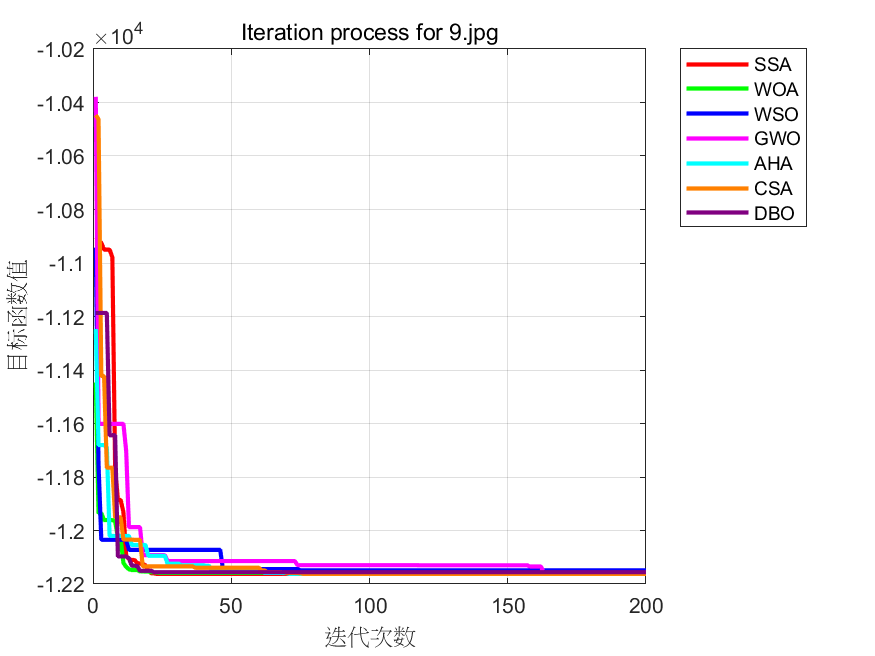

Supplement: S6 Data — (ZIP) [file pone.0297284.s006.zip › Level 3 processed Sample/iteration/9.jpg_iteration.png]

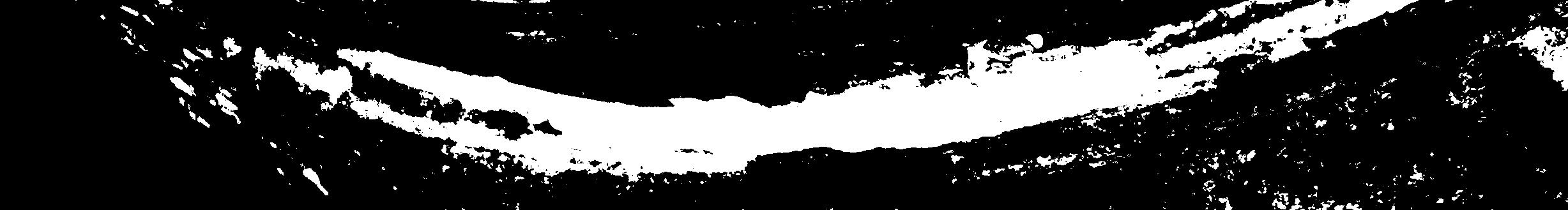

Supplement: S6 Data — (ZIP) [file pone.0297284.s006.zip › Level 3 processed Sample/processed_10/latex/AHA_latex.jpg]

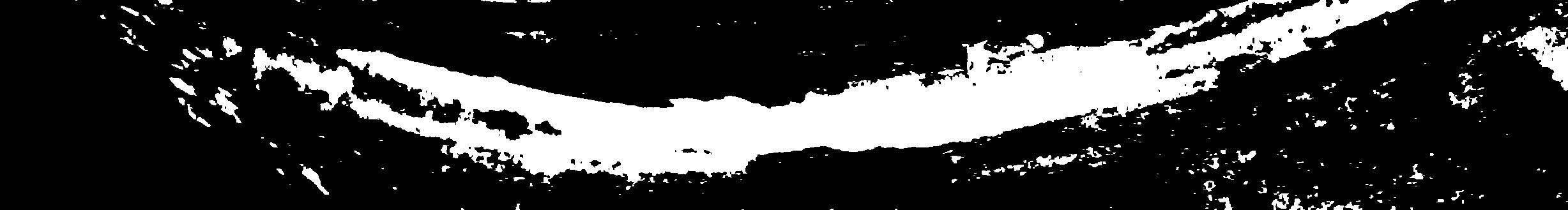

Supplement: S6 Data — (ZIP) [file pone.0297284.s006.zip › Level 3 processed Sample/processed_10/latex/DBO_latex.jpg]

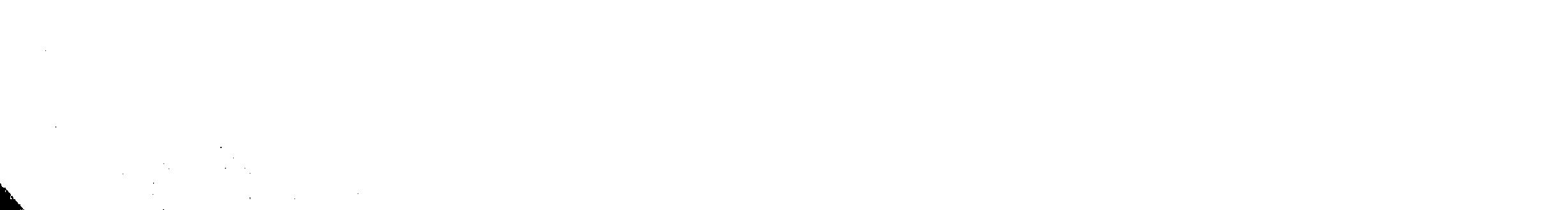

Supplement: S6 Data — (ZIP) [file pone.0297284.s006.zip › Level 3 processed Sample/processed_10/latex/OTSU_latex.jpg]

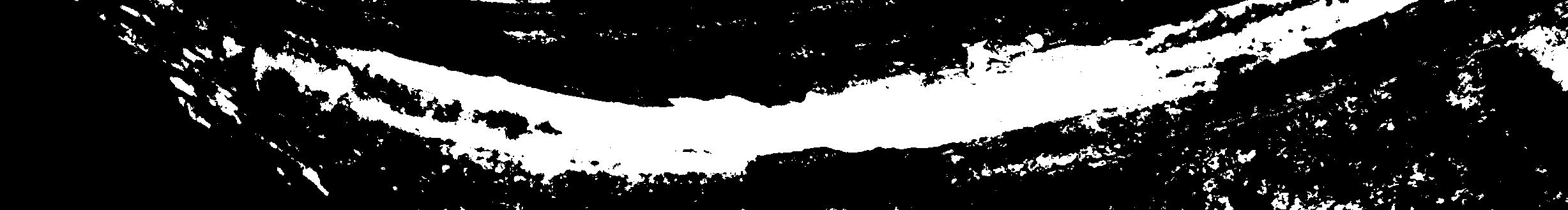

Supplement: S6 Data — (ZIP) [file pone.0297284.s006.zip › Level 3 processed Sample/processed_10/latex/WSO_latex.jpg]

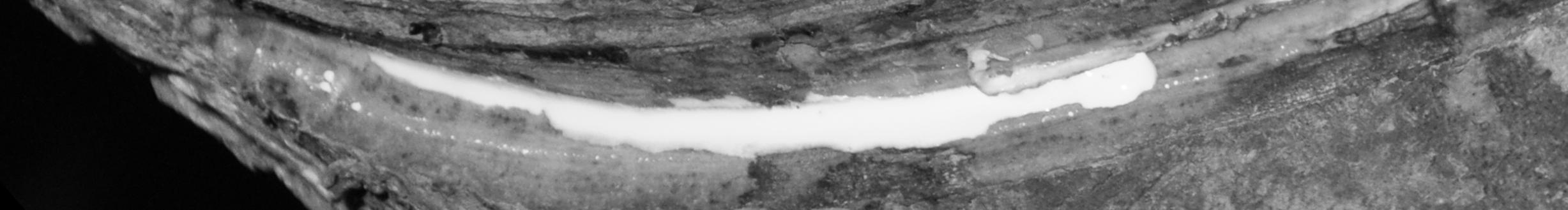

Supplement: S6 Data — (ZIP) [file pone.0297284.s006.zip › Level 3 processed Sample/processed_10/original_image.jpg]

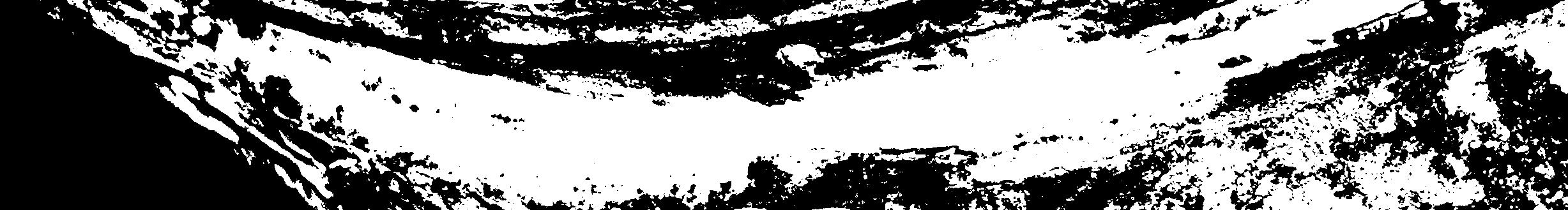

Supplement: S6 Data — (ZIP) [file pone.0297284.s006.zip › Level 3 processed Sample/processed_10/scar/AHA_scar.jpg]

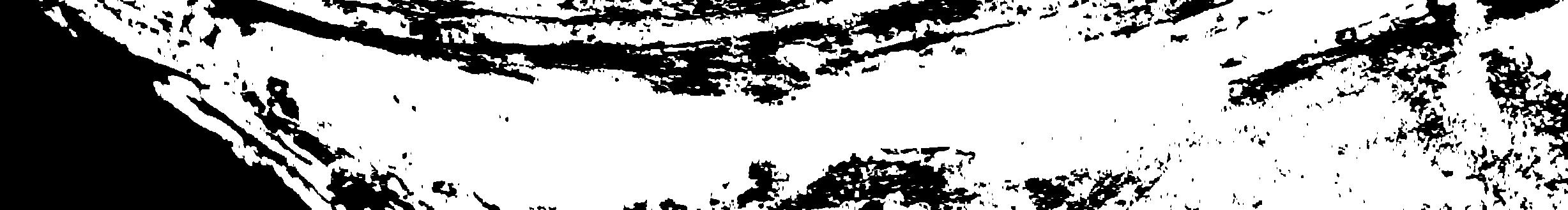

Supplement: S6 Data — (ZIP) [file pone.0297284.s006.zip › Level 3 processed Sample/processed_10/scar/DBO_scar.jpg]

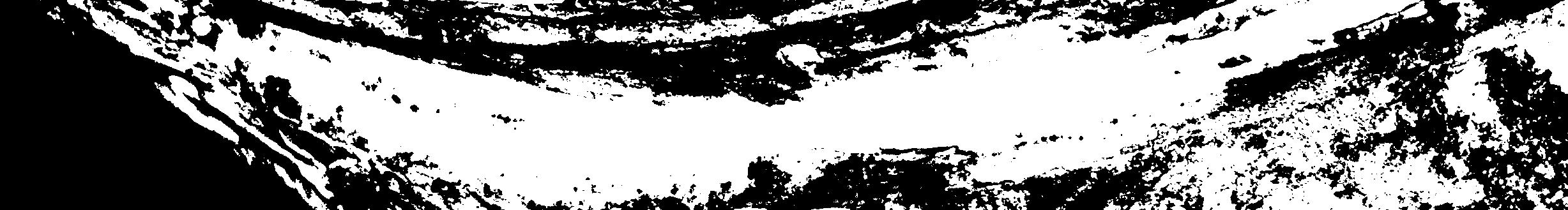

Supplement: S6 Data — (ZIP) [file pone.0297284.s006.zip › Level 3 processed Sample/processed_10/scar/WSO_scar.jpg]

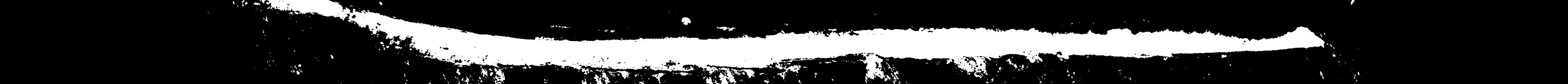

Supplement: S6 Data — (ZIP) [file pone.0297284.s006.zip › Level 3 processed Sample/processed_11/latex/AHA_latex.jpg]

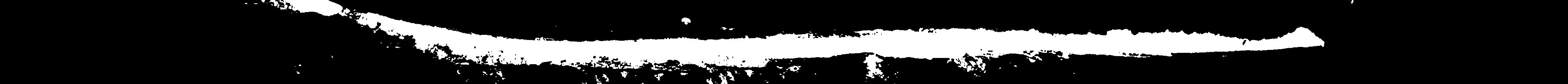

Supplement: S6 Data — (ZIP) [file pone.0297284.s006.zip › Level 3 processed Sample/processed_11/latex/DBO_latex.jpg]

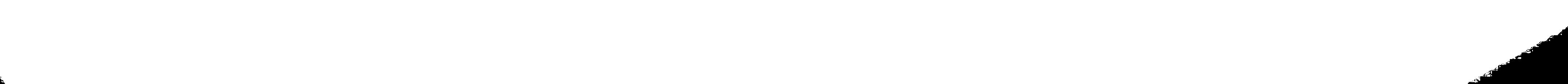

Supplement: S6 Data — (ZIP) [file pone.0297284.s006.zip › Level 3 processed Sample/processed_11/latex/OTSU_latex.jpg]

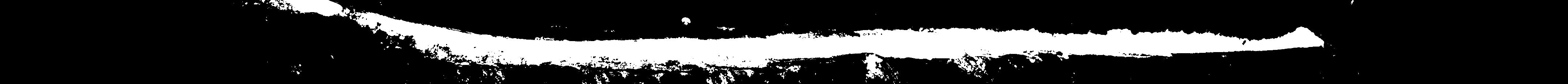

Supplement: S6 Data — (ZIP) [file pone.0297284.s006.zip › Level 3 processed Sample/processed_11/latex/WSO_latex.jpg]

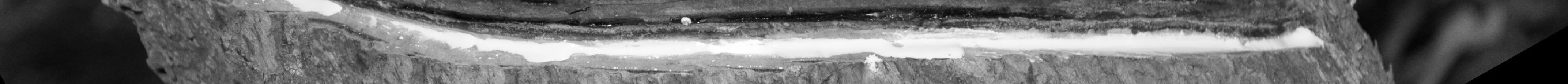

Supplement: S6 Data — (ZIP) [file pone.0297284.s006.zip › Level 3 processed Sample/processed_11/original_image.jpg]

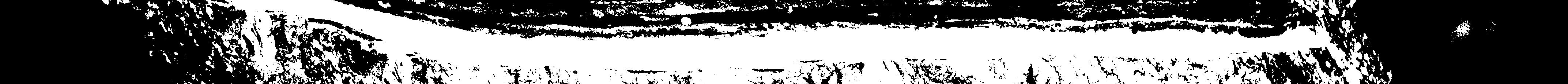

Supplement: S6 Data — (ZIP) [file pone.0297284.s006.zip › Level 3 processed Sample/processed_11/scar/AHA_scar.jpg]

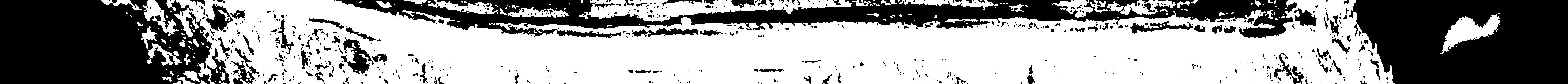

Supplement: S6 Data — (ZIP) [file pone.0297284.s006.zip › Level 3 processed Sample/processed_11/scar/DBO_scar.jpg]

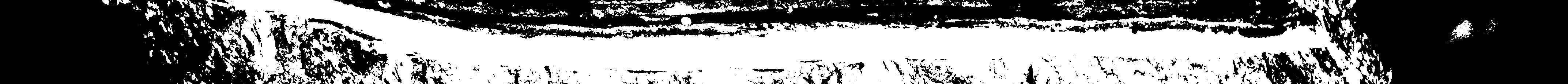

Supplement: S6 Data — (ZIP) [file pone.0297284.s006.zip › Level 3 processed Sample/processed_11/scar/WSO_scar.jpg]

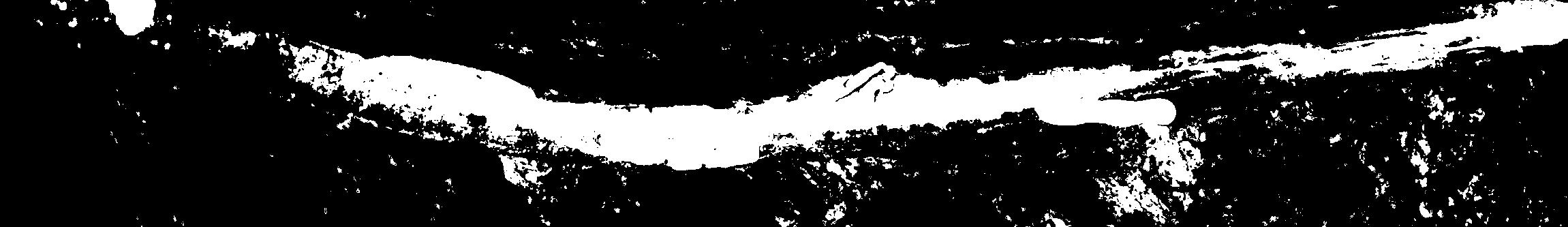

Supplement: S6 Data — (ZIP) [file pone.0297284.s006.zip › Level 3 processed Sample/processed_12/latex/AHA_latex.jpg]

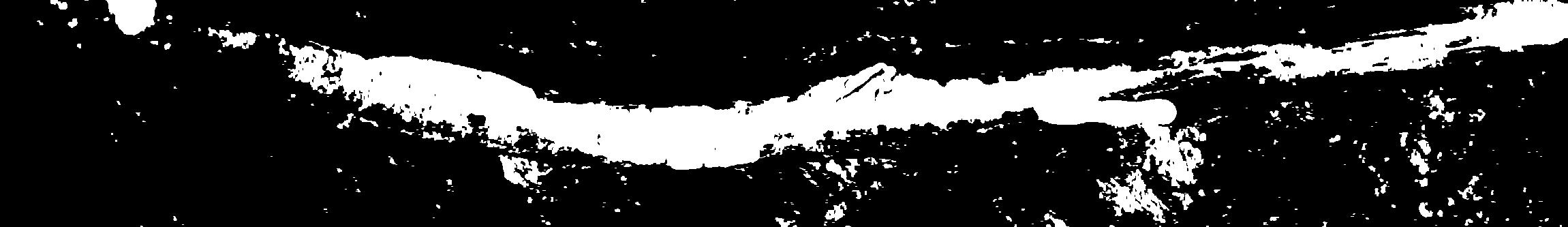

Supplement: S6 Data — (ZIP) [file pone.0297284.s006.zip › Level 3 processed Sample/processed_12/latex/DBO_latex.jpg]

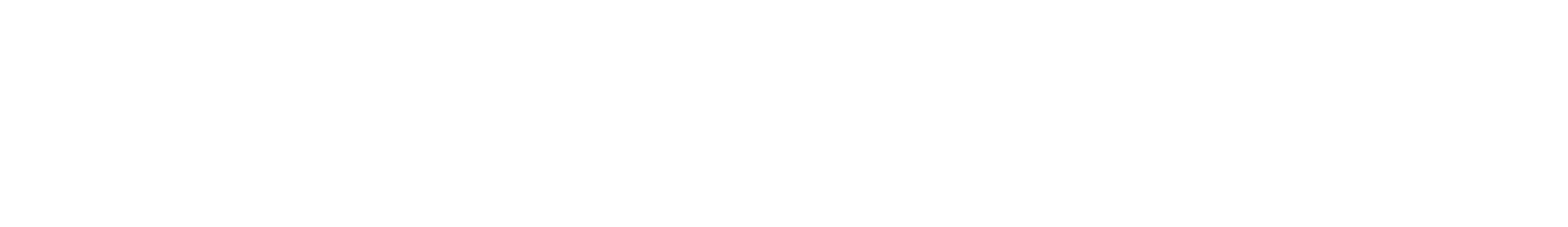

Supplement: S6 Data — (ZIP) [file pone.0297284.s006.zip › Level 3 processed Sample/processed_12/latex/OTSU_latex.jpg]

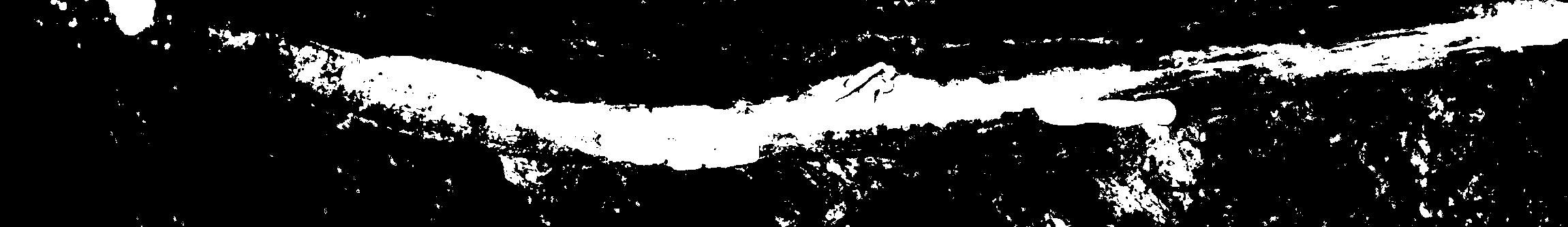

Supplement: S6 Data — (ZIP) [file pone.0297284.s006.zip › Level 3 processed Sample/processed_12/latex/WOA_latex.jpg]

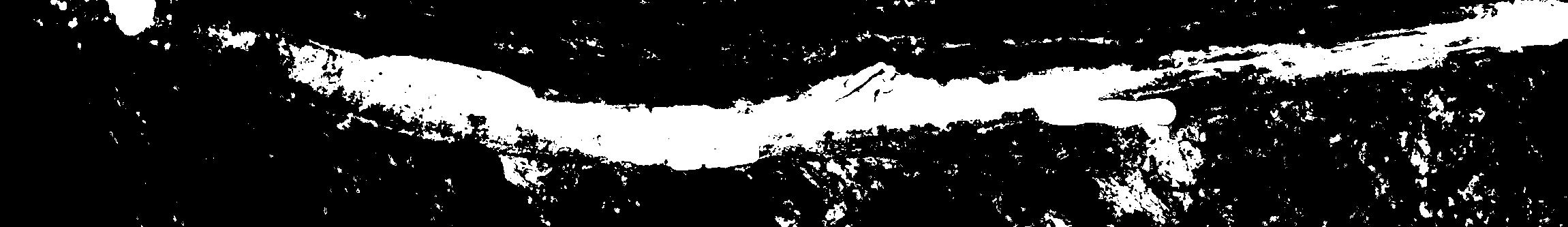

Supplement: S6 Data — (ZIP) [file pone.0297284.s006.zip › Level 3 processed Sample/processed_12/latex/WSO_latex.jpg]

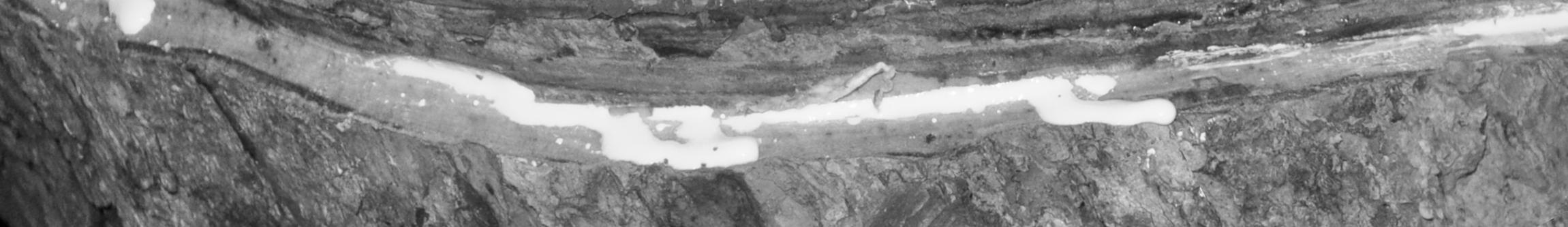

Supplement: S6 Data — (ZIP) [file pone.0297284.s006.zip › Level 3 processed Sample/processed_12/original_image.jpg]

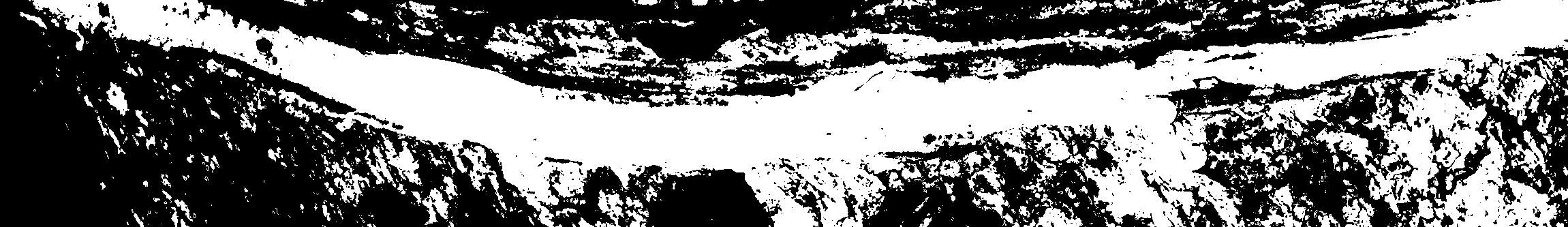

Supplement: S6 Data — (ZIP) [file pone.0297284.s006.zip › Level 3 processed Sample/processed_12/scar/AHA_scar.jpg]

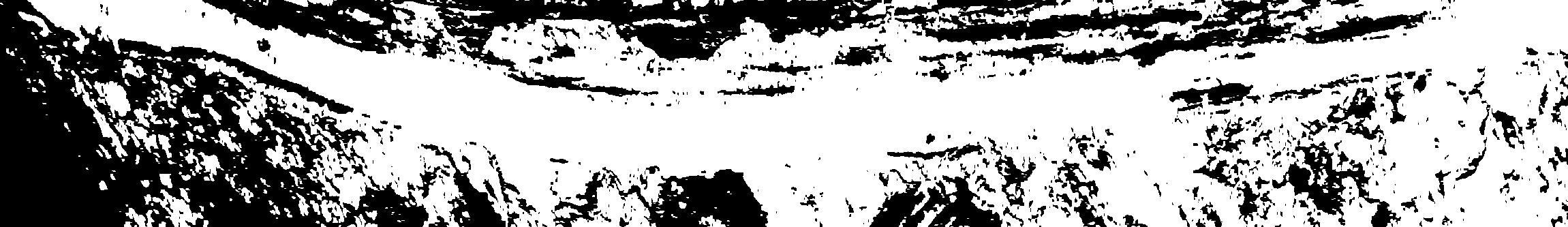

Supplement: S6 Data — (ZIP) [file pone.0297284.s006.zip › Level 3 processed Sample/processed_12/scar/DBO_scar.jpg]

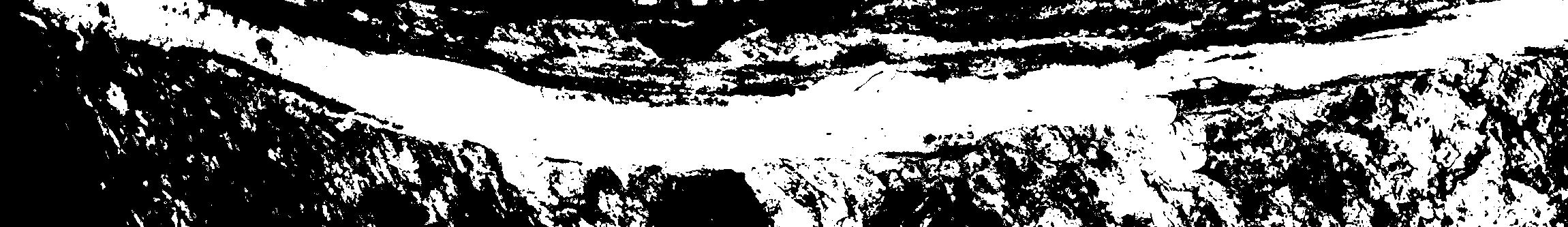

Supplement: S6 Data — (ZIP) [file pone.0297284.s006.zip › Level 3 processed Sample/processed_12/scar/WOA_scar.jpg]

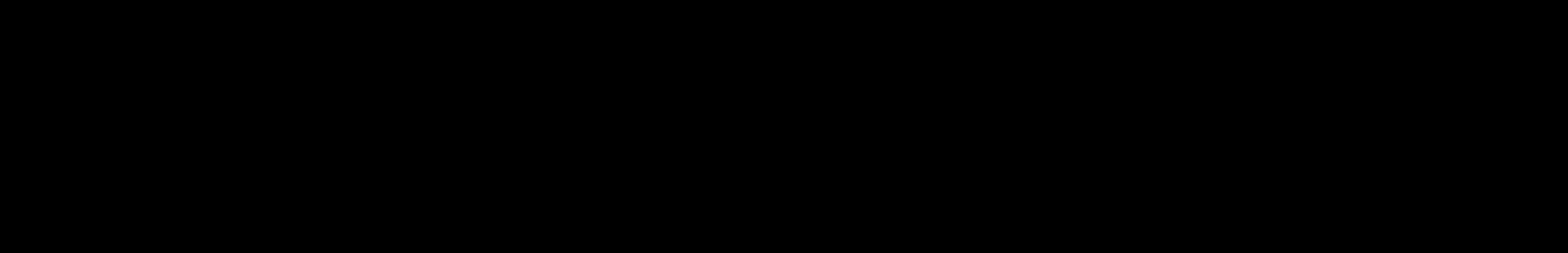

Supplement: S6 Data — (ZIP) [file pone.0297284.s006.zip › Level 3 processed Sample/processed_13/latex/AHA_latex.jpg]

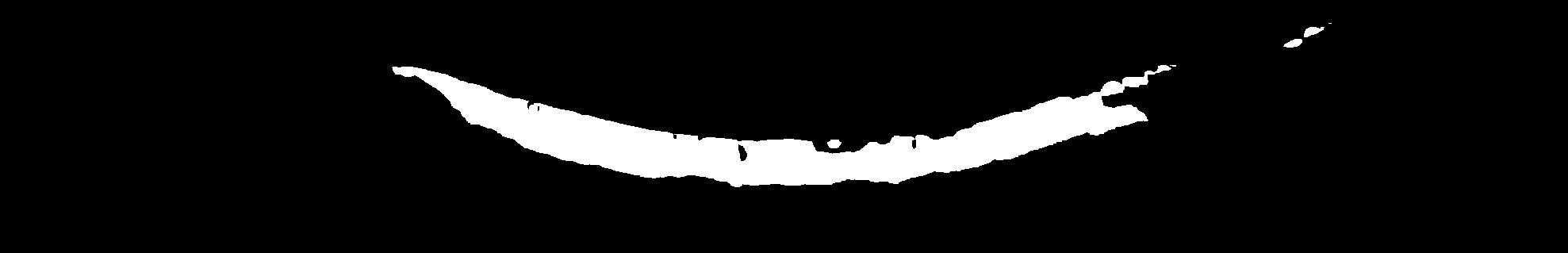

Supplement: S6 Data — (ZIP) [file pone.0297284.s006.zip › Level 3 processed Sample/processed_13/latex/DBO_latex.jpg]

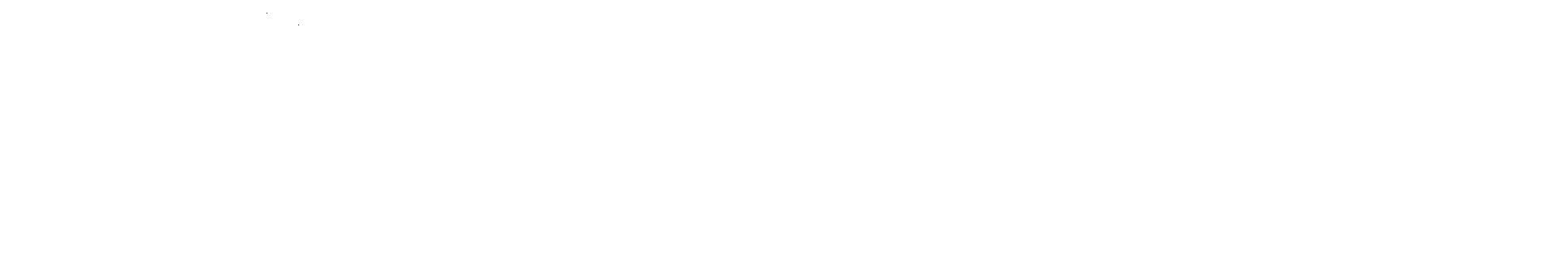

Supplement: S6 Data — (ZIP) [file pone.0297284.s006.zip › Level 3 processed Sample/processed_13/latex/OTSU_latex.jpg]

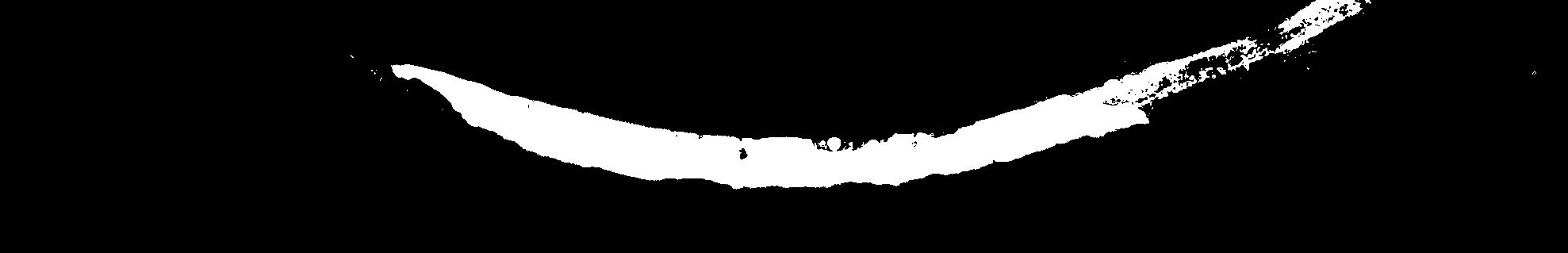

Supplement: S6 Data — (ZIP) [file pone.0297284.s006.zip › Level 3 processed Sample/processed_13/latex/WSO_latex.jpg]

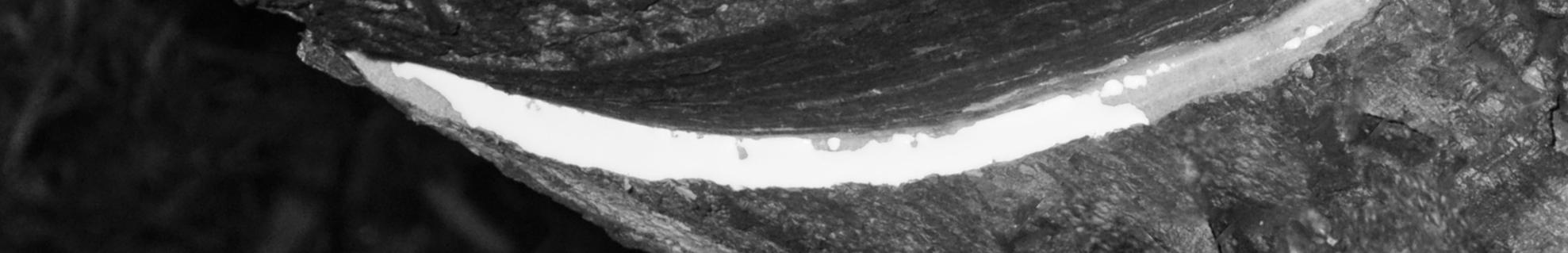

Supplement: S6 Data — (ZIP) [file pone.0297284.s006.zip › Level 3 processed Sample/processed_13/original_image.jpg]

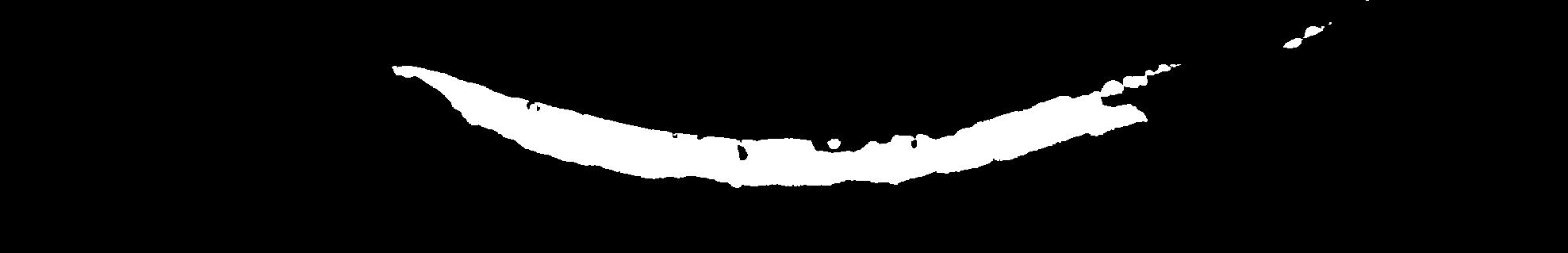

Supplement: S6 Data — (ZIP) [file pone.0297284.s006.zip › Level 3 processed Sample/processed_13/scar/AHA_scar.jpg]

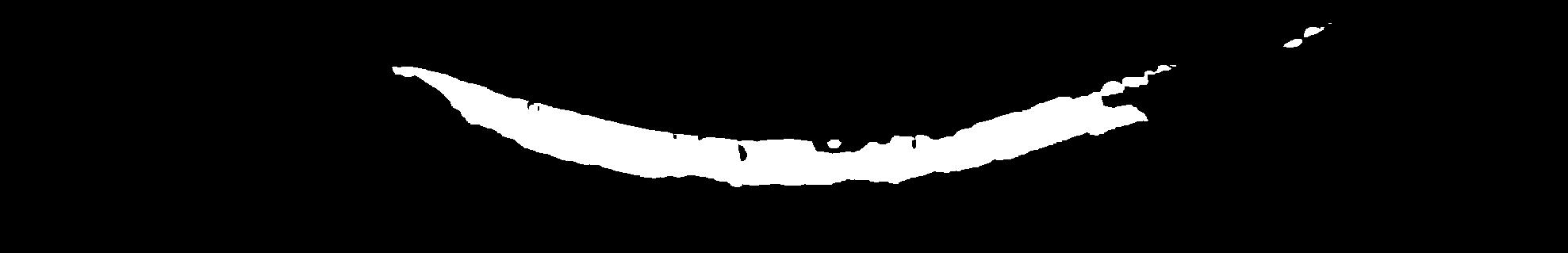

Supplement: S6 Data — (ZIP) [file pone.0297284.s006.zip › Level 3 processed Sample/processed_13/scar/DBO_scar.jpg]

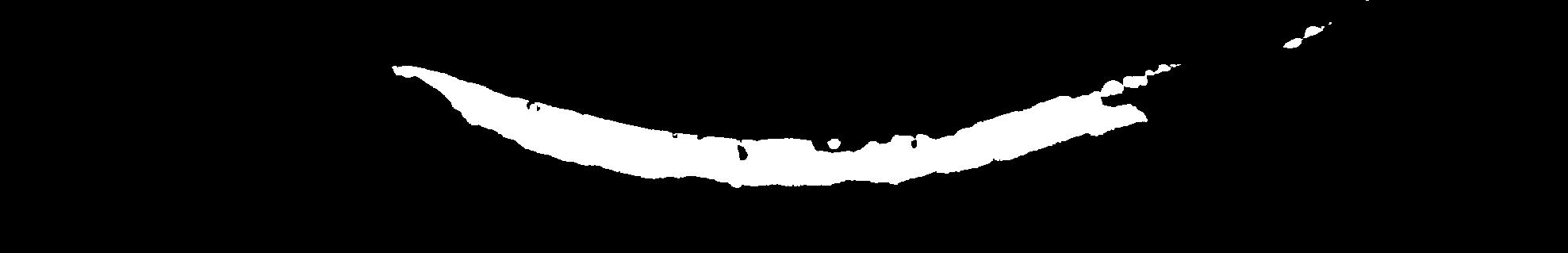

Supplement: S6 Data — (ZIP) [file pone.0297284.s006.zip › Level 3 processed Sample/processed_13/scar/WOA_scar.jpg]

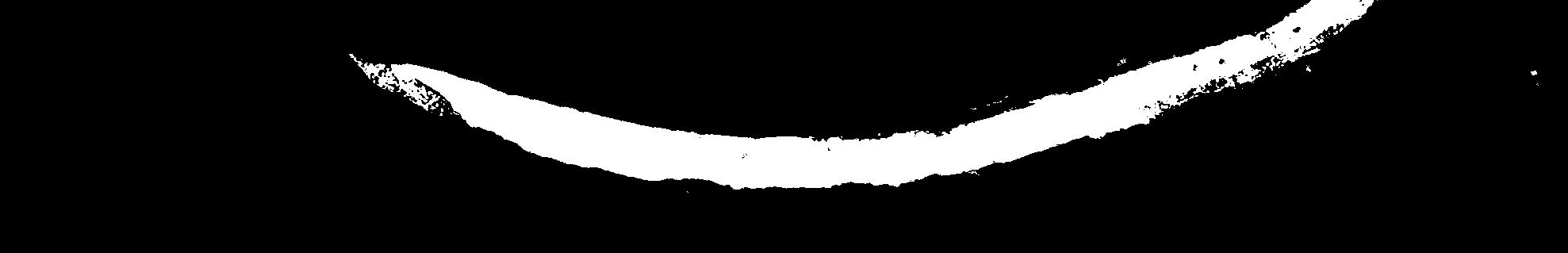

Supplement: S6 Data — (ZIP) [file pone.0297284.s006.zip › Level 3 processed Sample/processed_13/scar/WSO_scar.jpg]

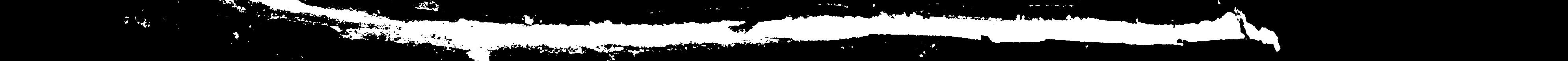

Supplement: S6 Data — (ZIP) [file pone.0297284.s006.zip › Level 3 processed Sample/processed_14/latex/AHA_latex.jpg]

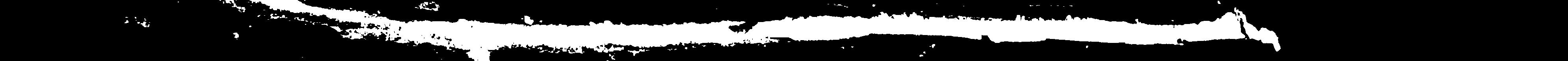

Supplement: S6 Data — (ZIP) [file pone.0297284.s006.zip › Level 3 processed Sample/processed_14/latex/DBO_latex.jpg]

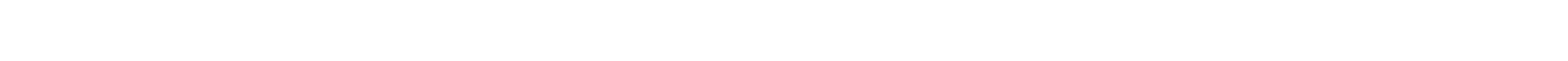

Supplement: S6 Data — (ZIP) [file pone.0297284.s006.zip › Level 3 processed Sample/processed_14/latex/OTSU_latex.jpg]

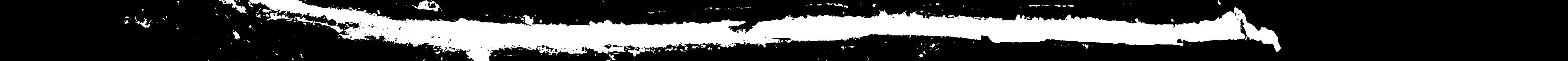

Supplement: S6 Data — (ZIP) [file pone.0297284.s006.zip › Level 3 processed Sample/processed_14/latex/WSO_latex.jpg]

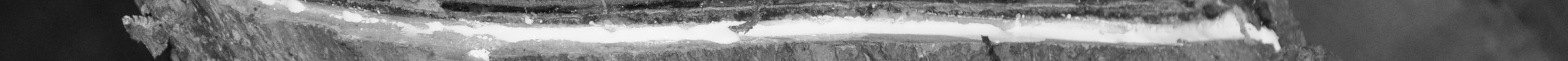

Supplement: S6 Data — (ZIP) [file pone.0297284.s006.zip › Level 3 processed Sample/processed_14/original_image.jpg]

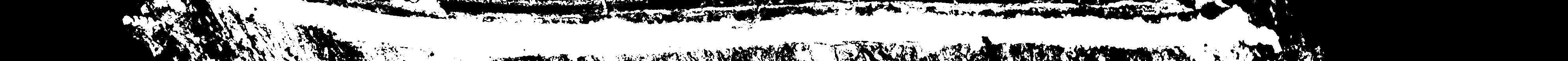

Supplement: S6 Data — (ZIP) [file pone.0297284.s006.zip › Level 3 processed Sample/processed_14/scar/AHA_scar.jpg]

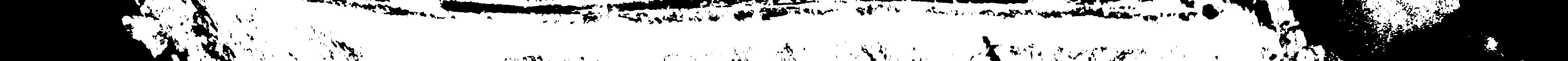

Supplement: S6 Data — (ZIP) [file pone.0297284.s006.zip › Level 3 processed Sample/processed_14/scar/DBO_scar.jpg]

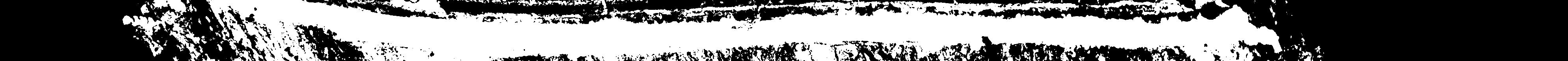

Supplement: S6 Data — (ZIP) [file pone.0297284.s006.zip › Level 3 processed Sample/processed_14/scar/WSO_scar.jpg]

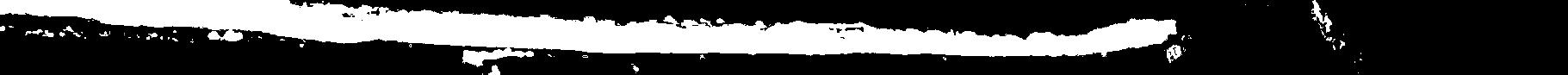

Supplement: S6 Data — (ZIP) [file pone.0297284.s006.zip › Level 3 processed Sample/processed_15/latex/AHA_latex.jpg]

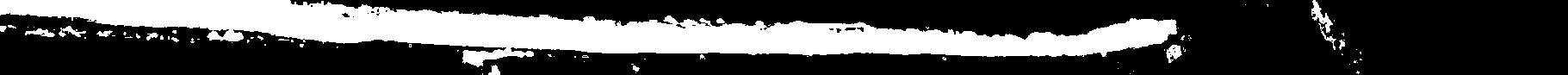

Supplement: S6 Data — (ZIP) [file pone.0297284.s006.zip › Level 3 processed Sample/processed_15/latex/CSA_latex.jpg]

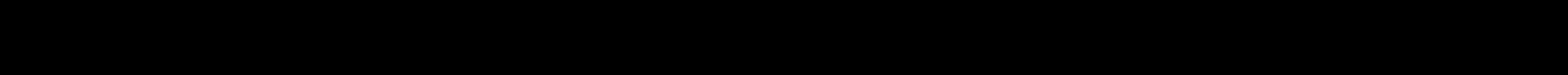

Supplement: S6 Data — (ZIP) [file pone.0297284.s006.zip › Level 3 processed Sample/processed_15/latex/DBO_latex.jpg]

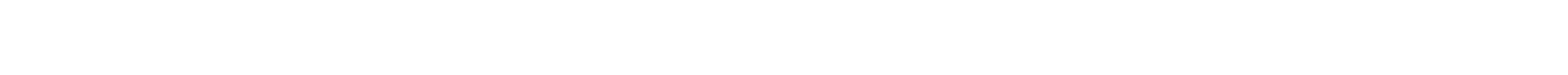

Supplement: S6 Data — (ZIP) [file pone.0297284.s006.zip › Level 3 processed Sample/processed_15/latex/OTSU_latex.jpg]

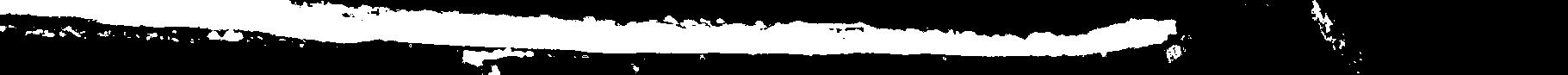

Supplement: S6 Data — (ZIP) [file pone.0297284.s006.zip › Level 3 processed Sample/processed_15/latex/WSO_latex.jpg]

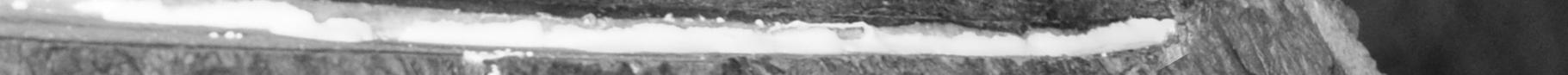

Supplement: S6 Data — (ZIP) [file pone.0297284.s006.zip › Level 3 processed Sample/processed_15/original_image.jpg]

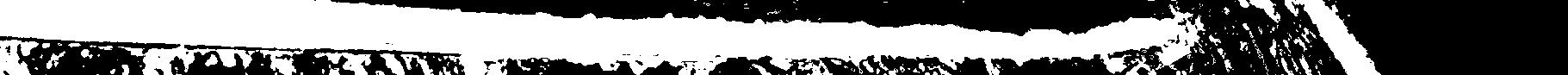

Supplement: S6 Data — (ZIP) [file pone.0297284.s006.zip › Level 3 processed Sample/processed_15/scar/AHA_scar.jpg]

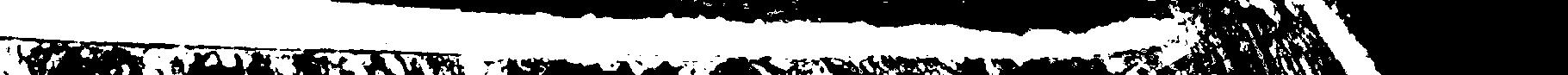

Supplement: S6 Data — (ZIP) [file pone.0297284.s006.zip › Level 3 processed Sample/processed_15/scar/CSA_scar.jpg]

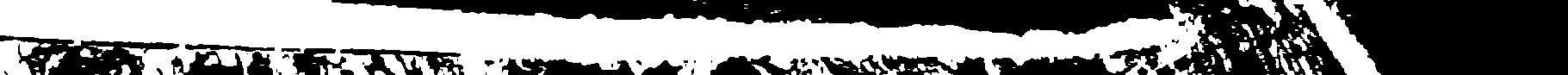

Supplement: S6 Data — (ZIP) [file pone.0297284.s006.zip › Level 3 processed Sample/processed_15/scar/DBO_scar.jpg]

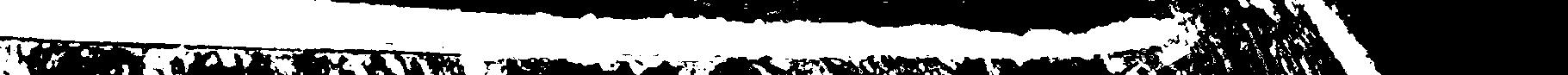

Supplement: S6 Data — (ZIP) [file pone.0297284.s006.zip › Level 3 processed Sample/processed_15/scar/WSO_scar.jpg]

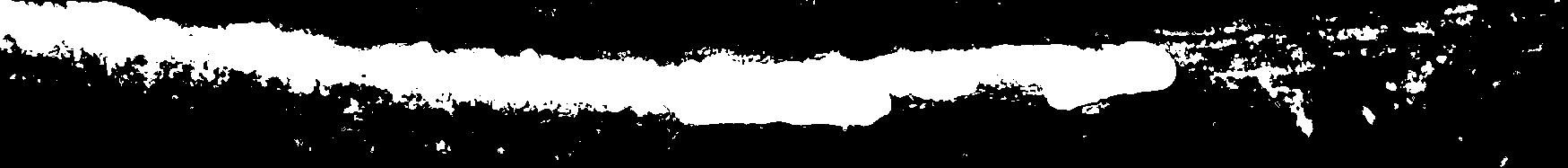

Supplement: S6 Data — (ZIP) [file pone.0297284.s006.zip › Level 3 processed Sample/processed_16/latex/AHA_latex.jpg]

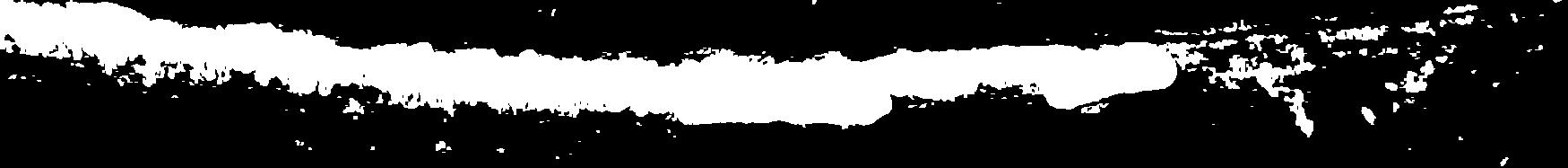

Supplement: S6 Data — (ZIP) [file pone.0297284.s006.zip › Level 3 processed Sample/processed_16/latex/DBO_latex.jpg]

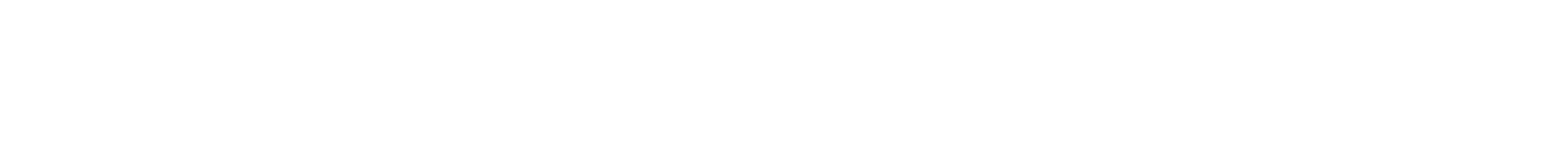

Supplement: S6 Data — (ZIP) [file pone.0297284.s006.zip › Level 3 processed Sample/processed_16/latex/OTSU_latex.jpg]

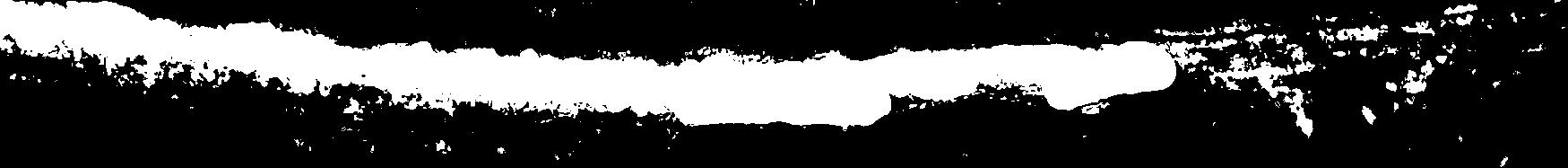

Supplement: S6 Data — (ZIP) [file pone.0297284.s006.zip › Level 3 processed Sample/processed_16/latex/WOA_latex.jpg]

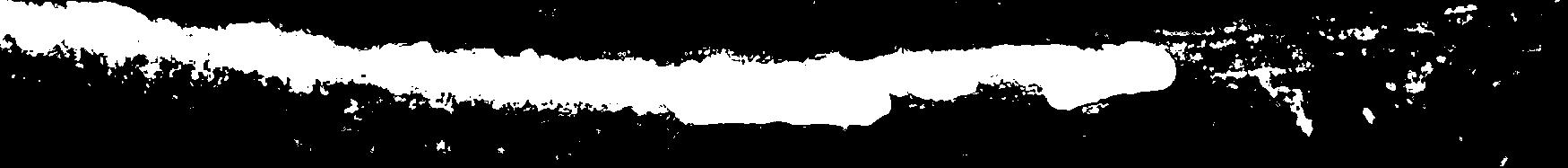

Supplement: S6 Data — (ZIP) [file pone.0297284.s006.zip › Level 3 processed Sample/processed_16/latex/WSO_latex.jpg]

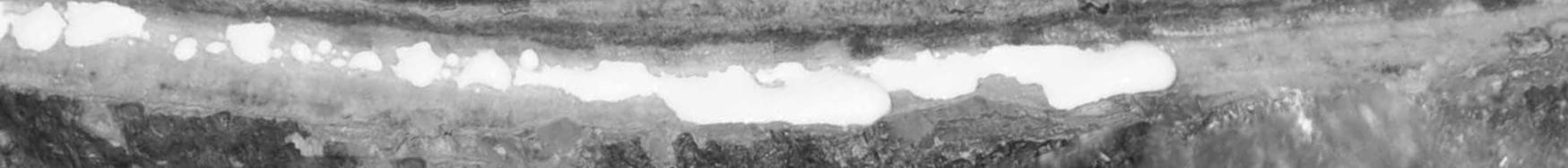

Supplement: S6 Data — (ZIP) [file pone.0297284.s006.zip › Level 3 processed Sample/processed_16/original_image.jpg]

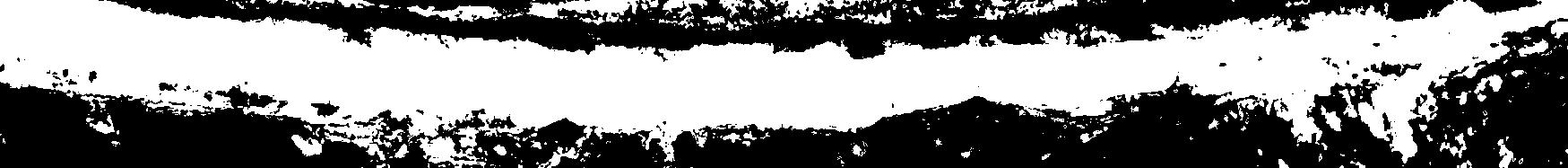

Supplement: S6 Data — (ZIP) [file pone.0297284.s006.zip › Level 3 processed Sample/processed_16/scar/AHA_scar.jpg]

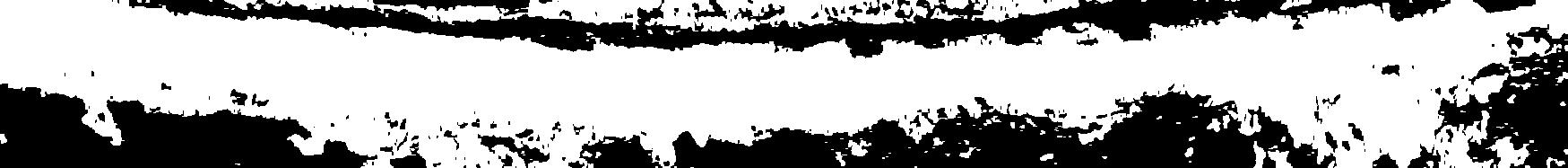

Supplement: S6 Data — (ZIP) [file pone.0297284.s006.zip › Level 3 processed Sample/processed_16/scar/DBO_scar.jpg]

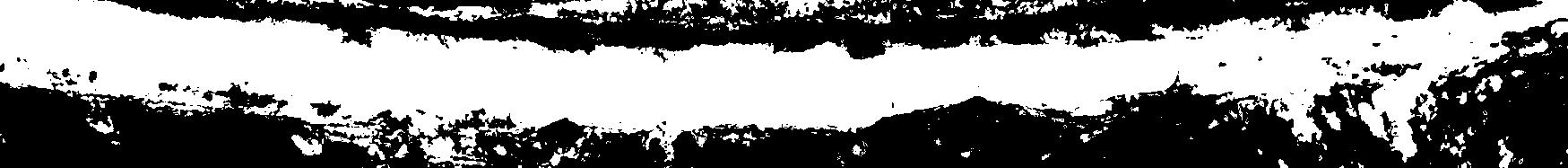

Supplement: S6 Data — (ZIP) [file pone.0297284.s006.zip › Level 3 processed Sample/processed_16/scar/GWO_scar.jpg]

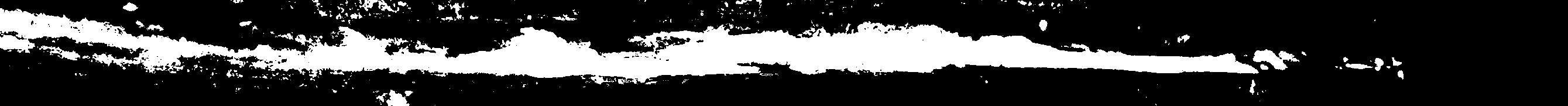

Supplement: S6 Data — (ZIP) [file pone.0297284.s006.zip › Level 3 processed Sample/processed_17/latex/AHA_latex.jpg]

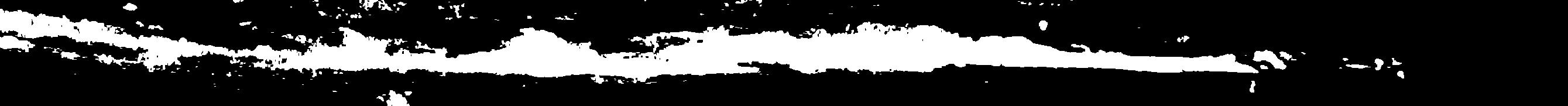

Supplement: S6 Data — (ZIP) [file pone.0297284.s006.zip › Level 3 processed Sample/processed_17/latex/DBO_latex.jpg]

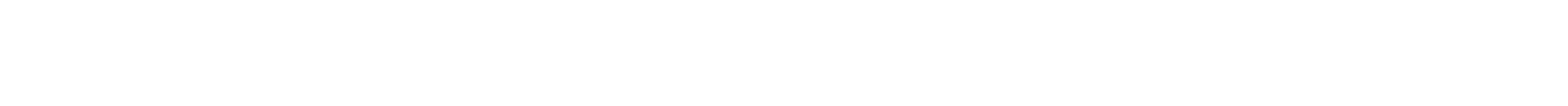

Supplement: S6 Data — (ZIP) [file pone.0297284.s006.zip › Level 3 processed Sample/processed_17/latex/OTSU_latex.jpg]

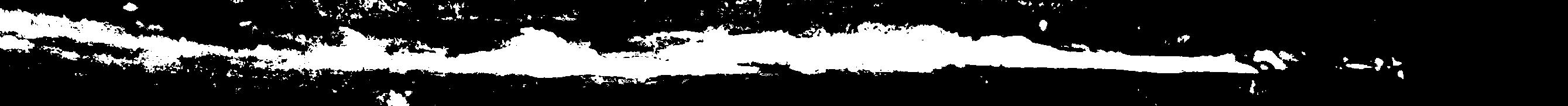

Supplement: S6 Data — (ZIP) [file pone.0297284.s006.zip › Level 3 processed Sample/processed_17/latex/WOA_latex.jpg]

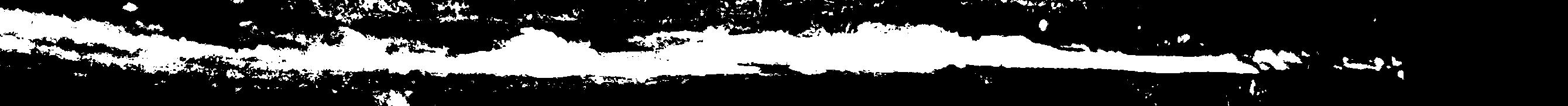

Supplement: S6 Data — (ZIP) [file pone.0297284.s006.zip › Level 3 processed Sample/processed_17/latex/WSO_latex.jpg]

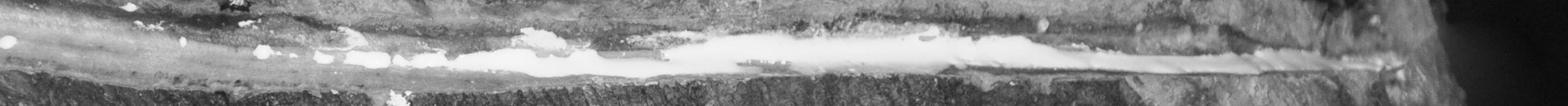

Supplement: S6 Data — (ZIP) [file pone.0297284.s006.zip › Level 3 processed Sample/processed_17/original_image.jpg]

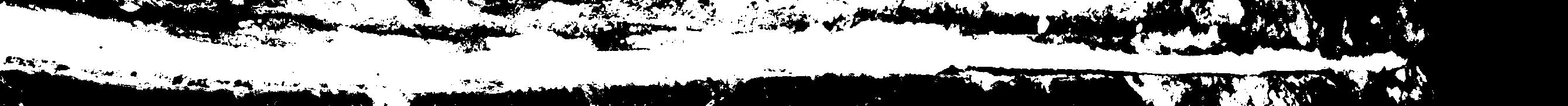

Supplement: S6 Data — (ZIP) [file pone.0297284.s006.zip › Level 3 processed Sample/processed_17/scar/AHA_scar.jpg]

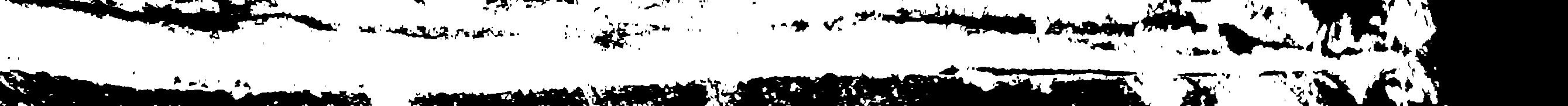

Supplement: S6 Data — (ZIP) [file pone.0297284.s006.zip › Level 3 processed Sample/processed_17/scar/DBO_scar.jpg]

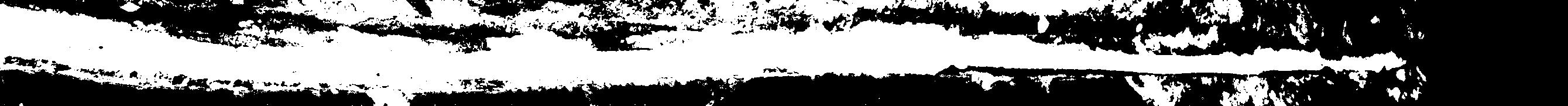

Supplement: S6 Data — (ZIP) [file pone.0297284.s006.zip › Level 3 processed Sample/processed_17/scar/WSO_scar.jpg]

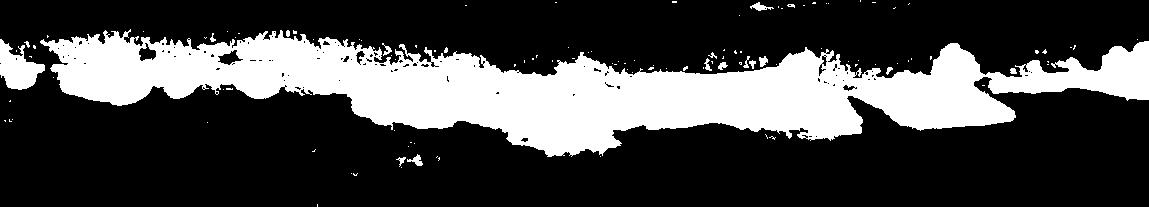

Supplement: S6 Data — (ZIP) [file pone.0297284.s006.zip › Level 3 processed Sample/processed_18/latex/AHA_latex.jpg]

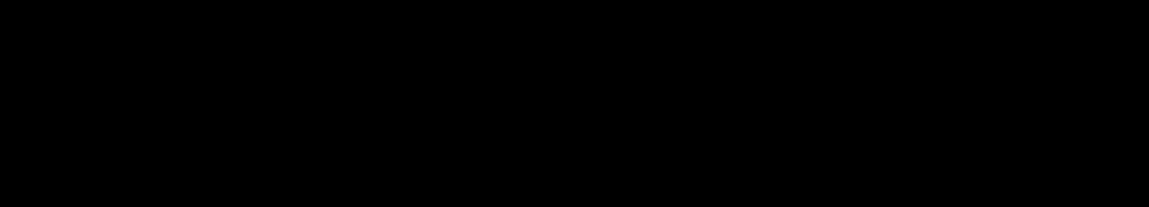

Supplement: S6 Data — (ZIP) [file pone.0297284.s006.zip › Level 3 processed Sample/processed_18/latex/DBO_latex.jpg]

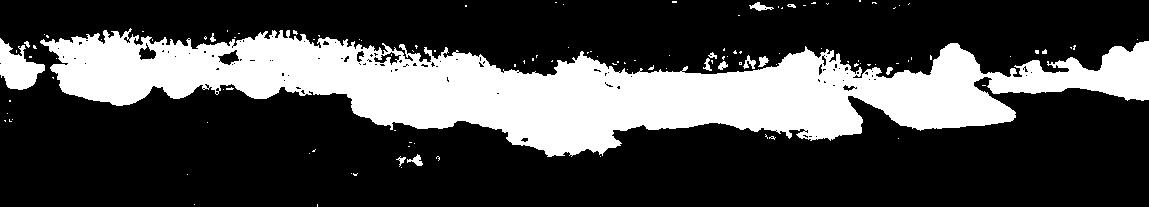

Supplement: S6 Data — (ZIP) [file pone.0297284.s006.zip › Level 3 processed Sample/processed_18/latex/GWO_latex.jpg]

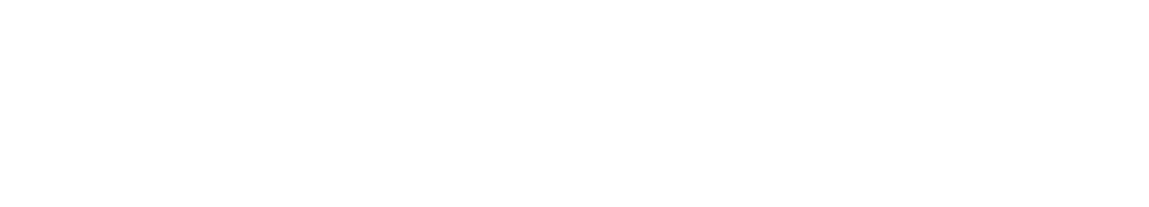

Supplement: S6 Data — (ZIP) [file pone.0297284.s006.zip › Level 3 processed Sample/processed_18/latex/OTSU_latex.jpg]

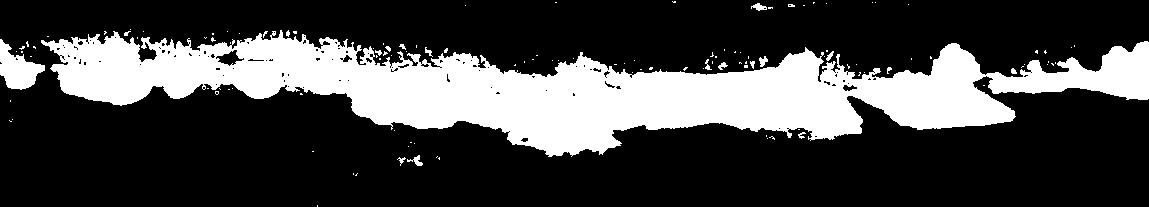

Supplement: S6 Data — (ZIP) [file pone.0297284.s006.zip › Level 3 processed Sample/processed_18/latex/WSO_latex.jpg]

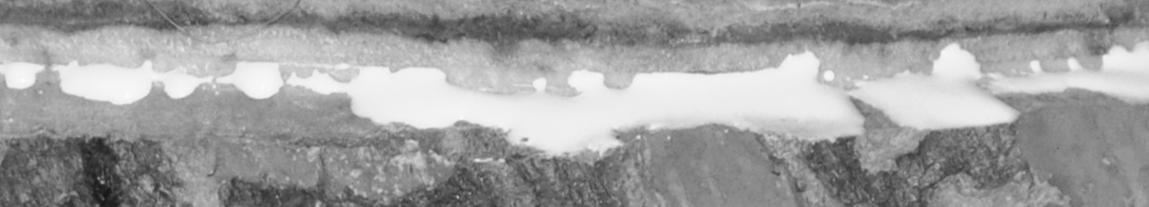

Supplement: S6 Data — (ZIP) [file pone.0297284.s006.zip › Level 3 processed Sample/processed_18/original_image.jpg]

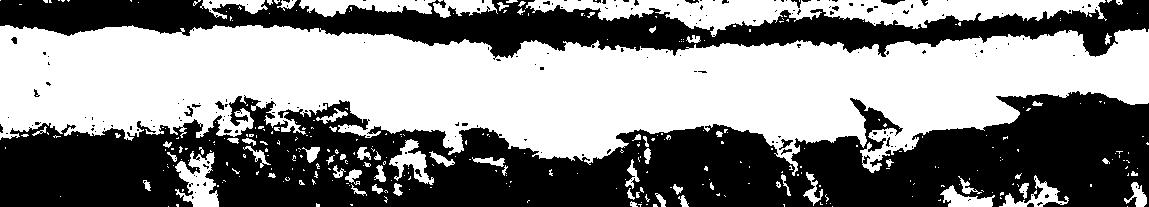

Supplement: S6 Data — (ZIP) [file pone.0297284.s006.zip › Level 3 processed Sample/processed_18/scar/AHA_scar.jpg]

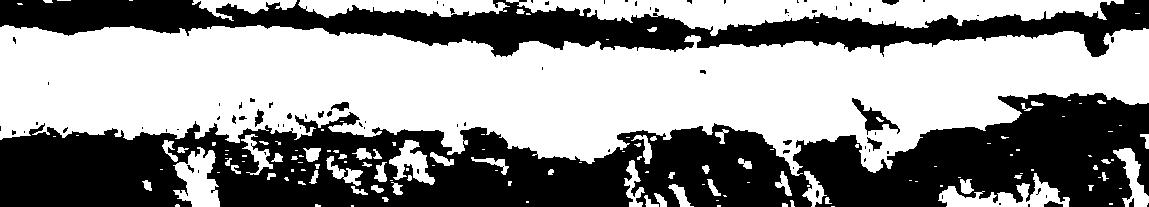

Supplement: S6 Data — (ZIP) [file pone.0297284.s006.zip › Level 3 processed Sample/processed_18/scar/DBO_scar.jpg]

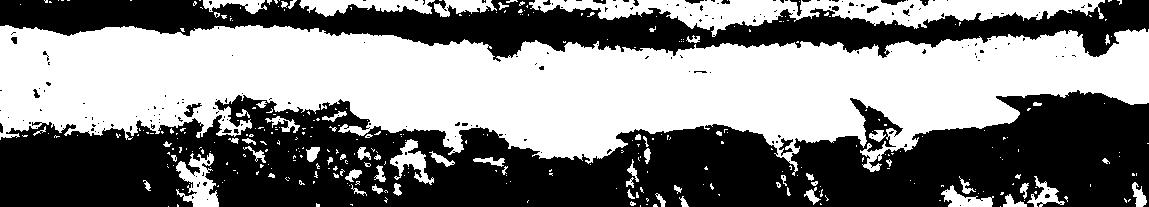

Supplement: S6 Data — (ZIP) [file pone.0297284.s006.zip › Level 3 processed Sample/processed_18/scar/WSO_scar.jpg]

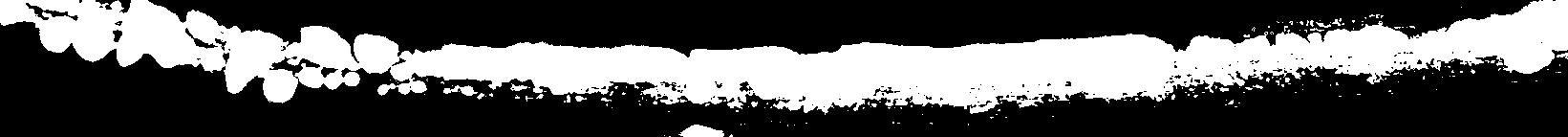

Supplement: S6 Data — (ZIP) [file pone.0297284.s006.zip › Level 3 processed Sample/processed_19/latex/AHA_latex.jpg]

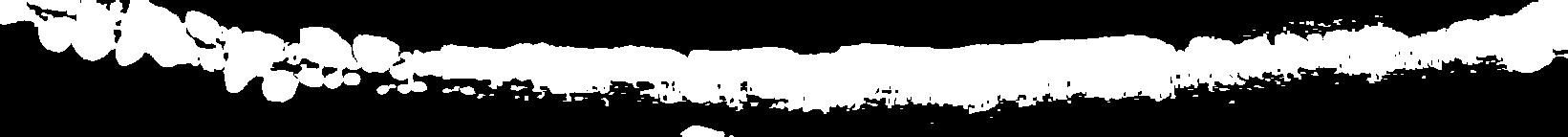

Supplement: S6 Data — (ZIP) [file pone.0297284.s006.zip › Level 3 processed Sample/processed_19/latex/DBO_latex.jpg]

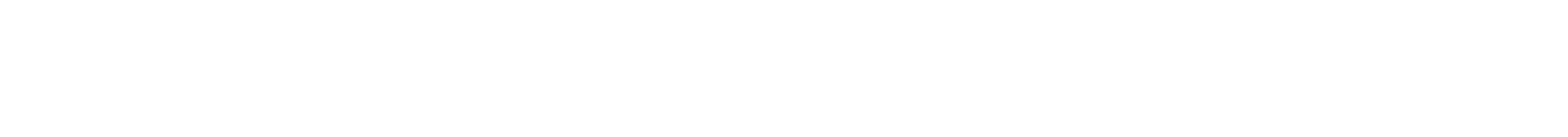

Supplement: S6 Data — (ZIP) [file pone.0297284.s006.zip › Level 3 processed Sample/processed_19/latex/OTSU_latex.jpg]

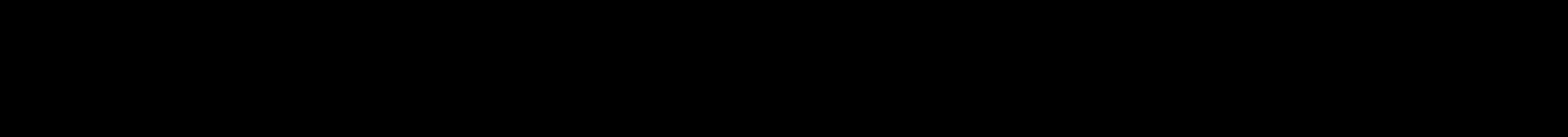

Supplement: S6 Data — (ZIP) [file pone.0297284.s006.zip › Level 3 processed Sample/processed_19/latex/SSA_latex.jpg]

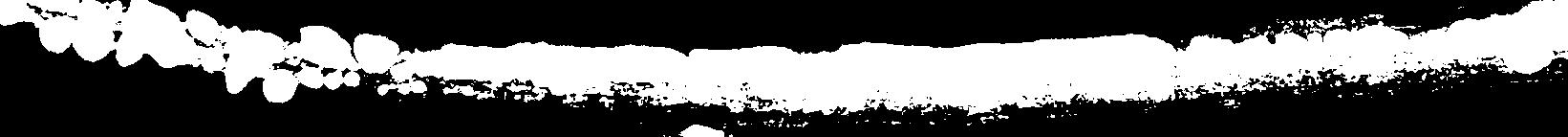

Supplement: S6 Data — (ZIP) [file pone.0297284.s006.zip › Level 3 processed Sample/processed_19/latex/WSO_latex.jpg]

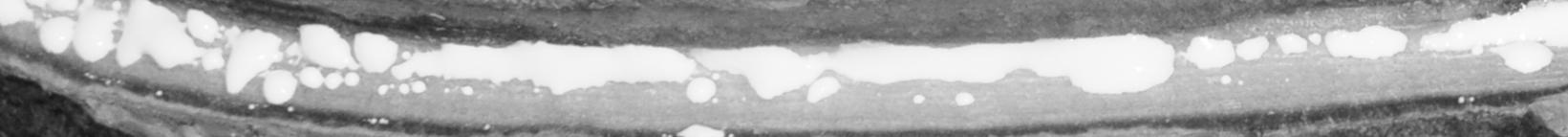

Supplement: S6 Data — (ZIP) [file pone.0297284.s006.zip › Level 3 processed Sample/processed_19/original_image.jpg]

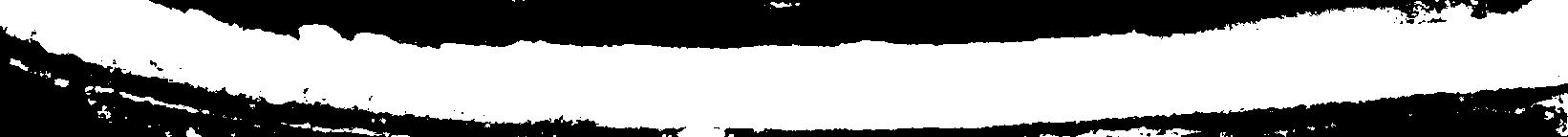

Supplement: S6 Data — (ZIP) [file pone.0297284.s006.zip › Level 3 processed Sample/processed_19/scar/AHA_scar.jpg]

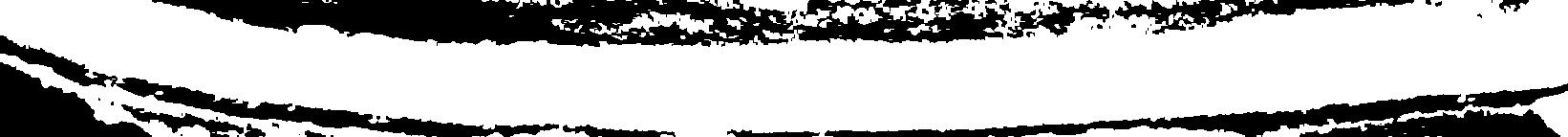

Supplement: S6 Data — (ZIP) [file pone.0297284.s006.zip › Level 3 processed Sample/processed_19/scar/DBO_scar.jpg]

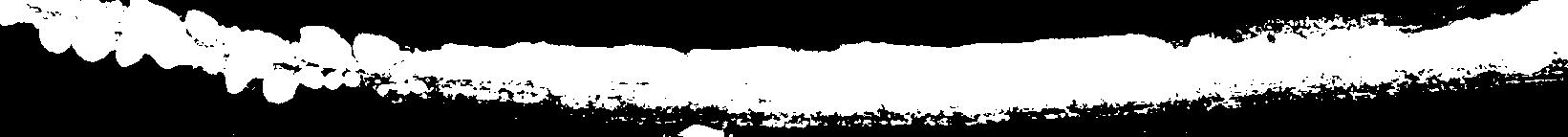

Supplement: S6 Data — (ZIP) [file pone.0297284.s006.zip › Level 3 processed Sample/processed_19/scar/SSA_scar.jpg]

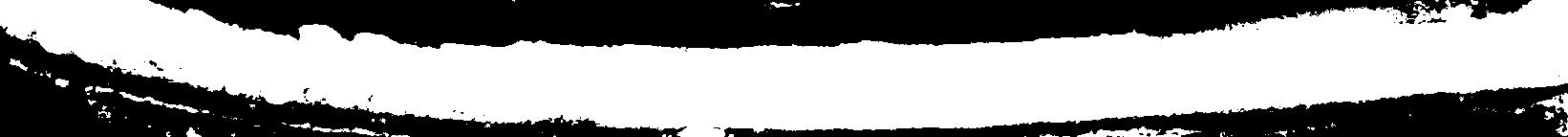

Supplement: S6 Data — (ZIP) [file pone.0297284.s006.zip › Level 3 processed Sample/processed_19/scar/WOA_scar.jpg]

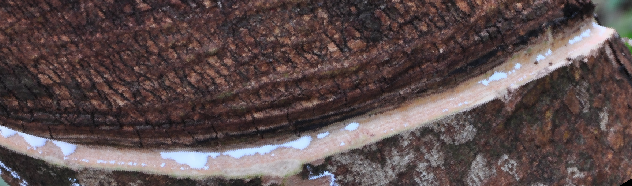

Supplement: S7 Data — (ZIP) [file pone.0297284.s007.zip › Level 4 Original Sample/4.1.jpg]

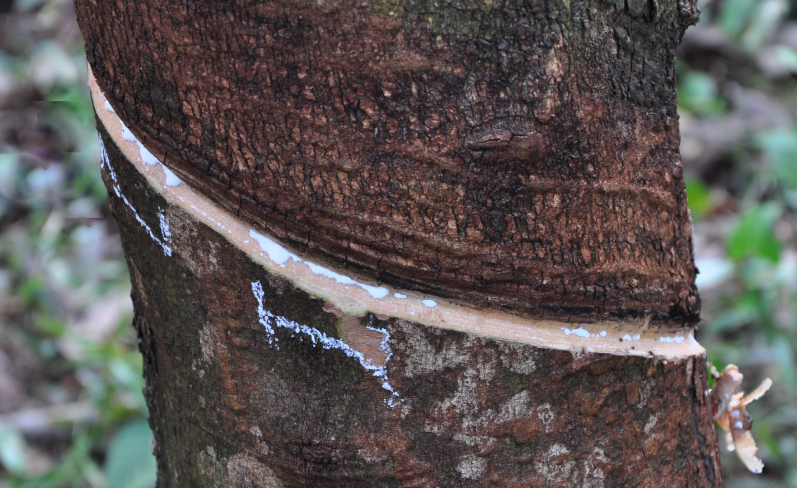

Supplement: S7 Data — (ZIP) [file pone.0297284.s007.zip › Level 4 Original Sample/4.1.png]
