# Supplementary figures and images for: Advancing image segmentation with DBO-Otsu: Addressing rubber tree diseases through enhanced threshold techniques (part 5 of 7)
Source: PLoS One. 2024 Mar 21;19(3):e0297284. doi: 10.1371/journal.pone.0297284 (PMC10956860; doi:10.1371/journal.pone.0297284)

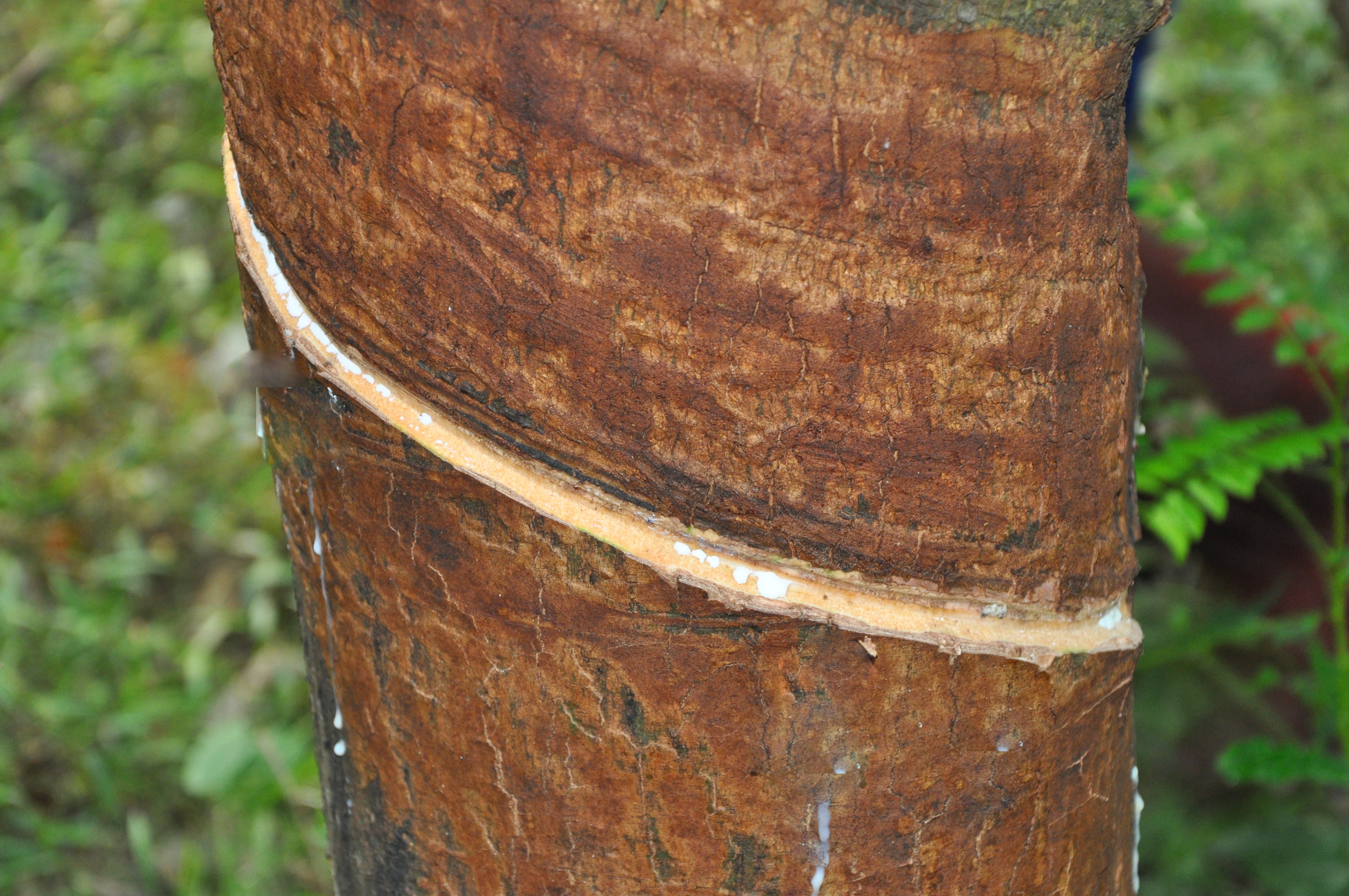

Supplement: S7 Data — (ZIP) [file pone.0297284.s007.zip › Level 4 Original Sample/4-33701-112-20141017-0440.JPG]

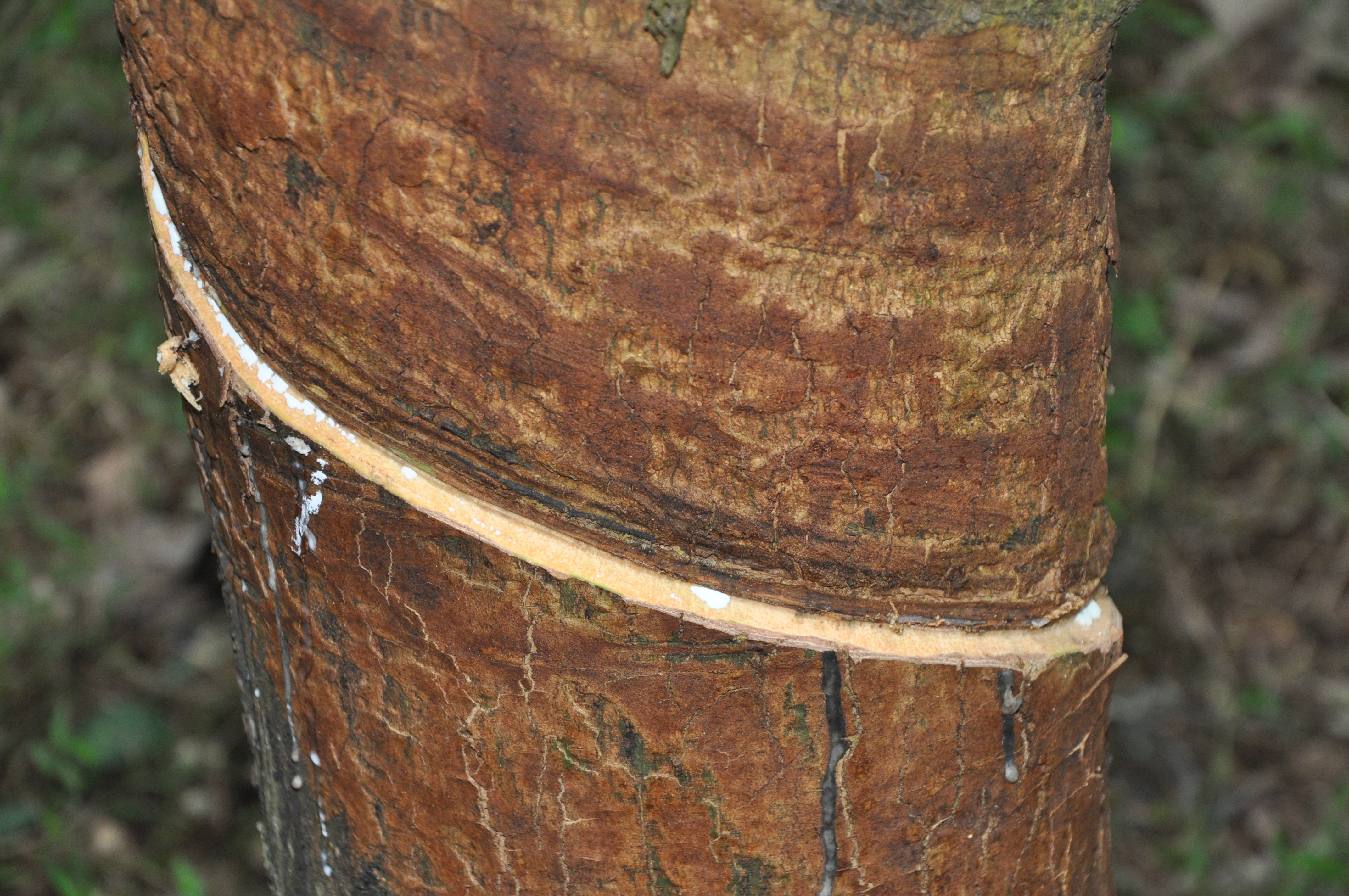

Supplement: S7 Data — (ZIP) [file pone.0297284.s007.zip › Level 4 Original Sample/4-33701-112-20141126-0019.JPG]

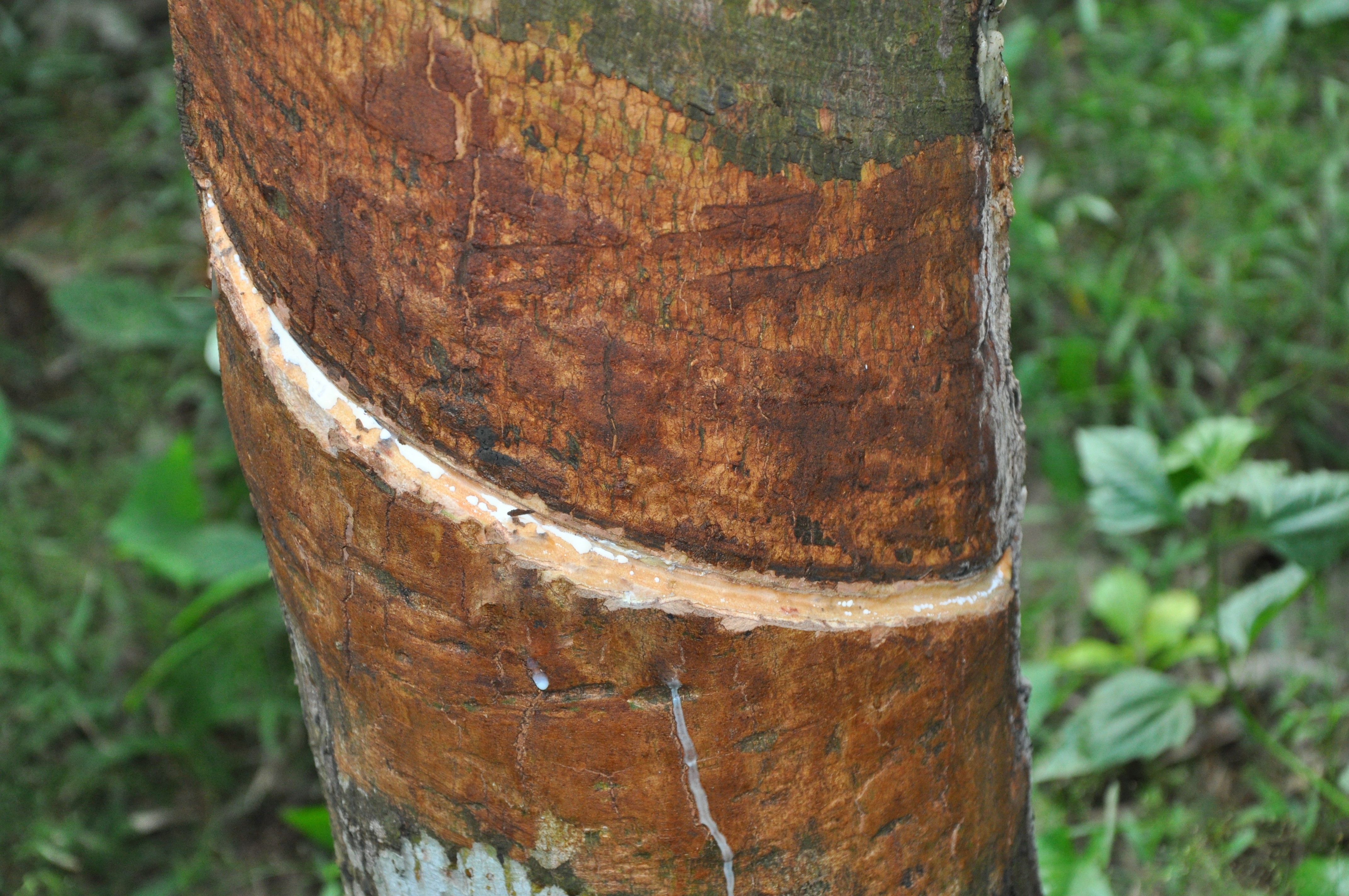

Supplement: S7 Data — (ZIP) [file pone.0297284.s007.zip › Level 4 Original Sample/4-33701-115-20141017-0442.JPG]

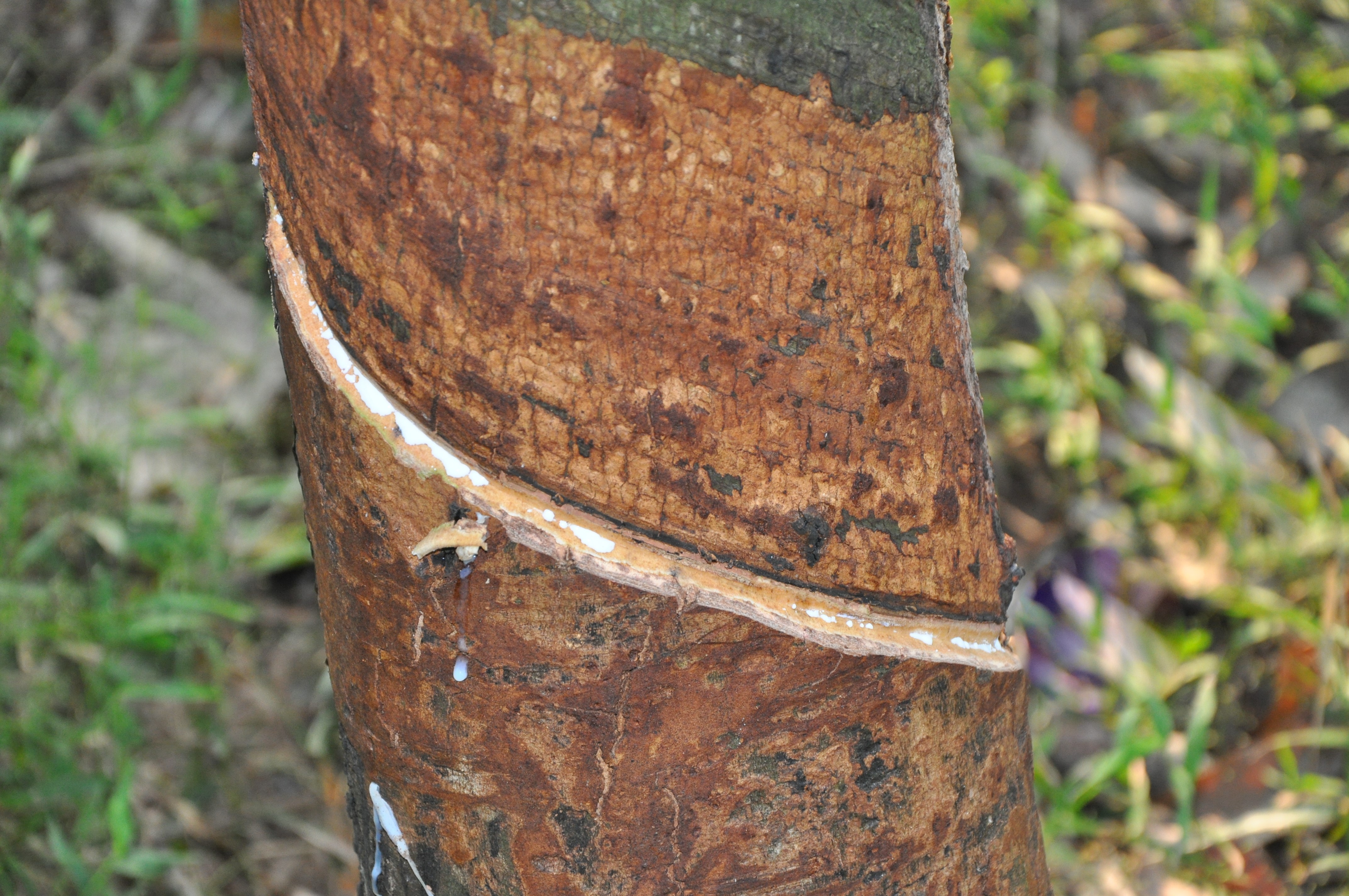

Supplement: S7 Data — (ZIP) [file pone.0297284.s007.zip › Level 4 Original Sample/4-33701-163-20141017-0446.JPG]

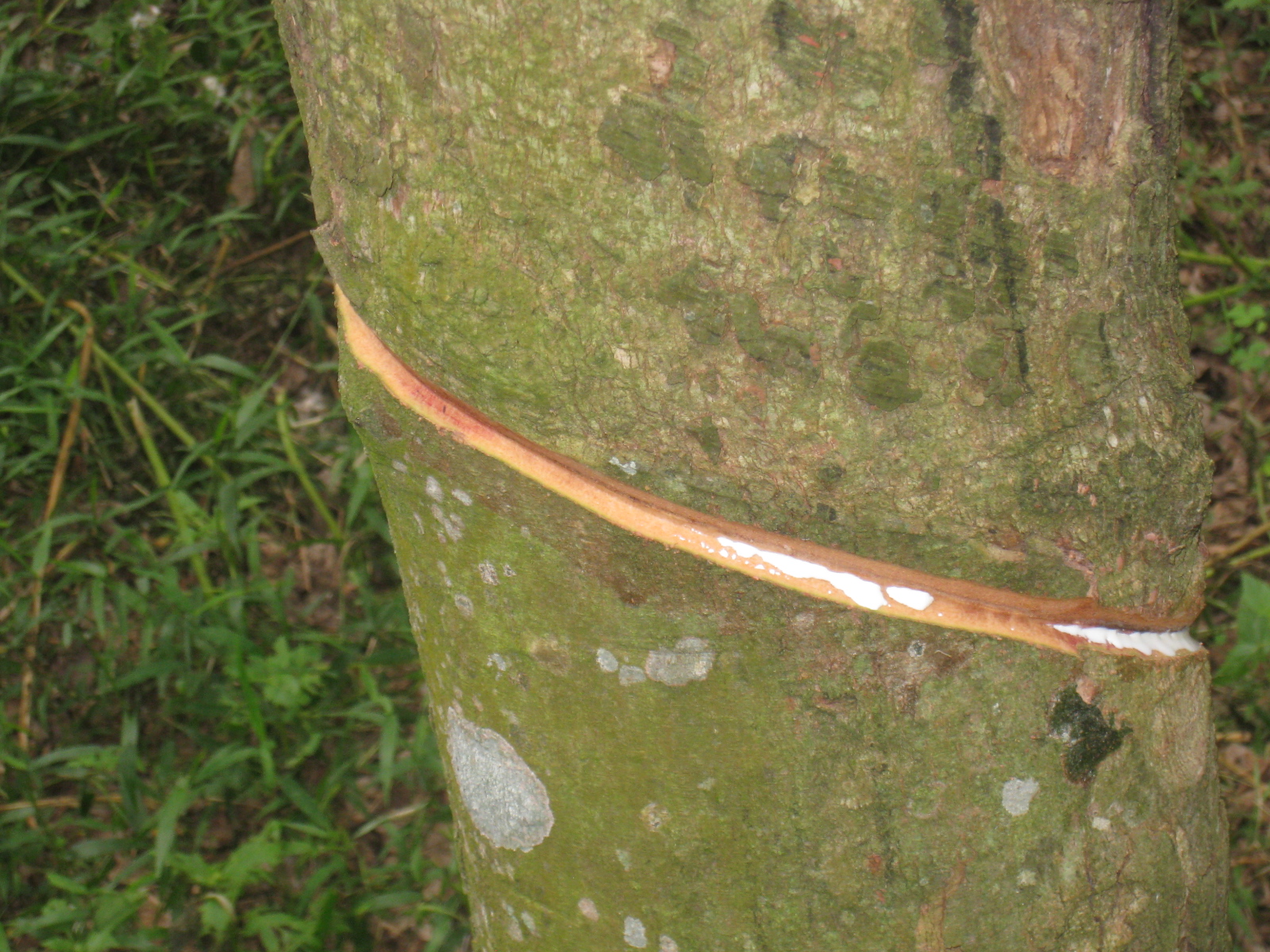

Supplement: S7 Data — (ZIP) [file pone.0297284.s007.zip › Level 4 Original Sample/4-33701-183-20140512-0001.JPG]

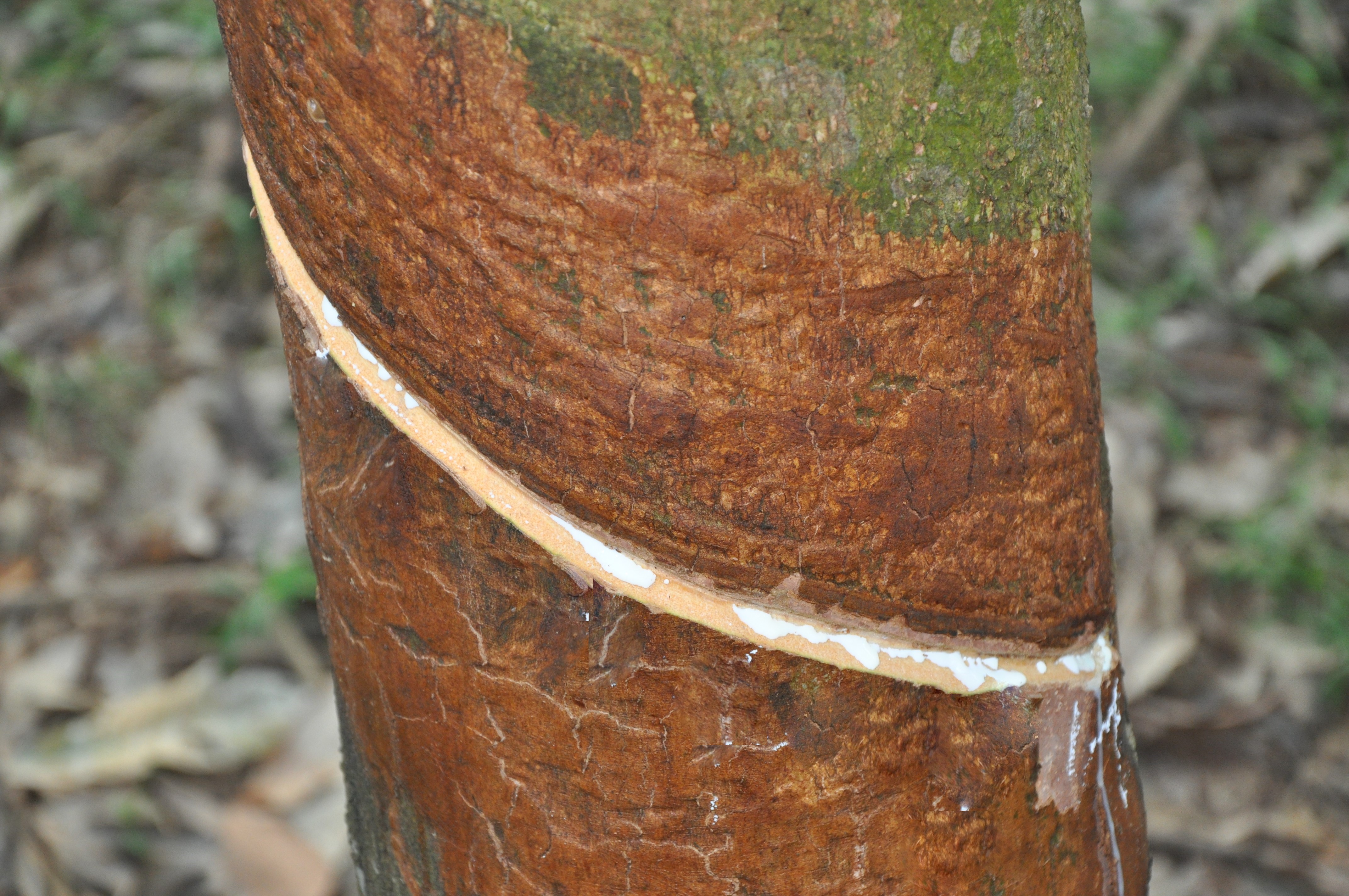

Supplement: S7 Data — (ZIP) [file pone.0297284.s007.zip › Level 4 Original Sample/4-33701-199-20141017-0478.JPG]

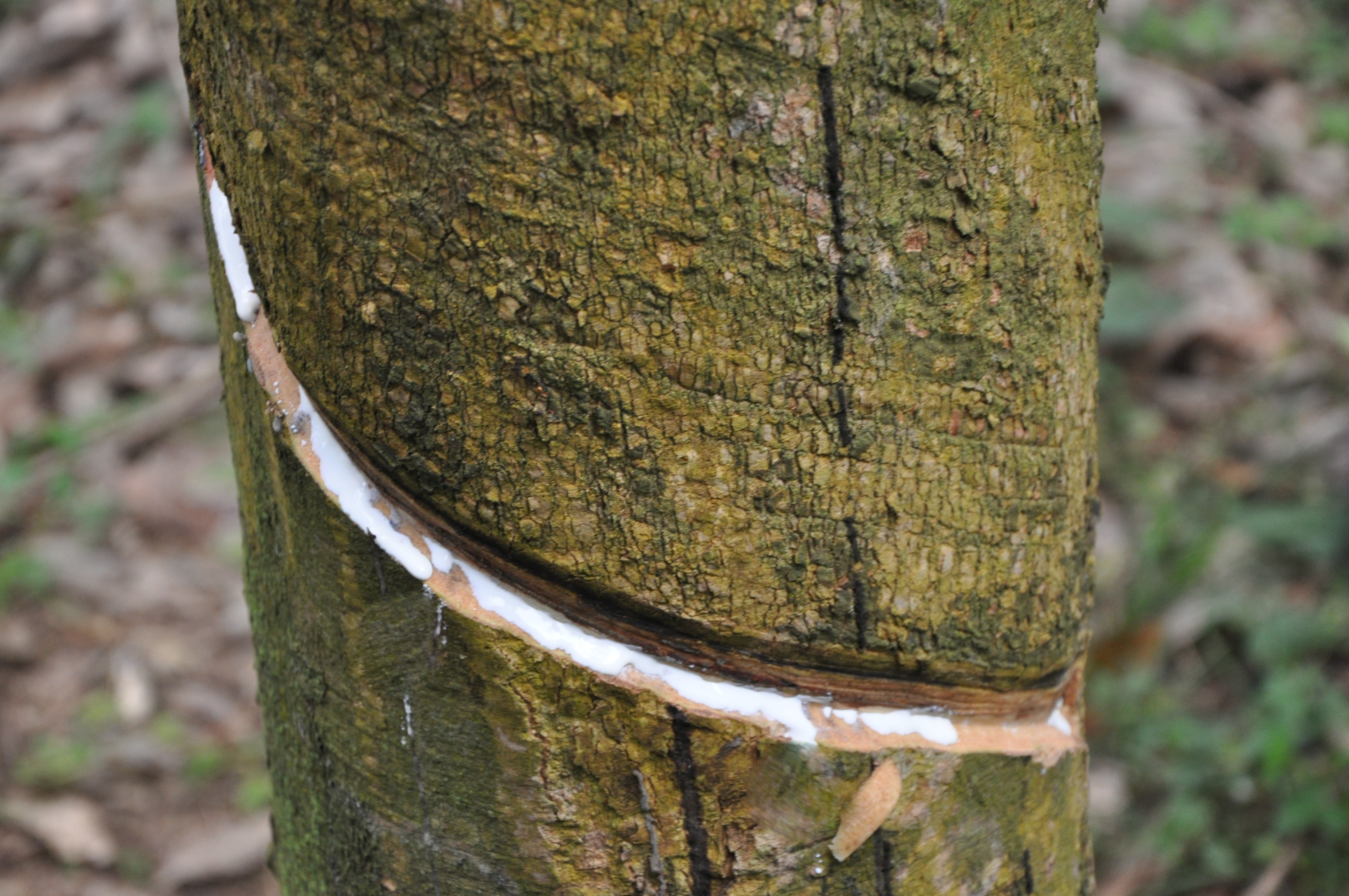

Supplement: S7 Data — (ZIP) [file pone.0297284.s007.zip › Level 4 Original Sample/4-33701-261-20141126-0056.JPG]

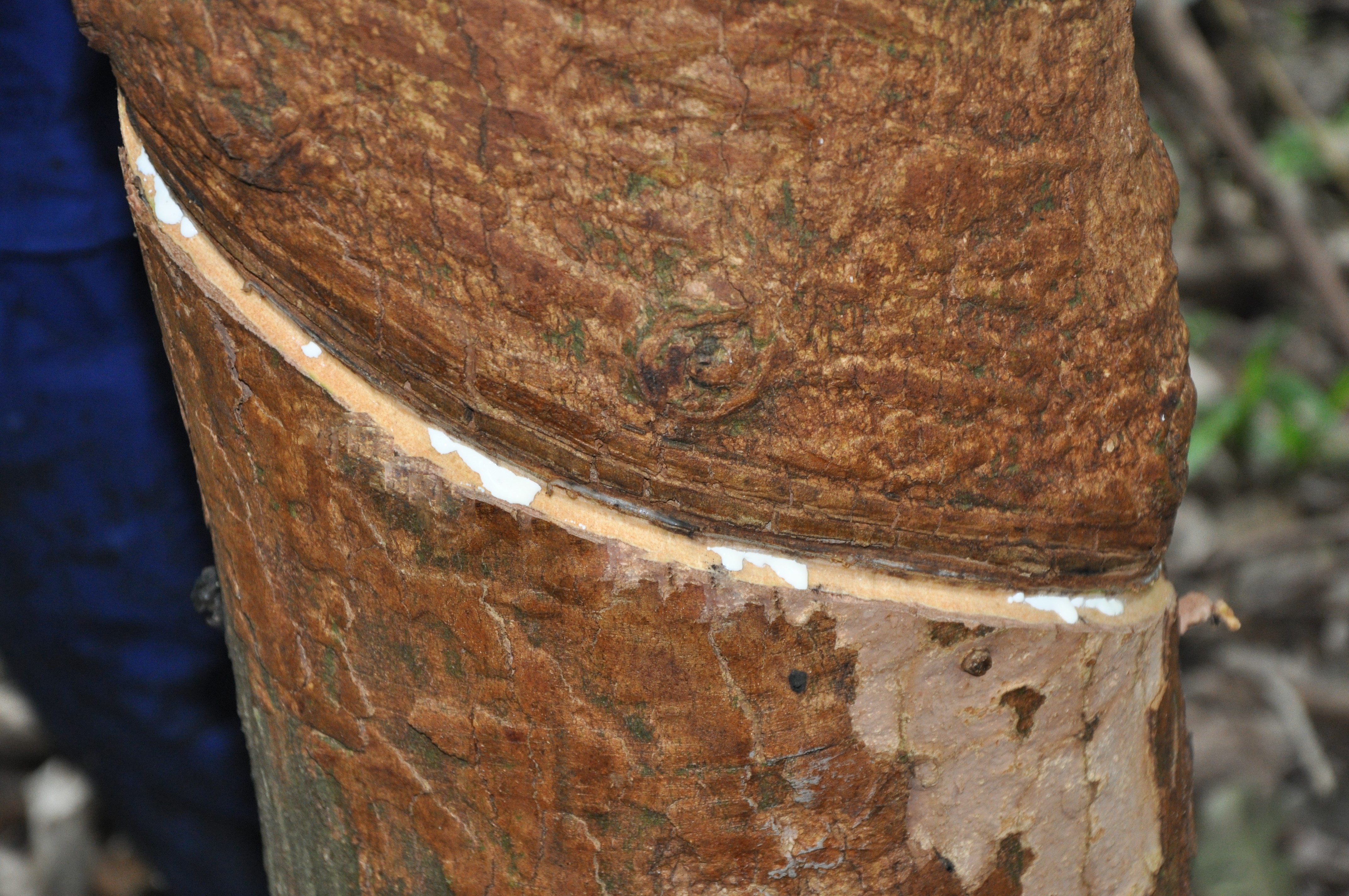

Supplement: S7 Data — (ZIP) [file pone.0297284.s007.zip › Level 4 Original Sample/4-33701-57-20141126-0015.JPG]

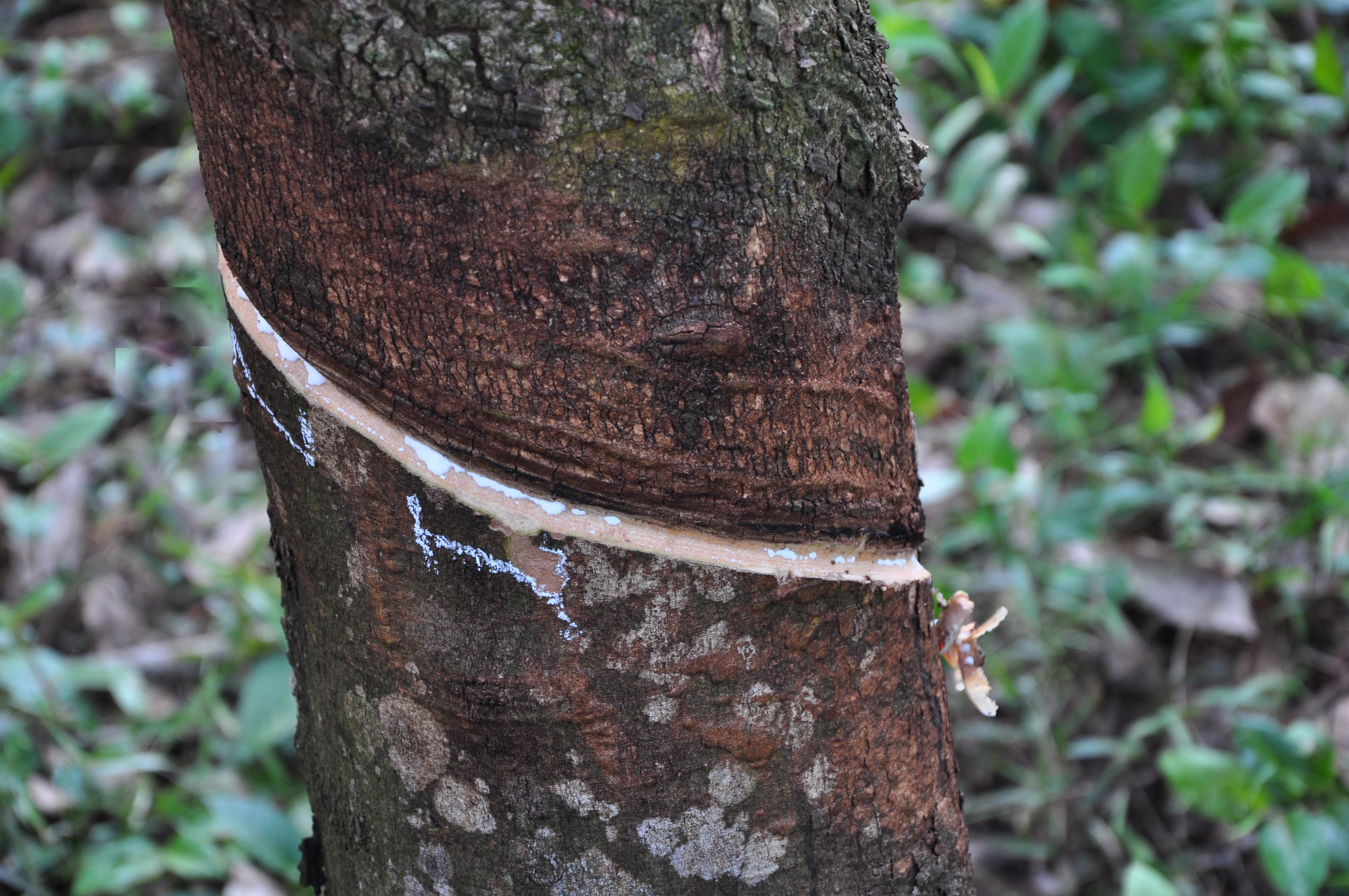

Supplement: S7 Data — (ZIP) [file pone.0297284.s007.zip › Level 4 Original Sample/4-33701-69-20141017-0528.JPG]

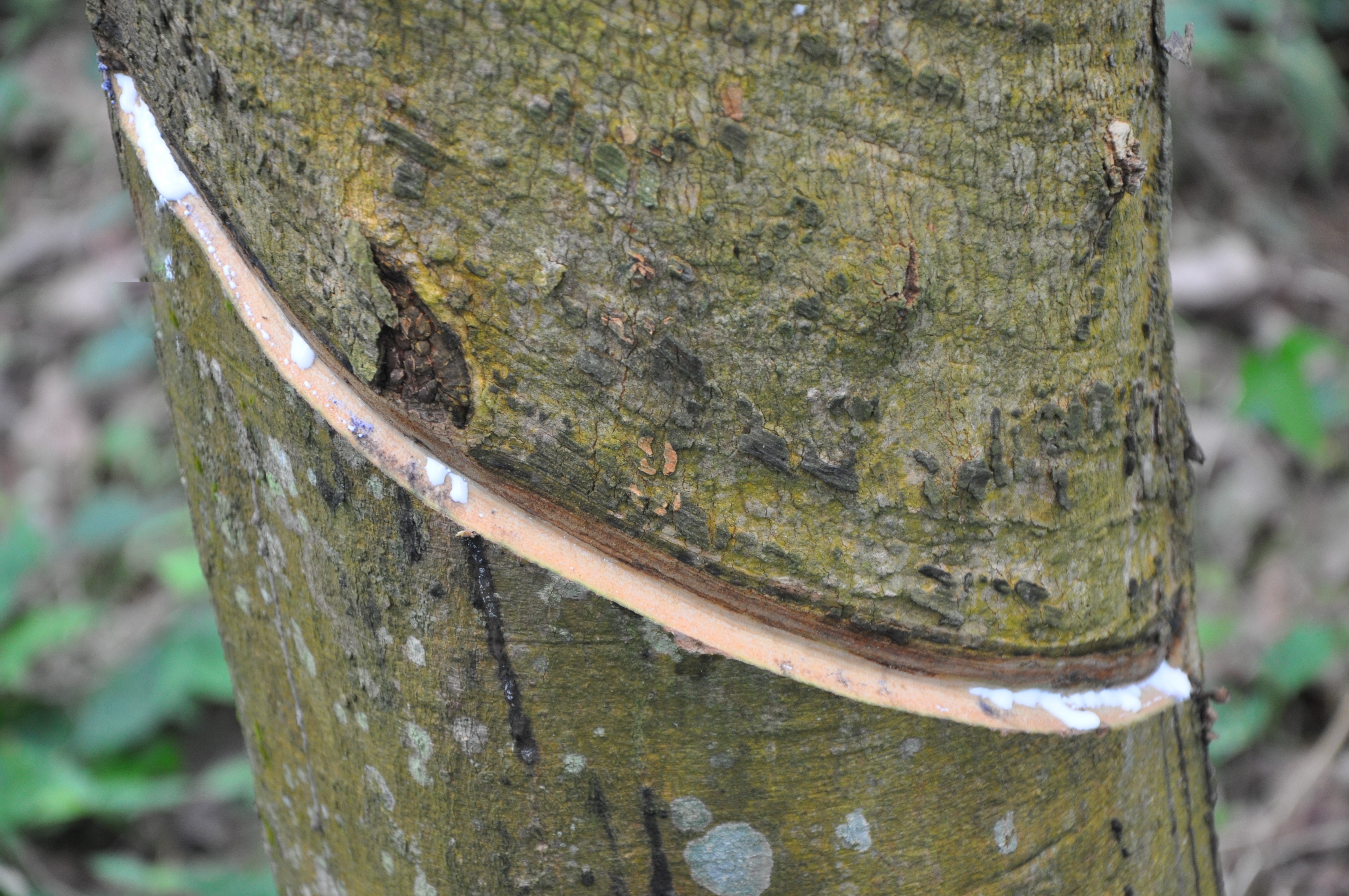

Supplement: S7 Data — (ZIP) [file pone.0297284.s007.zip › Level 4 Original Sample/4-33701-7-20141126-0076.JPG]

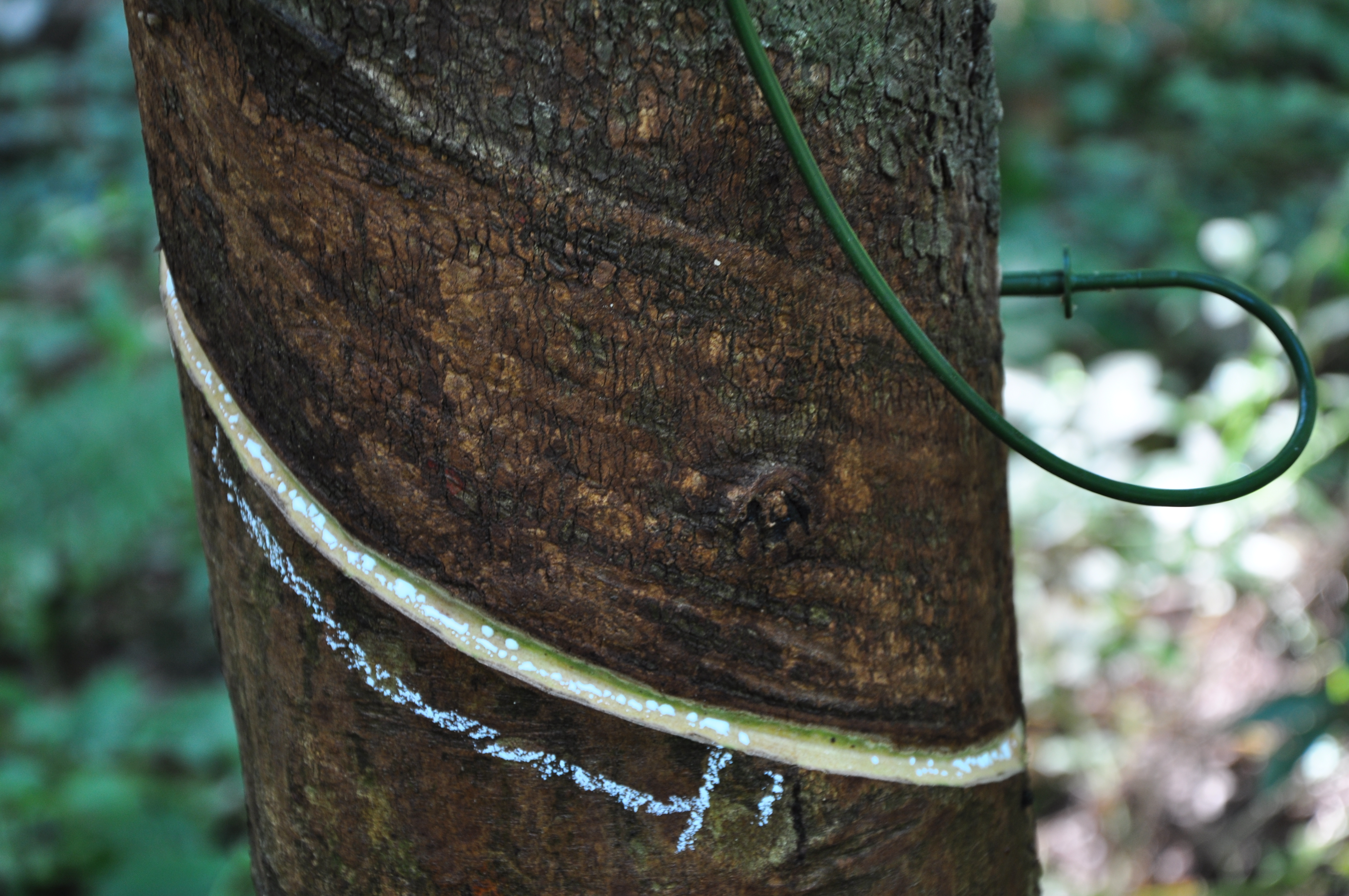

Supplement: S7 Data — (ZIP) [file pone.0297284.s007.zip › Level 4 Original Sample/4-33702-179-20140707-0153.JPG]

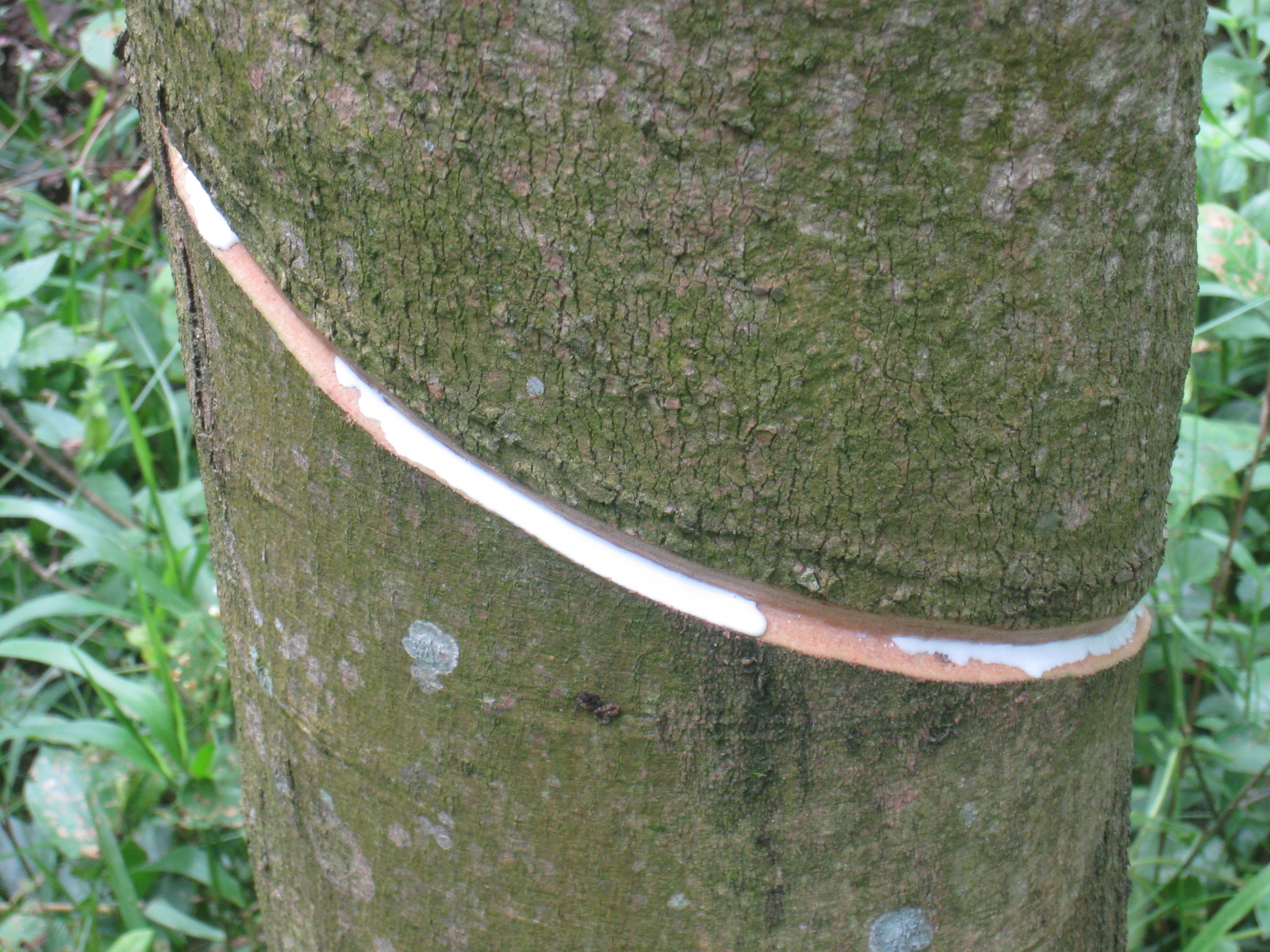

Supplement: S7 Data — (ZIP) [file pone.0297284.s007.zip › Level 4 Original Sample/4-33702-32-20140512-0041.JPG]

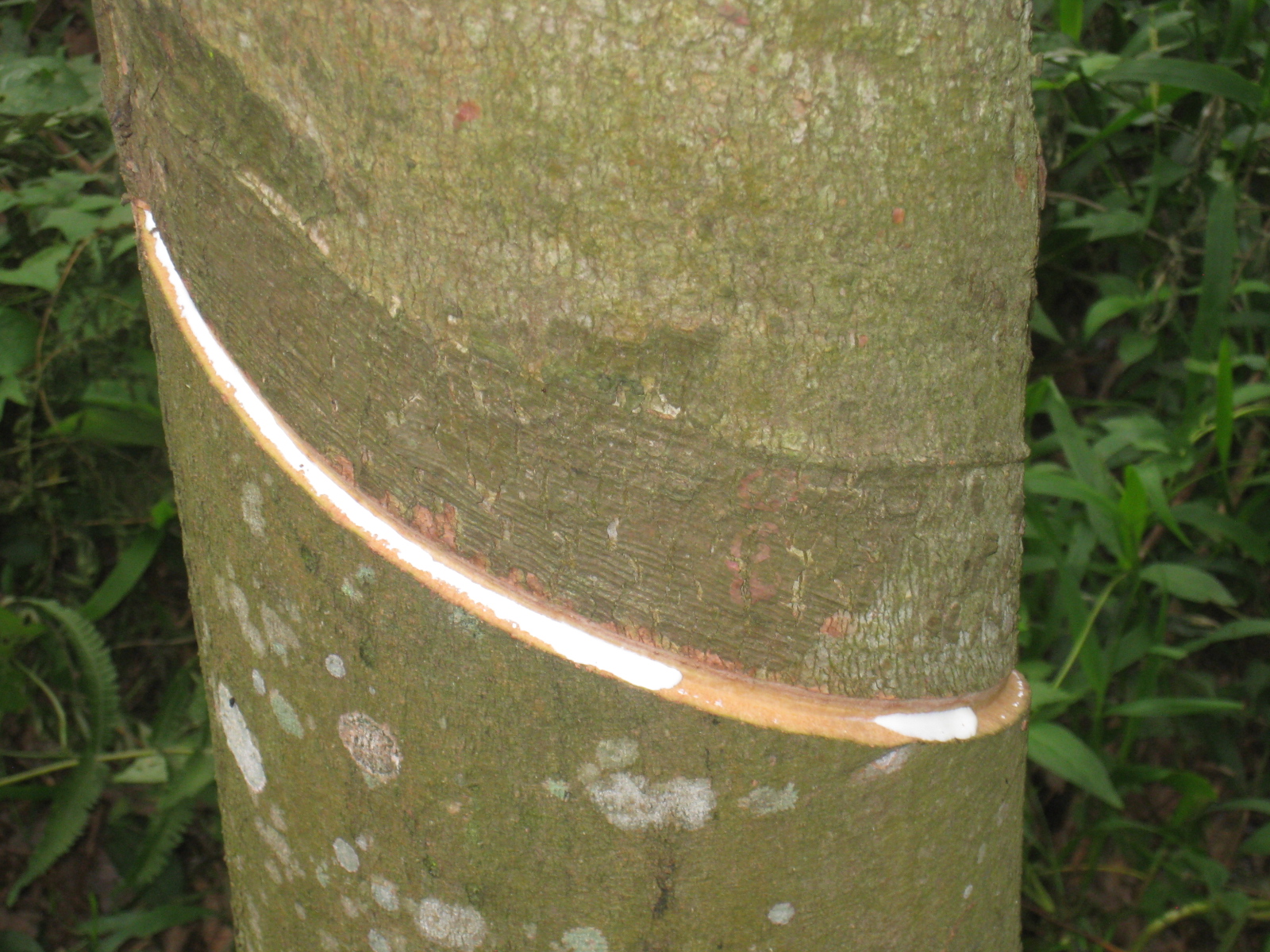

Supplement: S7 Data — (ZIP) [file pone.0297284.s007.zip › Level 4 Original Sample/4-33702-62-20140512-0055.JPG]

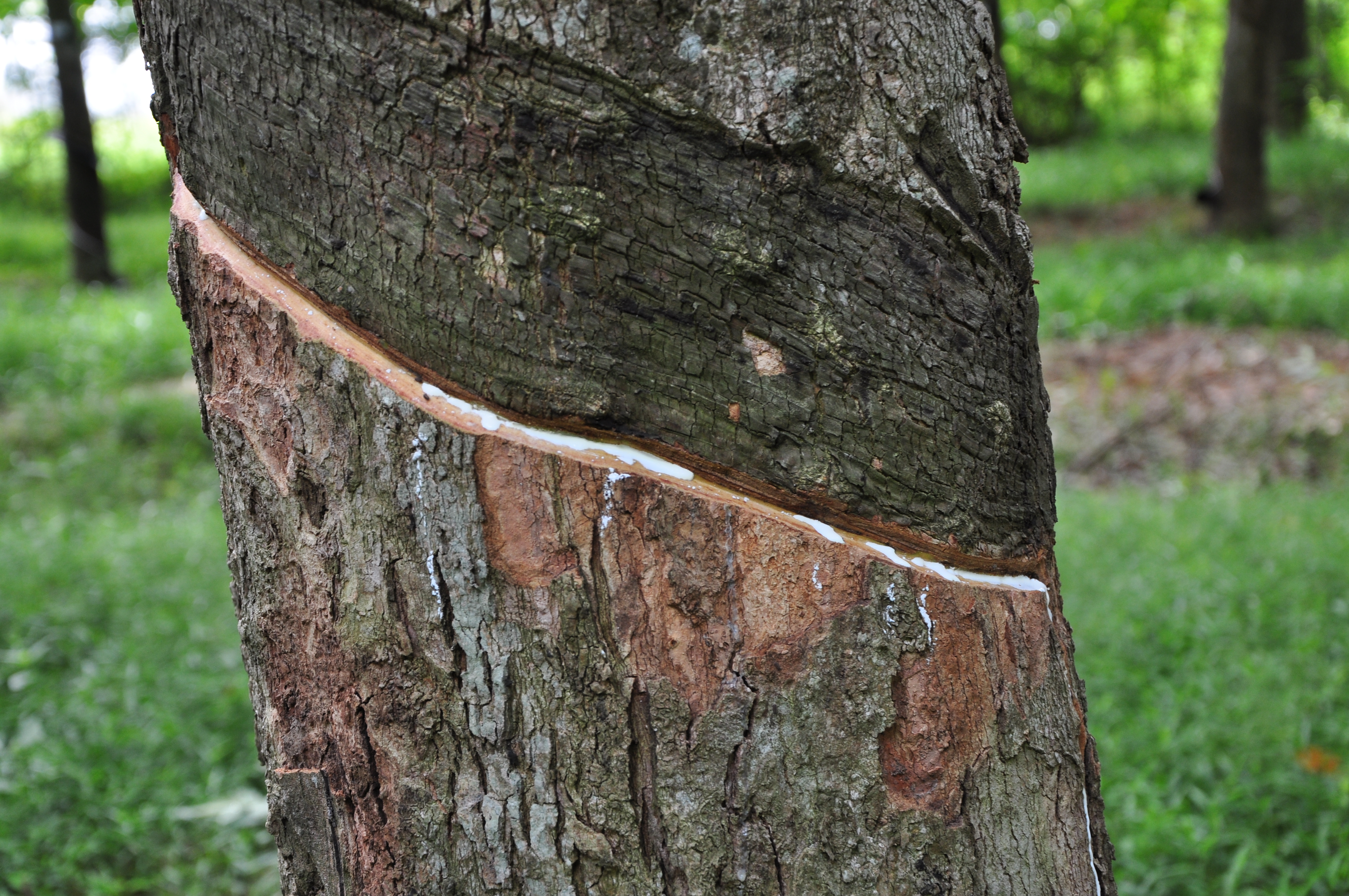

Supplement: S7 Data — (ZIP) [file pone.0297284.s007.zip › Level 4 Original Sample/4-62001-169-20140609-0417.JPG]

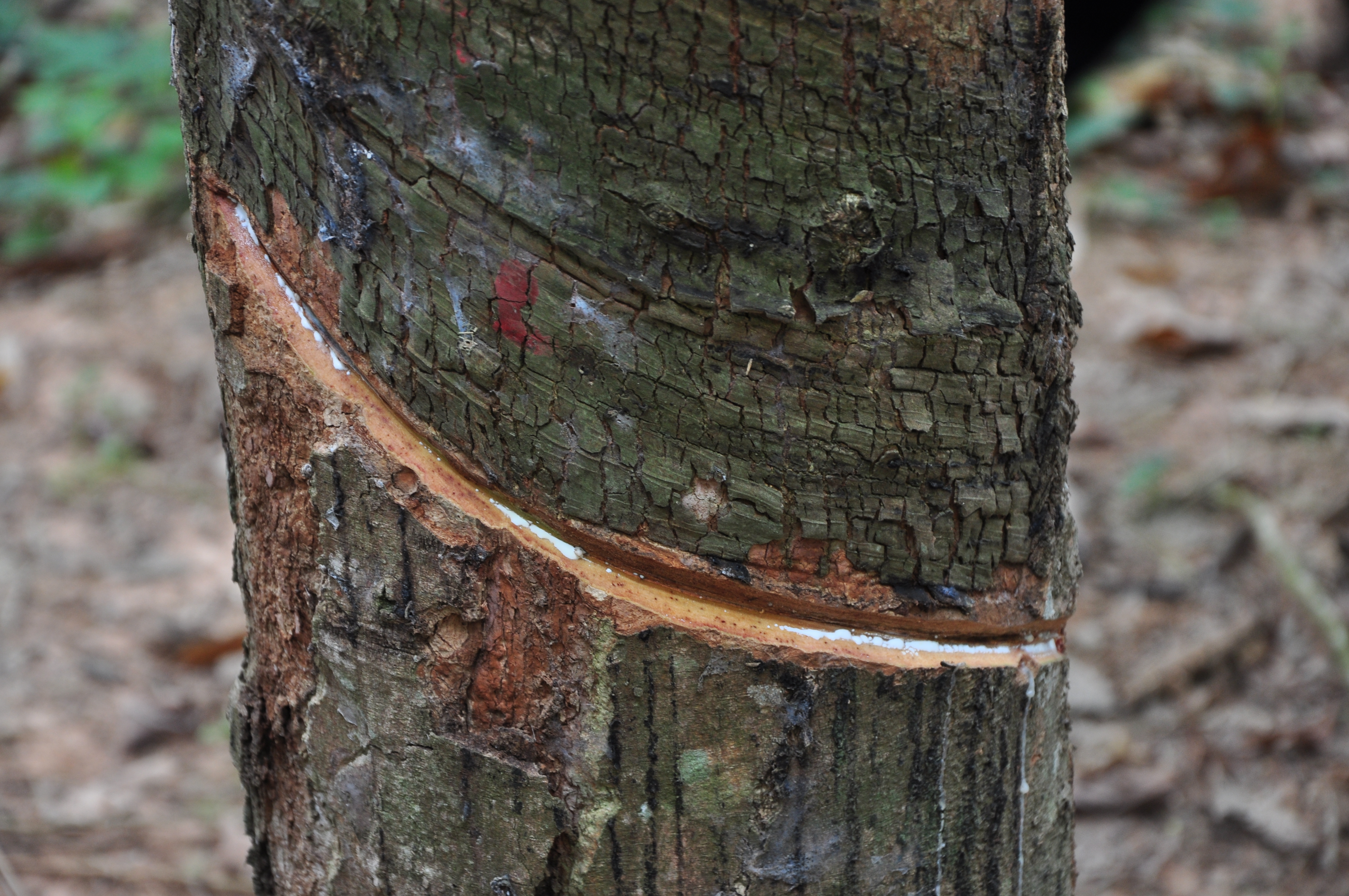

Supplement: S7 Data — (ZIP) [file pone.0297284.s007.zip › Level 4 Original Sample/4-62001-433-20140609-0555.JPG]

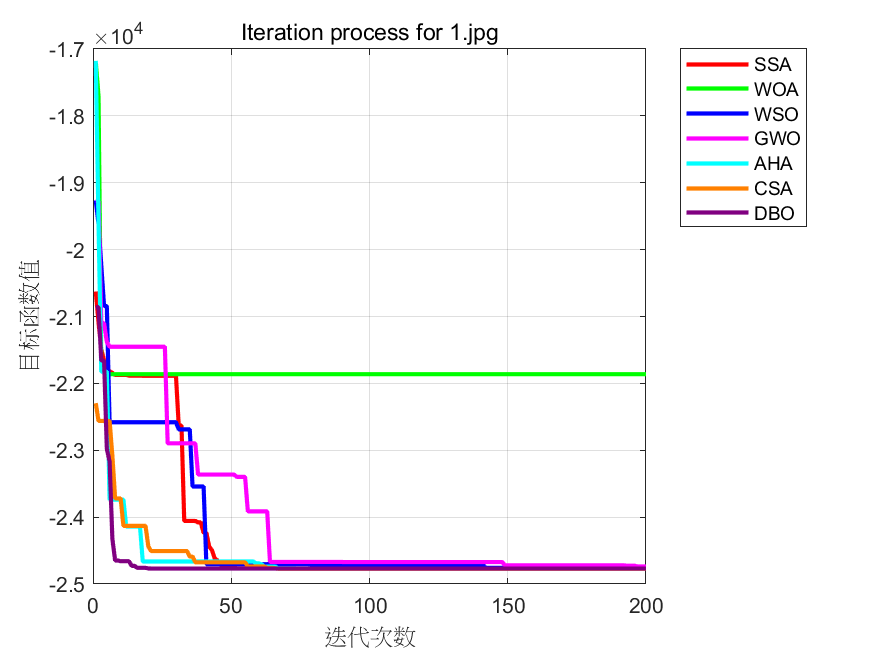

Supplement: S8 Data — (ZIP) [file pone.0297284.s008.zip › Level 4 processed Sample/iteration/1.jpg_iteration.png]

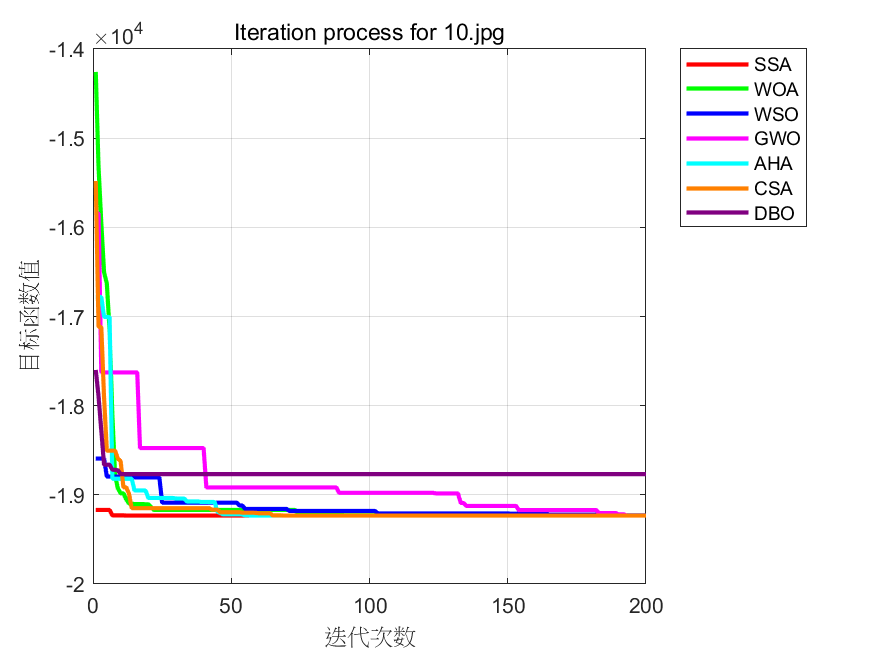

Supplement: S8 Data — (ZIP) [file pone.0297284.s008.zip › Level 4 processed Sample/iteration/10.jpg_iteration.png]

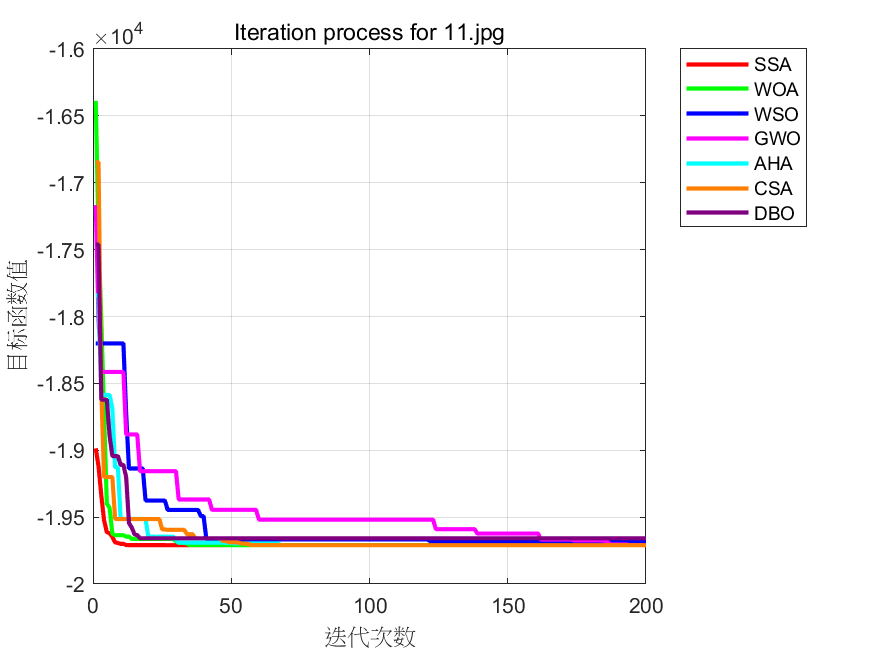

Supplement: S8 Data — (ZIP) [file pone.0297284.s008.zip › Level 4 processed Sample/iteration/11.jpg_iteration.png]

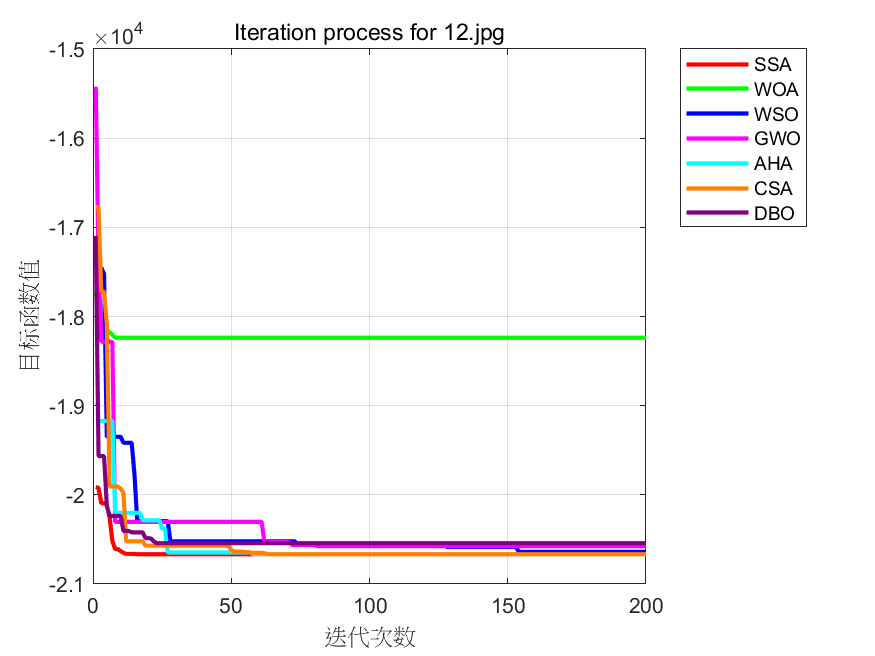

Supplement: S8 Data — (ZIP) [file pone.0297284.s008.zip › Level 4 processed Sample/iteration/12.jpg_iteration.png]

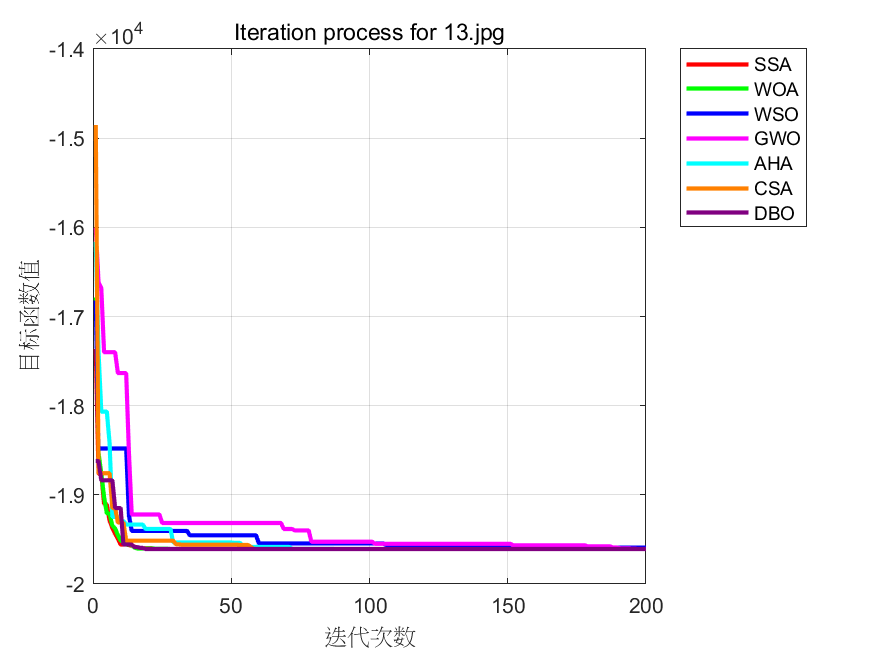

Supplement: S8 Data — (ZIP) [file pone.0297284.s008.zip › Level 4 processed Sample/iteration/13.jpg_iteration.png]

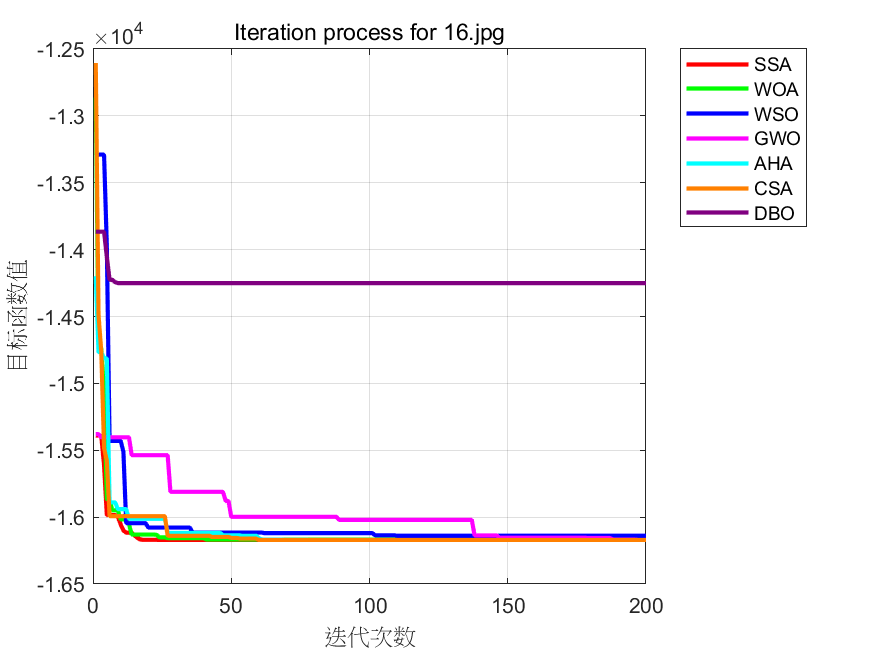

Supplement: S8 Data — (ZIP) [file pone.0297284.s008.zip › Level 4 processed Sample/iteration/16.jpg_iteration.png]

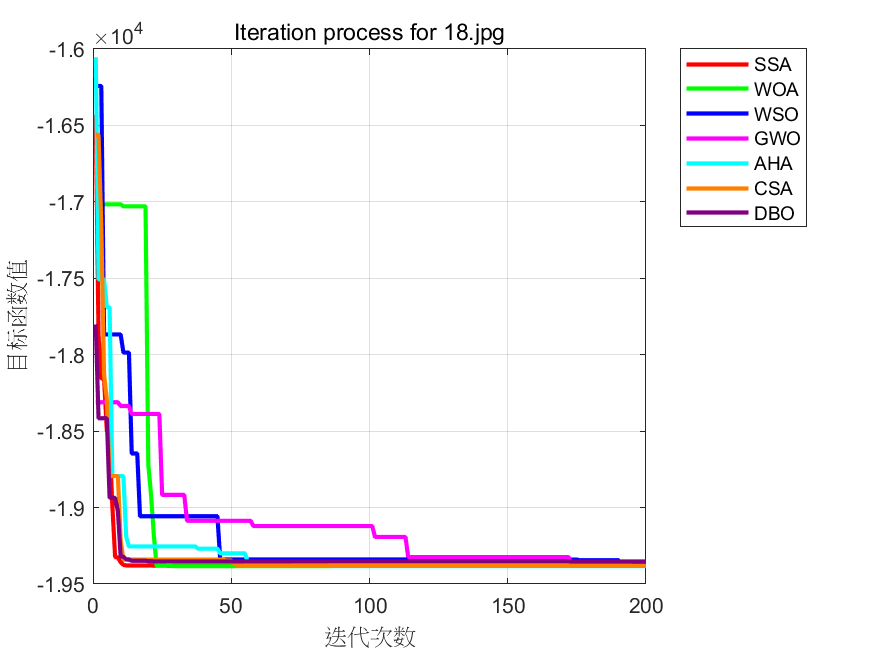

Supplement: S8 Data — (ZIP) [file pone.0297284.s008.zip › Level 4 processed Sample/iteration/18.jpg_iteration.png]

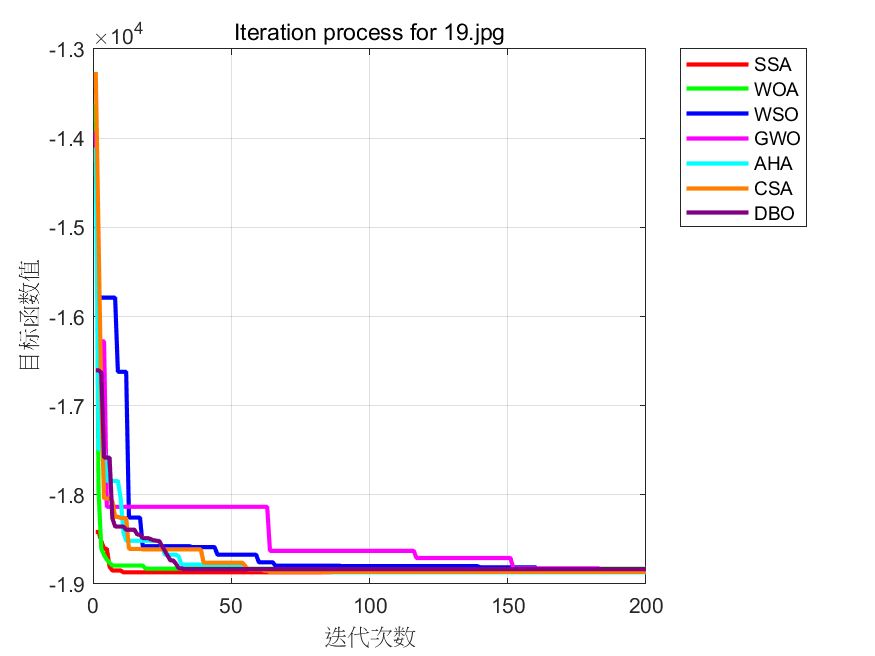

Supplement: S8 Data — (ZIP) [file pone.0297284.s008.zip › Level 4 processed Sample/iteration/19.jpg_iteration.png]

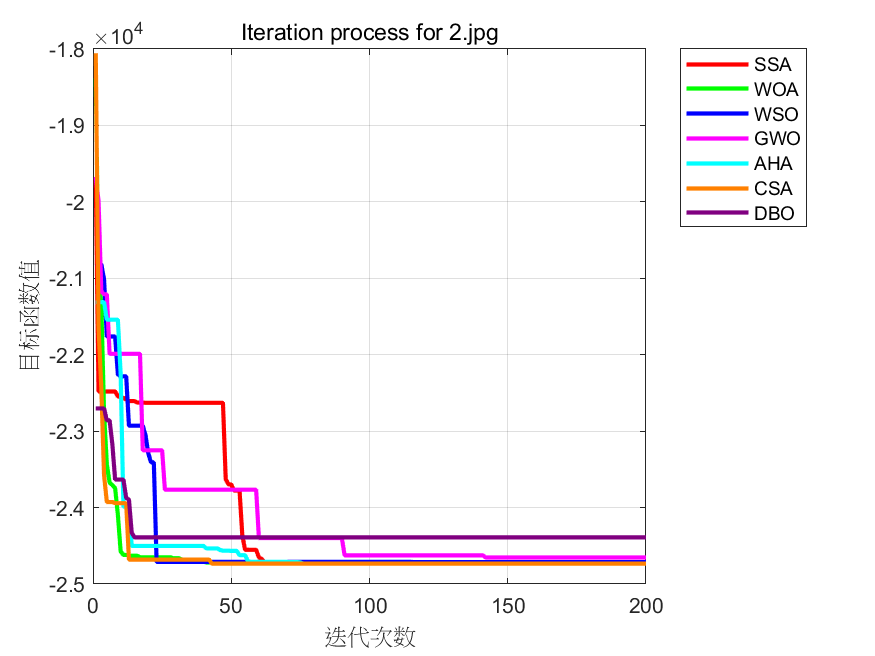

Supplement: S8 Data — (ZIP) [file pone.0297284.s008.zip › Level 4 processed Sample/iteration/2.jpg_iteration.png]

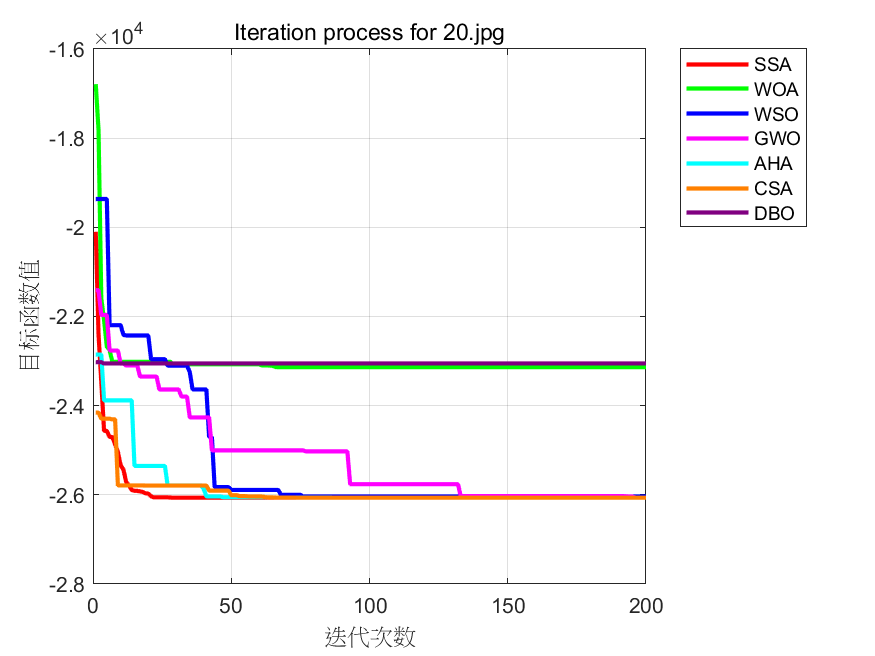

Supplement: S8 Data — (ZIP) [file pone.0297284.s008.zip › Level 4 processed Sample/iteration/20.jpg_iteration.png]

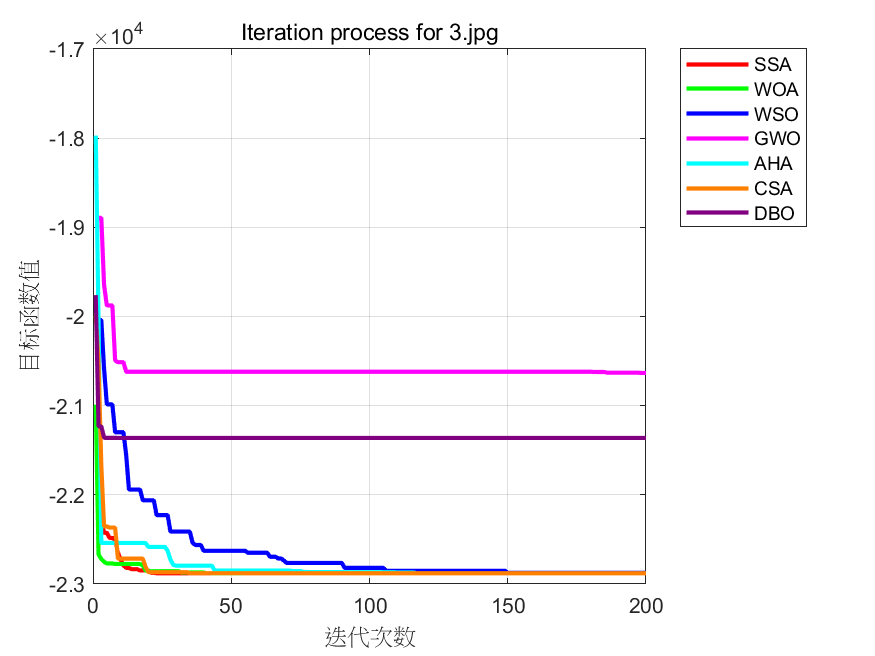

Supplement: S8 Data — (ZIP) [file pone.0297284.s008.zip › Level 4 processed Sample/iteration/3.jpg_iteration.png]

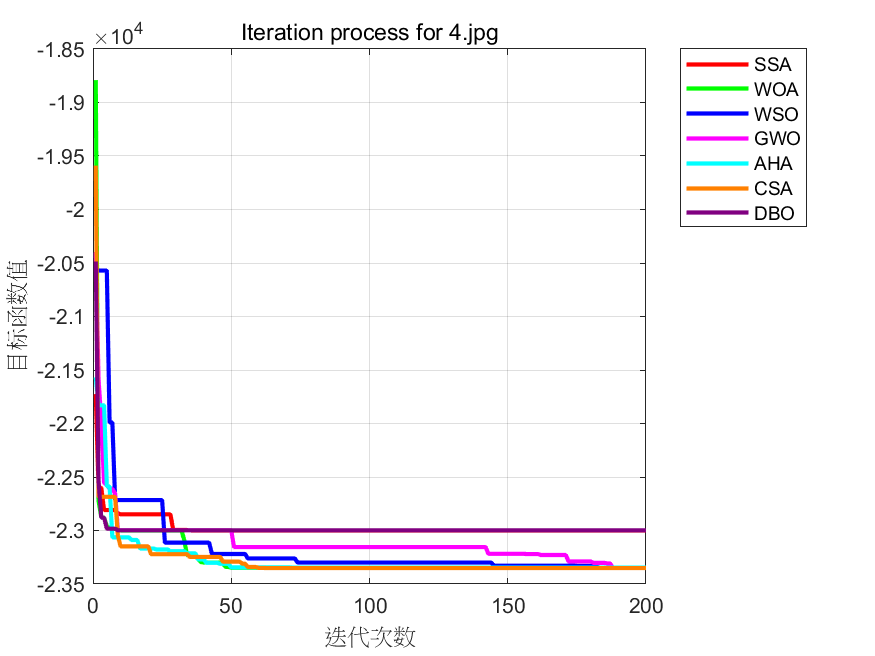

Supplement: S8 Data — (ZIP) [file pone.0297284.s008.zip › Level 4 processed Sample/iteration/4.jpg_iteration.png]

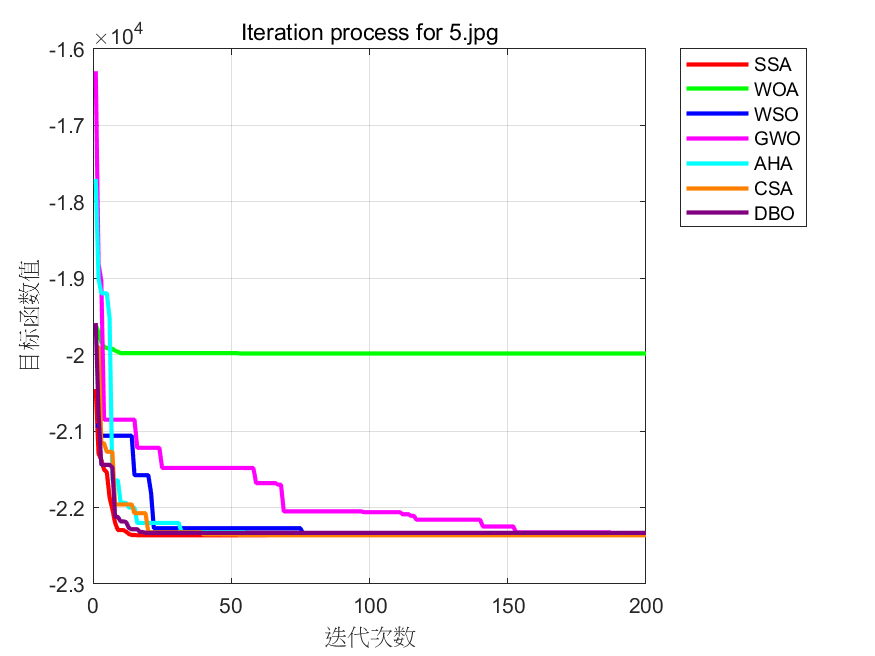

Supplement: S8 Data — (ZIP) [file pone.0297284.s008.zip › Level 4 processed Sample/iteration/5.jpg_iteration.png]

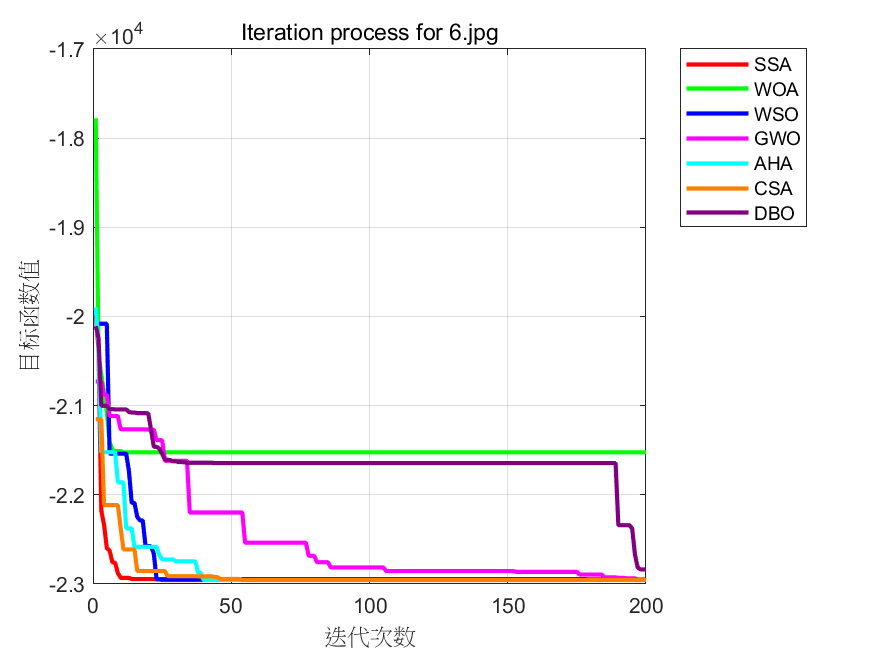

Supplement: S8 Data — (ZIP) [file pone.0297284.s008.zip › Level 4 processed Sample/iteration/6.jpg_iteration.png]

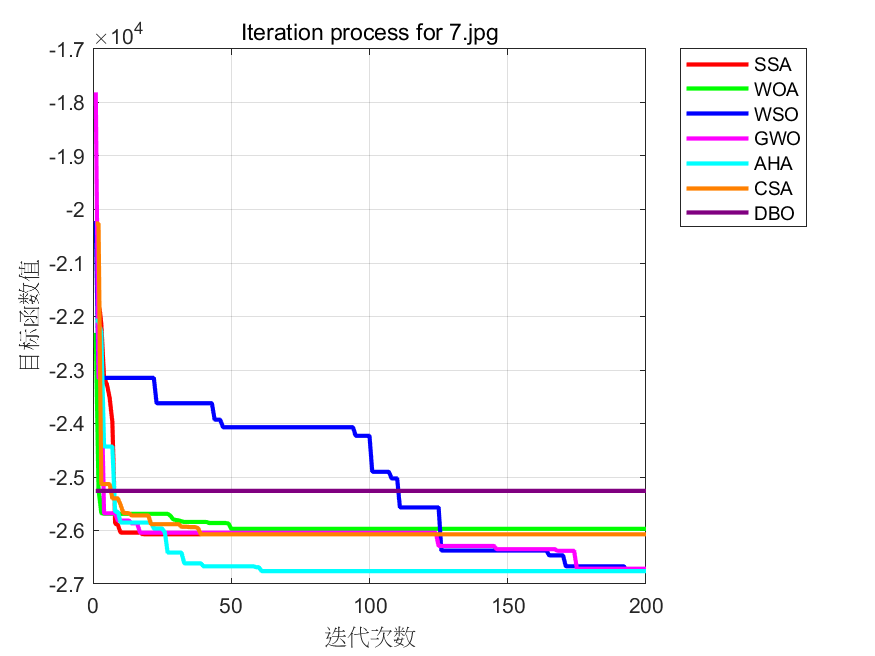

Supplement: S8 Data — (ZIP) [file pone.0297284.s008.zip › Level 4 processed Sample/iteration/7.jpg_iteration.png]

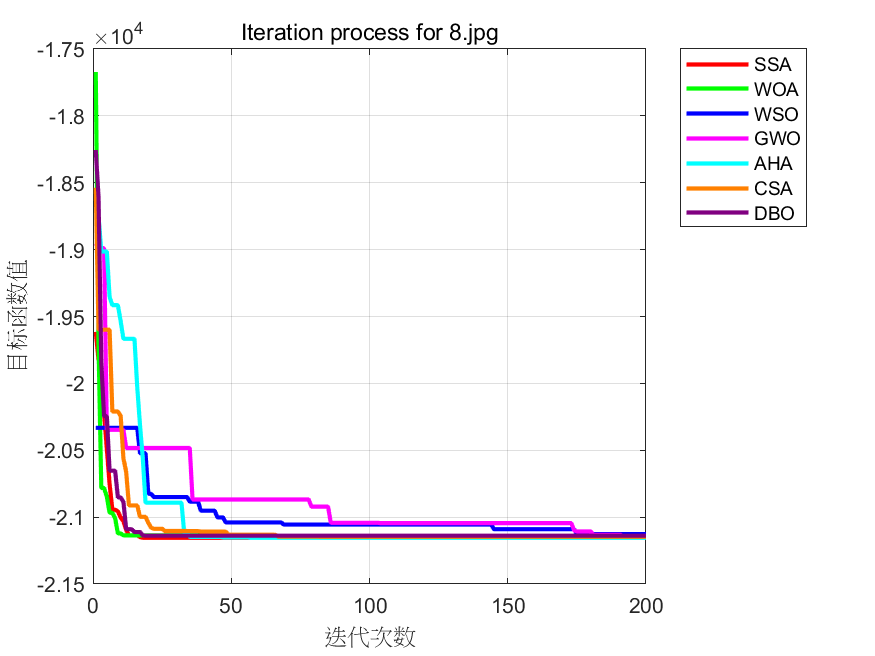

Supplement: S8 Data — (ZIP) [file pone.0297284.s008.zip › Level 4 processed Sample/iteration/8.jpg_iteration.png]

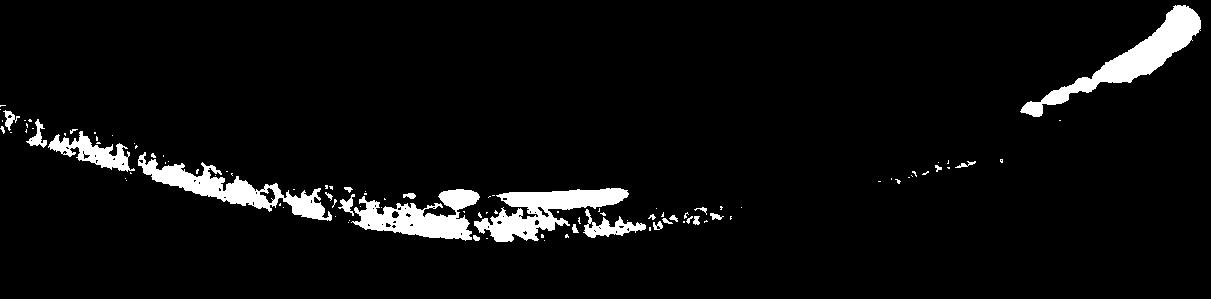

Supplement: S8 Data — (ZIP) [file pone.0297284.s008.zip › Level 4 processed Sample/processed_1/latex/AHA_latex.jpg]

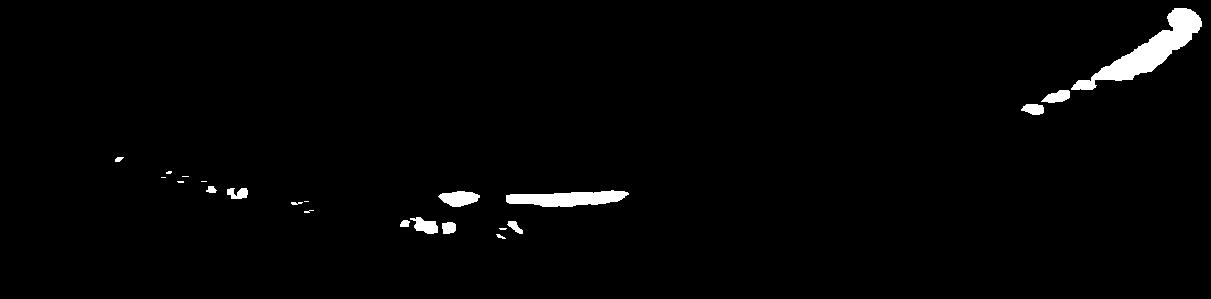

Supplement: S8 Data — (ZIP) [file pone.0297284.s008.zip › Level 4 processed Sample/processed_1/latex/DBO_latex.jpg]

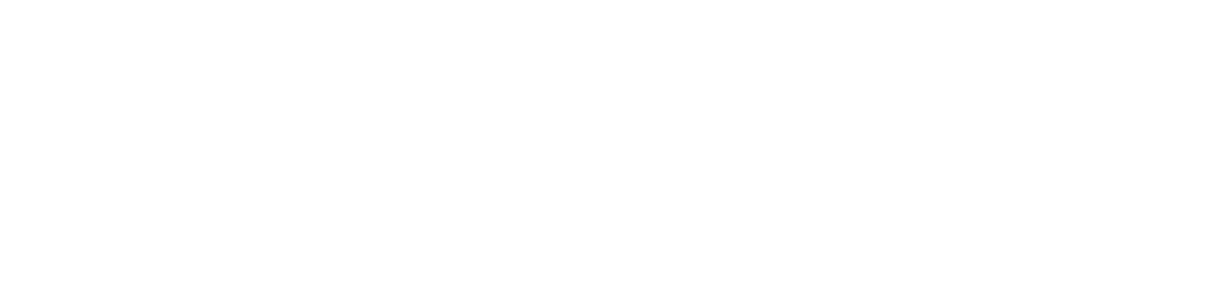

Supplement: S8 Data — (ZIP) [file pone.0297284.s008.zip › Level 4 processed Sample/processed_1/latex/OTSU_latex.jpg]

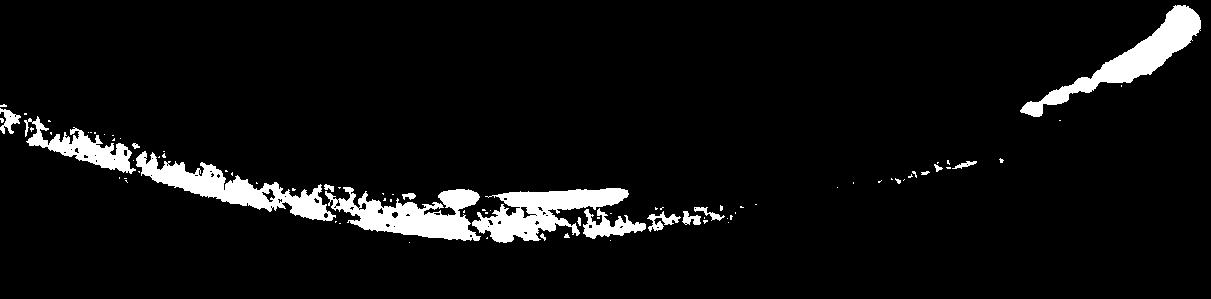

Supplement: S8 Data — (ZIP) [file pone.0297284.s008.zip › Level 4 processed Sample/processed_1/latex/WOA_latex.jpg]

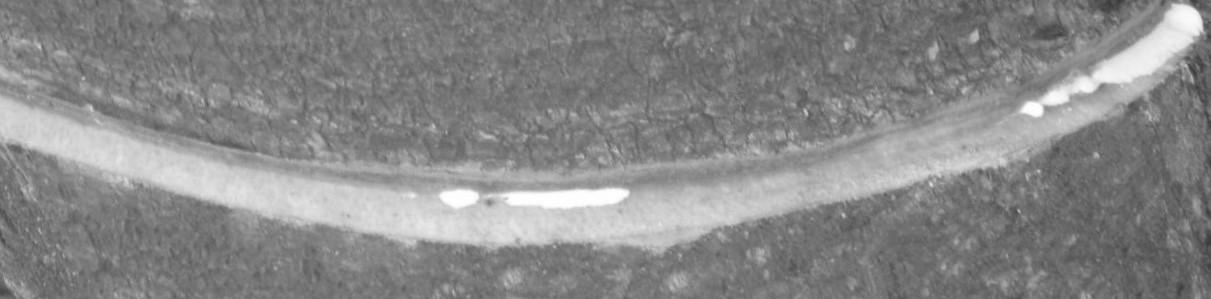

Supplement: S8 Data — (ZIP) [file pone.0297284.s008.zip › Level 4 processed Sample/processed_1/original_image.jpg]

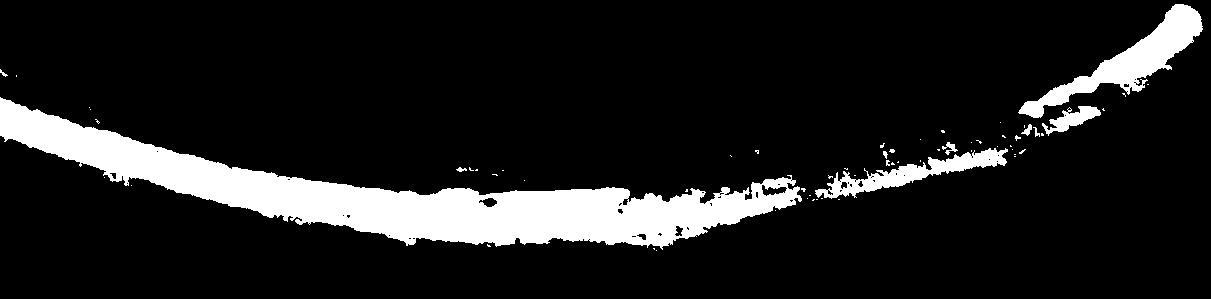

Supplement: S8 Data — (ZIP) [file pone.0297284.s008.zip › Level 4 processed Sample/processed_1/scar/AHA_scar.jpg]

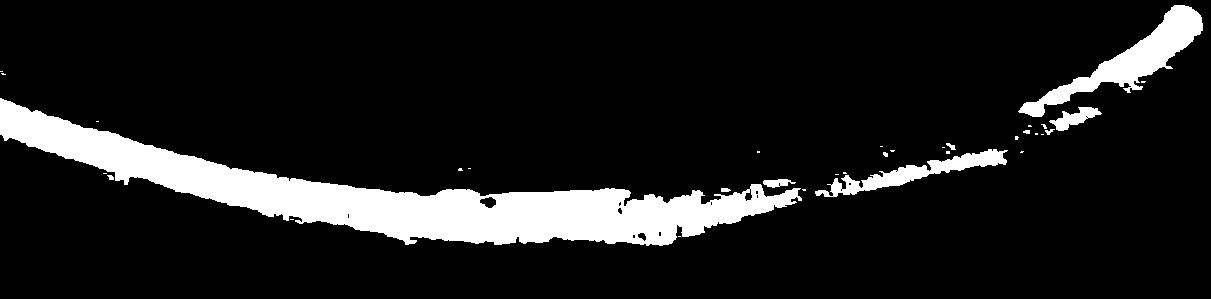

Supplement: S8 Data — (ZIP) [file pone.0297284.s008.zip › Level 4 processed Sample/processed_1/scar/DBO_scar.jpg]

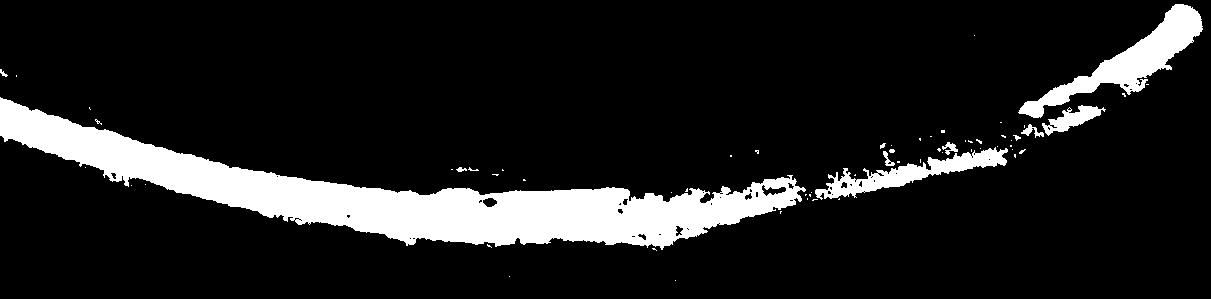

Supplement: S8 Data — (ZIP) [file pone.0297284.s008.zip › Level 4 processed Sample/processed_1/scar/GWO_scar.jpg]

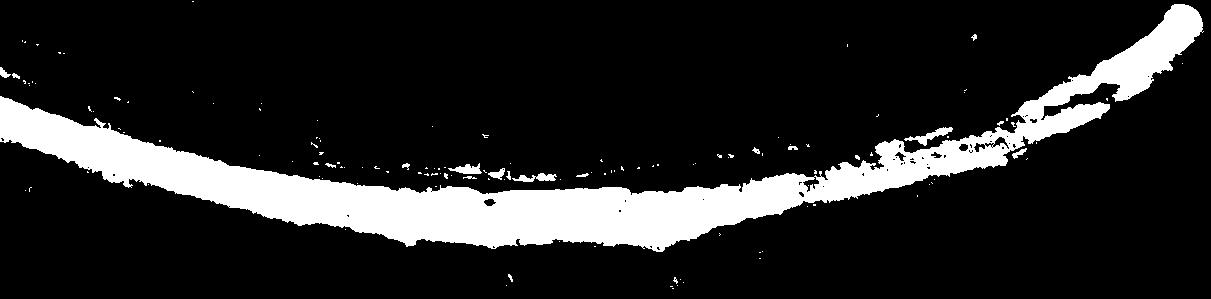

Supplement: S8 Data — (ZIP) [file pone.0297284.s008.zip › Level 4 processed Sample/processed_1/scar/WOA_scar.jpg]

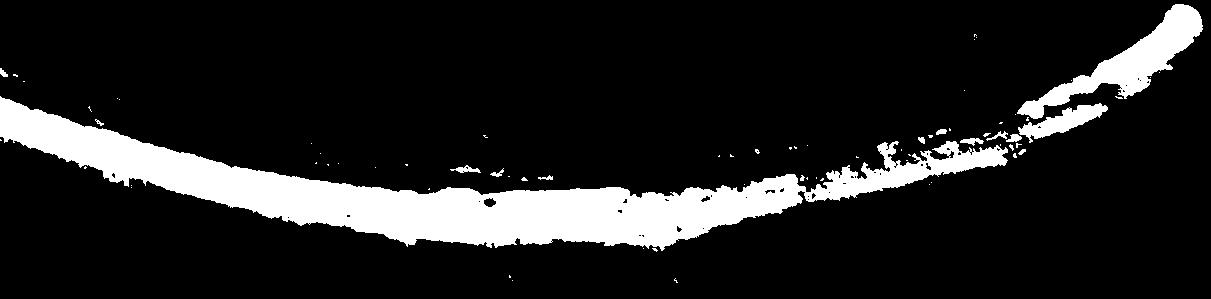

Supplement: S8 Data — (ZIP) [file pone.0297284.s008.zip › Level 4 processed Sample/processed_1/scar/WSO_scar.jpg]

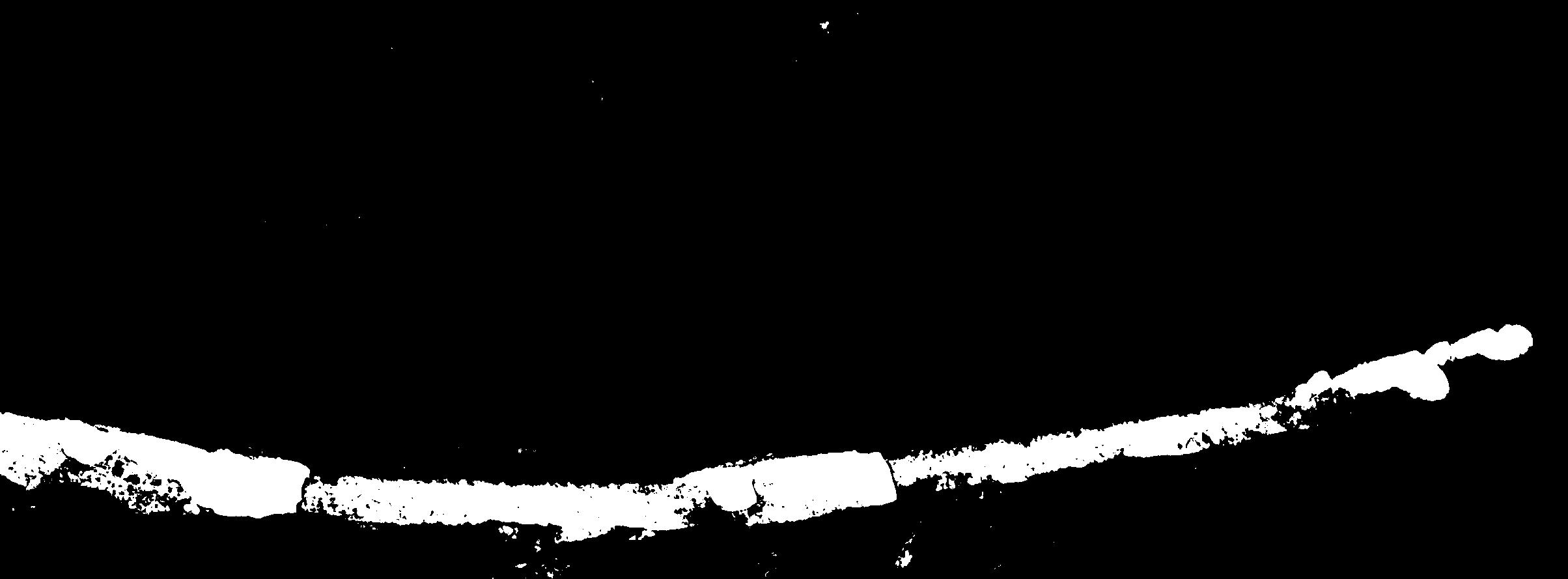

Supplement: S8 Data — (ZIP) [file pone.0297284.s008.zip › Level 4 processed Sample/processed_10/latex/AHA_latex.jpg]

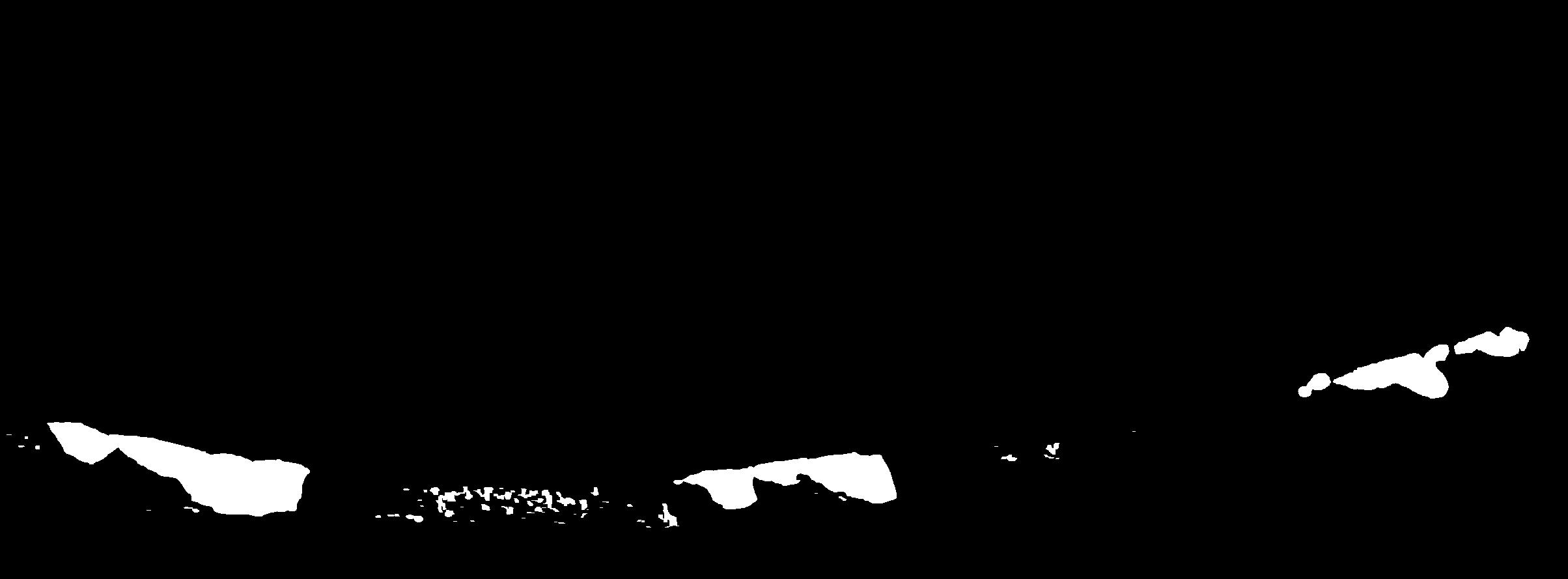

Supplement: S8 Data — (ZIP) [file pone.0297284.s008.zip › Level 4 processed Sample/processed_10/latex/DBO_latex.jpg]

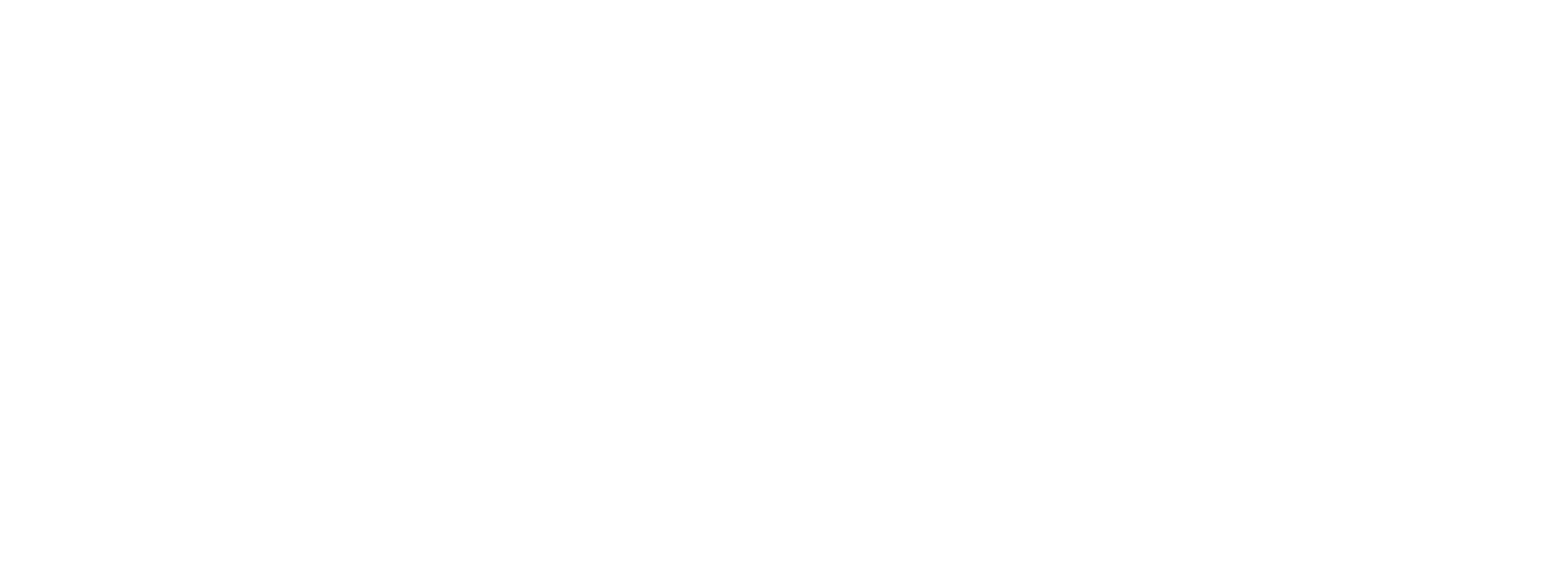

Supplement: S8 Data — (ZIP) [file pone.0297284.s008.zip › Level 4 processed Sample/processed_10/latex/OTSU_latex.jpg]

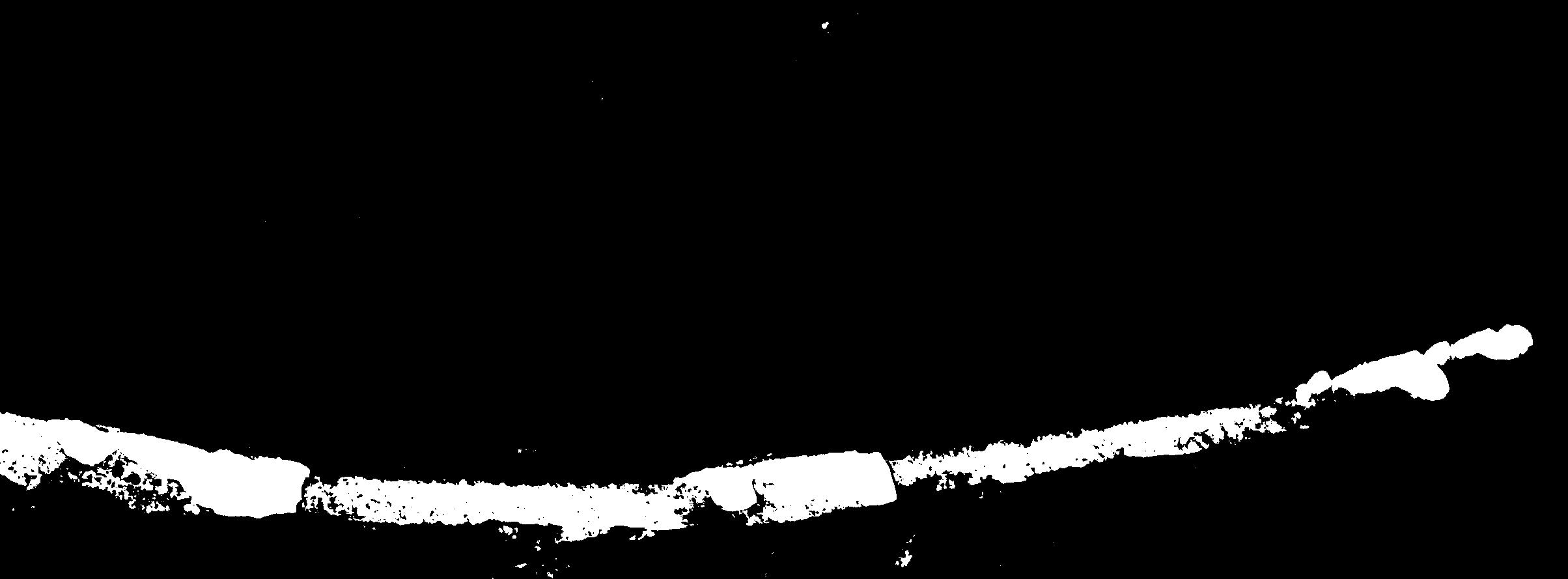

Supplement: S8 Data — (ZIP) [file pone.0297284.s008.zip › Level 4 processed Sample/processed_10/latex/WSO_latex.jpg]

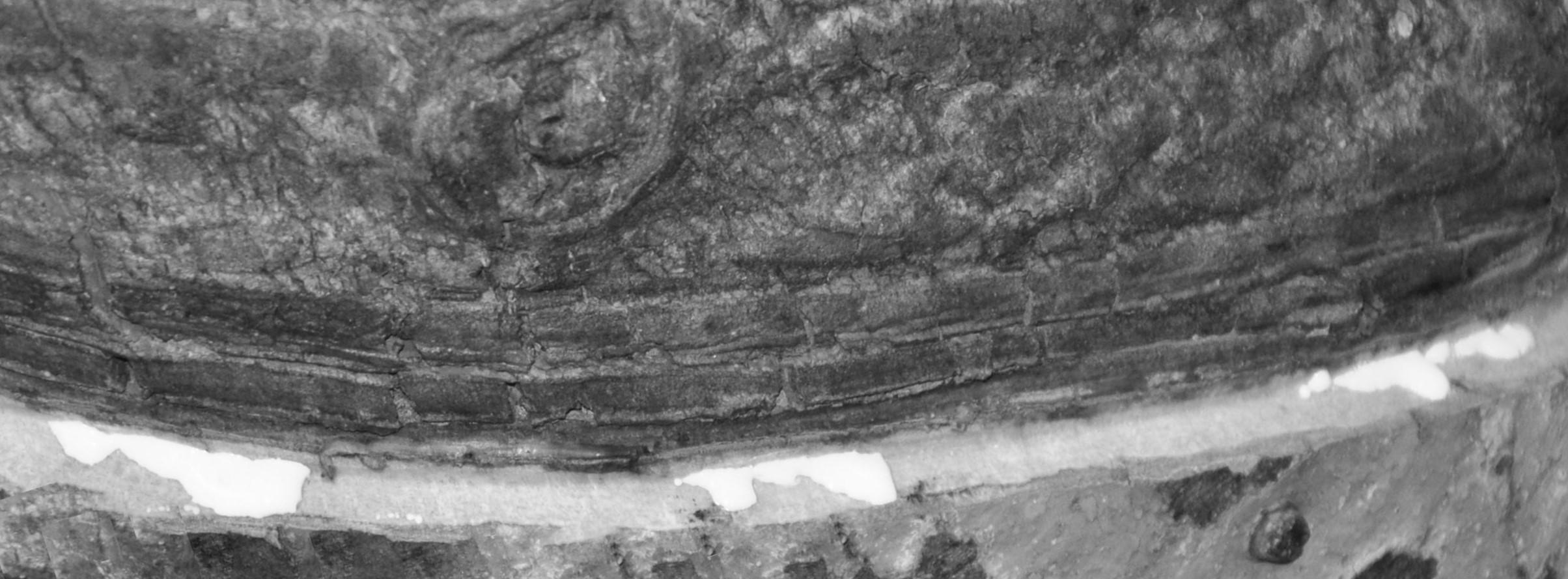

Supplement: S8 Data — (ZIP) [file pone.0297284.s008.zip › Level 4 processed Sample/processed_10/original_image.jpg]

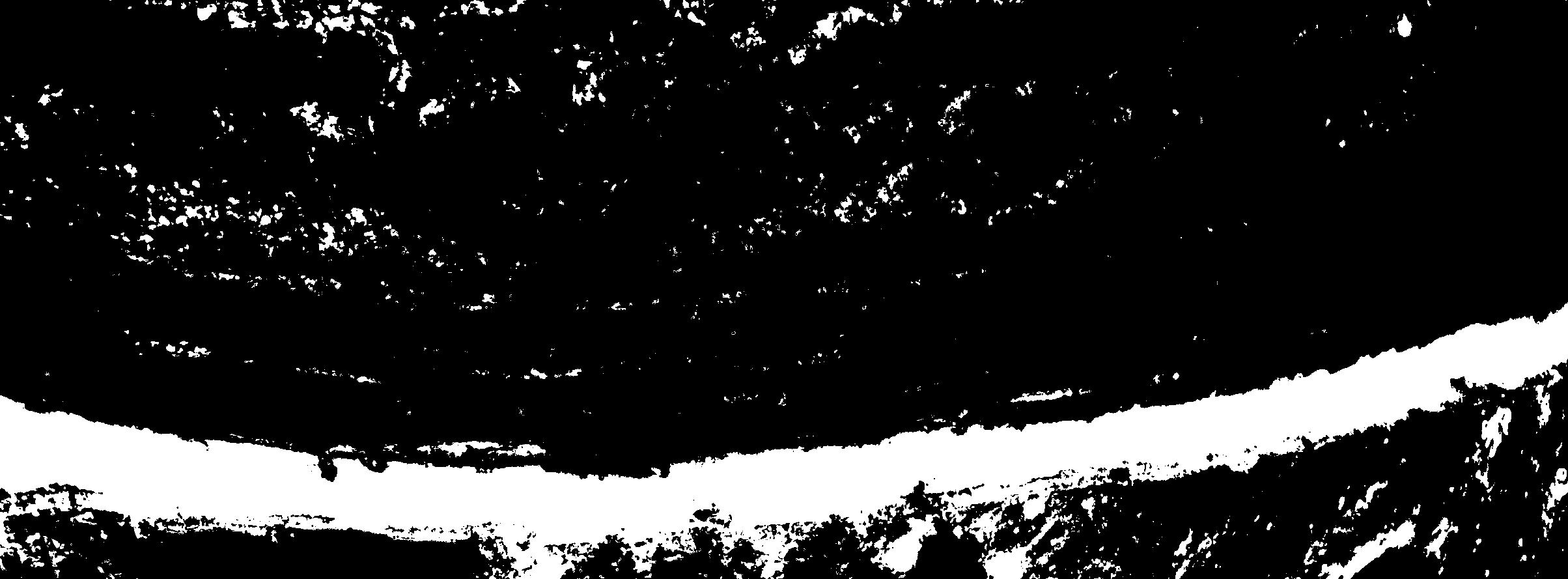

Supplement: S8 Data — (ZIP) [file pone.0297284.s008.zip › Level 4 processed Sample/processed_10/scar/AHA_scar.jpg]

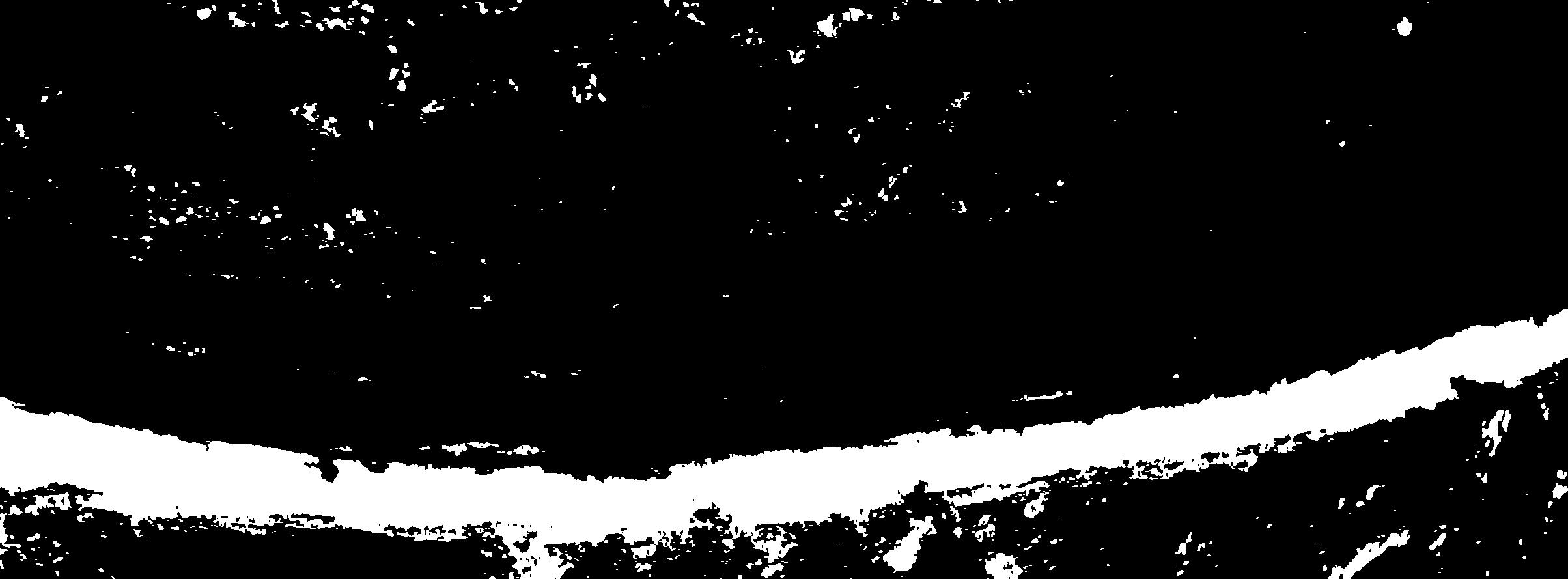

Supplement: S8 Data — (ZIP) [file pone.0297284.s008.zip › Level 4 processed Sample/processed_10/scar/DBO_scar.jpg]

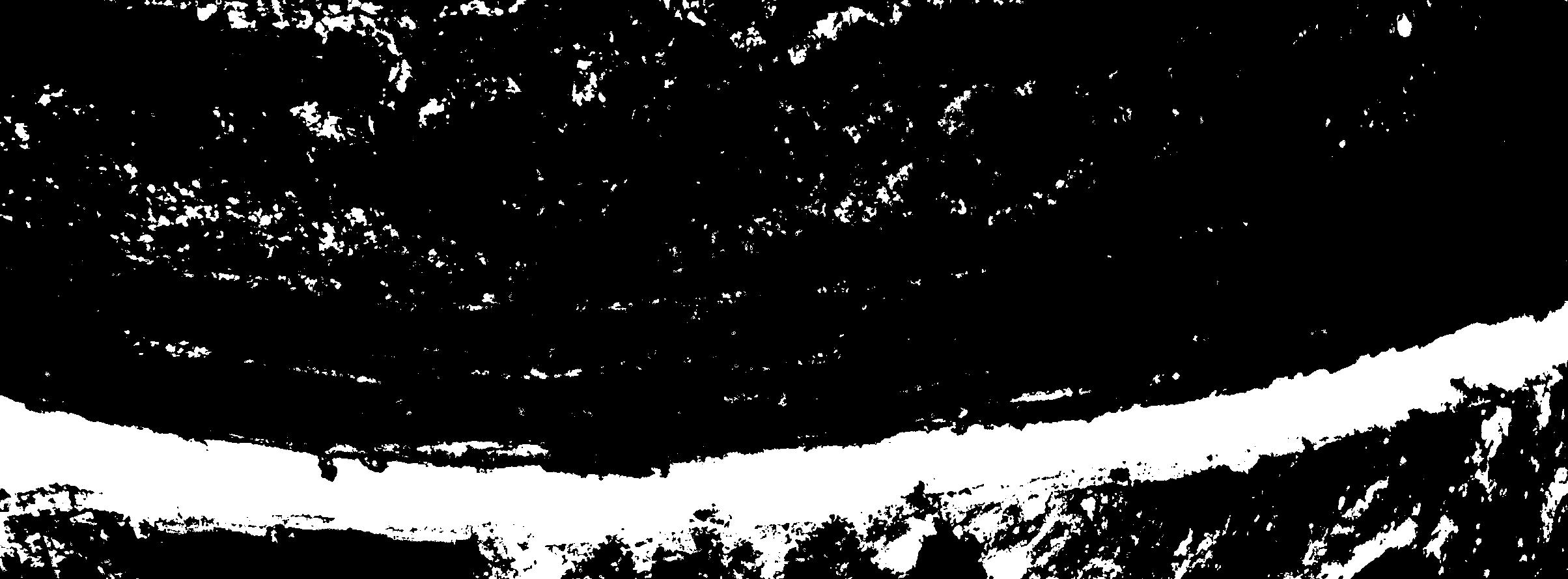

Supplement: S8 Data — (ZIP) [file pone.0297284.s008.zip › Level 4 processed Sample/processed_10/scar/WSO_scar.jpg]

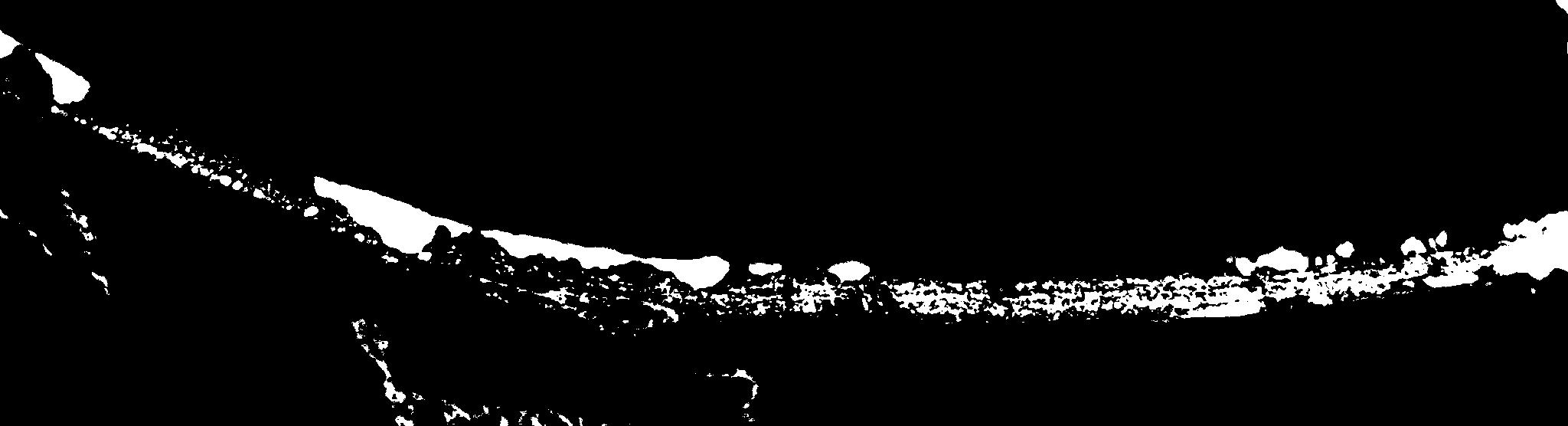

Supplement: S8 Data — (ZIP) [file pone.0297284.s008.zip › Level 4 processed Sample/processed_11/latex/AHA_latex.jpg]

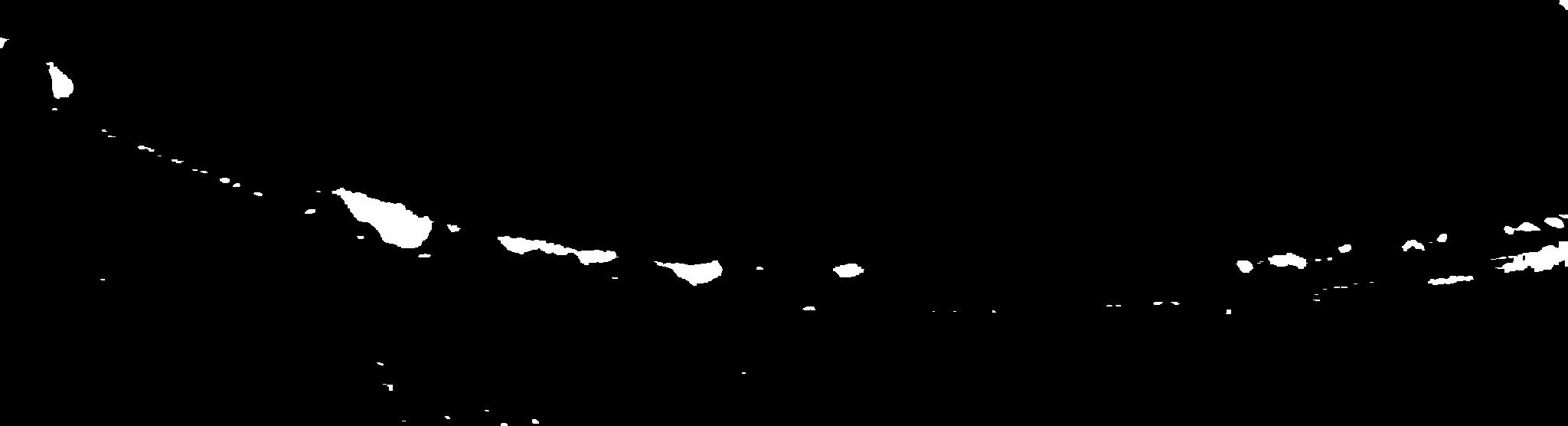

Supplement: S8 Data — (ZIP) [file pone.0297284.s008.zip › Level 4 processed Sample/processed_11/latex/DBO_latex.jpg]

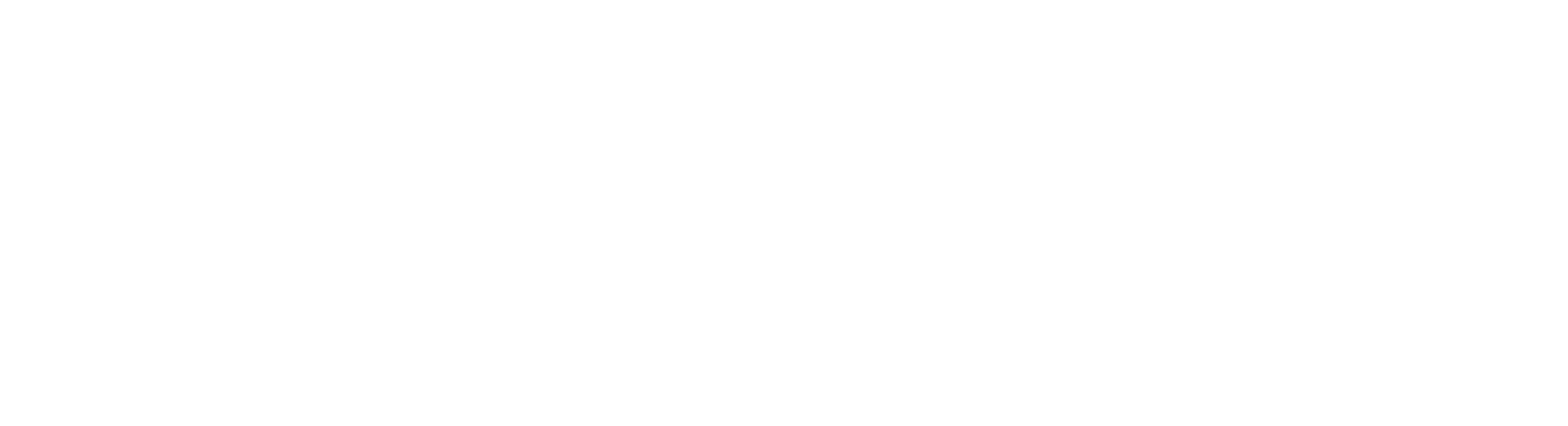

Supplement: S8 Data — (ZIP) [file pone.0297284.s008.zip › Level 4 processed Sample/processed_11/latex/OTSU_latex.jpg]

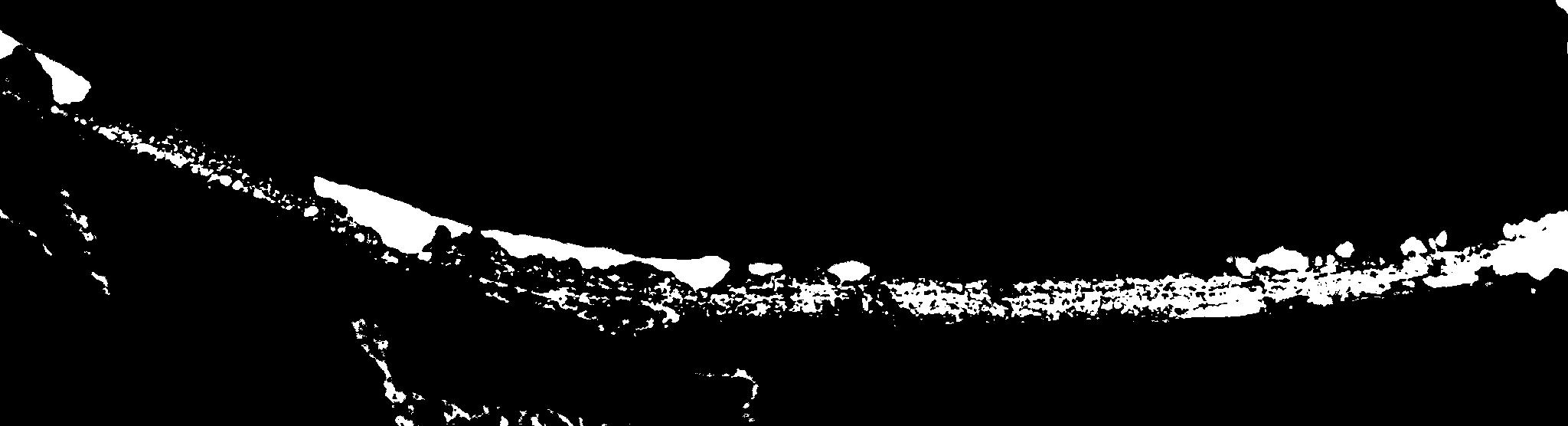

Supplement: S8 Data — (ZIP) [file pone.0297284.s008.zip › Level 4 processed Sample/processed_11/latex/WSO_latex.jpg]

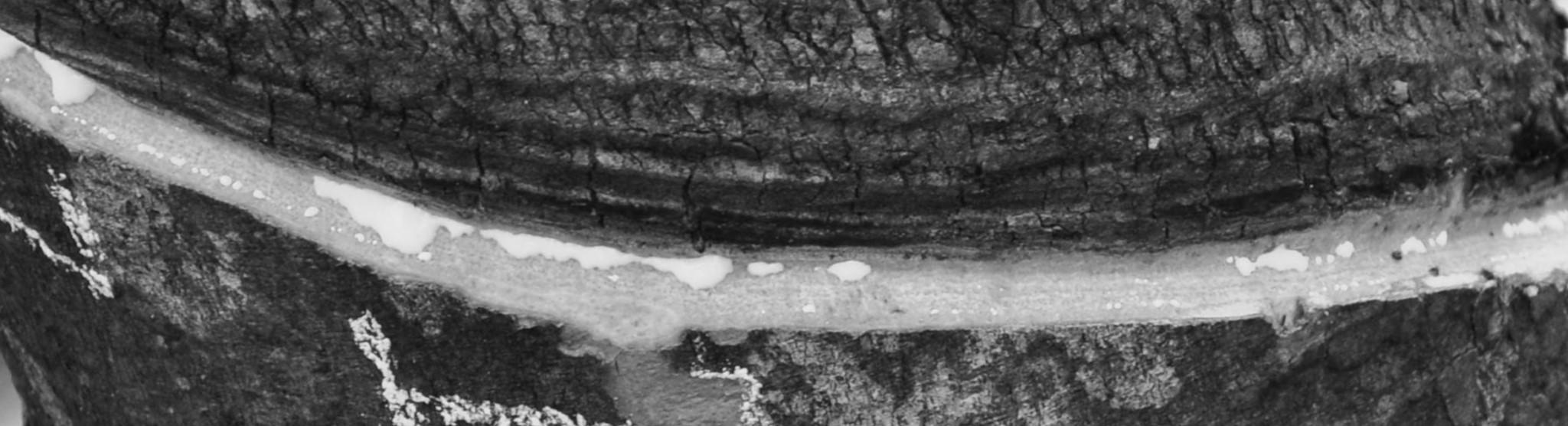

Supplement: S8 Data — (ZIP) [file pone.0297284.s008.zip › Level 4 processed Sample/processed_11/original_image.jpg]

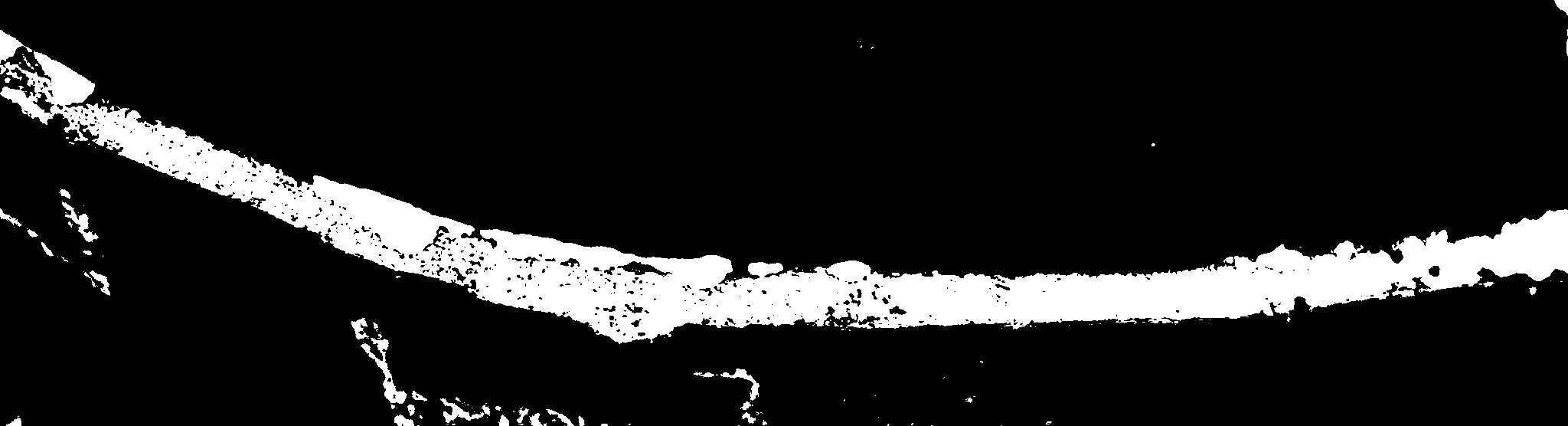

Supplement: S8 Data — (ZIP) [file pone.0297284.s008.zip › Level 4 processed Sample/processed_11/scar/AHA_scar.jpg]

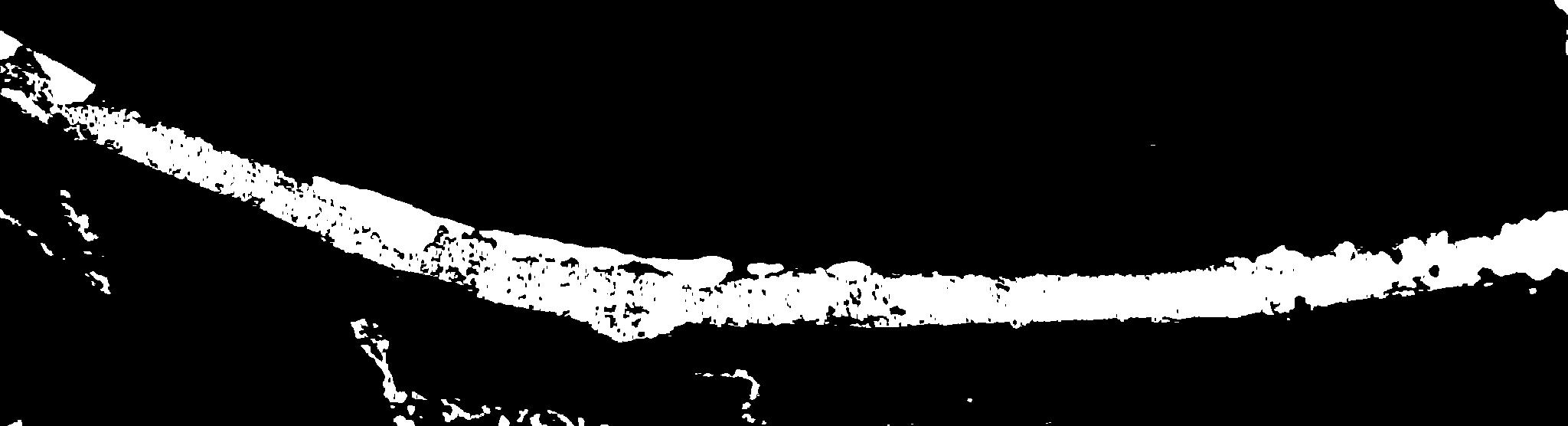

Supplement: S8 Data — (ZIP) [file pone.0297284.s008.zip › Level 4 processed Sample/processed_11/scar/DBO_scar.jpg]

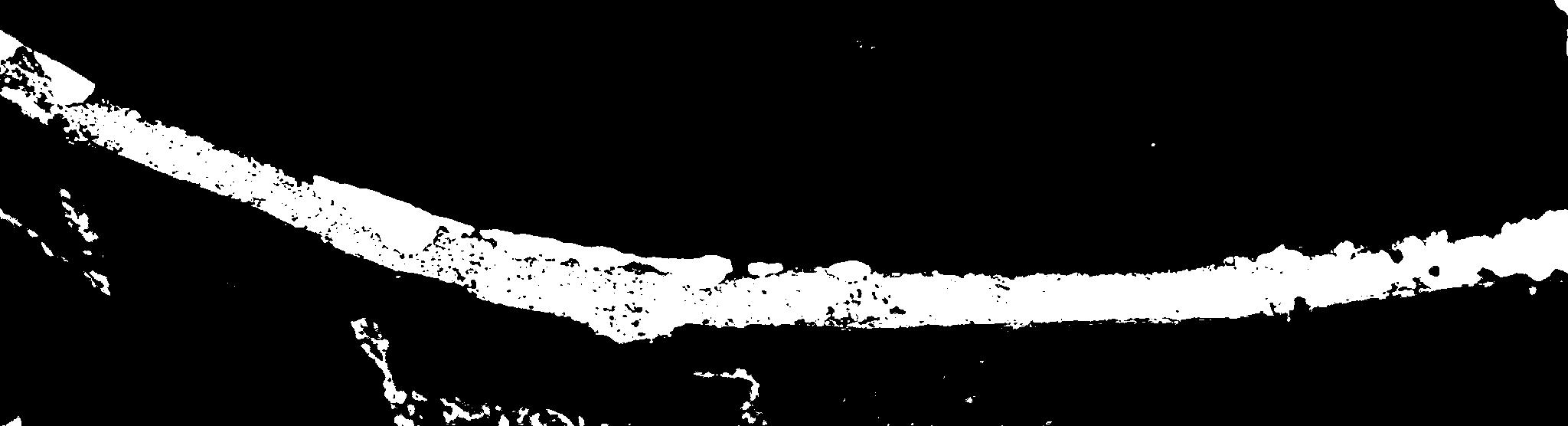

Supplement: S8 Data — (ZIP) [file pone.0297284.s008.zip › Level 4 processed Sample/processed_11/scar/WOA_scar.jpg]

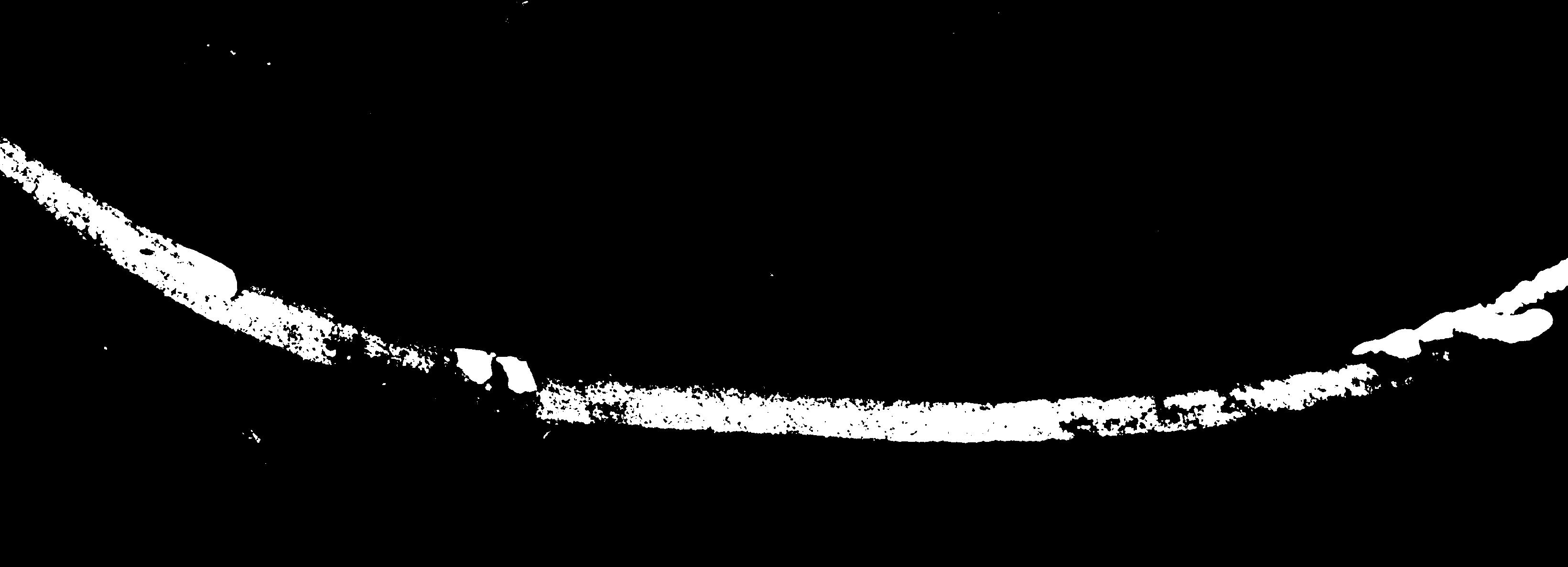

Supplement: S8 Data — (ZIP) [file pone.0297284.s008.zip › Level 4 processed Sample/processed_12/latex/AHA_latex.jpg]

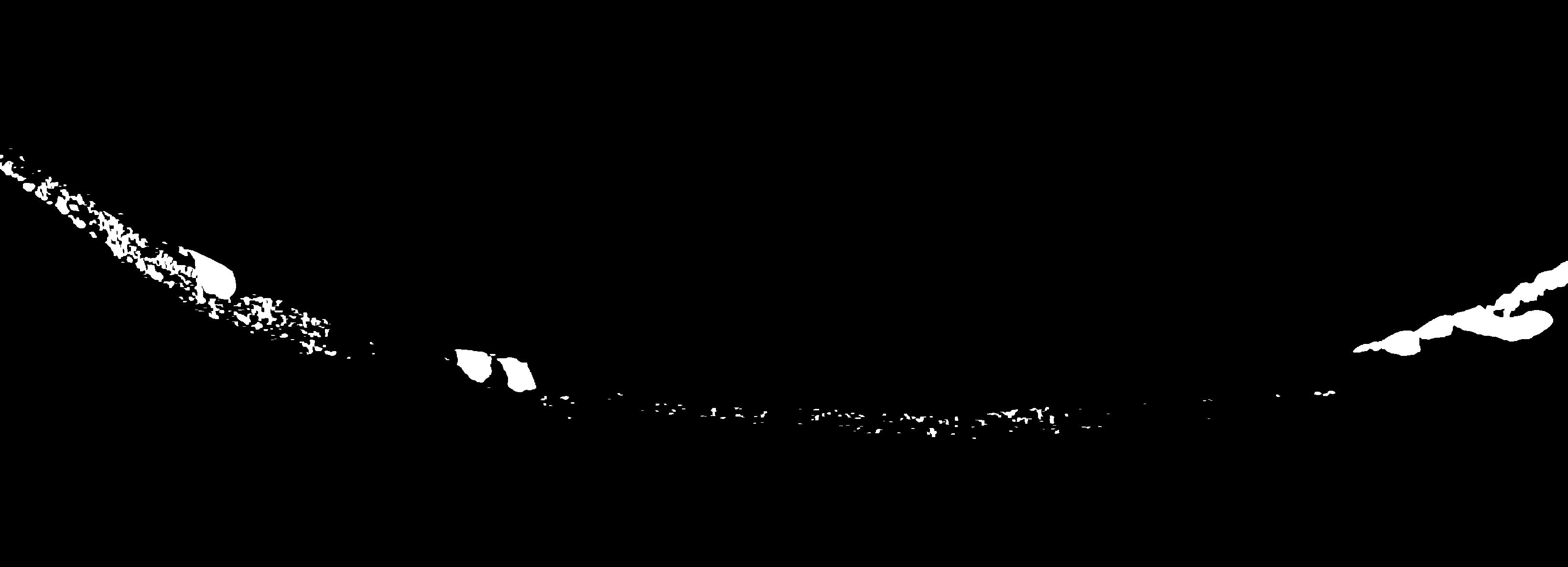

Supplement: S8 Data — (ZIP) [file pone.0297284.s008.zip › Level 4 processed Sample/processed_12/latex/DBO_latex.jpg]

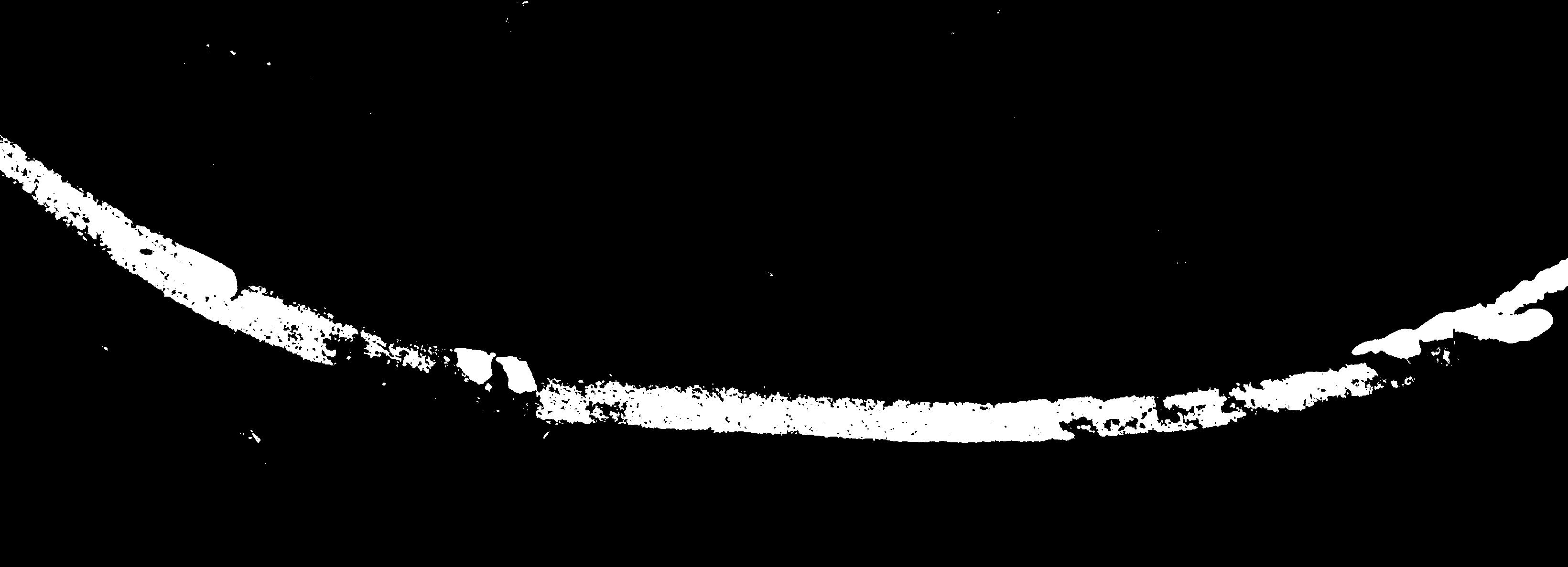

Supplement: S8 Data — (ZIP) [file pone.0297284.s008.zip › Level 4 processed Sample/processed_12/latex/GWO_latex.jpg]

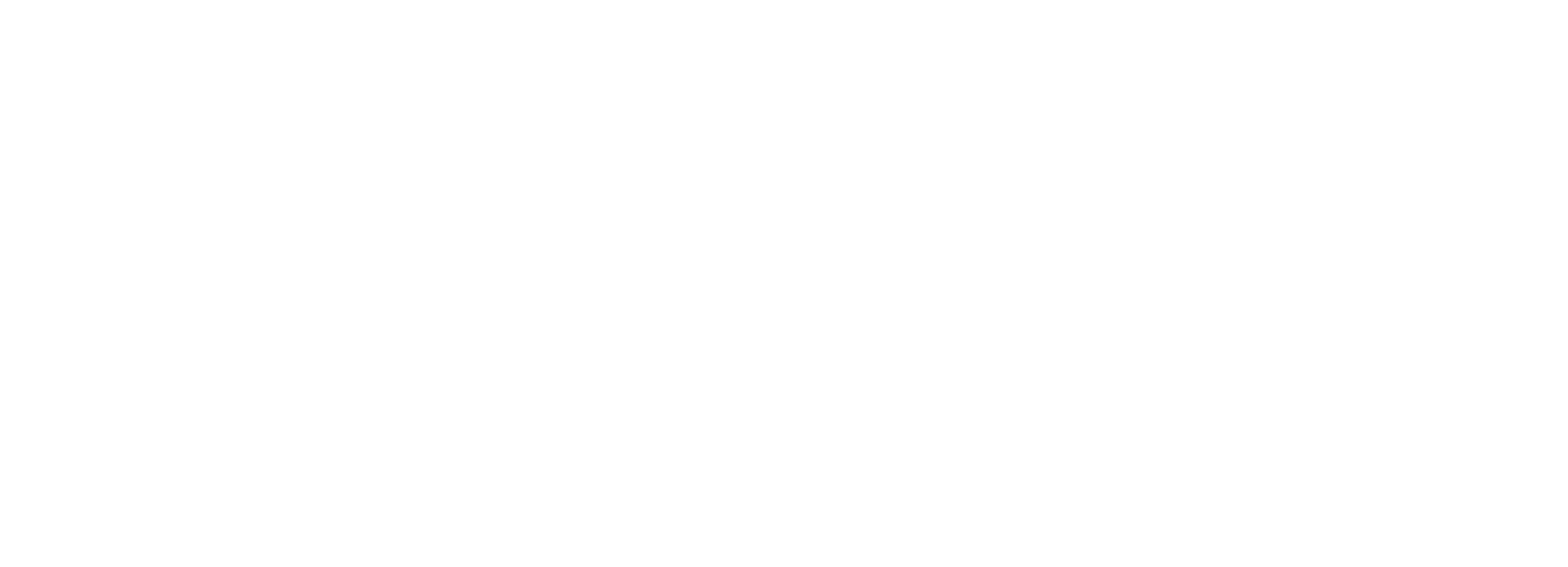

Supplement: S8 Data — (ZIP) [file pone.0297284.s008.zip › Level 4 processed Sample/processed_12/latex/OTSU_latex.jpg]

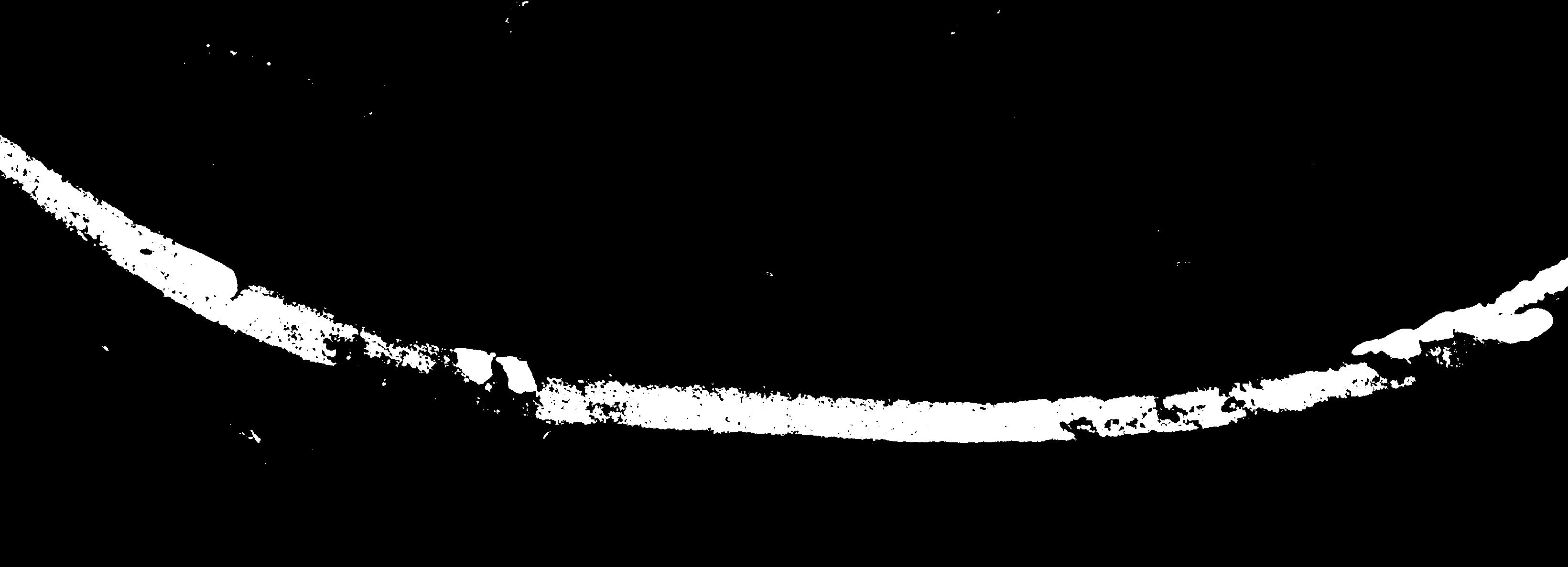

Supplement: S8 Data — (ZIP) [file pone.0297284.s008.zip › Level 4 processed Sample/processed_12/latex/WOA_latex.jpg]

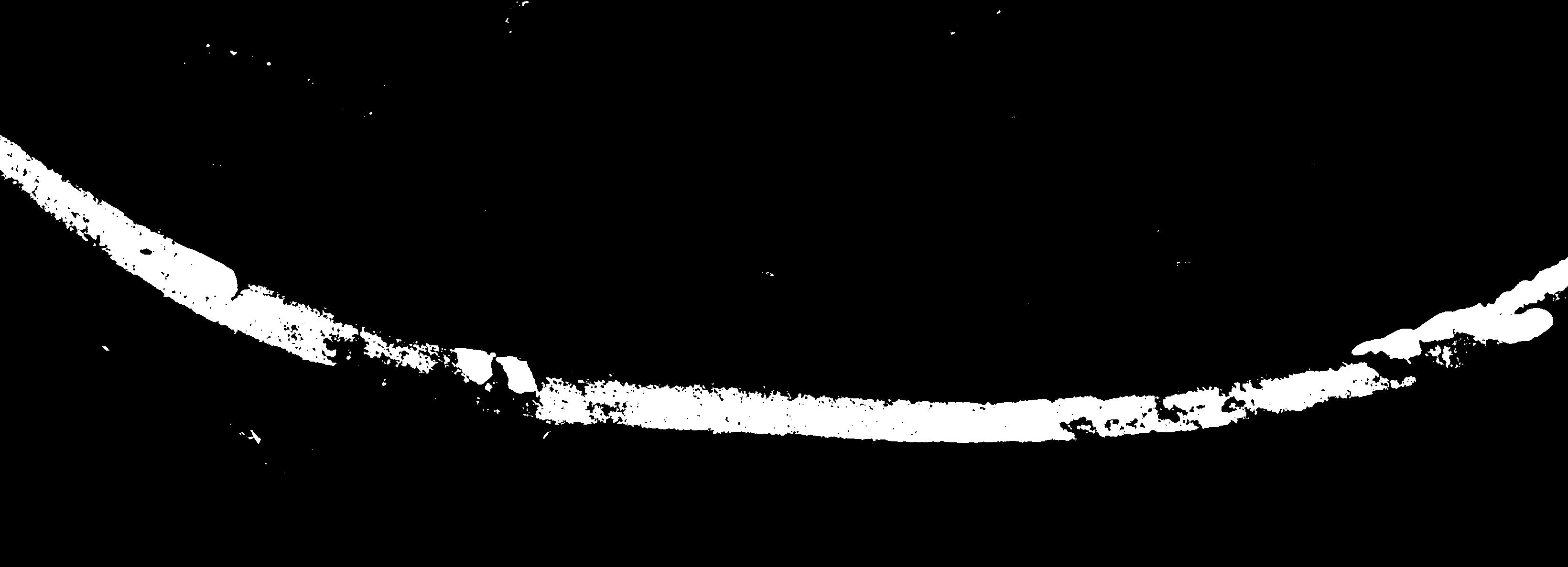

Supplement: S8 Data — (ZIP) [file pone.0297284.s008.zip › Level 4 processed Sample/processed_12/latex/WSO_latex.jpg]

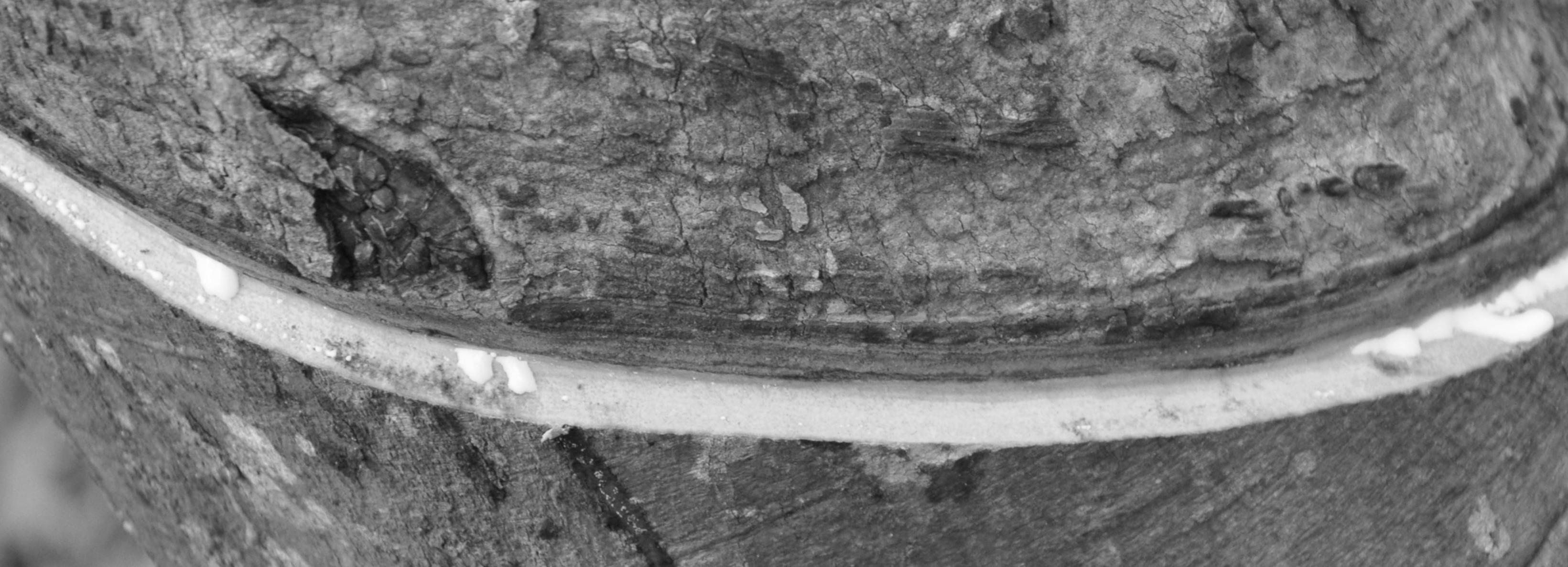

Supplement: S8 Data — (ZIP) [file pone.0297284.s008.zip › Level 4 processed Sample/processed_12/original_image.jpg]

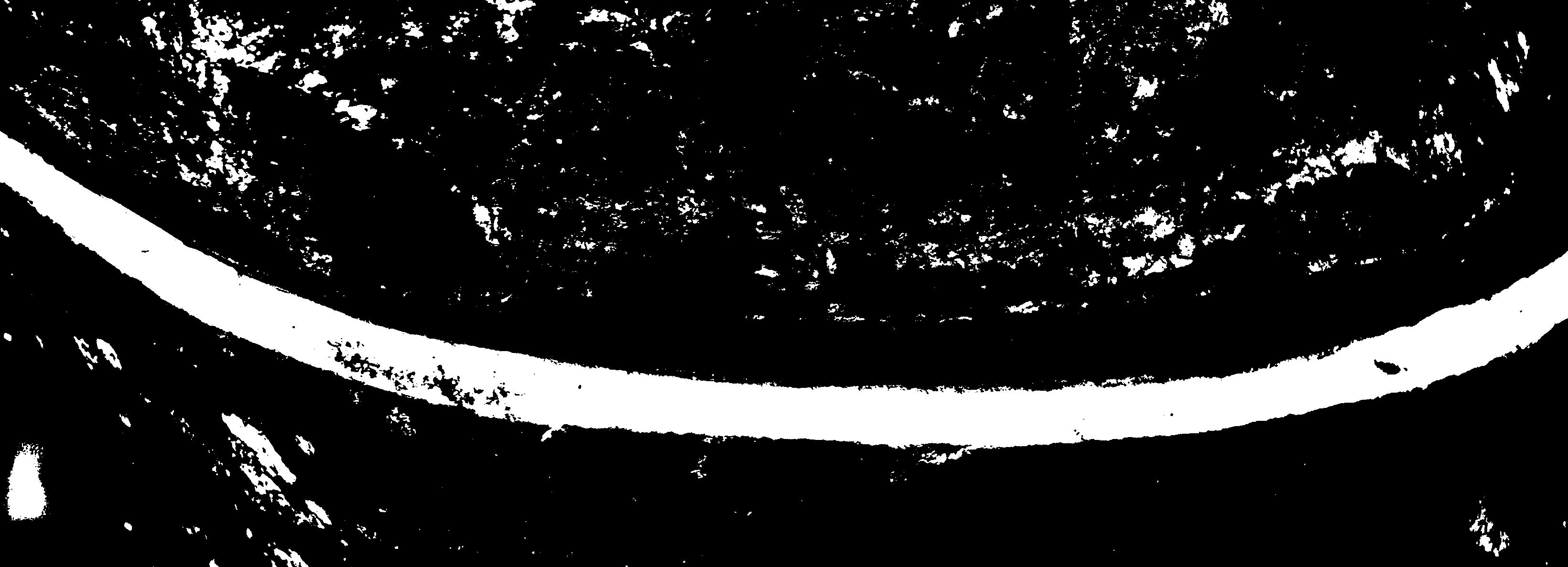

Supplement: S8 Data — (ZIP) [file pone.0297284.s008.zip › Level 4 processed Sample/processed_12/scar/AHA_scar.jpg]

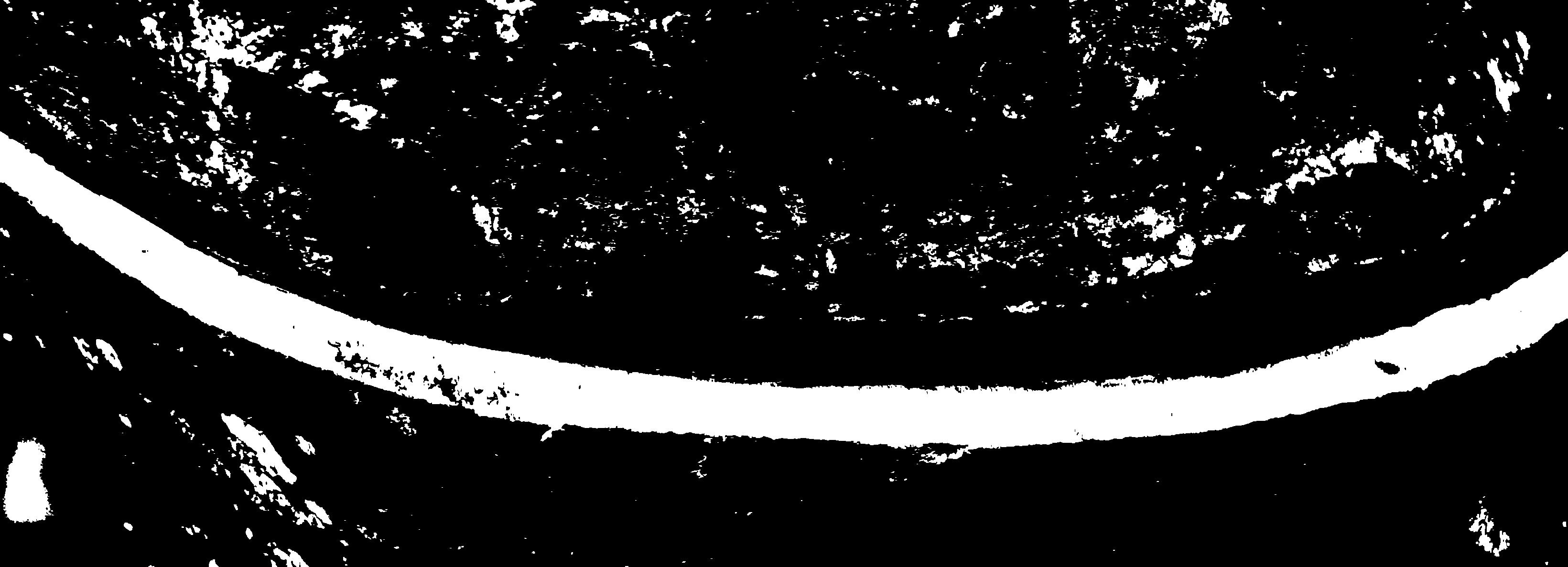

Supplement: S8 Data — (ZIP) [file pone.0297284.s008.zip › Level 4 processed Sample/processed_12/scar/DBO_scar.jpg]

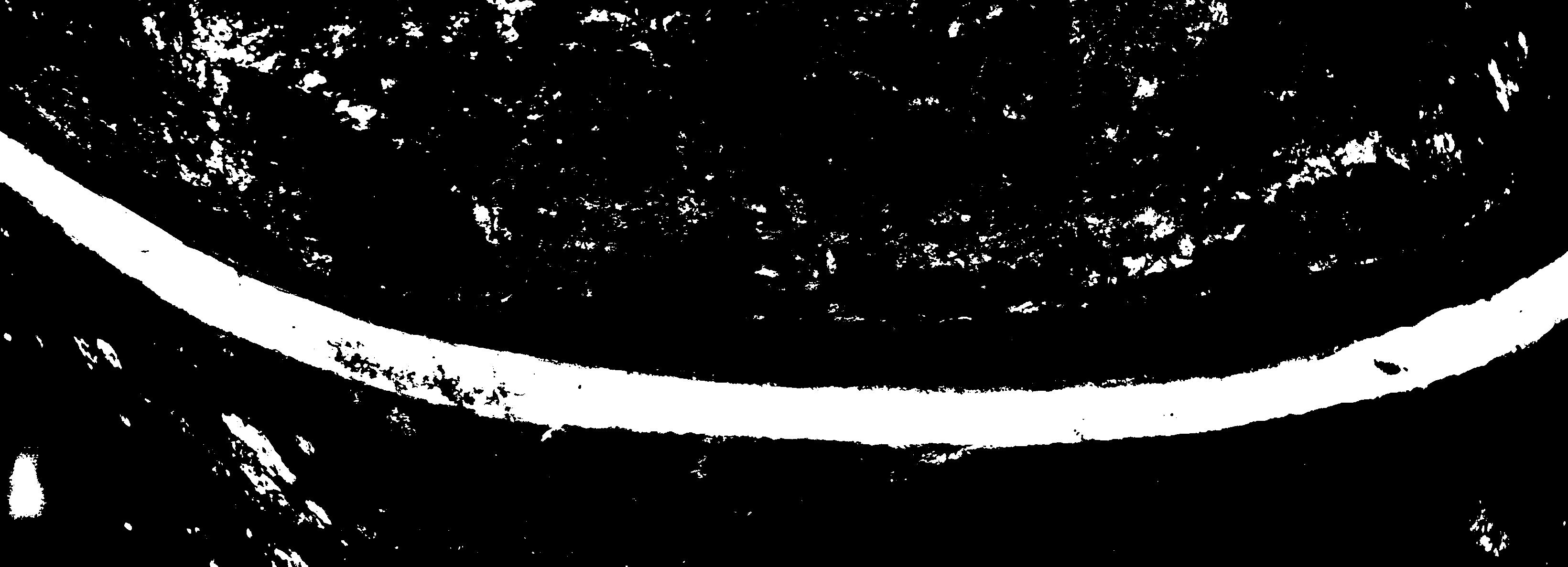

Supplement: S8 Data — (ZIP) [file pone.0297284.s008.zip › Level 4 processed Sample/processed_12/scar/GWO_scar.jpg]

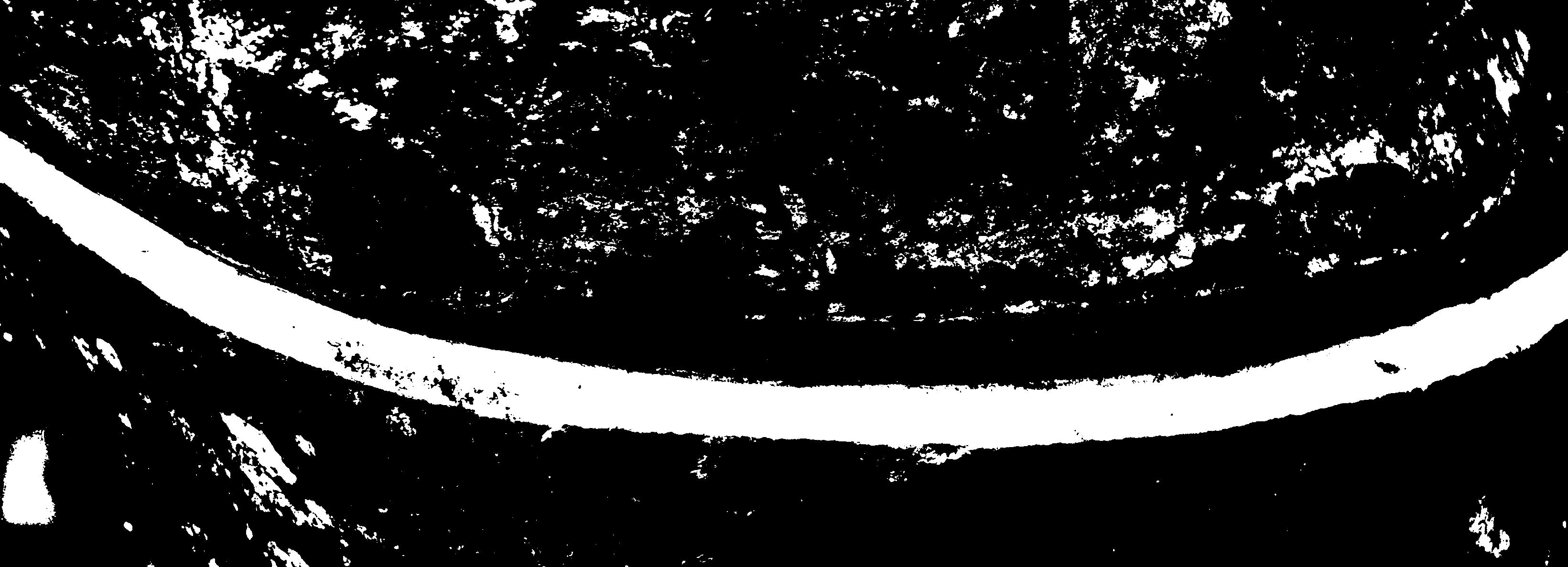

Supplement: S8 Data — (ZIP) [file pone.0297284.s008.zip › Level 4 processed Sample/processed_12/scar/WOA_scar.jpg]

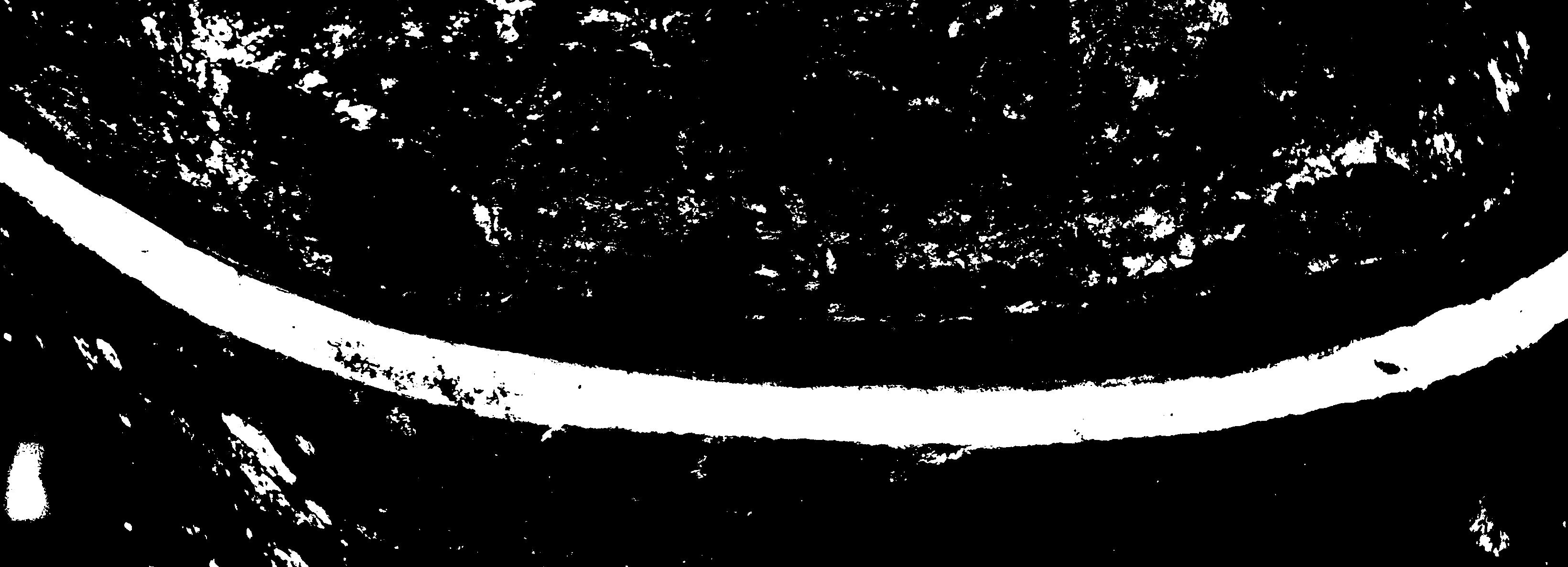

Supplement: S8 Data — (ZIP) [file pone.0297284.s008.zip › Level 4 processed Sample/processed_12/scar/WSO_scar.jpg]

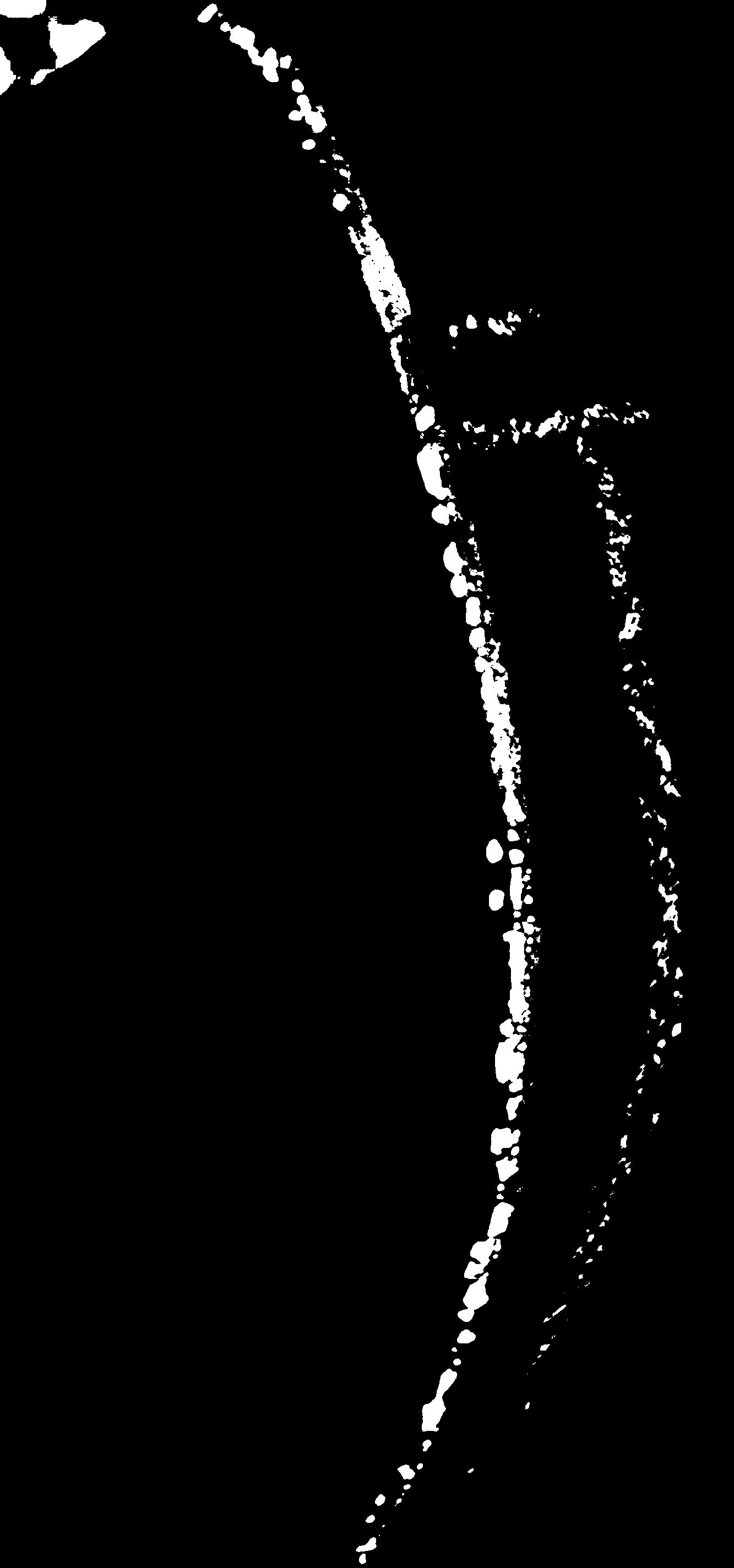

Supplement: S8 Data — (ZIP) [file pone.0297284.s008.zip › Level 4 processed Sample/processed_13/latex/AHA_latex.jpg]

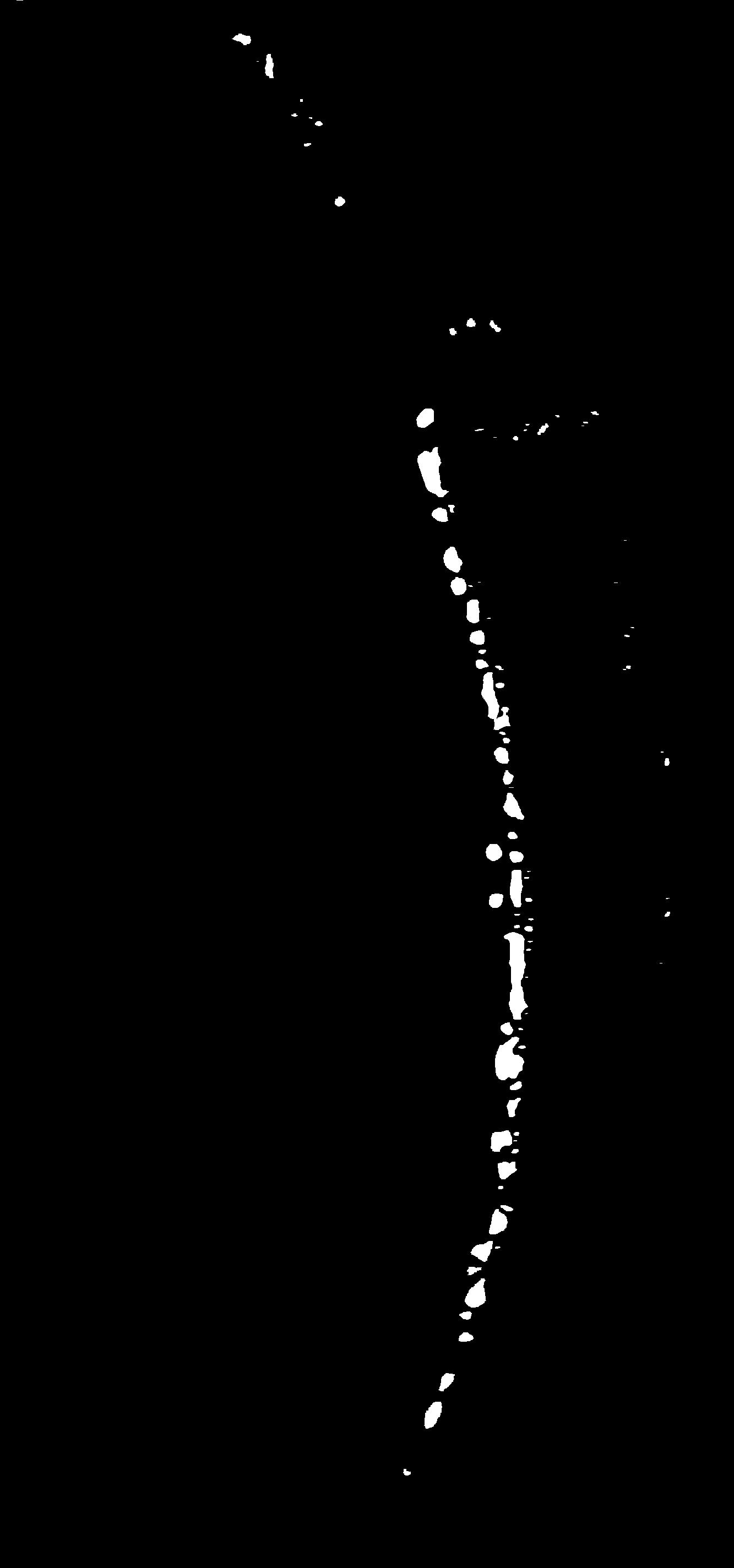

Supplement: S8 Data — (ZIP) [file pone.0297284.s008.zip › Level 4 processed Sample/processed_13/latex/DBO_latex.jpg]

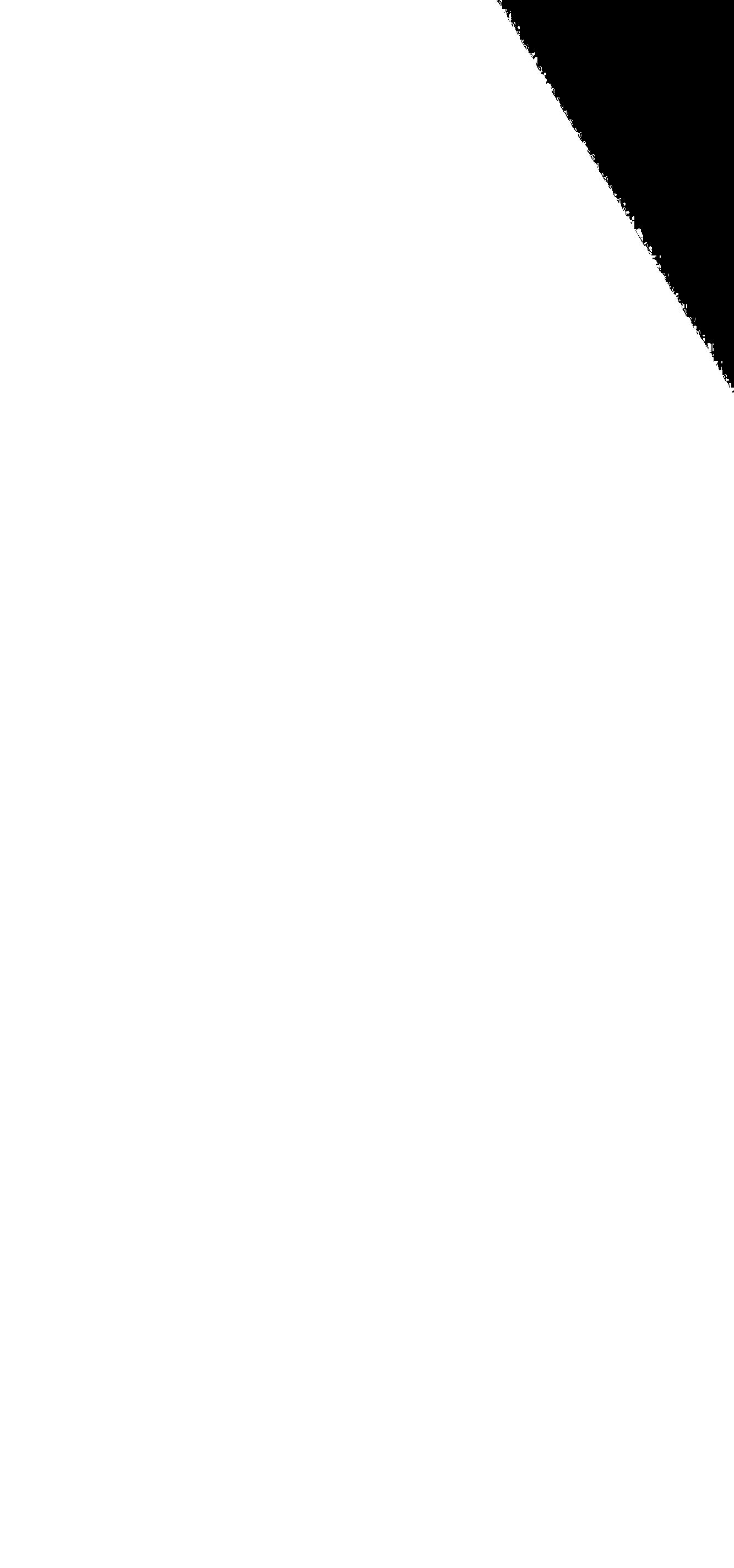

Supplement: S8 Data — (ZIP) [file pone.0297284.s008.zip › Level 4 processed Sample/processed_13/latex/OTSU_latex.jpg]

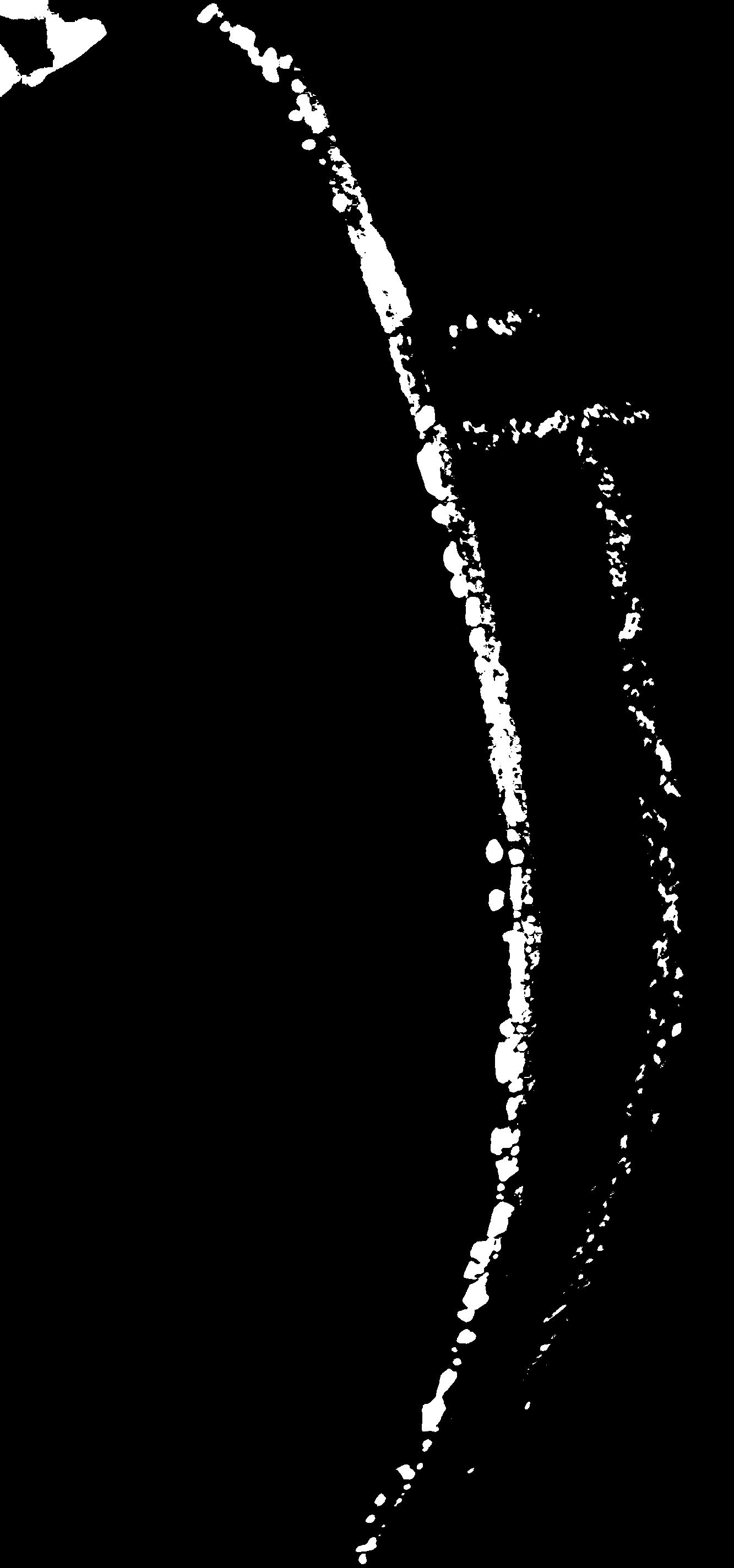

Supplement: S8 Data — (ZIP) [file pone.0297284.s008.zip › Level 4 processed Sample/processed_13/latex/WSO_latex.jpg]

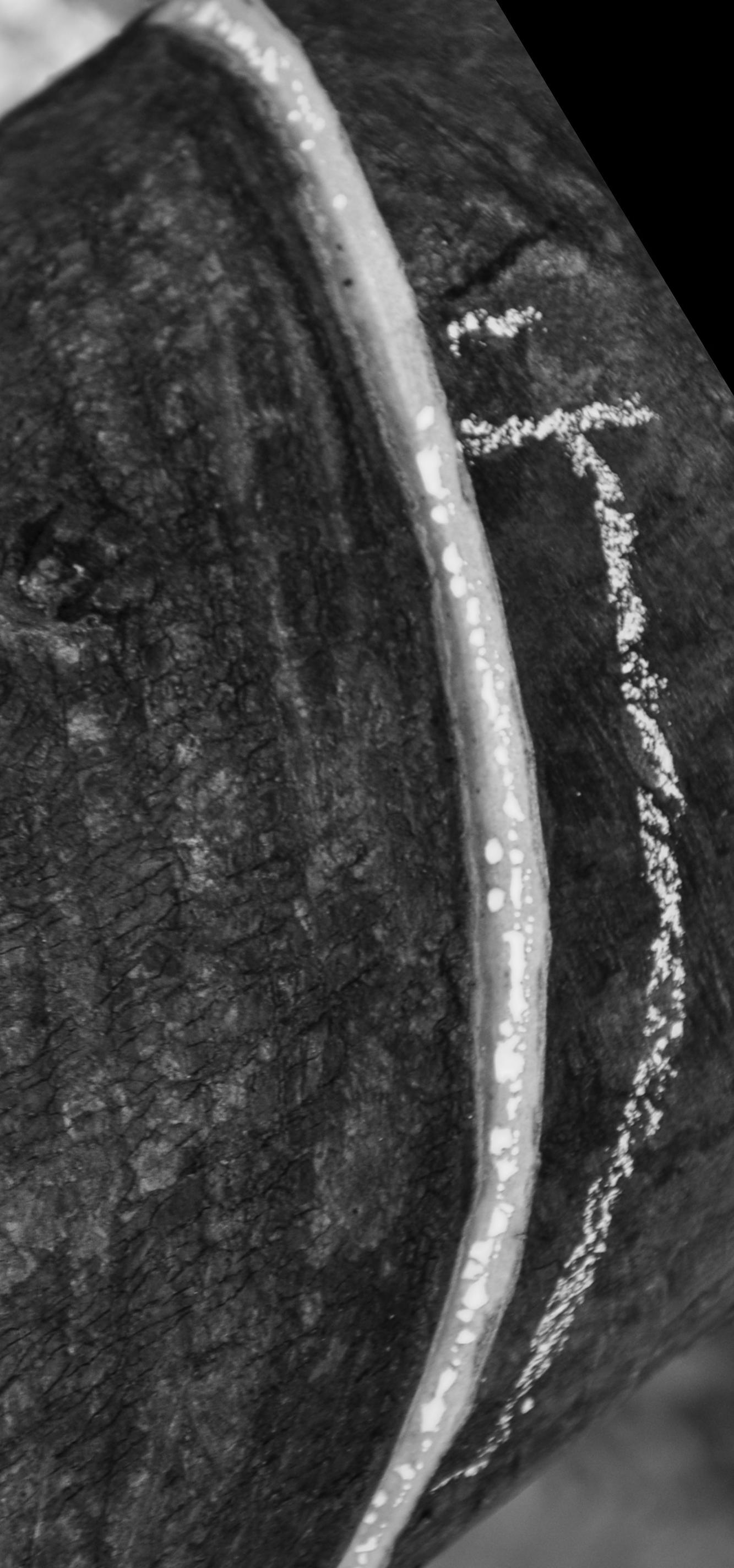

Supplement: S8 Data — (ZIP) [file pone.0297284.s008.zip › Level 4 processed Sample/processed_13/original_image.jpg]

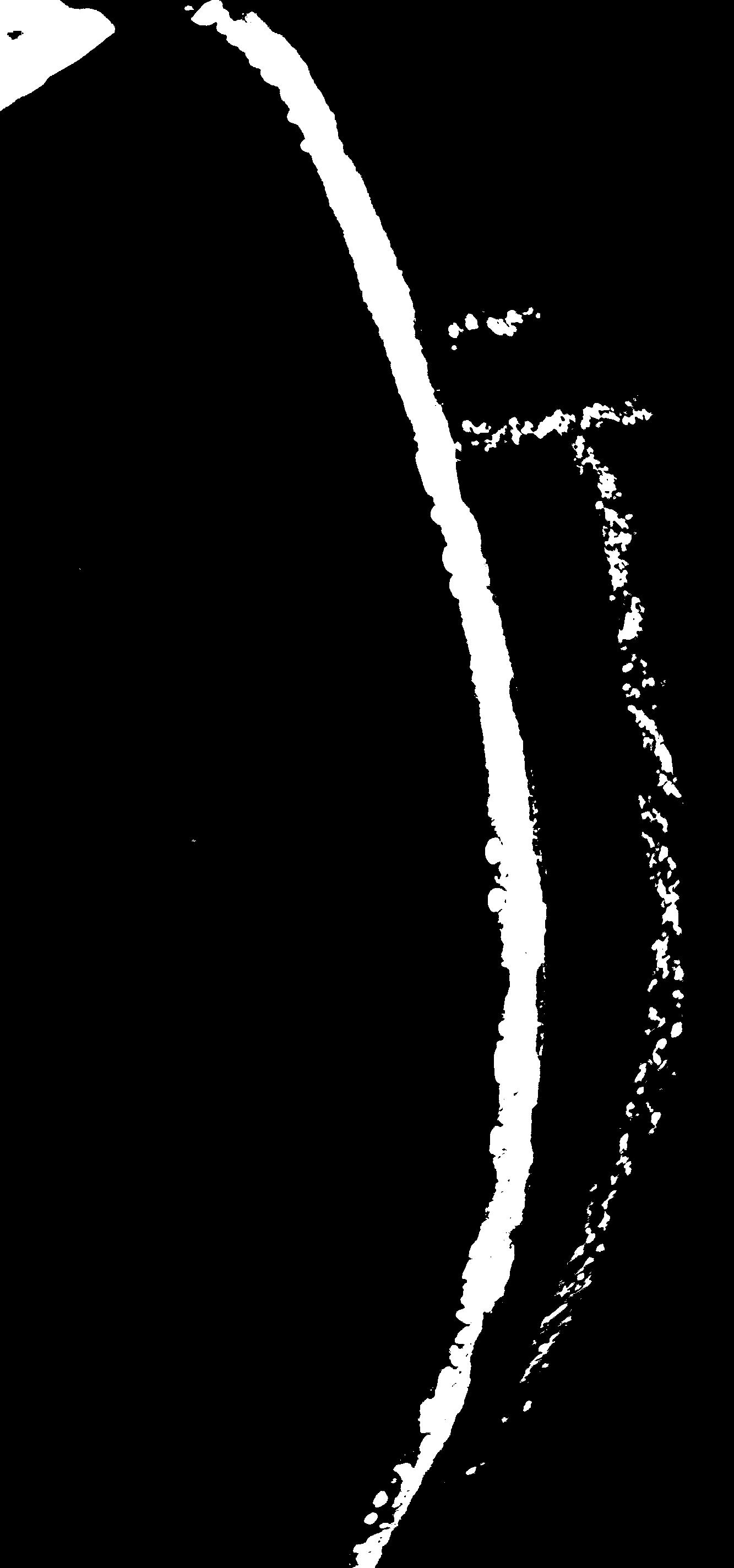

Supplement: S8 Data — (ZIP) [file pone.0297284.s008.zip › Level 4 processed Sample/processed_13/scar/AHA_scar.jpg]

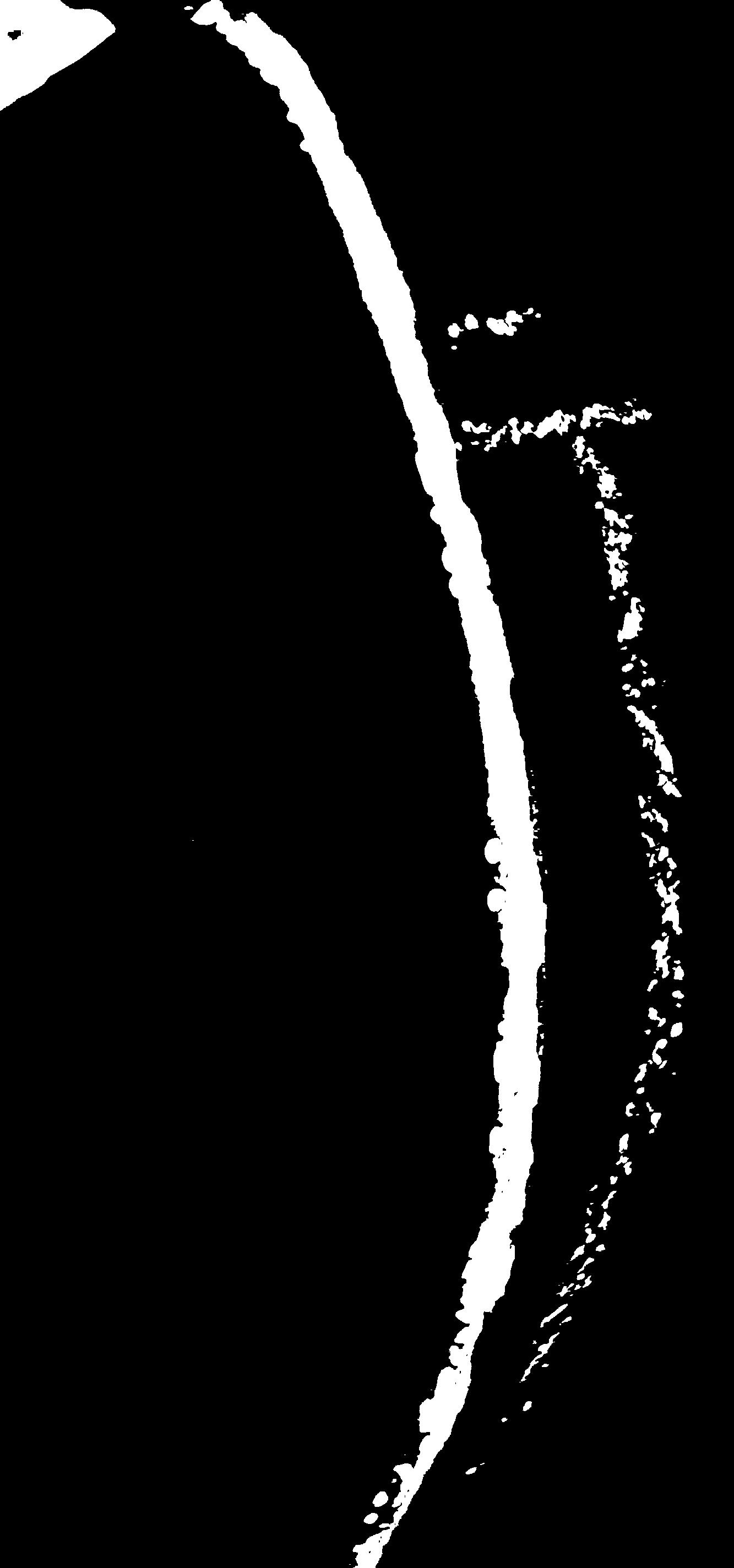

Supplement: S8 Data — (ZIP) [file pone.0297284.s008.zip › Level 4 processed Sample/processed_13/scar/DBO_scar.jpg]

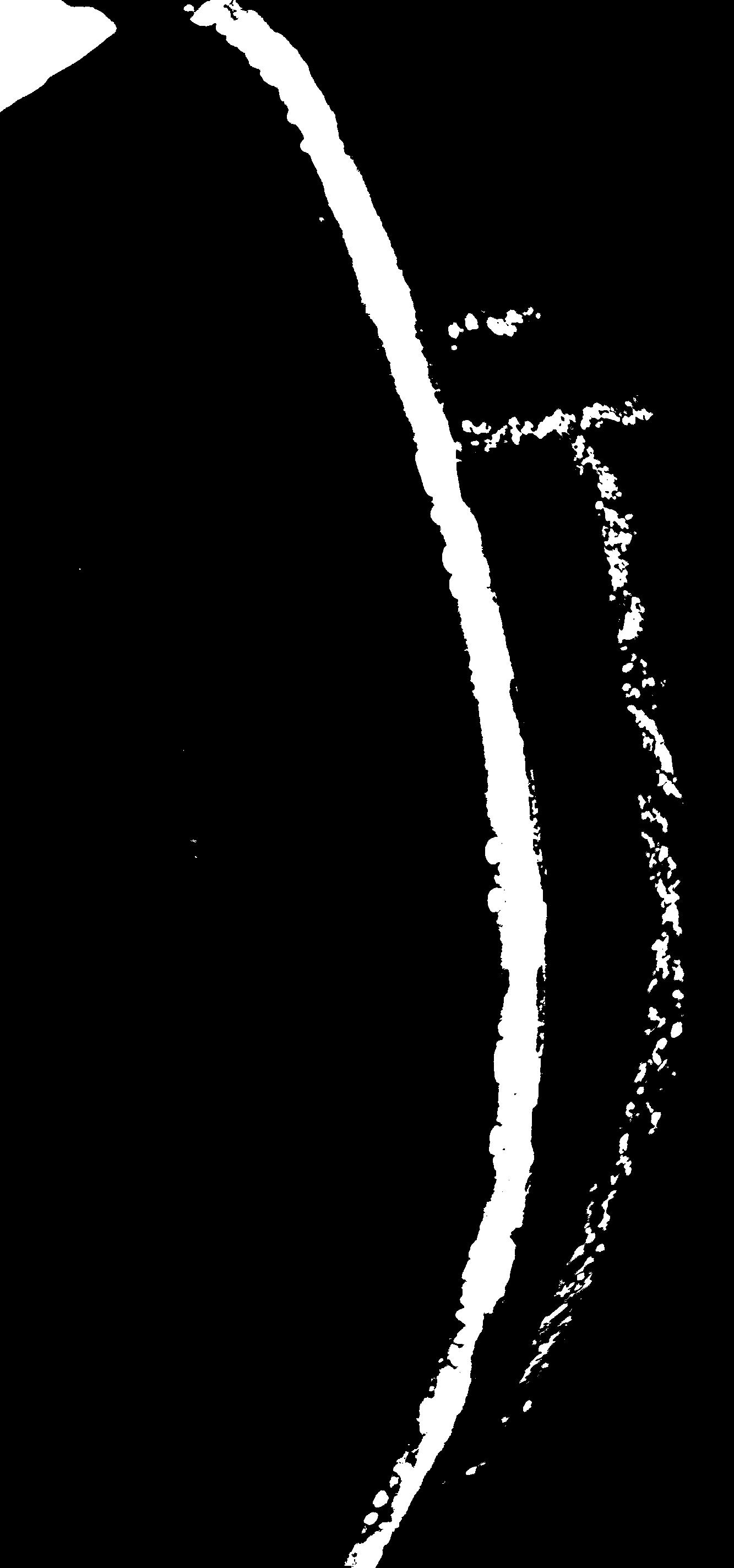

Supplement: S8 Data — (ZIP) [file pone.0297284.s008.zip › Level 4 processed Sample/processed_13/scar/WSO_scar.jpg]

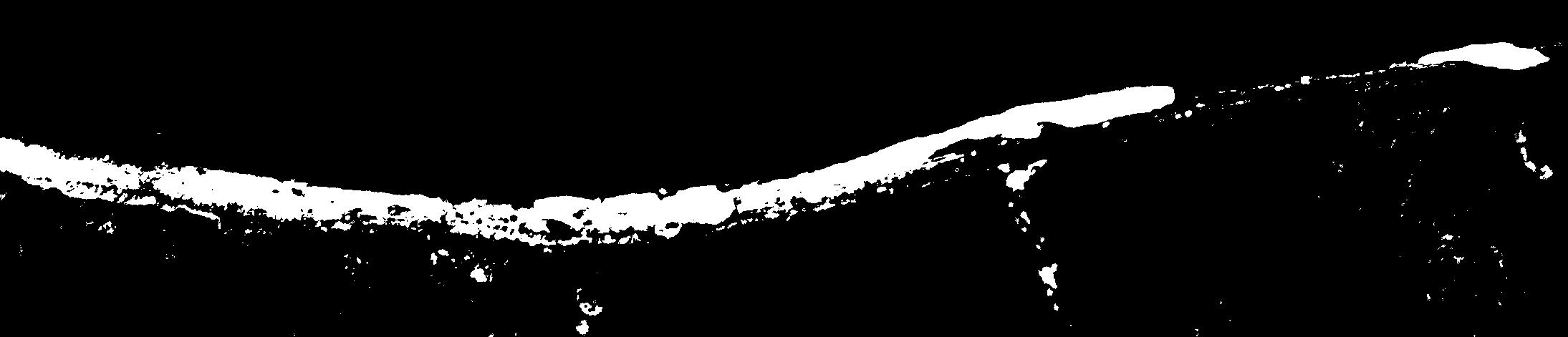

Supplement: S8 Data — (ZIP) [file pone.0297284.s008.zip › Level 4 processed Sample/processed_16/latex/AHA_latex.jpg]

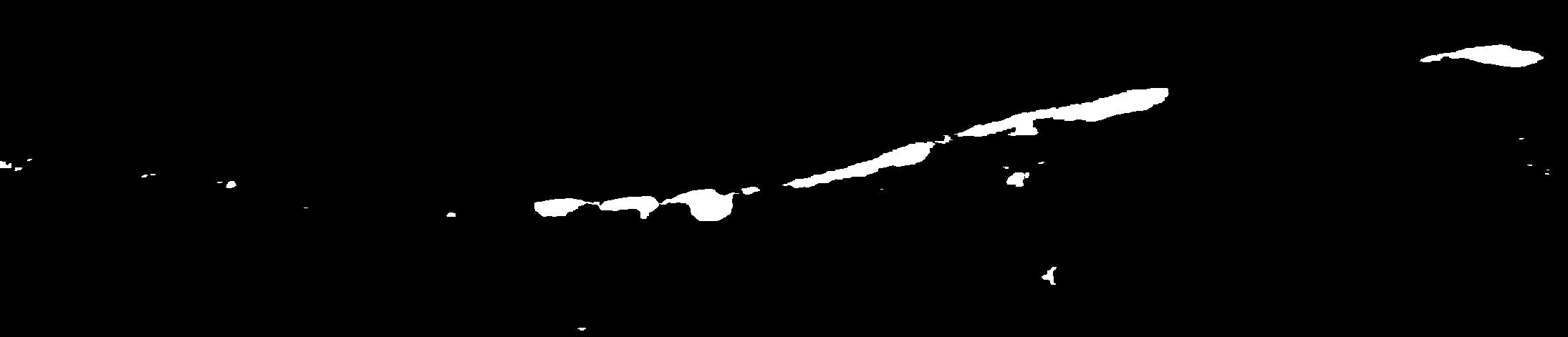

Supplement: S8 Data — (ZIP) [file pone.0297284.s008.zip › Level 4 processed Sample/processed_16/latex/DBO_latex.jpg]

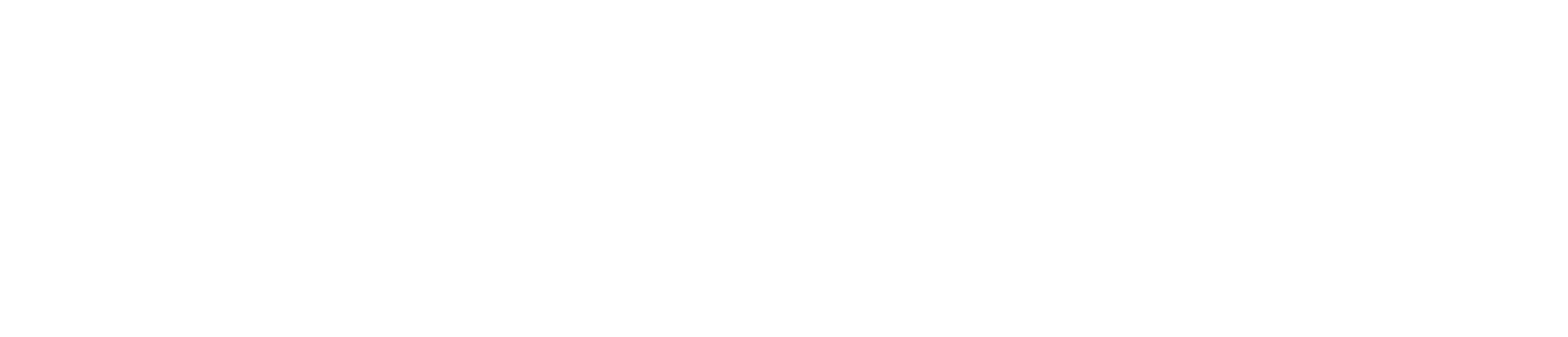

Supplement: S8 Data — (ZIP) [file pone.0297284.s008.zip › Level 4 processed Sample/processed_16/latex/OTSU_latex.jpg]

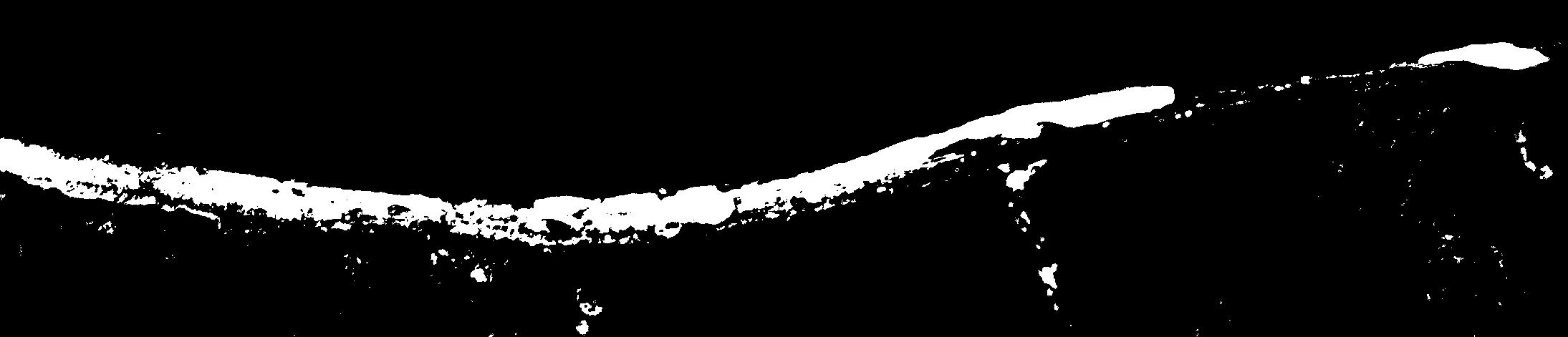

Supplement: S8 Data — (ZIP) [file pone.0297284.s008.zip › Level 4 processed Sample/processed_16/latex/WOA_latex.jpg]

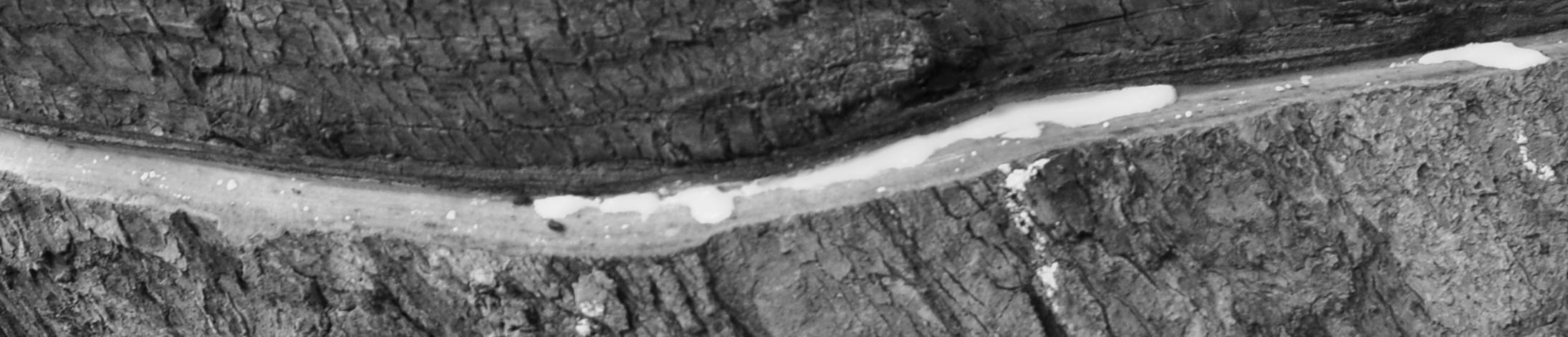

Supplement: S8 Data — (ZIP) [file pone.0297284.s008.zip › Level 4 processed Sample/processed_16/original_image.jpg]

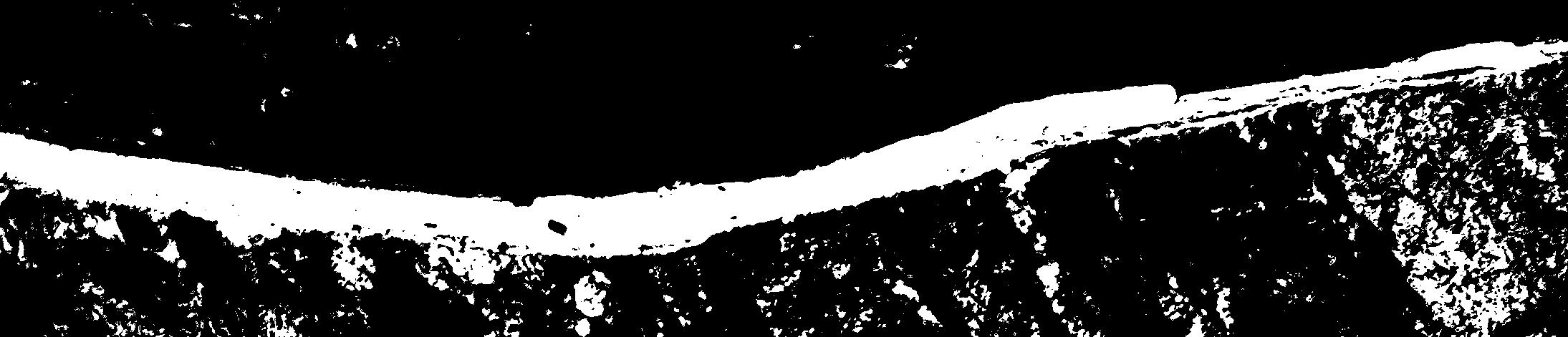

Supplement: S8 Data — (ZIP) [file pone.0297284.s008.zip › Level 4 processed Sample/processed_16/scar/AHA_scar.jpg]

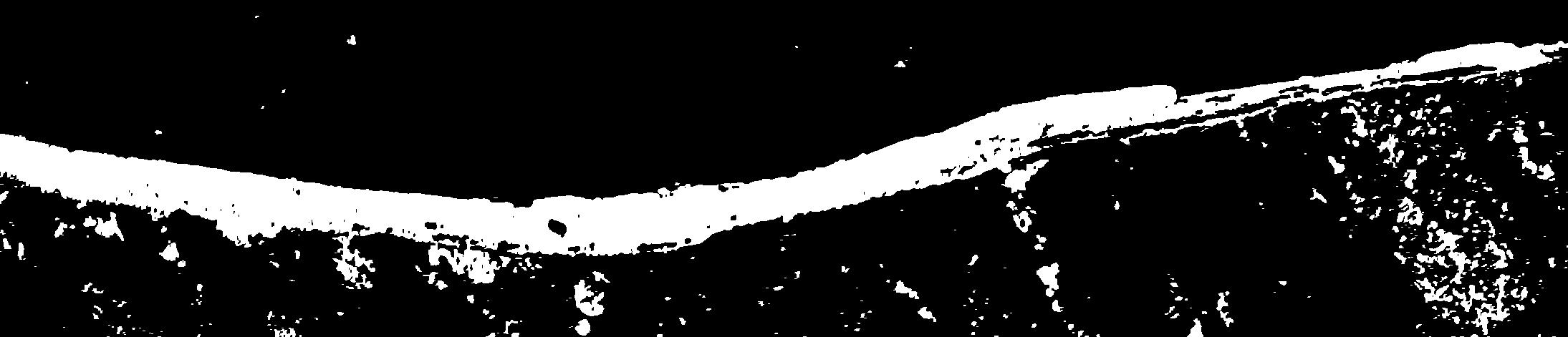

Supplement: S8 Data — (ZIP) [file pone.0297284.s008.zip › Level 4 processed Sample/processed_16/scar/DBO_scar.jpg]

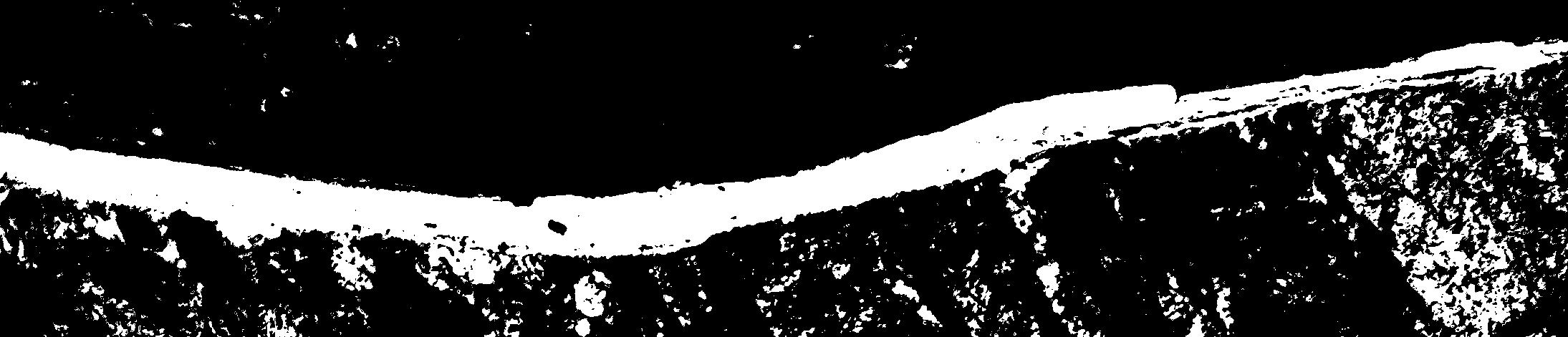

Supplement: S8 Data — (ZIP) [file pone.0297284.s008.zip › Level 4 processed Sample/processed_16/scar/WSO_scar.jpg]

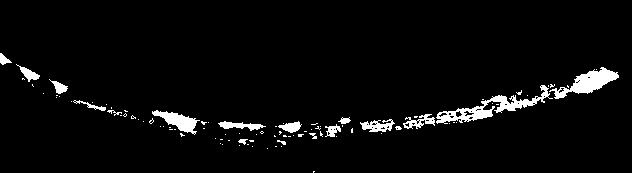

Supplement: S8 Data — (ZIP) [file pone.0297284.s008.zip › Level 4 processed Sample/processed_18/latex/AHA_latex.jpg]

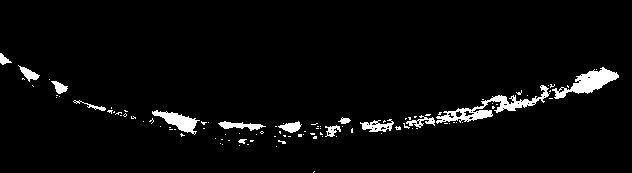

Supplement: S8 Data — (ZIP) [file pone.0297284.s008.zip › Level 4 processed Sample/processed_18/latex/CSA_latex.jpg]

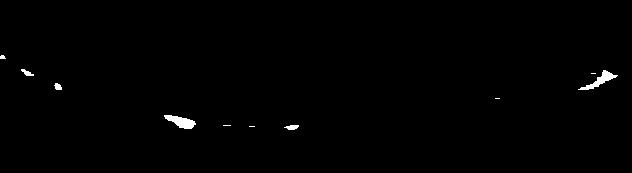

Supplement: S8 Data — (ZIP) [file pone.0297284.s008.zip › Level 4 processed Sample/processed_18/latex/DBO_latex.jpg]

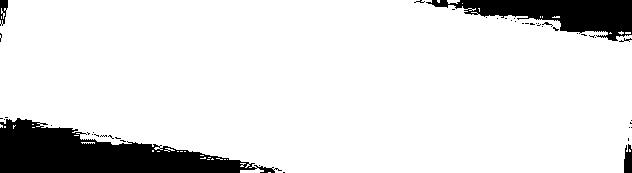

Supplement: S8 Data — (ZIP) [file pone.0297284.s008.zip › Level 4 processed Sample/processed_18/latex/OTSU_latex.jpg]

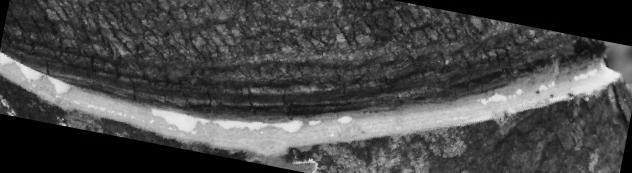

Supplement: S8 Data — (ZIP) [file pone.0297284.s008.zip › Level 4 processed Sample/processed_18/original_image.jpg]

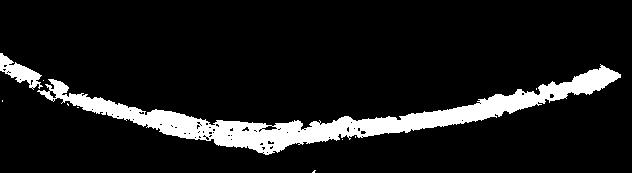

Supplement: S8 Data — (ZIP) [file pone.0297284.s008.zip › Level 4 processed Sample/processed_18/scar/AHA_scar.jpg]

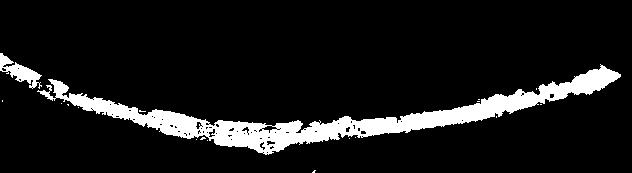

Supplement: S8 Data — (ZIP) [file pone.0297284.s008.zip › Level 4 processed Sample/processed_18/scar/CSA_scar.jpg]

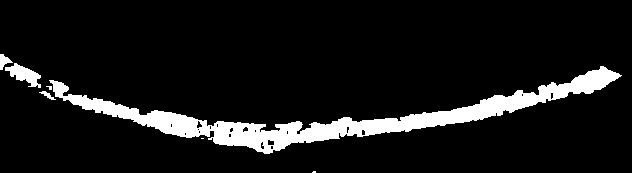

Supplement: S8 Data — (ZIP) [file pone.0297284.s008.zip › Level 4 processed Sample/processed_18/scar/DBO_scar.jpg]

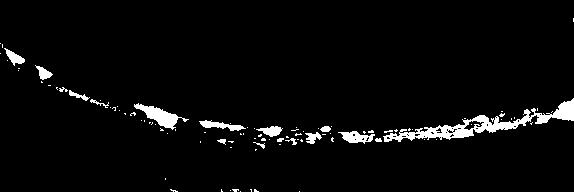

Supplement: S8 Data — (ZIP) [file pone.0297284.s008.zip › Level 4 processed Sample/processed_19/latex/AHA_latex.jpg]

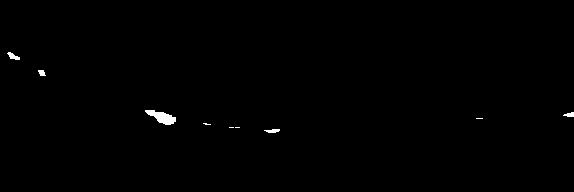

Supplement: S8 Data — (ZIP) [file pone.0297284.s008.zip › Level 4 processed Sample/processed_19/latex/DBO_latex.jpg]

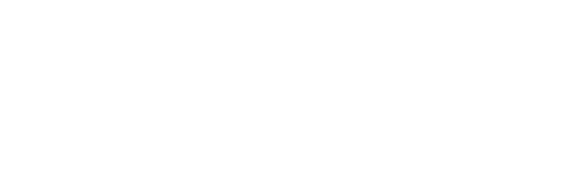

Supplement: S8 Data — (ZIP) [file pone.0297284.s008.zip › Level 4 processed Sample/processed_19/latex/OTSU_latex.jpg]

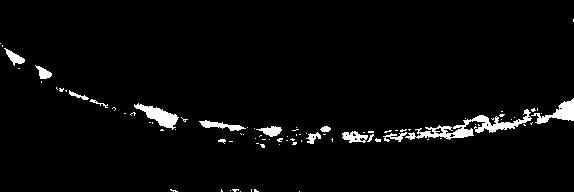

Supplement: S8 Data — (ZIP) [file pone.0297284.s008.zip › Level 4 processed Sample/processed_19/latex/WSO_latex.jpg]

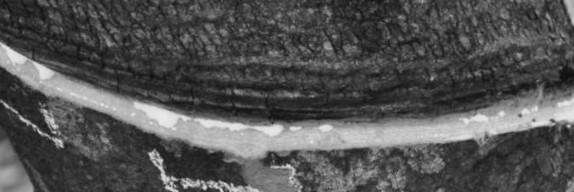

Supplement: S8 Data — (ZIP) [file pone.0297284.s008.zip › Level 4 processed Sample/processed_19/original_image.jpg]

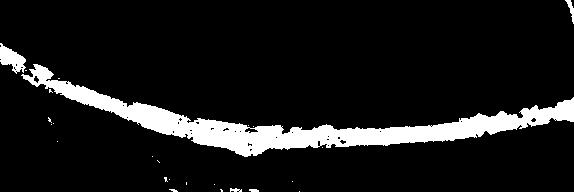

Supplement: S8 Data — (ZIP) [file pone.0297284.s008.zip › Level 4 processed Sample/processed_19/scar/AHA_scar.jpg]

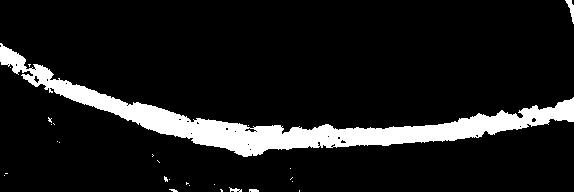

Supplement: S8 Data — (ZIP) [file pone.0297284.s008.zip › Level 4 processed Sample/processed_19/scar/CSA_scar.jpg]
