# Supplementary figures and images for: Advancing image segmentation with DBO-Otsu: Addressing rubber tree diseases through enhanced threshold techniques (part 6 of 7)
Source: PLoS One. 2024 Mar 21;19(3):e0297284. doi: 10.1371/journal.pone.0297284 (PMC10956860; doi:10.1371/journal.pone.0297284)

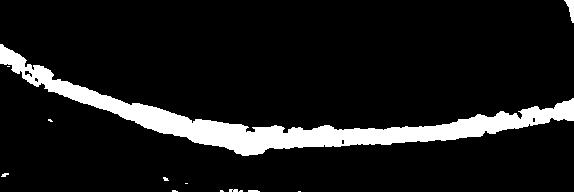

Supplement: S8 Data — (ZIP) [file pone.0297284.s008.zip › Level 4 processed Sample/processed_19/scar/DBO_scar.jpg]

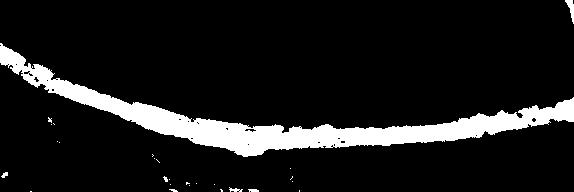

Supplement: S8 Data — (ZIP) [file pone.0297284.s008.zip › Level 4 processed Sample/processed_19/scar/WSO_scar.jpg]

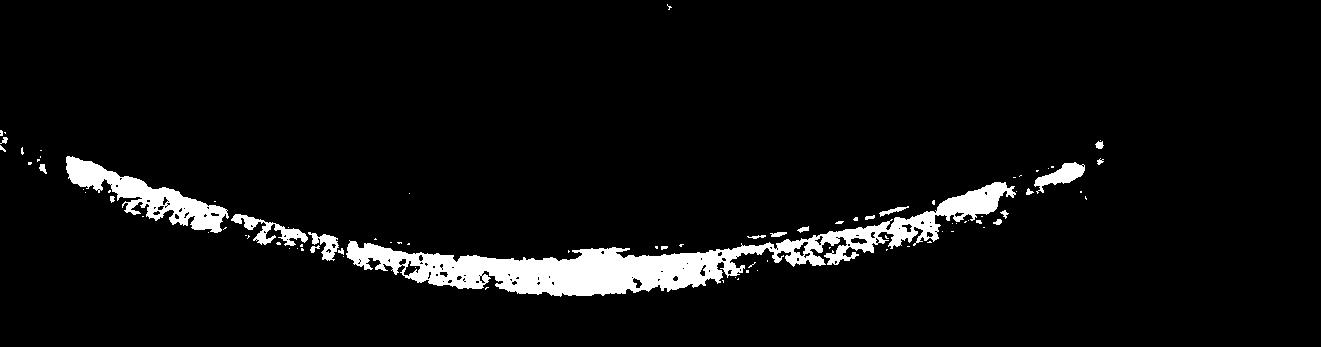

Supplement: S8 Data — (ZIP) [file pone.0297284.s008.zip › Level 4 processed Sample/processed_2/latex/AHA_latex.jpg]

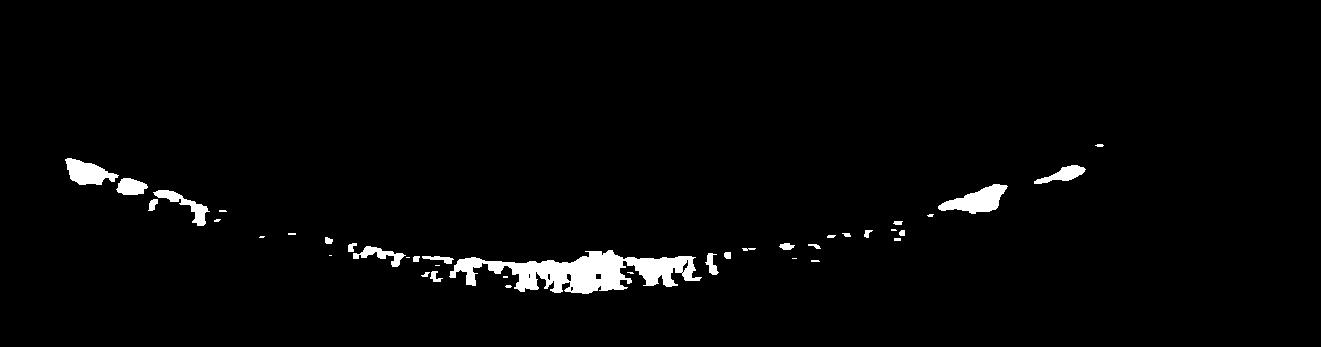

Supplement: S8 Data — (ZIP) [file pone.0297284.s008.zip › Level 4 processed Sample/processed_2/latex/DBO_latex.jpg]

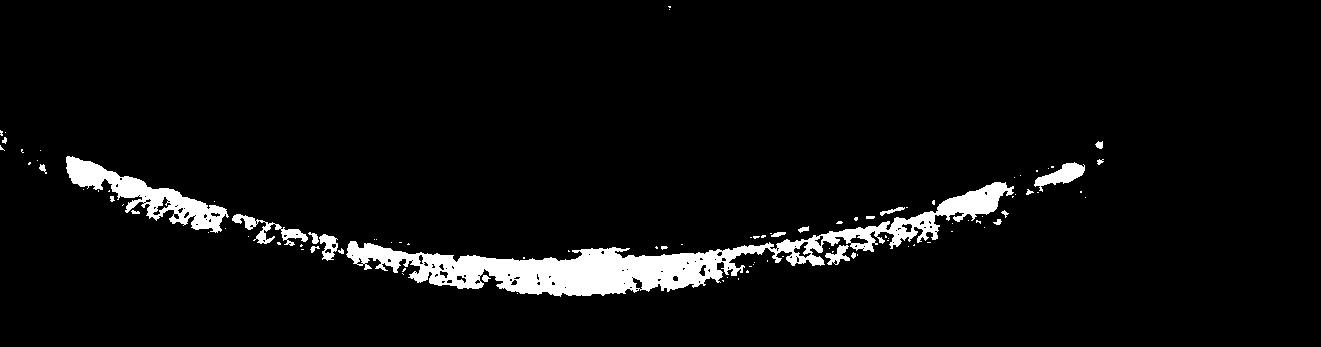

Supplement: S8 Data — (ZIP) [file pone.0297284.s008.zip › Level 4 processed Sample/processed_2/latex/GWO_latex.jpg]

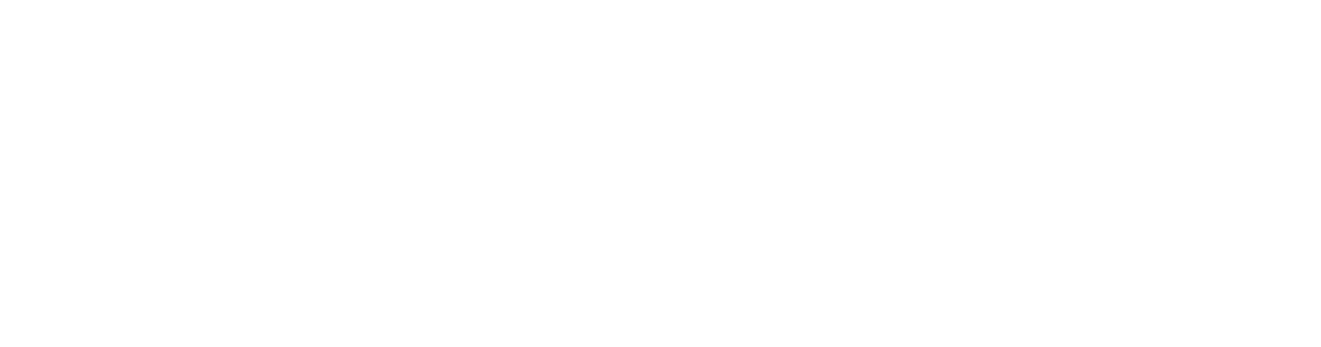

Supplement: S8 Data — (ZIP) [file pone.0297284.s008.zip › Level 4 processed Sample/processed_2/latex/OTSU_latex.jpg]

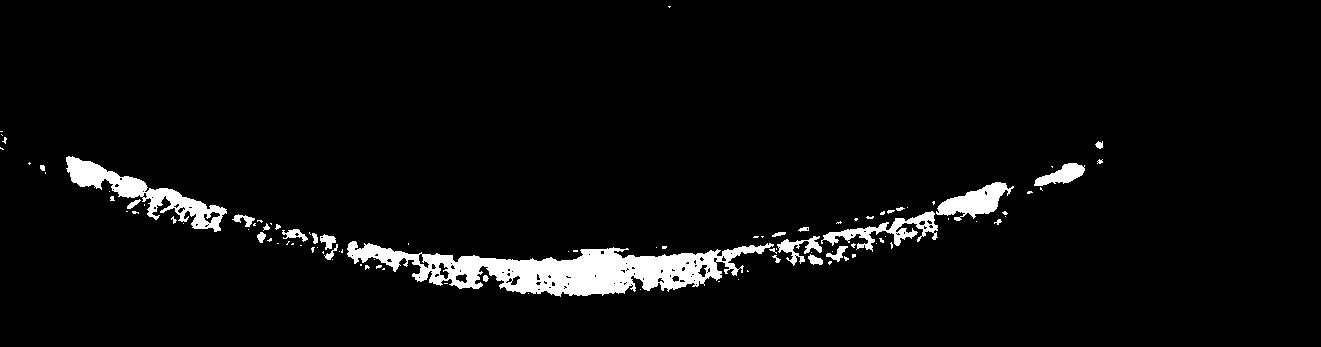

Supplement: S8 Data — (ZIP) [file pone.0297284.s008.zip › Level 4 processed Sample/processed_2/latex/WSO_latex.jpg]

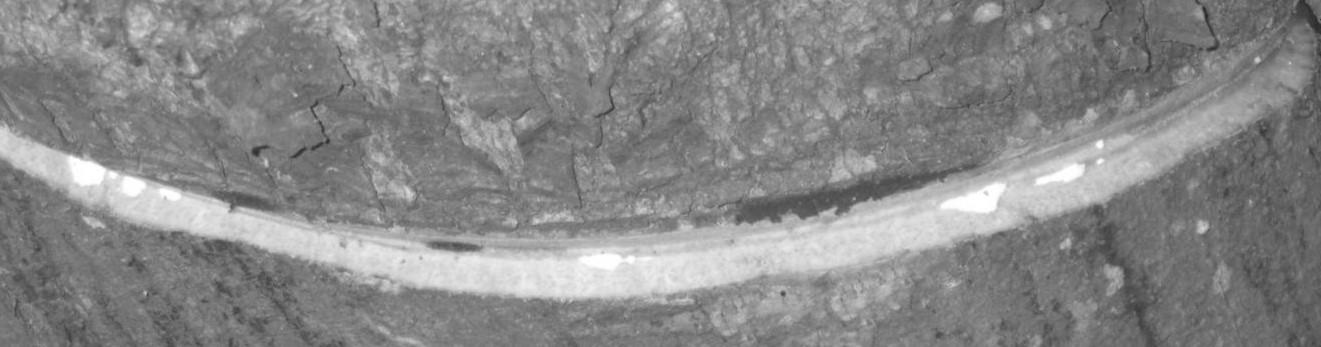

Supplement: S8 Data — (ZIP) [file pone.0297284.s008.zip › Level 4 processed Sample/processed_2/original_image.jpg]

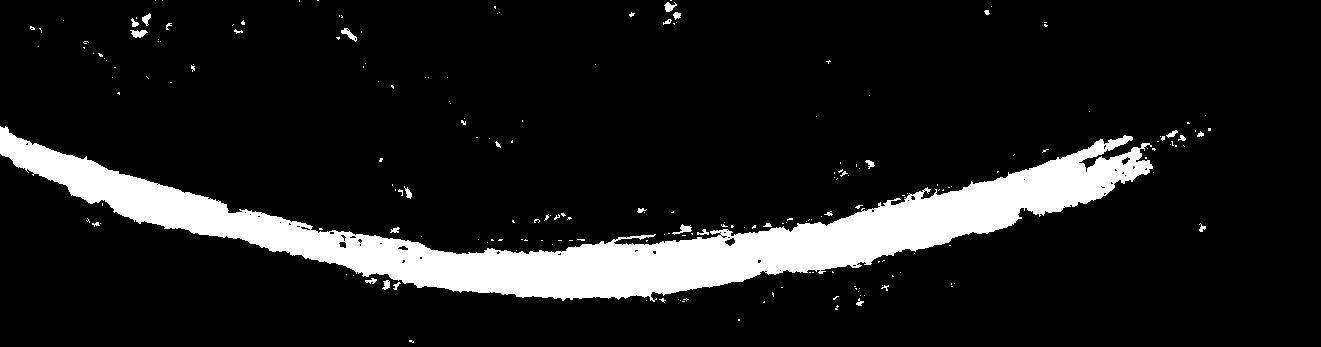

Supplement: S8 Data — (ZIP) [file pone.0297284.s008.zip › Level 4 processed Sample/processed_2/scar/AHA_scar.jpg]

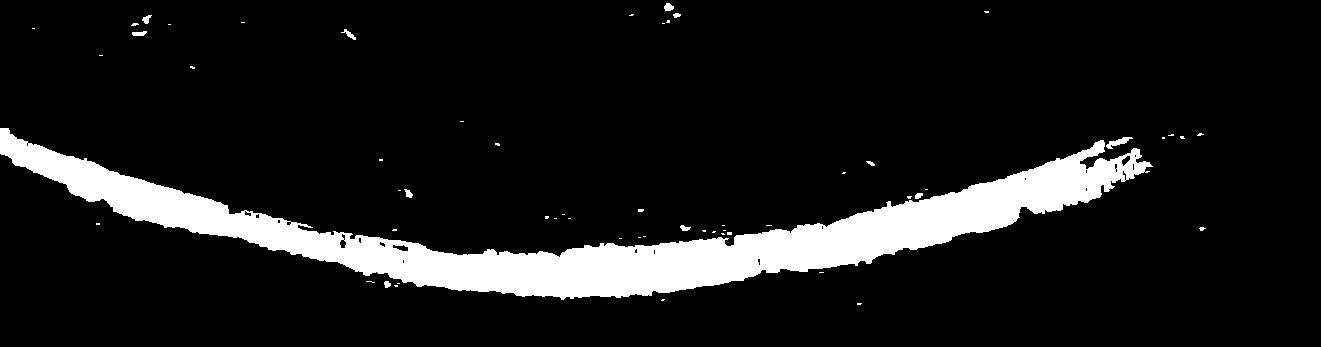

Supplement: S8 Data — (ZIP) [file pone.0297284.s008.zip › Level 4 processed Sample/processed_2/scar/DBO_scar.jpg]

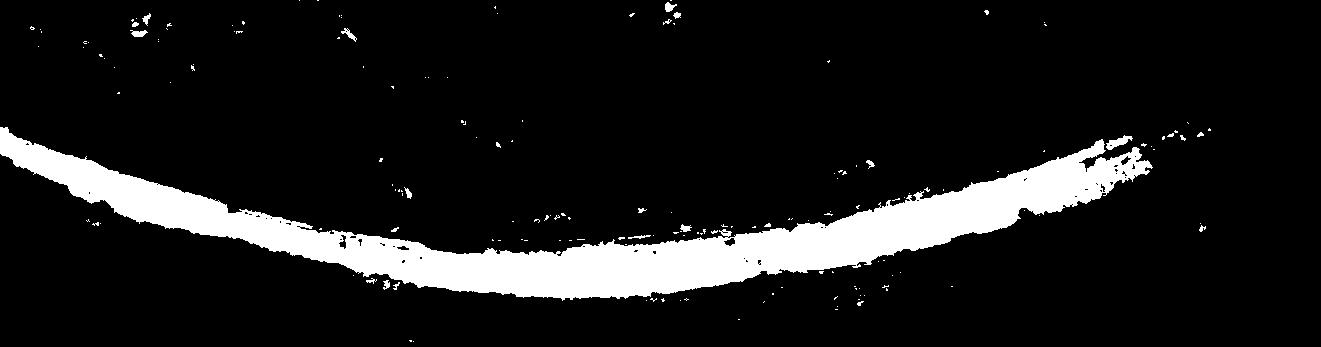

Supplement: S8 Data — (ZIP) [file pone.0297284.s008.zip › Level 4 processed Sample/processed_2/scar/GWO_scar.jpg]

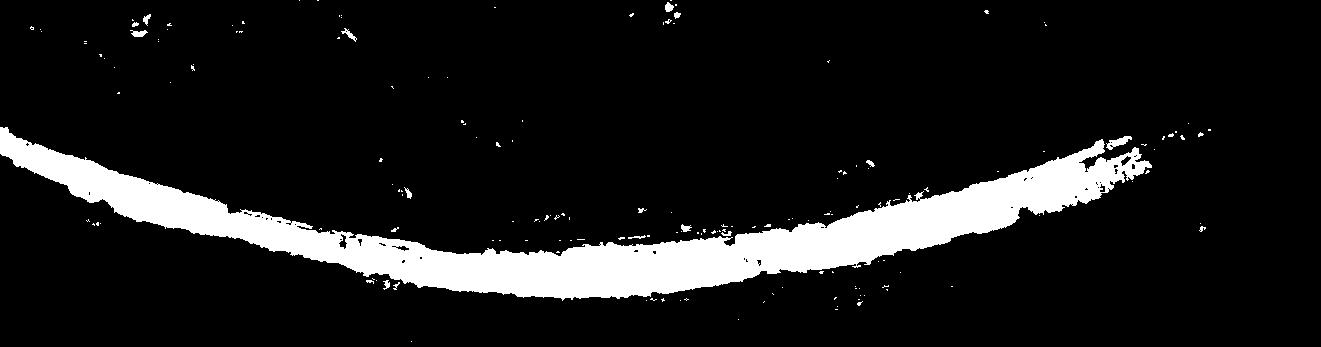

Supplement: S8 Data — (ZIP) [file pone.0297284.s008.zip › Level 4 processed Sample/processed_2/scar/WSO_scar.jpg]

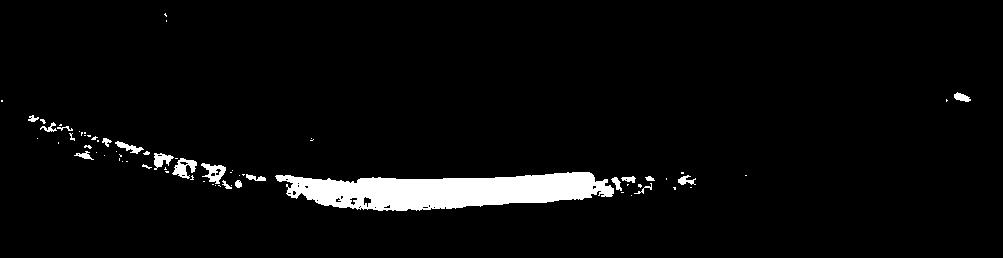

Supplement: S8 Data — (ZIP) [file pone.0297284.s008.zip › Level 4 processed Sample/processed_20/latex/AHA_latex.jpg]

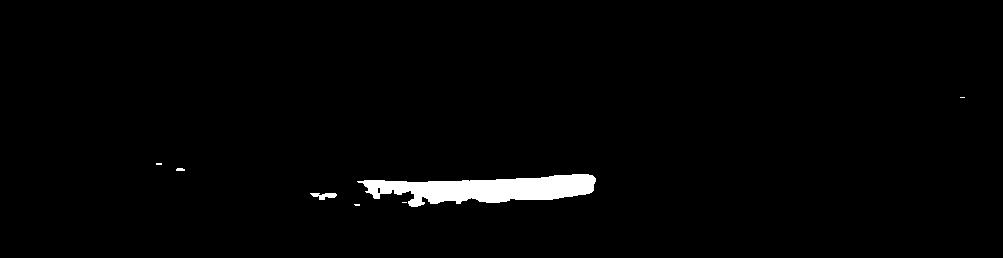

Supplement: S8 Data — (ZIP) [file pone.0297284.s008.zip › Level 4 processed Sample/processed_20/latex/DBO_latex.jpg]

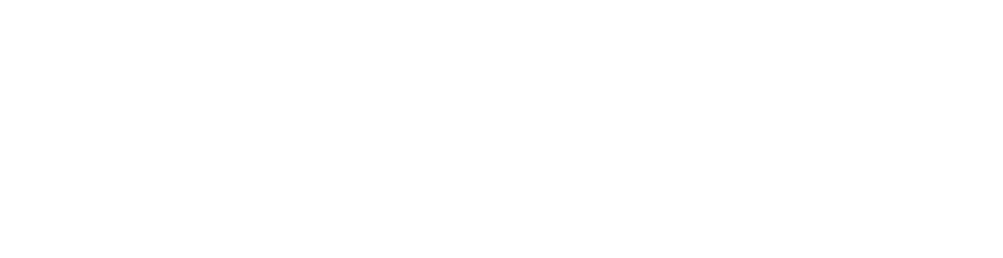

Supplement: S8 Data — (ZIP) [file pone.0297284.s008.zip › Level 4 processed Sample/processed_20/latex/OTSU_latex.jpg]

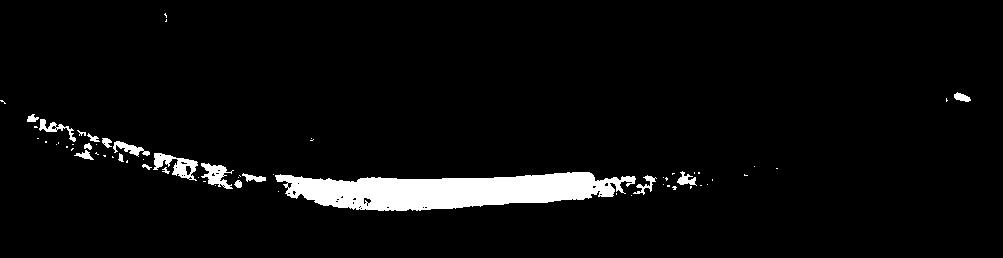

Supplement: S8 Data — (ZIP) [file pone.0297284.s008.zip › Level 4 processed Sample/processed_20/latex/WOA_latex.jpg]

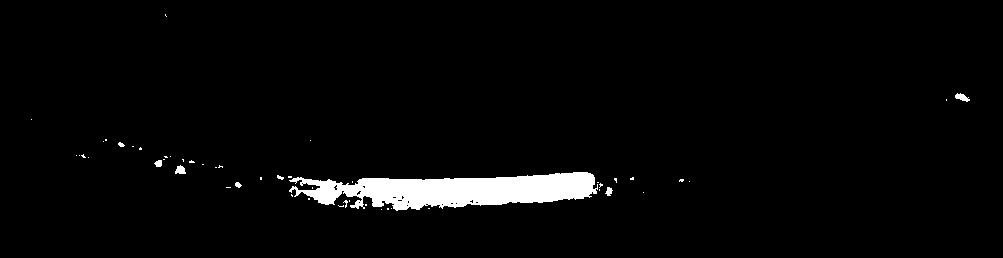

Supplement: S8 Data — (ZIP) [file pone.0297284.s008.zip › Level 4 processed Sample/processed_20/latex/WSO_latex.jpg]

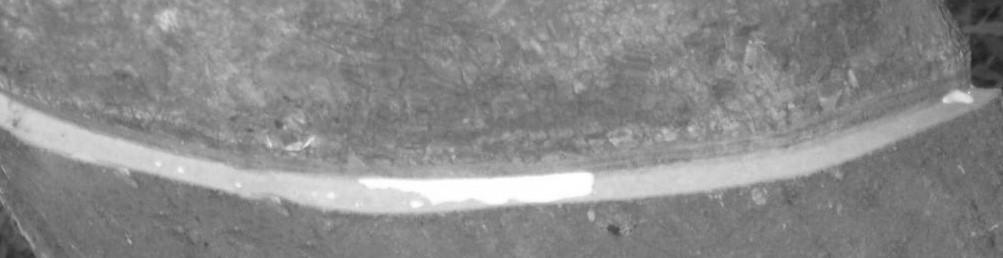

Supplement: S8 Data — (ZIP) [file pone.0297284.s008.zip › Level 4 processed Sample/processed_20/original_image.jpg]

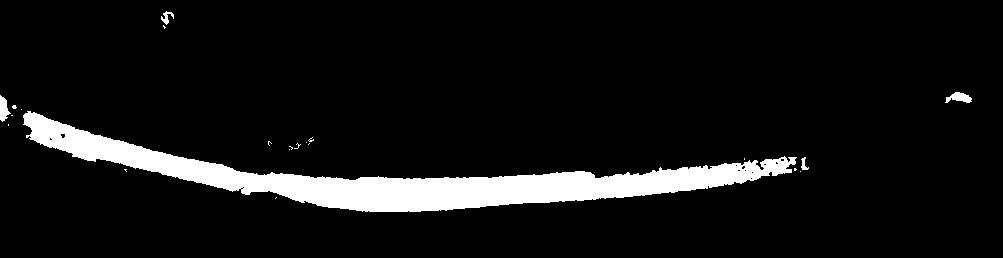

Supplement: S8 Data — (ZIP) [file pone.0297284.s008.zip › Level 4 processed Sample/processed_20/scar/AHA_scar.jpg]

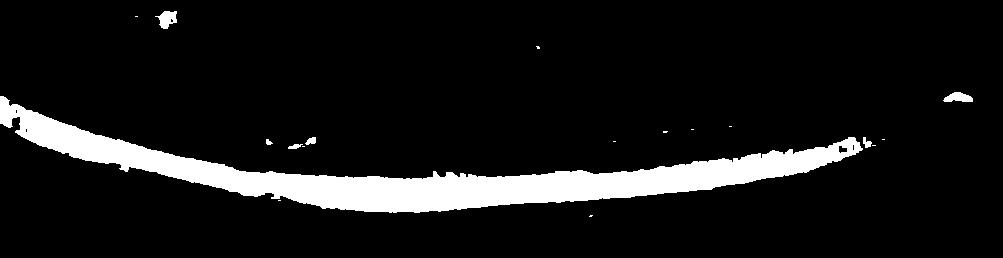

Supplement: S8 Data — (ZIP) [file pone.0297284.s008.zip › Level 4 processed Sample/processed_20/scar/DBO_scar.jpg]

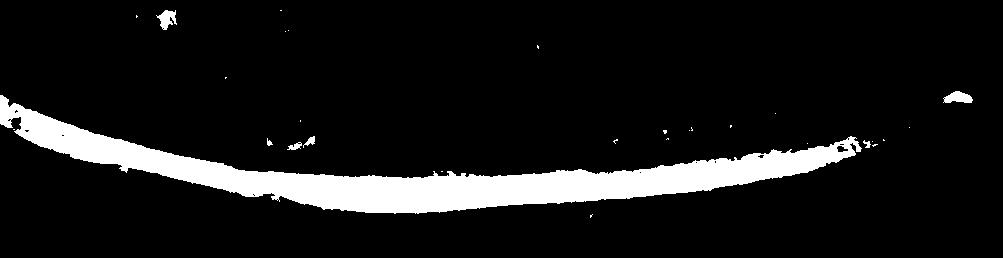

Supplement: S8 Data — (ZIP) [file pone.0297284.s008.zip › Level 4 processed Sample/processed_20/scar/WOA_scar.jpg]

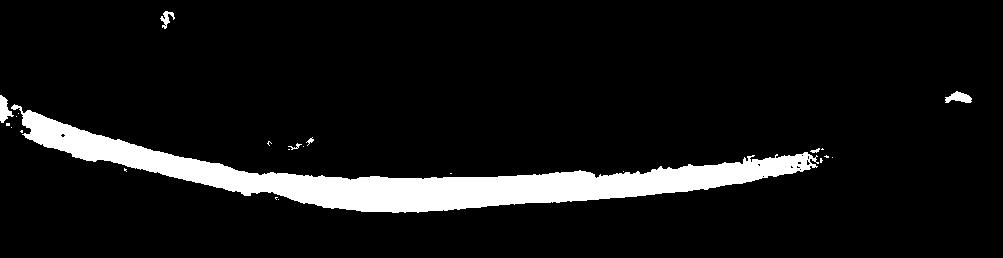

Supplement: S8 Data — (ZIP) [file pone.0297284.s008.zip › Level 4 processed Sample/processed_20/scar/WSO_scar.jpg]

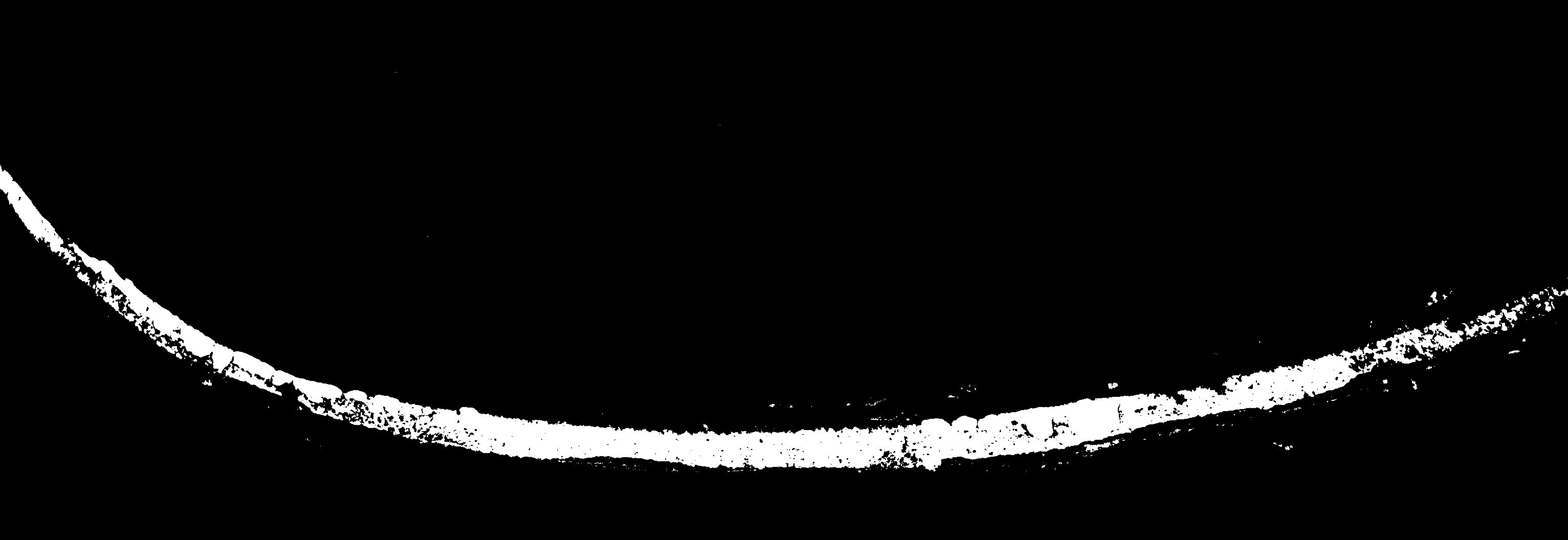

Supplement: S8 Data — (ZIP) [file pone.0297284.s008.zip › Level 4 processed Sample/processed_3/latex/AHA_latex.jpg]

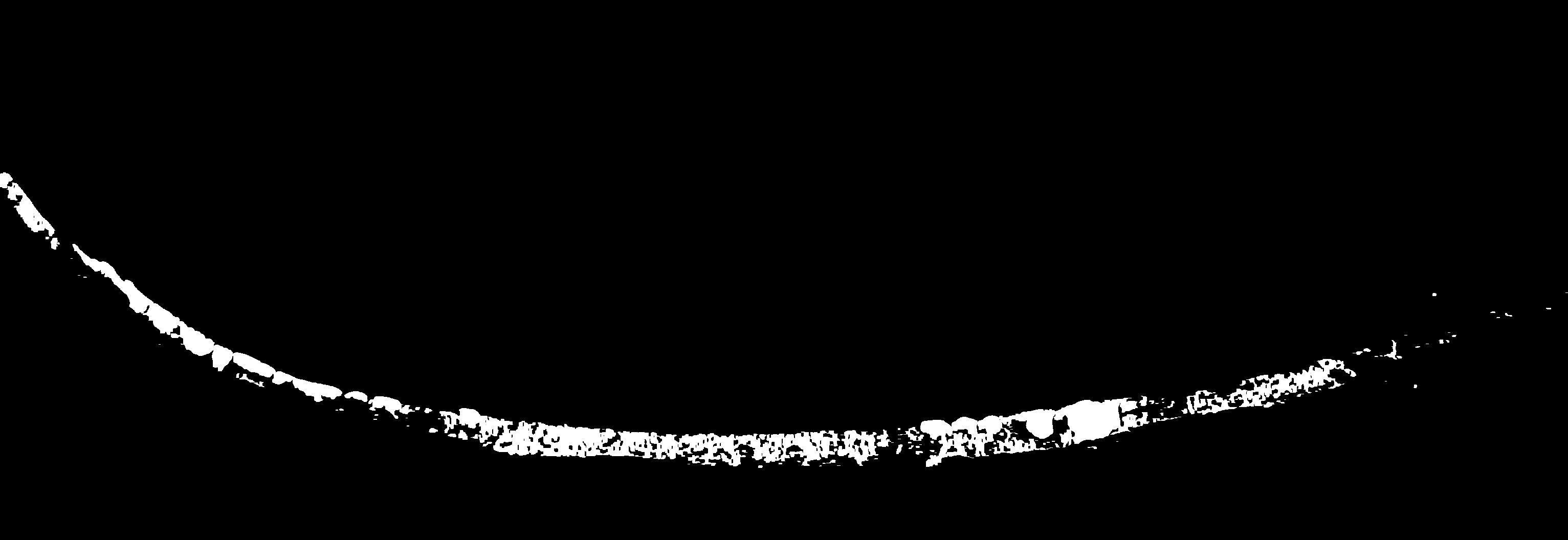

Supplement: S8 Data — (ZIP) [file pone.0297284.s008.zip › Level 4 processed Sample/processed_3/latex/DBO_latex.jpg]

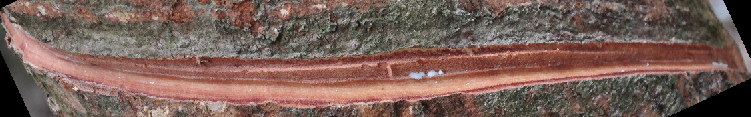

Supplement: S9 Data — (ZIP) [file pone.0297284.s009.zip › Level 5 Original Sample/5-1.jpg]

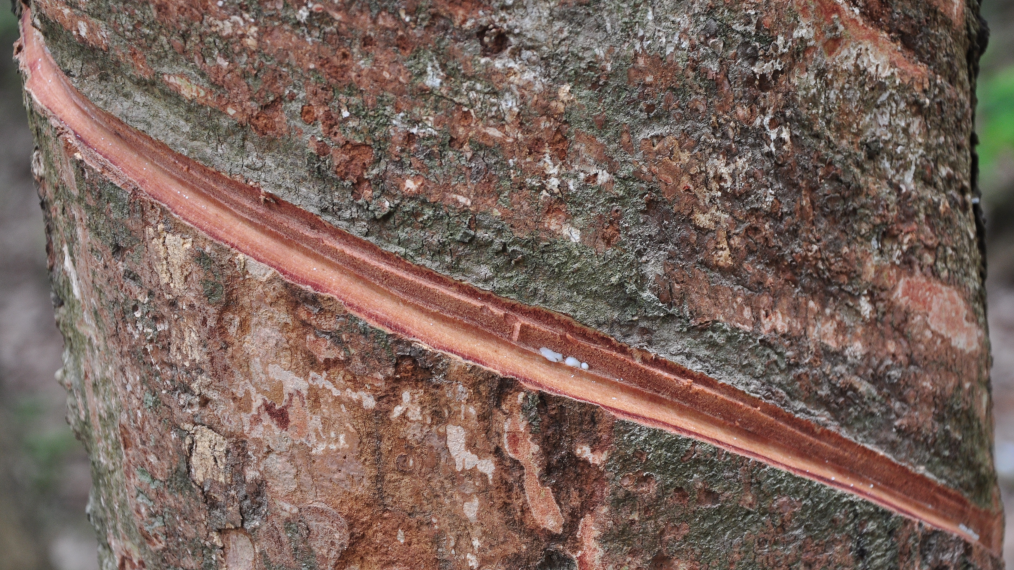

Supplement: S9 Data — (ZIP) [file pone.0297284.s009.zip › Level 5 Original Sample/5-1.png]

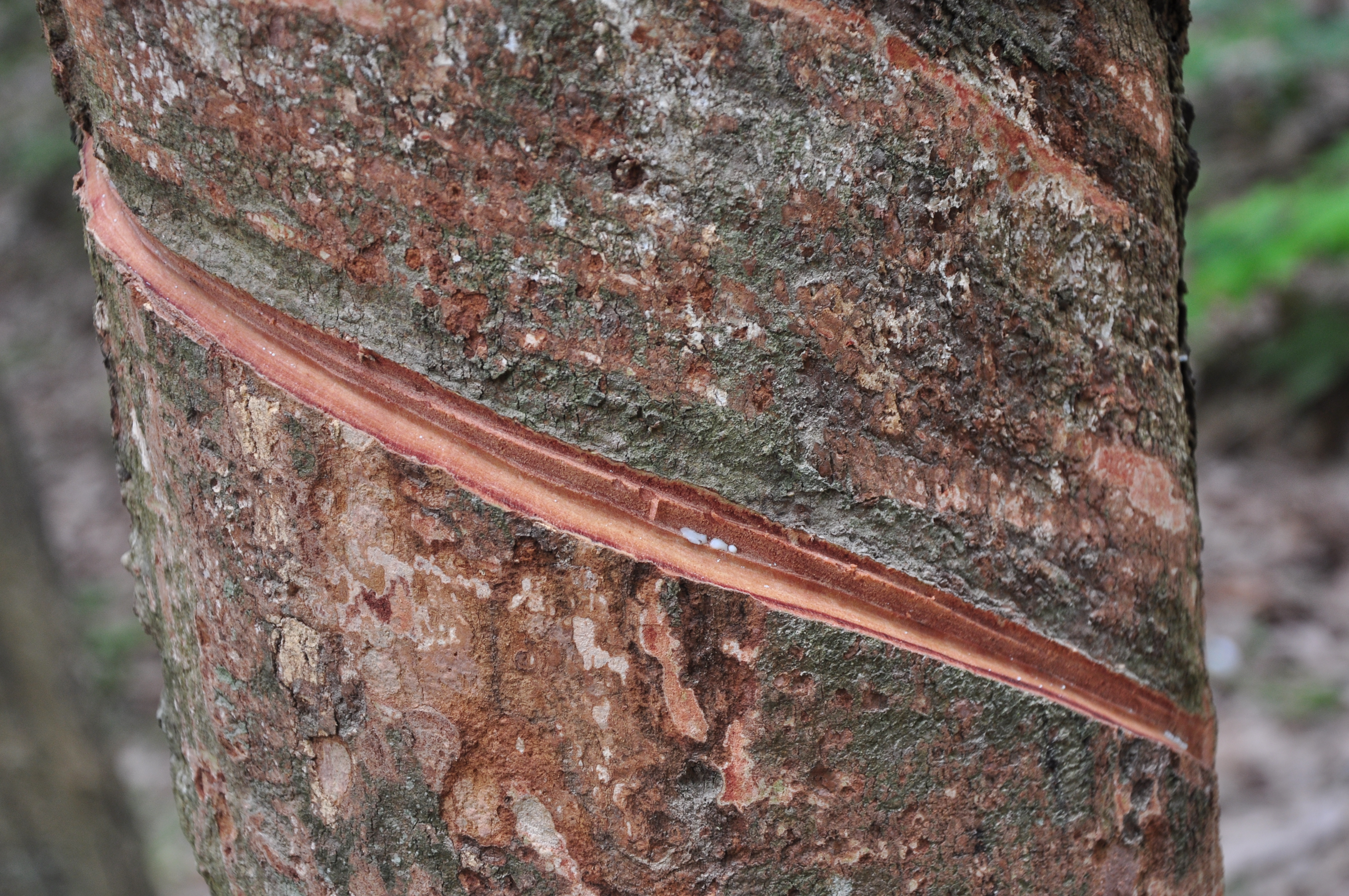

Supplement: S9 Data — (ZIP) [file pone.0297284.s009.zip › Level 5 Original Sample/5-144-20140528-0298.JPG]

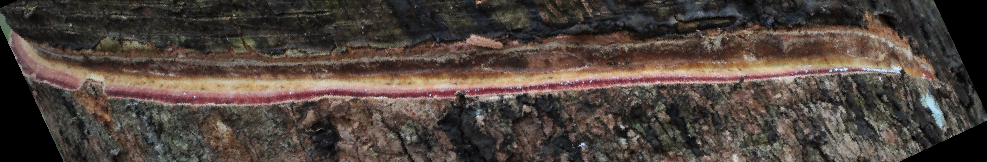

Supplement: S9 Data — (ZIP) [file pone.0297284.s009.zip › Level 5 Original Sample/5-2.jpg]

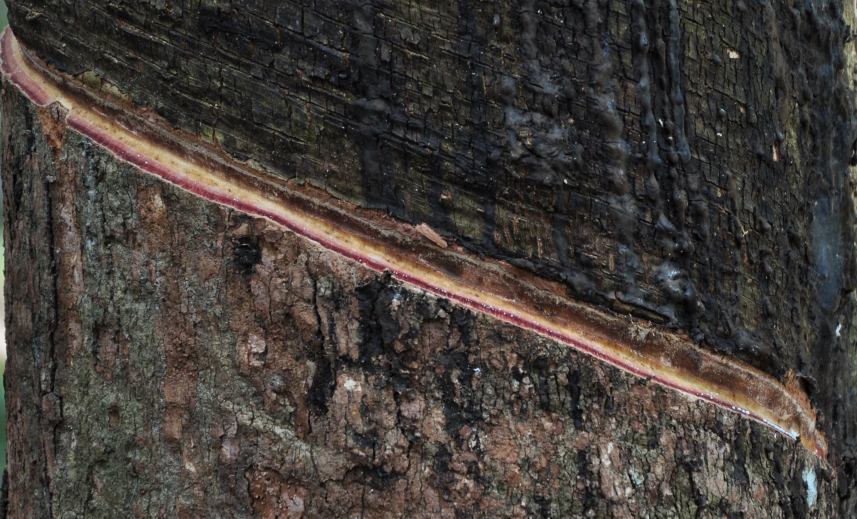

Supplement: S9 Data — (ZIP) [file pone.0297284.s009.zip › Level 5 Original Sample/5-2.png]

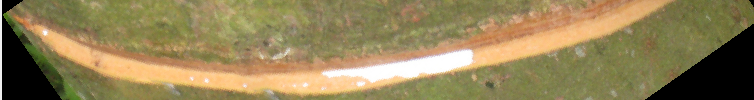

Supplement: S9 Data — (ZIP) [file pone.0297284.s009.zip › Level 5 Original Sample/5-3.jpg]

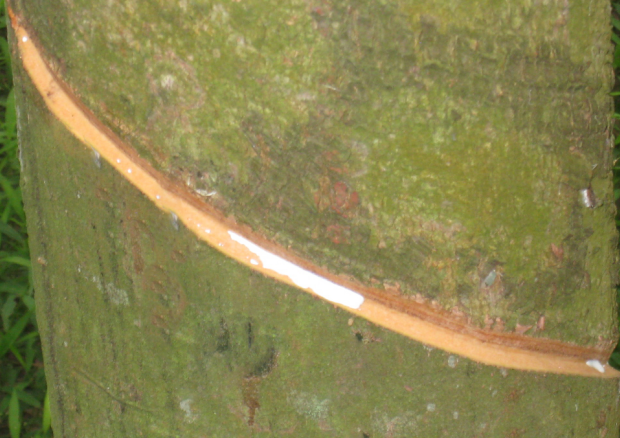

Supplement: S9 Data — (ZIP) [file pone.0297284.s009.zip › Level 5 Original Sample/5-3.png]

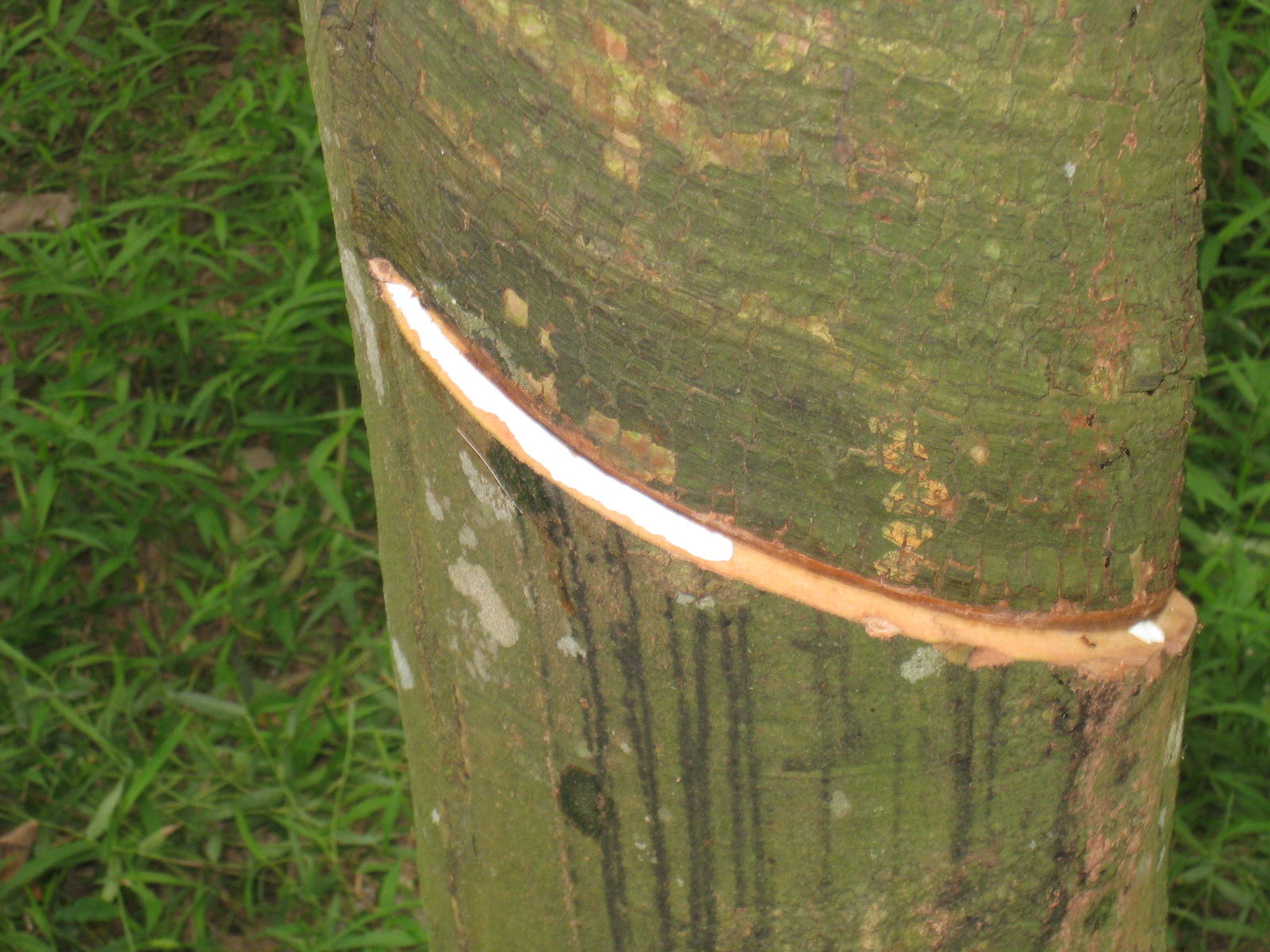

Supplement: S9 Data — (ZIP) [file pone.0297284.s009.zip › Level 5 Original Sample/5-33701-198-20140512-0010.JPG]

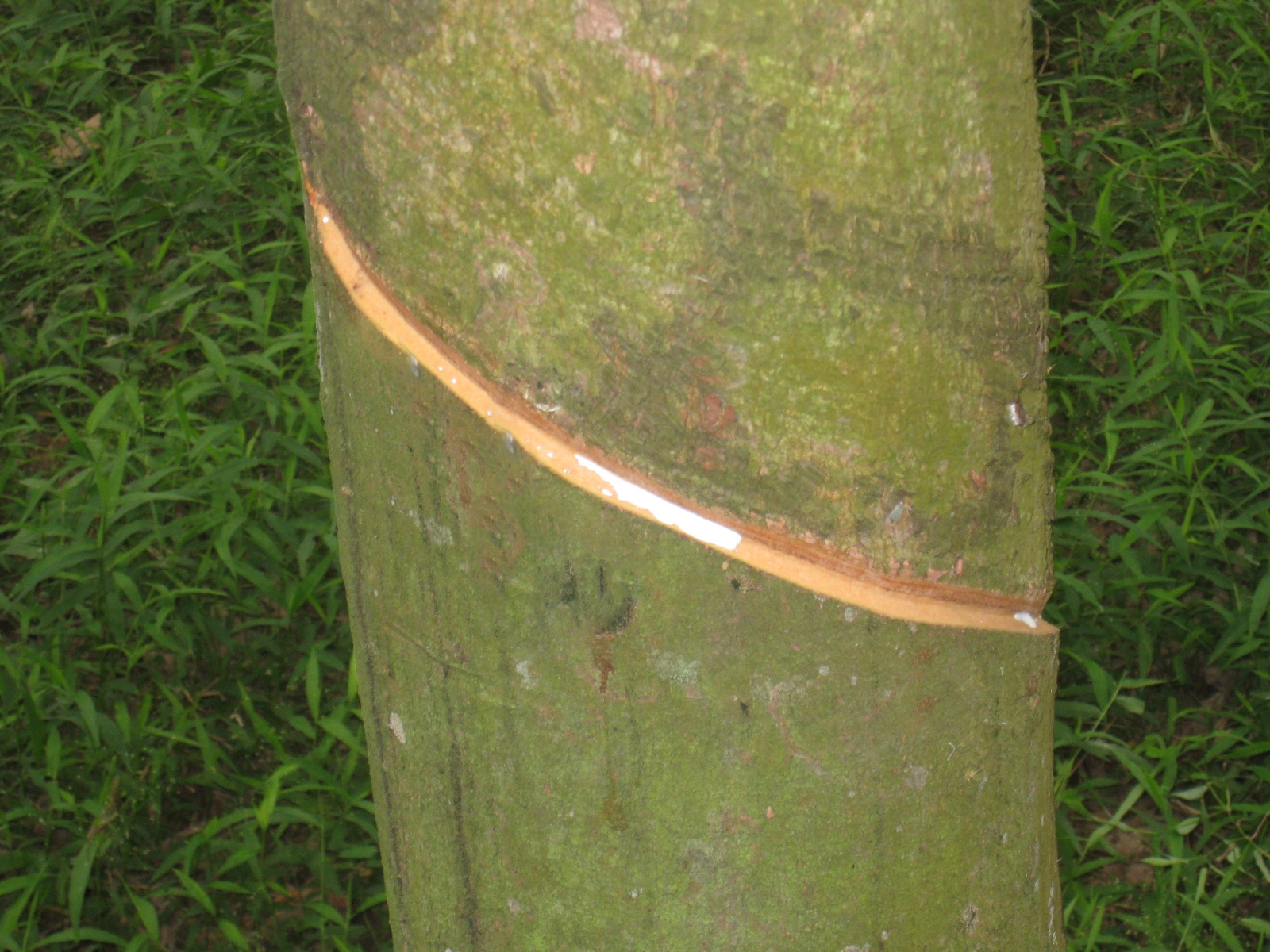

Supplement: S9 Data — (ZIP) [file pone.0297284.s009.zip › Level 5 Original Sample/5-33701-199-20140512-0008.JPG]

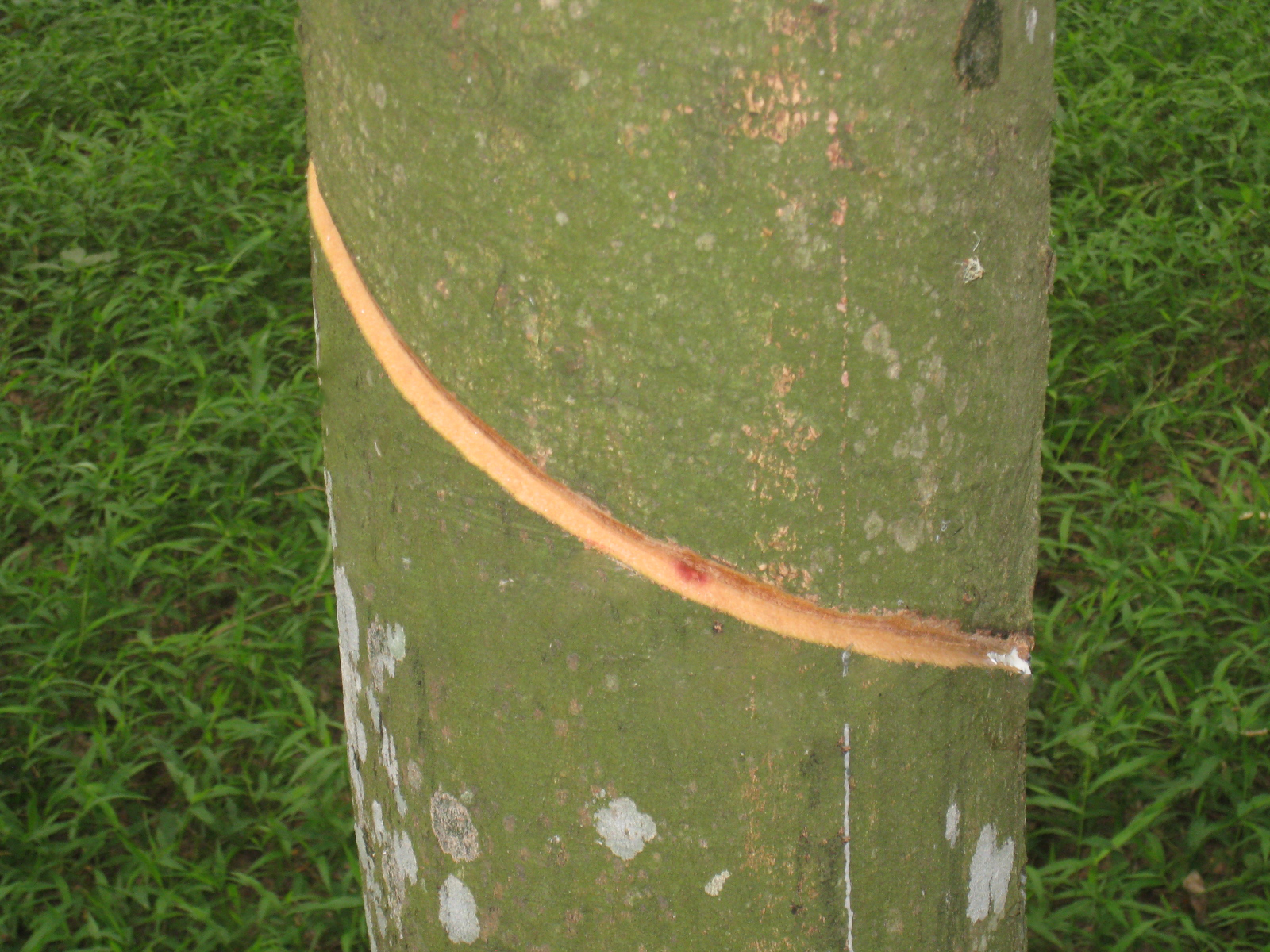

Supplement: S9 Data — (ZIP) [file pone.0297284.s009.zip › Level 5 Original Sample/5-33701-201-20140512-0006.JPG]

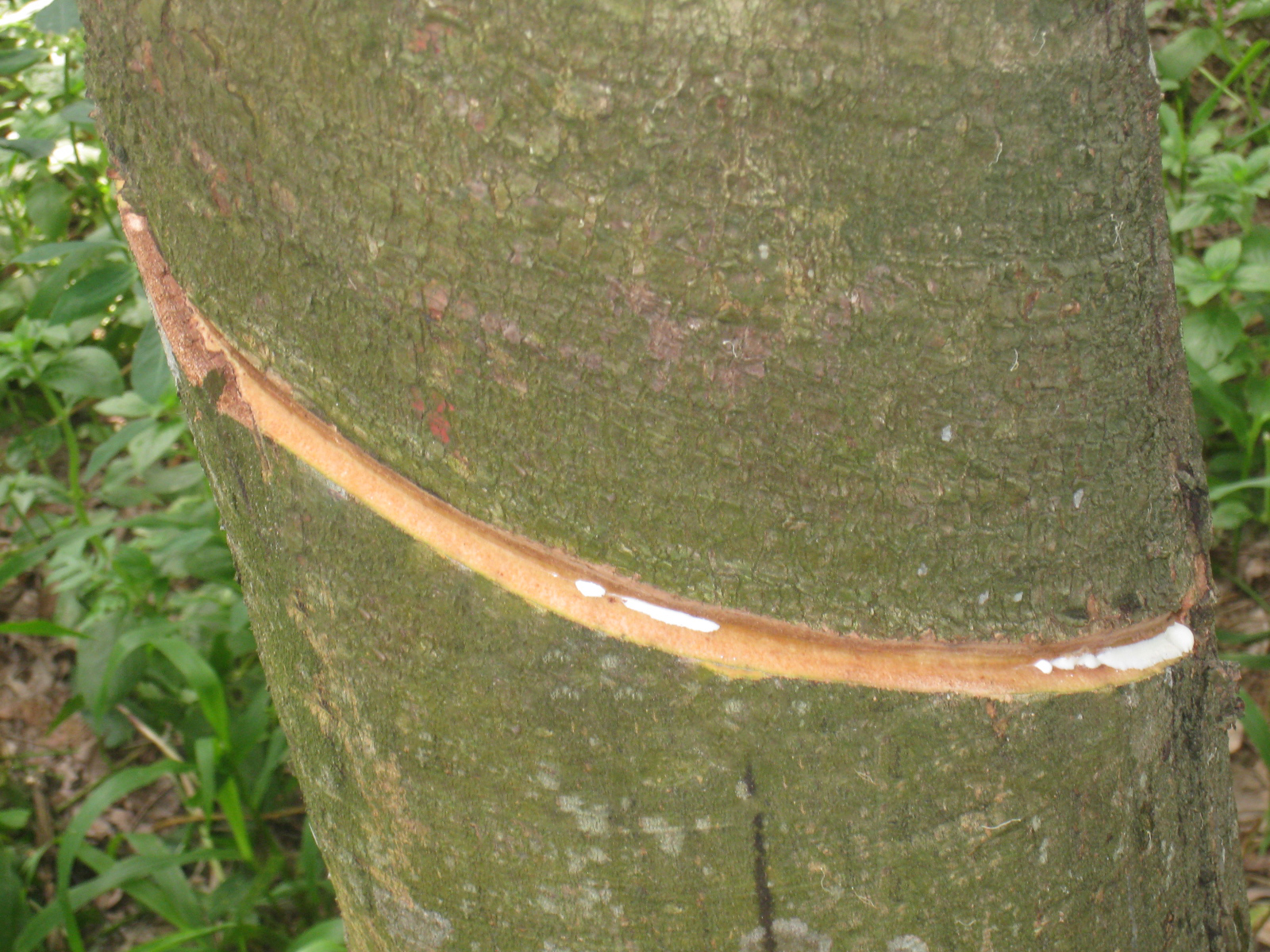

Supplement: S9 Data — (ZIP) [file pone.0297284.s009.zip › Level 5 Original Sample/5-33701-297-20140512-0028.JPG]

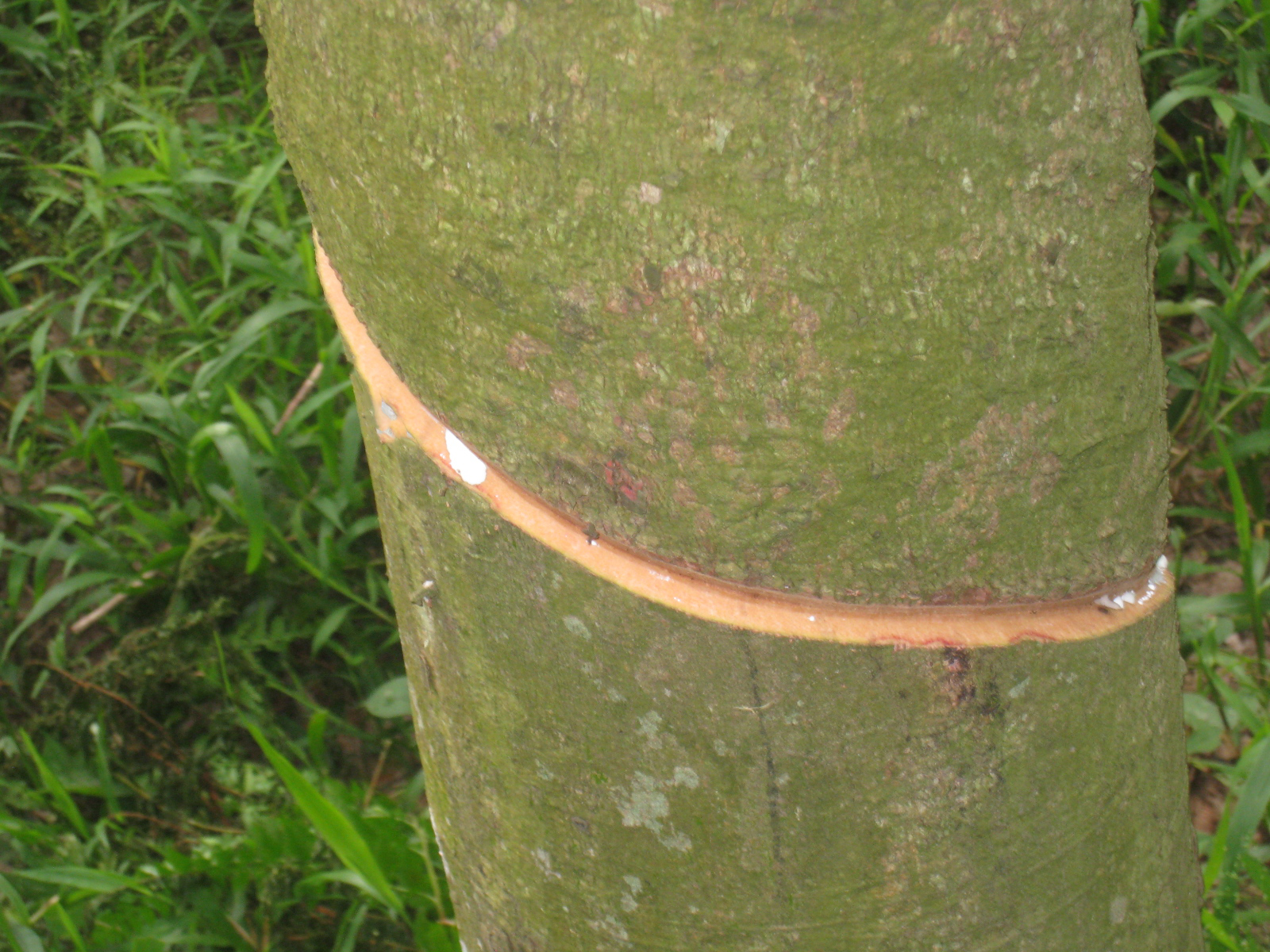

Supplement: S9 Data — (ZIP) [file pone.0297284.s009.zip › Level 5 Original Sample/5-33702-042-20140512-0044.JPG]

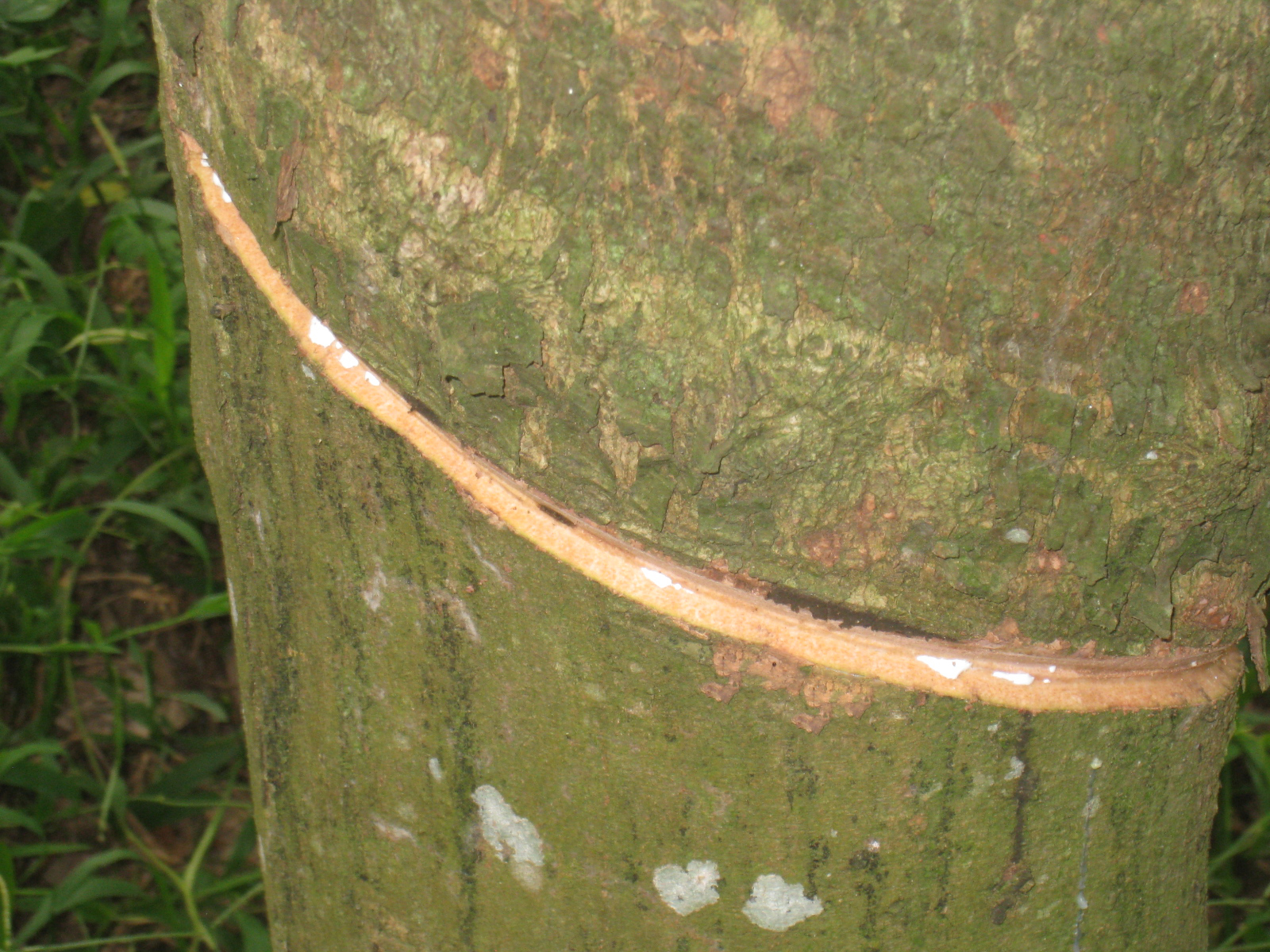

Supplement: S9 Data — (ZIP) [file pone.0297284.s009.zip › Level 5 Original Sample/5-33702-061-20140512-0053.JPG]

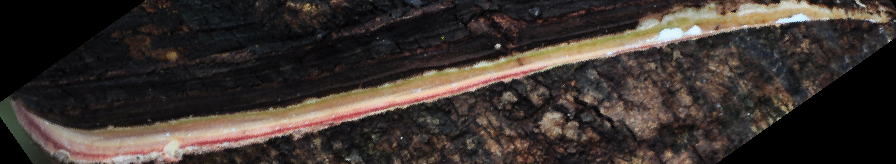

Supplement: S9 Data — (ZIP) [file pone.0297284.s009.zip › Level 5 Original Sample/5-4.jpg]

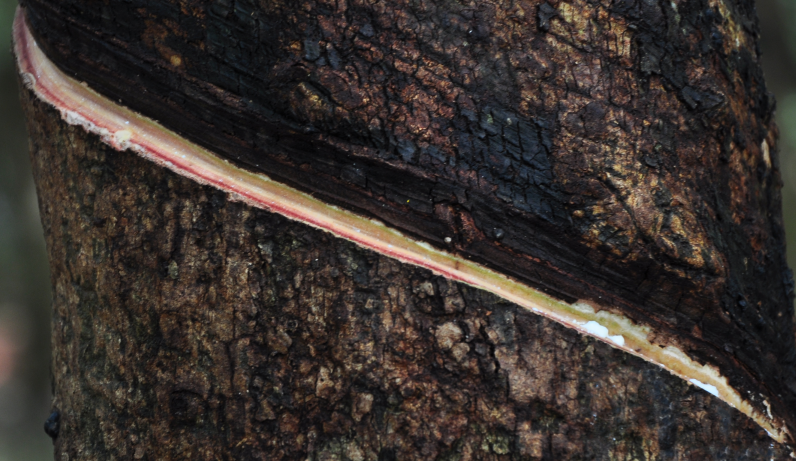

Supplement: S9 Data — (ZIP) [file pone.0297284.s009.zip › Level 5 Original Sample/5-4.png]

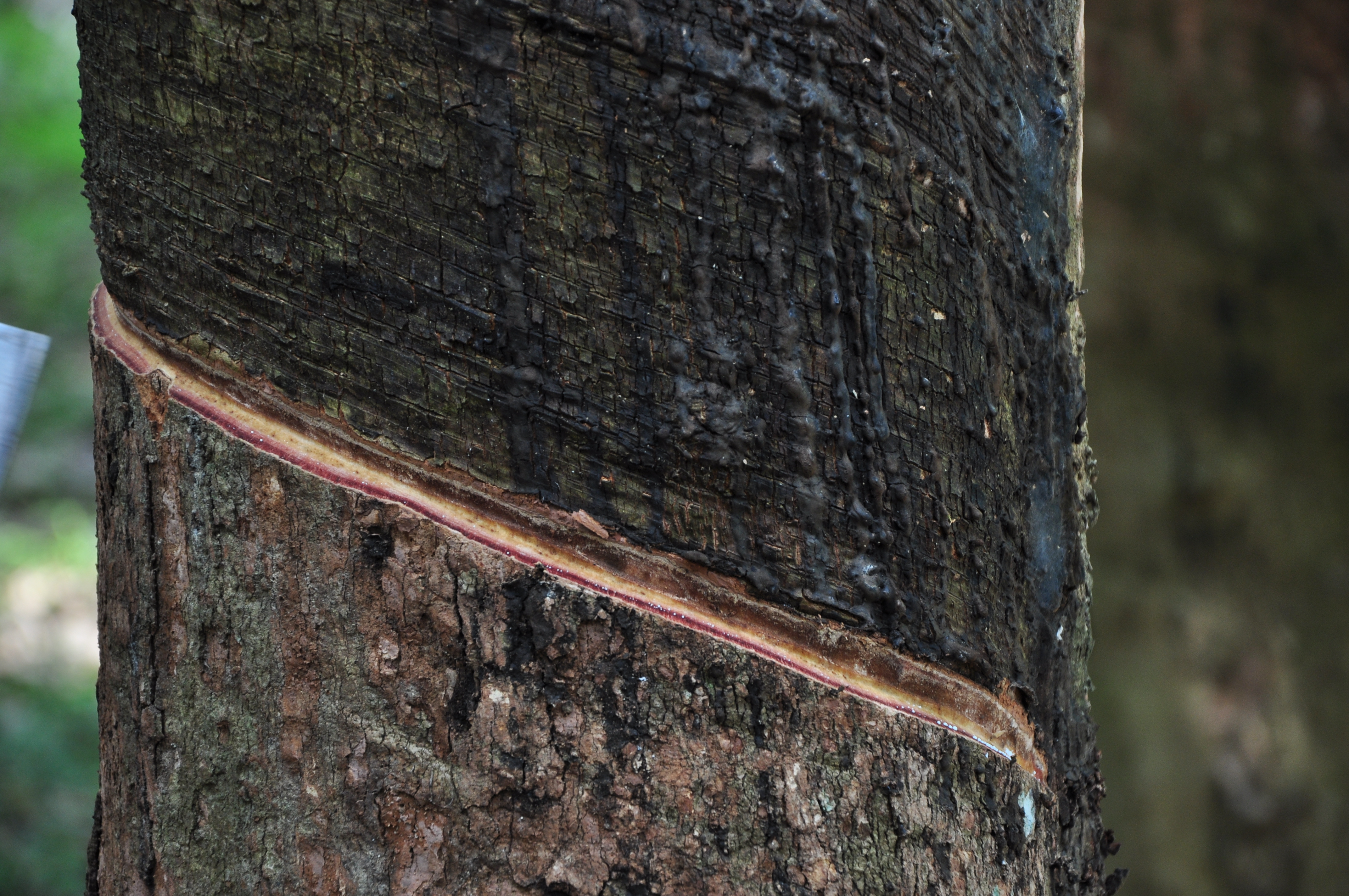

Supplement: S9 Data — (ZIP) [file pone.0297284.s009.zip › Level 5 Original Sample/5-422-20140528-0870.JPG]

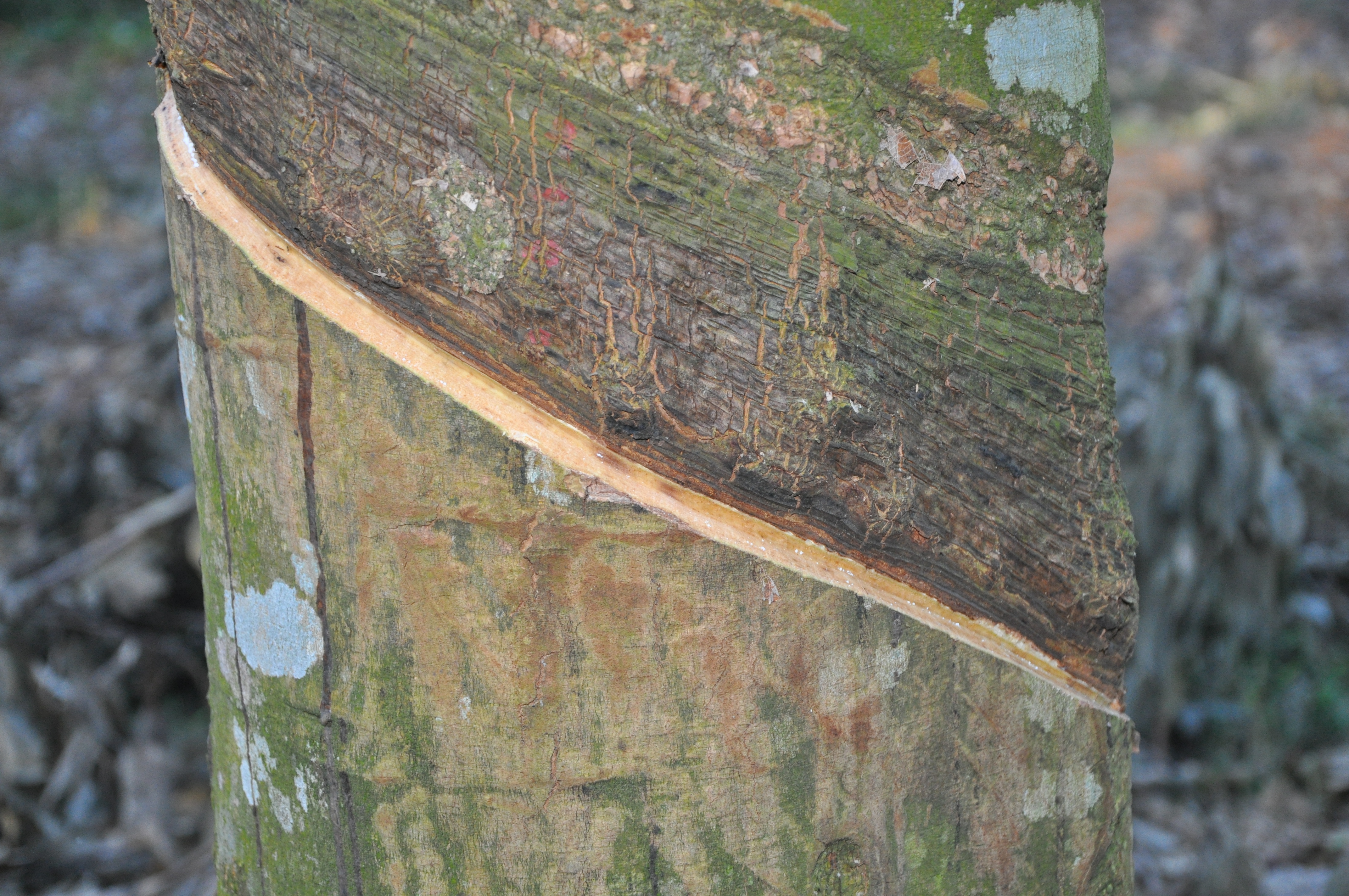

Supplement: S9 Data — (ZIP) [file pone.0297284.s009.zip › Level 5 Original Sample/5-60101-093-20140929-0185.JPG]

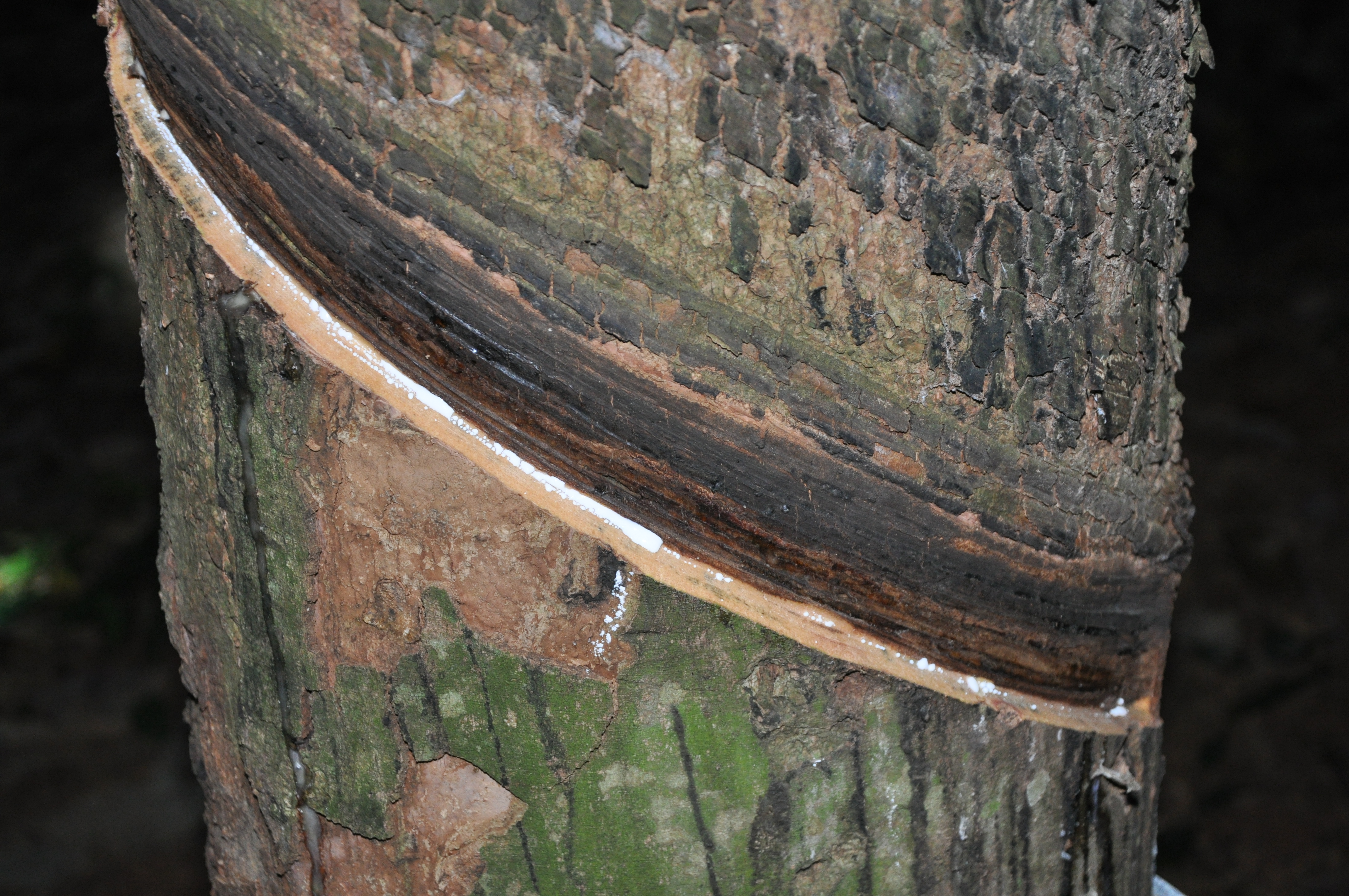

Supplement: S9 Data — (ZIP) [file pone.0297284.s009.zip › Level 5 Original Sample/5-61601-354-20151201-354.JPG]

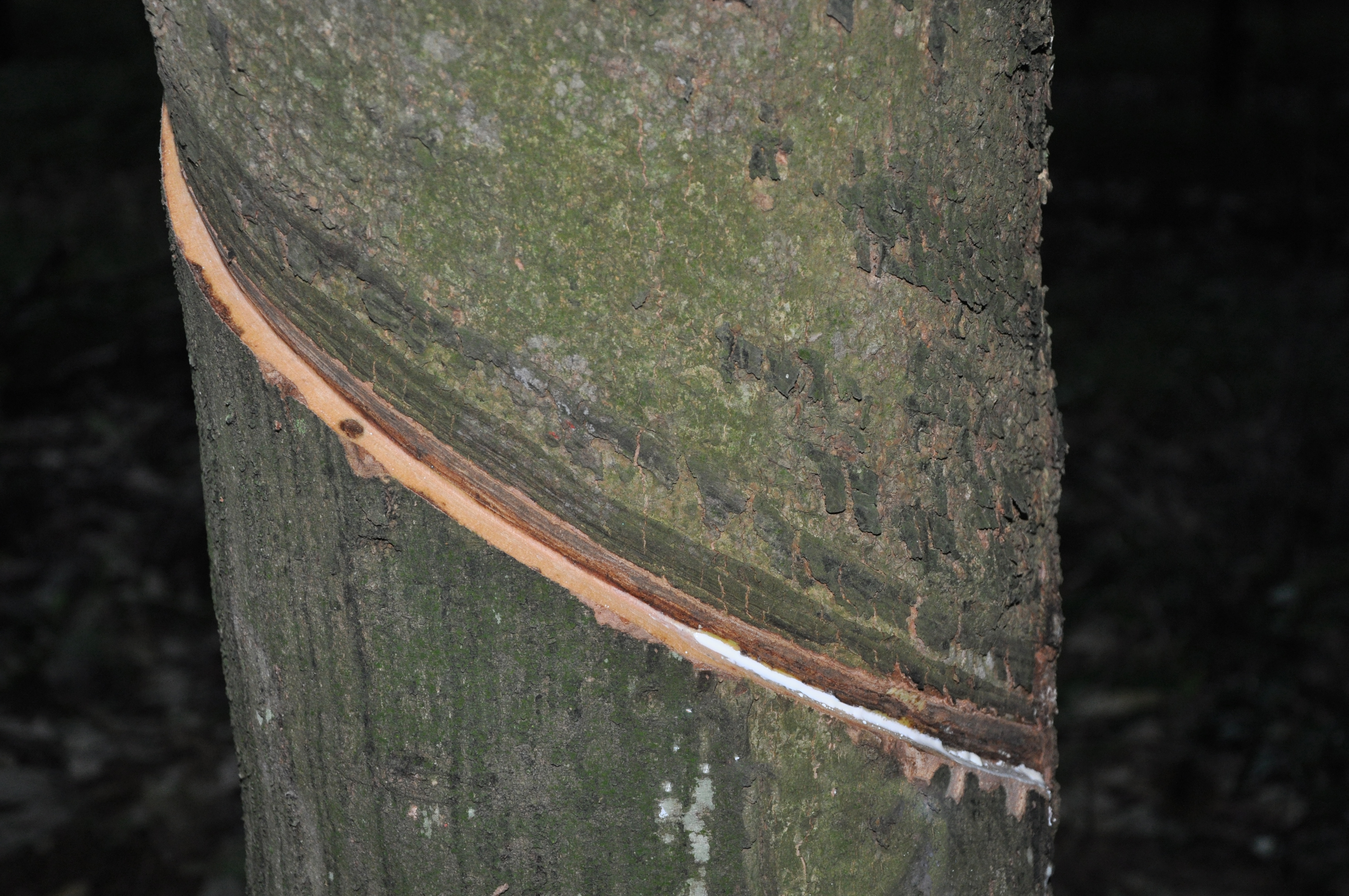

Supplement: S9 Data — (ZIP) [file pone.0297284.s009.zip › Level 5 Original Sample/5-61602-050-20150803-050.JPG]

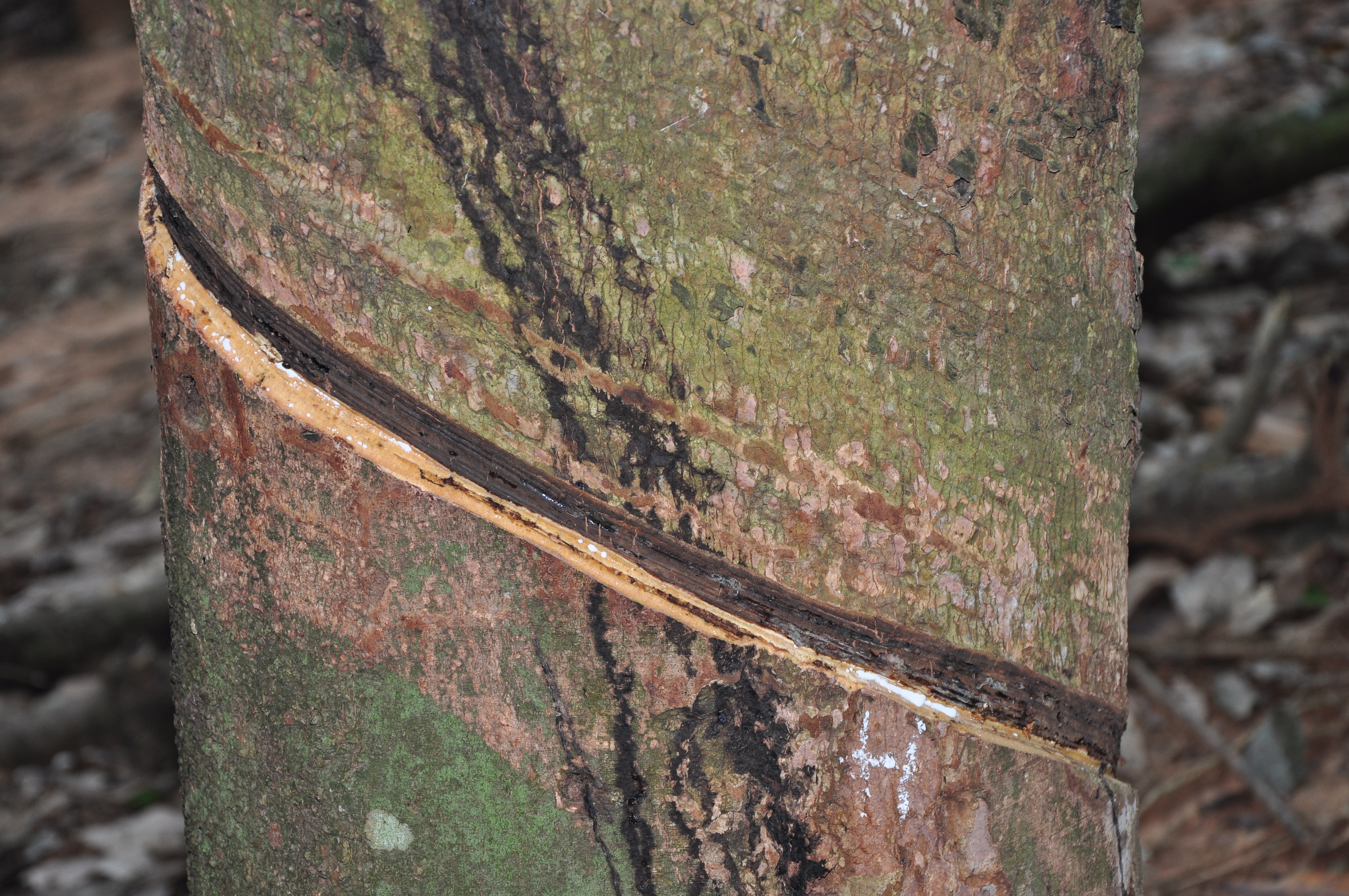

Supplement: S9 Data — (ZIP) [file pone.0297284.s009.zip › Level 5 Original Sample/5-61602-241-20140818-0169.JPG]

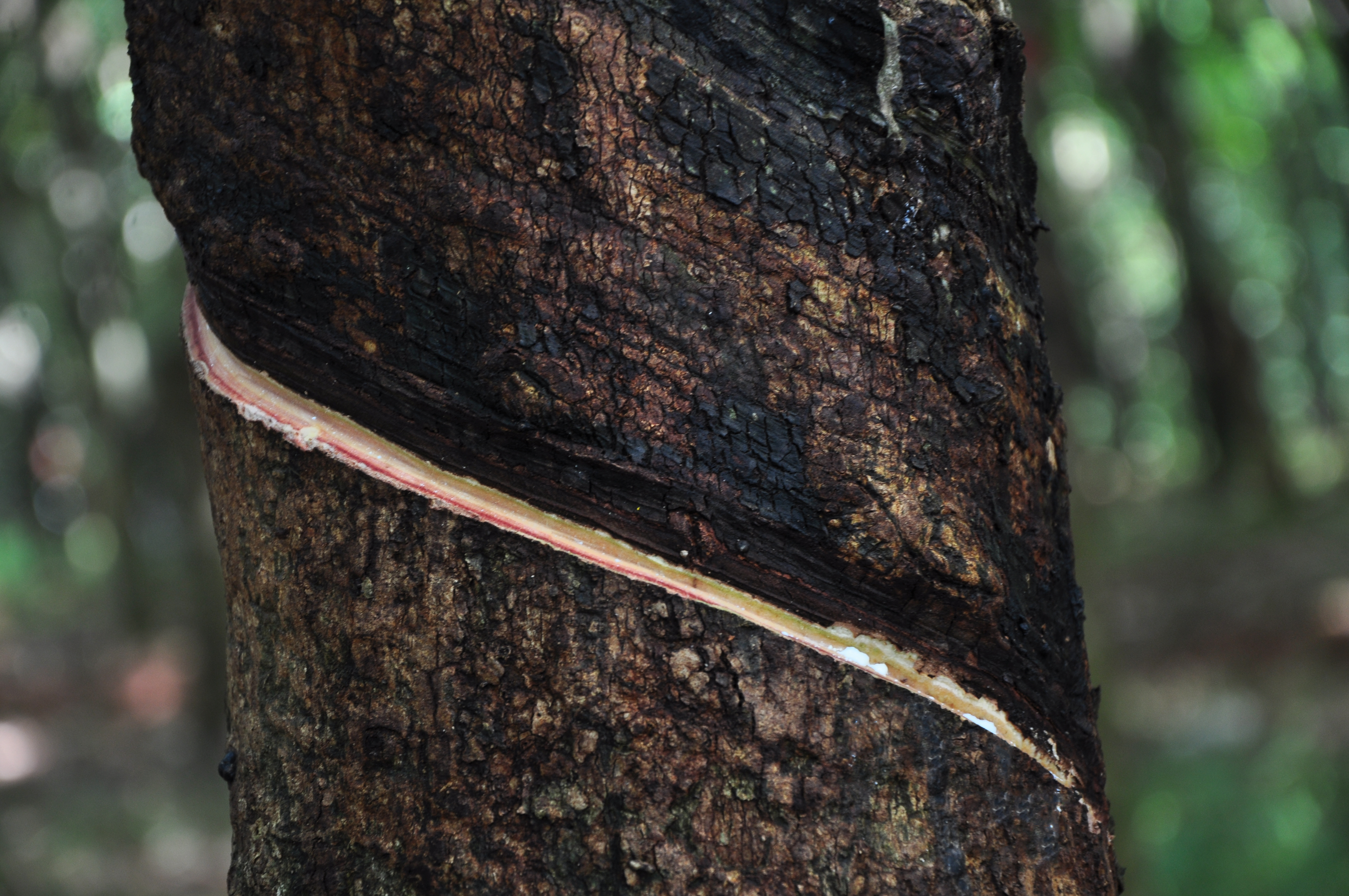

Supplement: S9 Data — (ZIP) [file pone.0297284.s009.zip › Level 5 Original Sample/5-61603-360-20140708-0236.JPG]

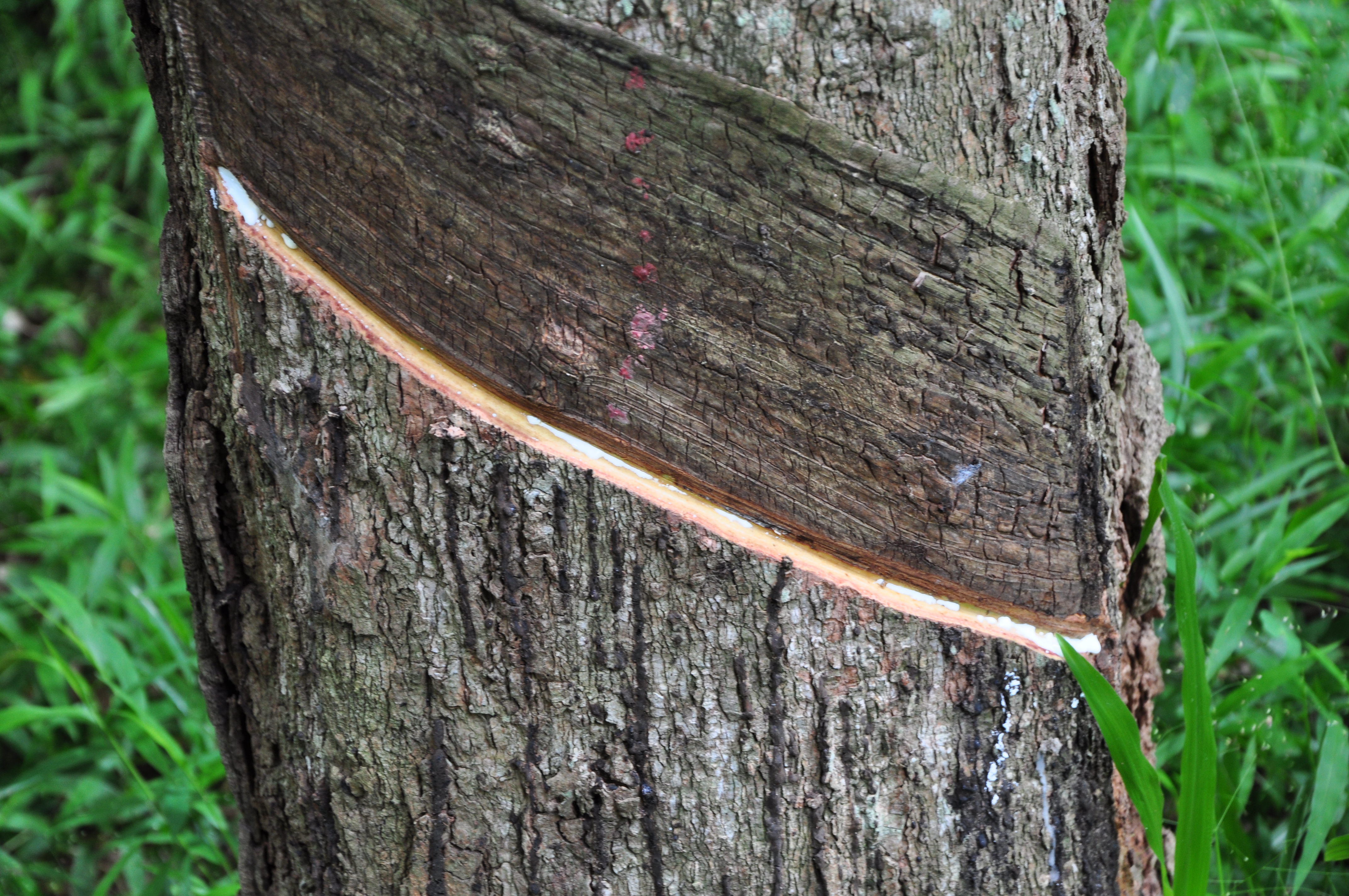

Supplement: S9 Data — (ZIP) [file pone.0297284.s009.zip › Level 5 Original Sample/5-62001-367-20140609-0510.JPG]

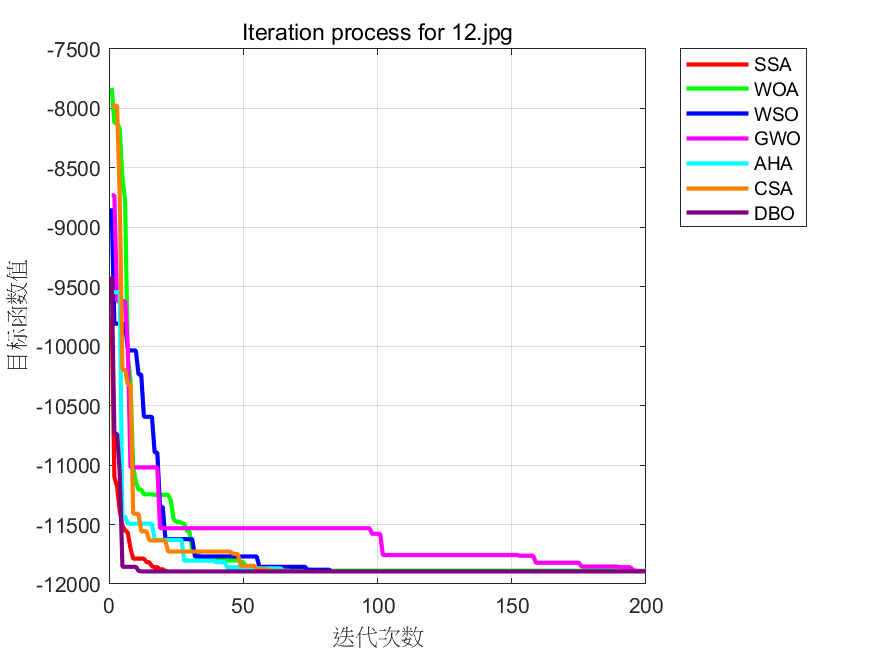

Supplement: S10 Data — (ZIP) [file pone.0297284.s010.zip › Level 5 processed Sample/iteration/12.jpg_iteration.png]

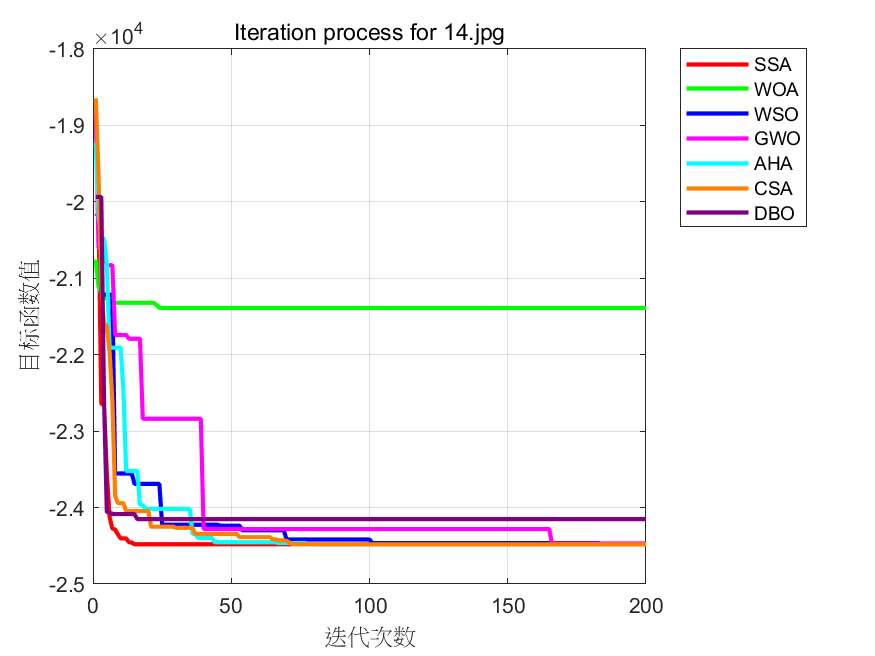

Supplement: S10 Data — (ZIP) [file pone.0297284.s010.zip › Level 5 processed Sample/iteration/14.jpg_iteration.png]

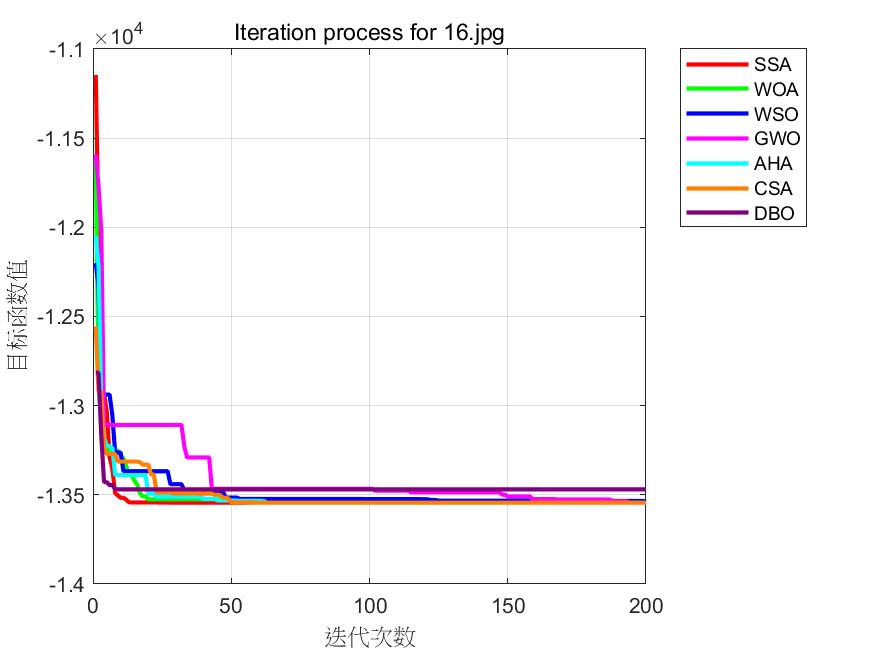

Supplement: S10 Data — (ZIP) [file pone.0297284.s010.zip › Level 5 processed Sample/iteration/16.jpg_iteration.png]

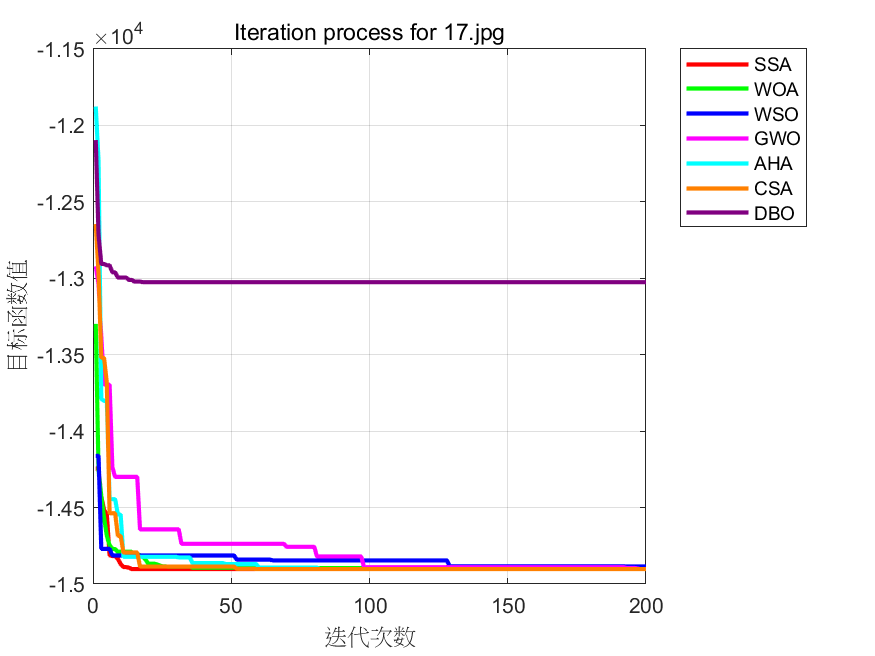

Supplement: S10 Data — (ZIP) [file pone.0297284.s010.zip › Level 5 processed Sample/iteration/17.jpg_iteration.png]

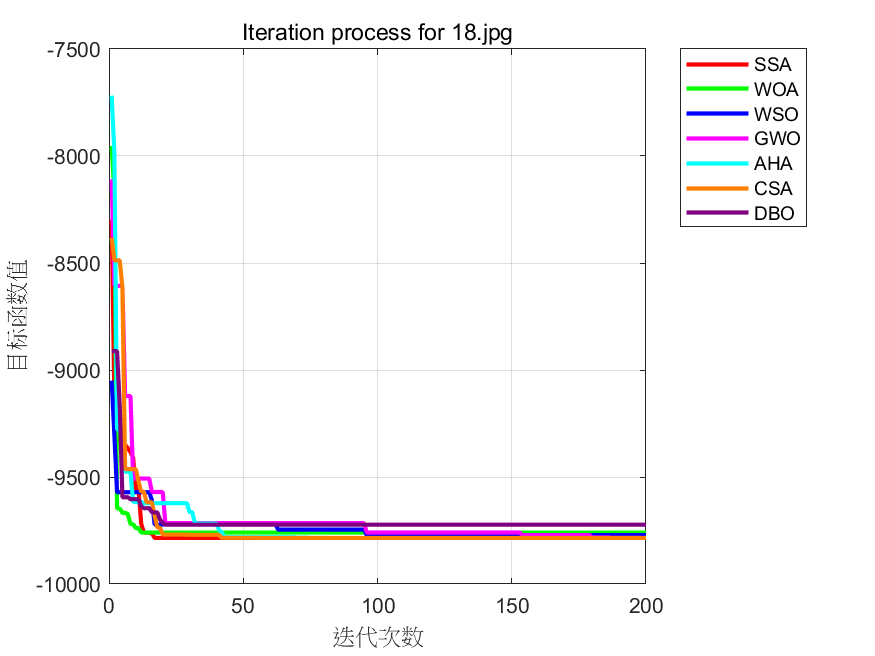

Supplement: S10 Data — (ZIP) [file pone.0297284.s010.zip › Level 5 processed Sample/iteration/18.jpg_iteration.png]

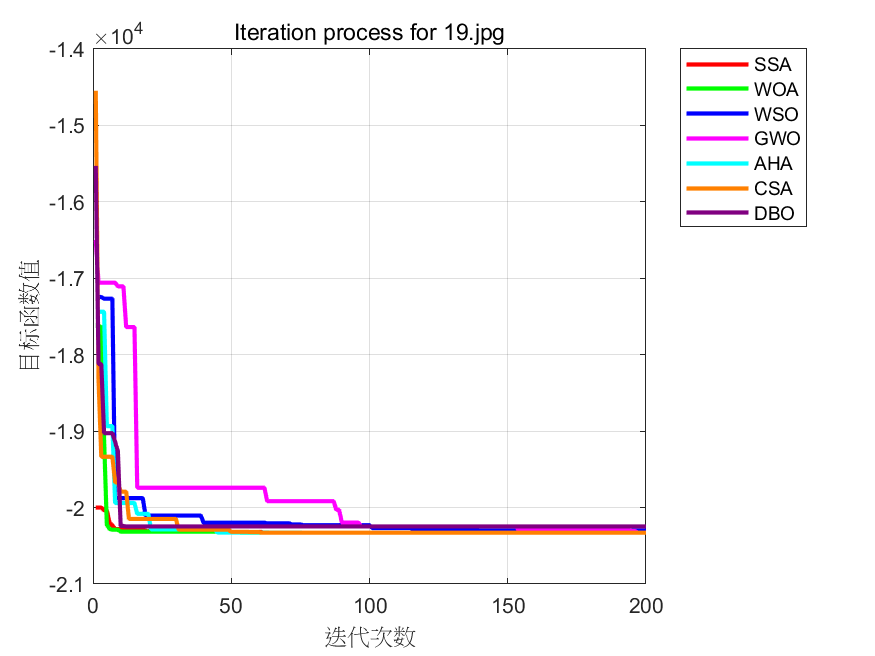

Supplement: S10 Data — (ZIP) [file pone.0297284.s010.zip › Level 5 processed Sample/iteration/19.jpg_iteration.png]

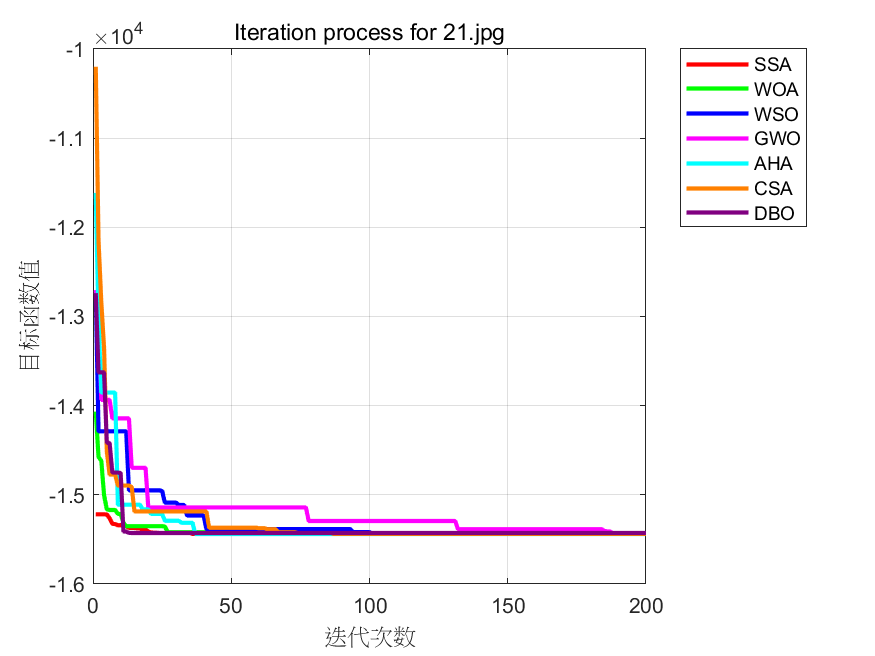

Supplement: S10 Data — (ZIP) [file pone.0297284.s010.zip › Level 5 processed Sample/iteration/21.jpg_iteration.png]

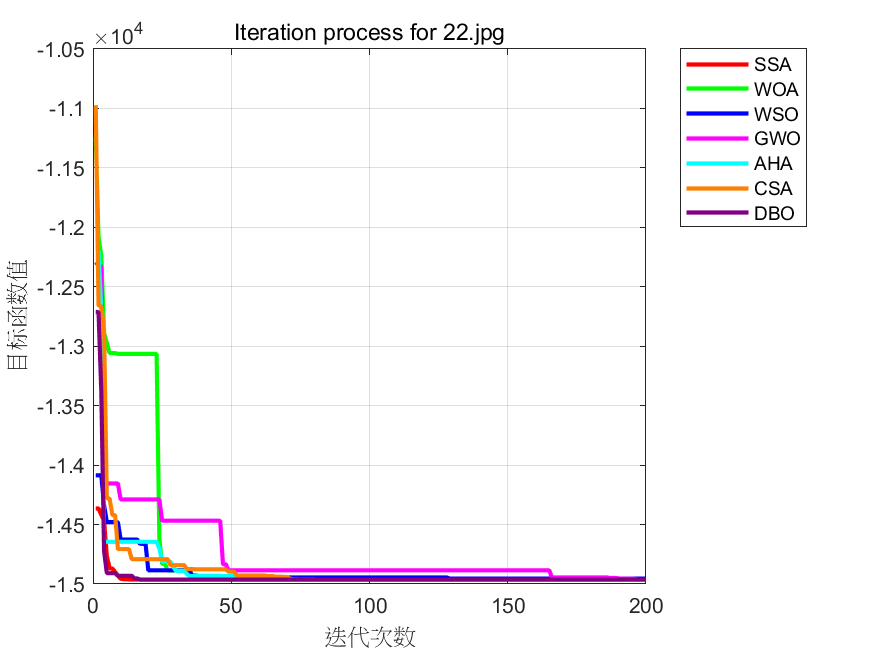

Supplement: S10 Data — (ZIP) [file pone.0297284.s010.zip › Level 5 processed Sample/iteration/22.jpg_iteration.png]

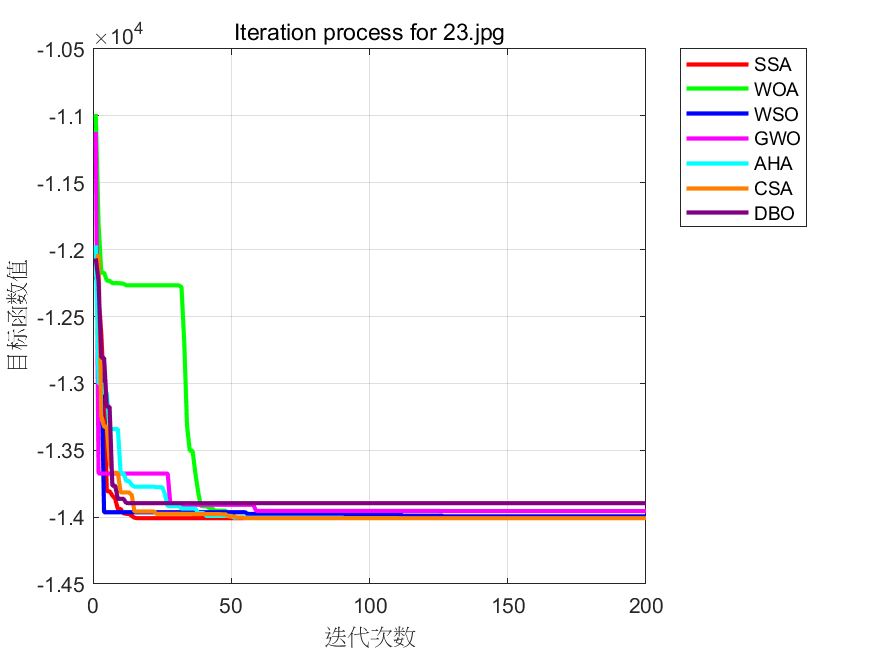

Supplement: S10 Data — (ZIP) [file pone.0297284.s010.zip › Level 5 processed Sample/iteration/23.jpg_iteration.png]

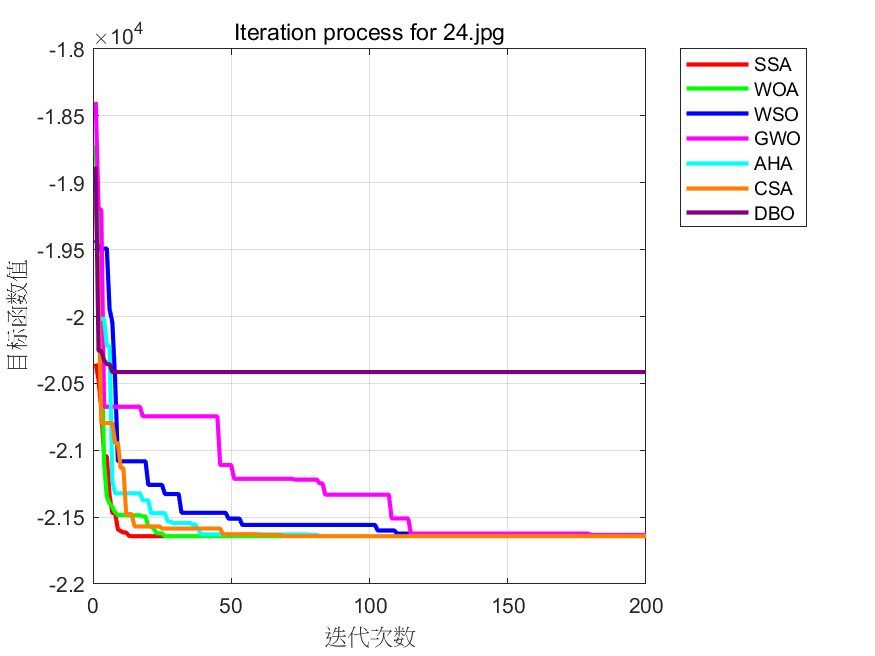

Supplement: S10 Data — (ZIP) [file pone.0297284.s010.zip › Level 5 processed Sample/iteration/24.jpg_iteration.png]

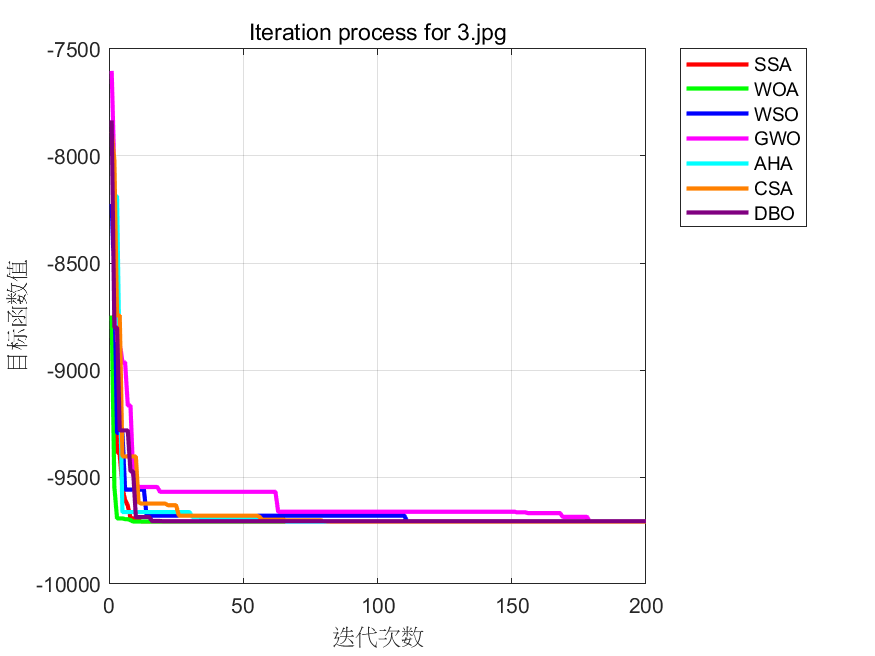

Supplement: S10 Data — (ZIP) [file pone.0297284.s010.zip › Level 5 processed Sample/iteration/3.jpg_iteration.png]

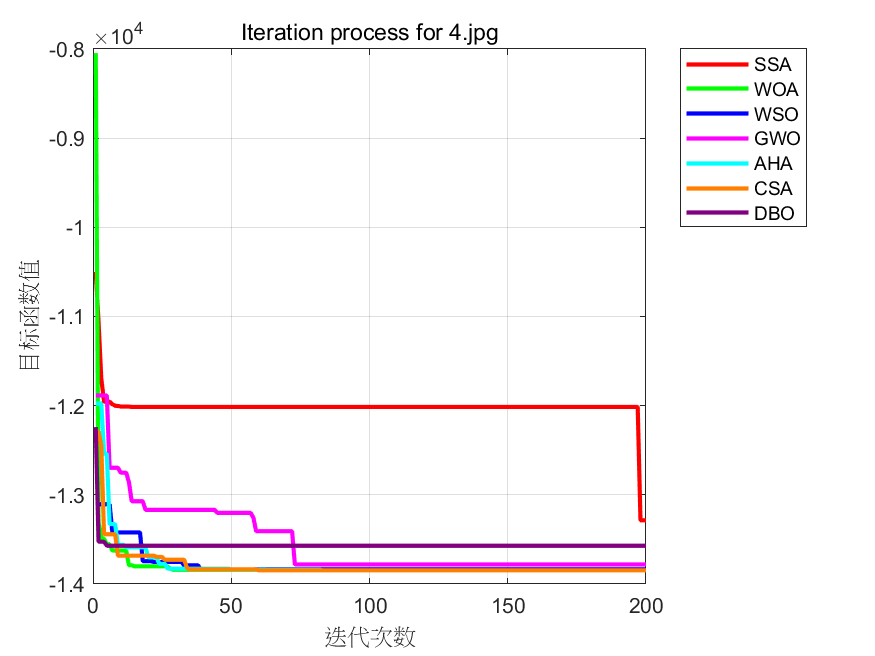

Supplement: S10 Data — (ZIP) [file pone.0297284.s010.zip › Level 5 processed Sample/iteration/4.jpg_iteration.png]

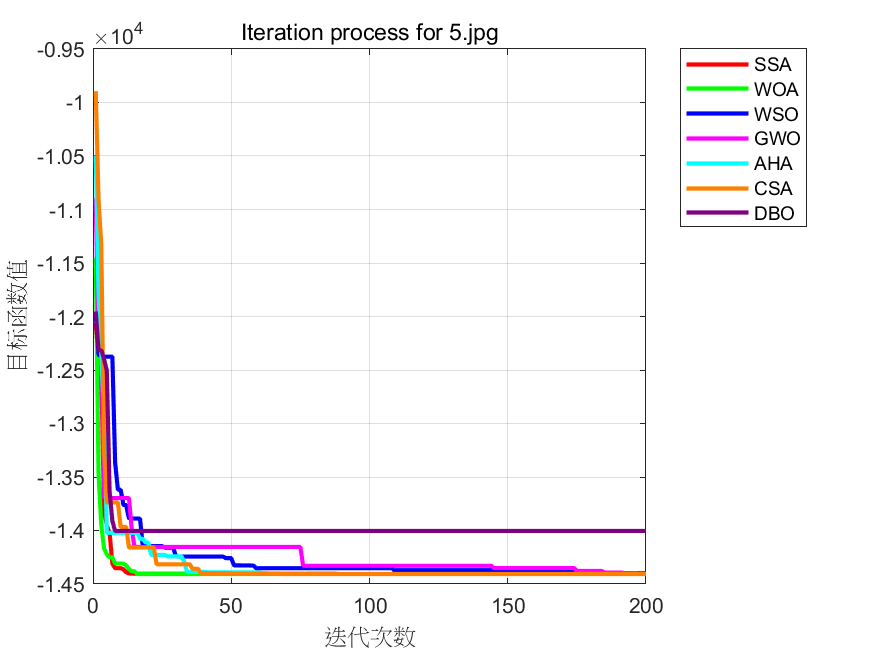

Supplement: S10 Data — (ZIP) [file pone.0297284.s010.zip › Level 5 processed Sample/iteration/5.jpg_iteration.png]

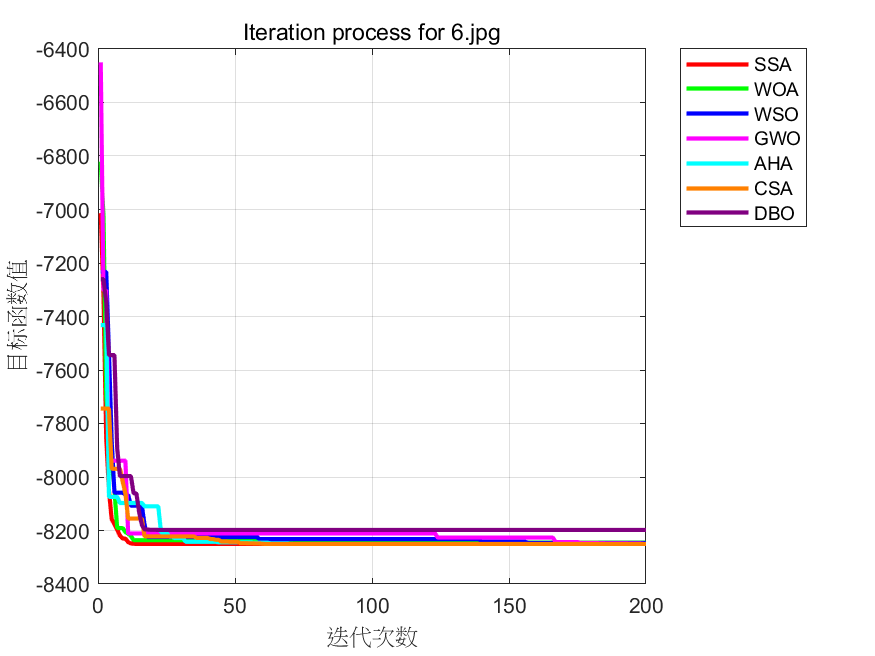

Supplement: S10 Data — (ZIP) [file pone.0297284.s010.zip › Level 5 processed Sample/iteration/6.jpg_iteration.png]

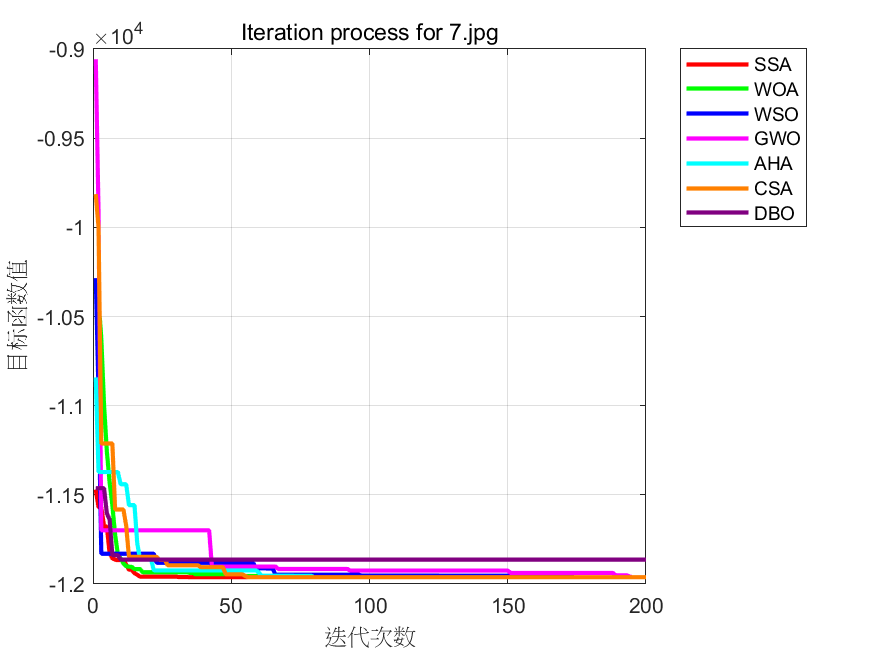

Supplement: S10 Data — (ZIP) [file pone.0297284.s010.zip › Level 5 processed Sample/iteration/7.jpg_iteration.png]

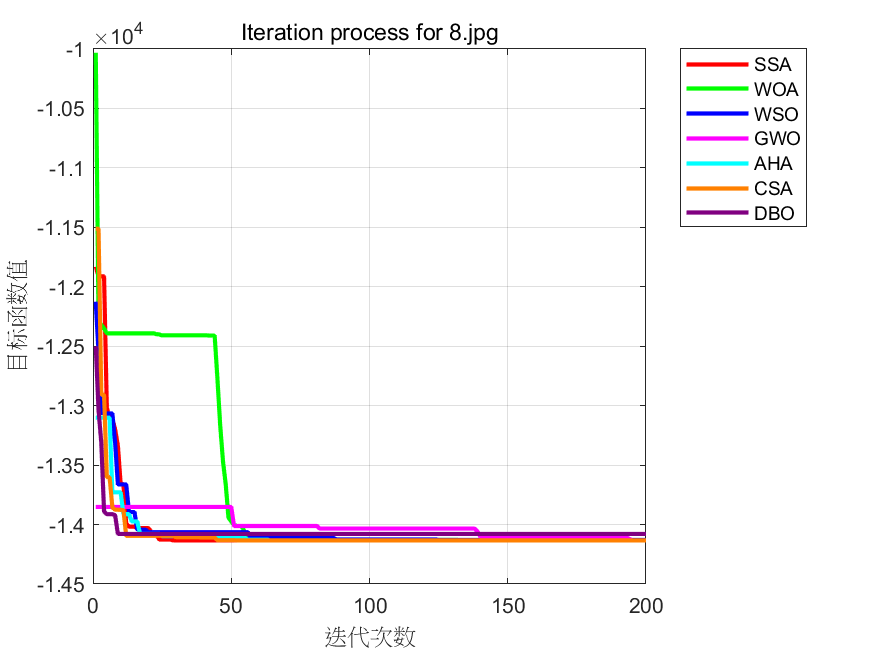

Supplement: S10 Data — (ZIP) [file pone.0297284.s010.zip › Level 5 processed Sample/iteration/8.jpg_iteration.png]

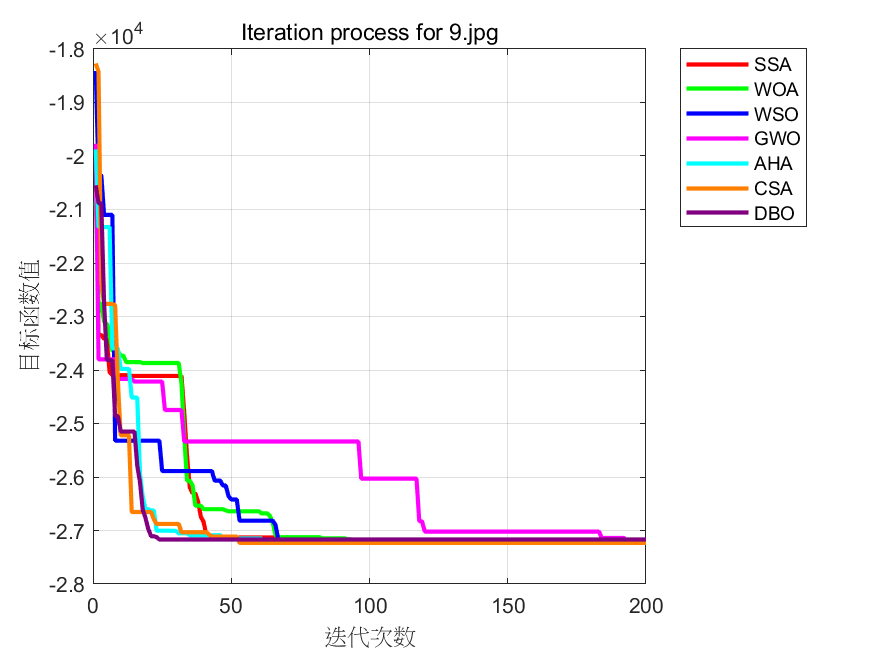

Supplement: S10 Data — (ZIP) [file pone.0297284.s010.zip › Level 5 processed Sample/iteration/9.jpg_iteration.png]

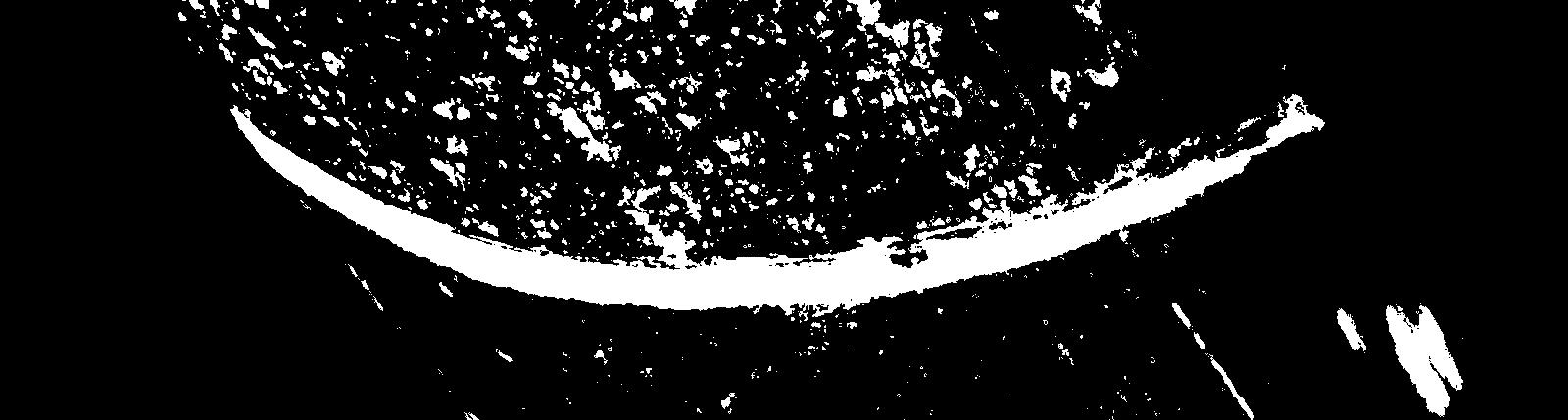

Supplement: S10 Data — (ZIP) [file pone.0297284.s010.zip › Level 5 processed Sample/processed_12/latex/AHA_latex.jpg]

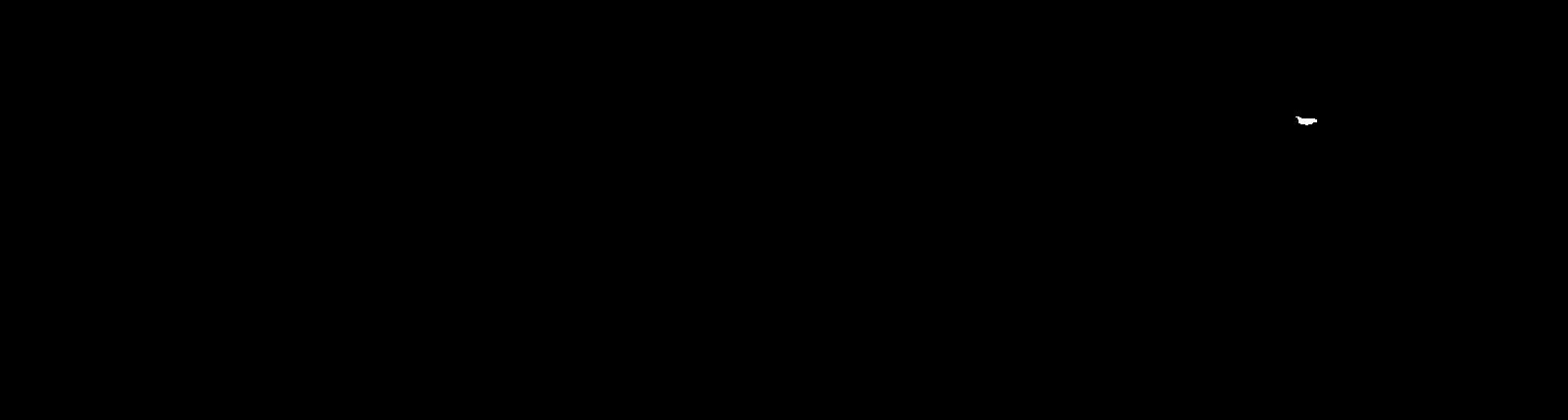

Supplement: S10 Data — (ZIP) [file pone.0297284.s010.zip › Level 5 processed Sample/processed_12/latex/DBO_latex.jpg]

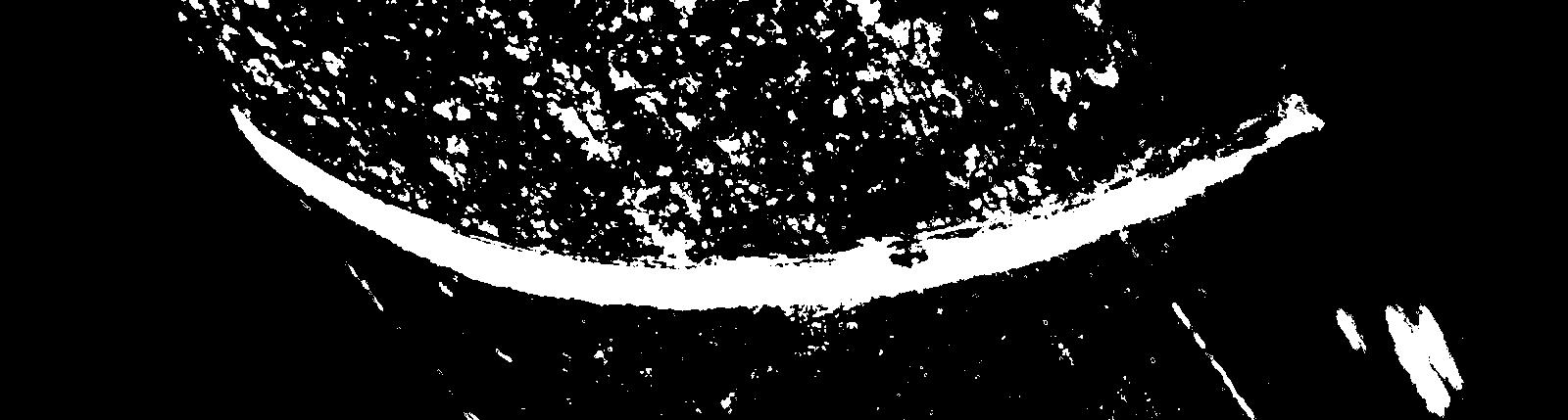

Supplement: S10 Data — (ZIP) [file pone.0297284.s010.zip › Level 5 processed Sample/processed_12/latex/GWO_latex.jpg]

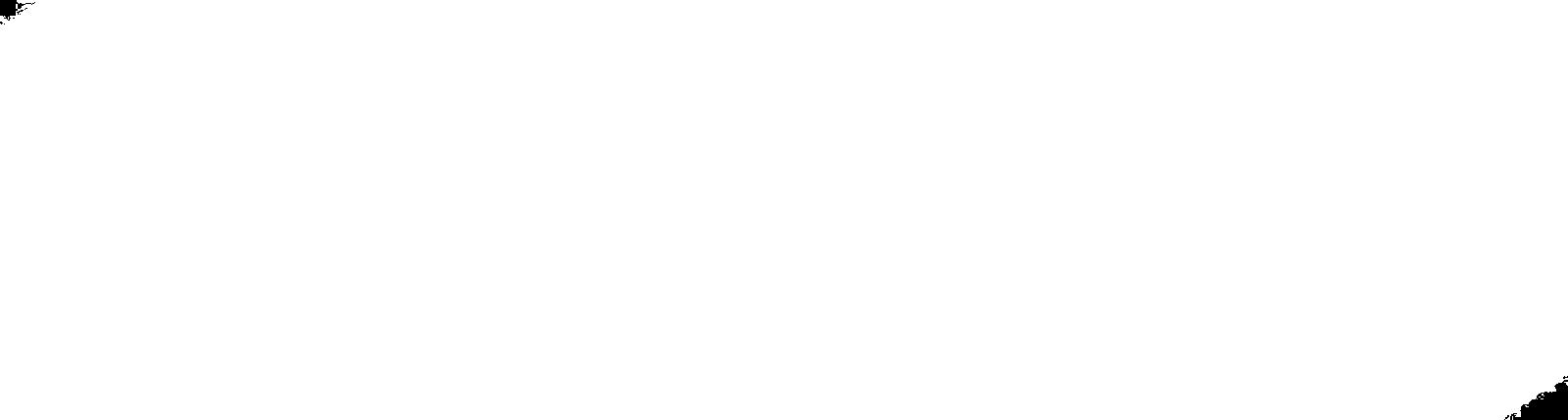

Supplement: S10 Data — (ZIP) [file pone.0297284.s010.zip › Level 5 processed Sample/processed_12/latex/OTSU_latex.jpg]

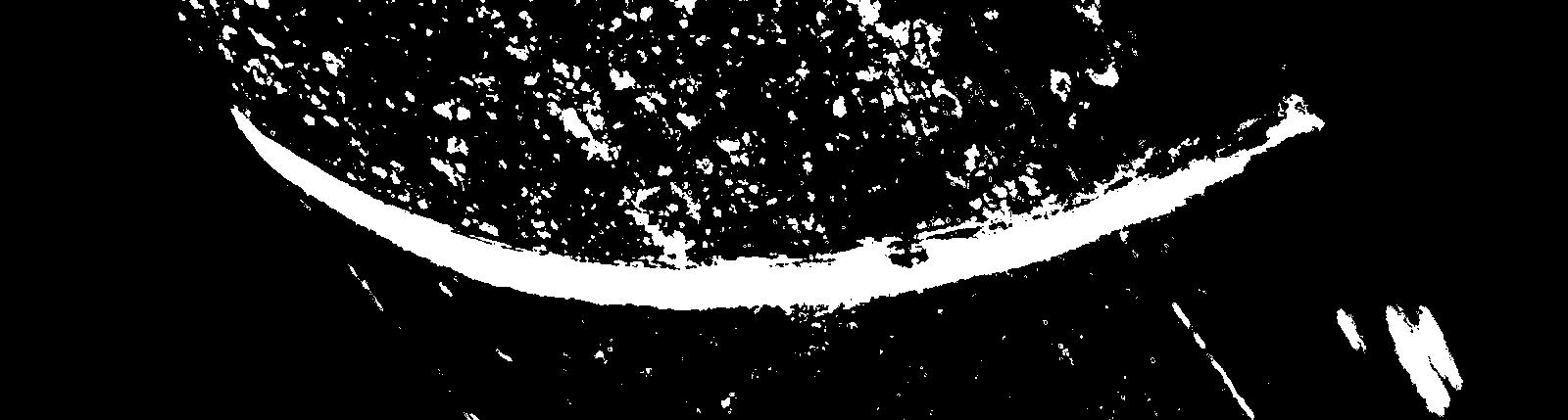

Supplement: S10 Data — (ZIP) [file pone.0297284.s010.zip › Level 5 processed Sample/processed_12/latex/WOA_latex.jpg]

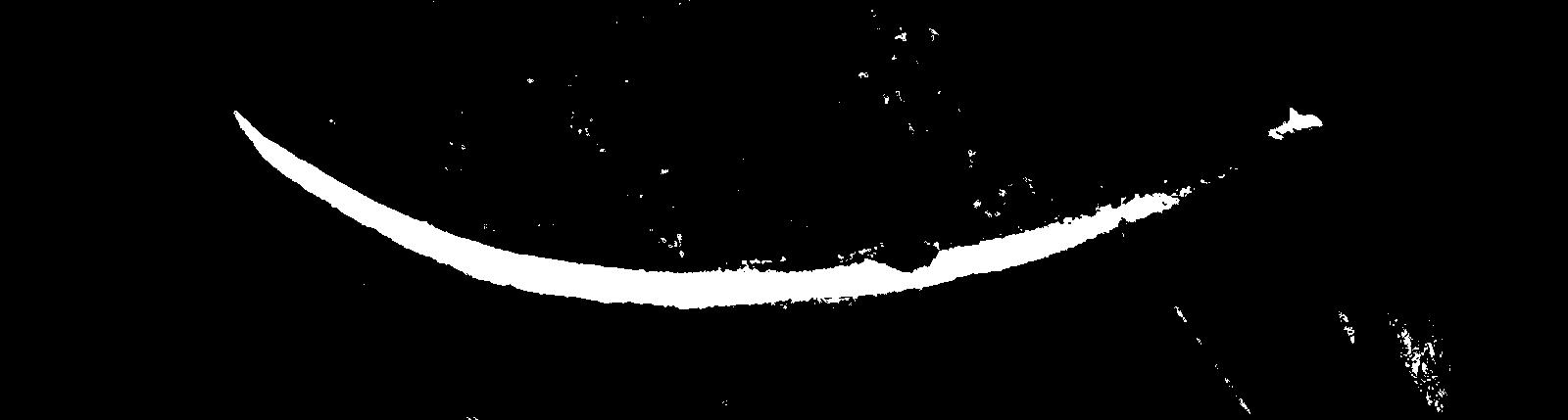

Supplement: S10 Data — (ZIP) [file pone.0297284.s010.zip › Level 5 processed Sample/processed_12/latex/WSO_latex.jpg]

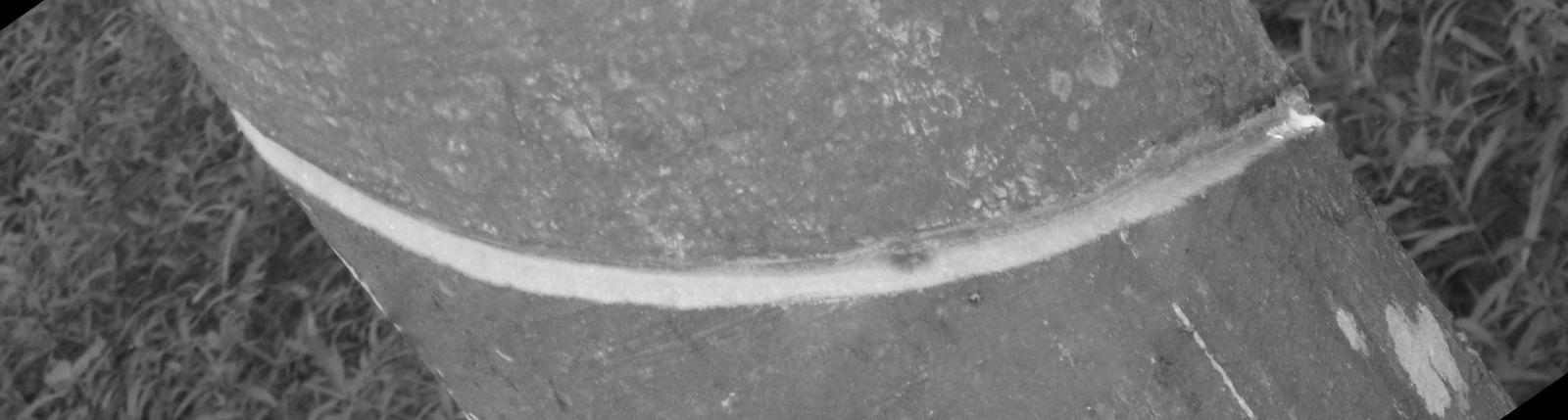

Supplement: S10 Data — (ZIP) [file pone.0297284.s010.zip › Level 5 processed Sample/processed_12/original_image.jpg]

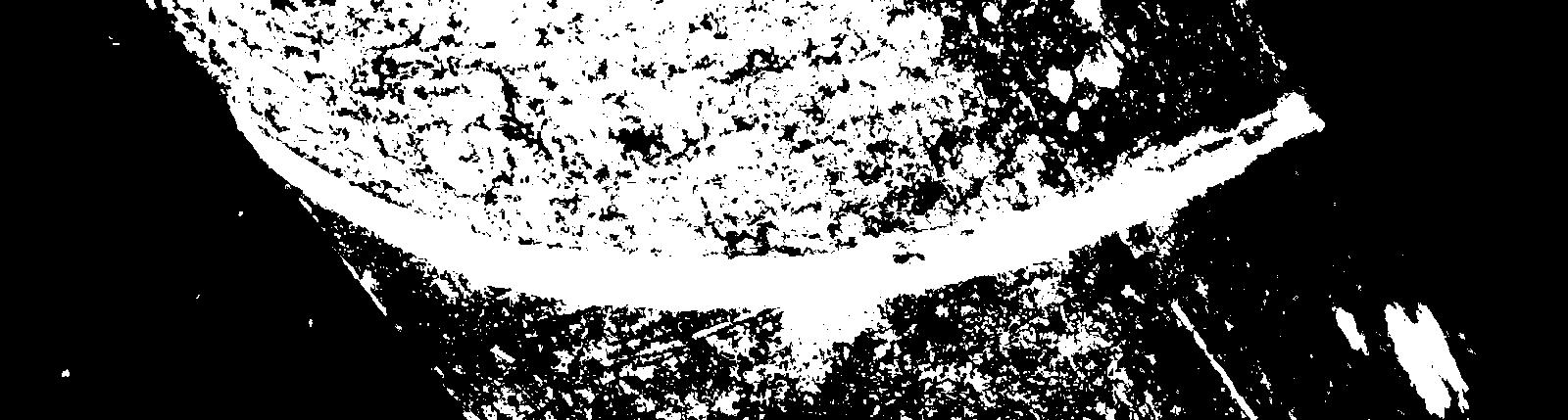

Supplement: S10 Data — (ZIP) [file pone.0297284.s010.zip › Level 5 processed Sample/processed_12/scar/AHA_scar.jpg]

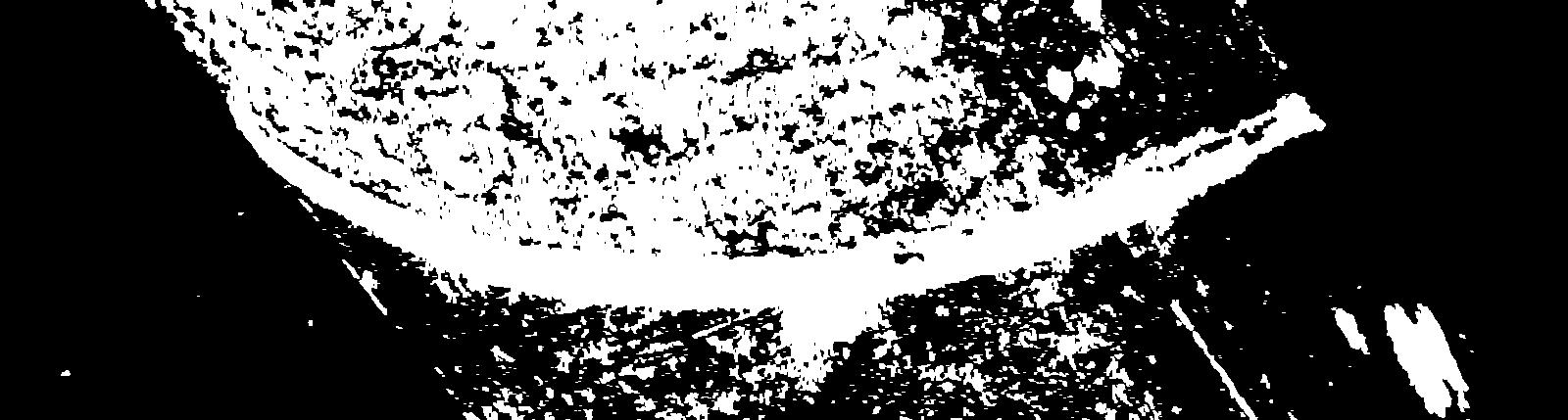

Supplement: S10 Data — (ZIP) [file pone.0297284.s010.zip › Level 5 processed Sample/processed_12/scar/DBO_scar.jpg]

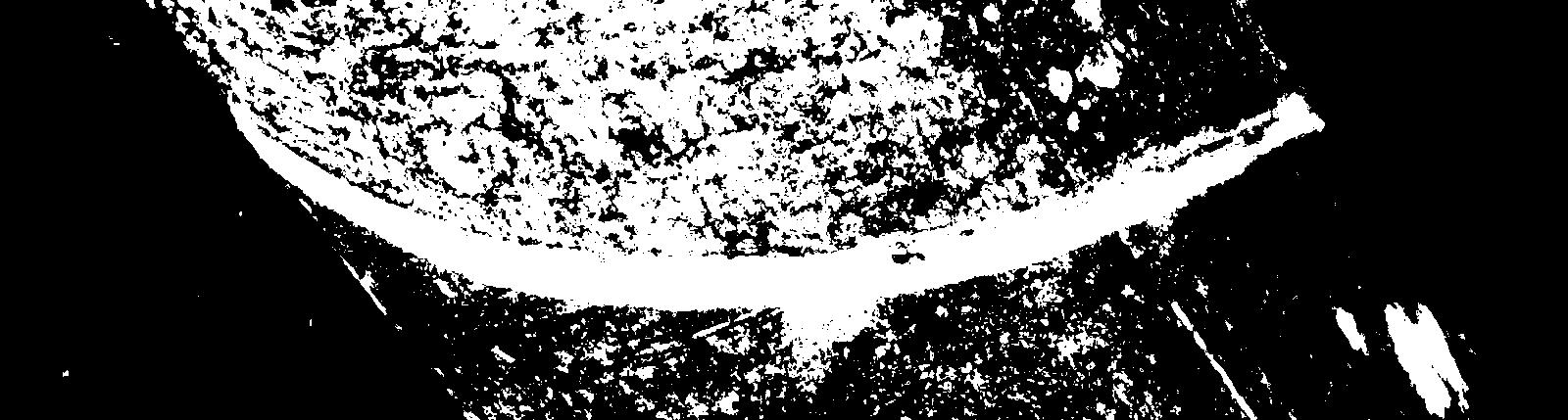

Supplement: S10 Data — (ZIP) [file pone.0297284.s010.zip › Level 5 processed Sample/processed_12/scar/WSO_scar.jpg]

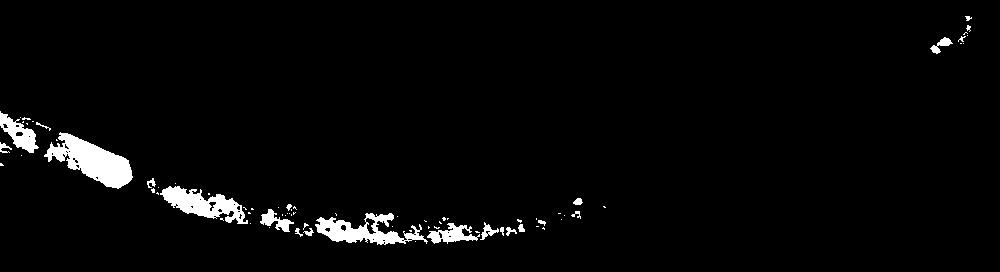

Supplement: S10 Data — (ZIP) [file pone.0297284.s010.zip › Level 5 processed Sample/processed_14/latex/AHA_latex.jpg]

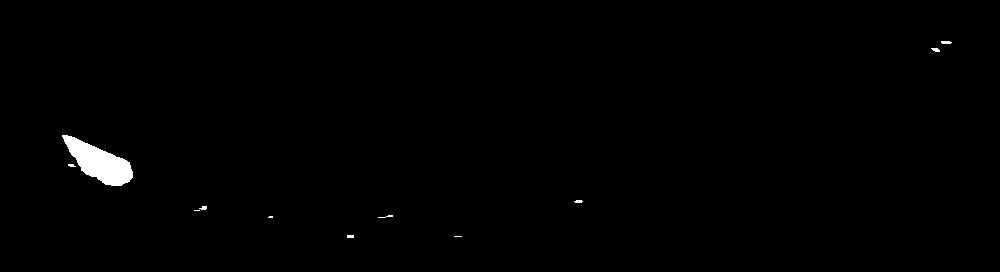

Supplement: S10 Data — (ZIP) [file pone.0297284.s010.zip › Level 5 processed Sample/processed_14/latex/DBO_latex.jpg]

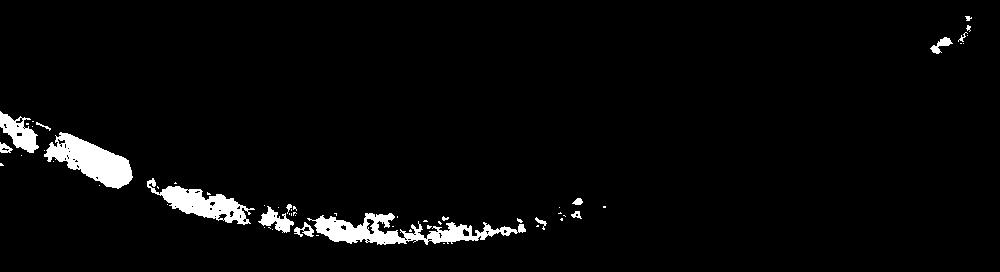

Supplement: S10 Data — (ZIP) [file pone.0297284.s010.zip › Level 5 processed Sample/processed_14/latex/GWO_latex.jpg]

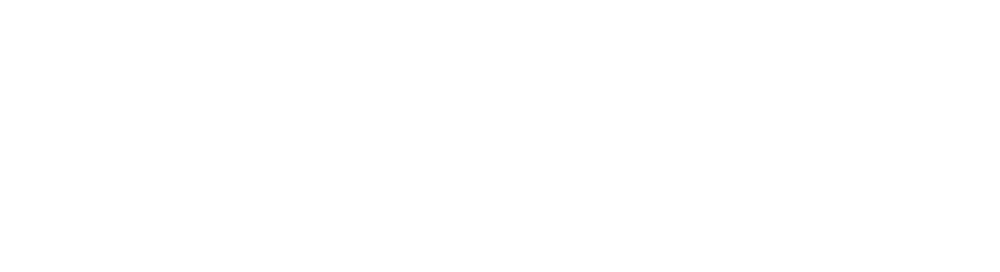

Supplement: S10 Data — (ZIP) [file pone.0297284.s010.zip › Level 5 processed Sample/processed_14/latex/OTSU_latex.jpg]

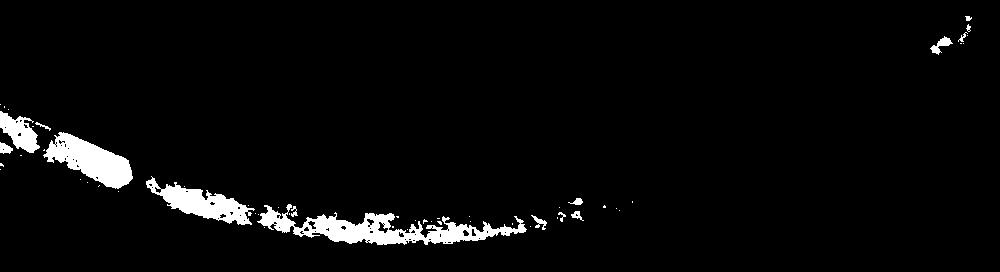

Supplement: S10 Data — (ZIP) [file pone.0297284.s010.zip › Level 5 processed Sample/processed_14/latex/WOA_latex.jpg]

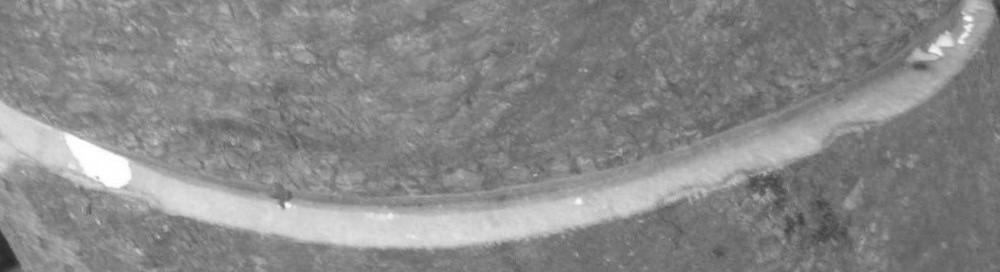

Supplement: S10 Data — (ZIP) [file pone.0297284.s010.zip › Level 5 processed Sample/processed_14/original_image.jpg]

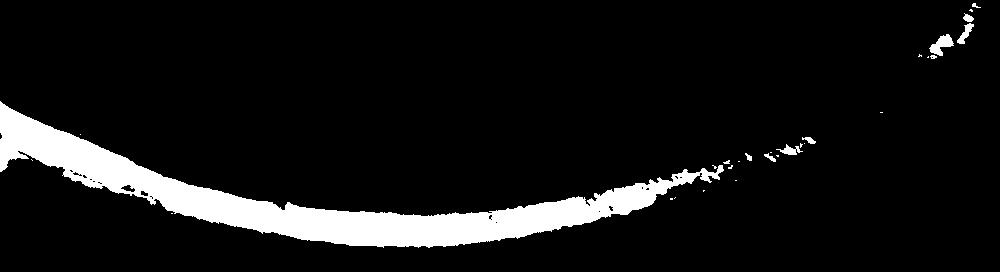

Supplement: S10 Data — (ZIP) [file pone.0297284.s010.zip › Level 5 processed Sample/processed_14/scar/AHA_scar.jpg]

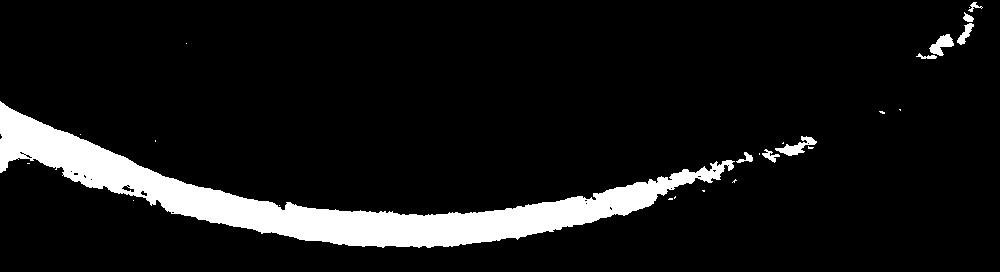

Supplement: S10 Data — (ZIP) [file pone.0297284.s010.zip › Level 5 processed Sample/processed_14/scar/CSA_scar.jpg]

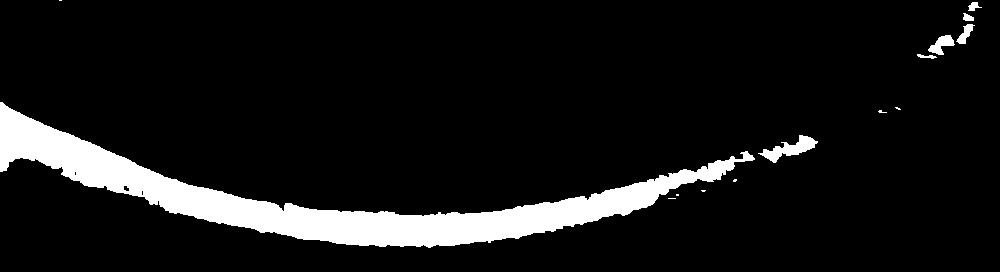

Supplement: S10 Data — (ZIP) [file pone.0297284.s010.zip › Level 5 processed Sample/processed_14/scar/DBO_scar.jpg]

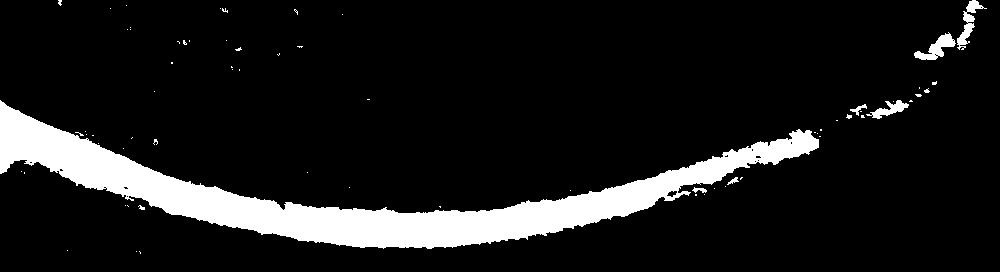

Supplement: S10 Data — (ZIP) [file pone.0297284.s010.zip › Level 5 processed Sample/processed_14/scar/WOA_scar.jpg]

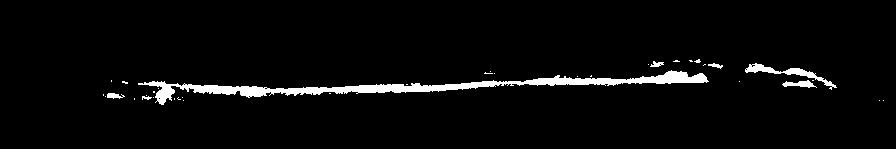

Supplement: S10 Data — (ZIP) [file pone.0297284.s010.zip › Level 5 processed Sample/processed_16/latex/AHA_latex.jpg]

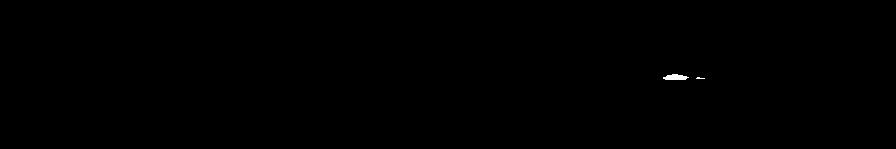

Supplement: S10 Data — (ZIP) [file pone.0297284.s010.zip › Level 5 processed Sample/processed_16/latex/DBO_latex.jpg]

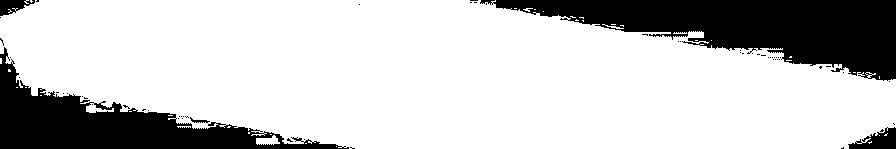

Supplement: S10 Data — (ZIP) [file pone.0297284.s010.zip › Level 5 processed Sample/processed_16/latex/OTSU_latex.jpg]

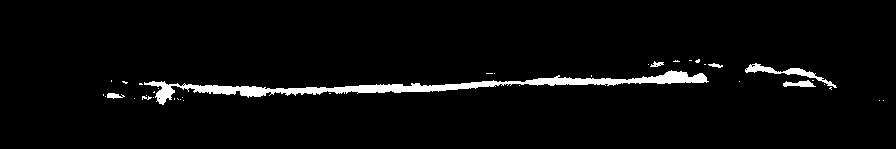

Supplement: S10 Data — (ZIP) [file pone.0297284.s010.zip › Level 5 processed Sample/processed_16/latex/WOA_latex.jpg]

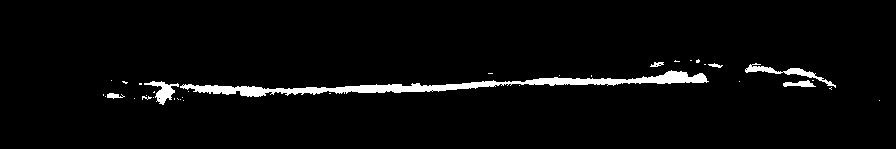

Supplement: S10 Data — (ZIP) [file pone.0297284.s010.zip › Level 5 processed Sample/processed_16/latex/WSO_latex.jpg]

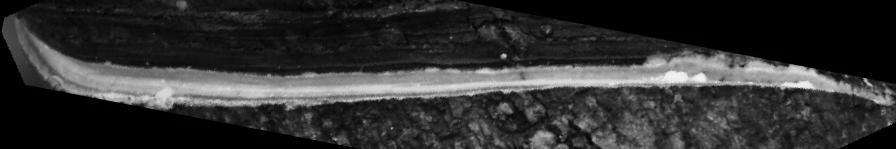

Supplement: S10 Data — (ZIP) [file pone.0297284.s010.zip › Level 5 processed Sample/processed_16/original_image.jpg]

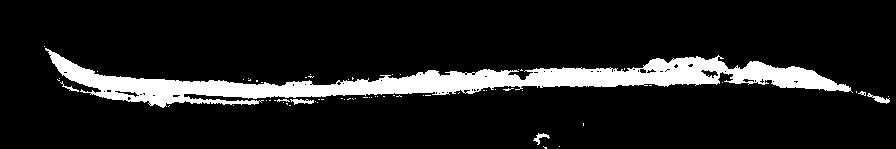

Supplement: S10 Data — (ZIP) [file pone.0297284.s010.zip › Level 5 processed Sample/processed_16/scar/AHA_scar.jpg]

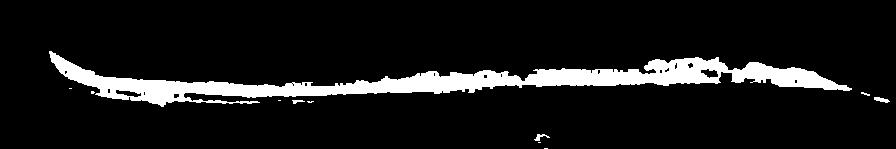

Supplement: S10 Data — (ZIP) [file pone.0297284.s010.zip › Level 5 processed Sample/processed_16/scar/DBO_scar.jpg]

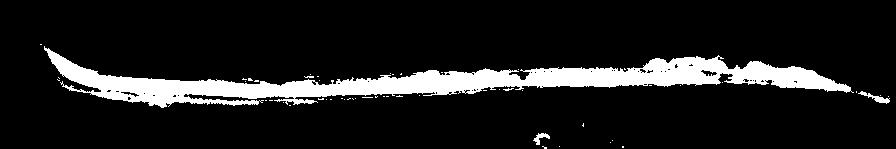

Supplement: S10 Data — (ZIP) [file pone.0297284.s010.zip › Level 5 processed Sample/processed_16/scar/WSO_scar.jpg]

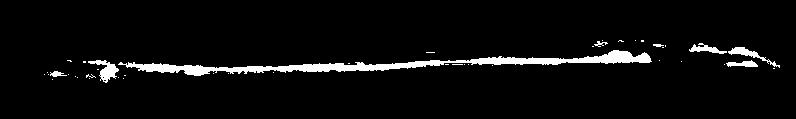

Supplement: S10 Data — (ZIP) [file pone.0297284.s010.zip › Level 5 processed Sample/processed_17/latex/AHA_latex.jpg]

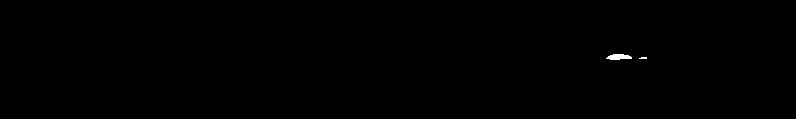

Supplement: S10 Data — (ZIP) [file pone.0297284.s010.zip › Level 5 processed Sample/processed_17/latex/DBO_latex.jpg]

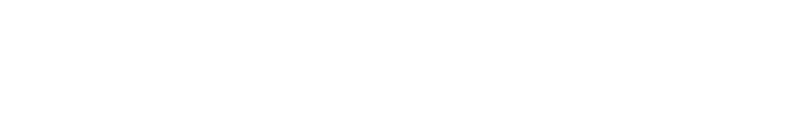

Supplement: S10 Data — (ZIP) [file pone.0297284.s010.zip › Level 5 processed Sample/processed_17/latex/OTSU_latex.jpg]

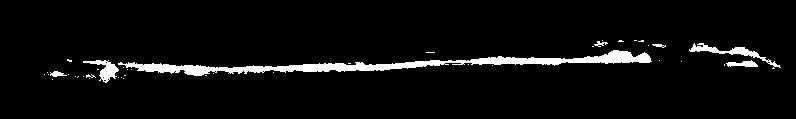

Supplement: S10 Data — (ZIP) [file pone.0297284.s010.zip › Level 5 processed Sample/processed_17/latex/WOA_latex.jpg]

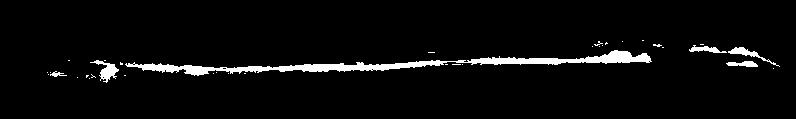

Supplement: S10 Data — (ZIP) [file pone.0297284.s010.zip › Level 5 processed Sample/processed_17/latex/WSO_latex.jpg]

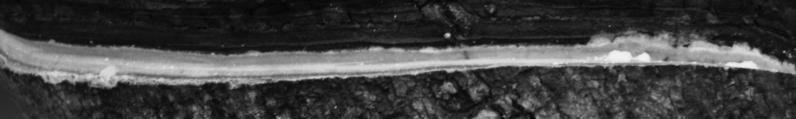

Supplement: S10 Data — (ZIP) [file pone.0297284.s010.zip › Level 5 processed Sample/processed_17/original_image.jpg]

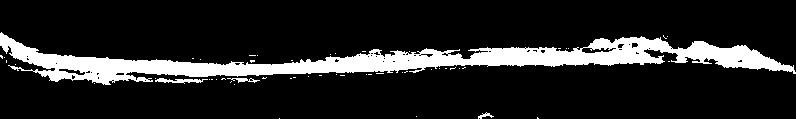

Supplement: S10 Data — (ZIP) [file pone.0297284.s010.zip › Level 5 processed Sample/processed_17/scar/AHA_scar.jpg]

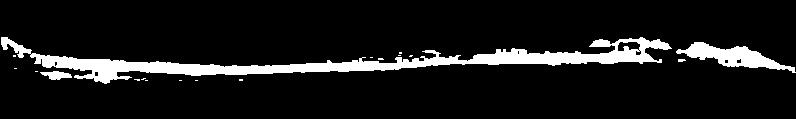

Supplement: S10 Data — (ZIP) [file pone.0297284.s010.zip › Level 5 processed Sample/processed_17/scar/DBO_scar.jpg]
