# Supplementary figures and images for: Advancing image segmentation with DBO-Otsu: Addressing rubber tree diseases through enhanced threshold techniques (part 7 of 7)
Source: PLoS One. 2024 Mar 21;19(3):e0297284. doi: 10.1371/journal.pone.0297284 (PMC10956860; doi:10.1371/journal.pone.0297284)

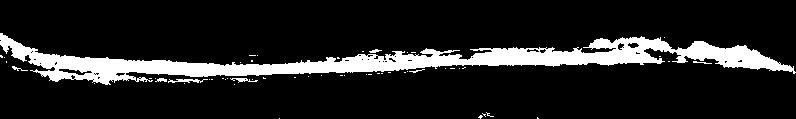

Supplement: S10 Data — (ZIP) [file pone.0297284.s010.zip › Level 5 processed Sample/processed_17/scar/WSO_scar.jpg]

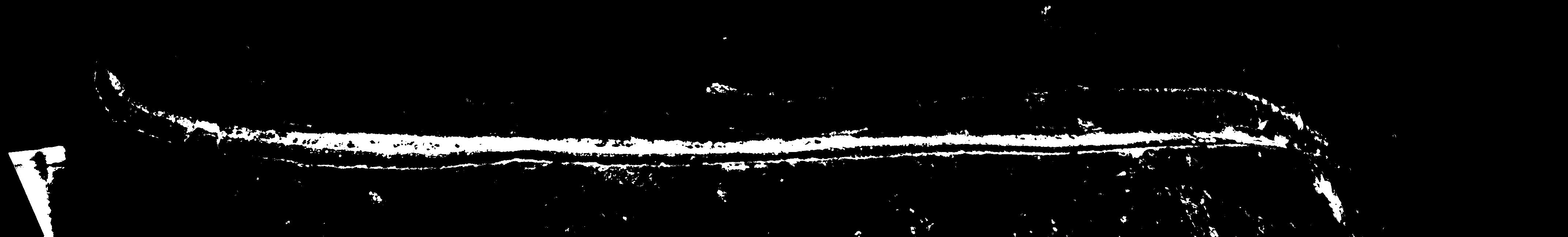

Supplement: S10 Data — (ZIP) [file pone.0297284.s010.zip › Level 5 processed Sample/processed_18/latex/AHA_latex.jpg]

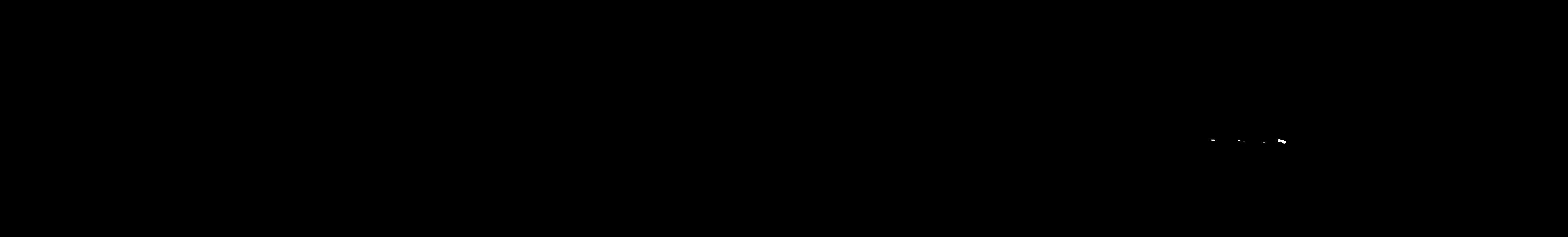

Supplement: S10 Data — (ZIP) [file pone.0297284.s010.zip › Level 5 processed Sample/processed_18/latex/DBO_latex.jpg]

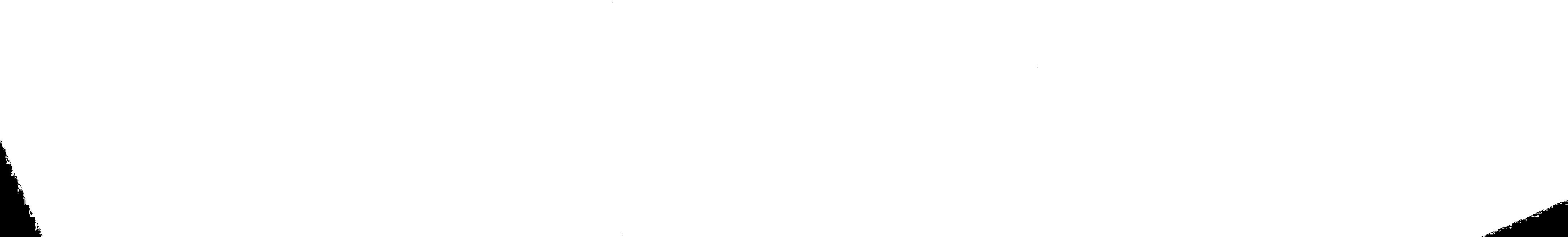

Supplement: S10 Data — (ZIP) [file pone.0297284.s010.zip › Level 5 processed Sample/processed_18/latex/OTSU_latex.jpg]

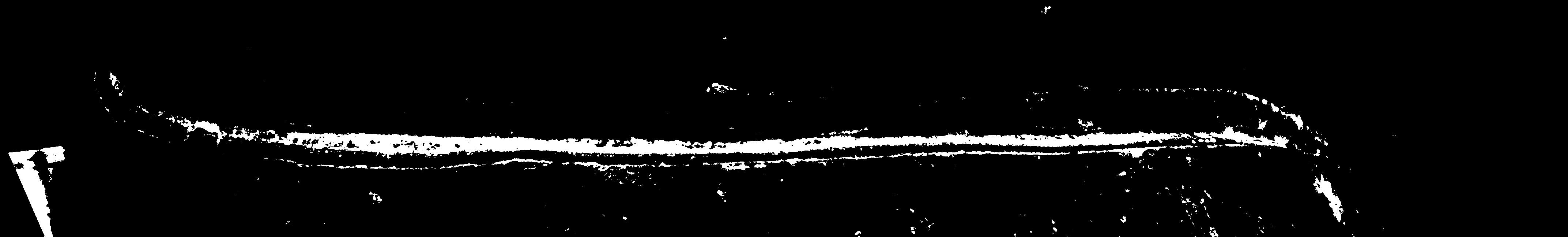

Supplement: S10 Data — (ZIP) [file pone.0297284.s010.zip › Level 5 processed Sample/processed_18/latex/WOA_latex.jpg]

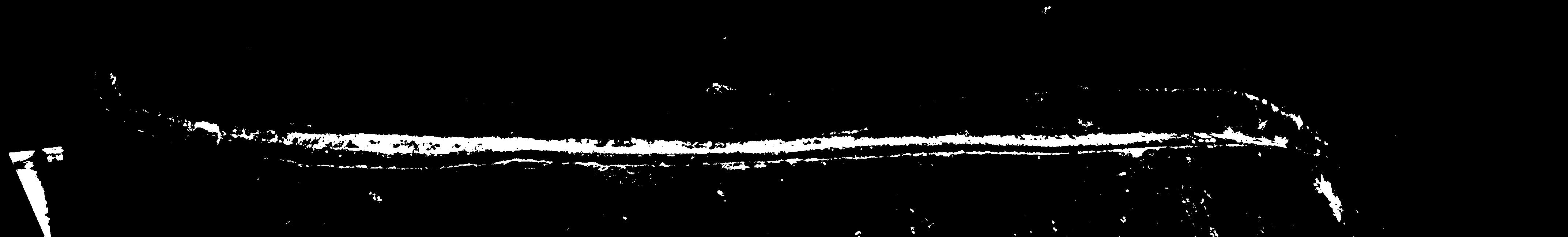

Supplement: S10 Data — (ZIP) [file pone.0297284.s010.zip › Level 5 processed Sample/processed_18/latex/WSO_latex.jpg]

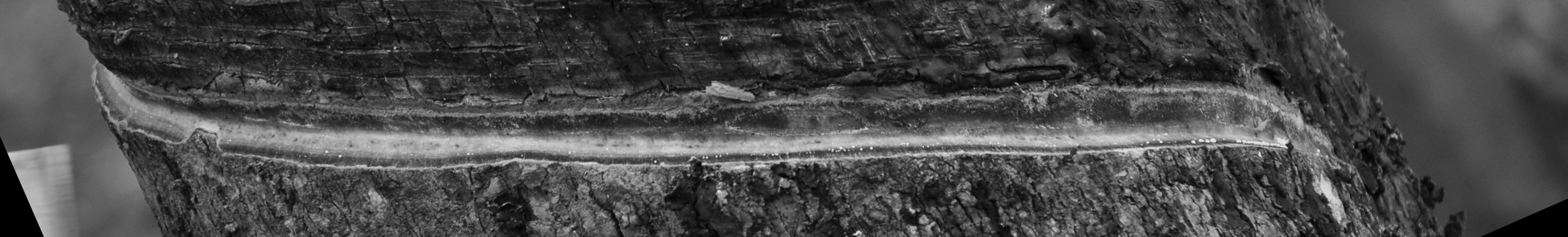

Supplement: S10 Data — (ZIP) [file pone.0297284.s010.zip › Level 5 processed Sample/processed_18/original_image.jpg]

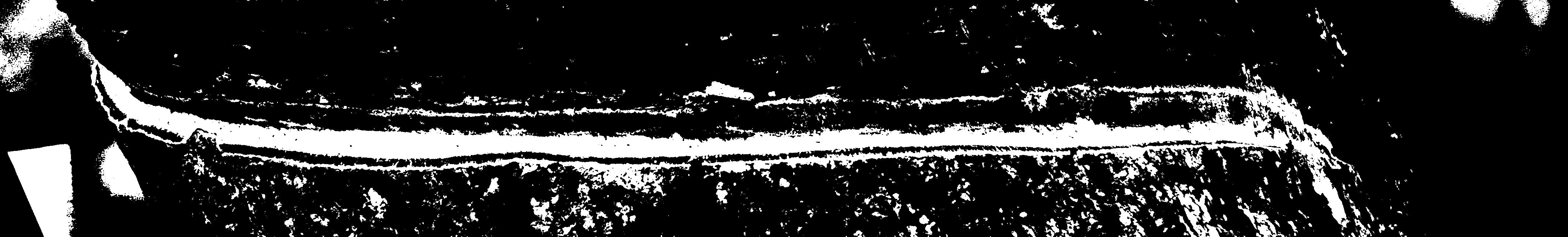

Supplement: S10 Data — (ZIP) [file pone.0297284.s010.zip › Level 5 processed Sample/processed_18/scar/AHA_scar.jpg]

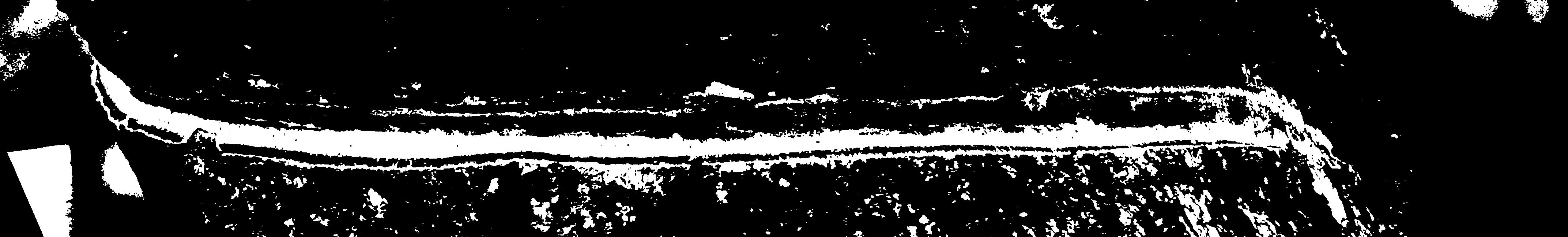

Supplement: S10 Data — (ZIP) [file pone.0297284.s010.zip › Level 5 processed Sample/processed_18/scar/DBO_scar.jpg]

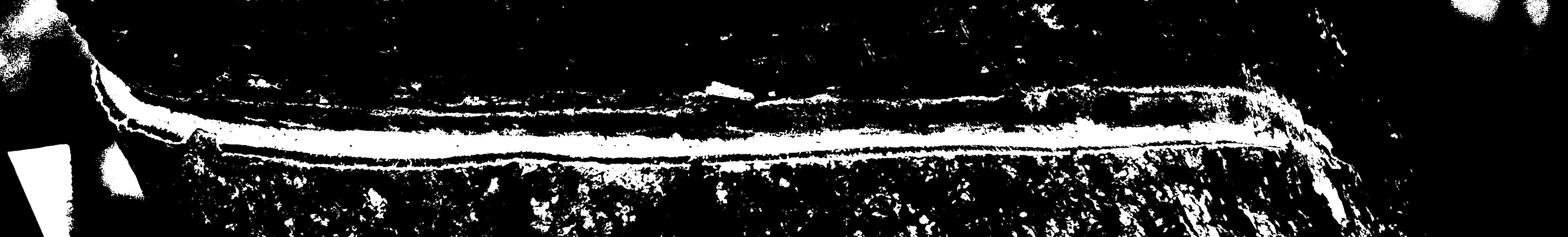

Supplement: S10 Data — (ZIP) [file pone.0297284.s010.zip › Level 5 processed Sample/processed_18/scar/WOA_scar.jpg]

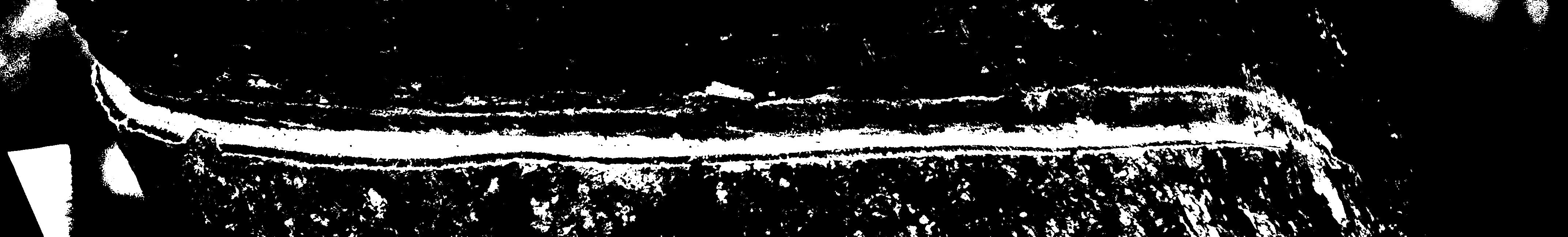

Supplement: S10 Data — (ZIP) [file pone.0297284.s010.zip › Level 5 processed Sample/processed_18/scar/WSO_scar.jpg]

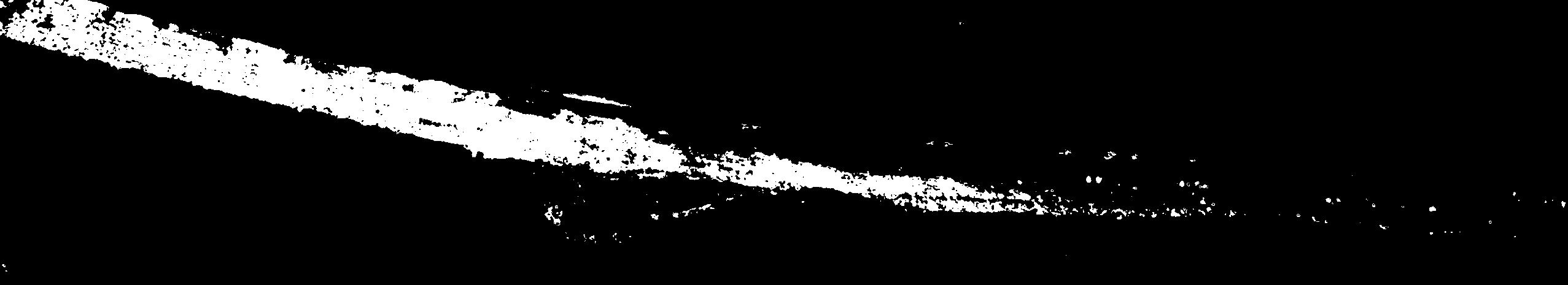

Supplement: S10 Data — (ZIP) [file pone.0297284.s010.zip › Level 5 processed Sample/processed_19/latex/AHA_latex.jpg]

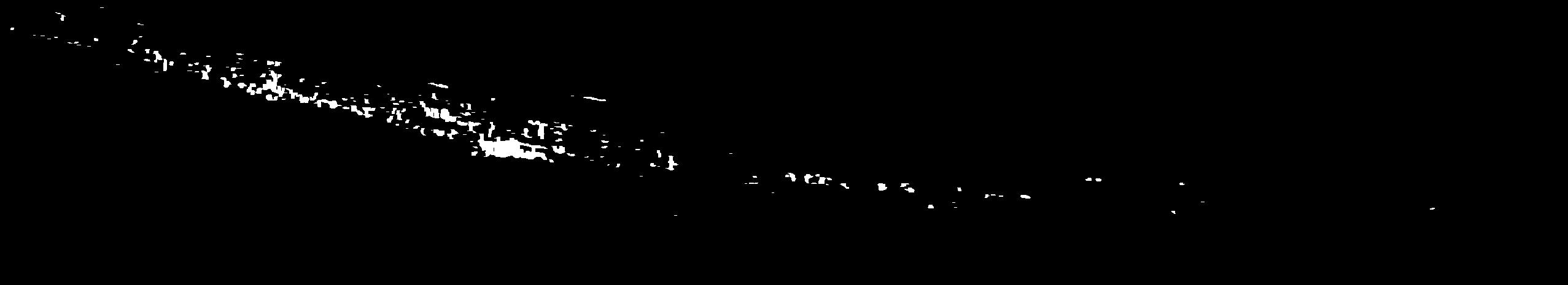

Supplement: S10 Data — (ZIP) [file pone.0297284.s010.zip › Level 5 processed Sample/processed_19/latex/DBO_latex.jpg]

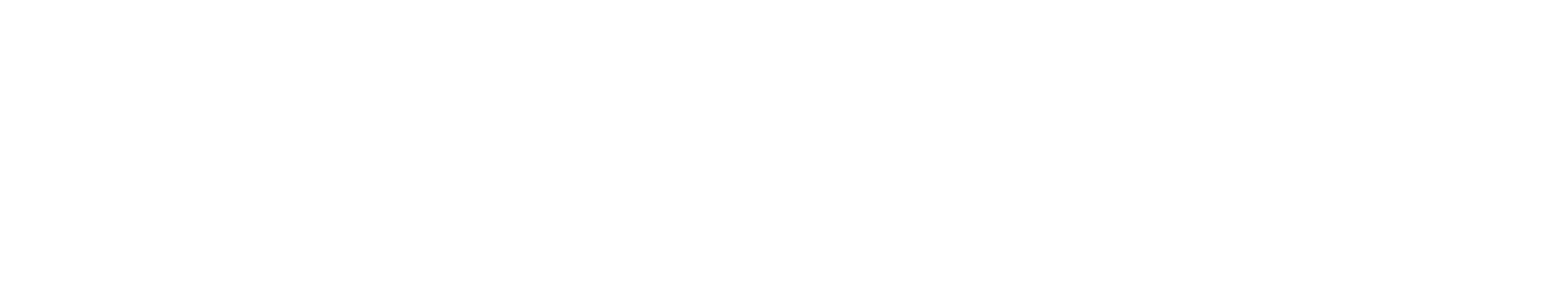

Supplement: S10 Data — (ZIP) [file pone.0297284.s010.zip › Level 5 processed Sample/processed_19/latex/OTSU_latex.jpg]

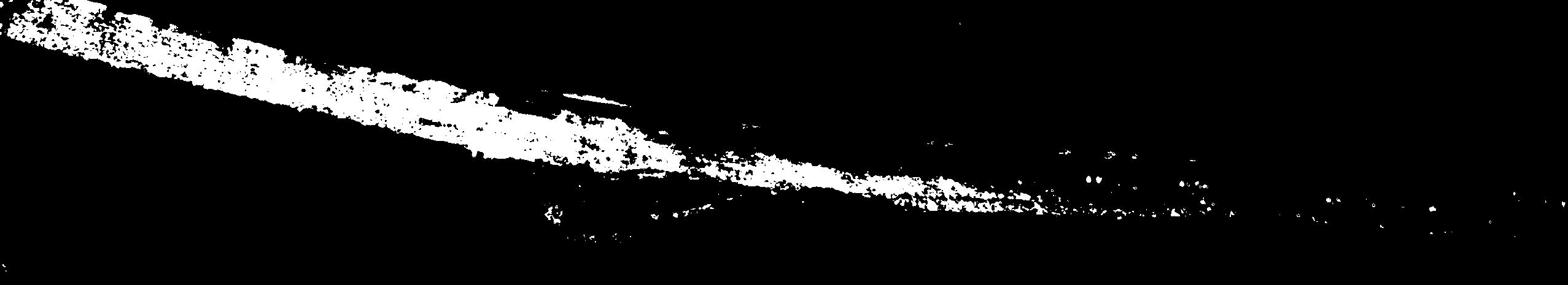

Supplement: S10 Data — (ZIP) [file pone.0297284.s010.zip › Level 5 processed Sample/processed_19/latex/WSO_latex.jpg]

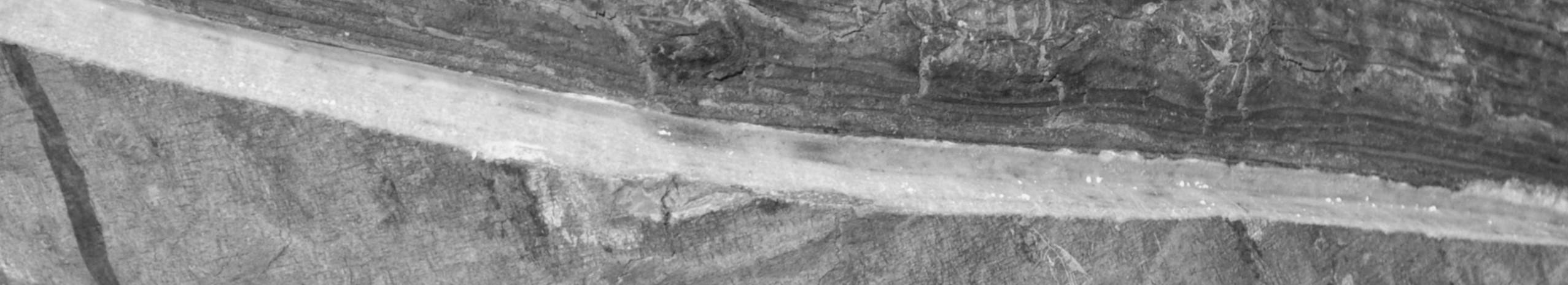

Supplement: S10 Data — (ZIP) [file pone.0297284.s010.zip › Level 5 processed Sample/processed_19/original_image.jpg]

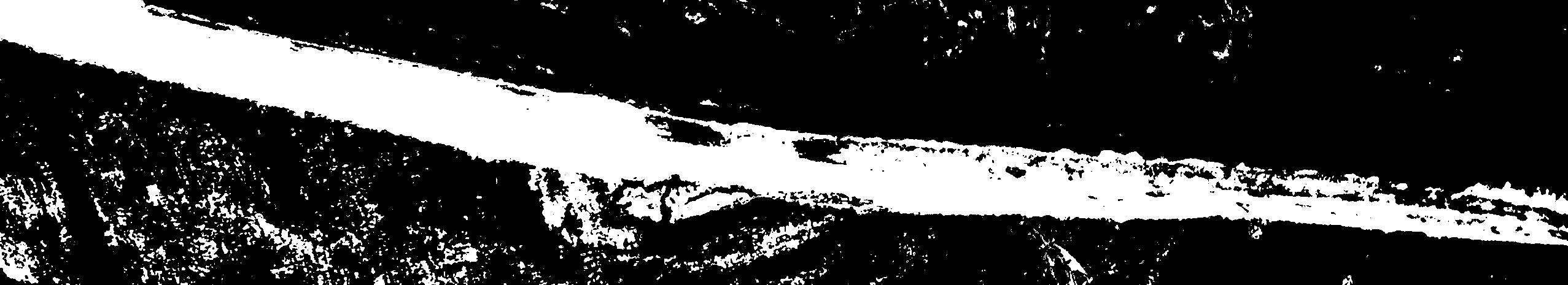

Supplement: S10 Data — (ZIP) [file pone.0297284.s010.zip › Level 5 processed Sample/processed_19/scar/AHA_scar.jpg]

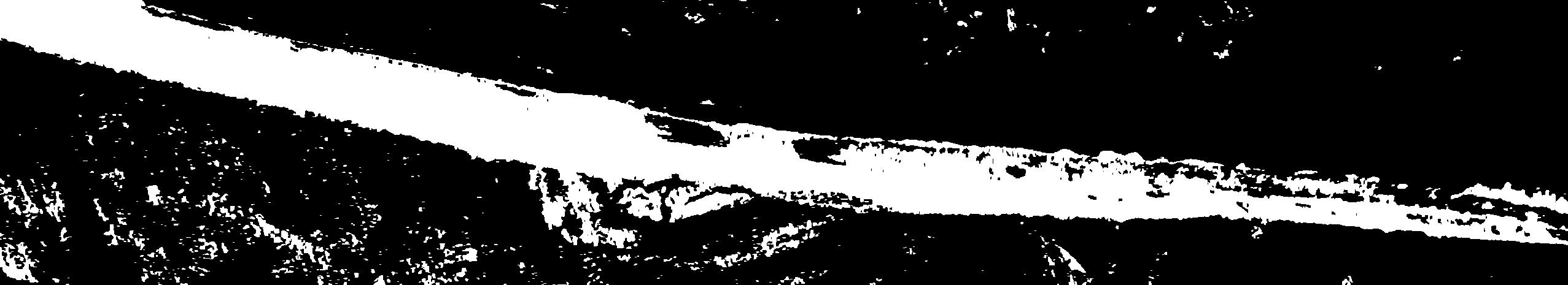

Supplement: S10 Data — (ZIP) [file pone.0297284.s010.zip › Level 5 processed Sample/processed_19/scar/DBO_scar.jpg]

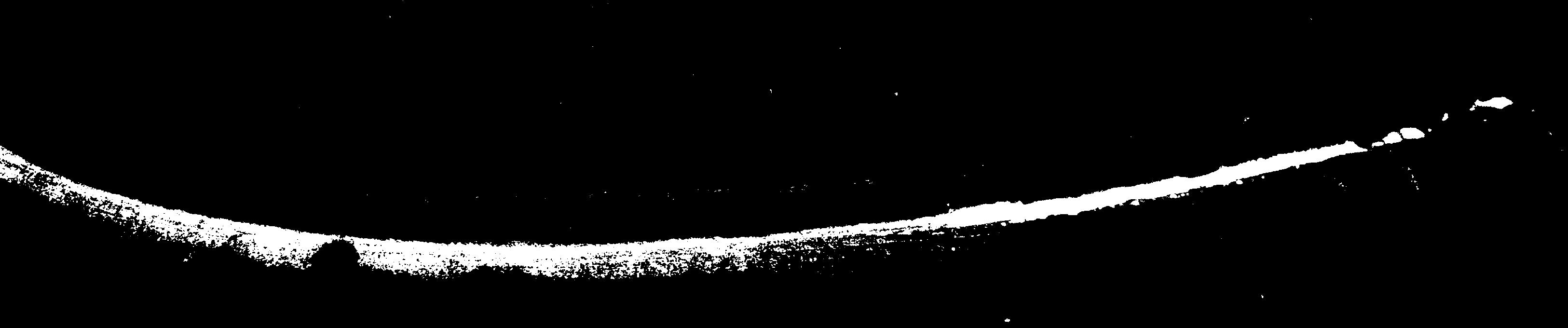

Supplement: S10 Data — (ZIP) [file pone.0297284.s010.zip › Level 5 processed Sample/processed_21/latex/AHA_latex.jpg]

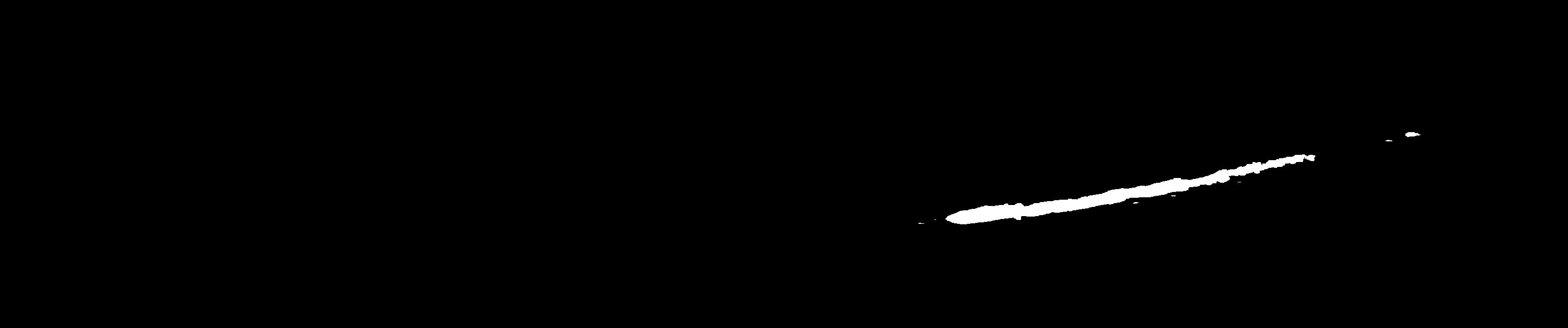

Supplement: S10 Data — (ZIP) [file pone.0297284.s010.zip › Level 5 processed Sample/processed_21/latex/DBO_latex.jpg]

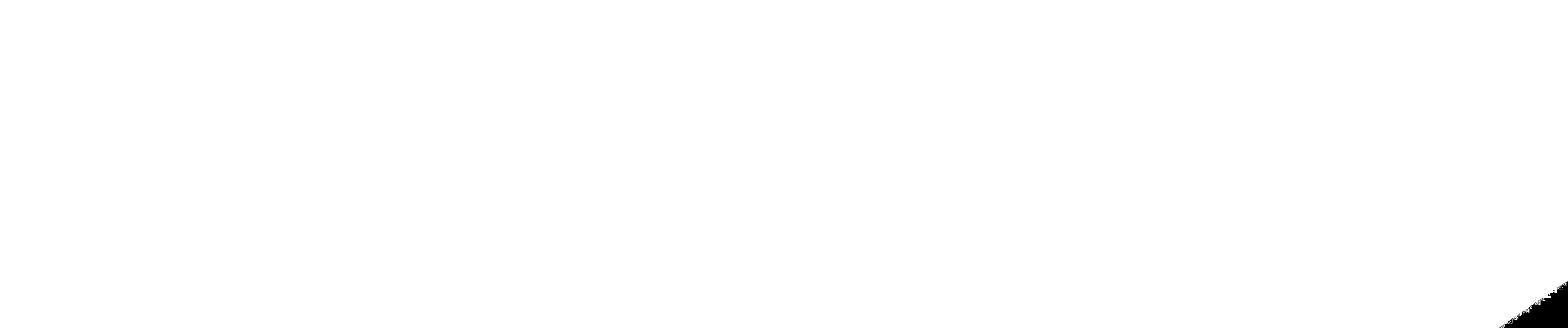

Supplement: S10 Data — (ZIP) [file pone.0297284.s010.zip › Level 5 processed Sample/processed_21/latex/OTSU_latex.jpg]

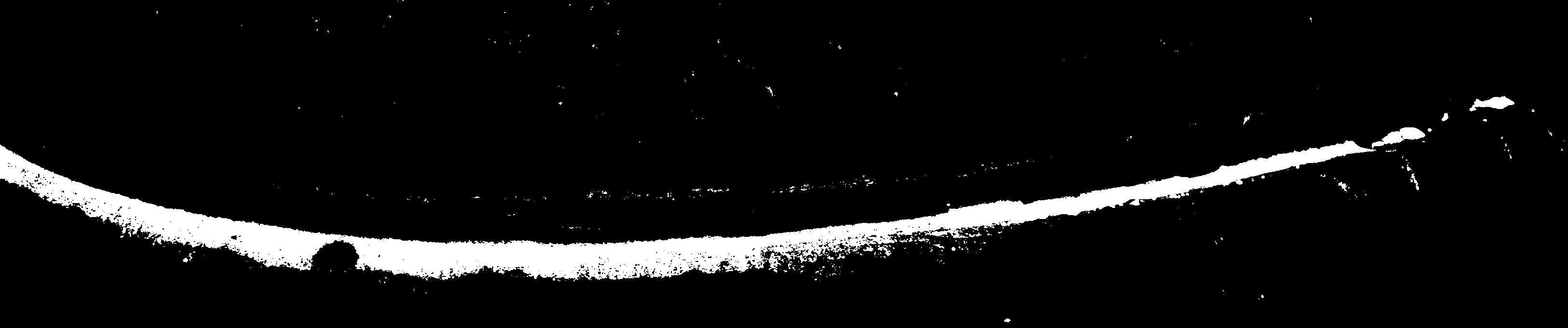

Supplement: S10 Data — (ZIP) [file pone.0297284.s010.zip › Level 5 processed Sample/processed_21/latex/WSO_latex.jpg]

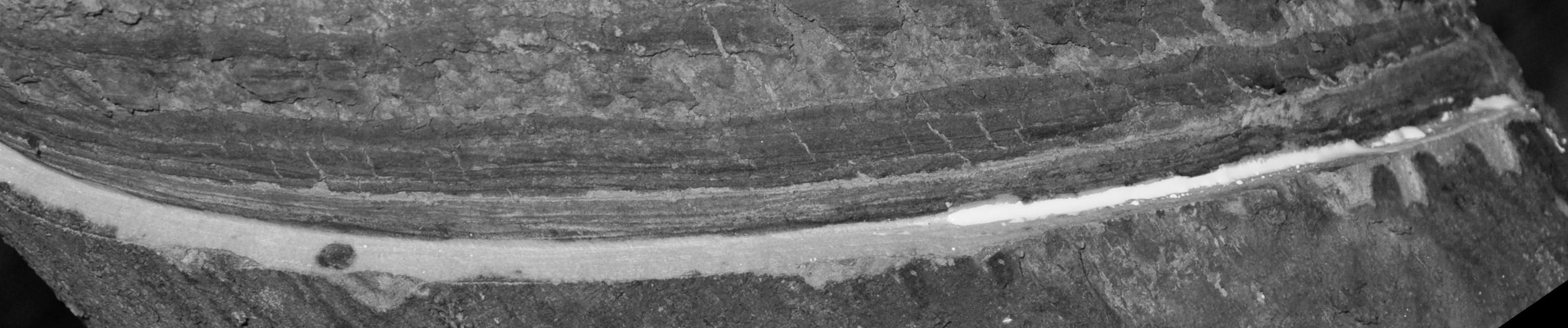

Supplement: S10 Data — (ZIP) [file pone.0297284.s010.zip › Level 5 processed Sample/processed_21/original_image.jpg]

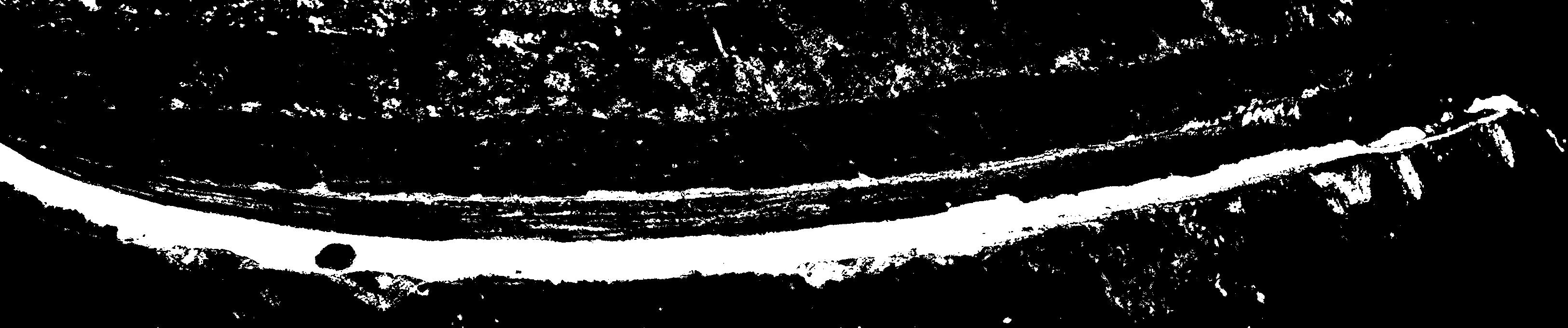

Supplement: S10 Data — (ZIP) [file pone.0297284.s010.zip › Level 5 processed Sample/processed_21/scar/AHA_scar.jpg]

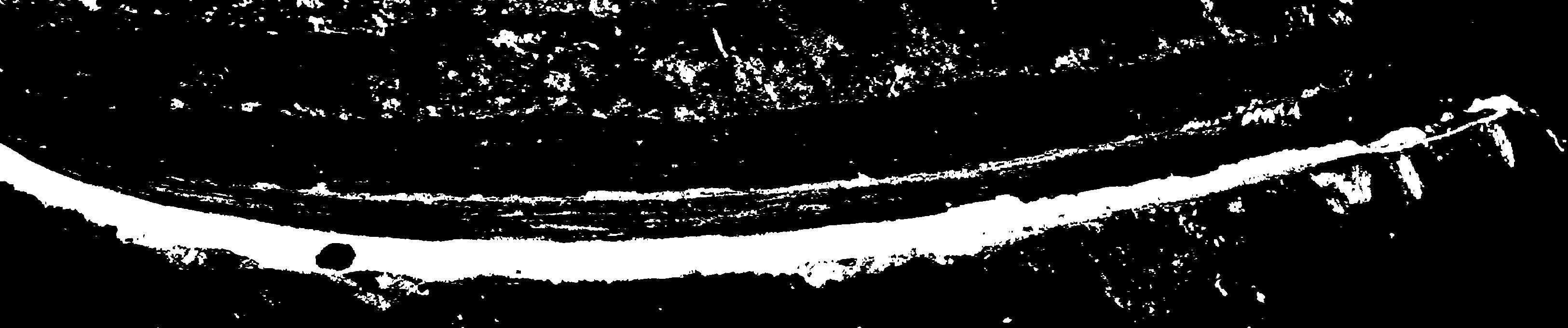

Supplement: S10 Data — (ZIP) [file pone.0297284.s010.zip › Level 5 processed Sample/processed_21/scar/DBO_scar.jpg]

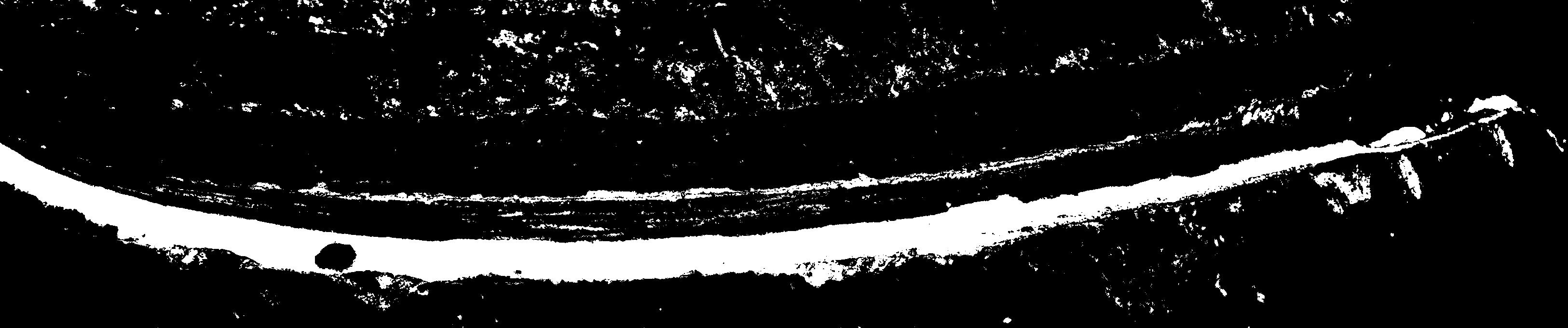

Supplement: S10 Data — (ZIP) [file pone.0297284.s010.zip › Level 5 processed Sample/processed_21/scar/WSO_scar.jpg]

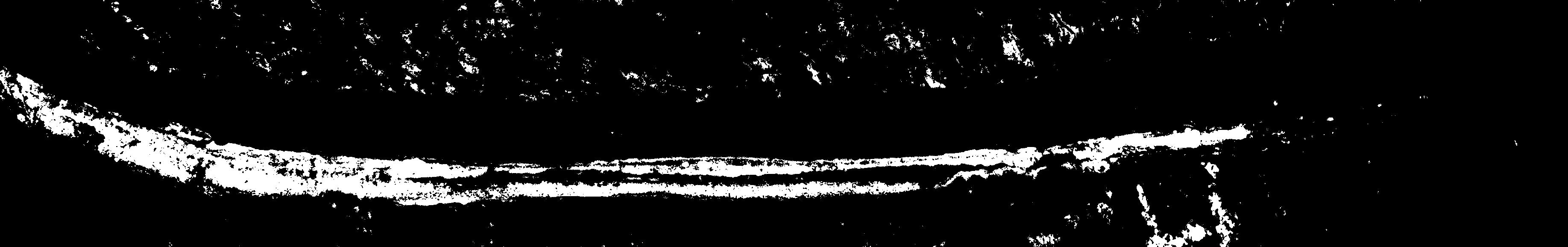

Supplement: S10 Data — (ZIP) [file pone.0297284.s010.zip › Level 5 processed Sample/processed_22/latex/AHA_latex.jpg]

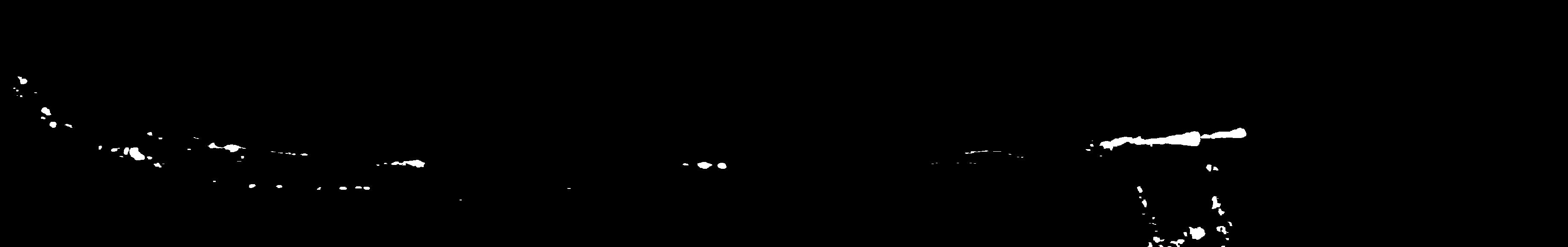

Supplement: S10 Data — (ZIP) [file pone.0297284.s010.zip › Level 5 processed Sample/processed_22/latex/DBO_latex.jpg]

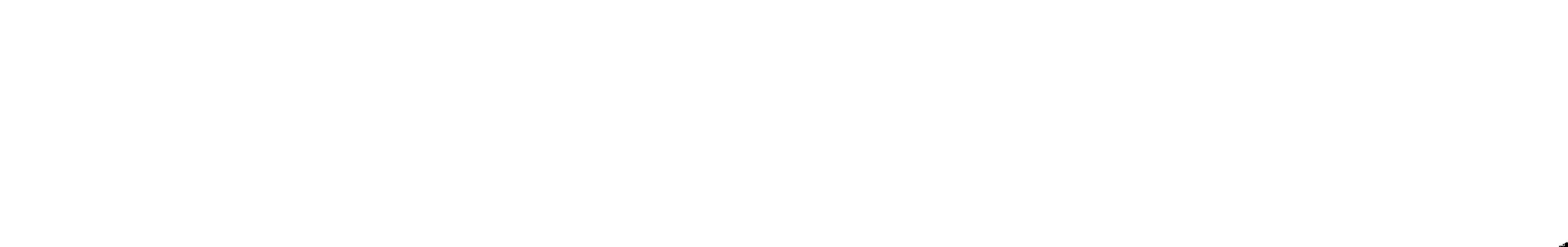

Supplement: S10 Data — (ZIP) [file pone.0297284.s010.zip › Level 5 processed Sample/processed_22/latex/OTSU_latex.jpg]

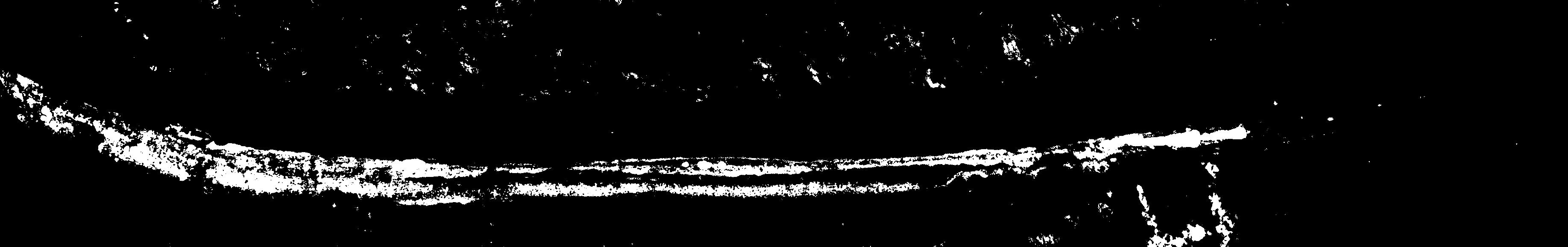

Supplement: S10 Data — (ZIP) [file pone.0297284.s010.zip › Level 5 processed Sample/processed_22/latex/WSO_latex.jpg]

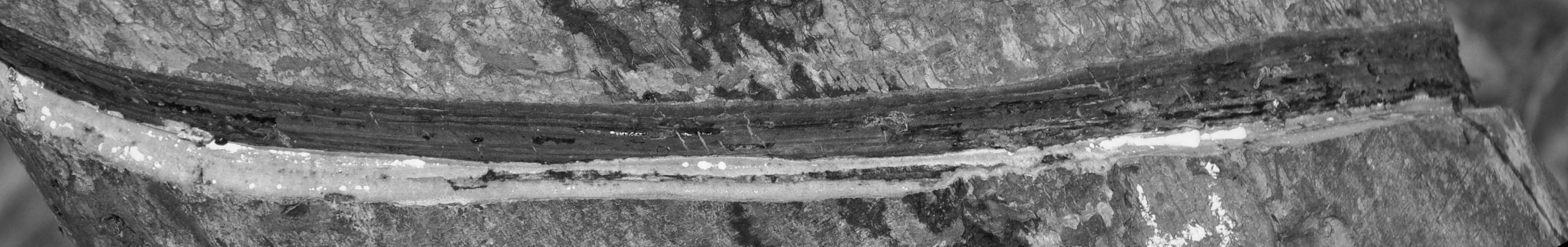

Supplement: S10 Data — (ZIP) [file pone.0297284.s010.zip › Level 5 processed Sample/processed_22/original_image.jpg]

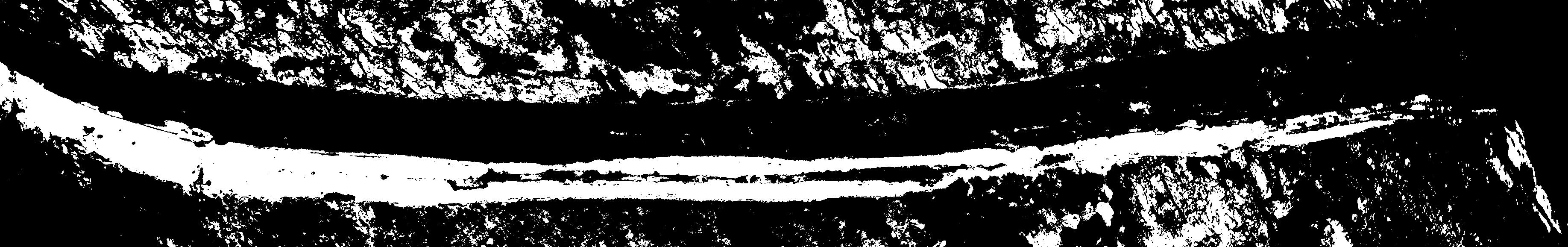

Supplement: S10 Data — (ZIP) [file pone.0297284.s010.zip › Level 5 processed Sample/processed_22/scar/AHA_scar.jpg]

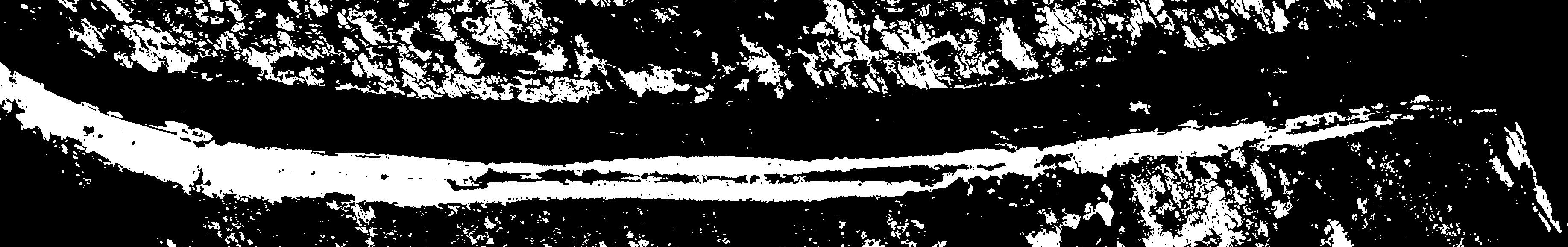

Supplement: S10 Data — (ZIP) [file pone.0297284.s010.zip › Level 5 processed Sample/processed_22/scar/DBO_scar.jpg]

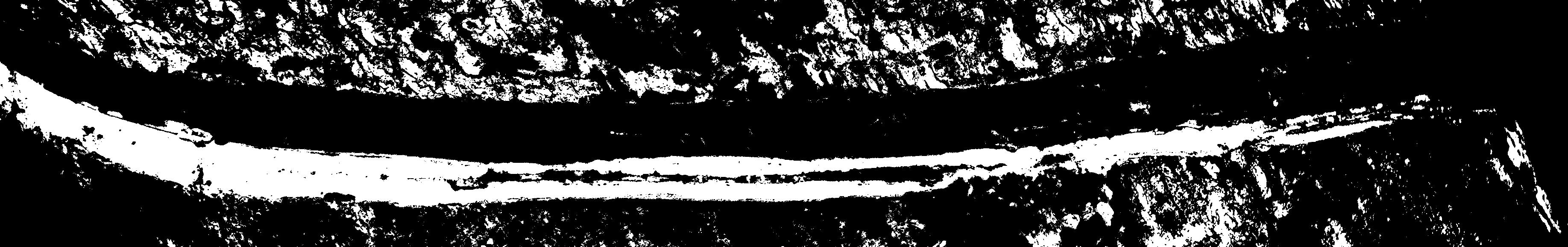

Supplement: S10 Data — (ZIP) [file pone.0297284.s010.zip › Level 5 processed Sample/processed_22/scar/WSO_scar.jpg]

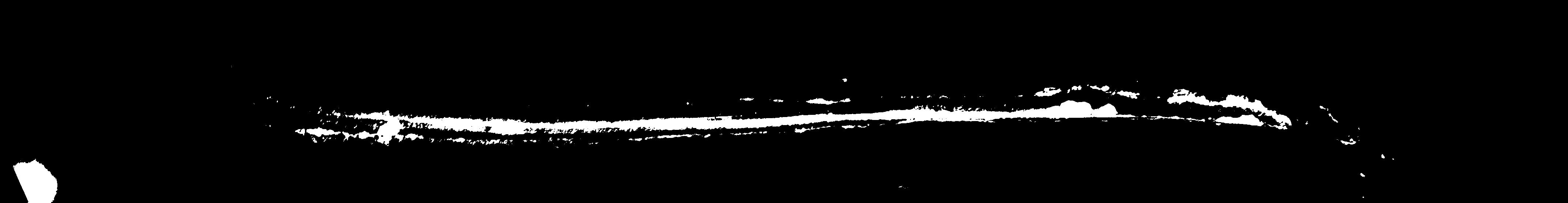

Supplement: S10 Data — (ZIP) [file pone.0297284.s010.zip › Level 5 processed Sample/processed_23/latex/AHA_latex.jpg]

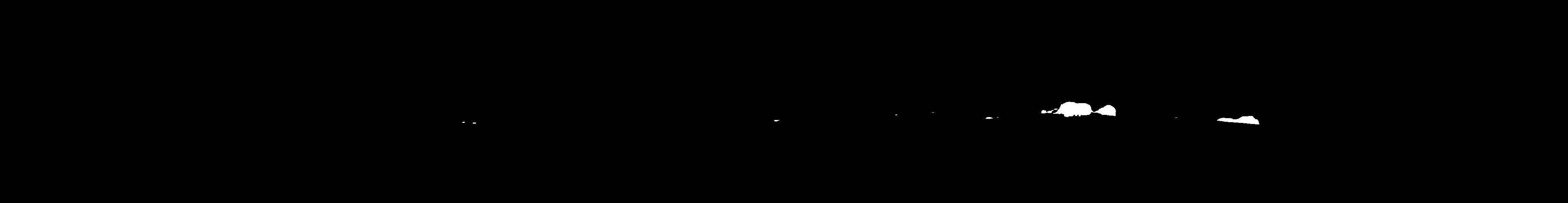

Supplement: S10 Data — (ZIP) [file pone.0297284.s010.zip › Level 5 processed Sample/processed_23/latex/DBO_latex.jpg]

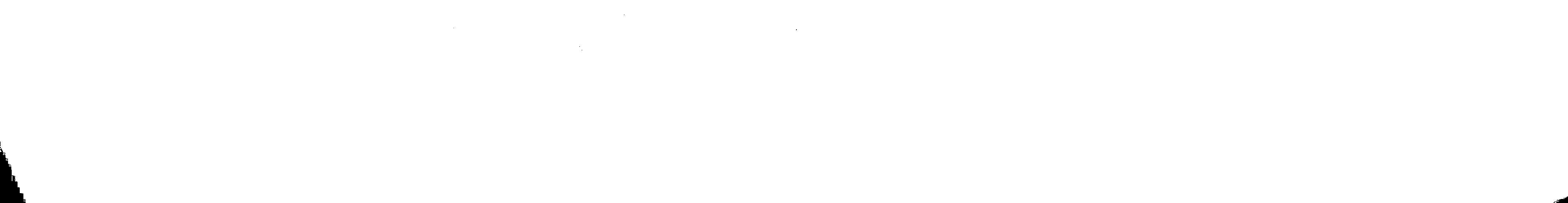

Supplement: S10 Data — (ZIP) [file pone.0297284.s010.zip › Level 5 processed Sample/processed_23/latex/OTSU_latex.jpg]

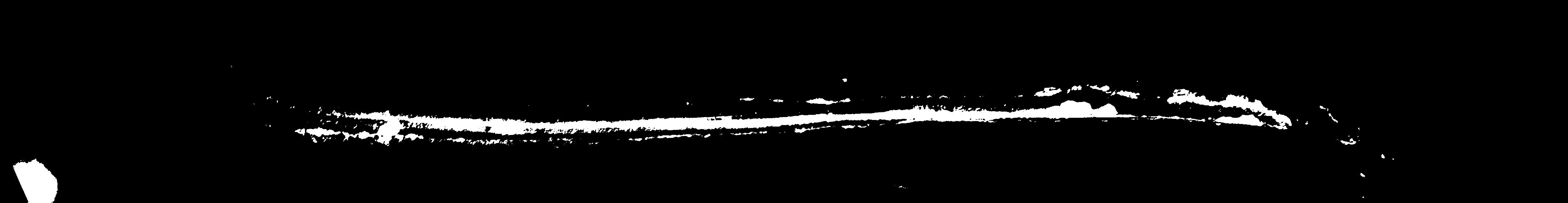

Supplement: S10 Data — (ZIP) [file pone.0297284.s010.zip › Level 5 processed Sample/processed_23/latex/WOA_latex.jpg]

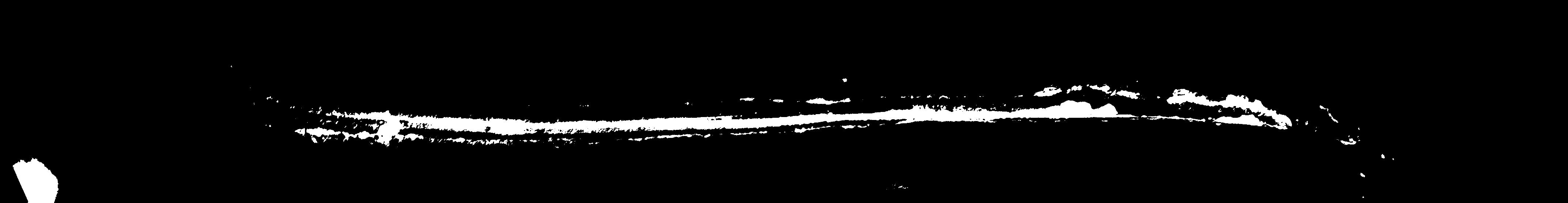

Supplement: S10 Data — (ZIP) [file pone.0297284.s010.zip › Level 5 processed Sample/processed_23/latex/WSO_latex.jpg]

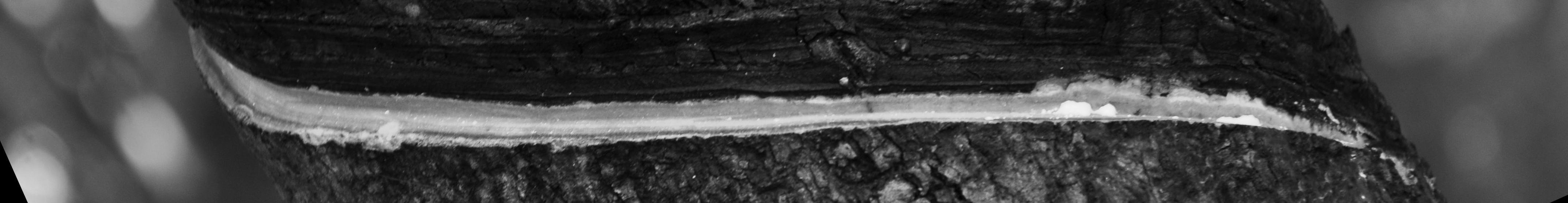

Supplement: S10 Data — (ZIP) [file pone.0297284.s010.zip › Level 5 processed Sample/processed_23/original_image.jpg]

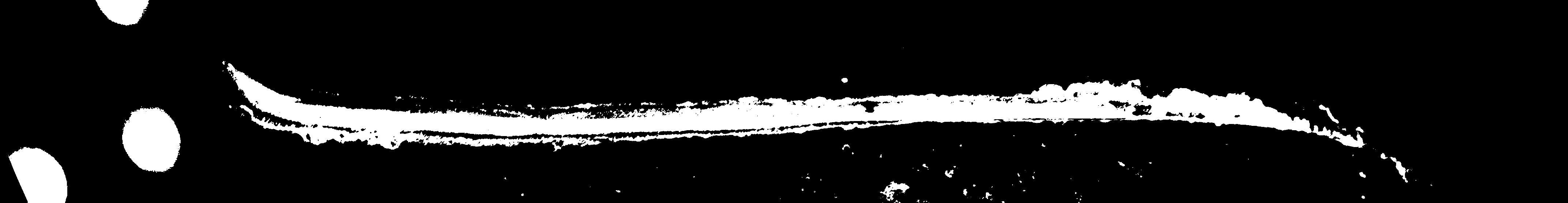

Supplement: S10 Data — (ZIP) [file pone.0297284.s010.zip › Level 5 processed Sample/processed_23/scar/AHA_scar.jpg]

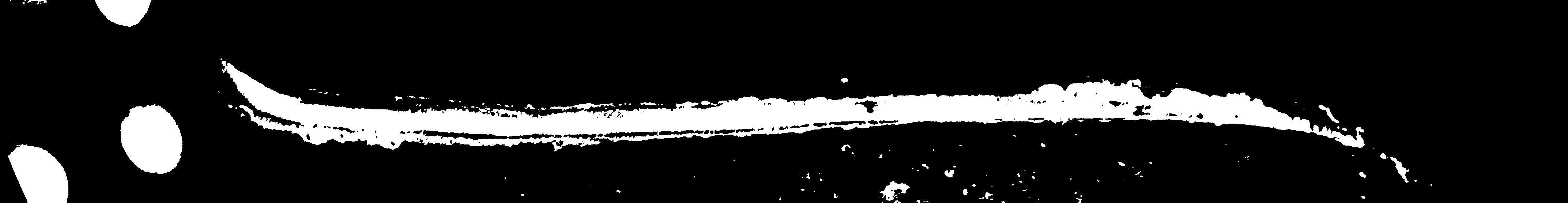

Supplement: S10 Data — (ZIP) [file pone.0297284.s010.zip › Level 5 processed Sample/processed_23/scar/DBO_scar.jpg]

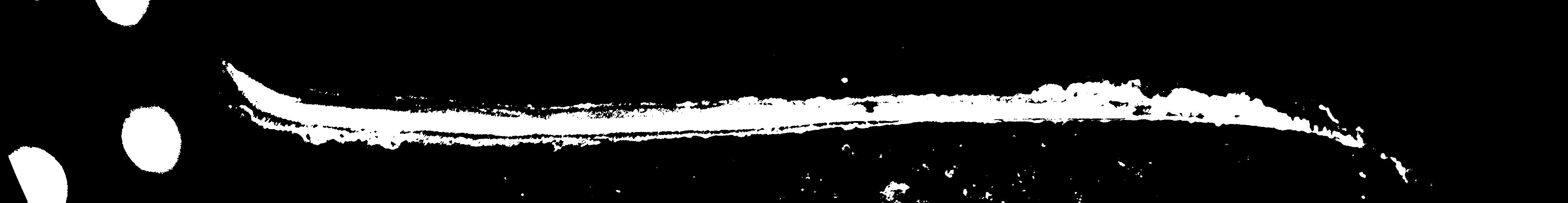

Supplement: S10 Data — (ZIP) [file pone.0297284.s010.zip › Level 5 processed Sample/processed_23/scar/GWO_scar.jpg]

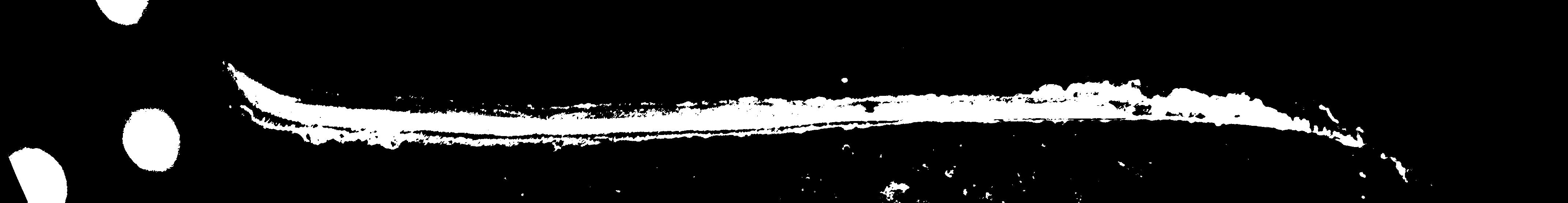

Supplement: S10 Data — (ZIP) [file pone.0297284.s010.zip › Level 5 processed Sample/processed_23/scar/WOA_scar.jpg]

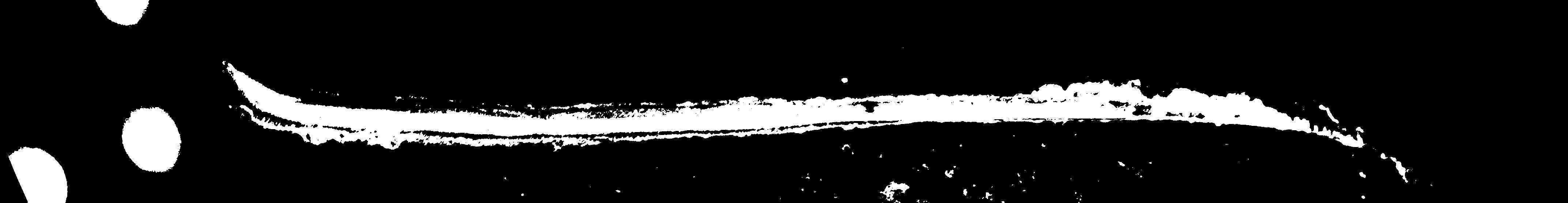

Supplement: S10 Data — (ZIP) [file pone.0297284.s010.zip › Level 5 processed Sample/processed_23/scar/WSO_scar.jpg]

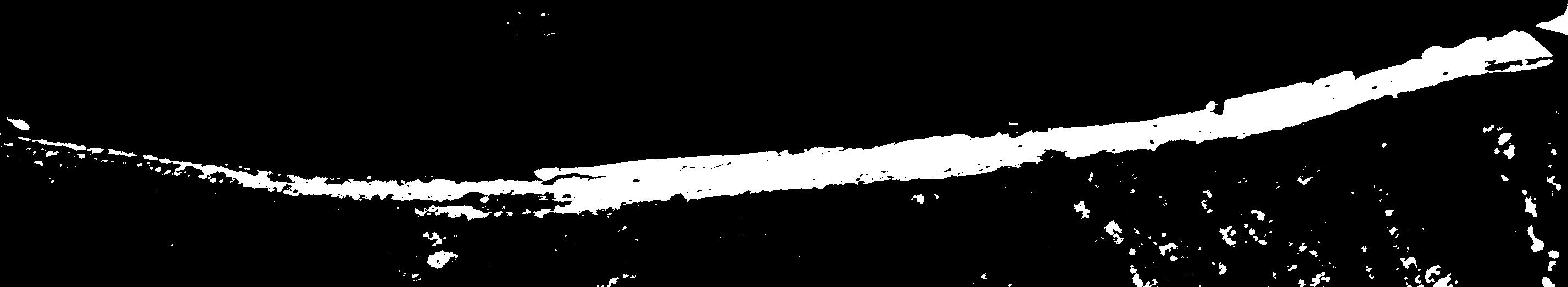

Supplement: S10 Data — (ZIP) [file pone.0297284.s010.zip › Level 5 processed Sample/processed_24/latex/AHA_latex.jpg]

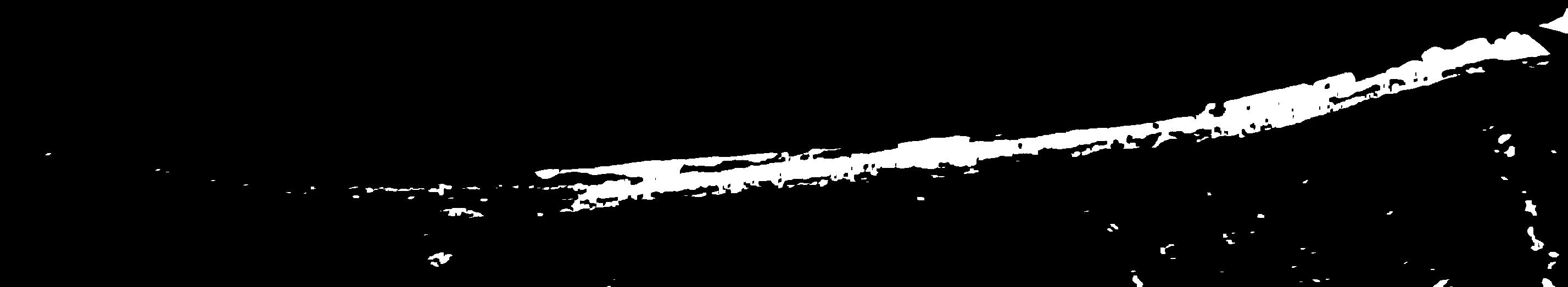

Supplement: S10 Data — (ZIP) [file pone.0297284.s010.zip › Level 5 processed Sample/processed_24/latex/DBO_latex.jpg]

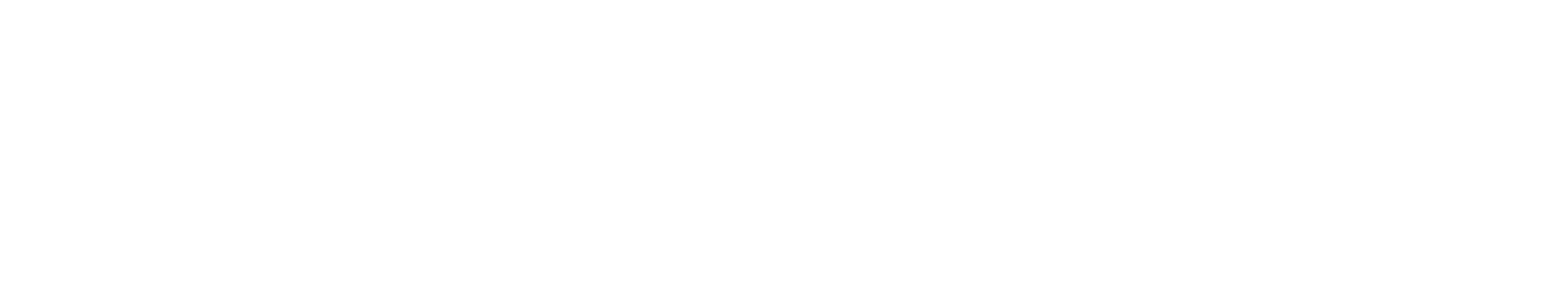

Supplement: S10 Data — (ZIP) [file pone.0297284.s010.zip › Level 5 processed Sample/processed_24/latex/OTSU_latex.jpg]

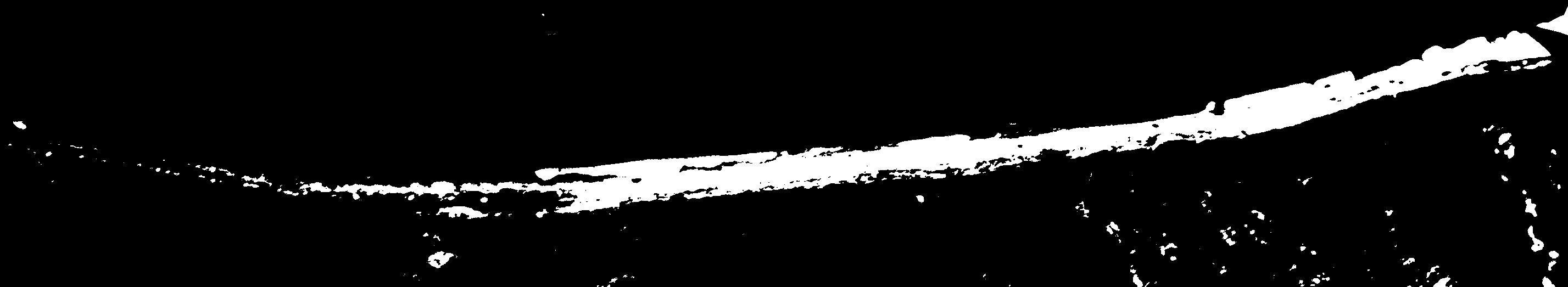

Supplement: S10 Data — (ZIP) [file pone.0297284.s010.zip › Level 5 processed Sample/processed_24/latex/WSO_latex.jpg]

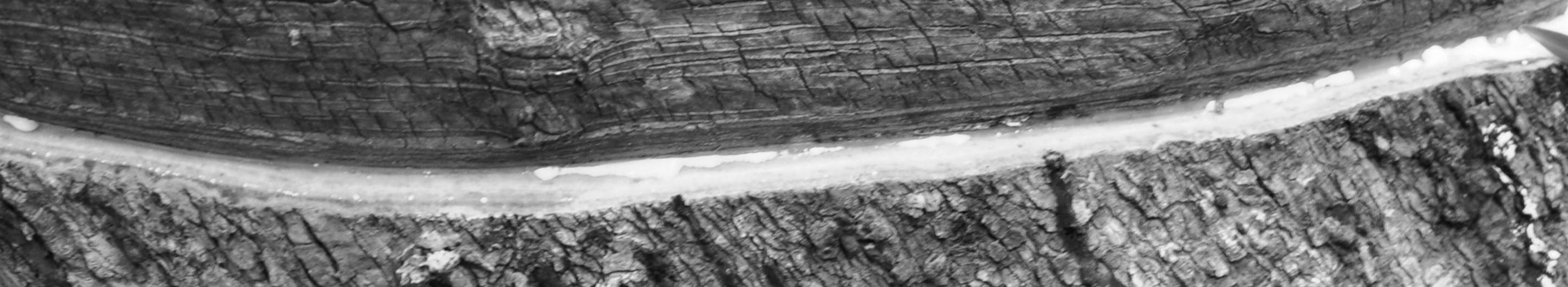

Supplement: S10 Data — (ZIP) [file pone.0297284.s010.zip › Level 5 processed Sample/processed_24/original_image.jpg]

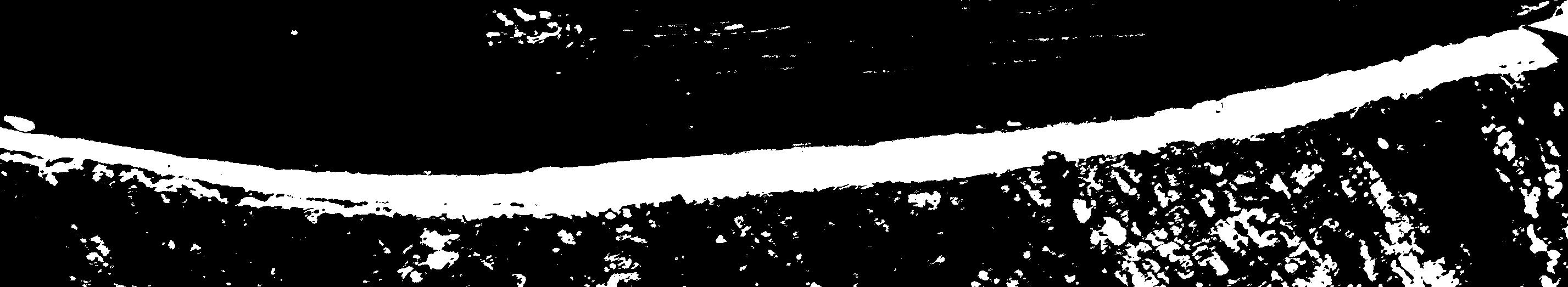

Supplement: S10 Data — (ZIP) [file pone.0297284.s010.zip › Level 5 processed Sample/processed_24/scar/AHA_scar.jpg]

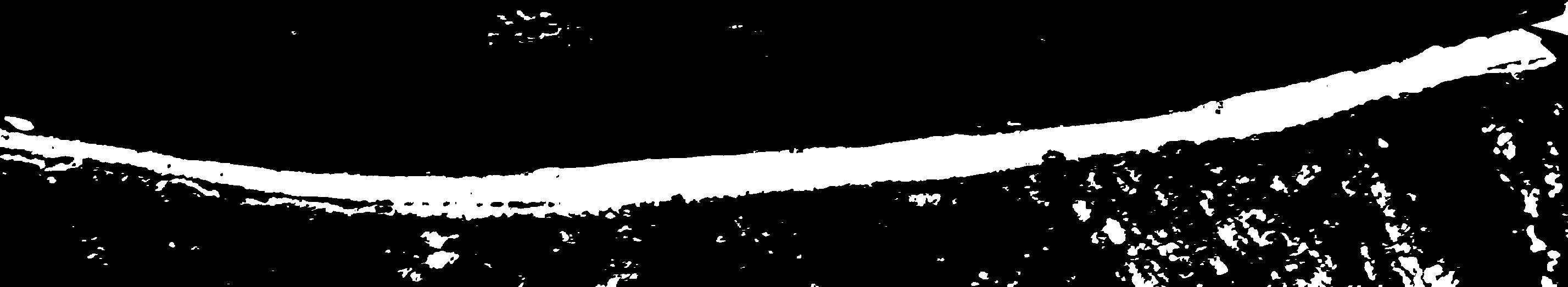

Supplement: S10 Data — (ZIP) [file pone.0297284.s010.zip › Level 5 processed Sample/processed_24/scar/DBO_scar.jpg]

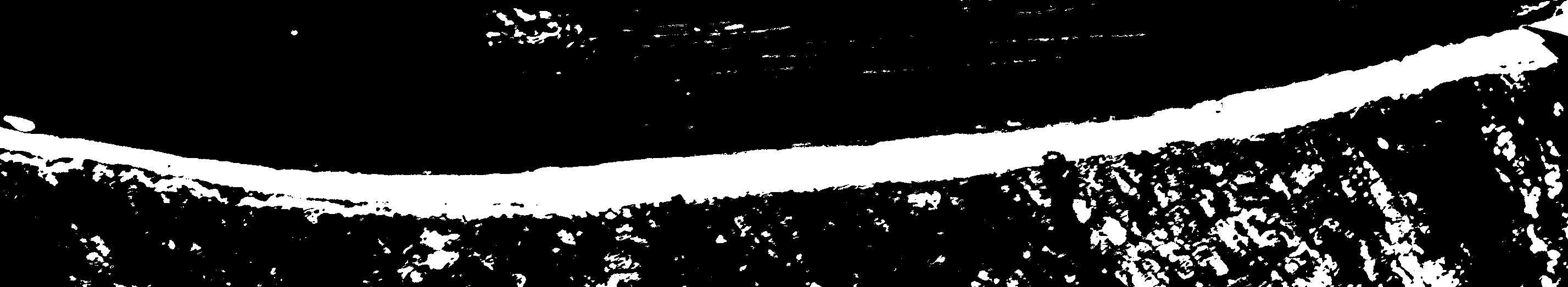

Supplement: S10 Data — (ZIP) [file pone.0297284.s010.zip › Level 5 processed Sample/processed_24/scar/GWO_scar.jpg]

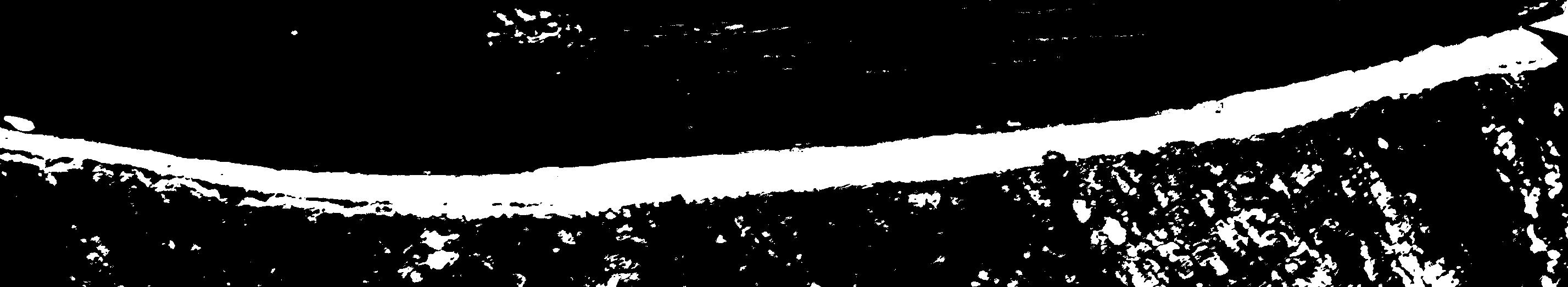

Supplement: S10 Data — (ZIP) [file pone.0297284.s010.zip › Level 5 processed Sample/processed_24/scar/WSO_scar.jpg]

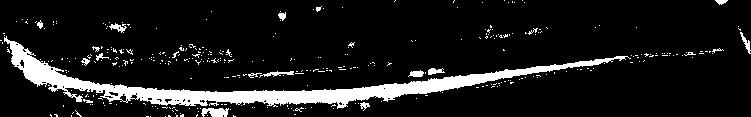

Supplement: S10 Data — (ZIP) [file pone.0297284.s010.zip › Level 5 processed Sample/processed_3/latex/AHA_latex.jpg]

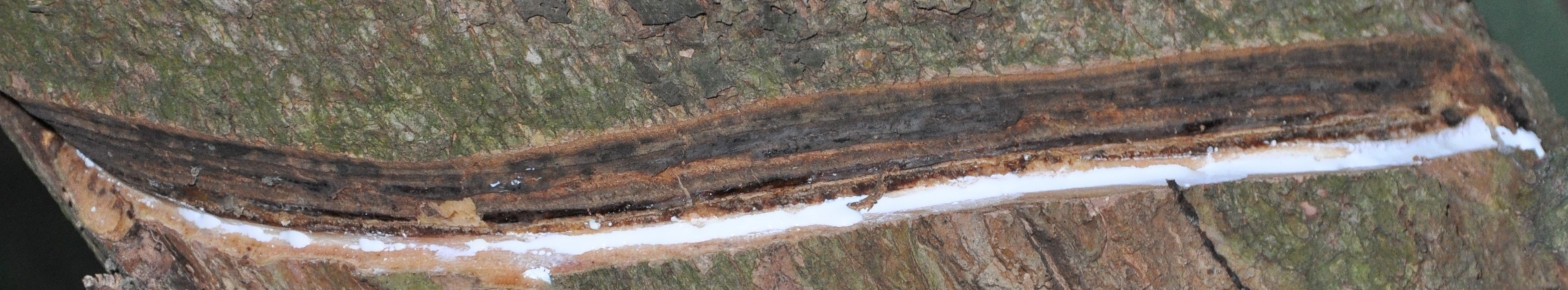

Supplement: S1 File — (ZIP) [file pone.0297284.s011.zip › The first-time code and data/3-6.jpg]

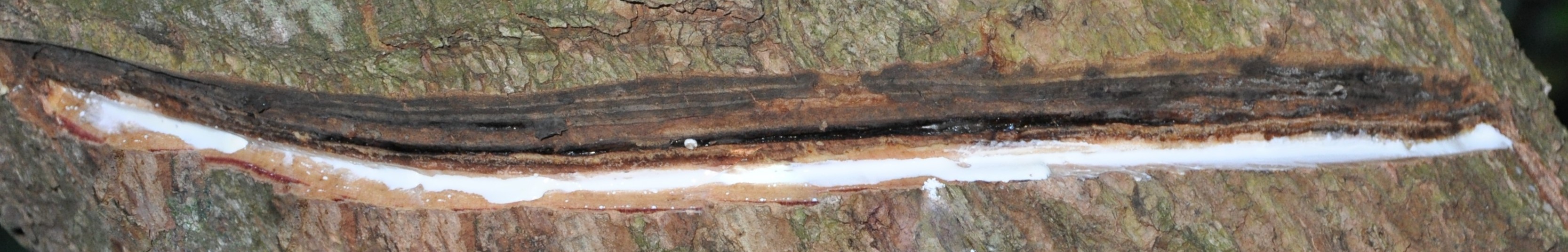

Supplement: S1 File — (ZIP) [file pone.0297284.s011.zip › The first-time code and data/3-7.jpg]

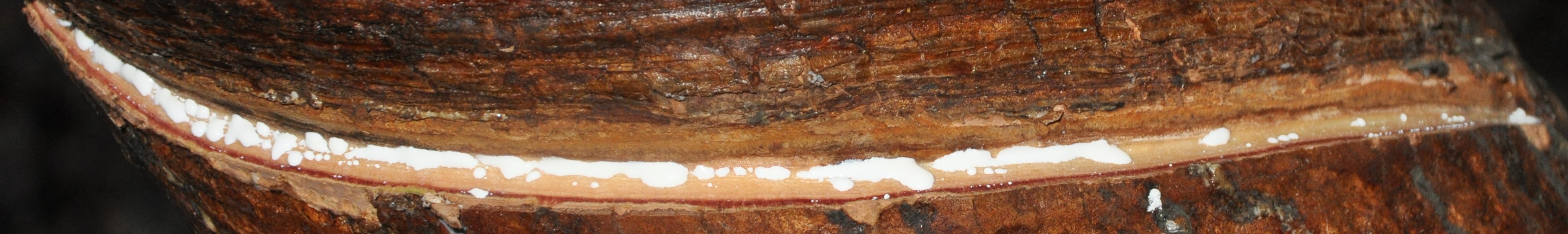

Supplement: S1 File — (ZIP) [file pone.0297284.s011.zip › The first-time code and data/3-8.jpg]
