# Supplementary material for: Advancing image segmentation with DBO-Otsu: Addressing rubber tree diseases through enhanced threshold techniques
Source: PLoS One. 2024 Mar 21;19(3):e0297284. doi: 10.1371/journal.pone.0297284 (PMC10956860; doi:10.1371/journal.pone.0297284)
Supplement: S1 File — (ZIP) [file pone.0297284.s011.zip › The first-time code and data/FR_FSIMc/TIP_IQA_FSIM.pdf]

FSIM: A Feature Similarity Index for Image Quality Assessment

Lin Zhang[a], *Student Member, IEEE,* Lei Zhang[a,1], *Member, IEEE*
Xuanqin Mou[b], *Member, IEEE,* and David Zhang[a], *Fellow, IEEE*

[a]Department of Computing, The Hong Kong Polytechnic University, Hong Kong
[b]Institute of Image Processing and Pattern Recognition, Xi'an Jiaotong University, China

Abstract: Image quality assessment (IQA) aims to use computational models to measure the image quality consistently with subjective evaluations. The well-known structural-similarity (SSIM) index brings IQA from pixel-based stage to structure-based stage. In this paper, a novel feature-similarity (FSIM) index for full reference IQA is proposed based on the fact that human visual system (HVS) understands an image mainly according to its low-level features. Specifically, the phase congruency (PC), which is a dimensionless measure of the significance of a local structure, is used as the primary feature in FSIM. Considering that PC is contrast invariant while the contrast information does affect HVS' perception of image quality, the image gradient magnitude (GM) is employed as the secondary feature in FSIM. PC and GM play complementary roles in characterizing the image local quality. After calculating the similarity between reference and distorted PC or GM maps, we use PC again as a weighting function to derive a single quality score. Extensive experiments performed on six benchmark IQA databases demonstrate that FSIM can achieve much higher consistency with the subjective evaluations than state-of-the-art IQA metrics.

Index Terms: Image quality assessment, phase congruency, gradient, low-level feature

I. INTRODUCTION

With the rapid proliferation of digital imaging and communication technologies, image quality assessment (IQA) has been becoming an important issue in numerous applications such as image acquisition, transmission, compression, restoration and enhancement, etc. Since the subjective IQA methods cannot be

[1] Corresponding author. Email: cslzhang@comp.polyu.edu.hk. This project is supported by the Hong Kong RGC General Research Fund (PolyU 5330/07E) and the Ho Tung Fund (5-ZH25).

readily and routinely used for many scenarios, e.g. real-time and automated systems, it is necessary to develop objective IQA metrics to automatically and robustly measure the image quality. Meanwhile, it is anticipated that the evaluation results should be statistically consistent with those of the human observers. To this end, the scientific community has developed various IQA methods in the past decades. According to the availability of a reference image, objective IQA metrics can be classified as full reference (FR), no-reference (NR) and reduced-reference (RR) methods [1]. In this paper, the discussion is confined to FR methods, where the original "distortion free" image is known as the reference image.

The conventional metrics such as the peak signal-to-noise ratio (PSNR) and the mean squared error (MSE) operate directly on the intensity of the image and they do not correlate well with the subjective fidelity ratings. Thus many efforts have been made on designing human visual system (HVS) based IQA metrics. Such kinds of models emphasize the importance of HVS' sensitivity to different visual signals, such as the luminance, the contrast, the frequency content, and the interaction between different signal components [2-4]. The noise quality measure (NQM) [2] and the visual signal-to-noise ratio (VSNR) [3] are two representatives. Methods such as the structural similarity (SSIM) index [1] are motivated by the need to capture the loss of structure in the image. SSIM is based on the hypothesis that HVS is highly adapted to extract the structural information from the visual scene; therefore, a measurement of structural similarity should provide a good approximation of perceived image quality. The multi-scale extension of SSIM, called MS-SSIM [5], produces better results than its single-scale counterpart. In [6], the authors presented a 3-component weighted SSIM (3-SSIM) by assigning different weights to the SSIM scores according to the local region type: edge, texture or smooth area. In [7], Sheikh *et al.* introduced the information theory into image fidelity measurement, and proposed the information fidelity criterion (IFC) for IQA by quantifying the information shared between the distorted and the reference images. IFC was later extended to the visual information fidelity (VIF) metric in [4]. In [8], Sampat *et al.* made use of the steerable complex wavelet transform to measure the structural similarity of the two images and proposed the CW-SSIM index.

The great success of SSIM and its extensions owes to the fact that HVS is adapted to the structural information in images. The visual information in an image, however, is often very redundant, while the HVS understands an image mainly based on its low-level features, such as edges and zero-crossings [9-11]. In other words, the salient low-level features convey crucial information for the HVS to interpret the scene. Accordingly, perceptible image degradations will lead to perceptible changes in image low-level features,

and hence a good IQA metric could be devised by comparing the low-level feature sets between the reference image and the distorted image. Based on the above analysis, in this paper we propose a novel low-level feature similarity induced FR IQA metric, namely FSIM (Feature SIMilarity).

One key issue is then what kinds of features could be used in designing FSIM? Based on the physiological and psychophysical evidence, it is found that visually discernable features coincide with those points where the Fourier waves at different frequencies have congruent phases [11-14]. That is, at points of high phase congruency (PC) we can extract highly informative features. Such a conclusion has been further corroborated by some recent studies in neurobiology using functional magnetic resonance imaging (fMRI) [15]. Therefore, PC is used as the primary feature in computing FSIM. Meanwhile, considering that PC is contrast invariant but image local contrast does affect HVS' perception on the image quality, the image gradient magnitude (GM) is computed as the secondary feature to encode the contrast information. PC and GM are complementary and they reflect different aspects of the HVS in assessing the local quality of the input image. After computing the local similarity map, PC is utilized again as a weighting function to derive a single similarity score. Although FSIM is designed for grayscale images (or the luminance components of color images), the chrominance information can be easily incorporated by means of a simple extension of FSIM, and we call this extension $FSIM_C$.

PC has already been used for IQA in the literature. In [16], Liu and Laganière proposed a PC-based IQA metric. In their method, PC maps are partitioned into sub-blocks of size 5×5. Then, the cross correlation is used to measure the similarity between two corresponding PC sub-blocks. The overall similarity score is obtained by averaging the cross correlation values from all block pairs. In [17], PC was extended to phase coherence which can be used to characterize the image blur. Based on [17], Hassen *et al.* proposed an NR IQA metric to assess the sharpness of an input image [18].

The proposed FSIM and $FSIM_C$ are evaluated on six benchmark IQA databases in comparison with eight state-of-the-art IQA methods. The extensive experimental results show that FSIM and $FSIM_C$ can achieve very high consistency with human subjective evaluations, outperforming all the other competitors. Particularly, FSIM and $FSIM_C$ work consistently well across all the databases, while other methods may work well only on some specific databases. To facilitate repeatable experimental verifications and comparisons, the Matlab source code of the proposed FSIM/$FSIM_C$ indices and our evaluation results are available online at http://www.comp.polyu.edu.hk/~cslzhang/IQA/FSIM/FSIM.htm.

The remainder of this paper is organized as follows. Section II discusses the extraction of PC and GM. Section III presents in detail the computation of the FSIM and FSIM$_C$ indices. Section IV reports the experimental results. Finally, Section V concludes the paper.

II. EXTRACTION OF PHASE CONGRUENCY AND GRADIENT MAGNITUDE

A. Phase congruency (PC)

Rather than define features directly at points with sharp changes in intensity, the PC model postulates that features are perceived at points where the Fourier components are maximal in phase. Based on the physiological and psychophysical evidences, the PC theory provides a simple but biologically plausible model of how mammalian visual systems detect and identify features in an image [11-15]. PC can be considered as a dimensionless measure for the significance of a local structure.

Under the definition of PC in [12], there can be different implementations to compute the PC map of a given image. In this paper we adopt the method developed by Kovesi in [14], which is widely used in literature. We start from the 1D signal $g(x)$. Denote by M_n^e and M_n^o the even-symmetric and odd-symmetric filters on scale n and they form a quadrature pair. Responses of each quadrature pair to the signal will form a response vector at position x on scale n: $[e_n(x), o_n(x)] = [g(x)* M_n^e, g(x)* M_n^o]$, and the local amplitude on scale n is $A_n(x) = \sqrt{e_n(x)^2 + o_n(x)^2}$. Let $F(x) = \sum_n e_n(x)$ and $H(x) = \sum_n o_n(x)$. The 1D PC can be computed as

$$PC(x) = E(x) / \left(\varepsilon + \sum_n A_n(x)\right) \tag{1}$$

where $E(x) = \sqrt{F^2(x) + H^2(x)}$ and ε is a small positive constant.

With respect to the quadrature pair of filters, i.e. M_n^e and M_n^o, Gabor filters [19] and log-Gabor filters [20] are two widely used candidates. We adopt the log-Gabor filters because: 1) one cannot construct Gabor filters of arbitrarily bandwidth and still maintain a reasonably small DC component in the even-symmetric filter, while log-Gabor filters, by definition, have no DC component; and 2) the transfer function of the log-Gabor filter has an extended tail at the high frequency end, which makes it more capable to encode natural images than ordinary Gabor filters [14, 20]. The transfer function of a log-Gabor filter in the frequency domain is $G(\omega) = \exp(-(\log(\omega/\omega_0))^2/2\sigma_r^2)$, where ω_0 is the filter's center frequency and σ_r controls the filter's bandwidth.

To compute the PC of 2D grayscale images, we can apply the 1D analysis over several orientations and then combine the results using some rule. The 1D log-Gabor filters described above can be extended to 2D ones by simply applying some spreading function across the filter perpendicular to its orientation. By using Gaussian as the spreading function, the 2D log-Gabor function has the following transfer function

$$G_2(\omega,\theta_j) = \exp\left(-\frac{\left(\log(\omega/\omega_0)\right)^2}{2\sigma_r^2}\right)\cdot\exp\left(-\frac{\left(\theta-\theta_j\right)^2}{2\sigma_\theta^2}\right) \tag{2}$$

where $\theta_j = j\pi / J$, j = {0,1,…, J-1} is the orientation angle of the filter, J is the number of orientations and σ_θ determines the filter's angular bandwidth. An example of the 2D log-Gabor filter in the frequency domain, with ω_0 = 1/6, θ_j = 0, σ_r = 0.3, and σ_θ = 0.4, is shown in Fig. 1.

By modulating ω_0 and θ_j and convolving G_2 with the 2D image, we get a set of responses at each point **x** as $\left[e_{n,\theta_j}(\mathbf{x}), o_{n,\theta_j}(\mathbf{x})\right]$. The local amplitude on scale n and orientation θ_j is $A_{n,\theta_j}(\mathbf{x}) = \sqrt{e_{n,\theta_j}(\mathbf{x})^2 + o_{n,\theta_j}(\mathbf{x})^2}$, and the local energy along orientation θ_j is $E_{\theta_j}(\mathbf{x}) = \sqrt{F_{\theta_j}(\mathbf{x})^2 + H_{\theta_j}(\mathbf{x})^2}$, where $F_{\theta_j}(\mathbf{x}) = \sum_n e_{n,\theta_j}(\mathbf{x})$ and $H_{\theta_j}(\mathbf{x}) = \sum_n o_{n,\theta_j}(\mathbf{x})$. The 2D PC at **x** is defined as

$$PC_{2D}(\mathbf{x}) = \frac{\sum_j E_{\theta_j}(\mathbf{x})}{\varepsilon + \sum_n \sum_j A_{n,\theta_j}(\mathbf{x})} \tag{3}$$

It should be noted that $PC_{2D}(\mathbf{x})$ is a real number within 0 ~ 1. Examples of the PC maps of 2D images can be found in Fig. 2.

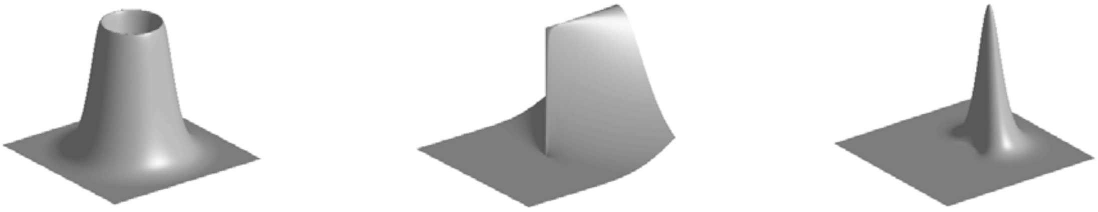

(a) (b) (c)

Fig. 1: An example of the log-Gabor filter in the frequency domain, with ω_0 = 1/6, θ_j = 0, σ_r = 0.3, and σ_θ = 0.4. (a) The radial component of the filter. (b) The angular component of the filter. (c) The log-Gabor filter, which is the product of the radial component and the angular component.

B. Gradient magnitude (GM)

Image gradient computation is a traditional topic in image processing. Gradient operators can be expressed

by convolution masks. Three commonly used gradient operators are the Sobel operator [21], the Prewitt operator [21] and the Scharr operator [22]. Their performances will be examined in the section of experimental results. The partial derivatives $G_x(\mathbf{x})$ and $G_y(\mathbf{x})$ of the image $f(\mathbf{x})$ along horizontal and vertical directions using the three gradient operators are listed in Table I. The gradient magnitude (GM) of $f(\mathbf{x})$ is then defined as $G = \sqrt{G_x^2 + G_y^2}$.

TABLE I. PARTIAL DERIVATIVES OF $f(\mathbf{x})$ USING DIFFERENT GRADIENT OPERATORS

	Sobel	Prewitt	Scharr
$G_x(\mathbf{x})$	$\frac{1}{4}\begin{bmatrix}1 & 0 & -1\\ 2 & 0 & -2\\ 1 & 0 & -1\end{bmatrix} * f(\mathbf{x})$	$\frac{1}{3}\begin{bmatrix}1 & 0 & -1\\ 1 & 0 & -1\\ 1 & 0 & -1\end{bmatrix} * f(\mathbf{x})$	$\frac{1}{16}\begin{bmatrix}3 & 0 & -3\\ 10 & 0 & -10\\ 3 & 0 & -3\end{bmatrix} * f(\mathbf{x})$
$G_y(\mathbf{x})$	$\frac{1}{4}\begin{bmatrix}1 & 2 & 1\\ 0 & 0 & 0\\ -1 & -2 & -1\end{bmatrix} * f(\mathbf{x})$	$\frac{1}{3}\begin{bmatrix}1 & 1 & 1\\ 0 & 0 & 0\\ -1 & -1 & -1\end{bmatrix} * f(\mathbf{x})$	$\frac{1}{16}\begin{bmatrix}3 & 10 & 3\\ 0 & 0 & 0\\ -3 & -10 & -3\end{bmatrix} * f(\mathbf{x})$

III. THE FEATURE SIMILARITY (FSIM) INDEX

With the extracted PC and GM feature maps, in this section we present a novel Feature SIMilarity (FSIM) index for IQA. Suppose that we are going to calculate the similarity between images f_1 and f_2. Denote by PC_1 and PC_2 the PC maps extracted from f_1 and f_2, and G_1 and G_2 the GM maps extracted from them. It should be noted that for color images, PC and GM features are extracted from their luminance channels. FSIM will be defined and computed based on PC_1, PC_2, G_1 and G_2. Furthermore, by incorporating the image chrominance information into FSIM, an IQA index for color images, denoted by FSIM$_C$, will be obtained.

A. The FSIM index

The computation of FSIM index consists of two stages. In the first stage, the local similarity map is computed, and then in the second stage, we pool the similarity map into a single similarity score.

We separate the feature similarity measurement between $f_1(\mathbf{x})$ and $f_2(\mathbf{x})$ into two components, each for PC or GM. First, the similarity measure for $PC_1(\mathbf{x})$ and $PC_2(\mathbf{x})$ is defined as

$$S_{PC}(\mathbf{x}) = \frac{2PC_1(\mathbf{x}) \cdot PC_2(\mathbf{x}) + T_1}{PC_1^2(\mathbf{x}) + PC_2^2(\mathbf{x}) + T_1} \tag{4}$$

where T_1 is a positive constant to increase the stability of S_{PC} (such a consideration was also included in SSIM [1]). In practice, the determination of T_1 depends on the dynamic range of PC values. Eq. (4) is a commonly used measure to define the similarity of two positive real numbers [1] and its result ranges within (0, 1]. Similarly, the GM values $G_1(\mathbf{x})$ and $G_2(\mathbf{x})$ are compared and the similarity measure is defined as

$$S_G(\mathbf{x}) = \frac{2G_1(\mathbf{x}) \cdot G_2(\mathbf{x}) + T_2}{G_1^2(\mathbf{x}) + G_2^2(\mathbf{x}) + T_2} \tag{5}$$

where T_2 is a positive constant depending on the dynamic range of GM values. In our experiments, both T_1 and T_2 will be fixed to all databases so that the proposed FSIM can be conveniently used. Then, $S_{PC}(\mathbf{x})$ and $S_G(\mathbf{x})$ are combined to get the similarity $S_L(\mathbf{x})$ of $f_1(\mathbf{x})$ and $f_2(\mathbf{x})$. We define $S_L(\mathbf{x})$ as

$$S_L(\mathbf{x}) = [S_{PC}(\mathbf{x})]^\alpha \cdot [S_G(\mathbf{x})]^\beta \tag{6}$$

where α and β are parameters used to adjust the relative importance of PC and GM features. In this paper, we set $\alpha = \beta = 1$ for simplicity. Thus, $S_L(\mathbf{x}) = S_{PC}(\mathbf{x}) \cdot S_G(\mathbf{x})$.

Having obtained the similarity $S_L(\mathbf{x})$ at each location $\mathbf{x}$, the overall similarity between f_1 and f_2 can be calculated. However, different locations have different contributions to HVS' perception of the image. For example, edge locations convey more crucial visual information than the locations within a smooth area. Since human visual cortex is sensitive to phase congruent structures [15], the PC value at a location can reflect how likely it is a perceptibly significant structure point. Intuitively, for a given location $\mathbf{x}$, if anyone of $f_1(\mathbf{x})$ and $f_2(\mathbf{x})$ has a significant PC value, it implies that this position $\mathbf{x}$ will have a high impact on HVS in evaluating the similarity between f_1 and f_2. Therefore, we use $PC_m(\mathbf{x}) = \max(PC_1(\mathbf{x}), PC_2(\mathbf{x}))$ to weight the importance of $S_L(\mathbf{x})$ in the overall similarity between f_1 and f_2, and accordingly the FSIM index between f_1 and f_2 is defined as

$$\text{FSIM} = \frac{\sum_{\mathbf{x} \in \Omega} S_L(\mathbf{x}) \cdot PC_m(\mathbf{x})}{\sum_{\mathbf{x} \in \Omega} PC_m(\mathbf{x})} \tag{7}$$

where Ω means the whole image spatial domain.

B. Extension to color image quality assessment

The FSIM index is designed for grayscale images or the luminance components of color images. Since the chrominance information will also affect HVS in understanding the images, better performance can be expected if the chrominance information is incorporated in FSIM for color IQA. Such a goal can be achieved by applying a straightforward extension to the FSIM framework.

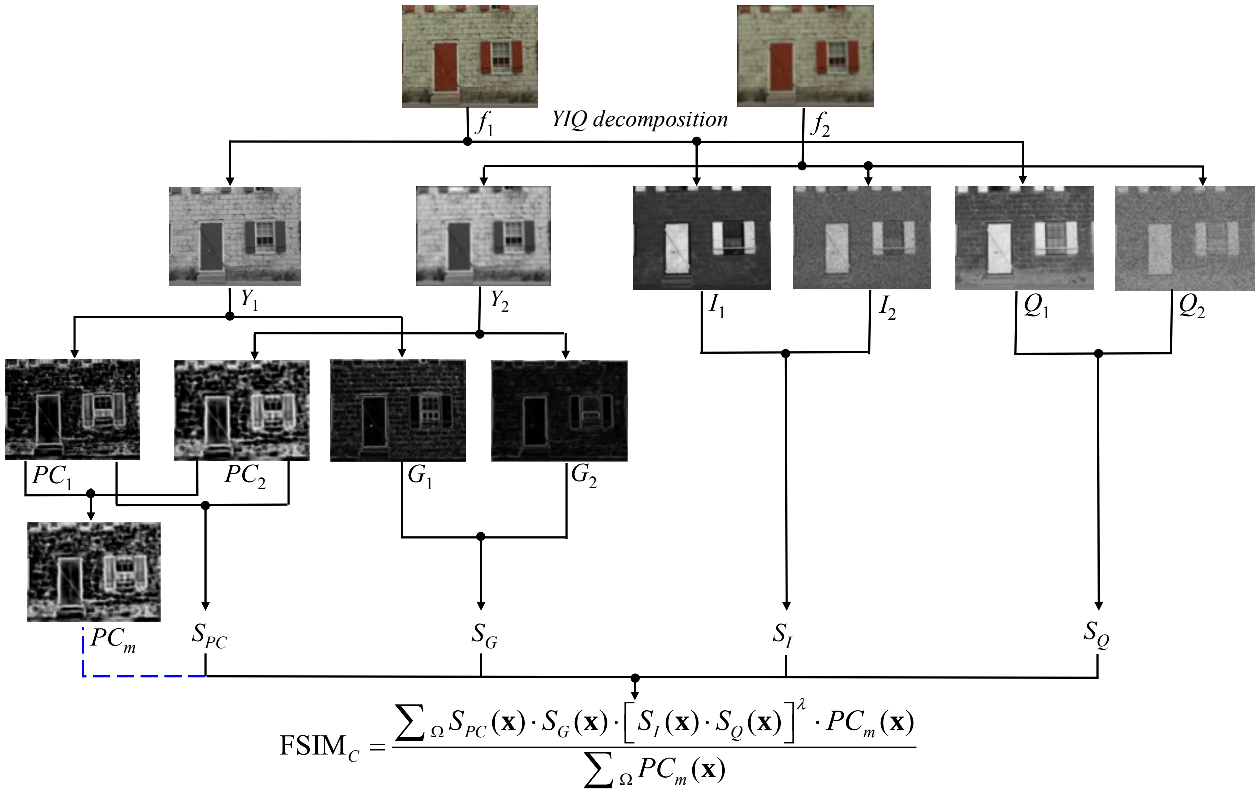

$$\text{FSIM}_C = \frac{\sum_{\Omega} S_{PC}(\mathbf{x}) \cdot S_G(\mathbf{x}) \cdot \left[S_I(\mathbf{x}) \cdot S_Q(\mathbf{x}) \right]^{\lambda} \cdot PC_m(\mathbf{x})}{\sum_{\Omega} PC_m(\mathbf{x})}$$

Fig. 2: Illustration for the FSIM/FSIM$_C$ index computation. f_1 is the reference image and f_2 is a distorted version of f_1.

At first, the original RGB color images are converted into another color space, where the luminance can be separated from the chrominance. To this end, we adopt the widely used YIQ color space [23], in which Y represents the luminance information and I and Q convey the chrominance information. The transform from the RGB space to the YIQ space can be accomplished via [23]:

$$\begin{bmatrix} Y \\ I \\ Q \end{bmatrix} = \begin{bmatrix} 0.299 & 0.587 & 0.114 \\ 0.596 & -0.274 & -0.322 \\ 0.211 & -0.523 & 0.312 \end{bmatrix} \begin{bmatrix} R \\ G \\ B \end{bmatrix} \quad (8)$$

Let I_1 (I_2) and Q_1 (Q_2) be the I and Q chromatic channels of the image f_1 (f_2), respectively. Similar to the

definitions of $S_{PC}(\mathbf{x})$ and $S_G(\mathbf{x})$, we define the similarity between chromatic features as

$$S_I(\mathbf{x}) = \frac{2I_1(\mathbf{x}) \cdot I_2(\mathbf{x}) + T_3}{I_1^2(\mathbf{x}) + I_2^2(\mathbf{x}) + T_3}, \quad S_Q(\mathbf{x}) = \frac{2Q_1(\mathbf{x}) \cdot Q_2(\mathbf{x}) + T_4}{Q_1^2(\mathbf{x}) + Q_2^2(\mathbf{x}) + T_4} \tag{9}$$

where T_3 and T_4 are positive constants. Since I and Q components have nearly the same dynamic range, in this paper we set $T_3 = T_4$ for simplicity. $S_I(\mathbf{x})$ and $S_Q(\mathbf{x})$ can then be combined to get the chrominance similarity measure, denoted by $S_C(\mathbf{x})$, of $f_1(\mathbf{x})$ and $f_2(\mathbf{x})$:

$$S_C(\mathbf{x}) = S_I(\mathbf{x}) \cdot S_Q(\mathbf{x}) \tag{10}$$

Finally, the FSIM index can be extended to FSIM$_C$ by incorporating the chromatic information in a straightforward manner:

$$\text{FSIM}_C = \frac{\sum_{\mathbf{x}\in\Omega} S_L(\mathbf{x}) \cdot [S_C(\mathbf{x})]^\lambda \cdot PC_m(\mathbf{x})}{\sum_{\mathbf{x}\in\Omega} PC_m(\mathbf{x})} \tag{11}$$

where $\lambda > 0$ is the parameter used to adjust the importance of the chromatic components. The procedures to calculate the FSIM/FSIM$_C$ indices are illustrated in Fig. 2. If the chromatic information is ignored in Fig. 2, the FSIM$_C$ index is reduced to the FSIM index.

IV. EXPERIMENTAL RESULTS AND DISCUSSIONS

A. Databases and methods for comparison

To the best of our knowledge, there are six publicly available image databases in the IQA community, including TID2008 [24], CSIQ [25], LIVE [26], IVC [27], MICT [28] and A57 [29]. All of them will be used here for algorithm validation and comparison. The characteristics of these six databases are summarized in Table II.

TABLE II. BENCHMARK TEST DATABASES FOR IQA

**Database**	Source Images	Distorted Images	Distortion Types	Image Type	Observers
**TID2008**	25	1700	17	color	838
**CSIQ**	30	866	6	color	35
**LIVE**	29	779	5	color	161
**IVC**	10	185	4	color	15
**MICT**	14	168	2	color	16
**A57**	3	54	6	gray	unknown

The performance of the proposed FSIM and FSIM_C indices will be evaluated and compared with eight representative IQA metrics, including seven state-of-the-arts (SSIM [1], MS-SSIM [5], VIF [4], VSNR [3], IFC [7], NQM [2], and Liu *et al*'s method [16]) and the classical PSNR. For Liu *et al*'s method [16], we implemented it by ourselves. For SSIM [1], we used the implementation by the author, which is available at [30]. For all the other methods evaluated, we used the public software MeTriX MuX [31]. The Matlab source code of the proposed FSIM/FSIM_C indices is available online at http://www.comp.polyu.edu.hk/~cslzhang/IQA/FSIM/FSIM.htm.

Four commonly used performance metrics are employed to evaluate the competing IQA metrics. The first two are the Spearman rank-order correlation coefficient (SROCC) and the Kendall rank-order correlation coefficient (KROCC), which can measure the prediction monotonicity of an IQA metric. These two metrics operate only on the rank of the data points and ignore the relative distance between data points. To compute the other two metrics we need to apply a regression analysis, as suggested by the video quality experts group (VQEG) [32], to provide a nonlinear mapping between the objective scores and the subjective mean opinion scores (MOS). The third metric is the Pearson linear correlation coefficient (PLCC) between MOS and the objective scores after nonlinear regression. The fourth metric is the root mean squared error (RMSE) between MOS and the objective scores after nonlinear regression. For the nonlinear regression, we used the following mapping function [33]:

$$f(x) = \beta_1 \left(\frac{1}{2} - \frac{1}{1 + e^{\beta_2 (x - \beta_3)}} \right) + \beta_4 x + \beta_5 \tag{12}$$

where β_i, i =1, 2, …, 5, are the parameters to be fitted. A better objective IQA measure is expected to have higher SROCC, KROCC and PLCC while lower RMSE values.

B. Determination of parameters

There are several parameters need to be determined for FSIM and FSIM_C. To this end, we tuned the parameters based on a sub-dataset of TID2008 database, which contains the first 8 reference images in TID2008 and the associated 544 distorted images. The 8 reference images used in the tuning process are shown in Fig. 3. The tuning criterion was that the parameter value leading to a higher SROCC would be chosen. As a result, the parameters required in the proposed methods were set as: $n = 4$, $J = 4$, $\sigma_r = 0.5978$, σ_θ

$= 0.6545$, $T_1 = 0.85$, $T_2 = 160$, $T_3 = T_4 = 200$, and λ = 0.03. Besides, the center frequencies of the log-Gabor filters at four scales were set as: 1/6, 1/12, 1/24 and 1/48. These parameters were then fixed for all the following experiments conducted.

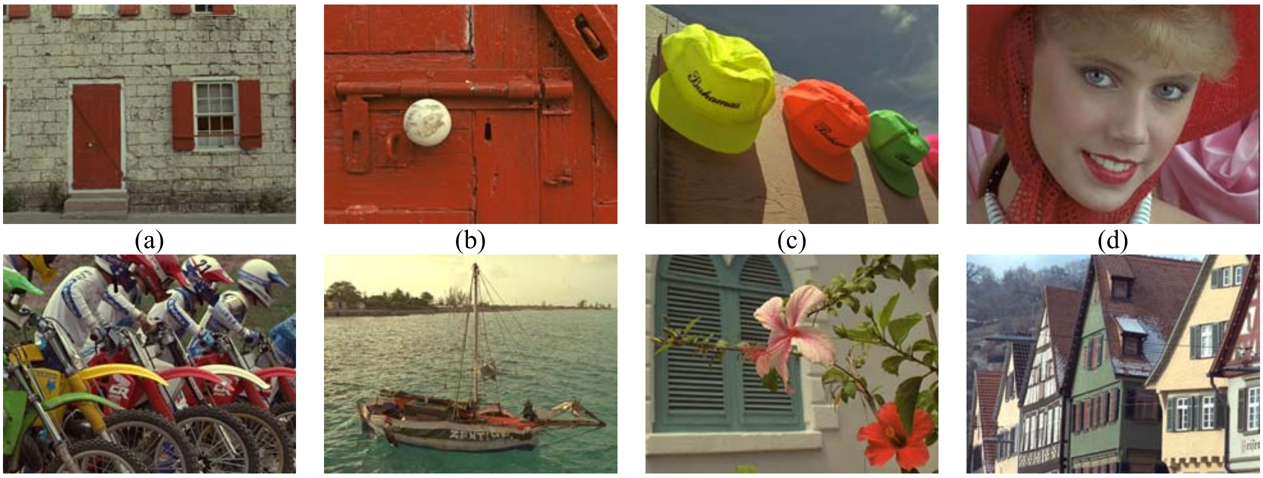

(a) (b) (c) (d)
(e) (f) (g) (h)

Fig. 3: Eight reference images used for the parameter tuning process. They are extracted from the TID2008 database.

It should be noted that the FSIM/FSIM$_C$ indices will be most effective if used on the appropriate scale. The precisely "right" scale depends on both the image resolution and the viewing distance and hence is difficult to be obtained. In practice, we used the following empirical steps proposed by Wang [30] to determine the scale for images viewed from a typical distance: 1) let $F = \max(1, \text{round}(N / 256))$, where N is the number of pixels in image height or width; 2) average local $F \times F$ pixels and then down-sample the image by a factor of F.

C. Gradient operator selection

TABLE III. SROCC VALUES USING THREE GRADIENT OPERATORS

Database	SROCC
Sobel	0.8797
Prewitt	0.8776
Scharr	0.8825

In our proposed IQA metrics FSIM/FSIM$_C$, the gradient magnitude (GM) needs to be calculated. To this end, three commonly used gradient operators listed in Table I were examined, and the one providing the best

result was selected. Such a gradient operator selection process was carried out by assuming that all the parameters discussed in Section IV-B were fixed. The selection criterion was also that the gradient operator leading to a higher SROCC would be selected. The sub-dataset used in Section IV-B was used here. The SROCC values obtained by the three gradient operators on the tuning dataset are listed in Table III, from which we can see that the Scharr operator could achieve slightly better performance than the other two. Thus, in all of the following experiments, the Scharr operator was used to calculate the gradient in FSIM/FSIM$_C$.

D. Example to demonstrate the effectiveness of FSIM/FSIM$_C$

In this subsection, we use an example to demonstrate the effectiveness of FSIM/FSIM$_C$ in evaluating the perceptible image quality. Fig. 4a is the I17 reference image in the TID2008 database, and Figs. 4b~4f show five distorted images of I17: I17_01_2, I17_03_2, I17_09_1, I17_11_2, and I17_12_2. We compute the image quality of Figs. 4b ~ 4f using various IQA metrics and the results are summarized in Table IV. We also list the subjective scores (extracted from TID2008) of these 5 images in Table IV. For each IQA metric and the subjective evaluation, higher scores mean higher image quality.

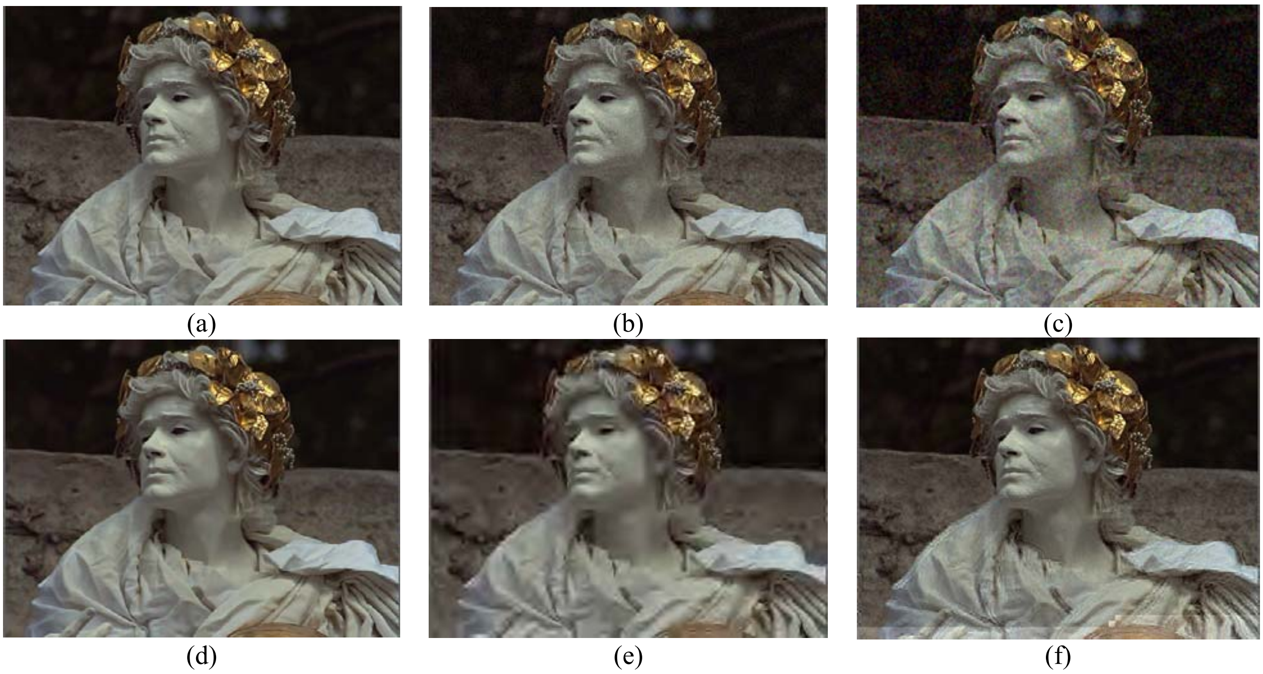

Fig. 4: (a) A reference image; (b) ~ (f) are the distorted versions of (a) in the TID2008 database.

In order to show the correlation of each IQA metric with the subjective evaluation more clearly, in Table V, we rank the images according to their quality scores computed by each metric as well as the subjective evaluation. From Tables IV and V, we can see that the quality scores computed by FSIM/FSIM$_C$ correlate with the subjective evaluation much better than the other IQA metrics. From Table V we can also see that other than the proposed FSIM/FSIM$_C$ metrics, all the other IQA metrics cannot give the same ranking as the subjective evaluations.

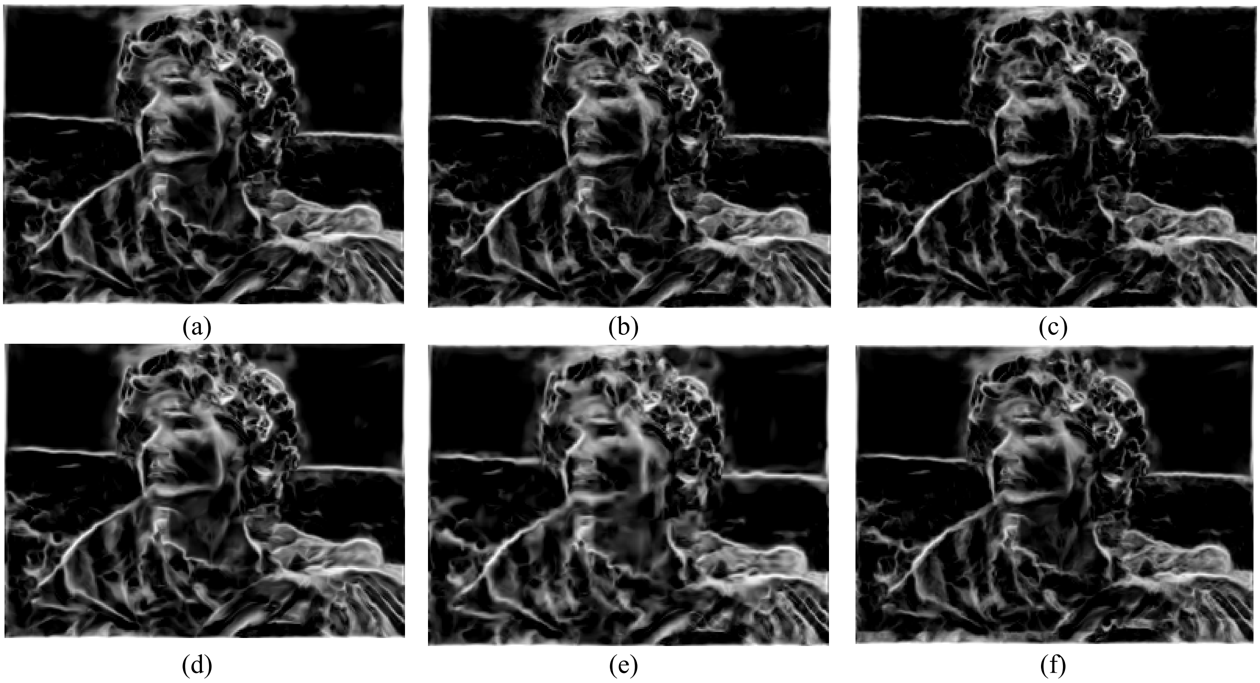

Fig. 5: (a) ~ (f) are PC maps extracted from images Figs. 4a ~ 4f, respectively.

TABLE IV. QUALITY EVALUATION OF IAMGES IN Fig. 4

	Fig. 4b	Fig. 4c	Fig. 4d	Fig. 4e	Fig. 4f
Subjective score	5.2222	4.0571	6.1389	3.3429	5.2000
FSIM	0.9776	0.9281	0.9827	0.9085	0.9583
FSIM$_C$	0.9741	0.9195	0.9817	0.9071	0.9582
MS-SSIM	0.9590	0.9109	0.9832	0.9170	0.9633
VIF	0.5803	0.4037	0.5670	0.1876	0.6484
SSIM	0.9268	0.8419	0.9686	0.8464	0.9306
IFC	3.5416	2.3441	3.9036	1.1710	8.0318
VSNR	30.7669	22.6702	32.2891	21.1772	18.6739
NQM	29.5608	21.0153	29.4932	19.4725	16.3083
[16]	0.8790	0.7158	0.9110	0.6503	0.9172
PSNR	27.1845	27.1577	34.0126	27.0330	26.9246

TABLE V. RANKING OF IMAGES ACCORDING TO THEIR QUALITY COMPUTED BY EACH IQA METRIC

	Fig. 4b	Fig. 4c	Fig. 4d	Fig. 4e	Fig. 4f
Subjective score	2	4	1	5	3
FSIM	2	4	1	5	3
$FSIM_C$	2	4	1	5	3
MS-SSIM	3	5	1	4	2
VIF	2	4	3	5	1
SSIM	3	5	1	4	2
IFC	3	4	2	5	1
VSNR	2	3	1	4	5
NQM	1	3	2	4	5
[16]	3	4	2	5	1
PSNR	2	3	1	4	5

The success of FSIM/$FSIM_C$ actually owes to the proper use of PC maps. Figs. 5a ~ 5f show the PC maps of the images in Figs. 4a ~ 4f, respectively. We can see that images in Figs. 4b and 4d have better perceptible qualities than those in Figs. 4c and 4e; meanwhile, by visually examination we can see that maps in Figs. 5b and 5d (PC maps of images in Figs. 4b and 4d) are more similar to the map in Fig. 5a (PC map of the reference image in Fig. 4a) than the maps in Figs. 5c and 5e (PC maps of images in Fig. 4c and 4e). This example clearly illustrates that images of higher quality will have more similar PC maps to that of the reference image than images of lower quality. Therefore, by properly making use of PC maps in FSIM/$FSIM_C$, we can predict the image quality consistently with human subjective evaluations. More statistically convincing results will be presented in the next two sub-sections.

E. Overall performance comparison

In this section, we compare the general performance of the competing IQA metrics. Table VI lists the SROCC, KROCC, PLCC, and RMSE results of FSIM/$FSIM_C$ and the other 8 IQA algorithms on the TID2008, CSIQ, LIVE, IVC, MICT, and A57 databases. For each performance measure, the three IQA indices producing the best results are highlighted in boldface for each database. It should be noted that except for $FSIM_C$, all the other IQA indices are based on the luminance component of the image. From Table VI, we can see that the proposed feature-similarity based IQA metric FSIM or $FSIM_C$ performs consistently well across all the databases. In order to demonstrate this consistency more clearly, in Table VII we list the

performance ranking of all the IQA metrics according to their SROCC values. For fairness, the $FSIM_C$ index, which also exploits the chrominance information of images, is excluded in Table VII.

TABLE VI: PERFORMANCE COMPARISON OF IQA METRICS ON 6 BENCHMARK DATABASES

		FSIM	$FSIM_C$	MS-SSIM	VIF	SSIM	IFC	VSNR	NQM	[16]	PSNR
TID 2008	SROCC	**0.8805**	**0.8840**	**0.8528**	0.7496	0.7749	0.5692	0.7046	0.6243	0.7388	0.5245
	KROCC	**0.6946**	**0.6991**	**0.6543**	0.5863	0.5768	0.4261	0.5340	0.4608	0.5414	0.3696
	PLCC	**0.8738**	**0.8762**	**0.8425**	0.8090	0.7732	0.7359	0.6820	0.6135	0.7679	0.5309
	RMSE	**0.6525**	**0.6468**	**0.7299**	0.7888	0.8511	0.9086	0.9815	1.0598	0.8595	1.1372
CSIQ	SROCC	**0.9242**	**0.9310**	0.9138	**0.9193**	0.8756	0.7482	0.8106	0.7402	0.7642	0.8057
	KROCC	**0.7567**	**0.7690**	0.7397	**0.7534**	0.6907	0.5740	0.6247	0.5638	0.5811	0.6080
	PLCC	**0.9120**	**0.9192**	0.8998	**0.9277**	0.8613	0.8381	0.8002	0.7433	0.8222	0.8001
	RMSE	**0.1077**	**0.1034**	0.1145	**0.0980**	0.1334	0.1432	0.1575	0.1756	0.1494	0.1575
LIVE	SROCC	**0.9634**	**0.9645**	0.9445	**0.9631**	0.9479	0.9234	0.9274	0.9086	0.8650	0.8755
	KROCC	**0.8337**	**0.8363**	0.7922	**0.8270**	0.7963	0.7540	0.7616	0.7413	0.6781	0.6864
	PLCC	**0.9597**	**0.9613**	0.9430	**0.9598**	0.9449	0.9248	0.9231	0.9122	0.8765	0.8721
	RMSE	**7.6780**	**7.5296**	9.0956	**7.6734**	8.9455	10.392	10.506	11.193	13.155	13.368
IVC	SROCC	**0.9262**	**0.9293**	0.8847	0.8966	**0.9018**	0.8978	0.7983	0.8347	0.8383	0.6885
	KROCC	**0.7564**	**0.7636**	0.7012	0.7165	**0.7223**	0.7192	0.6036	0.6342	0.6441	0.5220
	PLCC	**0.9376**	**0.9392**	0.8934	0.9028	**0.9119**	0.9080	0.8032	0.8498	0.8454	0.7199
	RMSE	**0.4236**	**0.4183**	0.5474	0.5239	**0.4999**	0.5105	0.7258	0.6421	0.6507	0.8456
MICT	SROCC	**0.9059**	**0.9067**	0.8864	**0.9086**	0.8794	0.8387	0.8614	0.8911	0.6923	0.6130
	KROCC	**0.7302**	**0.7303**	0.7029	**0.7329**	0.6939	0.6413	0.6762	0.7129	0.5152	0.4447
	PLCC	**0.9078**	**0.9075**	0.8935	**0.9144**	0.8887	0.8434	0.8710	0.8955	0.7208	0.6426
	RMSE	**0.5248**	**0.5257**	0.5621	**0.5066**	0.5738	0.6723	0.6147	0.5569	0.8674	0.9588
A57	SROCC	**0.9181**	-	**0.8394**	0.6223	0.8066	0.3185	**0.9355**	0.7981	0.7155	0.6189
	KROCC	**0.7639**	-	**0.6478**	0.4589	0.6058	0.2378	**0.8031**	0.5932	0.5275	0.4309
	PLCC	**0.9252**	-	**0.8504**	0.6158	0.8017	0.4548	**0.9472**	0.8020	0.7399	0.6587
	RMSE	**0.0933**	-	**0.1293**	0.1936	0.1469	0.2189	**0.0781**	0.1468	0.1653	0.1849

TABLE VII: RANKING OF IQA METRICS' PERFORMANCE (EXCEPT FOR $FSIM_C$) ON SIX DATABASES

	TID2008	CSIQ	LIVE	IVC	MICT	A57
FSIM	**1**	**1**	**1**	**1**	2	2
MS-SSIM	2	3	4	5	4	3
VIF	4	2	2	4	**1**	7
SSIM	3	4	3	2	5	4
IFC	8	8	6	3	7	9
VSNR	6	5	5	8	6	**1**
NQM	7	9	7	7	3	5
[16]	5	7	9	6	8	6
PSNR	9	6	8	9	9	8

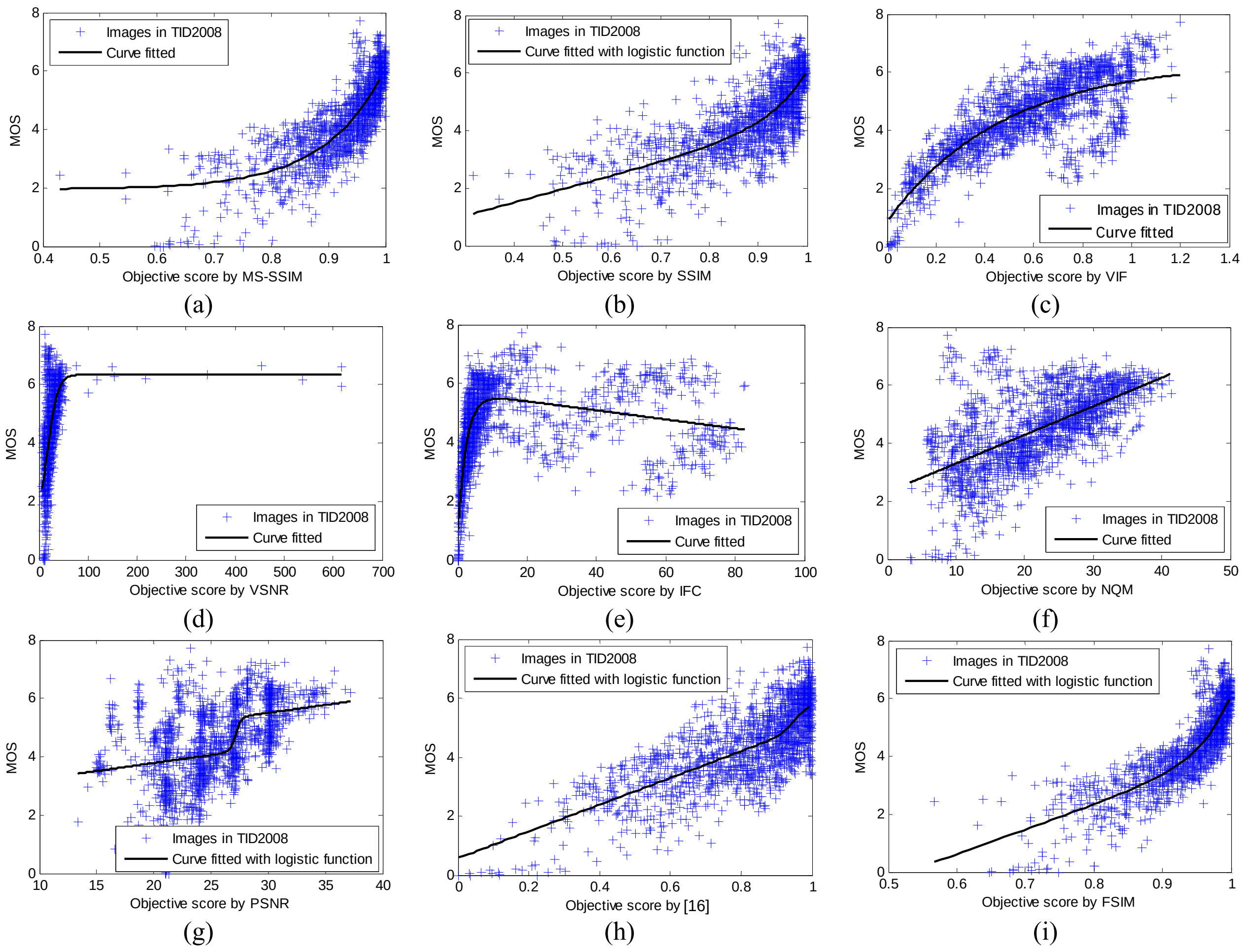

Fig. 6: Scatter plots of subjective MOS versus scores obtained by model prediction on the TID 2008 database. (a) MS-SSIM; (b) SSIM; (c) VIF; (d) VSNR; (e) IFC; (f) NQM; (g) PSNR; (h) method in [16] and (i) FSIM.

From the experimental results summarized in Table VI and Table VII, we can see that our methods achieve the best results on almost all the databases, except for MICT and A57. Even on these two databases, however, the proposed FSIM (or $FSIM_C$) is only slightly worse than the best results. Moreover, considering the scales of the databases, including the number of images, the number of distortion types, and the number of observers, we think that the results obtained on TID2008, CSIQ, LIVE and IVC are much more convincing than those obtained on MICT and A57. Overall, FSIM and $FSIM_C$ achieve the most consistent and stable performance across all the 6 databases. By contrast, for the other methods, they may work well on some databases but fail to provide good results on other databases. For example, although VIF can get very pleasing results on LIVE, it performs poorly on TID2008 and A57. The experimental results also demonstrate that the chromatic information of an image does affect its perceptible quality since $FSIM_C$ has better performance than FSIM on all color image databases. Fig. 6 show the scatter distributions of

subjective MOS versus the predicted scores by FSIM and the other 8 IQA indices on the TID 2008 database. The curves shown in Fig. 6 were obtained by a nonlinear fitting according to Eq. (12). From Fig. 6, one can see that the objective scores predicted by FSIM correlate much more consistently with the subjective evaluations than the other methods.

F. Performance on individual distortion types

TABLE VIII: SROCC VALUES OF IQA METRICS FOR EACH DISTORTION TYPE

		FSIM	$FSIM_C$	MS-SSIM	VIF	SSIM	IFC	VSNR	NQM	[16]	PSNR
	awgn	0.8566	**0.8758**	0.8094	**0.8799**	0.8107	0.5817	0.7728	0.7679	0.5069	**0.9114**
	awgn-color	0.8527	**0.8931**	0.8064	**0.8785**	0.8029	0.5528	0.7793	0.7490	0.4625	**0.9068**
	spatial corr-noise	0.8483	**0.8711**	0.8195	**0.8703**	0.8144	0.5984	0.7665	0.7720	0.6065	**0.9229**
	masked noise	0.8021	**0.8264**	0.8155	**0.8698**	0.7795	0.7326	0.7295	0.7067	0.5301	**0.8487**
	high-fre-noise	**0.9093**	**0.9156**	0.8685	0.9075	0.8729	0.7361	0.8811	0.9015	0.6935	**0.9323**
	impulse noise	0.7452	**0.7719**	0.6868	**0.8331**	0.6732	0.5334	0.6471	0.7616	0.4537	**0.9177**
	quantization noise	**0.8564**	**0.8726**	0.8537	0.7956	0.8531	0.5911	0.8270	0.8209	0.6214	**0.8699**
	blur	0.9472	0.9472	**0.9607**	**0.9546**	**0.9544**	0.8766	0.9330	0.8846	0.8883	0.8682
TID 2008	denoising	**0.9603**	**0.9618**	**0.9571**	0.9189	0.9530	0.8002	0.9286	0.9450	0.7878	0.9381
	jpg-comp	**0.9279**	**0.9294**	**0.9348**	0.9170	0.9252	0.8181	0.9174	0.9075	0.8186	0.9011
	jpg2k-comp	**0.9773**	**0.9780**	**0.9736**	0.9713	0.9625	0.9445	0.9515	0.9532	0.9301	0.8300
	jpg-trans-error	**0.8708**	**0.8756**	**0.8736**	0.8582	0.8678	0.7966	0.8056	0.7373	0.8334	0.7665
	jpg2k-trans-error	**0.8544**	**0.8555**	0.8525	0.8510	**0.8577**	0.7303	0.7909	0.7262	0.7164	0.7765
	pattern-noise	0.7491	0.7514	0.7336	**0.7608**	0.7107	**0.8410**	0.5716	0.6800	**0.7677**	0.5931
	block-distortion	**0.8492**	**0.8464**	0.7617	0.8320	**0.8462**	0.6767	0.1926	0.2348	0.7282	0.5852
	mean shift	0.6720	0.6554	**0.7374**	0.5132	**0.7231**	0.4375	0.3715	0.5245	0.3487	**0.6974**
	contrast	**0.6481**	**0.6510**	0.6400	**0.8190**	0.5246	0.2748	0.4239	0.6191	0.3883	0.6126
	awgn	0.9262	0.9359	**0.9471**	**0.9571**	0.8974	0.8460	0.9241	**0.9384**	0.7501	0.9363
	jpg-comp	**0.9654**	**0.9664**	0.9622	**0.9705**	0.9546	0.9395	0.9036	0.9527	0.9088	0.8882
	jpg2k-comp	**0.9685**	**0.9704**	**0.9691**	0.9672	0.9606	0.9262	0.9480	0.9631	0.8886	0.9363
CSIQ	1/f noise	0.9234	**0.9370**	0.9330	**0.9509**	0.8922	0.8279	0.9084	0.9119	0.7905	**0.9338**
	blur	**0.9729**	**0.9729**	0.9720	**0.9747**	0.9609	0.9593	0.9446	0.9584	0.9551	0.9289
	contrast	0.9420	**0.9438**	**0.9521**	0.9361	0.7922	0.5416	0.8700	**0.9479**	0.4326	0.8622
	jpg2k-comp	**0.9717**	**0.9724**	0.9654	**0.9683**	0.9614	0.9100	0.9551	0.9435	0.8533	0.8954
	jpg-comp	**0.9834**	**0.9840**	0.9793	**0.9842**	0.9764	0.9440	0.9657	0.9647	0.9127	0.8809
LIVE	awgn	0.9652	0.9716	0.9731	**0.9845**	0.9694	0.9377	0.9785	**0.9863**	0.9079	**0.9854**
	blur	**0.9708**	**0.9708**	0.9584	**0.9722**	0.9517	0.9649	0.9413	0.8397	0.9365	0.7823
	jpg2k-trans-error	0.9499	0.9519	0.9321	**0.9652**	**0.9556**	**0.9644**	0.9027	0.8147	0.8765	0.8907

In this experiment, we examined the performance of the competing methods on different image distortion types. We used the SROCC score, which is a widely accepted and used evaluation measure for IQA metrics [1, 32], as the evaluation measure. By using the other measures, such as KROCC, PLCC, and RMSE, similar conclusions could be drawn. The three largest databases, TID2008, CSIQ and LIVE, were used in this experiment. The experimental results are summarized in Table VIII. For each database and each distortion type, the first 3 IQA indices producing the highest SROCC values are highlighted in boldface. We can have some observations based on the results listed in Table VIII. In general, when the distortion type is known beforehand, FSIM_C performs the best, while FSIM and VIF have comparable performance. FSIM, FSIM_C and VIF perform much better than the other IQA indices. Compared with VIF, FSIM and FSIM_C are more capable in dealing with the distortions of "denoising", "quantization noise", and "mean shift". By contrast, for the distortions of "masked noise" and "impulse noise", VIF performs better than FSIM and FSIM_C. Moreover, results in Table VIII once again corroborates that the chromatic information does affect the perceptible quality since FSIM_C has better performance than FSIM on each database for nearly all the distortion types.

V. CONCLUSIONS

In this paper, we proposed a novel low-level feature based image quality assessment (IQA) metric, namely Feature-SIMilarity (FSIM) index. The underlying principle of FSIM is that HVS perceives an image mainly based on its salient low-level features. Specifically, two kinds of features, the phase congruency (PC) and the gradient magnitude (GM), are used in FSIM, and they represent complementary aspects of the image visual quality. The PC value is also used to weight the contribution of each point to the overall similarity of two images. We then extended FSIM to FSIM_C by incorporating the image chromatic features into consideration. The FSIM and FSIM_C indices were compared with eight representative and prominent IQA metrics on six benchmark databases, and very promising results were obtained by FSIM and FSIM_C. When the distortion type is known beforehand, FSIM_C performs the best while FSIM achieves comparable performance with VIF. When all the distortion types are involved (i.e. all the images in a test database are used), FSIM and FSIM_C outperform all the other IQA metrics used in comparison. Particularly, they perform consistently well across all the test databases, validating that they are very robust IQA metrics.

REFERENCES

[1] Z. Wang, A.C. Bovik, H.R. Sheikh, and E.P. Simoncelli, "Image quality assessment: from error visibility to structural similarity", *IEEE Trans. Image Process.*, vol. 13, no. 4, pp. 600-612, Apr. 2004.

[2] N. Damera-Venkata, T.D. Kite, W.S. Geisler, B.L. Evans, and A.C. Bovik, "Image quality assessment based on a degradation model", *IEEE Trans. Image Process.*, vol. 9, no. 4, pp. 636-650, Apr. 2000.

[3] D.M. Chandler and S.S. Hemami, "VSNR: a wavelet-based visual signal-to-noise ratio for natural images", *IEEE Trans. Image Process.*, vol. 16, no. 9, pp. 2284-2298, Sep. 2007.

[4] H.R. Sheikh and A.C. Bovik, "Image information and visual quality", *IEEE Trans. Image Process.*, vol. 15, no. 2, pp. 430-444, Feb. 2006.

[5] Z. Wang, E.P. Simoncelli, and A.C. Bovik, "Multi-scale structural similarity for image quality assessment", presented at the IEEE Asilomar Conf. Signals, Systems and Computers, Nov. 2003.

[6] C. Li and A.C. Bovik, "Three-component weighted structural similarity index", in *Proc. SPIE*, vol. 7242, 2009.

[7] H.R. Sheikh, A.C. Bovik, and G. de Veciana, "An information fidelity criterion for image quality assessment using natural scene statistics", *IEEE Trans. Image Process.*, vol. 14, no. 12, pp. 2117-2128, Dec. 2005.

[8] M.P. Sampat, Z. Wang, S. Gupta, A.C. Bovik, and M.K. Markey, "Complex wavelet structural similarity: a new image similarity index", *IEEE Trans. Image Process.*, vol. 18, no. 11, pp. 2385-2401, Nov. 2009.

[9] D. Marr, *Vision*. New York: W. H. Freeman and Company, 1980.

[10] D. Marr and E. Hildreth, "Theory of edge detection", *Proc. R. Soc. Lond. B*, vol. 207, no. 1167, pp. 187-217, Feb. 1980.

[11] M.C. Morrone and D.C. Burr, "Feature detection in human vision: a phase-dependent energy model", *Proc. R. Soc. Lond. B*, vol. 235, no. 1280, pp. 221-245, Dec. 1988.

[12] M.C. Morrone, J. Ross, D.C. Burr, and R. Owens, "Mach bands are phase dependent", *Nature*, vol. 324, no. 6049, pp. 250-253, Nov. 1986.

[13] M.C. Morrone and R.A. Owens, "Feature detection from local energy", *Pattern Recognition Letters*, vol. 6, no. 5, pp. 303-313, Dec. 1987.

[14] P. Kovesi, "Image features from phase congruency", *Videre: J. Comp. Vis. Res.*, vol. 1, no. 3, pp. 1-26, 1999.

[15] L. Henriksson, A. Hyvärinen, and S. Vanni, "Representation of cross-frequency spatial phase relationships in human visual cortex", *J. Neuroscience*, vol. 29, no. 45, pp. 14342-14351, Nov. 2009.

[16] Z. Liu and R. Laganière, "Phase congruence measurement for image similarity assessment", *Pattern Recognition Letters*, vol. 28, no. 1, pp. 166-172, Jan. 2007.

[17] Z. Wang and E.P. Simoncelli, "Local phase coherence and the perception of blur", in *Adv. Neural Information Processing Systems.*, 2004, pp. 786-792.

[18] R. Hassen, Z. Wang, and M. Salama, "No-reference image sharpness assessment based on local phase coherence measurement", in *Proc. IEEE Int. Conf. Acoust., Speech, and Signal Processing*, 2010, pp. 2434-2437.

[19] D. Gabor, "Theory of communication", *J. Inst. Elec. Eng.*, vol. 93, no. III, pp. 429-457, 1946.

[20] D. J. Field, "Relations between the statistics of natural images and the response properties of cortical cells", *J. Opt. Soc. Am. A*, vol. 4, no. 12, pp. 2379-2394, Dec. 1987.

[21] R. Jain, R. Kasturi, and B.G. Schunck, *Machine Vision*. McGraw-Hill, Inc, 1995.

[22] B. Jähne, H. Haubecker, and P. Geibler, *Handbook of Computer Vision and Applications*. Academic Press, 1999.

[23] C. Yang and S.H. Kwok, "Efficient gamut clipping for color image processing using LHS and YIQ", *Optical Engineering*, vol. 42, no. 3, pp.701-711, Mar. 2003.

[24] N. Ponomarenko, V. Lukin, A. Zelensky, K. Egiazarian, M. Carli, and F. Battisti, "TID2008 - A database for evaluation of full-reference visual quality assessment metrics", *Advances of Modern Radioelectronics*, vol. 10, pp. 30-45, 2009.

[25] E.C. Larson and D.M. Chandler, "Categorical Image Quality (CSIQ) Database", http://vision.okstate.edu/csiq.

[26] H.R. Sheikh, K. Seshadrinathan, A.K. Moorthy, Z. Wang, A.C. Bovik, and L.K. Cormack, "Image and video quality assessment research at LIVE", http://live.ece.utexas.edu/research/quality.

[27] A. Ninassi, P. Le Callet, and F. Autrusseau, "Subjective quality assessment-IVC database", http://www2.irccyn.ec-nantes.fr/ivcdb.

[28] Y. Horita, K. Shibata, Y. Kawayoke, and Z.M. Parves Sazzad, "MICT Image Quality Evaluation Database", http://mict.eng.u-toyama.ac.jp/mict/index2.html.

[29] D.M. Chandler and S.S. Hemami, "A57 database", http://foulard.ece.cornell.edu/dmc27/vsnr/vsnr.html.

[30] Z. Wang, "SSIM Index for Image Quality Assessment", http://www.ece.uwaterloo.ca/~z70wang/research/ssim/.

[31] M. Gaubatz and S.S. Hemami, "MeTriX MuX Visual Quality Assessment Package", http://foulard.ece.cornell.edu/gaubatz/metrix_mux.

[32] VQEG, "Final report from the video quality experts group on the validation of objective models of video quality assessment", http://www.vqeg.org, 2000.

[33] H.R. Sheikh, M.F. Sabir, and A.C. Bovik, "A statistical evaluation of recent full reference image quality assessment algorithms", *IEEE Trans. Image Process.*, vol. 15, no. 11, pp. 3440-3451, Nov. 2006.
