# Supplementary material for: Advancing image segmentation with DBO-Otsu: Addressing rubber tree diseases through enhanced threshold techniques
Source: PLoS One. 2024 Mar 21;19(3):e0297284. doi: 10.1371/journal.pone.0297284 (PMC10956860; doi:10.1371/journal.pone.0297284)
Supplement: Attachment — Submitted filename: review_report_PONE-D-23-30775.pdf [file pone.0297284.s013.pdf]

Review Report

Manuscript Title: Advancing Image Segmentation with DBO-Otsu: Addressing Rubber Tree Diseases through Enhanced Threshold Techniques

Manuscript No.: PONE-D-23-30775

Journal: PLOS ONE

This paper presents a multi-level thresholding image segmentation model, DBO-Otsu, designed to address the need for precise classification and early diagnosis of Rubber Tree Top Wilt Disease. DBO-Otsu, based on Dung Beetle Optimization, outperforms standard Otsu methods, especially in high grayscale areas. The proposed DBO-Otsu is evaluated over three images from the rubber dataset as benchmark images. Comparative analysis with six other meta-heuristic Otsu algorithms shows a notable 10% enhancement in the Structural Similarity Index.

The paper highlights the utilization of image segmentation methods as a preprocessing step in the field of medical imaging, focusing on a timely and critical issue related to healthcare diagnostics.

The reviewer appreciates the effort invested in this manuscript. While the paper exhibits notable merits, there are significant improvements that should be considered before the paper becomes suitable for publication. Therefore, the reviewer recommends addressing these additional revisions.

1) Major comments:

Introduction:

The introduction section, as it stands, is not satisfactory and should be reviewed again. It lacks the necessary cohesion flow and clarity of ideas. To address these issues, I recommend the following revisions and clarifications:

1. Begin the introduction with a brief overview of the Rubber Tree Top Wilt Disease and how cutting-edge AI techniques and computer vision play a pivotal role in identifying and understanding this disease. This will allow you to smoothly introduce image segmentation as a preprocessing step in medical imaging, setting the foundation for the subsequent discussion.
2. The authors mentioned that (traditional Otsu algorithms often face challenges in complex scenarios) Please clearly highlight the limitations of traditional Otsu algorithms in complex scenarios, to underscore the motivation for adopting metaheuristics as an alternative approach.
3. Introduce thresholding-based techniques, emphasizing their popularity and relevance as similarity-based methods in the context of your research.
4. Provide a more detailed explanation for the selection of Dung Beetle Optimization as the chosen metaheuristic approach and discuss why it was preferred over other available metaheuristics.
5. It is difficult to discern the unique contributions of the proposed model. It would be beneficial to highlight the contributions in bullet points. This would provide readers with a concise and structured overview of the specific advancements by the proposed model.

Inadequate literature review

1. discussion of related works should be expanded with more recent metaheuristics-based image segmentation studies to highlight the limitations of the existing literature, situate the contribution, establish the research gap and highlight the novelty of the proposed approach.
2. Some recent related papers that could be included:

Chen, Jiaochen & Cai, Zhen-Nao & Heidari, Ali Asghar & Liu, Lei & Chen, Huiling & Pan, Jingye. (2023). Dynamic mechanism-assisted artificial bee colony optimization for image segmentation of COVID-19 chest X-ray. Displays. 79. 102485. 10.1016/j.displa.2023.102485.

[Su et al., 2022] Su, H., Zhao, D., Elmannai, H., Heidari, A. A., Bourouis, S., Wu, Z., Cai, Z.-N., Gui, W., and Chen, M. (2022). Multilevel threshold image segmentation for covid-19 chest radiography: A framework using horizontal and vertical multiverse optimization. *Computers in Biology and Medicine*, 146:105618.

Sabha M, Thaher T, Emam MM (2023) Cooperative Swarm Intelligence Algorithms for Adaptive Multilevel Thresholding Segmentation of COVID-19 CT-Scan Images. JUCS - Journal of Universal Computer Science 29(7): 759-804. https://doi.org/10.3897/jucs.93498

[Abdel-Basset et al., 2021] Abdel-Basset, M., Chang, V., and Mohamed, R. (2021). A novel equilibrium optimization algorithm for multi-thresholding image segmentation problems. Neural Computing and Applications, 33:10685–10718.

[Abualigah et al., 2021b] Abualigah, L., Diabat, A., Sumari, P., and Gandomi, A. H. (2021b). A novel evolutionary arithmetic optimization algorithm for multilevel thresholding segmentation of covid-19 ct images. *Processes*, 9(7).

[Chen et al., 2022] Chen, Y., Wang, M., Heidari, A. A., Shi, B., Hu, Z., Zhang, Q., Chen, H., Mafarja, M., and Turabieh, H. (2022). Multi-threshold image segmentation using a multi-strategy shuffled frog leaping algorithm. *Expert Systems with Applications*, 194:116511.

[Emam et al., 2022] Emam, M. M., Houssein, E. H., and Ghoniem, R. M. (2022). A modified reptile search algorithm for global optimization and image segmentation: Case study brain mri images. *Computers in Biology and Medicine*, page 106404.

[Liu et al., 2021] Liu, L., Zhao, D., Yu, F., Heidari, A. A., Li, C., Ouyang, J., Chen, H., Mafarja, M., Turabieh, H., and Pan, J. (2021). Ant colony optimization with cauchy and greedy levy mutations for multilevel covid 19 x-ray image segmentation. *Computers in Biology and Medicine*, 136:104609.

3. DBO-Otsu

1. Please expand the DBO-Otsu approach in Section 3, explaining how DBO is integrated with Otsu. Clarify the solution representation and fitness function. Consider using schematic views and visualizations for better clarity.

2. Section 3.1 is a critical component of the manuscript which represent the main contribution. Hence, it is essential to highlight and justify the proposed enhancements.

4. Experiments and analysis of results

1. It is not clear from the manuscript whether the parameter settings have been tuned specifically for your research or if they are adopted from recommended studies.
2. It is important to cite the utilized algorithms, specifying which versions of SSA, CSA, WOA, GWO, WSO, and AHA have been used in this research?
3. Please provide a brief description of the rubber dataset used as a benchmark in this study.
4. While the selection of three images from the rubber dataset is a good starting point, it's important to note that this sample size may not be sufficient to comprehensively verify the efficiency of the proposed model. It would be beneficial, if possible, to expand the testing to include a more substantial number of images to ensure robust evaluation
5. Please discuss the convergence curves of the proposed approach.

6. Inadequate analysis and discussion of results: The results are not thoroughly analyzed. A more in-depth analysis of the results, including statistical analysis is needed.
7. Statistical analysis is important to judge the significance of the findings.

Conclusion:

The conclusion should be explored better and it needs to contemplate the eventual restrictions of the developed technique to address future works in this area.

2) Minor notes:

- Please verify that acronym definitions are provided upon their initial use in the manuscript
- Please enhance the quality of figures. it would be beneficial to consider converting them into vector-based formats
- Please check some mistakes:
  Error! Reference source not found. depicts the movement of rolling dung beetles,
  For illustrative purposes, let's assume a total population of 30 dung beetles. Using **Error! Reference source not found**.
