# Supplementary material for: Advancing image segmentation with DBO-Otsu: Addressing rubber tree diseases through enhanced threshold techniques
Source: PLoS One. 2024 Mar 21;19(3):e0297284. doi: 10.1371/journal.pone.0297284 (PMC10956860; doi:10.1371/journal.pone.0297284)
Supplement: Attachment — Submitted filename: review-report-R1.pdf [file pone.0297284.s015.pdf]

Review Report R1

Manuscript Title: Advancing Image Segmentation with DBO-Otsu: Addressing Rubber Tree Diseases through Enhanced Threshold Techniques

Manuscript No.: PONE-D-23-30775

Journal: PLOS ONE

I would like to thank authors for addressing the majority of my comments. However, some concerns still need further revision before the paper can be accepted.

1. The justification for selecting DBO over other MHs is still insufficient. It would be beneficial to emphasize the successful applications of DBO in the literature for addressing real-world problems. Additionally, please explicitly highlight the merits of DBO in comparison to other algorithms. Considering the problem-dependent nature of metaheuristics and referring to the principles of the free lunch theorem would enhance the justification. Please include references.

2. It is difficult to discern the unique contributions of the proposed model. While the integration of a recent DBO with Otsu is good, it does not constitute a novel contribution. In this regard, I recommend highlighting the unique enhancement to the traditional Otsu (presented is section 3.1) explicitly in the list of contributions.

3. The authors state "Providing a theoretical analysis of the DBO algorithm", but the presented work is primarily experimental.

4. Authors mentioned the use of an early convergence critera "During the iteration process, if the optimal threshold obtained is the same as the result of the first 5 iterations, the optimization process is considered to have converged and the iteration can be ended earlier, thus reducing the time consumption of the algorithm". It is unclear whether this approach is applied to other compared algorithms. Additionally, the rationale behind choosing 5 iterations as the threshold for convergence needs clarification.

5. Results in Table 2 does not demonstrate the superiority of the proposed approach in most cases. Authors mentioned that "In evaluating average PSNR, FSIM, and SSIM scores, DBO-OTSU consistently ranked at or near the top…" However, it is not clear if a specific ranking method was employed to support this claim. If so, kindly present the rank for each method. Utilizing a widely used ranking method like Friedman rank would be beneficial.

6. How do the authors justify the claim that, despite achieving better fitness scores, certain algorithms may not perform better in terms of other evaluation metrics like PSNR, FSIM, and SSIM?

7. Statistical analysis is important to judge the significance of the findings. In this comment I mean that Utilizing tests such as the Wilcoxon rank sum test would be beneficial to determine whether the differences presented in Table 2, such as those in PSNR (e.g., 8.762745 and 8.783505), are statistically significant or not."

8. Please verify that acronym definitions are provided upon their initial use in the manuscript and consistently use the acronym in subsequent parts.

Examples:

- Line 97 (GWO)
- Line(110) Whale Optimization Algorithm (use previously defined acronym)
- **Line (134)** Tapping Panel Dryness (acronym)

9. Please double check some mistakes
   Examples:
   - Line (75) HHO not HEO
   - Line (399): In Table 1, we present the selected thresholds and describe the division of the image into five regions based on these thresholds (you men Table 2)

10. Please enhance the quality of figures. (still need improvements, for instance the DBO flowchart …..)

11. Please review the order and placement of figures to ensure that captions are appropriately associated with the corresponding figures throughout the manuscript.
