# Supplementary material for: Advancing image segmentation with DBO-Otsu: Addressing rubber tree diseases through enhanced threshold techniques
Source: PLoS One. 2024 Mar 21;19(3):e0297284. doi: 10.1371/journal.pone.0297284 (PMC10956860; doi:10.1371/journal.pone.0297284)
Supplement: Attachment — Submitted filename: Response to Reviewers.docx [file pone.0297284.s016.docx]

Zhenjing Xie

Hainan University

Haikou, 570228

China

2023.11.28

Khan Bahadar Khan, Ph.D

Academic Editor

PLOS ONE

Dear Editor Khan Bahadar Khan,

Thank you and the reviewers for the review and recommendations on our manuscript (Title: "Advancing Image Segmentation with DBO-Otsu: Addressing Rubber Tree Diseases through Enhanced Threshold Techniques," Manuscript ID: PONE-D-23-30775). We have made detailed revisions based on the reviewer's feedback. Below is our response to the reviewer's comments:

1. Comment: The justification for selecting DBO over other MHs is still insufficient. It would be beneficial to emphasize the successful applications of DBO in the literature for addressing real-world problems. Additionally, please explicitly highlight the merits of DBO in comparison to other algorithms. Considering the problemdependent nature of metaheuristics and referring to the principles of the free lunch theorem would enhance the justification. Please include references.

Response: In response to your comment, Section 1.3 has been updated to include four recent publications featuring the application of the DBO optimization algorithm. These additions emphasize the suitability of the DBO algorithm for our research, particularly in terms of segmentation speed and accuracy, compared to other metaheuristic algorithms. Additionally, we have incorporated the 'No Free Lunch' theorem to acknowledge that no algorithm is universally perfect. This inclusion underscores the importance of selecting an algorithm that is most apt for the specific requirements of our study. Relevant references have been added to support these points.

2. Comment: It is difficult to discern the unique contributions of the proposed model. While the integration of a recent DBO with Otsu is good, it does not constitute a novel contribution. In this regard, I recommend highlighting the unique enhancement to the traditional Otsu (presented is section 3.1) explicitly in the list of contributions.
    Response: In light of your observation regarding the distinctiveness of our model's contributions, we have revised the final part of the Introduction to explicitly include the unique enhancements made to the traditional Otsu method in the list of contributions. Specifically, this update is reflected in the second point of the contributions section. This revision aims to clearly delineate our novel contribution, namely the integration of the recent DBO with Otsu, and its significance in advancing the methodology.
3. Comment: The authors state “Providing a theoretical analysis of the DBO algorithm”, but the presented work is primarily experimental.

Response: In response to your comment on the theoretical analysis of the DBO algorithm, we have updated the contribution section by removing the reference to 'Providing a theoretical analysis of the DBO algorithm.' Acknowledging the primarily experimental nature of our work, we agree that mentioning a theoretical analysis could be misleading. This revision ensures that the contributions listed are in alignment with the experimental focus of our research.

4. Comment: Authors mentioned the use of an early convergence critera “During the iteration process, if the optimal threshold obtained is the same as the result of the first 5 iterations, the optimization process is considered to have converged and the iteration can be ended earlier, thus reducing the time consumption of the algorithm”. It is unclear whether this approach is applied to other compared algorithms. Additionally, the rationale behind choosing 5 iterations as the threshold for convergence needs clarification.

Response: Regarding the early convergence criteria mentioned in our manuscript, we acknowledge that the theoretical basis of such criteria is detailed in the paper 'Effective Probabilistic Stopping Rules for Randomized Metaheuristics: GRASP Implementations.' However, it is important to note that this is not the focal point of our current study. The specific approach of terminating the iteration process after five identical results in our study was based on empirical experience and has minimal impact on the experimental outcomes. Consequently, we have decided to remove this content from the original manuscript. Furthermore, we clarify that this early termination method has not been applied to the other algorithms compared in our research, ensuring consistency in methodology across all tested approaches.

5. Comment: Results in Table 2 does not demonstrate the superiority of the proposed approach in most cases. Authors mentioned that “In evaluating average PSNR, FSIM, and SSIM scores, DBO-OTSU consistently ranked at or near the top…” However, it is not clear if a specific ranking method was employed to support this claim. If so, kindly present the rank for each method. Utilizing a widely used ranking method like Friedman rank would be beneficial.

Response: To address your concern regarding the demonstration of our approach's superiority in Table 2, we have conducted a comprehensive statistical analysis. We calculated the average rankings for each algorithm in terms of PSNR, FSIM, and SSIM scores. These rankings have been incorporated into Section 4.3 in a tabular format, accompanied by a brief explanation. This addition provides a clearer and more intuitive comparison among the algorithms, especially in scenarios where the differences in scores are not significantly pronounced. The use of average rankings offers a more structured way to assess the relative performance of each method, allowing for a fair and transparent comparison.

6. Comment: How do the authors justify the claim that, despite achieving better fitness scores, certain algorithms may not perform better in terms of other evaluation metrics like PSNR, FSIM, and SSIM?

Response: To justify our claim regarding the discrepancy between achieving better fitness scores and the performance in other evaluation metrics like PSNR, FSIM, and SSIM, we clarify that maximizing fitness, which is the inter-class variance in the mid-to-high threshold range, primarily focuses on enhancing the contrast between the foreground and background in grayscale. While this can improve segmentation effectiveness, it does not necessarily translate to higher overall image quality. PSNR, FSIM, and SSIM evaluate the segmented multi-threshold image against the original image. The application of multi-threshold Otsu's method might lead to loss of certain details in the image, particularly in high-contrast areas and regions with complex textures like tree barks, potentially lowering PSNR. FSIM considers image phase congruency and gradient magnitude to assess visual quality. However, the algorithm alters local features, especially at edges and textures, affecting the FSIM score. SSIM measures the structural, luminance, and contrast similarity between two images. The segmentation changes brought by the algorithm can alter local structures, thereby impacting the SSIM score.

However, this should not be construed as diminishing the relevance of PSNR, FSIM, and SSIM scores. These metrics provide a comprehensive assessment of the segmented multi-threshold images from a conventional perspective, complementing and corroborating our specialized evaluation approach.

7. Comment: Statistical analysis is important to judge the significance of the findings. In this comment I mean that Utilizing tests such as the Wilcoxon rank sum test would be beneficial to determine whether the differences presented in Table 2, such as those in PSNR (e.g., 8.762745 and 8.783505), are statistically significant or not."

Response: To address the need for statistical analysis to ascertain the significance of our findings, we have augmented Section 4.3 with a table presenting the results of the Wilcoxon signed-rank test. This test compares the DBO algorithm with other algorithms, using a predefined significance level of 0.1. The p-values obtained from this analysis have been duly recorded. The results demonstrate that the DBO algorithm shows statistically significant improvements in the PSNR and SSIM metrics. However, for the FSIM metric, the improvements were not found to be statistically significant. This additional analysis provides a robust statistical basis for validating the superiority of the DBO algorithm in specific aspects of our study.

8. Comment: Please verify that acronym definitions are provided upon their initial use in the manuscript and consistently use the acronym in subsequent parts.

Response: We have meticulously reviewed the entire manuscript in response to your comment regarding the use of acronyms. We have corrected the instances where acronyms were incorrectly used or not defined upon their initial use. This ensures that all acronyms are now properly introduced and consistently applied throughout the manuscript, enhancing its readability and clarity.

9. Comment: Please double check some mistakes

Response: In response to your comment regarding the presence of errors, we have thoroughly re-examined the manuscript and have identified and corrected the mistakes at the relevant locations. We appreciate your attention to detail and are committed to ensuring the accuracy and quality of our work.

10. Comment: Please enhance the quality of figures. (still need improvements, for instance the DBO flowchart …..)

Response: In response to your feedback on the quality of the figures, specifically mentioning the DBO flowchart, we have undertaken a comprehensive redesign of this figure to enhance its clarity and overall presentation. We hope the revised figure now meets your expectations in terms of visual quality and effectiveness in conveying the intended information.

11. Comment: Please review the order and placement of figures to ensure that captions are appropriately associated with the corresponding figures throughout the manuscript.

Response: In response to your comment on ensuring appropriate alignment of figure captions with their corresponding figures, we have conducted a thorough review of the entire manuscript. This review focused on verifying the order and placement of all figures to guarantee that each caption accurately reflects and is correctly associated with its respective figure. We have made necessary adjustments to ensure this consistency throughout the document.

We hope that these revisions meet the requirements of the reviewers and the editor, making our manuscript more suitable for publication. We look forward to your further guidance and feedback.

Sincerely,

Zhenjing Xie

Undergraduate Student

Hainan University

Haikou, 570228

China

20213005583@hainanu.edu.cn
